# Barriers and facilitators to healthcare facility utilization by non-Ebola patients during the 2018–2020 Ebola outbreak in the Democratic Republic of Congo

**DOI:** 10.1186/s41256-024-00387-6

**Published:** 2024-11-19

**Authors:** Gabriel Kalombe Kyomba, Michael Robert Law, Karen Ann Grépin, Serge Manitu Mayaka, Thérèse Nyangi-Mondo Mambu, Branly Kilola Mbunga, Celestin Hategeka, Mala Ali Mapatano, Joël Nkiama-Numbi Konde, Dosithée Ngo-Bebe, Pélagie Diambalula Babakazo, Eric Musalu Mafuta, Guillaume Mbela Kiyombo

**Affiliations:** 1 Kinshasa School of Public Health, Université de Kinshasa, Kinshasa, Democratic Republic of Congo; 2 Centre for Health Services and Policy Research, School of Population and Public Health, The University of British Columbia, Vancouver, BC Canada; 3 School of Public Health, University of Hong Kong, Pokfulam, Hong Kong SAR

**Keywords:** Healthcare facility, Health service utilization, Ebola Virus Disease, Preparedness and response, Free care policy, Personal protective equipment, North Kivu, Democratic Republic of Congo

## Abstract

**Background:**

An Ebola Virus Disease (EVD) outbreak occurred in North Kivu between 2018 and 2020. This eastern province of the Democratic Republic of Congo was also grappling with insecurity caused by several armed groups. This study aimed to explore the barriers and facilitators to utilizing Healthcare Facilities (HCFs) by non-Ebola patients during the crisis.

**Methods:**

A qualitative case study was conducted in Beni and Butembo with 24 relatives of 15 deceased non-EVD patients, 47 key informants from healthcare workers (HCWs), as well as community leaders. Semi-structured interviews were conducted to explore three key areas: (i) the participants’ illness history, care pathway, care, and social support; (ii) their perceptions of how EVD affected the care outcome; and (iii) their opinions on the preparedness, supply, use, and quality of healthcare before and during the outbreak. All interviews were recorded, transcribed verbatim, and thematically analysed using Atlas-ti 8.0.

**Results:**

Nine of the 15 deaths were female and their ages ranged from 7 to 79 years. The causes of death were non-communicable (13) or infectious (2) diseases. Conspiracy theories, failure to establish security, and the concept of the ''Ebola business'' were associated with misinformation and lower levels of trust in government and HCFs. The negative perceptions, fear of being identified as an Ebola case, apprehension about the triage unit, and inadequacy of personal protective equipment resulted in a preference for private or informal HCFs. For half of the deceased’s relatives, the Ebola outbreak hastened their death. Conversely, community involvement, employing familiar, neutral, and credible HCWs, and implementing a free care policy increased the number of visits. These results were observable despite a lack of funds, overstretched HCWs, and long waiting time.

**Conclusions:**

Our findings can inform policies before and during future outbreaks to enhance the resilience of routine HCFs by maintaining dialogue between HCWs and patients, and rebuilding confidence in HCFs. Quantitative studies including context analysis are essential to identify the determinants of care-seeking during such a crisis.

**Supplementary Information:**

The online version contains supplementary material available at 10.1186/s41256-024-00387-6.

## Background

Improving the supply and use of healthcare services is integral to strengthening health systems [1, 2]. However, several challenges, such as infectious disease outbreaks, can undermine these efforts [3, 4]. Indeed, the supply and use of healthcare services declined during outbreaks of SARS [5] and Ebola virus disease (EVD) in West Africa [6, 7]. Similar trends were observed at the onset of the Ebola outbreak in Equateur, located in the west of the Democratic Republic of Congo (DRC) [8], and of the COVID-19 pandemic [9, 10].

The socioeconomic effects of outbreaks also indirectly affect health systems [11]. People tend to avoid formal healthcare facilities (HCFs) due to fear of infection [12, 13], leading to decreased use of public HCFs [14–16]. Healthcare workers (HCWs) also experience fear, anxiety, frustration, and helplessness [17], resulting in reduced healthcare supply, and increased non-Ebola-specific mortality [18, 19].

The DRC had its tenth EVD outbreak between August 2018 and June 2020. It significantly affected the Ituri and North Kivu provinces, which were also grappling with several years of insecurity stemming from armed groups. These provinces experienced killings, rapes, and mass displacement, which exacerbated poverty and eroded trust in the government, which was charged with not taking sufficient action to ensure its residents’ safety. North Kivu province, which sustained the highest burden of the outbreak, reported 84% of 3470 cases and 88% of 2280 deaths.

Beyond the suffering, this outbreak presented opportunities. The government and its partners entirely and partially subsidised healthcare during the outbreak. This free care policy (FCP) aimed to improve EVD detection by removing financial barriers [20, 21]. The patient flow was organised by setting up triage and screening units. EVD diagnostic tests were deployed, and wearing personal protective equipment (PPE) became standard procedure among HCWs [22, 23]. In other situations, wearing and removing PPE slowed down healthcare processes and had psychological effects [3]. Similarly, implementing the FCP increased patient numbers [8, 24], which could affect healthcare quality [25].

During the outbreak of the EVD in West Africa from 2014 to 2016, its effects on HCFs were extensively documented. However, information is scarce regarding the impact on HCFs during the 2018–2020 EVD outbreak in DRC. Bardosh et al. [26] raised concerns about HCFs in Ebola-affected communities, yet there remains an inadequate understanding of the changes in HCWs' practices. Mutombo et al. [27] explored communities’ experience with EVD, the support received by the participants when facing EVD, and participants’ perceptions regarding control measures in Kivu and Ituri. Carter et al. [28] focused on patients with or suspected of Ebola disease in Sierra Leone. The objective of this study was to assess the factors influencing non-Ebola patients’ treatment choices and healthcare-seeking behaviour. A better understanding of these factors can contribute to strengthening the resilience of HCFs, which is imperative for achieving the third sustainable development goal [29, 30].

## Methods

### Study design, site, and study period

**Fig. 1:**
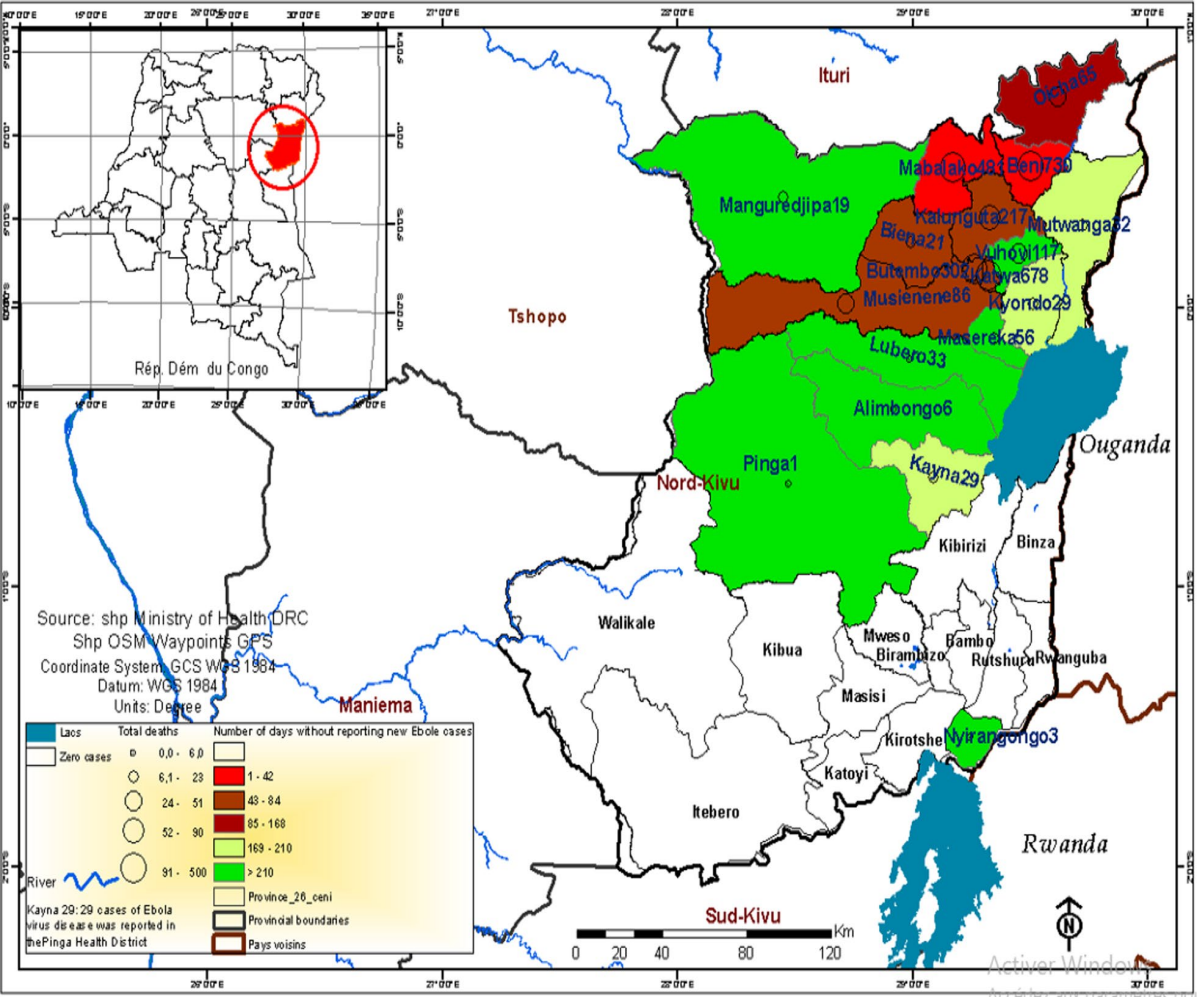
Spatial distribution of the reported cases of EVD by health districts on September 9th, 2019. [31] Source: Ministry of Health of RDC and WHO

A qualitative case study was conducted in Beni and Butembo, the two cities most affected by the 2018–2020 EVD outbreak (Fig. 1). The hospitals in both cities offered healthcare to patients referred from nearby health districts such as Mabalako (the location of the initial outbreak), Musienene, and others. Data were collected from March to June 2020.

### Tools and data sources

Data collection tools were developed based on Kroeger's framework as adapted by Chenge [32]. This model outlines three groups of factors that influence therapeutic choices. This includes patients’ demographic and socioeconomic characteristics, disease, and HCFs variables. Patient data were collected through medical record reviews and semi-structured interviews (SSIs) conducted with the deceased's relatives. Utilizing a clinical data extraction form and a pre-tested interview guide, information concerning the patient’s medical history, symptoms, treatment pathways, condition upon admission, care received, access to laboratory tests and medications, social support, and the impact of Ebola on treatment outcomes were documented. Additional SSIs were conducted with key informants (KIs), with a focus on their opinions on the preparedness, supply, use, and quality of HCFs before and during the EVD outbreak.

### Study population, sample size, and selection of participants

This study selected 15 deaths from HCFs. All deaths occurred due to non-Ebola illness. The research team believed that relatives could provide valuable insights into the effects of the outbreak on HCFs. The sample size was both large enough to encompass a diversity of patient profiles and small enough for in-depth analysis. The disease that led to death had to be clinically distinct from Ebola. This included non-communicable diseases, chronic infectious diseases such as HIV and tuberculosis, trauma, and obstetric emergencies. The deceased had to be admitted to HCFs for a minimum of 72 h within the 6 months preceding data collection (September 1, 2019, to February 29, 2020), to mitigate recall bias.

Purposive sampling combined with snowballing was used to select deceased relatives with as much information as possible about the deceased, their illness, and their treatment. Trained community guides established contact with the families and identified relatives who could handle the interview emotions (i.e. sadness, pain, …) and those who could not, related to the ethical principle of non-maleficence. Twenty-four relatives were included, representing one, two, or three relatives per deceased individual. Interviews with KIs were conducted to gain insights into the context and the claims made by relatives. KIs were purposively selected among HCWs, managers, and community leaders at provincial and local levels. Even though saturation was reached with approximately 20 KIs (indicated by the repetition of information and the absence of new themes), the number of interviews was up to 47 KIs to ensure a sufficient number of respondents in the sub-samples. These sub-samples included outbreak duration and severity, security context, the FCP modalities and content, and the respondents' function.

The SSIs were conducted in French and Swahili and were recorded on Dictaphone. SSIs lasted an average of 30 min with deceased relatives and nearly 40 min with KIs. Notes and non-verbal expressions were also taken. All interviews were private and conducted in a one-on-one setting, either at the participant’s home or workplace, except for one interview conducted by telephone.

### Data quality assurance

The principal investigator conducted the extraction of all clinical data and SSIs with KIs to ensure the validity of the results. An experienced, trained, and supervised assistant conducted the majority of SSIs with the deceased’s relatives. Triangulation methods involved comparing the deceased's relatives’ statements with medical records and aligning their opinions with those of KIs from various levels or affiliations. The medical records were prioritised as the primary for clinical characteristics and socio-demographic characteristics of the deceased in instances of inconsistencies. Other inconsistencies were treated as the respondents’ opinions. Finally, two members of the team separately carried out the analyses.

### Data analysis

Interviews were transcribed verbatim in French by experienced transcribers with a mastery of French (for interviews conducted in French) or both French and Swahili (for interviews conducted in Swahili). The principal investigator, who is fluent in both French and Swahili, carefully proofread the transcripts. The investigator carefully scrutinized any unsatisfactory translations with the transcribers to guarantee the highest level of accuracy. An independent third party conducted back-translation from French to Swahili, demonstrating strong equivalence for selected audios [33].

The transcripts were read several times, and the audios were listened to for familiarisation with their content. Atlas-ti 8.0 software was used for coding and conducting thematic analysis. This consisted of identifying, grouping, and examining systematically and objectively the themes that emerged from the theoretical framework and the study objectives. Additionally, new emerging themes were considered to reveal participants’ experiences, while maintaining a critical, neutral, and reflexive approach [34]. The sociodemographic characteristics of the deceased and their relatives, and KIs were entered and analysed using Excel software. Mean was reported for age and length of service and proportions for all categorical variables.

### Ethics statement

The Ethics Committee of the Kinshasa School of Public Health (n° ESP/CE/11/2020) approved this research project. Participation was voluntary, and confidentiality was ensured through one-on-one interviews and aggregated result presentations. Consent was obtained for the interview and recordings. No financial compensation was provided, but small gifts valued at less than 5 US dollars were offered to relatives in need.

## Results

### Social and medical characteristics of the deceased

Nine of the 15 deceased were female and their ages at death ranged from 7 to 79 years. The causes of death were non-communicable diseases (13) or infectious diseases (2). All cases died in hospital except two who died at home shortly after leaving hospital. Further details are presented in Table 1.

**Table 1:** Distribution of the deceased by site, and sociodemographic and medical characteristics

Characteristics	Number N = 15	%
*Gender*		
Male	6	40.0
Female	9	60.0
*Age (years old)*		
7 to 17	3	20.0
19 to 49	5	33.3
50 to 79	7	46.7
*Occupation*		
Agriculture	5	33.3
Small trade	5	33.3
Student	3	20.0
Other	2	13.3
*Cause of death*		
Prostate hypertrophy with or without nephropathy	2	13.3
Neurological diseases	3	20.0
Diabetes	2	13.3
High blood pressure	1	6.7
Cancer	2	13.3
Trauma	2	13.3
Measles	1	6.7
HIV/Tuberculosis	1	6.7
Pregnancy complication	1	6.7
*Death location*		
District hospital	8	53.3
Front line facility	5	33.3
Home shortly after a hospital stay	2	13.3

### Characteristics of the deceased’s relatives

Interviews were conducted with 24 relatives of the deceased, with one, two, or three relatives representing each death. The majority were female (17), had attended secondary school (10), or earned their living from small trade (11) or agriculture (5). Their ages ranged from 22 to 62 years with an average age of 43.7 years (standard deviation of 12.1 years). The relative's family ties with the deceased included parents (4), spouses (2), children (4), aunts (3), siblings/cousins (2), sisters-in-law (2), grandchildren (2), niece (1), and friends/neighbour (4). These relatives comforted and supported the deceased in various ways (12), took responsibility (6), or cared for the patients during hospitalization (5).

### Affiliations and KIs’ characteristics

The 47 KIs were selected from different levels. Their average age was 47.6 years (standard deviation of 7.6 years). The KIs from the provincial level were heads of offices (2), program managers (3), analysts (2), or partner organisation staff (2). KIs from health district offices were medical officers (2), supervisors (5), or administrators (4). Most of the KIs from facilities were in charge of clinical care and preventive activities in the health area, which is the lowest tier of the health system in DRC. The KIs from the community level were opinion leaders (2) or volunteer community health workers (6).

### Conspiracy theories on Ebola

Although the EVD outbreak was recognised as dangerous, conspiracy theories emerged, including beliefs about population control or destruction, and even a supernatural origin. Some declared that the depopulation could make it easier for another population to seize North Kivu’s resources. Others claimed Ebola was used as a political strategy to hold back general elections and keep the authorities in power. These led to a loss of confidence in the government and a reluctance to use HCFs. Sometimes, attacks on HCFs were reported because HCWs were considered accomplices of the aggressors or motivated by personal enrichment. This enrichment led to the term "Ebola business" as one respondent said: "The population doubts the disease and suspects financial and political motives (…) There were two versions: Ebola business, Ebola politics to exterminate people, and finally that the enemy takes our land” (KI11).

### Mis-information about Ebola treatment centres (ETCs)

The high mortality in ETCs led to fear and rumours at an early stage of the outbreak. These rumours were about harmful practices such as the existence of one substance that HCWs injected or of a hammer that they banged on the heads of patients. For KIs, various services were affected. It included challenges in the supply of blood products because blood donors were afraid. Attributing her aunt's death to the lack of blood donors, one informant stated: "Nobody was willing to donate blood because everyone feared going to the hospital. They feared being taken to the ETC and injected with a substance that would cause their death" (D3r2).

Another theory was that human organs had been removed from ETCs. This rumour was amplified by limited contact between patients and their family members, and the prohibition of observing and burying their corpses. For others, the ETC was a place for people to die. "They thought the Ebola Treatment Centre was a place of death. Once you're there, you're a dead man. It's true. Just to get out alive from the Ebola Treatment Center was lucky" (KI05).

### Fear of infection control measures

Some KIs reported fear regarding the use of chlorinated water for hand washing and the Thermo-flash for temperature monitoring. There were rumours that the chlorinated water smell was due to the Ebola virus or poison. Therefore, washing hands with chlorinated water was a way of inoculating the Ebola virus or poison. The Thermo-flash was rumoured to be like a revolver, used for injecting a product or extracting the ability to think and thus make them tameable. A community leader said: "But what created resistance was the Thermo-flash device. (…). They thought it would suck their intelligence out of their heads" (KI13).

### Divergent views of PPE

There were divergent perspectives on the use of PPE. For HCWs, PPE was for protection. Some patients feared the HCWs who used PPE when treating patients. They expressed regret at no longer being touched by HCWs or being examined only with gloved hands, a sign of contempt. For others, HCWs dressed in PPE were no longer recognisable and were scary. Several KIs indicated that the process of donning PPE was time-consuming and contributed to misunderstandings, particularly in situations requiring urgent care (Table 2).

**Table 2:** Distribution of the KIs by level, socio-demographic, and professional characteristics

Characteristics	Number n = 47	(%)
*Gender*		
Male	39	83.0
Female	8	17.0
*Age group (year)*		
35 to 44	8	17.0
45 to 54	18	38.3
55 and +	3	6.4
Not specified	18	38.3
*Length of service (year)*		
3 to 10	6	12.8
11 to 20	25	53.2
21 and over	11	23.4
Not specified	5	10.6
*Education level*		
Primary	2	(4.2
Secondary	6	12.8
University	36	76.6
Not specified	3	6.4
*Qualification/Function*		
Nurses	21	44.7
Administrators	9	19.1
Physician	7	14.9
Other	4	8.5
None/ Not specified	6	12.8
*Site of interview*		
Goma city	6	12.8
Beni health districts	19	40.4
Butembo health districts	10	21.3
Katwa health districts	7	14.9
Other*	5	10.3
*Responsibility level*		
Community	8	17.0
1st line facility	13	27.7
2nd line facility	6	12.8
Health District offices	11	23.4
Provincial officer	9	19.1

*3 KIs from Mabalko, a health district close to Beni and the starting point of the 2018–2020 outbreak; and 2 KIs from Musienene Health District, which is close to Butembo health district

### Patients waiting time

Patients experienced stress and delays in being welcomed by HCWs. Some felt there was no change. They believed that there were problems even before the Ebola outbreak. Many patients complained of HCWs' reluctance and a lack of enthusiasm. For others, the screening process was so upsetting that they gave up without receiving treatment. For example, a deceased’s relative suggested that:

… In the past, that was not the case. (…,) when the healthcare providers saw you, they ran quickly to pick up the patient and start treating him. In short, the reception has changed, Maybe they were afraid of the Ebola Virus Disease (D1r1).

### Change of healthcare-seeking behavior

Fear was also triggered by the arrival of the ambulance, the wait for Ebola test results, and potential transfer to an ETC, during the quarantine period. To avoid suspicion of Ebola, some patients were taking treatment shortly before arrival at the hospital to mask symptoms, while others lied about their identities, contacts, or the disease evolution. Despite testing all suspected cases, there were reports of delayed treatment for patients awaiting test results, resulting in fatal consequences for many. One informant confided:

… The time to go to the Ebola Treatment Center [for investigation] and come back when the patient is seriously sick can cause his death. And maybe he wasn't going to die (D1r2).

### Availability, motivation, and attitudes of HCWs

The KIs reported that some HCWs abandoned routine care for outbreak response activities or due to fear of contamination. The remaining personnel were described as understaffed and overwhelmed. Some HCWs felt that the patient influx and FCP led to higher salaries, but others denounced delays and salary disparities. Other HCWs reported a drop in remuneration when changing from a total to partial FCP. The reduction in the utilization of unsubsidised services, coupled with the rise in unpaid patient invoices, led to diminished revenue for HCFs and decreased provider incentives. Some HCWs felt that the increase in earnings was not commensurate with the workload, leading to discomfort and complaints. Believing that the negative attitude of HCWs was associated with remuneration, one informant confided: " We had noticed that they were neglecting us. We don't know if they were paid or not" (D8r3).

### Perceptions and effects toward FCP

Implemented as soon as the EVD outbreak began, the FCP was initially opposed but quickly accepted. Some KIs supported the idea that this rapid change was due to community poverty. There were rumours of expired or low-quality free drugs. There were also suspicions that the FCP aimed to attract people to ETCs to exterminate them. As part of a performance-based funding policy, the collection of data at the individual level (home address, patient's identity card) has become essential. This was perceived as a threat in this area with recurrent massacres. This worsened the distrust of HCFs, and therefore, some patients gave false addresses. As a result, some medical acts were untraceable in the community (one of the performance-based financing requirements), and some invoices were partially honoured for suspicion of fraud, hence leading to the discouragement of HCWs.

FCP implementation varied across locations, with some experiencing changes or interruptions. All care was completely free of charge in some places throughout the outbreak. In others, care was free at the early stages and partially free after that. Sometimes the changes were abrupt and patients were informed only when they arrived at the facility. Some patients had to return home without treatment when they could not pay. Public and some private HCFs experienced changes, including increased patient influx in HCFs under FCP and closures of private HCFs without FCP for lack of clients. One respondent said: "… private healthcare facilities were better utilised because people were afraid of being considered infected by Ebola in public facilities. But, when healthcare became free, it was the opposite: everyone wanted to go where healthcare was free" (KI04).

An opinion leader confided, talking about FCP consequences on the influx and patient reception: " Free healthcare unbalanced certain private structures, while the influx of patients caused delays in reception and treatment at public healthcare facilities" (KI14).

### Patient preferences for HCFs

Based on informants’ opinions, during the outbreak, patients initially avoided public HCFs due to fear of EVD contamination and sought care from private or informal HCFs. Some patients preferred traditional healers and priests for illnesses which they believed were caused by poisoning or a supernatural cause. Patients had limited interest in public HCFs due to restrictions on injectable drug use and limited access to laboratory tests. These tests were only available in ETCs and a few hospitals. As a result, many patients opted for self-medication.

Private HCFs and traditional healers reported several Ebola cases due to insufficient hospital hygiene. The response managers extended triage units and EVD surveillance, resulting in the referral of suspected Ebola cases to ETCs from any facilities. Self-medication and the refusal to consult HCFs increased, especially if the disease was not considered critically serious. A deceased's relative said: "… people avoided the healthcare facilities due to fearing misdiagnosis of Ebola. While you could be treated and cured, when the illness is Ebola, there is a slight chance of surviving. Some died without seeking hospital treatment "(D4r2).

### Influences of EVD on healthcare quality and outcomes

Some KIs felt that the care provided during the EVD outbreak was superficial. A few HCWs and several deceased relatives reported scanty anamnesis and premature release of hospitalised patients to make room for new admissions. A close relative expressed himself in these terms: "There was superficial treatment. Even if you are hospitalised, you will be discharged without being healed. Because of the outbreak, many people died" (D8r2).

## Discussion

The findings of this study demonstrated that trust in the health system and the government was lost due to conspiracy theories and misinformation. This led to a reluctance to use public HCFs and attacks against these facilities. In Liberia, Morse et al. [35] identified distrust of government as one of the predictors of HCFs utilization during the EVD outbreak. Mutombo et al. [27] reported mistrust of the response team and the national authorities in Ituri and North Kivu provinces (Eastern RDC) during the 2018–2020 Ebola outbreak. It is crucial to prioritize the restoration of trust, the dissemination of accurate information early in the outbreak about Ebola, and the roles of stakeholders. Indeed, Morse et al. proved that awareness predicted higher trust and use of services [35].

We found that patients were afraid to use HCFs, resulting in delayed treatment. Patients feared contracting Ebola during the continuum of care, being declared as an Ebola case, being isolated in quarantine, or being sent to an ETC. Patients who avoided HCFs close to ETCs or who had reported EVD cases. A reduction in the number of deliveries in HCFs was reported in Liberia among people who believed they could contract Ebola there [36]. Triage units and ETCs were seen as entry points to “death corridors” because of the high mortality rates observed there in the earlier and middle periods of the outbreak. This prompted patients to prefer informal facilities or HCFs without a triage unit. During a previous EVD outbreak [17], researchers documented this psychosocial effect of Ebola. Family visits to patients in ETCs may help dispel misconceptions about ETCs, as advocated by Carter et al. [28].

Rapidly, informal and private HCFs became amplification sites of the outbreak, supporting the idea of closing some and triage’s expansion to almost all facilities, resulting in outbreak control. Therefore, an earlier extension of triage units to almost all facilities, regardless of size or affiliation, could maintain access to quality healthcare that informal HCFs may be challenged to provide health care. Although this approach will require additional resources, it would facilitate rapid outbreak control.

Patients expressed fear of the chlorinated water used for hand washing and the Thermo-flash device used for temperature monitoring. Some patients expressed concerns about receiving care from HCWs fully covered in PPE and not recognizable. Despite this, there is insufficient evidence that covering more body parts offers better protection. Other evidence shows that the more uncomfortable or difficult to put on or remove PPE is, the higher the risk of contamination for HCWs [37]. These fingdings highlight the need to consider patients' expectations on effective PPE for protection against highly infectious diseases [38, 39]. Simulation exercises could prepare populations for more resilience to future outbreaks.

Resources were predominantly earmarked for the outbreak response rather than for routine healthcare services, according to HCWs and managers. This focus on the outbreak caused disruptions to HCFs as revealed by Takahashi et al. [40]. Helleringer and Noymer suggested that the focus should not be on a single disease but on the health system and all the important components of the disease burden [19]. The massive importation of unfamiliar and non-credible HCWs into local communities also increased distrust and led to attacks against HCFs. Future outbreak managers should involve more local staff in community activities such as sensitisation, investigation, and vaccination against Ebola [28].

This study showed that free HCP was widely implemented and adopted as one of the key policies in the response to the outbreak as stated in the literature [21, 41]. This alternative to direct payment discounts resulted in a significant increase in visits for several services, supporting the finding that the FCP can mitigate the effects of the Ebola outbreak. This was the case in Equateur, western DRC [8], and North Kivu, eastern DRC [42]. The same effect was documented in various settings beyond Ebola outbreaks in Burkina Faso [43] and in Africa [44]. The introduction of the FCP led to the abandonment of private HCFs, despite the positive effect discussed above. FCP was implemented using several approaches, depending on the funding source, HCFs’ affiliation, and the epidemic's duration.

Service utilization decreased when the FCP changed from completely free to partially free. The findings suggest that the effects of FCP depend on its content, and funding availability is crucial for the intervention’s sustainability. In addition to the lack of funding, challenges such as interruptions or reductions of services eligible to FCP must be addressed, as well as the workload for HCWs and longer patient waiting time documented during the same Ebola epidemic in North Kivu [42]. In other settings, concerns about lower quality of health care were reported in the context of free health care published by Ridde and Queuille [25], Ridde et al. [45], and De Sardan and Ridde [46]. This study also reveals the interest in standardizing the content of FCP, including other services such as access to blood products in FCP and increasing the number of staff dedicated to routine healthcare services. According to Chu's recommendations [47], additional research is necessary to evaluate various FCP approaches, to identify the most at-risk services, and the most efficient and effective approach, and to evaluate their long-term impacts. It is also essential to implement reforms that lead to sustainable solutions such as universal health coverage [48–50].

Decongesting HCFs by offering other alternatives, such as the organisation of "advanced triage" or family medicine, can help manage the patient influx [51, 52]. Restoring the relationship between providers and patients needs to combat distrust, misinformation, and negative attitudes and behaviours, as concluded by Brown et al. [53, 54]. Sensitizing and fostering a permanent dialogue between patients and HCWs can address the differences in interests about PPE and expectations [55, 56]. HCWs used less invasive procedures, laboratory tests, and injectable drugs, probably due to fear of contamination. Indeed, there was no official instruction about restrictions. Patients preferred private HCFs with fewer restrictions. The findings suggest that drugs were utlised more rationally in public HCFs during the epidemic.

In the hospital, patients and their attendants were systematically screened using clinical and biological criteria, resulting in delayed treatment. Half of the family members interviewed in this study believed that the Ebola outbreak had an impact on their relative’s death. They stated that their relative was unable to access the appropriate HCFs or had to wait a long time for treatment while waiting for Ebola test results. Further efforts are necessary to bring Ebola rapid diagnostic tests closer to the first-line HCFs [26] to minimize this treatment delay, while acknowledging the efforts in laboratory deployment [57]. Introducing protocols for managing patients while waiting for Ebola tests may reduce non-specific Ebola mortality. The significant rates of mortality attributed to malaria, human immunodeficiency virus, and tuberculosis have been previously documented in West Africa [18]. As this study did not seek to explain reasons for high mortality non-specific to Ebola, further in-depth investigations are needed for this objective and to identify appropriate interventions. This study emphasizes the importance for managers of healthcare services to focus on routine healthcare as much as they do in response to the outbreak. It also highlights the need to combat misinformation, better prepare communities for potential crises, and build trust in the government, the healthcare system, and the interventions implemented.

## Limitations

This study has some limitations. All cases in this study died after seeking HCFs. Individuals who died at home or patients who did not seek care were not included. This choice might not fully reflect all patients’ preferences for HCFs and the barriers to HCFs utilization. However, our study sheds light on the management of non-Ebola patients, supposed to be relegated to the back during the outbreak [19]. Although there may be a potential bias in our selection, it does not appear to have significantly affected the study, as a higher number of KIs at the community level were integrated. The case study design and sample size did not allow for the generalization of the findings obtained. However, these findings are consistent with the literature. Furthermore, this study did not address certain aspects that emerged during the analysis such as the payment and working conditions of routine HCWs, treatment protocols for common illnesses, and patients waiting for the Ebola test results. Future studies are needed to explore these aspects.

## Conclusions

This study has identified barriers that hindered the use of HCFs during the 2018–2020 Ebola outbreak in North Kivu. These include fear, misinformation, lack of trust in government and HCFs, negative attitudes and perceptions, HCWs’ overload and demotivation, and long patient waiting time. Conversely, community involvement, employing familiar, neutral, and credible staff, and implementing the FCP improved the use of HCFs. These findings may not apply beyond its setting, but they can inform policies before and during future outbreaks, in any setting, to enhance the resilience of routine HCFs. Keeping up community engagement activities in the post-epidemic period and maintaining dialogue between HCWs and patients could help rebuild confidence in HCFs in North Kivu. As a perspective to the current study, quantitative studies including analysis of the context and disruption elements are essential to identify the major determinants of care-seeking during such a crisis.

## Supplementary Information

**Additional file 1**. Clinical Data Extraction Grid.

**Additional file 2**. Interview Guide for Relatives of the Deceased.

**Additional file 3**. Semi-Structured Questionnaire for Key Informant Interviews.

**Additional file 4**. Codes tree for this study.

**Additional file 5**. The COREQ checklist for this study identifies standardised information required for qualitative studies.

**Additional file 6**. STROBE Statement checklist of items that should be included in reports of observational studies.

**Additional file 7**. Relatives Interviews Output files from Atlas-ti.

**Additional file 8**. Key Informants Interviews Output files from Atlas-ti.

**Additional file 9**. Ethical committee letter French.

**Additional file 10**. Ethical committee letter English.

## Data Availability

The data supporting the findings of this paper are available upon reasonable request addressed to the corresponding author.

## Abbreviations

DRC: Democratic Republic of Congo
ETC: Ebola treatment centre
EVD: Ebola virus disease
FCP: Free care policy
HCFs: Healthcare Facilities
HCWs: Healthcare workers
KI: Key informants
PPE: Personal protective equipment
SSIs: Semi-structured interviews
