# Supplementary material for: Barriers and facilitators to healthcare facility utilization by non-Ebola patients during the 2018–2020 Ebola outbreak in the Democratic Republic of Congo
Source: Glob Health Res Policy. 2024 Nov 19;9:47. doi: 10.1186/s41256-024-00387-6 (PMC11575170; doi:10.1186/s41256-024-00387-6)
Supplement: Supplementary file 1 — Additional file 1. Clinical Data Extraction Grid. [file 41256_2024_387_MOESM1_ESM.docx]

Additional file 1. Clinical Data Extraction Grid

Annexe 1: Grille d’extraction des données cliniques

NUMERO D’IDENTIFICATION : /__/__/

| **Numéro** | **Question** | **Réponse** |
| --- | --- | --- |
|  | **Identification** |  |
| D11 | Zone de Santé |  |
| D12 | Nom de l’établissement de soins de santé |  |
| D13 | Type d’établissement de la santé | 1. HGR 2. CSR 3. Autre à préciser : ……. ….. |
| D14 | Appartenance de l’établissement | 1. Publique 2. Privée confessionnelle 3. Privée à but lucratif 4. Autre à préciser : ……. ….. |
| D15 | Service | 1. Pédiatrie 2. Gynécologie obstétrique 3. Médecine interne 4. Chirurgie 5. Autre à préciser : ……. ….. |
| D16 | Adresse |  |
|  | **Caractéristiques socio-démographique** |  |
| D21 | Nom et pré nom |  |
| D22 | Sexe | 1. Masculin 2. Féminin |
| D23 | Age (mois/années) | /__/__/ Jour  /__/__/ Mois  /__/__/ Année |
| D24 | Niveau d’éducation | 1. Inconnu 2. Université/Supérieur 3. Secondaire 4. Primaire 5. Aucun |
| D25 | Qualification |  |
| D26 | Occupation pendant sa vie |  |
| D27 | Si Femme de 14 ans ou plus,  S’agissait-il d’une femme enceinte ? | 1. Oui 2. Non |
|  | **Caractéristiques Cliniques** |  |
| D31 | Date d’admission/Hospitalisation | __ __ /__ __ / 20 __ __ |
| D32 | Date de décès | __ __ /__ __ / 20 __ __ |
| D33 | Durée d’hospitalisation (En jour) |  |
| D34 | Brève description du tableau clinique/ de la maladie (principales plaintes) |  |
| D35 | Antécédents médicaux (ou autres pathologie associées) |  |
| D36 | Itinéraire de soins de patients |  |
| D36a | Passage par le CTE | Oui/Non |
| D36b | Passage dans l’isolement | Oui/Non |
| D37 | Lieu/Circonstances de décès |  |
| D38 | Diagnostic/Cause de décès selon les soignants (Cause principale du décès telles attribuées par le clinicien (Diagnostic) |  |
| D39 | Commentaire |  |
