# Supplementary material for: Barriers and facilitators to healthcare facility utilization by non-Ebola patients during the 2018–2020 Ebola outbreak in the Democratic Republic of Congo
Source: Glob Health Res Policy. 2024 Nov 19;9:47. doi: 10.1186/s41256-024-00387-6 (PMC11575170; doi:10.1186/s41256-024-00387-6)
Supplement: Supplementary file 2 — Additional file 2. Interview Guide for Relatives of the Deceased. [file 41256_2024_387_MOESM2_ESM.docx]

Additional file 2. Interview Guide for Relatives of the Deceased

**Annexe 2. Guide d’entretien avec les proches parents des personnes décédées**

**Effets de l’épidémie d’Ebola sur l’utilisation des établissements des soins de santé**

1. **FORMULAIRE D’INFORMATION ET DE CONSENTEMENT**
   1. **Introduction**

Mon nom est …… Je suis accompagné de ……. Nous sommes membres de l’équipe de recherche de l’Ecole de Santé Publique de Kinshasa. Vous avez été identifié comme personne ressource pour nous renseigner sur l’étude intitulée : *« Effets de l’épidémie d’Ebola sur l’utilisation des établissements de soins de santé »*. Ce projet vise à évaluer l’effet de l’épidémie à virus Ebola sur les services de santé. Nous sollicitons votre participation aujourd’hui pour recueillir un témoignage sur le cas de … [nom du défunt] votre … [lien de parenté] qui a été pris en charge dans l’établissement de soins de santé … [nom de l’établissement]. L’étude cherche à comprendre dans quelles circonstances le décès est survenu afin, le cas échéant, de contribuer à améliorer les interventions sanitaires au cours de cette crise et de futures crises.

D’abord nous vous présentons nos sincères condoléances pour cette perte. Nous vous rassurons ensuite que cette étude n’est pas un jugement contre la structure, les prestataires ou la famille de …. [nom du défunt]. Son intérêt est plutôt d’identifier les facteurs, évitables ou non, qui ont contribué au décès depuis l’apparition de la maladie en passant par la prise en charge avant l’hospitalisation et après l’admission dans la structure. Vous allez pour cela vous appuyez sur ses antécédents et l’histoire de la maladie pour décrire sa prise en charge au niveau communautaire et dans les établissements de santé par où il est passé. Bien avant, je vais vous lire tour à tour certaines points afin de vous aider à avoir plus d’informations sur l’étude.

- 1. **Informations sur l’étude**

***Objectif*** : Déterminer l’effets de l’épidémie d’Ebola sur l’utilisation des établissements de soins de santé , sur les pratiques de recours aux soins et sur la qualité des services de santé à l’Est de la RDC.

***Participants à l’étude****:* Ce sont les proches parents qui ont le plus d’informations sur le début de la maladie, les soins reçus, sur le séjour hospitalier et sur les circonstances de décès retenus.

***Raisons et attentes de votre participation à l’étude****:* nous vous abordons car vous êtes une personne ressource habilitée à nous renseigner le mieux possible sur le sujet de recherche. L’entretien durera environ 30 minutes, se réalisera en français ou dans la langue qui vous convient le mieux et se déroulera dans un lieu de votre convenance. En aucun moment nous voulons interférer avec votre travail : n’hésitez donc pas de nous fixer le rendez-vous au moment qui vous semblera plus approprié. Vous serez autorisé à consulter, avant et pendant l’interview, tout document disponible sur *[nom du défunt]*. N’hésitez pas non plus de faire appel à quelqu’un qui pourrait vous compléter. Ceci est important pour que vous n’omettiez aucun détail qui pourrait être important. Nous sollicitons aussi votre accord pour utiliser un enregistreur audio lors de cet entretien. L’entretien enregistré sera retranscrit intégralement sur papier pour fins d’analyses.

***Confidentialité et anonymat des données de la recherche****:* tous les renseignements recueillis pour l’étude sont anonymes et ne seront utilisés que dans le cadre de cette recherche. Les enregistrements audios seront conservés dans un endroit sécurisé auquel seuls les chercheurs auront accès. A la fin du processus, ces enregistrements seront effacés alors que les transcrits seront conservés pendant 5 ans après lesquels ils seront également détruits. Si nous prenons vos informations personnelles, c’est pour nous fournir des données devant nous permettre de mieux comprendre le contexte. Ces informations personnelles ne seront en aucun cas utilisées pour vous identifier. Pour renforcer l’anonymat, ces informations personnelles seront gardées sur des documents bien séparés. Seuls les codes seront utilisés pour les relier.

***Risques et inconvénients liés à votre participation à la recherche****:* votre participation à cette étude présente quelques risques. En dehors du temps que vous allez consacrer à l’entretien, l’entretien va raviver les douleurs de la perte d’un être cher dont les cicatrices sont encore fraiches. Nous vous rassurons toute notre compensions et notre soutien moral. Nous pourrions également en parlez à votre médecin, à votre pasteur ou à votre entourage pour qu’ils continuent à vous soutenir. Par ailleurs, votre opinion sur le système de santé ou le contexte dans lequel *[nom du défunt]* a été pris en charge peut être considérée comme une critique contre laquelle vous pourriez craindre des représailles. Cependant, les mesures d’anonymat et de confidentialité expliquées ci-dessus devraient être suffisantes pour vous rassurer. Cela n’affectera pas la qualité des soins lors de votre prochaine visite dans l’établissement. Bien plus, les résultats seront présentés de manière anonyme.

***Bénéfices liés à la participation à la recherche****:* Le plus grand bénéfice de votre participation à l’étude n’est pas personnel. Votre participation aidera le Ministère de la Santé d’en savoir davantage sur la vulnérabilité de son système de santé face aux épidémies comme Ebola afin d’orienter ses décisions. Cependant, comme il est dit de pleurer avec ceux qui pleurent, l’équipe de recherche est engagée à plaider pour que vous ayez plus de soutien moral, psychosocial, financière et matériel de la part de votre entourage. L’équipe de recherche pourra aussi vous apporter ce même soutien dans la mesure de ses moyens.

***Participation volontaire et possibilité de retrait****:* vous êtes libre de participer ou de ne pas participer à la présente étude. Vous pouvez interrompre votre participation à ton moment sans préjudice aucun sur l’accueil et les soins lors de prochaines visites à l’établissement de soins. Cependant, nous vous rassurons que votre participation est très importante pour nous et pour le système de santé.

***Communication des résultats aux participants****:* les résultats obtenus à l’issue de cette recherche permettront de rédiger une thèse qui sera soutenue publiquement selon les modalités d’usage à l’université de Kinshasa. Ils seront également partagés lors d’une restitution des résultats aux parties prenantes, présentés sous forme de poster lors des conférences ou publiés dans les revues scientifiques internationales.

***Approbation du protocole de recherche****:* le protocole et les procédures opérationnelles de cette recherche ont été soumis pour approbation au Comité d’Ethique de l’Ecole de Santé Publique de l’Université de Kinshasa qui a donné son approbation.

***Personne à contacter en cas de problème****:* n'hésitez pas à poser des questions à tout moment pendant l'étude. Si vous avez des questions à propos de l'étude ou sur vos droits en tant que participants, veuillez communiquer avec l’investigateur principal, le Docteur Kyomba Kalombe Gabriel (+243 81 220 76 33, [gabriel.kyomba@unikin.ac.cd](mailto:gabriel.kyomba@unikin.ac.cd)) ou aux superviseurs de l’étude notamment le professeur Kiyombo Mbela Guillaume (+243 81 518 68 72) et Serge Mayaka (+243 82 414 35 97). Avez-vous des questions au sujet de l’étude ?

Avons-nous votre accord de participer à l’étude ? 1. Oui 2. Non

Si oui, continuer l’interview. Si non, clôturer et identifier un autre participant

1. **GUIDE D’ENTRETIEN**

**qR01. Informations sur le répondant et sur l’étude**

Nom et pré nom du répondant : Nom du défunt : Téléphone :

| Age | | : |  |  | Sexe | : |  |
| --- | --- | --- | --- | --- | --- | --- | --- |
| Qualification | | : |  |  | Occupation(s) | : |  |
| Niveau d’éducation | | : |  |  | Lien de parenté avec le défunt | : |  |
| Fonction | | : |  |  |  | : |  |
| Contacts | – | : |  |  | – email | : |  |
| Date de l’interview | | : |  |  | Nom de l’enquêteur | : |  |
| Heure de début | | : |  |  | Heure de la fin | : |  |

- 1. **Thèmes de discussion**
     1. **Questions introductives**

**Nous allons commencer par quelques questions qui vous concernent et d’autres qui concernent le défunt.**

| ***Questions principales*** | **Questions fouilles** |
| --- | --- |
| **qR01.** *Parlez-nous brièvement de vous et du défunt et du liens de parenté qui vous unit*  **qR02.** *Quel rôle avez-vous joué dans la prise en charge de [nom du défunt] et comment vous êtes-vous retrouvé à jouer cette responsabilité ?* | - *Liens de parenté entre vous et lui* - *Ce qu’a été le défunt* - *Rôle dans la prise en charge (responsabilité du répondant face au défunt* |

- - 1. **Thème 1 : Maladie et prise en charge**

**Nous voulons à présent parler de l’histoire de la maladie, de la prise en charge avant l’admission à l’hôpital, de l’accueil et de l’état du défunt à l’admission dans le dernier hôpital où il a été pris en charge.**

| ***Questions principales*** | **Questions fouilles** |
| --- | --- |
| **qR11.** *Parlez-nous de l’histoire de la maladie et de tout ce que vous savez sur ce qui s’est passé depuis l’apparition de la maladie jusqu’à l’arrivée à hôpital où [Nom du défunt……] est décédé*  **qR12.** *Dans quel état [Nom du défunt……] était-il arrivé à l’hôpital,*  **qR13.** *Que direz-vous de l’accueil,*  **qR14.** *Que direz-vous de la prise en charge*  **qR15.** *Que direz-vous du soutien (social, psychologique ou spirituel) dont il a bénéficié dans l’hôpital où il est décédé ?* | - Plaintes, - Antécédents (de quoi souffrait-il d’autres ou maladies associées) - Différents traitements reçus (types, lieu, qui a prescrit le traitement), - Evolution de la maladie, - Référence si applicable - Etat général à l’admission (urgence ou pas, etc…), - Attitude des prestataires, proactivité - Tentatives pour le sauver, disponibilité en médicaments, accès aux laboratoire (imagérie), situation financière, - Soutien de la famille/église, visite |

- - 1. **Thème 2 : Circonstances de décès**

**Nous voulons à présent parler des circonstances dans lesquelles le décès est survenu.**

| ***Questions principales*** | **Questions fouilles** |
| --- | --- |
| **qR21.** *Que direz-vous des circonstances ou des causes () du décès de [Nom du défunt……] ?* | - Facteurs favorisants - Causes lointaines et immédiates - Causes selon les soignants et selon la famille - Causes liées au service (personnels, médicaments, équipement, …et - Causes liées à la personne (adhérence au traitement, respect des conseils, …) |

- - 1. **Thème 3 : Opinions du répondant sur l’influence de l’épidémie sur l’évolution de la situation.**

**Nous voulons à présent obtenir vos propres opinions, c’est-à-dire ce que vous pensez sur ce qui s’est passé et sur ce qui serait passé s’il n’y avait pas eu l’Epidémie.**

| ***Questions principales*** | **Questions fouilles** |
| --- | --- |
| **qR31.** *Pensez-vous personnellement que les choses auraient pu se passer différemment s’il n’y avait pas eu Ebola dans cette zone où se trouvait … [Nom du défunt……] ?*  **qR32.** *Selon-vous, si c’était à refaire, qu’est-ce qui a marché (et qu’on peut garder) ou n’a pas marché (et que l’on doit changer) dans la prise en charge de [Nom du défunt……].* | - Organisation de la structure, - Choix de la structure et du moment de consulter la structure, - J’aimerai que vous répondiez en considérant à la fois du côté du malade, de sa famille/communauté et du coté des soignants et de la structure. |

- - 1. **Thème 4 : Conclusion et autres informations**

| ***Questions principales*** | **Questions fouilles** |
| --- | --- |
| **qR41.** *Avez-vous un commentaire ou une recommandation à ajouter ?* | - Point de vue, - Souhait - Recommandation |

Merci beaucoup d’avoir consacré de votre précieux temps pour répondre à nos questions. Il est possible que nous puissions avoir besoin de vous contacter encore pour clarifier ou compléter certaines informations dont nous n’avons pas parler mais qui pourraient être utiles pour la suite de l’étude. Si tel est le cas, serez-vous disposé à nous répondre ?

Encore une fois merci beaucoup !
