# Supplementary material for: Barriers and facilitators to healthcare facility utilization by non-Ebola patients during the 2018–2020 Ebola outbreak in the Democratic Republic of Congo
Source: Glob Health Res Policy. 2024 Nov 19;9:47. doi: 10.1186/s41256-024-00387-6 (PMC11575170; doi:10.1186/s41256-024-00387-6)
Supplement: Supplementary file 3 — Additional file 3. Semi-Structured Questionnaire for Key Informant Interviews. [file 41256_2024_387_MOESM3_ESM.docx]

Additional file 3. Semi-Structured questionnaire for Key Informant Interviews

**Annexe3. Questionnaire semi-structure pour entretiens avec les informateurs clés**

**Effets de l’épidémie d’Ebola sur l’utilisation des établissements des soins de santé**

1. **FORMULAIRE D’INFORMATION ET DE CONSENTEMENT**
   1. **Introduction**

Mon nom est…………………………… Je suis membre de l’équipe de recherche de l’Ecole de Santé Publique de Kinshasa. Vous avez été identifié comme personne ressource pour nous renseigner sur l’étude intitulée : *« Effets de l’épidémie d’Ebola sur l’utilisation des établissements de soins de santé ».* Nous sollicitons votre participation aujourd’hui pour recueillir vos perceptions concernant l’effet de l’épidémie à virus Ebola sur les services de santé. A présent je vais vous donner quelques informations sur l’étude.

- 1. **Informations sur l’étude**

***Objectif*** : Décrire l’effet de la riposte contre la MVE sur les six composantes du système de santé au niveau local à l’Est de la RDC

***Participants à l’étude****:* l’étude concerne les gestionnaires des services de santé au niveau provinciale, de la zone de santé et des formations sanitaires. Nous allons aussi discuter avec d’autres acteurs venant des ONG partenaires et des Centrales Régionales de Distribution des médicaments.

***Raisons et attentes de votre participation à l’étude****:* nous vous abordons car vous êtes une personne ressource habilitée à nous renseigner le mieux possible sur le sujet de recherche. L’entretien durera environ 60 minutes, se réalisera en français et se déroulera dans un lieu de votre convenance. En aucun moment nous voulons interférer sur votre travail : n’hésitez donc pas de nous fixer le rendez-vous au moment qui vous semblera plus approprié. Nous sollicitons aussi votre accord pour utiliser un enregistreur audio lors de cet entretien. L’entretien enregistré sera retranscrit intégralement sur papier pour fins d’analyses.

***Confidentialité et anonymat des données de la recherche****:* tous les renseignements recueillis pour l’étude sont anonymes et ne seront utilisés que dans le cadre de cette recherche. Les enregistrements audios seront conservés dans un endroit sécurisé auquel seuls les chercheurs auront accès. A la fin du processus, ces enregistrements seront effacés alors que les transcrits seront conservés pendant 5 ans après lesquels ils seront également détruits. Si nous prenons vos informations personnelles, c’est pour nous fournir des données devant nous permettre de mieux comprendre le contexte. Ces informations personnelles ne seront en aucun cas utilisées pour vous identifier. Pour renforcer l’anonymat, ces informations personnelles seront gardées sur des documents bien séparés. Seuls les codes seront utilisés pour les relier.

***Risques et inconvénients liés à votre participation à la recherche****:* votre participation n’aura pas d’inconvénients majeur en dehors du temps que vous devrez consacrer à l’entretien. Certes que votre opinion sur le système de santé ou le contexte dans lequel vous travaillez peut être considérée comme une critique contre laquelle vous pourriez craindre des représailles. Cependant, les mesures d’anonymat et de confidentialité expliquées ci-dessus devraient être suffisantes pour vous rassurer. Bien plus, les résultats seront présentés de manière anonyme.

***Participation volontaire et possibilité de retrait****:* vous êtes libre de participer ou de ne pas participer à la présente étude. Vous pouvez interrompre votre participation à ton moment sans préjudice aucun sur vous et vos activités. Cependant, nous vous rassurons que votre participation est très importante pour nous et pour le système de santé.

***Bénéfices liés à la participation à la recherche****:* vous n’aurez pas un bénéfice personnel en dehors du fait que votre participation aidera le Ministère de la Santé d’en savoir davantage sur la vulnérabilité de son système de santé face aux épidémies comme Ebola. Aucune compensation n’est prévue pour cet entretien.

***Communication des résultats aux participants****:* les résultats obtenus à l’issue de cette recherche permettront de rédiger une thèse qui sera soutenue publiquement selon les modalités d’usage à l’université de Kinshasa. Ils seront également partagés lors d’une restitution des résultats aux participants et autres parties prenantes (autorités sanitaires provinciales et nationales, partenaires techniques et financiers du secteur de la santé), présentés sous forme de poster lors des conférences et publiés dans les revues scientifiques internationales.

***Approbation du protocole de recherche****:* le protocole et les procédures opérationnelles de cette recherche ont été soumis pour approbation au Comité d’Ethique de l’Ecole de Santé Publique de l’Université de Kinshasa qui a donné son approbation.

***Personne à contacter en cas de problème****:* n'hésitez pas à poser des questions à tout moment pendant l'étude. Si vous avez des questions à propos de l'étude ou sur vos droits en tant que participants, veuillez communiquer avec l’investigateur principal, le Docteur Kyomba Kalombe Gabriel (+243 81 220 76 33, [gabriel.kyomba@unikin.ac.cd](mailto:gabriel.kyomba@unikin.ac.cd)) ou aux superviseurs de l’étude notamment le professeur Kiyombo Mbela Guillaume (+243 81 518 68 72) et Serge Mayaka (+243 82 414 35 97). Avez-vous des questions au sujet de l’étude ?

Avons-nous votre accord de participer à l’étude ? 1. Oui 2. Non

Si oui, continuer l’interview. Si non, clôturer et identifier un autre participant

1. **QUESTIONNAIRE D’ENTRETIEN PROPREMENT DIT**

**qI01. Informations sur le répondant et sur l’étude**

| Province | : |  |  | Institution | : |  |
| --- | --- | --- | --- | --- | --- | --- |
| Nom et pré nom | : |  |  | Sexe | : |  |
| Qualification | : |  |  | Niveau d’éducation | : |  |
| Fonction | : |  |  | Ancienneté | : |  |
| Date de l’interview | : |  |  | Nom de l’enquêteur | : |  |
| Heure de début | : |  |  | Heure de la fin | : |  |

- 1. **Thèmes de discussion**

**qI02. Questions introductives**

- ***Questions pour cadres de DPS, BCZS et FOSA***

1. En quoi consiste votre travail en rapport avec les services de routine ?
2. En quoi a consisté votre travail en rapport avec la lutte contre la MVE ?

- ***Questions pour représentants des ONG partenaires***

1. En quoi consiste habituellement le partenariat entre votre organisation et le services ou institution de santé ? Comment ce partenariat a-t-il été mis en œuvre pendant l'épidémie ?
2. Comme membre de l’organisation, pouvez-vous nous parler de votre rôle dans le partenariat entre votre organisation et le services ou institution de santé ?
3. Quel rôle vous avez joué pendant l’épidémie de maladie à virus Ebola aussi bien pour les activités de routines que celles de riposte ? A partir de quel moment votre organisation et vous-même avez été impliqué dans la riposte ?
   - 1. **Thème 1 : Gouvernance de services de santé de routine**

**Nous voulons d’abord parler de la manière que la population considéré l’épidémie et accueilli les mesures mises en place. Nous parlerons ensuite de la gouvernance de services de santé de routine en période d’Ebola.** Parmi les attributs de la gouvernance en santé il y a la *capacité administrative*qui traduit l’autorité ou l’espace nécessaire que dispose un gestionnaire pour prendre des décisions requises afin de guider et mener à terme un programme de santé conformément à la mission conférée à son poste. La gouvernance est l’existence de *mécanismes* de *coordination des parties prenantes (*y compris les partenaires et les structures privées). C’est aussi l’existence de mécanisme d’*implication des communautés* dans la planification et le suivi de la prestation de services. Enfin, la gouvernance implique la lutte contre la corruption, la redevabilité, la *production des rapports périodiques* et le fonctionnement des organes de gestion qui permettent de maintenir la confiance des communautés dans les services de santé.

| ***Questions principales*** | **Questions fouilles** |
| --- | --- |
| ***qI11****. Parlez-nous du début et de qu’a ou représente Ebola pour la communauté ?*  ***qI12.*** *En comparant la situation pendant l’épidémie de MVE et celle vécue avant la crise, dans quelle mesure la gouvernance locale des services de santé a-t-elle été impactée ?*  ***qI13.*** *Perlez-nous de l’implication des communautés et des acteurs locaux* | - Représente quoi, rumeurs, conséquences sur les activités de la vie courante, Qu’est-ce qui a changé ? - Autorité à prendre les décisions y compris l’affectation des ressources ; fixer les objectifs - Alignement et coordination des parties prenantes - Partenariat public-privé - Autodétermination des communautés, organes de gestion - Planification et rapport périodique des activités de routine - Influence de l’affluence des acteurs internationaux et nationaux |

- - 1. **Thème 2 : Ressources humaines**

**Parlons à présent du personnel des services de santé.** La présence d’un personnel qualifié et en nombre suffisant est l’une des conditions d’un fonctionnement harmonieux des services de santé. Et pourtant, la riposte contre Ebola a aussi mobilisé beaucoup d’agents. La conversion des certains prestataires vers d’autres fonctions a aussi été observée.

| ***Questions principales*** | **Questions fouilles** |
| --- | --- |
| ***qI21.*** *Diriez-vous qu’il y a eu des gaps en termes de qualification ou nombre du personnel pour l’offre des services de routine ? Si oui, comment avez-vous géré ce gap ?*  ***qI22.*** *Que diriez-vous des conditions de travail des agents de santé avant et pendant l’épidémie de MVE ?*  ***qI23.*** *Avez-vous constaté ou entendu parlé des changements dans les attitudes et pratiques des prestataires pendant l’épidémie de MVE ?*  ***qI24.*** *Ebola reste une maladie dangereuse et très contagieuse. Comment les prestataires ont-ils géré le devoir de soigner les patients et le besoin de se protéger contre Ebola pendant l’épidémie ?*  ***qI25.*** *A quel autre défi avez-vous été confronté en matière de gestion du personnel pendant l’épidémie de MVE ? Comment les avez-vous surmontés ?* | - Motivation du personnel - Exemple l’accueil, la manière de s’occuper des malades ou de soigner certaines maladies - Public vs Privé, CS vs HGR |

- - 1. **Thème 3 : Prestations des services de santé**

**Parlons à présent des prestations des services de santé.** Offrir aux communautés des soins de santé dont ils ont besoins reste un objectif clé du système de santé. L’utilisation des services de santé permet ainsi d’améliorer l’état de santé de la population.

| ***Questions principales*** | **Questions fouilles** |
| --- | --- |
| ***qI31.*** *Il y a-t-il eu des structures ou services qui n’ont pas correctement ou pas du tout fonctionné pendant l’épidémie ? Si oui, lesquels et pourquoi ?*  ***qI32.*** *Que pensez-vous de l’offre, de l’utilisation et de la qualité des services de santé avant et pendant l’épidémie de MVE ?*  ***qI33.*** *A quel défi avez-vous été confronté pour continuer à offrir les services de santé de routine de qualité en période d’Ebola et comment avez-vous surmontés ces défis ?*  ***qI34.*** *A votre connaissance, y a-t-il eu des problèmes de santé spécifiques pour lesquels les communautés ne fréquentaient pas les formations sanitaires pendant l’épidémie de la MVE ou les fréquentaient plus de la même manière qu’avant la crise ? Que faisaient-ils alors pour être soulagés ?* | - Motivation du personnel - Exemple l’accueil, la manière de s’occuper des malades ou de soigner certaines maladies - Public vs Privé, CS vs HGR |

- - 1. **Thème 4 : Médicament**

**Parlons à présent des médicaments.** L’un des piliers du système de santé est l’accès aux médicaments de qualité, au produits sanguins, commodités et technologies ou équipement de prise en charge ou diagnostic. Garantir la disponibilité des médicaments et le fonctionnement des équipements reste l’un des soucis permanents des gestionnaires. Cela nécessite que le cycle d’approvisionnement en médicaments et produits non spécifiques à Ebola soit performant. Ce cycle comprend les commandes, la gestion des stocks, la prévention des ruptures et péremptions, les capacités de transport et de stockage, l’utilisation et la disponibilité)

| ***Questions principales*** | **Questions fouilles** |
| --- | --- |
| ***qI41.*** *Avez-vous noté des différences ou lacunes dans le cycle d’approvisionnement en médicaments et produits de santé avant et pendant l’épidémie d’Ebola ? Lesquelles ?*  ***qI42.*** *Est-ce que cela à impacter l’offre et l’utilisation de certains services ? Comment ?*  ***qI43.*** *Si vous devez donner votre point de vue dans la planification d’une future riposte contre Ebola dans une région donnée, quel élément positif recommanderiez-vous et quel élément négatif déconseilleriez-vous en ce qui concerne l’approvisionnement en médicaments, produits sanguins et équipement ?* | - Commandes, la gestion des stocks, la prévention des ruptures et péremptions, les capacités de transport et de stockage, l’utilisation et la disponibilité - Produits sanguins - Equipements |

- - 1. **Thème 5 : Financement**

**Parlons à présent du financement des services de santé de routine pendant la crise d’Ebola.** La composante financement concerne les mécanismes de mobilisation, de mise en commun, de gestion et d’utilisation des fonds. Concernant la mobilisation, trois principales sources sont connues : les sources gouvernementales, les ressources extérieures venant des bailleurs et les sources privées constitués des ménages. S’agissant des ménages, plusieurs modes peuvent exister notamment la subvention totale (gratuité) ou partielle (tarif réduit) ou le tarif forfaitaire. La subvention peut elle-même être couverte par les partenaires, les assurances ou les mutuelles. L’utilisation concerne la priorisation des dépenses, le paiement des primes l’achat des performances, etc. En période d’épidémie, la priorité est accordée à la riposte, le flux financier augmente et le mode de financement est susceptible d’être modifié

| ***Questions principales*** | **Questions fouilles** |
| --- | --- |
| ***qI51.*** *Comment pouvez-vous mieux décrire le mode de financement dans votre entité avant et pendant Ebola ?*  ***qI52.*** *En ce qui vous concerne, quel est votre point de vue sur les conséquences de ce financement sur la performance des services de santé de routine ?*  ***qI53.*** *Si l’on devait financer à nouveau la riposte et les services de routine, quel élément positif recommanderiez-vous et quel élément négatif déconseilleriez-vous ?* | - Besoin vs ressources disponibles - Gratuité et motivation du personnel et prestation - Gratuité et utilisation des services - PBF et riposte (gratuité) - Mutuelle/assurance santé et Riposte - Riposte et coût (directes et indirects) des soins |

- - 1. **Thème 6 : Conclusion et autres informations**

| ***Questions principales*** | **Questions fouilles** |
| --- | --- |
| ***qI61.****Si une crise semblable resurgissait, quels types de changements aimeriez-vous qu’on apporte à la façon dont les services de santé de routine ont été gérés pendant l’actuelle épidémie de maladie à virus Ebola ?*  ***Iq.62.*** *Y a-t-il d'autres problèmes que vous aviez observés concernant la manière dont l’épidémie de la MVE a affecté le système de santé et que vous souhaiteriez nous partager ? Quelle recommandation faites-vous quant à ce ?* |  |

Merci beaucoup d’avoir consacré de votre précieux temps pour répondre à nos questions. Il est possible que nous puissions avoir besoin de vous contacter encore pour clarifier ou compléter certaines informations dont nous n’avons pas parlé mais qui pourraient être utiles pour la suite de l’étude. Si tel est le cas, serez-vous disposé à nous répondre ? Encore une fois merci beaucoup !
