# Supplementary material for: Barriers and facilitators to healthcare facility utilization by non-Ebola patients during the 2018–2020 Ebola outbreak in the Democratic Republic of Congo
Source: Glob Health Res Policy. 2024 Nov 19;9:47. doi: 10.1186/s41256-024-00387-6 (PMC11575170; doi:10.1186/s41256-024-00387-6)
Supplement: Supplementary file 4 — Additional file 4. Codes tree for this study. [file 41256_2024_387_MOESM4_ESM.docx]

O !!

Theme5: Therapeutic choices qD36,

qP11, qP21, qP31, qP32,

HealthCare practices & care parthway: C511-C514

Healthcare outcome: C521-C523

Perceived influence of EVD on healthcare outcole: C523

Demographic and socioeconomic characteristics: C11-C16

Knowledge attitude and practices : C57-C58

Healthcare delivery and quality: C311-C319

Care Providers availability, attitudes and practices : C321-C324

Healthcare funding: C331-C337

Access to drug and laboratory tests C341-C343

Healthcare management:C361-C362

Community engagement:  C318

EVD Considerations: C411-C413

Security situation: C421-C425

Opportunities offered by the outbreak: C431-C433

Changes resulting from the outbreak: C341-C343

Lessons and perspectives for futur crises: C441-C445

**Theme 1: Deceased caracteristics** qD11-qD27

**Relatives caracteristics qP01-qP02**

**Key informants caracteristics qI01-qI02**

**Theme2: Disease informations: qD31-qD39, qP11, qP12, qP21,**

**Theme 3: Health system component: qP14, qP15, qP31, qP32, qI21-qI25, qI12, qI13, qI31-qI34, qI41-qI43, qI51-qI53**

**Theme 4: The study context and lessons: qI11, qI61, qI62**

**Legend:**

Questions: qD (deceased), qR11 (Relative), qI11 (Key Informant),

Codes C11, C12, …C33 :

Illness history: C21, C22

Patient’s clinical condition on admission: C23
