# Supplementary material for: Barriers and facilitators to healthcare facility utilization by non-Ebola patients during the 2018–2020 Ebola outbreak in the Democratic Republic of Congo
Source: Glob Health Res Policy. 2024 Nov 19;9:47. doi: 10.1186/s41256-024-00387-6 (PMC11575170; doi:10.1186/s41256-024-00387-6)
Supplement: Supplementary file 6 — Additional file 6. STROBE Statement checklist of items that should be included in reports of observational studies. [file 41256_2024_387_MOESM6_ESM.docx]

STROBE Statement—checklist of items that should be included in reports of observational studies

|  | Item No. | Recommendation | Page  No. | Relevant text from manuscript |
| --- | --- | --- | --- | --- |
| **Title and abstract** | 1 | (*a*) Indicate the study’s design with a commonly used term in the title or the abstract | 1 | Barriers and Facilitators to Healthcare Services Utilization during the 2018-2020 Ebola Outbreak: A Qualitative Case Study in Beni and Butembo, Democratic Republic of Congo |
|  |  | (*b*) Provide in the abstract an informative and balanced summary of what was done and what was found | 2 | An Ebola Virus Disease (EVD) outbreak occurred in the Democratic Republic of Congo (DRC) between August 2018 and June 2020. This study aimed to explore the barriers and facilitators to utilizing routine healthcare services (HcS) during the outbreak in Beni and Butembo in North Kivu. |
| Introduction | | | |  |
| Background/rationale | 2 | Explain the scientific background and rationale for the investigation being reported | 4 | Several studies have addressed changes in HcS during outbreaks in general and Ebola outbreak in particular. Mutombo et al. explored communities experience with EVD, the support received by the participants when facing EVD, and participants’ perceptions regarding control measures in Kivu and Itur. In Sierra Leone, Carter et al., focused on patients with or suspected of Ebola disease. In this study, we investigated factors that motivated or demotivated non-Ebola patients to seek HcS |
| Objectives | 3 | State specific objectives, including any prespecified hypotheses | 4 | this study attempts to determine which factors influenced the treatment choices of non-Ebola patients during this outbreak. It should contribute to our understanding of how to strengthen HcS’ resilience as an imperative for achieving the third sustainable development goal |
| Methods | | | |  |
| Study design | 4 | Present key elements of study design early in the paper | 1, 2, 5 | A case study using the qualitative methods was conducted in Beni and Butembo cities. |
| Setting | 5 | Describe the setting, locations, and relevant dates, including periods of recruitment, exposure, follow-up, and data collection | 5,6 | 2.2.Study design: type, site, and study period  A case study using the qualitative methods was conducted in Beni and Butembo cities. As shown in Fig 1, these major cities were most severely affected by the 2018-2020 EVD outbreak and they comprised 3 health districts, namely Beni, Butembo and Katwa. These districts also provide care to patients referred from nearby health districts such as Mabalako (the location of the initial outbreak), Kalunguta, Oicha, Musienene and others. Data were collected from March to June 2020.  Recall bias was minimized by avoiding deaths that occurred more than six months prior. Therefore, we set a time limit from September 1, 2019 to February 29, 2020. |
| Participants | 6 | (*a*) *Cohort study*—Give the eligibility criteria, and the sources and methods of selection of participants. Describe methods of follow-up  *Case-control study*—Give the eligibility criteria, and the sources and methods of case ascertainment and control selection. Give the rationale for the choice of cases and controls  *Cross-sectional study*—Give the eligibility criteria, and the sources and methods of selection of participants | 6 | To be included in the study, the disease that led to death was required to be clinically distinct from Ebola. This included non-communicable diseases (e.g., hypertension, diabetes, cancer) or chronic communicable diseases such as HIV and tuberculosis. Trauma and obstetric emergencies were also considered. The deceased had to be admitted for at least 72 hours in a HcS. For security and logistic reasons, the deceased's household had to be located in Beni or Butembo cities. Recall bias was minimized by avoiding deaths that occurred more than six months prior. Therefore, we set a time limit from September 1, 2019 to February 29, 2020.  Deceased relatives were purposively selected among those who were available and consented to participate to the study, as they should have as much information as possible about the deceased, the illness, and the treatment. However, to respect our commitments to the Ethics Committee, those who were clearly affected by the death were excluded. In total, we included 24 relatives, representing one, two or three relatives per death. These KIs were purposively selected among providers, managers, and community leaders at provincial and local-levels. |
|  |  | (*b*) *Cohort study*—For matched studies, give matching criteria and number of exposed and unexposed  *Case-control study*—For matched studies, give matching criteria and the number of controls per case |  | N/A |
| Variables | 7 | Clearly define all outcomes, exposures, predictors, potential confounders, and effect modifiers. Give diagnostic criteria, if applicable | 2, 7 | The medical record reviews and interviews with the deceased's relatives focused on the illness history, in particular on complaints, care pathways, condition at admission, care, access to laboratory tests and drugs, social support; the perception of EVD's influence on the care outcome and causes or circumstances of death with family, as well as provider perspectives. Additional SSIs were conducted with key informants (KIs) selected among providers, HcS managers and community leaders, with a focus on their opinions on the preparedness, supply, use and quality of HcS before and during the EVD outbreak. |
| Data sources/ measurement | 8* | For each variable of interest, give sources of data and details of methods of assessment (measurement). Describe comparability of assessment methods if there is more than one group | *2, 7* | 2.4. Data collecting and sources  Patient data was collected through medical record reviews and semi-structured interviews (SSI) conducted with the deceased's relatives. |
| Bias | 9 | Describe any efforts to address potential sources of bias | 6 | Recall bias was minimized by avoiding deaths that occurred more than six months prior. |
| Study size | 10 | Explain how the study size was arrived at | 5, 6 | We purposely selected 15 deaths … We also believed this sample size was both large enough to have a diversity of patient profiles and small enough to be able to conduct in-depth analyses.  These KIs were purposively selected among providers, managers, and community leaders at provincial and local-levels. Although saturation was achieved with about 20 KIs (this was reflected in the repetition of the same information and the absence of new themes), the research team continued the interviews with up to 47 KIs to ensure a minimum number of respondents in the sub-samples. |

Continued on next page

| Quantitative variables | 11 | Explain how quantitative variables were handled in the analyses. If applicable, describe which groupings were chosen and why | 8-11 | The sociodemographic characteristics of the deceased, close relatives and KIs were entered and analyzed using Excel software. Mean was reported for age and length of service and proportions for all categorical variables. |
| --- | --- | --- | --- | --- |
| Statistical methods | 12 | (*a*) Describe all statistical methods, including those used to control for confounding | 8 | We used the thematic analysis which consisted in identifying, grouping and examining systematically and objectively the themes that emerged, in accordance with the themes of the theoretical framework and the objectives of this study. However, new themes that emerged were also considered. In this process, we looked to make visible the invisible dimensions of the participants’ experience, while remaining critical, neutral and reflexive as recommanded by Sundler et al. in their descriptive phenomenology |
|  |  | (*b*) Describe any methods used to examine subgroups and interactions | 8 | we used triangulation of methods (comparing declarations of the deceased's relatives with data from medical recorder) and triangulation of sources (by matching the opinions of relatives of the same deceased when we had two or three, the opinions of relatives with those of KIs and the opinions of KIs from different levels or affiliations) |
|  |  | (*c*) Explain how missing data were addressed |  | No applicable |
|  |  | (*d*) *Cohort study*—If applicable, explain how loss to follow-up was addressed  *Case-control study*—If applicable, explain how matching of cases and controls was addressed  *Cross-sectional study*—If applicable, describe analytical methods taking account of sampling strategy |  | No applicable |
|  |  | (*e*) Describe any sensitivity analyses |  | No applicable |
| Results | | | | |
| Participants | 13* | (a) Report numbers of individuals at each stage of study—eg numbers potentially eligible, examined for eligibility, confirmed eligible, included in the study, completing follow-up, and analysed | 9,10 | Of the 15 deaths, 9 were females and 6 males and their ages ranged from 7 to 79 years.  For the fifteen deaths, 23 relatives were interviewed, representing one, two or three relatives per deaths.  The 47 KIs were selected at the provincial level (9), from the health districts’ offices (11), health facilities (19) and from the community (8) |
|  |  | (b) Give reasons for non-participation at each stage | 5 | One informant did not wish to be recorded. He was not included in the study. |
|  |  | (c) Consider use of a flow diagram |  | No applicable |
| Descriptive data | 14* | (a) Give characteristics of study participants (eg demographic, clinical, social) and information on exposures and potential confounders | 9,10 | 3.1. Sample Description  Social and medical characteristics of the deceased |
|  |  | (b) Indicate number of participants with missing data for each variable of interest |  | No applicable |
|  |  | (c) *Cohort study*—Summarise follow-up time (eg, average and total amount) |  | No applicable |
| Outcome data | 15* | *Cohort study*—Report numbers of outcome events or summary measures over time |  | No applicable |
|  |  | *Case-control study—*Report numbers in each exposure category, or summary measures of exposure |  | No applicable |
|  |  | *Cross-sectional study—*Report numbers of outcome events or summary measures |  | No applicable |
| Main results | 16 | (*a*) Give unadjusted estimates and, if applicable, confounder-adjusted estimates and their precision (eg, 95% confidence interval). Make clear which confounders were adjusted for and why they were included |  | No applicable |
|  |  | (*b*) Report category boundaries when continuous variables were categorized |  | No applicable |
|  |  | (*c*) If relevant, consider translating estimates of relative risk into absolute risk for a meaningful time period |  | No applicable |

Continued on next page

| Other analyses | 17 | Report other analyses done—eg analyses of subgroups and interactions, and sensitivity analyses |  | No applicable |
| --- | --- | --- | --- | --- |
| Discussion | | | | |
| Key results | 18 | Summarise key results with reference to study objectives | 20 | We identied factors that had both a negative or positive influence on the availability, use and quality of HcS, and those could affect the patient’s satisfaction. Some of these factors were constant throughout the outbreak while others were at specific times, mainly at the early period of the outbreak or when new innovations were introduced in the outbreak response policy. |
| Limitations | 19 | Discuss limitations of the study, taking into account sources of potential bias or imprecision. Discuss both direction and magnitude of any potential bias | 25-26 | **Study limitations**  All cases included in this study ended in death after attending HcS. |
| Interpretation | 20 | Give a cautious overall interpretation of results considering objectives, limitations, multiplicity of analyses, results from similar studies, and other relevant evidence | 20 - 26 | **Discussion** |
| Generalisability | 21 | Discuss the generalisability (external validity) of the study results | 26 | Due to the small sample size and the limited number of sites, the results of this study cannot be generalized to other entities. However, this other study limitation was minimized by including more KIs from the community and through saturation achieved during data collection. |
| Other information | |  | | |
| Funding | 22 | Give the source of funding and the role of the funders for the present study and, if applicable, for the original study on which the present article is based | 27 | No direct funding was provided to this paper. However, to collect data, the principal investigator jumped on the opportunity to be in the field with a larger research project funded by the Rapid Research Fund for Ebola which was supported by the International Development Research Centre (IDRC), Grant number, 108966-002, KAG and SMM. |

*Give information separately for cases and controls in case-control studies and, if applicable, for exposed and unexposed groups in cohort and cross-sectional studies.

**Note:** An Explanation and Elaboration article discusses each checklist item and gives methodological background and published examples of transparent reporting. The STROBE checklist is best used in conjunction with this article (freely available on the Web sites of PLoS Medicine at http://www.plosmedicine.org/, Annals of Internal Medicine at http://www.annals.org/, and Epidemiology at http://www.epidem.com/). Information on the STROBE Initiative is available at www.strobe-statement.org.
