# Supplementary material for: Barriers and facilitators to healthcare facility utilization by non-Ebola patients during the 2018–2020 Ebola outbreak in the Democratic Republic of Congo
Source: Glob Health Res Policy. 2024 Nov 19;9:47. doi: 10.1186/s41256-024-00387-6 (PMC11575170; doi:10.1186/s41256-024-00387-6)
Supplement: Supplementary file 7 — Additional file 7. Relatives Interviews Output files from Atlas-ti. [file 41256_2024_387_MOESM7_ESM.docx]

**All current quotations (1046). Quotation-Filter: All**

______________________________________________________________________

HU: Audit décès pendant Ebola

File: [C:\Users\Dr Gabriel Kyomba\Documents\Scientific Software\A...\Audit décès pendant Ebola.hpr7]

Edited by: Super

Date/Time: 2024-05-21 18:47:26

______________________________________________________________________

**P20: Ktw_HopMtda_Audit_dcd-11_Proche-1.doc - 20:14 [R : Oui elle parlait mais lors..] (55:55) (Super)**

Codes: [Admission_Accueil]

No memos

R : Oui elle parlait mais lorsque nous avons quitté le CTE la maladie avait commencé à monter vers les membres supérieurs. C’est au CTE que j’avais rencontré une dame soignante qui m’avait injurié mais à Matanda on nous avait bien accue

**P20: Ktw_HopMtda_Audit_dcd-11_Proche-1.doc - 20:13 [R : Elle disait que je n’avais..] (59:59) (Super)**

Codes: [Admission_Accueil]

No memos

R : Elle disait que je n’avais pas soigné l’enfant et que j’étais où pour ne pas faire soigner l’enfant. Je lui avais dit que j’étais à l’hôpital et lorsqu’on y est on doit suivre leurs recommandations, ils nous avaient dit qu’ils allaient amener un échantillon au laboratoire spécialisé pour qu’on nous donne de bons résultats si c’est quoi, c’est ce qu’on attendait et qu’on nous fasse le transfert. Et venir à Butembo le transport était très cher avec la moto c’était trente dollars et j’avais d’enfants trouver l’argent pour assurer le transport, ça pris encore un mois pour venir au soins

**P 7: Bbo_HgrKttmba_Audit_dcd-04_Proche-1.doc - 7:15 [R : A son arrivée à l’hôpital ..] (62:62) (Super)**

Codes: [Admission_Accueil]

No memos

R : A son arrivée à l’hôpital j’étais au chams dans la forêt de Magumu après Byakato. J’étais revenue pendant qu’elle venait de faire une semaine à l’hôpital.

**P 6: Bbo_HgrKttmba_Audit_dcd-03_Proche-3.doc - 6:49 [R : Jadis il n’y avait pas de ..] (193:193) (Super)**

Codes: [Admission_Accueil]

No memos

R : Jadis il n’y avait pas de lamentation par rapport à l’accueil, d’ailleurs c’était notre hôpital de référence, c’est pendant l’épidémie qu’il a eu de lamentation comme quoi les malades trainent au service de triage toute une journée et moi-même j’avais vécu cette expérience quand j’amenais mes deux enfants aux soins. On vous tri le matin jusqu’à 16h00 sans aller au CTE pour le prélèvement il y avait une dame qui était rentré chez lui avec son malade parce qu’elle avait passé toute la journée sans médicament, elle les avait avertis qu’elle rentrait et elle était partie sans traitement. Les prestataires l’ont laissé rentrer chez lui.

**P17: Ktw_HopMtda_Audit_dcd-05_Proche-3.doc - 17:11 [R. A Kitatumba, on nous avait ..] (49:49) (Super)**

Codes: [Admission_Accueil]

No memos

R. A Kitatumba, on nous avait accueillis mais on ne nous avait pas donné gratuitement des médicaments. On avait payé la facture avant d’être transférer ailleurs.

**P20: Ktw_HopMtda_Audit_dcd-11_Proche-1.doc - 20:10 [R : On nous avait bien accueil..] (47:47) (Super)**

Codes: [Admission_Accueil]

No memos

R : On nous avait bien accueilli et au même moment elle fut amenée aux soins intensifs.

**P20: Ktw_HopMtda_Audit_dcd-11_Proche-1.doc - 20:11 [R : On lui avait reçu à la réc..] (51:51) (Super)**

Codes: [Admission_Accueil]

No memos

R : On lui avait reçu à la réception lorsqu’on provenait du CTE, après avoir consommé une perfusion là-bas quelques minutes après les soignants l’on amené aux soins intensifs et de là qu’elle était décédée.

**P 2: Bbo_HgrKttmba_Audit_dcd-01_Proche-2.doc - 2:14 [R : Pour qu’il arrive à décéde..] (61:61) (Super)**

Codes: [Admission_Accueil]

No memos

R : Pour qu’il arrive à décéder, c’était d’abord la période de l’épidémie d’Ebola et pendant cette période les soignants ne se souciaient pas des malades. Lorsque vous arrivez avec le malade on lui laisse d’abord à l’entrée et parfois ils partent s’occuper des malades qui sont internés et au retour ils vont vous demander de lui amener au CTE et au retour de ce dernier c’est là qu’ils peuvent commencer son traitement. Le temps d’aller au CTE et revenir alors que le malade est gravement malade ça peut causer sa mort et peut être qu’il n’allait pas mourir.

**P 8: Bbo_HgrKttmba_Audit_dcd-04_Proche-2.doc - 8:13 [R : L’accueil était bon, et il..] (54:54) (Super)**

Codes: [Admission_Accueil]

No memos

R : L’accueil était bon, et ils avaient fait tout pour lui traiter convenablement, ce qui avais fait que sa santé puisse s’améliorer immédiatement. Elle commençait à collaborer et faire de mouvement deux jours après elle rechuta et c’était fini pour elle.

**P 7: Bbo_HgrKttmba_Audit_dcd-04_Proche-1.doc - 7:42 [R : Les gens se lamentaient so..] (175:175) (Super)**

Codes: [Admission_Accueil]

No memos

R : Les gens se lamentaient souvent par rapport au retard au service de triage, lorsque vous y arriver et que vous présentez la fièvre élevée on doit vous arrêter pour vous examiner si ce n’est ne pas la MVE et cela prend de temps pour quelqu’un qui est venu se faire soigner. Mais s’il n’y a pas de la fièvre élevée on était bien accueilli et bien soigné.

**P16: Ktw_HopMtda_Audit_dcd-05_Proche-2.doc - 16:17 [R. On accueille bien les malad..] (72:72) (Super)**

Codes: [Admission_Accueil]

No memos

R. On accueille bien les malades

**P 8: Bbo_HgrKttmba_Audit_dcd-04_Proche-2.doc - 8:14 [R : Oui et était bien prise en..] (58:58) (Super)**

Codes: [Admission_Accueil]

No memos

R : Oui et était bien prise en charge y compris même les examens de laboratoire

**P 7: Bbo_HgrKttmba_Audit_dcd-04_Proche-1.doc - 7:23 [R : Elle était bien accueillie..] (94:94) (Super)**

Codes: [Admission_Accueil]

No memos

R : Elle était bien accueillie

**P 7: Bbo_HgrKttmba_Audit_dcd-04_Proche-1.doc - 7:22 [R : A l’hôpital on accueillait..] (90:90) (Super)**

Codes: [Admission_Accueil]

No memos

R : A l’hôpital on accueillait bien les malades ordinaires

**P16: Ktw_HopMtda_Audit_dcd-05_Proche-2.doc - 16:38 [R. L’accueil est bon et on soi..] (201:201) (Super)**

Codes: [Admission_Accueil]

No memos

R. L’accueil est bon et on soigne bien les patients.

**P16: Ktw_HopMtda_Audit_dcd-05_Proche-2.doc - 16:39 [R. Certains disent qu’ils ne p..] (205:205) (Super)**

Codes: [Admission_Accueil]

No memos

R. Certains disent qu’ils ne peuvent plus fréquenter l’hôpital parce que là il y a les malades de l’Ebola surtout ceux- là qui avaient une température élevée.

**P 4: Bbo_HgrKttmba_Audit_dcd-03_Proche-1.doc - 4:54 [R : Moi quand je leurs avais v..] (224:224) (Super)**

Codes: [Admission_Accueil]

No memos

R : Moi quand je leurs avais vu, ils m’avaient bien accueilli mais les autres disent que les soignants n’accueillent pas bien les malades pendant Ebola même avant l’épidémie il y avait toujours ceux qui disaient que l’accueil n’était pas bon.

**P 4: Bbo_HgrKttmba_Audit_dcd-03_Proche-1.doc - 4:28 [R : Oui elle était bien accuei..] (117:117) (Super)**

Codes: [Admission_Accueil]

No memos

R : Oui elle était bien accueillie

**P18: Ktw_HopMtda_Audit_dcd-06_Proche-1.doc - 18:21 [R. L’accueil est bon ; lorsqu’..] (84:84) (Super)**

Codes: [Admission_Accueil]

No memos

R. L’accueil est bon ; lorsqu’on arrive on est bien reçu.

**P 5: Bbo_HgrKttmba_Audit_dcd-03_Proche-2.doc - 5:34 [R : Lorsqu’ils avaient vu son ..] (127:127) (Super)**

Codes: [Admission_Accueil]

No memos

R : Lorsqu’ils avaient vu son état de santé ils l’avaient immédiatement envoyé au CTE elle n’avait pas passé la nuit à Kitatumba

**P 3: Bbo_HgrKttmba_Audit_dcd-01_Proche-3.doc - 3:32 [R : Ils lui avaient accueilli ..] (126:126) (Super)**

Codes: [Admission_Accueil]

No memos

R : Ils lui avaient accueilli en lui laissant à l’entrée au service de triage, ils n’avaient pas cherché une chambre où il pouvait se reposer et l’équipe de la riposte vient lui prendre et l’amener au CTE durant trois jours et puis il lui retourne au service de triage. Et ce sont les soignants qui vont lui amener dans la salle pour le traitement.

**P19: Ktw_HopMtda_Audit_dcd-10_Proche-1.doc - 19:16 [R : L’accueil était très rapid..] (73:73) (Super)**

Codes: [Admission_Accueil]

No memos

R : L’accueil était très rapide. Les médecins n’avaient pas trainé pour les examens gynéco obstétrique. Après quelques minutes, on l’amena directement au bloc opératoire pour une intervention chirurgicale laquelle était finir par un décès maternel. Ils avaient vu que c’était une urgence

**P18: Ktw_HopMtda_Audit_dcd-06_Proche-1.doc - 18:65 [R. C’est bien, là c’est bien !..] (270:270) (Super)**

Codes: [Admission_Accueil]

No memos

R. C’est bien, là c’est bien ! Ils reçoivent les malades bien

**P 3: Bbo_HgrKttmba_Audit_dcd-01_Proche-3.doc - 3:33 [R : L’accueil n’était pas bon] (130:130) (Super)**

Codes: [Admission_Accueil]

No memos

R : L’accueil n’était pas bon

**P 6: Bbo_HgrKttmba_Audit_dcd-03_Proche-3.doc - 6:26 [R : A cette période nous étion..] (103:103) (Super)**

Codes: [Admission_Accueil]

No memos

R : A cette période nous étions très déçus de leurs accueils, on ne sait pas si c’est à cause d’Ebola qu’ils étaient perturbés. Même les malades ordinaires étaient négligés et c’est la raison qui faisait qu’on fréquentait les postes de santé. Après le décès de consolée et moi-même être soignée il eut des enfants qui étaient venus avec un état de sante nutritionnel très aigus, ils étaient envoyés au CTE pour les prélèvements alors que c’était un problème nutritionnel. Et ils avaient fait deux jours sans traitement au CTE comme ils avaient beaucoup de malades

**P17: Ktw_HopMtda_Audit_dcd-05_Proche-3.doc - 17:32 [R. A Kitatumba, lorsque les in..] (137:137) (Super)**

Codes: [Admission_Accueil]

No memos

R. A Kitatumba, lorsque les infirmiers arrivaient dans notre salle, on nous observait et on nous donner ce dont nous avions besoins en soins.

**P17: Ktw_HopMtda_Audit_dcd-05_Proche-3.doc - 17:12 [R. Nous étions bien accueilli,..] (53:53) (Super)**

Codes: [Admission_Accueil]

No memos

R. Nous étions bien accueilli, mais le Médecin était à Goma raison pour laquelle on nous avait demandé d’attendre son retour pour sa meilleure prise en charge

**P 6: Bbo_HgrKttmba_Audit_dcd-03_Proche-3.doc - 6:29 [R : L’accueil était très bon c..] (115:115) (Super)**

Codes: [Admission_Accueil]

No memos

R : L’accueil était très bon c’est la structure où on nous soigne, c’est l’épidémie qui a tout perturbé et tout le monde avait peur.

**P 5: Bbo_HgrKttmba_Audit_dcd-03_Proche-2.doc - 5:58 [R : C’est seulement celle-là q..] (222:222) (Super)**

Codes: [Admission_Accueil]

No memos

R : C’est seulement celle-là qui était malade je n’avais pas vu cette place.

**P 5: Bbo_HgrKttmba_Audit_dcd-03_Proche-2.doc - 5:57 [R : A cette période je ne fréq..] (218:218) (Super)**

Codes: [Admission_Accueil]

No memos

R : A cette période je ne fréquentais pas l’hôpital.

**P17: Ktw_HopMtda_Audit_dcd-05_Proche-3.doc - 17:33 [R. L’accueil était conditionne..] (141:141) (Super)**

Codes: [Admission_Accueil]

No memos

R. L’accueil était conditionnel, si un malade envisageait obtenir gratuitement des soins, il ne pouvait pas être guéri. Mais par contre si l’on veut être guéri, il fallait payer son argent pour les soins, c’est ce que j’entendais dire les autres. Si un malade est venu pour être soigné gratuitement, il ne pouvait pas être bien soigné, mais quand on paye son argent, on sera vite guéri.

**P17: Ktw_HopMtda_Audit_dcd-05_Proche-3.doc - 17:34 [R. A Kalungamakuha, en cas d’a..] (145:145) (Super)**

Codes: [Admission_Accueil]

No memos

R. A Kalungamakuha, en cas d’absence du Médecin, les malades ne sont pas bien prise en charge et ne sauront pas leurs résultats. Le malade était censé connaitre seulement ce qui se passe dans la salle où il est interné mais ce qui se passe en dehors de cette salle, on ne sait pas.

**P11: Ktw_Hgr_Audit_dcd-08_Proche-1.doc - 11:41 [R/ ils nous avaient bien accue..] (168:168) (Super)**

Codes: [Admission_Accueil]

No memos

R/ ils nous avaient bien accueilli en nous guidant, les portes auxquelles nous allons entrer une après une autre pour voir les Médecins.

**P11: Ktw_Hgr_Audit_dcd-08_Proche-1.doc - 11:40 [R / Ils nous avaient bien accu..] (164:164) (Super)**

Codes: [Admission_Accueil]

No memos

R / Ils nous avaient bien accueilli bien qu’ils nous avaient fait payer beaucoup d’argents quand l’enfant quitter.

**P14: Ktw_Hgr_Audit_dcd-09_Proche-1.doc - 14:36 [R : - Avant la période de l’ép..] (152:153) (Super)**

Codes: [Admission_Accueil]

No memos

R : - Avant la période de l’épidémie l’hôpital de KATWA était bien parce que tous mes enfants y étaient nés, d’ailleurs j’y ai été opérée deux fois pour la césarienne et cela où ils se font soigner, pas de commentaire pour l’accueil et les soins. On arrive à l’hôpital les soignants se préoccupaient de vous et là on sentait la guérison. On pouvait disparaitre et on va comprendre que c’arrive.

- Mais pendant la période de l’épidémie, pas de bonne prise en charge parce que ils nous donnaient la moitié du traitement sans même se soucier des patients. Il n’y avait plus des visites des soignants aux des patients pour leurs réconforter. On laisser les médicaments au garde malade et c’est lui qui doit veillez pour donner à son patient. Ils travaillaient avec une démotivation comme s’ils n’étaient pas rémunérer. Par exemple si je cultivais gratuitement pour vous ça ne sera pas comme celle qui cultive pour l’argent.

**P15: Ktw_HopMtda_Audit_dcd-05_Proche-1.doc - 15:32 [R. Les malades et les autres v..] (125:125) (Super)**

Codes: [Admission_Accueil]

No memos

R. Les malades et les autres visiteurs étaient vraiment bien accueillis.

**P12: Ktw_Hgr_Audit_dcd-08_Proche-12.doc - 12:13 [R : Pour l’accueil c’est bon m..] (58:58) (Super)**

Codes: [Admission_Accueil]

No memos

R : Pour l’accueil c’est bon mais lorsqu’ils vous donnent la chambre ils ne se soucient plus de vous.

**P11: Ktw_Hgr_Audit_dcd-08_Proche-1.doc - 11:31 [R/ Bien, les infirmiers se son..] (128:128) (Super)**

Codes: [Admission_Accueil]

No memos

R/ Bien, les infirmiers se sont souciés de mon malade contrairement des autres du Canadien où j’étais passé.

**P11: Ktw_Hgr_Audit_dcd-08_Proche-1.doc - 11:38 [R/ ils m’avaient bien accueill..] (156:156) (Super)**

Codes: [Admission_Accueil]

No memos

R/ ils m’avaient bien accueilli mais il n’y avait plus des soins gratuits, ils nous avaient demandé l’argent juste au début.

**P20: Ktw_HopMtda_Audit_dcd-11_Proche-1.doc - 20:28 [R : Dans cette formation sanit..] (127:127) (Super)**

Codes: [Admission_Accueil]

No memos

R : Dans cette formation sanitaire il n’y a pas de problème nous nous sommes soignés sans difficulté on nous a accueilli et on soigne sans condition. Avant Ebola on ne peut pas savoir par ce que nous étions à Mangrujipa.

**P 1: Bbo_HgrKttmba_Audit_dcd-01_Proche-1.doc - 1:17 [RÉPONDANT : Non lui il était m..] (53:53) (Super)**

Codes: [Admission_Accueil]

No memos

RÉPONDANT : Non lui il était mis à côté.

**P11: Ktw_Hgr_Audit_dcd-08_Proche-1.doc - 11:32 [R/ Non, ici on n’hospitalisait..] (132:132) (Super)**

Codes: [Admission_Accueil]

No memos

R/ Non, ici on n’hospitalisait plus encore.

**P 1: Bbo_HgrKttmba_Audit_dcd-01_Proche-1.doc - 1:18 [RÉPONDANT : Je dirais que la f..] (56:56) (Super)**

Codes: [Admission_Accueil]

No memos

RÉPONDANT : Je dirais que la façon dont on nous a accueillie n’était bonne par ce qu’il y a une différence entre les personnels soignants actuel et ceux de l’époque. Actuellement lorsque vous arrivez avec le malade on vous indique le lieu où vous allez lui mettre et ils viendront avec un retard peut être qu’ils ont peur de la maladie mais jadis ce n’était pas le cas même si l’enclos était élevé à l’époque lorsqu’ils vous apercevaient ils couraient vite auprès de vous pour récupérer le malade et commencer à lui traiter. Bref l’accueil a changé, peut-être ils ont eu peur de l’épidémie de la Maladie à Virus Ebola.

**P15: Ktw_HopMtda_Audit_dcd-05_Proche-1.doc - 15:41 [R. Huu !] (163:163) (Super)**

Codes: [Admission_Accueil]

No memos

R. Huu !

**P15: Ktw_HopMtda_Audit_dcd-05_Proche-1.doc - 15:64 [R. Chacun est satisfait de ser..] (261:261) (Super)**

Codes: [Admission_Accueil]

No memos

R. Chacun est satisfait de services rendus qu’on lui a offert.

**P14: Ktw_Hgr_Audit_dcd-09_Proche-1.doc - 14:9 [R : Oui] (37:37) (Super)**

Codes: [Admission_Accueil]

No memos

R : Oui

**P13: Ktw_Hgr_Audit_dcd-08_Proche-2.doc - 13:11 [R : Normalement pendant la pér..] (45:45) (Super)**

Codes: [Admission_Accueil]

No memos

R : Normalement pendant la période de l’épidémie d’Ebola, les soignants négligeaient les patients en disant qu’ils ne sont pas bien rémunérés, ils étaient trop lents, ils soignaient avec retard. Et après la mort de l’enfant, la facture a été majorée jusqu’à quatre cent Cinquante dollars (450$). C’était cher.

**P13: Ktw_Hgr_Audit_dcd-08_Proche-2.doc - 13:33 [R : Avant l’épidémie les prest..] (136:137) (Super)**

Codes: [Admission_Accueil]

No memos

R : Avant l’épidémie les prestataires étaient souples l’accueil était très bon peut-être ils savaient que le patient devrait leurs satisfaire par rapport à la motivation et même la prise en charge était très bonne ils se donnaient au patient pour une meilleure satisfaction.

Pendant l’épidémie au niveau des structures il y avait une méfiance totale et pour la prise en charge il y avait une lenteur du côté des soignants. Pas d’hygiène dans les salles, il fallait voir les latrines de Katwa et lorsqu’on demandait on nous répondait que tous les soignants sont partis dans la riposte on a plus les agents ici.

**P15: Ktw_HopMtda_Audit_dcd-05_Proche-1.doc - 15:65 [R. Oui, ils sont bons. Les aut..] (265:265) (Super)**

Codes: [Admission_Accueil]

No memos

R. Oui, ils sont bons. Les autres disent cela

**P12: Ktw_Hgr_Audit_dcd-08_Proche-12.doc - 12:39 [R : C’est son petit-fils de Be..] (167:167) (Super)**

Codes: [Admission_Accueil]

No memos

R : C’est son petit-fils de Beni qui avait décidé qu’on lui amène à Katwa comme c’est là où il avait fini ses études médicales, alors il avait appelé qu’il faut amener sa grand-mère à Katwa.

**P10: Ktw_Hgr_Audit_dcd-02_Proche-1.doc - 10:10 [R : Nous n’avons pas constaté ..] (41:41) (Super)**

Codes: [Admission_Accueil]

No memos

R : Nous n’avons pas constaté quelque chose qui pouvait nous effrayer ou qui pouvait nous mettre dans un doute, à notre arrivé eux aussi avaient commencé leur travail.

**P10: Ktw_Hgr_Audit_dcd-02_Proche-1.doc - 10:9 [R : L’accueil était bon, à not..] (37:37) (Super)**

Codes: [Admission_Accueil]

No memos

R : L’accueil était bon, à notre arriver comme c’était la nuit, nous étions accueillis au triage et immédiatement on nous amena dans la salle d’urgence où on avait les différents examens et après on nous donna une chambre. Lorsqu’ils ont trouvé que certains examens nécessitent un certains un suivi approprié on nous amena dans les soins intensifs ou nous avons passé une nuit et le matin on retourna dans la chambre. Trois fois de mouvement au soins intensifs, ils décidèrent que nous restions dans ce dernier. Durant Six jours dans ce service c’était le 1^er^ janvier à Onze heure il décéda.

**P12: Ktw_Hgr_Audit_dcd-08_Proche-12.doc - 12:38 [R : Je peux raconter ce que j’..] (163:163) (Super)**

Codes: [Admission_Accueil]

No memos

R : Je peux raconter ce que j’ai vécu sur l’hospitalisation de ma mère Pendant Ebola je vous ai déjà dit ce que je sais, l’accueil était bon sauf la prise en charge qui était mauvaise par la qualité des soins et la négligence des patients par les soignants. Avant on ne tombait pas malade mais tous étaient bons au niveau de la structure par ce que le patient était plus considéré dans sa prise en charge. Seulement à Katwa où ils ont toujours négligé les patients, d’ailleurs je me fais soigner à l’hôpital Matanda.

**P15: Ktw_HopMtda_Audit_dcd-05_Proche-1.doc - 15:42 [R. La qualité de service est v..] (166:166) (Super)**

Codes: [Admission_Accueil]

No memos

R. La qualité de service est vraiment bonne.

**P14: Ktw_Hgr_Audit_dcd-09_Proche-1.doc - 14:8 [R : L’accueil était bon lorsqu..] (33:33) (Super)**

Codes: [Admission_Accueil] [Investigation_Ebola]

No memos

R : L’accueil était bon lorsque vous veniez avec le document du CTE, mais si vous arrivez sans papier du CTE l’accueil n’était pas bon. Même pour la deuxième fois que nous y étions j’avais dit à la petite sœur de préparer ses papiers du CTE et là on nous avait bien accueillis pour ce qui est de la prise en charge, je dirais que les soins entaient favorables dans les soins intensifs seulement mais dans les salles ou chambres d’hospitalisations pas de soins.

**P11: Ktw_Hgr_Audit_dcd-08_Proche-1.doc - 11:61 [R : Katwe était un meilleur hô..] (247:247) (Super)**

Codes: [Admission_Accueil] [Prise en charge_Médicale]

No memos

R : Katwe était un meilleur hôpital c’est là que cet enfant était né, peut-être qu’ils pourront bien nous soigner après la fin de cette épidémie, mais pendant l’épidémie vous savez que les soins gratuits ont de conséquences, tantôt ils vous soignaient bien tantôt on vous soigne mal, mais malgré lors de notre hospitalisation il n’avait plus les soins gratuits parce que j’avais payé beaucoup d’argent pour ce qui concerne la facture.

**P 4: Bbo_HgrKttmba_Audit_dcd-03_Proche-1.doc - 4:37 [R : Lui prenais son traitement..] (152:152) (Super)**

Codes: [Attitudes-Pratiques_Défunt]

No memos

R : Lui prenais son traitement sa difficulté par ce qu’il ya des malades que refuse les médicaments

**P 9: Bbo_HopScola_Audit_dcd-07_Proche-1.doc - 9:43 [R : Son garde disait qu’il pre..] (199:199) (Super)**

Codes: [Attitudes-Pratiques_Défunt]

No memos

R : Son garde disait qu’il prenait toujours ses médicaments, car on n’était pas là quand il prenait ses médicaments.

**P 5: Bbo_HgrKttmba_Audit_dcd-03_Proche-2.doc - 5:53 [R : Oui elle prenait le traite..] (202:202) (Super)**

Codes: [Attitudes-Pratiques_Défunt]

No memos

R : Oui elle prenait le traitement mais lorsqu’il y avait la crise épileptique elle n’était pas en mesure de prendre son médicament on essayait d’ouvrir la bouche mais sans ne s’ouvrait.

**P 4: Bbo_HgrKttmba_Audit_dcd-03_Proche-1.doc - 4:45 [R : Ce sont ses enfants qui dé..] (184:184) (Super)**

Codes: [Attitudes-Pratiques_Défunt]

No memos

R : Ce sont ses enfants qui dérangeaient la famille seulement, par ce qu’ils n’avaient pas un seul père.

**P 6: Bbo_HgrKttmba_Audit_dcd-03_Proche-3.doc - 6:38 [R : Oui, elle prenait bien le ..] (147:147) (Super)**

Codes: [Attitudes-Pratiques_Défunt]

No memos

R : Oui, elle prenait bien le médicament.

**P 2: Bbo_HgrKttmba_Audit_dcd-01_Proche-2.doc - 2:36 [R : Avant l’épidémie les perso..] (149:149) (Super)**

Codes: [Attitudes_Prestataires_Avant MVE]

No memos

R : Avant l’épidémie les personnels soignants étaient très accueillants et soignaient immédiatement et le malade pouvait guérir. Ils pouvaient toucher le malade sans problème Ils n’avaient pas de doute sur le malade. Mais pendant la période d’Ebola on ne pouvait pas toucher un malade et si tu lui touche et qu’il est porteur du virus Ebola, là tu meurs aussi. C’est pourquoi ils attendaient l’autorisation du CTE pour soigner les malades. Ils disaient qu’ils ne sont pas autorisés de toucher le malade.

**P 3: Bbo_HgrKttmba_Audit_dcd-01_Proche-3.doc - 3:50 [R : Ils entaient bien, ils acc..] (198:198) (Super)**

Codes: [Attitudes_Prestataires_Avant MVE]

No memos

R : Ils entaient bien, ils accueillaient et traiter les patients sans problème mais actuellement c’est très grave par ce que là où tu es arrivé et c’est là qu’on te laisse jusqu’à ce que vous y quittez vers le CTE tu peux mourir ou guérir.

**P 2: Bbo_HgrKttmba_Audit_dcd-01_Proche-2.doc - 2:35 [R : On ne sait pas s’ils étaie..] (145:145) (Super)**

Codes: [Attitudes_Prestataires_Pendant MVE]

No memos

R : On ne sait pas s’ils étaient payés, le fait de ne pas s’occupait de nous on pensait qu’ils n’étaient pas payés.

**P 2: Bbo_HgrKttmba_Audit_dcd-01_Proche-2.doc - 2:34 [R : Pour ce la non.] (141:141) (Super)**

Codes: [Attitudes_Prestataires_Pendant MVE]

No memos

R : Pour ce la non.

**P 2: Bbo_HgrKttmba_Audit_dcd-01_Proche-2.doc - 2:33 [R : On ne sait pas si dans leu..] (137:137) (Super)**

Codes: [Attitudes_Prestataires_Pendant MVE]

No memos

R : On ne sait pas si dans leurs Comité il y avait quelqu’un à qui on pouvait s’adresser pour nous population ; on avait remarqué qu’ils nous négligeaient. On ne sait pas s’ils étaient rémunérés ou pas.

**P 2: Bbo_HgrKttmba_Audit_dcd-01_Proche-2.doc - 2:37 [R : Oui cette maladie a été la..] (153:153) (Super)**

Codes: [Attitudes_Prestataires_Pendant MVE]

No memos

R : Oui cette maladie a été la cause de la négligence.

**P 3: Bbo_HgrKttmba_Audit_dcd-01_Proche-3.doc - 3:51 [R : Oui ils avaient peur aussi..] (202:202) (Super)**

Codes: [Attitudes_Prestataires_Pendant MVE]

No memos

R : Oui ils avaient peur aussi.

**P 3: Bbo_HgrKttmba_Audit_dcd-01_Proche-3.doc - 3:52 [R : Nous nous n’avons pas eu p..] (206:206) (Super)**

Codes: [Attitudes_Prestataires_Pendant MVE]

No memos

R : Nous nous n’avons pas eu peur, ce sont eux qui avaient peur de gens.

**P 2: Bbo_HgrKttmba_Audit_dcd-01_Proche-2.doc - 2:32 [R : Il parait dans cette pério..] (133:133) (Super)**

Codes: [Attitudes_Prestataires_Pendant MVE]

No memos

R : Il parait dans cette période les soins étaient gratuits et les personnels soignants n’étaient pas motivés à cause de la gratuité pendant l’épidémie et négligeaient les patients. Même un malade pouvait rechuter et on part appeler le personnel soignant il avait de fiers comme si rien n’était.

**P10: Ktw_Hgr_Audit_dcd-02_Proche-1.doc - 10:30 [R : Ça dépend d’une personne à..] (120:120) (Super)**

Codes: [Commentaire_A ajouter]

No memos

R : Ça dépend d’une personne à l’autre par ce qu’il y a des prestataires qui ont des caprices et de malades qui ont des caprices, s’ils ne s’entendent pas ça devient un problème alors que vous êtes sensé d’avoir une bonne compréhension pour la bonne prise en charge.

**P14: Ktw_Hgr_Audit_dcd-09_Proche-1.doc - 14:44 [R : Vous savez que ceux qui tr..] (186:186) (Super)**

Codes: [Commentaire_A ajouter]

No memos

R : Vous savez que ceux qui travaillent dans la base communautaire il y a une personne qui travaille dans chaque aire de santé sur 12 RECO.

**P 3: Bbo_HgrKttmba_Audit_dcd-01_Proche-3.doc - 3:61 [R : Nous demandons aux prestat..] (247:247) (Super)**

Codes: [Commentaire_A ajouter]

No memos

R : Nous demandons aux prestataires de soins de continuer à soigner comme avant l’épidémie par ce que beaucoup de gens ont perdu leurs vies à cause de la peur des soignants. Lorsque on part à l’hôpital vous garde à coté alors qu’on est venu au soins.

**P14: Ktw_Hgr_Audit_dcd-09_Proche-1.doc - 14:43 [R : Même celui qui a travaillé..] (182:182) (Super)**

Codes: [Commentaire_A ajouter]

No memos

R : Même celui qui a travaillé un mois jusqu’à présent il continue à gagner l’argent selon la communauté. Pour l’équipe de la riposte il fallait chercher comment encadrer les sensibilisateurs(RECO) en leurs donnant une occupation par ce que partout où nous passons nous sommes mal vu les gens disent (Abakaya eshebola) que nous continuons à « manger l’argent d’Ebola » alors que nous avions travaillé qu’un seul ou deux mois. Qu’ils nous aident avec un travail.

**P18: Ktw_HopMtda_Audit_dcd-06_Proche-1.doc - 18:79 [00 :32 :45 R. Concernant quoi ..] (326:327) (Super)**

Codes: [Commentaire_A ajouter]

No memos

00 :32 :45

R. Concernant quoi ?

**P13: Ktw_Hgr_Audit_dcd-08_Proche-2.doc - 13:39 [R : Il Fallait que les Centre ..] (161:161) (Super)**

Codes: [Commentaire_A ajouter]

No memos

R : Il Fallait que les Centre de santé de référence qui sont placés dans la communauté aient un suivi qui est approprié plus que les hôpitaux parce que ce sont eux qui reçoivent les malades pour les premiers soins et surtout comme le service du laboratoire doit être bien perfectionné pour donner les bons résultats qui permettraient le bon traitement du patient ou même transférer les cas à temps vers les grandes structures pour les soins appropriés

**P15: Ktw_HopMtda_Audit_dcd-05_Proche-1.doc - 15:74 [R. Non] (298:298) (Super)**

Codes: [Commentaire_A ajouter]

No memos

R. Non

**P18: Ktw_HopMtda_Audit_dcd-06_Proche-1.doc - 18:80 [R. Concernant les prestataires..] (331:331) (Super)**

Codes: [Commentaire_A ajouter]

No memos

R. Concernant les prestataires des soins je les interpelle à nous recevoir comme jadis parce que le fait que l’on ne recevait plus bien les malades, cela amenait que certains aillent suivre des soins dans des postes de santé où ils seront bien reçus en passant toujours par le triage. Qu’ils nous reçoivent vraiment bien et cela va encore susciter l’esprit de recourir aux soins dans les hôpitaux

**P 8: Bbo_HgrKttmba_Audit_dcd-04_Proche-2.doc - 8:41 [R : Non] (168:168) (Super)**

Codes: [Commentaire_A ajouter]

No memos

R : Non

**P 8: Bbo_HgrKttmba_Audit_dcd-04_Proche-2.doc - 8:40 [R : Pour le décès de maman non..] (164:164) (Super)**

Codes: [Commentaire_A ajouter]

No memos

R : Pour le décès de maman non. Cela était observé pour les autres.

**P16: Ktw_HopMtda_Audit_dcd-05_Proche-2.doc - 16:50 [R. Non, Seulement dire que les..] (248:248) (Super)**

Codes: [Commentaire_A ajouter]

No memos

R. Non, Seulement dire que les gens avaient peur du CTE sans fondement

**P 6: Bbo_HgrKttmba_Audit_dcd-03_Proche-3.doc - 6:59 [R : En cas d’épidémie, il faud..] (236:236) (Super)**

Codes: [Commentaire_A ajouter]

No memos

R : En cas d’épidémie, il faudrait commencer le processus et les décisions par la base par ce qu’au niveau de la base les gens se connaissent entre eux ; ça pour permettre une mobilisation pour lutter contre cette épidémie

**P 4: Bbo_HgrKttmba_Audit_dcd-03_Proche-1.doc - 4:63 [R : S’il s’agit d’une épidémie..] (260:260) (Super)**

Codes: [Commentaire_A ajouter]

No memos

R : S’il s’agit d’une épidémie il faut que les soignants puissent chaque fois sensibiliser la communauté avant son éclosion pour qu’il n’ait pas la trouble comme la fois passée, parce les uns disaient que ce n’était pas une maladie alors il y avait une confusion.

**P 9: Bbo_HopScola_Audit_dcd-07_Proche-1.doc - 9:65 [R : Non.] (292:292) (Super)**

Codes: [Commentaire_A ajouter]

No memos

R : Non.

**P11: Ktw_Hgr_Audit_dcd-08_Proche-1.doc - 11:70 [R : Non ;] (283:283) (Super)**

Codes: [Commentaire_A ajouter]

No memos

R : Non ;

**P17: Ktw_HopMtda_Audit_dcd-05_Proche-3.doc - 17:42 [R. Oui, j’ai parlé ce qui étai..] (177:177) (Super)**

Codes: [Commentaire_A ajouter]

No memos

R. Oui, j’ai parlé ce qui était dans ma tête.

**P10: Ktw_Hgr_Audit_dcd-02_Proche-1.doc - 10:29 [R : je ne n’ai pas de commenta..] (116:116) (Super)**

Codes: [Commentaire_A ajouter]

No memos

R : je ne n’ai pas de commentaire seulement que nous sommes dans une société reculée ou il y manque de civilisation et souvent il peut y avoir certaines difficultés et cela dépend d’une personne à l’autre et d’un cas à l’autre. Le savoir-faire et le savoir-vivre peuvent vous ouvrir certaines opportunités et vous vous sentez à l’aise là où l’autre ne pourra pas se sentir à l’aise. Le Soignant peut avoir ses caprices et si vous vous adaptez la tache devient facile et là où vous pourriez avoir des problèmes les choses marchent facilement. Certains rencontrent les difficultés par manque de savoir-faire et savoir vivre. Les hôpitaux que nous avons c’est comme ça qu’ils sont peut-être qu’il faut les renforcer ou les équiper. Nous leurs avons remercié et nous continuons à collaborer au cause de cette durée que nous avions passée ensemble.

**P 7: Bbo_HgrKttmba_Audit_dcd-04_Proche-1.doc - 7:55 [R : Non] (226:226) (Super)**

Codes: [Commentaire_A ajouter]

No memos

R : Non

**P 5: Bbo_HgrKttmba_Audit_dcd-03_Proche-2.doc - 5:78 [R : Les prestataires de soins ..] (302:302) (Super)**

Codes: [Commentaire_A ajouter]

No memos

R : Les prestataires de soins ont étudié pour ça, ils peuvent nous dires ceux nous devons faire pour se protéger nous n’avons pas étudié la médecine pour pouvoir donner des orientations par rapport au soins.je ne pas beaucoup à dire.

**P20: Ktw_HopMtda_Audit_dcd-11_Proche-1.doc - 20:33 [R : Rien] (146:146) (Super)**

Codes: [Commentaire_A ajouter]

No memos

R : Rien

**P19: Ktw_HopMtda_Audit_dcd-10_Proche-1.doc - 19:36 [R : Non.] (170:170) (Super)**

Codes: [Commentaire_A ajouter]

No memos

R : Non.

**P 2: Bbo_HgrKttmba_Audit_dcd-01_Proche-2.doc - 2:46 [R : Je vous demande de fournir..] (189:189) (Super)**

Codes: [Commentaire_A ajouter]

No memos

R : Je vous demande de fournir votre effort pour qu’une autre épidémie ne réapparait plus pour que les gens ne meurent plus. Et si les soignants n’ont pas de salaire, qu’on leurs donne par ce que c’est parmi les causes de la négligence.

**P 1: Bbo_HgrKttmba_Audit_dcd-01_Proche-1.doc - 1:38 [RÉPONDANT : Aujourd’hui il y a..] (137:137) (Super)**

Codes: [Commentaire_A ajouter]

No memos

RÉPONDANT : Aujourd’hui il y a les maladies qui apparaissent et on ne sait pas que peut etre malade ou pas, il faut que la hiérarchie conseil ses personnel soignants pour qu’ils accueillent les patients par ce que si un malade venait et que les soignants ne veulent pas s’approcher de lui, ils peuvent occasionner même son décès. Par ce que dans lui il va penser qu’il est porteur d’un virus et ça lui affecte.

**P 3: Bbo_HgrKttmba_Audit_dcd-01_Proche-3.doc - 3:25 [R : C’est chez Ebola ; on te r..] (98:98) (Super)**

Codes: [CTE_connaissance-opinion]

No memos

R : C’est chez Ebola ; on te renvoie et là on est obligé de rentrer à la maison pour ne pas aller au CTE.

**P 2: Bbo_HgrKttmba_Audit_dcd-01_Proche-2.doc - 2:15 [R : C’est une structure médica..] (65:65) (Super)**

Codes: [CTE_connaissance-opinion]

No memos

R : C’est une structure médicale qui s’occupe d’Ebola, et donc tu ne peux pas recevoir le traitement sans passer au CTE, même si vous arrivez en urgence on doit vous demander si vous êtes passé au CTE

**P 3: Bbo_HgrKttmba_Audit_dcd-01_Proche-3.doc - 3:53 [R : Par ce que beaucoup de gen..] (210:210) (Super)**

Codes: [CTE_connaissance-opinion]

No memos

R : Par ce que beaucoup de gens venaient d’y laisser leurs vies.et nombreux avaient la tension et lorsque on lui laisse au triage sans traitement et qu’on l’amène au CTE c’est pourquoi ils mouraient.

**P 9: Bbo_HopScola_Audit_dcd-07_Proche-1.doc - 9:57 [R : Il y’avait trop de rumeurs..] (257:257) (Super)**

Codes: [CTE_connaissance-opinion]

No memos

R : Il y’avait trop de rumeurs disant qu’on tuait les gens avec le marteau, et d’autres disent qu’il y’avait des comprimés, qu’ils utilisaient pour tuait les patients.

**P 3: Bbo_HgrKttmba_Audit_dcd-01_Proche-3.doc - 3:30 [R : Il avait fait trois jours...] (118:118) (Super)**

Codes: [CTE_connaissance-opinion]

No memos

R : Il avait fait trois jours.

**P 9: Bbo_HopScola_Audit_dcd-07_Proche-1.doc - 9:59 [R : Aujourd’hui les habitants ..] (266:266) (Super)**

Codes: [CTE_connaissance-opinion]

No memos

R : Aujourd’hui les habitants ont compris que la maladie était réelle car il y’a même de ces malades qui partent eux-mêmes aux CTE.

**P 9: Bbo_HopScola_Audit_dcd-07_Proche-1.doc - 9:60 [R : Au paravent les gens disai..] (270:270) (Super)**

Codes: [CTE_connaissance-opinion]

No memos

R : Au paravent les gens disaient que la maladie n’existe pas.

**P 3: Bbo_HgrKttmba_Audit_dcd-01_Proche-3.doc - 3:27 [R : Oui il mourait par ce que ..] (106:106) (Super)**

Codes: [CTE_connaissance-opinion]

No memos

R : Oui il mourait par ce que lui avait fait deux ou trois jours sans lui donner même un comprimé.et on lui amena à Kitatumba et trois jours après il mourut.

**P 3: Bbo_HgrKttmba_Audit_dcd-01_Proche-3.doc - 3:29 [R : Oui j’y étais partie lui v..] (114:114) (Super)**

Codes: [CTE_connaissance-opinion]

No memos

R : Oui j’y étais partie lui visiter

**P 9: Bbo_HopScola_Audit_dcd-07_Proche-1.doc - 9:61 [R : Ils disaient que c’étaient..] (274:275) (Super)**

Codes: [CTE_connaissance-opinion]

No memos

R : Ils disaient que c’étaient le marteau et le comprime rouge.

00 :25 :08

**P 3: Bbo_HgrKttmba_Audit_dcd-01_Proche-3.doc - 3:26 [R : On avait peur d’y aller pa..] (102:102) (Super)**

Codes: [CTE_connaissance-opinion]

No memos

R : On avait peur d’y aller par ce que chaque personne qu’on amenait devait mourir.

**P 9: Bbo_HopScola_Audit_dcd-07_Proche-1.doc - 9:58 [R : Non, sauf que les gens ne ..] (262:262) (Super)**

Codes: [CTE_connaissance-opinion]

No memos

R : Non, sauf que les gens ne voulaient pas y aller, et celui qui donnait cet avis était considéré comme ennemi de la communauté ici à Butembo et cela surtout au début de l’épidémie.

**P12: Ktw_Hgr_Audit_dcd-08_Proche-12.doc - 12:22 [R : La vie de son foyer avec s..] (94:94) (Super)**

Codes: [Décès_cause selon famille]

No memos

R : La vie de son foyer avec son mari.

**P 3: Bbo_HgrKttmba_Audit_dcd-01_Proche-3.doc - 3:46 [R : Il n’y avait pas d’autres ..] (182:182) (Super)**

Codes: [Décès_cause selon famille]

No memos

R : Il n’y avait pas d’autres maladies aucune autre maladie qu’on nous avait informé

**P 3: Bbo_HgrKttmba_Audit_dcd-01_Proche-3.doc - 3:45 [R : C’est sa maladie(Prostate)..] (178:178) (Super)**

Codes: [Décès_cause selon famille]

No memos

R : C’est sa maladie(Prostate) qui été la cause selon la famille.

**P18: Ktw_HopMtda_Audit_dcd-06_Proche-1.doc - 18:54 [R. En famille, rien n’a été di..] (217:217) (Super)**

Codes: [Décès_cause selon famille]

No memos

R. En famille, rien n’a été dit. Ses familiers regrettaient seulement la mort de la personne qui leur prenait en charge dans ses démarches.

**P12: Ktw_Hgr_Audit_dcd-08_Proche-12.doc - 12:21 [R : Nous en famille on savait ..] (90:90) (Super)**

Codes: [Décès_cause selon famille]

No memos

R : Nous en famille on savait que son problème était cette tension parce qu’elle ne mangeait pas les aliments contenant du seul. Mais lorsque nous sommes allés à l’hôpital on nous avait dit qu’il y avait un problème respiratoire et la tension. Lorsqu’elle était aux soins intensif on surveillait sa tension. Et surtout elle avait vécu une vie paisible sans la joie ni la paix dans son foyer, c’est ce qui avait provoqué sa maladie.

**P20: Ktw_HopMtda_Audit_dcd-11_Proche-1.doc - 20:18 [R : Nous c’est une dame qui no..] (74:74) (Super)**

Codes: [Décès_cause selon famille]

No memos

R : Nous c’est une dame qui nous avait combattu à Mangurujipa, elle avait commencé avec son père par ce que nous avions une buvette comme mon mari avait dit qu’il ne peut plus bleuir quelqu’un pour trouver quelque chose à manger dans la famille comme il y avait cette buvette, notre salaire vient à retard et cela où les enfants doivent être scolarisé et ils sont nombreux. Cette dame n’avait pas copris notre initiative comme elle avait la sienne , elle était en colère en disant que nous avons récupérer tous ses clients. Elle voulait faire de dispute souvent avec nous et un jour elle nous avait dit qu’elle va nous délocaliser dans le milieu. Elle avait commencé par mon mari et voilà qu’elle reste avec une seule jambe et en suite elle est partie également sur Angel.

**P20: Ktw_HopMtda_Audit_dcd-11_Proche-1.doc - 20:19 [R : Oui même hier nous avons t..] (82:82) (Super)**

Codes: [Décès_cause selon famille]

No memos

R : Oui même hier nous avons tonné sur cette dame croyant que c’est elle parce qu’actuellement elle cherche notre adresse pour venir demander pardon par ce qu’elle n’est pas en paix et on l’avait chassé dans le milieu, elle reste maintenant à Kitulu mais nous on n’accepte pas vu la perte de l’enfant et son père qui ne reste qu’avec une jambe quel genre du pardon ? Hier lorsque vous appelez on pensait que c’est elle et c’est pourquoi je vous demandais vous venais faire quoi ?

**P 2: Bbo_HgrKttmba_Audit_dcd-01_Proche-2.doc - 2:28 [R : La cause de son décès c’es..] (117:117) (Super)**

Codes: [Décès_cause selon famille]

No memos

R : La cause de son décès c’est cette prostate je ne sais aucune autre maladie.

**P12: Ktw_Hgr_Audit_dcd-08_Proche-12.doc - 12:23 [R : Non il ne lui touchait pas..] (98:98) (Super)**

Codes: [Décès_cause selon famille]

No memos

R : Non il ne lui touchait pas.

**P12: Ktw_Hgr_Audit_dcd-08_Proche-12.doc - 12:27 [R : Il mange] (113:113) (Super)**

Codes: [Décès_cause selon famille]

No memos

R : Il mange

**P12: Ktw_Hgr_Audit_dcd-08_Proche-12.doc - 12:26 [R : Il vit sa vie aussi] (109:109) (Super)**

Codes: [Décès_cause selon famille]

No memos

R : Il vit sa vie aussi

**P12: Ktw_Hgr_Audit_dcd-08_Proche-12.doc - 12:25 [R : Non] (106:106) (Super)**

Codes: [Décès_cause selon famille]

No memos

R : Non

**P12: Ktw_Hgr_Audit_dcd-08_Proche-12.doc - 12:24 [R : Il était là sans parler, e..] (102:102) (Super)**

Codes: [Décès_cause selon famille]

No memos

R : Il était là sans parler, et on vit avec lui mais il ne peut même pas dire bonjour ou me demander les nouvelles

**P 5: Bbo_HgrKttmba_Audit_dcd-03_Proche-2.doc - 5:48 [R : Nous on pensait que c’est ..] (182:182) (Super)**

Codes: [Décès_cause selon famille]

No memos

R : Nous on pensait que c’est le fait de manquer le sang parce qu’à tout moment elle était en carence.

**P16: Ktw_HopMtda_Audit_dcd-05_Proche-2.doc - 16:32 [R. La famille a pensé que c’ét..] (138:138) (Super)**

Codes: [Décès_cause selon famille]

No memos

R. La famille a pensé que c’était ces fractures

**P15: Ktw_HopMtda_Audit_dcd-05_Proche-1.doc - 15:49 [R. Rien n’était dit.] (196:196) (Super)**

Codes: [Décès_cause selon famille]

No memos

R. Rien n’était dit.

**P 4: Bbo_HgrKttmba_Audit_dcd-03_Proche-1.doc - 4:36 [R : Nous on avait pensé à sa m..] (148:148) (Super)**

Codes: [Décès_cause selon famille]

No memos

R : Nous on avait pensé à sa maladie habituelle(épilepsie)

**P11: Ktw_Hgr_Audit_dcd-08_Proche-1.doc - 11:51 [R/ Certains avaient dit, comme..] (207:207) (Super)**

Codes: [Décès_cause selon famille]

No memos

R/ Certains avaient dit, comme il était mort étant trop maigre et il avait une grande toux ça doit être du poison ; et mon mari s’était mis ça dans la tête mais il s’était dit encore que qui pouvait donner à un enfant comme celui-là du poison et où il pouvait en trouver lui-même se consola ? deux jours avant sa mort l’enfant commençait à vomir beaucoup qui remplissait un petit sceau du sang en provenance de la bouche et les nez. Il avait docteur(HANGI) de Katwa avait dit que comme les parents de cet enfant pensent qu’il a du poison je vais faire les examens et il les avait faits ensuite il nous avait donné le résultat en disant que ce n’était pas du poison ; alors le docteur nous avait dit que si l’enfant meurt c’est seulement une maladie, la toux mais ce n’est du poison et il est mort après deux jours.

**P15: Ktw_HopMtda_Audit_dcd-05_Proche-1.doc - 15:51 [R. Non, je n’avais pas suivi c..] (204:204) (Super)**

Codes: [Décès_cause selon famille]

No memos

R. Non, je n’avais pas suivi cela

**P 6: Bbo_HgrKttmba_Audit_dcd-03_Proche-3.doc - 6:37 [R : La famille a accepté ce la..] (143:143) (Super)**

Codes: [Décès_cause selon famille]

No memos

R : La famille a accepté ce la cause du décès selon les prestataires

**P19: Ktw_HopMtda_Audit_dcd-10_Proche-1.doc - 19:18 [R : la famille n’avait pas pen..] (81:81) (Super)**

Codes: [Décès_cause selon famille]

No memos

R : la famille n’avait pas pensé à quelque chose comme c’est une maladie qui lui avait attaqué jadis, et on a compris que c’est la mort, peut qu’elle souffrait sans nous informer à temps. S’elle nous aurait informé tôt on pourrait l’amener à l’hôpital à temps.

**P 6: Bbo_HgrKttmba_Audit_dcd-03_Proche-3.doc - 6:34 [R : peut-être par ce que les p..] (135:135) (Super)**

Codes: [Décès_cause selon famille]

No memos

R : peut-être par ce que les prestataires ne disent pas la cause du décès je pense qu’avant sa mort la famille restreinte était déjà informé de sa maladie.

**P17: Ktw_HopMtda_Audit_dcd-05_Proche-3.doc - 17:23 [R. C’était seulement le problè..] (97:97) (Super)**

Codes: [Décès_cause selon famille]

No memos

R. C’était seulement le problème d’écartement des articulations, et puis lorsqu’elle était transférée pour suivre d’autres soins appropriés, elle avait connu la fracture au niveau du bras.

**P 1: Bbo_HgrKttmba_Audit_dcd-01_Proche-1.doc - 1:30 [RÉPONDANT : Ici à hôpital on n..] (101:101) (Super)**

Codes: [Décès_cause selon famille]

No memos

RÉPONDANT : Ici à hôpital on ne nous avait pas dit quelque chose mais lorsqu’il était soigné à horizon c’est là qu’on nous avait dit qu’il souffrait de prostate.

**P 8: Bbo_HgrKttmba_Audit_dcd-04_Proche-2.doc - 8:17 [R : Au niveau de la famille et..] (70:70) (Super)**

Codes: [Décès_cause selon famille]

No memos

R : Au niveau de la famille et même ses enfants savaient que la cause de son décès était ce diabète. Parfois elle était tombée en hypoglycémie ou en hyperglycémie maintenant c’était une grande hypoglycémie.

**P12: Ktw_Hgr_Audit_dcd-08_Proche-12.doc - 12:29 [R : Oui] (121:121) (Super)**

Codes: [Décès_cause selon famille]

No memos

R : Oui

**P10: Ktw_Hgr_Audit_dcd-02_Proche-1.doc - 10:26 [R : Ce que nous savions comme ..] (105:105) (Super)**

Codes: [Décès_cause selon famille]

No memos

R : Ce que nous savions comme problème qui lui faisait souffrir c’était la prostate

**P12: Ktw_Hgr_Audit_dcd-08_Proche-12.doc - 12:30 [R : Elle nous a fait grandir d..] (125:125) (Super)**

Codes: [Décès_cause selon famille]

No memos

R : Elle nous a fait grandir dans la souffrance. Pour notre génération si on avait un mari pareil on ne peut pas atteindre 30ans d’existence. Elle avait enduré beaucoup.

**P 2: Bbo_HgrKttmba_Audit_dcd-01_Proche-2.doc - 2:38 [R : Nous en tant que famille n..] (157:157) (Super)**

Codes: [Décès_cause selon famille]

No memos

R : Nous en tant que famille nous avons compris que le vieux était avancé en âge et venez d’arriver à la fin de ses jours sur la terre sauf qu’il y avait une négligence et un retard pour son traitement de la part des soignants. Par ce que ces derniers avaient fier de soigner à cause de l’Ebola et cela a été la cause du décès de notre grand père.

**P14: Ktw_Hgr_Audit_dcd-09_Proche-1.doc - 14:25 [R : La famille est très furieu..] (101:101) (Super)**

Codes: [Décès_cause selon famille]

No memos

R : La famille est très furieuse de l’hôpital Katwa parce qu’il est décédé par manque d’une bonne charge à la Structure sanitaire.

**P 9: Bbo_HopScola_Audit_dcd-07_Proche-1.doc - 9:41 [R : Peut-être, car la famille ..] (190:190) (Super)**

Codes: [Décès_cause selon famille]

No memos

R : Peut-être, car la famille a fait de son mieux, parce que les médecins disaient qu’ils vont d’abord soigner et ensuite ils donneront la facture après car ils ne donnaient pas des ordonnances médicales.

**P 1: Bbo_HgrKttmba_Audit_dcd-01_Proche-1.doc - 1:28 [RÉPONDANT : Nous en tant que f..] (93:93) (Super)**

Codes: [Décès_cause selon famille]

No memos

RÉPONDANT : Nous en tant que famille, nous avons pensé qu’il est décédé cause de la maladie.

**P12: Ktw_Hgr_Audit_dcd-08_Proche-12.doc - 12:28 [R : Oui c’est moi qui prépare ..] (117:117) (Super)**

Codes: [Décès_cause selon famille]

No memos

R : Oui c’est moi qui prépare la nourriture parfois il peut manger et parfois il laisse c’est son habitude. Lorsqu’il a pris de l’alcool, il arrive et comment à tonner sur nous et à la fin il dit que je ne mange pas votre nourriture et c’est fini.

**P 9: Bbo_HopScola_Audit_dcd-07_Proche-1.doc - 9:33 [R : La famille avait demandé a..] (149:149) (Super)**

Codes: [Décès_cause selon famille]

No memos

R : La famille avait demandé aux médecins et ils avaient dit que vers 2h00, il avait une hypertension qui a conduit au décès

**P13: Ktw_Hgr_Audit_dcd-08_Proche-2.doc - 13:20 [R : On ne sait pas vraiment qu..] (83:83) (Super)**

Codes: [Décès_cause selon famille]

No memos

R : On ne sait pas vraiment quelle a été la cause de son décès, ils ne nous avaient pas dit la cause de sa mort.

**P13: Ktw_Hgr_Audit_dcd-08_Proche-2.doc - 13:21 [R : Personnellement je pense q..] (87:87) (Super)**

Codes: [Décès_cause selon famille]

No memos

R : Personnellement je pense que la cause de son décès était la tuberculose parce que le garçon qui le surveillait, ils étaient toujours ensemble corps à corps. Aussi, l’enfant toussait de façon que sa respiration s’arrêtait avec beaucoup de douleurs et d’amaigrissement. On pouvait voir la colonne vertébral

**P 9: Bbo_HopScola_Audit_dcd-07_Proche-1.doc - 9:34 [R : Aucune autre idée à part c..] (153:153) (Super)**

Codes: [Décès_cause selon famille]

No memos

R : Aucune autre idée à part ce que les soignants avaient dit.

**P 5: Bbo_HgrKttmba_Audit_dcd-03_Proche-2.doc - 5:45 [R : On ne peut pas savoir les ..] (170:170) (Super)**

Codes: [Décès_cause selon prestataires]

No memos

R : On ne peut pas savoir les demander tout cela mais ils nous avaient dit qu’elle n’était pas décédée d’Ebola et on ne l’avait pas mis dans un sac mortuaire.

**P 5: Bbo_HgrKttmba_Audit_dcd-03_Proche-2.doc - 5:46 [R : Elle tombait dans l’anémie..] (174:174) (Super)**

Codes: [Décès_cause selon prestataires]

No memos

R : Elle tombait dans l’anémie chaque fois.

**P11: Ktw_Hgr_Audit_dcd-08_Proche-1.doc - 11:52 [R/ les Soignants avaient dit q..] (211:211) (Super)**

Codes: [Décès_cause selon prestataires]

No memos

R/ les Soignants avaient dit qu’eux n’avaient pas trouvé la maladie, ils ne pouvaient pas dire que c’était la tuberculose ou du poison ou la toux ou autre maladie, ils soignaient seulement pour soigner mais ne sachant pas la maladie de l’enfant ; car si c’était la toux ils avaient déjà donné trop des médicaments pour la toux, mais je savais que c’était seulement la toux qui l’avait tué car lorsqu’il toussait il s’évanouirait même de fois.

**P11: Ktw_Hgr_Audit_dcd-08_Proche-1.doc - 11:53 [R : Oui] (215:215) (Super)**

Codes: [Décès_cause selon prestataires]

No memos

R : Oui

**P11: Ktw_Hgr_Audit_dcd-08_Proche-1.doc - 11:54 [R : Non mais c’est la toux qui..] (219:219) (Super)**

Codes: [Décès_cause selon prestataires]

No memos

R : Non mais c’est la toux qui était à la base par ce qu’il toussait et il pouvait perdre de connaissance et il pouvait faire une heure du temps en train de tousser et il avait les douleurs dans les côtes, comme il n’avait plus la force de tousser il commençait à tousser à l’intérieur.

**P 5: Bbo_HgrKttmba_Audit_dcd-03_Proche-2.doc - 5:47 [R : Elle était tombée dans l’a..] (178:178) (Super)**

Codes: [Décès_cause selon prestataires]

No memos

R : Elle était tombée dans l’anémie et on avait cherché quelqu’un qui pourrait lui aider en lui donnant le sang personne n’était disponible pour qu’on lui prélève et qu’elle soit transfusée.

**P 2: Bbo_HgrKttmba_Audit_dcd-01_Proche-2.doc - 2:29 [R : Ils nous avaient dit que c..] (121:121) (Super)**

Codes: [Décès_cause selon prestataires]

No memos

R : Ils nous avaient dit que c’est cette prostate qui était la cause.

**P10: Ktw_Hgr_Audit_dcd-02_Proche-1.doc - 10:27 [mais Lorsque le malade est déc..] (105:105) (Super)**

Codes: [Décès_cause selon prestataires]

No memos

mais Lorsque le malade est décédé, j’étais curieux de poser la question aux prestataires des soins si nous pouvons retenir quoi comme cause de son décès, ils m’avaient répondu que ces sont les reins et c’est ce que j’avais communiqué au niveau de la famille car nous on pensait que son problème était la prostate. Et celui qui avait dit cela était l’un de soignant permanant du soin intensif il n’était pas un medecin.

**P17: Ktw_HopMtda_Audit_dcd-05_Proche-3.doc - 17:25 [R. Nous, on ne faisait que ple..] (104:104) (Super)**

Codes: [Décès_cause selon prestataires]

No memos

R. Nous, on ne faisait que pleurer le décès de notre cousine ; c’est peut-être la personne qui était partie la facture à qui ils avaient donné cette information.

**P14: Ktw_Hgr_Audit_dcd-09_Proche-1.doc - 14:26 [R : Ils nous avaient dit de re..] (105:105) (Super)**

Codes: [Décès_cause selon prestataires]

No memos

R : Ils nous avaient dit de rentrer à la maison et attendre la volonté Divine.

**P18: Ktw_HopMtda_Audit_dcd-06_Proche-1.doc - 18:51 [R. Ils n’avaient rien dit. Leu..] (201:201) (Super)**

Codes: [Décès_cause selon prestataires]

No memos

R. Ils n’avaient rien dit. Leurs rôles étaient seulement celui de soigner.

**P16: Ktw_HopMtda_Audit_dcd-05_Proche-2.doc - 16:31 [R. Moi je ne sais pas, mais c’..] (134:134) (Super)**

Codes: [Décès_cause selon prestataires]

No memos

R. Moi je ne sais pas, mais c’est le gardien qui connait. De ma part, je peux dire que c’était suite aux douleurs répétitives de la jambe et du bras.

**P 8: Bbo_HgrKttmba_Audit_dcd-04_Proche-2.doc - 8:16 [R : On nous avait dit que c’ét..] (66:66) (Super)**

Codes: [Décès_cause selon prestataires]

No memos

R : On nous avait dit que c’était l’hypotension artérielle du par le diabète.

**P16: Ktw_HopMtda_Audit_dcd-05_Proche-2.doc - 16:30 [R. Je ne sais pas parce que je..] (127:130) (Super)**

Codes: [Décès_cause selon prestataires]

No memos

R. Je ne sais pas parce que je ne l’avais pas rendu visite là. J’avais seulement entendu qu’elle est internée là.

00 :06 :46

00 :07 :05

R. Je ne peux pas connaitre,ceux qui les gardaient qui peuvent conaitre

**P19: Ktw_HopMtda_Audit_dcd-10_Proche-1.doc - 19:17 [R : Ils avaient dit que ça n’a..] (77:77) (Super)**

Codes: [Décès_cause selon prestataires]

No memos

R : Ils avaient dit que ça n’a pas tenu, ils voulaient sauver la vie de la maman parce que l’enfant était déjà mort suite au fibrome était déjà éclaté dans le ventre, c’est pourquoi on l’a opéré mais chose qui n’a pas été possible.

**P 6: Bbo_HgrKttmba_Audit_dcd-03_Proche-3.doc - 6:35 [R : Ils avaient appelé la fami..] (139:139) (Super)**

Codes: [Décès_cause selon prestataires]

No memos

R : Ils avaient appelé la famille restreinte et on leurs avait informé la cause du décès.

**P12: Ktw_Hgr_Audit_dcd-08_Proche-12.doc - 12:20 [R : Ils nous avaient dit que c..] (86:86) (Super)**

Codes: [Décès_cause selon prestataires]

No memos

R : Ils nous avaient dit que c’était une crise cardiaque.

**P 3: Bbo_HgrKttmba_Audit_dcd-01_Proche-3.doc - 3:44 [R : Je n’étais pas là lors de ..] (174:174) (Super)**

Codes: [Décès_cause selon prestataires]

No memos

R : Je n’étais pas là lors de ce résultat mais on savait que c’était la prostate qui été la cause.

**P15: Ktw_HopMtda_Audit_dcd-05_Proche-1.doc - 15:50 [R. Moi, je n’avais pas suivi c..] (200:200) (Super)**

Codes: [Décès_cause selon prestataires]

No memos

R. Moi, je n’avais pas suivi cela.

**P 4: Bbo_HgrKttmba_Audit_dcd-03_Proche-1.doc - 4:35 [R : Ils nous avaient dit que c..] (144:144) (Super)**

Codes: [Décès_cause selon prestataires]

No memos

R : Ils nous avaient dit que ce n’est pas Ebola mais que cette maladie d’épilepsie.

**P 1: Bbo_HgrKttmba_Audit_dcd-01_Proche-1.doc - 1:29 [RÉPONDANT : L’hôpital ne nous ..] (97:97) (Super)**

Codes: [Décès_cause selon prestataires]

No memos

RÉPONDANT : L’hôpital ne nous avait rien dit par rapport à son décès mais nous on connaissait déjà sa maladie il longtemps.

**P13: Ktw_Hgr_Audit_dcd-08_Proche-2.doc - 13:22 [R : Je l’ai déjà dit qu’ils n’..] (91:91) (Super)**

Codes: [Décès_cause selon prestataires]

No memos

R : Je l’ai déjà dit qu’ils n’ont jamais dit la cause, parce qu’ils disaient qu’ils n’ont pas pu diagnostiqué sa maladie qu’il souffrait, jusqu’à maintenant la famille ne sait pas de quoi il est mort.

**P18: Ktw_HopMtda_Audit_dcd-06_Proche-1.doc - 18:52 [R. Ils viennent seulement dire..] (205:205) (Super)**

Codes: [Décès_cause selon prestataires]

No memos

R. Ils viennent seulement dire que la personne est morte

**P18: Ktw_HopMtda_Audit_dcd-06_Proche-1.doc - 18:53 [R. Non ! Je ne sais pas. C’est..] (209:209) (Super)**

Codes: [Décès_cause selon prestataires]

No memos

R. Non ! Je ne sais pas. C’est peut-être ses familiers qui peuvent connaitre, mais de ma part, je sais que c’était la fatigue généralisée ses activités passées et selon sa fréquentation aux différentes structures pour les soins.

**P20: Ktw_HopMtda_Audit_dcd-11_Proche-1.doc - 20:17 [R : Ils avaient dit qu’elle es..] (71:71) (Super)**

Codes: [Décès_cause selon prestataires]

No memos

R : Ils avaient dit qu’elle est décédée et ne nous avait pas dit la cause.

**P16: Ktw_HopMtda_Audit_dcd-05_Proche-2.doc - 16:26 [R. A cause de beaucoup de doul..] (110:110) (Super)**

Codes: [Décès_Circonstances de survenue]

No memos

R. A cause de beaucoup de douleurs et avait eu un problème d’anémie

**P16: Ktw_HopMtda_Audit_dcd-05_Proche-2.doc - 16:27 [R. Oui, puis transférée à Mata..] (114:114) (Super)**

Codes: [Décès_Circonstances de survenue]

No memos

R. Oui, puis transférée à Matanda à cause de cette anémie.

**P12: Ktw_Hgr_Audit_dcd-08_Proche-12.doc - 12:19 [R : Elle décédé dans la circon..] (82:82) (Super)**

Codes: [Décès_Circonstances de survenue]

No memos

R : Elle décédé dans la circonstance sa pathologie gastrique et la tension artérielle.

**P18: Ktw_HopMtda_Audit_dcd-06_Proche-1.doc - 18:49 [R. Tout le monde était surpris..] (193:194) (Super)**

Codes: [Décès_Circonstances de survenue]

No memos

R. Tout le monde était surpris. Entant que voisin, on disait que son corps était déjà fatigué parce qu’elle a fait autant de moments sans tomber malade alors qu’elle travaillait beaucoup (faire le champ, partir à Kasindi pour s’approvisionner les poissons et partir revendre au marché). Nous on se disait que c’est le « NDUHI » en Kinande qui veut dire fatigue.

00 :17 :02

**P12: Ktw_Hgr_Audit_dcd-08_Proche-12.doc - 12:17 [R : Oui au deuxième jour, nous..] (74:74) (Super)**

Codes: [Décès_Circonstances de survenue]

No memos

R : Oui au deuxième jour, nous étions arrivés le lundi vers 15h et on lui plaça une perfusion qui avait la nuit et la journée de mardi on ne lui avait pas donné aucun traitement, ces sont les soignant de la garde qui lui avait visité et à 20h on l’amena aux soins intensifs.

**P13: Ktw_Hgr_Audit_dcd-08_Proche-2.doc - 13:18 [R : Ils nous avaient déjà tran..] (75:75) (Super)**

Codes: [Décès_Circonstances de survenue]

No memos

R : Ils nous avaient déjà transféré au cardiologie à l’UCG le jour où il était mort, parce que les battements de son cœur étaient accélérés, au moment d’y aller, ils avaient alors dit qu’ils n’ont pas d’ambulance. On avait commencé la démarche pour obtenir l’ambulance de CTE et on avait attendu l’ambulance jusqu’au lendemain. Avec ce retard malgré que nous avions les moyens de transport, il fallait une ambulance comme il était sous oxygène, alors on avait attendu l’ambulance qui possédait les matériels de réanimations. Nous avons eu peur de lui amener avec la voiture simple, c’est alors qu’il décéda dans cette nuit.

**P19: Ktw_HopMtda_Audit_dcd-10_Proche-1.doc - 19:2 [R : C’était pendant l’opératio..] (13:13) (Super)**

Codes: [Décès_Circonstances de survenue]

No memos

R : C’était pendant l’opération parce qu’elle n’avait pas quitté la salle d’opération. Nous étions informes que l’enfant était mort et deux heures après on nous dira que la maman était morte aussi pendant qu’elle était en train de subir les traitements.

**P 3: Bbo_HgrKttmba_Audit_dcd-01_Proche-3.doc - 3:58 [R : Oui il ne parlait plus pen..] (234:234) (Super)**

Codes: [Décès_Circonstances de survenue]

No memos

R : Oui il ne parlait plus pendant les trois jours qu’il avait fait au CTE

**P 1: Bbo_HgrKttmba_Audit_dcd-01_Proche-1.doc - 1:26 [RÉPONDANT : Les personnels soi..] (85:85) (Super)**

Codes: [Décès_Circonstances de survenue]

No memos

RÉPONDANT : Les personnels soignants nous avaient dit que la situation c’était complique pour dire qu’il venait de décéder et donc les médicaments n’étaient plus efficaces à sa maladie, par ce que deux jours avant son décès on lui avait amené dans les soins intensifs et lorsque le moteur c’était éteint et il décéda. Ils n’avaient pas dit la cause de son décès.

**P 1: Bbo_HgrKttmba_Audit_dcd-01_Proche-1.doc - 1:27 [RÉPONDANT : Le moteur était là..] (89:89) (Super)**

Codes: [Décès_Circonstances de survenue]

No memos

RÉPONDANT : Le moteur était là pour lui réanimer, si vous l’appelez comment en français je ne sais pas

**P17: Ktw_HopMtda_Audit_dcd-05_Proche-3.doc - 17:22 [R. Elle est morte quand elle n..] (93:93) (Super)**

Codes: [Décès_Circonstances de survenue]

No memos

R. Elle est morte quand elle ne parlait plus. Un jour le Medecin était venu et nous avait dit qu’elle avait le problème respiratoire et l’avait amenée dans les soins intensifs. Un jour après, elle était décédée.

**P13: Ktw_Hgr_Audit_dcd-08_Proche-2.doc - 13:19 [R : Je ne sais pas, mais ils m..] (79:79) (Super)**

Codes: [Décès_Circonstances de survenue]

No memos

R : Je ne sais pas, mais ils m’avaient seulement dit qu’ils n’ont pas d’ambulance, qu’on puisse se débrouiller nous-même.

**P20: Ktw_HopMtda_Audit_dcd-11_Proche-1.doc - 20:16 [R : Oui] (67:67) (Super)**

Codes: [Décès_Circonstances de survenue]

No memos

R : Oui

**P14: Ktw_Hgr_Audit_dcd-09_Proche-1.doc - 14:23 [R : On lui donnait le jus de p..] (93:93) (Super)**

Codes: [Décès_Circonstances de survenue]

No memos

R : On lui donnait le jus de pomme de terre mélangé avec le miel et ça lui avait aidé pendant quatre jours sans douleur abdominale.

**P14: Ktw_Hgr_Audit_dcd-09_Proche-1.doc - 14:22 [R : Il était décédé étant à la..] (89:89) (Super)**

Codes: [Décès_Circonstances de survenue]

No memos

R : Il était décédé étant à la maison, âpres que les médicaments qu’on lui avait donné à l’hôpital soient fini et comme les douleurs abdominales ne finissaient pas on était obligé de chercher un calmant dans la nourriture comme remède.

**P14: Ktw_Hgr_Audit_dcd-09_Proche-1.doc - 14:24 [R : Ceux qui nous avait enseig..] (97:97) (Super)**

Codes: [Décès_Circonstances de survenue]

No memos

R : Ceux qui nous avait enseigné nous avaient dit que c’est un calmant pour le cancer mais ce n’est ne pas un traitement.

**P 9: Bbo_HopScola_Audit_dcd-07_Proche-1.doc - 9:32 [R : Il est décédé la nuit, par..] (144:144) (Super)**

Codes: [Décès_Circonstances de survenue]

No memos

R : Il est décédé la nuit, parce que chaque jour on était là, on se parlait et ce jour-là c’était le même rythme, c’est ainsi qu’on nous avait informé le matin du lendemain qu’il était déjà décédé.

**P11: Ktw_Hgr_Audit_dcd-08_Proche-1.doc - 11:50 [R/Il avait déjà trop maigri de..] (203:203) (Super)**

Codes: [Décès_Circonstances de survenue]

No memos

R/Il avait déjà trop maigri de manière exagéré en lui voyant, vous pouviez avoir la compassion envers lui.

**P 3: Bbo_HgrKttmba_Audit_dcd-01_Proche-3.doc - 3:59 [R : Il avait quitté la maison ..] (238:238) (Super)**

Codes: [Décès_Circonstances de survenue]

No memos

R : Il avait quitté la maison dans un état grave « Ametoha »

**P17: Ktw_HopMtda_Audit_dcd-05_Proche-3.doc - 17:8 [R. Il y avait un problème d’éc..] (33:33) (Super)**

Codes: [Décès_Début-Histoire de la maladie]

No memos

R. Il y avait un problème d’écartement des articulations au niveau de la jambe. Cela avait occasionné un problème de déplacement, ce qui avait fait à ce qu’on lui achète des béquilles. Au lieu de l’entreprendre comment marcher avec les béquilles, elle était délaissée, ce qui avait fait à ce qu’elle tombe par terre qui avait occasionné une fracture au niveau du bras. Il fallait la transférer à Matanda où on avait découvert qu’elle n’était pas bien bien prise en charge. Il fallait au niveau de Matanda, attendre comment elle va rétablir un peu pour enlever le plâtre, ce qui était fait par les soignants.

**P 2: Bbo_HgrKttmba_Audit_dcd-01_Proche-2.doc - 2:8 [R : Même une année.] (37:37) (Super)**

Codes: [Décès_Début-Histoire de la maladie]

No memos

R : Même une année.

**P 2: Bbo_HgrKttmba_Audit_dcd-01_Proche-2.doc - 2:7 [R : Il venait de souffrir long..] (33:33) (Super)**

Codes: [Décès_Début-Histoire de la maladie]

No memos

R : Il venait de souffrir longtemps.

**P17: Ktw_HopMtda_Audit_dcd-05_Proche-3.doc - 17:9 [R. Elle était portée au dos pa..] (37:37) (Super)**

Codes: [Décès_Début-Histoire de la maladie]

No memos

R. Elle était portée au dos parce qu’elle avait plus la possibilité de marcher.

**P18: Ktw_HopMtda_Audit_dcd-06_Proche-1.doc - 18:47 [R. Non, elle était déjà sortie..] (185:185) (Super)**

Codes: [Décès_Début-Histoire de la maladie]

No memos

R. Non, elle était déjà sortie de l’hôpital et arrivée chez elle. Mais en y arrivant, la santé était devenue médiocre avec une paralysie simple

**P 8: Bbo_HgrKttmba_Audit_dcd-04_Proche-2.doc - 8:7 [R : Oui c’est l’unique maladie..] (30:30) (Super)**

Codes: [Décès_Début-Histoire de la maladie]

No memos

R : Oui c’est l’unique maladie qui avait fait que nous l’amenions à l’hôpital et elle venait de faire longtemps avec ça ; c’était la fin.

**P16: Ktw_HopMtda_Audit_dcd-05_Proche-2.doc - 16:23 [R. Oui] (100:100) (Super)**

Codes: [Décès_Début-Histoire de la maladie]

No memos

R. Oui

**P18: Ktw_HopMtda_Audit_dcd-06_Proche-1.doc - 18:46 [R. Là vraiment, elle ne pouvai..] (181:181) (Super)**

Codes: [Décès_Début-Histoire de la maladie]

No memos

R. Là vraiment, elle ne pouvait pas

**P 8: Bbo_HgrKttmba_Audit_dcd-04_Proche-2.doc - 8:6 [R : Le début de sa maladie ava..] (26:26) (Super)**

Codes: [Décès_Début-Histoire de la maladie]

No memos

R : Le début de sa maladie avait commencé par le diabète qui a duré sept ans

**P10: Ktw_Hgr_Audit_dcd-02_Proche-1.doc - 10:7 [R : Depuis le début jusqu’à l’..] (29:29) (Super)**

Codes: [Décès_Début-Histoire de la maladie]

No memos

R : Depuis le début jusqu’à l’hôpital ça faisait sept mois mais dans cette période il pouvait faire le mouvement seulement que nous lui avons interdit de faire quelque chose compte tenu de son état. Nous pouvons signaler que les deux dernières semaines que son état était affaibli dont une semaine à la maison et une à l’hôpital. Les restes de ces sept mois il se déplaçait sans problème. Même ceux qui ont appris son décès s’étonnaient car ils lui voyaient à la maison.

**P10: Ktw_Hgr_Audit_dcd-02_Proche-1.doc - 10:8 [R : Pendant ces deux semaines ..] (33:33) (Super)**

Codes: [Décès_Début-Histoire de la maladie]

No memos

R : Pendant ces deux semaines il avait un problème d’aller au toilette et même d’uriner, ce qui avait fait qui nous nous démenions pour lui chercher le médicament de deux coté. Il suivait le traitement moderne et le traitement traditionnel. Souvent les gens disent que les médicaments modernes ne soignent pas le poison, ceux qui fabriquent le médicament traditionnel ce sont eux qui soigne le poison. Il y a même les appareils qui ne détectent pas le poison au niveau de l’hôpital moderne, ce qui nous avait pousser de poursuivre le deux traitement. Pendant les deux semaines le fait de ne pas faire ses besoins son état de santé s’était dégradé.et lorsqu’on lui avait donné le traitement et reprendre ses besoins on passait que le médicament va lui soigner et nous avons continué le médicament lui étant à une seule place. Il apparaissait comme quelqu’un qui sent bien et d’autre fois en état de gravité, ce qui nous avait poussé de l’amener dans une grande Fosa Katwa

**P 6: Bbo_HgrKttmba_Audit_dcd-03_Proche-3.doc - 6:12 [R : Je ne connais pas par ce q..] (45:45) (Super)**

Codes: [Décès_Début-Histoire de la maladie]

No memos

R : Je ne connais pas par ce qu’elle se rendait au centre de santé pour y prendre le traitement et cela pour six mois.

**P18: Ktw_HopMtda_Audit_dcd-06_Proche-1.doc - 18:15 [R. Elle disait qu’elle sentait..] (60:60) (Super)**

Codes: [Décès_Début-Histoire de la maladie]

No memos

R. Elle disait qu’elle sentait des douleurs suite aux problèmes liés aux muscles avec asthénie physique

**P18: Ktw_HopMtda_Audit_dcd-06_Proche-1.doc - 18:10 [R. Sa santé était bonne quand ..] (37:37) (Super)**

Codes: [Décès_Début-Histoire de la maladie]

No memos

R. Sa santé était bonne quand elle faisait les travaux champêtres. Ce n’est qu’après elle avait commencé à sentir mal au niveau des muscles, ce qui ne la permettait plus de faire cette activité. Elle achetait des médicaments dans une pharmacie qu’elle consommait chez elle. Mais cela ne promettait pas. On avait décidé de l’amener dans une structure sanitaire qu’on appelle Kalungamakuha vers Kimemik. Là où on soigne les gens qui ont connus des accidents (fractures), qui ont de problème des nerfs et des muscles. C’est là où on l’amena pour les soins appropriés. Plus tard, j’ai compris qu’elle était déjà paralysée. Il parait que la douleur des muscles qu’elle avait, a fait qu’il y ait séparation des os de la colonne vertébrale. Elle a suivi les soins dans cette structure jusqu’à ce qu’elle était guérie et sortie pour son domicile. En arrivant à la maison, elle avait encore rechuté et on amena à l’Horizon (structure sanitaire) où elle était suivie et guérie puis retourna chez elle. Après deux mois à la maison, elle avait encore rechuté et admise à l’hôpital de Kyondo où elle était suivie pendant un moment donné, elle fut déchargée ramenée à son domicile où la famille n’a pas su la supporter. Il fallait encore qu’on l’amène à Matanda où elle a succombé.

**P 5: Bbo_HgrKttmba_Audit_dcd-03_Proche-2.doc - 5:26 [R : Non s’est juste après la s..] (103:103) (Super)**

Codes: [Décès_Début-Histoire de la maladie]

No memos

R : Non s’est juste après la séparation qu’elle avait commencée à tomber malade.

**P 6: Bbo_HgrKttmba_Audit_dcd-03_Proche-3.doc - 6:13 [R : Cette maladie a duré envir..] (49:49) (Super)**

Codes: [Décès_Début-Histoire de la maladie]

No memos

R : Cette maladie a duré environs trois mois, parfois elle allait au centre de santé et elle se sentait mieux de fois elle rechutait et y retourne et c’est de là qu’elle était transférée.

**P 7: Bbo_HgrKttmba_Audit_dcd-04_Proche-1.doc - 7:19 [R : Nous savons que les diabét..] (78:78) (Super)**

Codes: [Décès_Début-Histoire de la maladie]

No memos

R : Nous savons que les diabétiques se rendent toujours à l’hôpital pour y recevoir le traitement.

**P 7: Bbo_HgrKttmba_Audit_dcd-04_Proche-1.doc - 7:18 [R : Non je ne sais pas le comm..] (74:74) (Super)**

Codes: [Décès_Début-Histoire de la maladie]

No memos

R : Non je ne sais pas le commencement de la maladie.

**P11: Ktw_Hgr_Audit_dcd-08_Proche-1.doc - 11:7 [R : C’est la toux.] (29:29) (Super)**

Codes: [Décès_Début-Histoire de la maladie]

No memos

R : C’est la toux.

**P 2: Bbo_HgrKttmba_Audit_dcd-01_Proche-2.doc - 2:6 [R : Comme il était avancé en â..] (29:29) (Super)**

Codes: [Décès_Début-Histoire de la maladie]

No memos

R : Comme il était avancé en âge, il n’avait plus la force et c’est ainsi que la maladie lui a affaibli et c’est ainsi que nous lui avons amené à l’hôpital où les résultats ont montré qu’il souffrait de la prostate.

**P 7: Bbo_HgrKttmba_Audit_dcd-04_Proche-1.doc - 7:20 [R : Il y avait un changement.] (82:82) (Super)**

Codes: [Décès_Début-Histoire de la maladie]

No memos

R : Il y avait un changement.

**P 5: Bbo_HgrKttmba_Audit_dcd-03_Proche-2.doc - 5:25 [R : ça date de longtemps, c’es..] (99:99) (Super)**

Codes: [Décès_Début-Histoire de la maladie]

No memos

R : ça date de longtemps, c’est depuis qu’elle s’est séparée avec son mari que la maladie avait commencé. Mais lorsqu’ils vivaient ensemble elle n’avait jamais tombé malade

**P 9: Bbo_HopScola_Audit_dcd-07_Proche-1.doc - 9:12 [R : ça commencé par la fatigue..] (52:55) (Super)**

Codes: [Décès_Début-Histoire de la maladie]

No memos

R : ça commencé par la fatigue, il nous disait qu’il était vraiment fatigué, et ensuite la fièvre, c’est ainsi qu’on l’amena à l’hôpital. Et lui-même nous avait dit de n’est pas l’amener au centre de santé mais uniquement à l’hôpital.

On l’amena au Centre Hospitalier tout juste à côté de chez lui, chez maman Scola.

Il avait cru comme il toussait et sentait la fatigue, il croyait qu’il était empoisonné. Et comme cette maman soignait aussi le poison, elle nous avait dit que ce n’était pas du poison mais trop de fatigue mais aussi vu qu’il faisait une longue distance, et l’endroit où il cultiver c’était une région chaude, ce changement climatique a été à la base de sa maladie.

A l’hôpital, il s’exprimait bien et on ne savait pas qu’il va mourir

**P 9: Bbo_HopScola_Audit_dcd-07_Proche-1.doc - 9:11 [R : Il ne souffrait pas, sauf ..] (47:47) (Super)**

Codes: [Décès_Début-Histoire de la maladie]

No memos

R : Il ne souffrait pas, sauf quelque fois il avait la fièvre ou les maux de tête, il achetait des médicaments à la pharmacie et tout allait bien.

**P16: Ktw_HopMtda_Audit_dcd-05_Proche-2.doc - 16:21 [R. C’est lorsqu’elle était tom..] (92:92) (Super)**

Codes: [Décès_Début-Histoire de la maladie]

No memos

R. C’est lorsqu’elle était tombée sur le bras, qu’elle avait commencé à souffrir davantage.

**P18: Ktw_HopMtda_Audit_dcd-06_Proche-1.doc - 18:48 [R. Non, il fallait qu’elle ait..] (189:189) (Super)**

Codes: [Décès_Début-Histoire de la maladie]

No memos

R. Non, il fallait qu’elle ait un soutien , une personne sur laquelle elle va s’appuyer. Les familiers ont fini par décider qu’il ne fallait pas la laisser à la maison dans cet état et décidèrent de l’amener à Matanda, une structure médicale où elle finira par y décéder ?

**P 3: Bbo_HgrKttmba_Audit_dcd-01_Proche-3.doc - 3:13 [R : La maladie vient de durer ..] (50:50) (Super)**

Codes: [Décès_Début-Histoire de la maladie]

No memos

R : La maladie vient de durer peu de temps mais à l’horizon il était soigné en ambulatoire.

**P18: Ktw_HopMtda_Audit_dcd-06_Proche-1.doc - 18:50 [R. Oui, c’est ce que nous on s..] (197:197) (Super)**

Codes: [Décès_Début-Histoire de la maladie]

No memos

R. Oui, c’est ce que nous on se disait dans le voisinage parce que celle-là peut-être à la base du problème des muscles et aussi de la malaria.

**P16: Ktw_HopMtda_Audit_dcd-05_Proche-2.doc - 16:16 [R. Je ne savais pas ; J’avais ..] (65:65) (Super)**

Codes: [Décès_Début-Histoire de la maladie]

No memos

R. Je ne savais pas ; J’avais seulement compris qu’elle est amenée là puis qu’on avait mis sous plâtre son pied

**P19: Ktw_HopMtda_Audit_dcd-10_Proche-1.doc - 19:14 [R : Non, elle était opéré le m..] (65:65) (Super)**

Codes: [Décès_Début-Histoire de la maladie]

No memos

R : Non, elle était opéré le même jour pour Mort in utero. Ça faisait déjà deux jours qu’elle sentait des douleurs abdominales intenses.

**P19: Ktw_HopMtda_Audit_dcd-10_Proche-1.doc - 19:13 [R : Oui, sa grossesse évolua j..] (61:61) (Super)**

Codes: [Décès_Début-Histoire de la maladie]

No memos

R : Oui, sa grossesse évolua jusqu’à atteindre si mois. C’est alors qu’on amena à Matanda. Et c’est ce moment qu’elle avait subi une intervention chirurgicale.

**P14: Ktw_Hgr_Audit_dcd-09_Proche-1.doc - 14:5 [R : Il avait quitté à Bunia sa..] (21:21) (Super)**

Codes: [Décès_Début-Histoire de la maladie]

No memos

R : Il avait quitté à Bunia sa femme était enceinte, il était venu attendre l’accouchement de sa femme et le nouveau-né décéda après trois semaine. C’est ainsi qu’il resta chez lui, ce n’est pas à dire qu’il était venu avec la maladie, la maladie avait commencé lorsqu’il était déjà de retour et il se plaignait du ventre et la tête, on nous dira que c’était le paludisme et la gastrite à Katwa. Deux semaines après il retourna à Katwa pour le contrôle les médecins lui diront qu’il n’a plus le paludisme mais le malaise continua.

**P 9: Bbo_HopScola_Audit_dcd-07_Proche-1.doc - 9:35 [R : Son séjour dans la structu..] (158:158) (Super)**

Codes: [Décès_Début-Histoire de la maladie]

No memos

R : Son séjour dans la structure pour la prise en charge était de trente jours.

**P 4: Bbo_HgrKttmba_Audit_dcd-03_Proche-1.doc - 4:12 [R : Comme je l’ai dit elle sou..] (53:53) (Super)**

Codes: [Décès_Début-Histoire de la maladie]

No memos

R : Comme je l’ai dit elle souffrait de la tête et c’est à ce moment-là qu’on l’avait amené à KABIKENE dans un hôpital en place

**P 1: Bbo_HgrKttmba_Audit_dcd-01_Proche-1.doc - 1:7 [RÉPONDANT : C’était un malade ..] (23:23) (Super)**

Codes: [Décès_Début-Histoire de la maladie]

No memos

RÉPONDANT : C’était un malade de longue durée. Il a fait au moins deux ans avec sa maladie et il souffrait de prostate. Maintenant les prestataires qui lui soignaient étaient à l’horizon.

**P16: Ktw_HopMtda_Audit_dcd-05_Proche-2.doc - 16:22 [R. On croyait que l’os avait c..] (96:96) (Super)**

Codes: [Décès_Début-Histoire de la maladie]

No memos

R. On croyait que l’os avait connu une fracture, il fallait y aller pour la kinésithérapie

**P 9: Bbo_HopScola_Audit_dcd-07_Proche-1.doc - 9:16 [R : Premièrement fois c’était ..] (73:73) (Super)**

Codes: [Décès_Début-Histoire de la maladie]

No memos

R : Premièrement fois c’était la fatigue, ensuite la fièvre et enfin la céphalée.

**P12: Ktw_Hgr_Audit_dcd-08_Proche-12.doc - 12:7 [R : J’allais dans la forêt.] (30:30) (Super)**

Codes: [Décès_Début-Histoire de la maladie]

No memos

R : J’allais dans la forêt.

**P12: Ktw_Hgr_Audit_dcd-08_Proche-12.doc - 12:6 [R : Au début, elle avait comme..] (26:26) (Super)**

Codes: [Décès_Début-Histoire de la maladie]

No memos

R : Au début, elle avait commencé à avoir du mal à respirer quand elle montait sur la montagne, elle commençait à respirer plus vite. S’elle ne se repose pas elle ne peut plus continuer à escalader la montagne. C’est ainsi qu’on amena à l’hôpital pour faire des examens au C H Mutiri. C’est ainsi que les soignants diront que ses veines étaient bouchées. C’est ainsi que les soignant lui donnèrent le traitement, elle finit le traitement sans être soulagé, après un temps on l’amena encore à l’hôpital de Vungi, parce qu’elle n’y avait pas de changement malgré le traitement reçu. Je n’étais pas là lorsqu’on l’avait amené à Vungi. Et là aussi, elle avait reçu le traitement mais il n’y avait pas eu de changement également. C’est à ce moment que je suis revenue et la trouver là.

**P 1: Bbo_HgrKttmba_Audit_dcd-01_Proche-1.doc - 1:6 [RÉPONDANT : C’est à Graben] (25:25) (Super)**

Codes: [Décès_Début-Histoire de la maladie] [Décès_Lieu de survenu]

No memos

RÉPONDANT : C’est à Graben

**P10: Ktw_Hgr_Audit_dcd-02_Proche-1.doc - 10:6 [R : Dans sa vie il avait la fo..] (25:25) (Super)**

Codes: [Décès_Début-Histoire de la maladie] [Diagnostic-Antécédants_Médicaux]

No memos

R : Dans sa vie il avait la force, il n’avait pas aucun problème de santé, par ce que dans sa vie depuis sa jeunnesse il vendait le poisson et il transportait sur la tête en provenance de Kyavinyonge vers Bembo c’est après qu’il avait commencé à transporter sur le vélo plus tard il avait commencé la route de Kisangani pour le commerce ; ainsi il arrêta et repris ses activités champêtres et donc il n’avait pas aucun problème de santé. Sauf qu’aux sept dernier mois son corps était fatigué compte tenu de l’âge et aussi durant son temps il n’avait pas eu un repos. Après un moment d’observation nous avons constaté qu’il était empoisonné, par ce que nous lui avons amené à l’hôpital mais ce dernier n’avait trouvé aucune maladie. C’est après que nous avions cherché un soignant qui traite avec la médecine moderne et la médecine traditionnelle. Après lui avoir examiné c’est à ce moment-là qu’il nous avait dit, qu’il était empoisonné. Il commençant le traitement et il y avait une amélioration. C’est un peu après qu’il constata qu’il avait un problème de prostate, et la respiration. Pour le poison c’était la guérison après le contrôle mais pour la prostate c’était impossible qui nous poussa à chercher un autre soignant qui à son tour échoua. Nous avons cherché un troisième soignant qui lui avait donné le traitement et il commença à uriner et comme on voulait lui amener à la structure sanitaire nous avons arrêté de lui amener à cette dernière. Une semaine de traitement nous avons remarqué qu’il ne faisait plus de mouvement et c’est à ce moment-là que nous l’avions amené à l’hôpital KATWA. En arrivant à l’hôpital Katwa on lui avait traiter et il avait fait les différents examens. Pendant les examens j’avais la curiosité de comprendre les résultats mais j’avais constaté qu’ils avaient remarqué quelque chose qu’ils n’ont pas dit. Deux ou trois jours après j’ai demandé à un infirmier les résultats des examens. Il m’avait répondu qu’il y a un examen qui n’est pas bien sortie mais il ne m’avait pas dit c’est quel examen. Après que le malade décède, j’avais reposé la question de savoir ce que nous pouvons retenir comme cause du décès ; l’un des infirmiers me dira que c’était un problème des reins que ce n’est pas un problème de Prostate. A l’arrivé on nous avait dit qu’il souffrait de l’asthme et une semaine avant d’être reçus à l’hôpital sa respiration n’était pas bonne ; on entendait un petit sifflet lors de sa respiration. C’est pourquoi nous avons conclus à deux éléments la prostate et l’asthme qui sont à la base de sa mort plus le troisième qui est les reins que les soignants ont découvert.

**P 2: Bbo_HgrKttmba_Audit_dcd-01_Proche-2.doc - 2:4 [R : J’ai connu sa maladie aprè..] (21:21) (Super)**

Codes: [Décès_Début-Histoire de la maladie] [Diagnostic-Antécédants_Médicaux]

No memos

R : J’ai connu sa maladie après qu’il soit gravement malade mais on nous disait qu’il souffrait de la prostate.

**P11: Ktw_Hgr_Audit_dcd-08_Proche-1.doc - 11:8 [R : Il avait commencé à tousse..] (33:33) (Super)**

Codes: [Décès_Début-Histoire de la maladie] [Fréquentation_Autre_Fosa]

No memos

R : Il avait commencé à tousser, une toux exagérée, om l’avait amené ici CS où on l’a suivi pendant trois mois la toux pas de soulagement, puis ils nous ont transféré à CANADIENS vers Makasi

**P16: Ktw_HopMtda_Audit_dcd-05_Proche-2.doc - 16:11 [R. Elle souffrait des muscles ..] (45:45) (Super)**

Codes: [Décès_Début-Histoire de la maladie] [Fréquentation_Autre_Fosa]

No memos

R. Elle souffrait des muscles et était partie aux soins à KITATUMBA puis transférée à Kalungamakuha (Centre pour handicapé)

**P 6: Bbo_HgrKttmba_Audit_dcd-03_Proche-3.doc - 6:21 [R : ça on n’avait pas demandé ..] (82:82) (Super)**

Codes: [Diagnostic-Antécédants_Médicaux]

No memos

R : ça on n’avait pas demandé aux soignants.

**P 2: Bbo_HgrKttmba_Audit_dcd-01_Proche-2.doc - 2:5 [R : On nous l’avait dit à l’hô..] (25:25) (Super)**

Codes: [Diagnostic-Antécédants_Médicaux]

No memos

R : On nous l’avait dit à l’hôpital Kitatumba

**P 6: Bbo_HgrKttmba_Audit_dcd-03_Proche-3.doc - 6:19 [R : On ne nous avait pas dit s..] (74:74) (Super)**

Codes: [Diagnostic-Antécédants_Médicaux]

No memos

R : On ne nous avait pas dit ses pathologies, mais on avait constaté que ce centre de santé traite le malade lorsque le malade tient encore ; on nous avait dit qu’on lui transfère parce que la santé ne s’améliore pas.

**P 3: Bbo_HgrKttmba_Audit_dcd-01_Proche-3.doc - 3:6 [R : Oui, et puis la maladie av..] (22:22) (Super)**

Codes: [Diagnostic-Antécédants_Médicaux]

No memos

R : Oui, et puis la maladie avait continué et on lui avait amené à Kitatumba où on lui avait transféré au CTE pendant trois jours sans traitement et la maladie s’aggrava, on lui retourna à Kitatumba trois jours après il décéda

**P 9: Bbo_HopScola_Audit_dcd-07_Proche-1.doc - 9:24 [R : Il m’avait dit qu’on lui s..] (108:108) (Super)**

Codes: [Diagnostic-Antécédants_Médicaux]

No memos

R : Il m’avait dit qu’on lui soignait le paludisme mais aussi qu’il était trop fatigué.

**P 7: Bbo_HgrKttmba_Audit_dcd-04_Proche-1.doc - 7:16 [R : Je savais qu’elle était so..] (66:66) (Super)**

Codes: [Diagnostic-Antécédants_Médicaux]

No memos

R : Je savais qu’elle était souffrante du diabète

**P 4: Bbo_HgrKttmba_Audit_dcd-03_Proche-1.doc - 4:6 [R : Oui c’était toujours sa ma..] (29:29) (Super)**

Codes: [Diagnostic-Antécédants_Médicaux]

No memos

R : Oui c’était toujours sa maladie

**P 7: Bbo_HgrKttmba_Audit_dcd-04_Proche-1.doc - 7:17 [R : Oui c’était la cause de so..] (70:70) (Super)**

Codes: [Diagnostic-Antécédants_Médicaux]

No memos

R : Oui c’était la cause de son hospitalisation

**P 4: Bbo_HgrKttmba_Audit_dcd-03_Proche-1.doc - 4:5 [R : Elle souffrait de la tête] (25:25) (Super)**

Codes: [Diagnostic-Antécédants_Médicaux]

No memos

R : Elle souffrait de la tête

**P 2: Bbo_HgrKttmba_Audit_dcd-01_Proche-2.doc - 2:21 [R : A ma connaissance c’est la..] (89:89) (Super)**

Codes: [Diagnostic-Antécédants_Médicaux]

No memos

R : A ma connaissance c’est la prostate seulement.

**P11: Ktw_Hgr_Audit_dcd-08_Proche-1.doc - 11:18 [R/ Il souffrait des muscles de..] (73:73) (Super)**

Codes: [Diagnostic-Antécédants_Médicaux]

No memos

R/ Il souffrait des muscles de la jambe, de fois la jambe gonflé mais ce n’est pas la jambe qui l’a tuée

**P20: Ktw_HopMtda_Audit_dcd-11_Proche-1.doc - 20:1 [R : Elle avait connu une paral..] (11:11) (Super)**

Codes: [Diagnostic-Antécédants_Médicaux]

No memos

R : Elle avait connu une paralysie de membre inferieurs

**P11: Ktw_Hgr_Audit_dcd-08_Proche-1.doc - 11:19 [R/ On l’amener toujours ici au..] (77:77) (Super)**

Codes: [Diagnostic-Antécédants_Médicaux]

No memos

R/ On l’amener toujours ici au centre de santé pour les soins et ça lui soulageait

**P15: Ktw_HopMtda_Audit_dcd-05_Proche-1.doc - 15:11 [R. C’était le problème du musc..] (46:46) (Super)**

Codes: [Diagnostic-Antécédants_Médicaux]

No memos

R. C’était le problème du muscle au niveau de la jambe ;

**P15: Ktw_HopMtda_Audit_dcd-05_Proche-1.doc - 15:12 [R. C’est sur la partie inférie..] (50:51) (Super)**

Codes: [Diagnostic-Antécédants_Médicaux]

No memos

R. C’est sur la partie inférieure, c’est ainsi qu’on l’a amené à l’hôpital de KITATUMBA.

00 :02 :54

**P 6: Bbo_HgrKttmba_Audit_dcd-03_Proche-3.doc - 6:11 [R : Sa santé n’était pas du to..] (41:41) (Super)**

Codes: [Diagnostic-Antécédants_Médicaux]

No memos

R : Sa santé n’était pas du tout forte, souvent elle avait de malaise, tousser et elle est partie faire les examens qui donnera le résultat comme quoi c’était la tuberculose. On lui donna le traitement et elle guéri.

**P13: Ktw_Hgr_Audit_dcd-08_Proche-2.doc - 13:23 [R : Depuis son arrivée dans ce..] (95:95) (Super)**

Codes: [Diagnostic-Antécédants_Médicaux]

No memos

R : Depuis son arrivée dans ce milieu, il n’avait jamais souffert d’autres maladie à part cette toux.

**P15: Ktw_HopMtda_Audit_dcd-05_Proche-1.doc - 15:48 [R. Si, mais le problème d’’ané..] (192:192) (Super)**

Codes: [Diagnostic-Antécédants_Médicaux]

No memos

R. Si, mais le problème d’’anémie ne se terminait pas. Il y avait vraiment des gestes répétitifs des transfusions sanguines mais qui n’aboutissaient pas au bon résultat.

**P 8: Bbo_HgrKttmba_Audit_dcd-04_Proche-2.doc - 8:18 [R : Elle souffrait de Myosite ..] (74:74) (Super)**

Codes: [Diagnostic-Antécédants_Médicaux]

No memos

R : Elle souffrait de Myosite pendant vingt ans et c’a été commencé il y a longtemps à peu près 20ans et on était partis avec elle à l’hôpital de Kyondo quand j’étais en 3eme primaire et maintenant j’ai cinq naissances.

**P15: Ktw_HopMtda_Audit_dcd-05_Proche-1.doc - 15:14 [R. Non, elle n’avait jamais to..] (57:57) (Super)**

Codes: [Diagnostic-Antécédants_Médicaux]

No memos

R. Non, elle n’avait jamais tombé malade

**P 1: Bbo_HgrKttmba_Audit_dcd-01_Proche-1.doc - 1:10 [Interviewer : Vous m’avez dit ..] (33:33) (Super)**

Codes: [Diagnostic-Antécédants_Médicaux]

No memos

**Interviewer** : Vous m’avez dit qu’il souffrait de la prostate, comment avez-vous su que réellement c’était cette maladie ?

**P 4: Bbo_HgrKttmba_Audit_dcd-03_Proche-1.doc - 4:27 [R : Non à tout moment elle ne ..] (113:113) (Super)**

Codes: [Diagnostic-Antécédants_Médicaux]

No memos

R : Non à tout moment elle ne nous disait que c’est la tête qui lui dérangeait.

**P 3: Bbo_HgrKttmba_Audit_dcd-01_Proche-3.doc - 3:22 [00 :04 :58 R : Non il n’y avai..] (85:86) (Super)**

Codes: [Diagnostic-Antécédants_Médicaux]

No memos

00 :04 :58

R : Non il n’y avait pas d’autres maladies.

**P 8: Bbo_HgrKttmba_Audit_dcd-04_Proche-2.doc - 8:19 [R : Apres l’avoir traité à l’h..] (78:78) (Super)**

Codes: [Diagnostic-Antécédants_Médicaux]

No memos

R : Apres l’avoir traité à l’hôpital de Kyondo elle n’avait plus de douleur et comme on habitait très loin à Kyanzaba on n’était plus partis au contrôle mais ça ne dérangeait plus.

**P12: Ktw_Hgr_Audit_dcd-08_Proche-12.doc - 12:31 [R : Oui mais elle était en for..] (129:130) (Super)**

Codes: [Diagnostic-Antécédants_Médicaux]

No memos

R : Oui mais elle était en forme, elle allait cultiver à Buhinga et lorsqu’elle y restait sa santé était bonne par ce qu’elle grossissait, on se disait Maman est encore jeune. Lorsqu’elle rentre ici un mois seulement elle maigrit encore et un jour elle dit que moi je peux vivre dans la foret (le lieu où elle cultive). Son dernier fils lui avait acheté une parcelle à Buhinga soit disant qu’elle va chaque fois cultiver en y résidant. C’était un grand problème jusqu’à ce que le papa commençait à tonner sur nous en disant « s’il pourra coucher avec nous » par ce que nous sommes allés garder sa femme là au champs. On était obligé de dire à maman qu’elle avait accepté de vivre avec son mari, on ne va pas porter votre croix. Elle rentra et voilà son sort. Mais lorsqu’elle était à Buhinga elle ne pouvait pas dire qu’elle souffre de l’estomac, de tension et de quoi que ce soit. Elle mangeait, cultivait bien sans difficulté.

Nous on avait compris que le problème était notre père qui lui faisait souffrir par ce qu’il pouvait venir étant ivre, quand on veut manger et pourtant qu’il n’a pas donné même cent franc pour l’acheter, il part jeter ça à l’extérieur.

**P 5: Bbo_HgrKttmba_Audit_dcd-03_Proche-2.doc - 5:10 [R : Trois à quatre fois par jo..] (39:39) (Super)**

Codes: [Diagnostic-Antécédants_Médicaux]

No memos

R : Trois à quatre fois par jour. Dit-on que pendant la nuit c’est plus grave

**P 7: Bbo_HgrKttmba_Audit_dcd-04_Proche-1.doc - 7:9 [R : Oui elle était venue chez ..] (38:38) (Super)**

Codes: [Diagnostic-Antécédants_Médicaux]

No memos

R : Oui elle était venue chez sa fille pendant son traitement.

**P15: Ktw_HopMtda_Audit_dcd-05_Proche-1.doc - 15:47 [R. C’est seulement cette malad..] (188:188) (Super)**

Codes: [Diagnostic-Antécédants_Médicaux]

No memos

R. C’est seulement cette maladie d’anémie. Lorsqu’il y a diminution du sang (anémie), c’est la mort parce qu’il constitue l’essence de l’organisme humain.

**P18: Ktw_HopMtda_Audit_dcd-06_Proche-1.doc - 18:40 [R. C’était question des douleu..] (158:158) (Super)**

Codes: [Diagnostic-Antécédants_Médicaux]

No memos

R. C’était question des douleurs au niveau de tout l’organisme pour lequel elle sera admise à Horizon.

**P18: Ktw_HopMtda_Audit_dcd-06_Proche-1.doc - 18:39 [R. Elle disait qu’elle avait l..] (154:154) (Super)**

Codes: [Diagnostic-Antécédants_Médicaux]

No memos

R. Elle disait qu’elle avait les malaises du corps.

**P 7: Bbo_HgrKttmba_Audit_dcd-04_Proche-1.doc - 7:5 [R : Oui elle recevait le trait..] (22:22) (Super)**

Codes: [Diagnostic-Antécédants_Médicaux]

No memos

R : Oui elle recevait le traitement

**P17: Ktw_HopMtda_Audit_dcd-05_Proche-3.doc - 17:24 [R. Non] (101:101) (Super)**

Codes: [Diagnostic-Antécédants_Médicaux]

No memos

R. Non

**P 1: Bbo_HgrKttmba_Audit_dcd-01_Proche-1.doc - 1:9 [RÉPONDANT : Quand il se faisai..] (35:35) (Super)**

Codes: [Diagnostic-Antécédants_Médicaux]

No memos

RÉPONDANT : Quand il se faisait soigner à l’horizon c’est de là que les prestataires nous avaient dit que c’était la prostate

**P 4: Bbo_HgrKttmba_Audit_dcd-03_Proche-1.doc - 4:16 [R : En français c’est l’épilep..] (69:69) (Super)**

Codes: [Diagnostic-Antécédants_Médicaux]

No memos

R : En français c’est l’épilepsie

**P15: Ktw_HopMtda_Audit_dcd-05_Proche-1.doc - 15:72 [R. Je finis par là] (293:293) (Super)**

Codes: [Ebola_considérations-opinions]

No memos

R. Je finis par là

**P15: Ktw_HopMtda_Audit_dcd-05_Proche-1.doc - 15:71 [R. Je ne sais pas] (290:290) (Super)**

Codes: [Ebola_considérations-opinions]

No memos

R. Je ne sais pas

**P13: Ktw_Hgr_Audit_dcd-08_Proche-2.doc - 13:36 [R : Nombreux disent que c’est ..] (149:149) (Super)**

Codes: [Ebola_considérations-opinions]

No memos

R : Nombreux disent que c’est virus fondé, que ce n’est ne pas un virus qui était dans d’autres provinces car il avait meme un médecin qui voulait le nommer « un virus Mangina » par ce que c’est le milieu où l’épidémie avait commencé mais il fut assassiné par fusillade. La population a compris qu’il été tué par ce qu’il voulait changer le nom au détriment de ce qui sont dans la riposte, par ce que cette dernière voulait bénéficier au nom d’Ebola. Il était tué à Mangina pour cette cause.

**P16: Ktw_HopMtda_Audit_dcd-05_Proche-2.doc - 16:40 [R. Les gens d’ici ont peur de ..] (209:209) (Super)**

Codes: [Ebola_considérations-opinions]

No memos

R. Les gens d’ici ont peur de l’Ebola ou d’être traité d’un malade à Ebola parce que dans tous les hôpitaux il y a des cas. Certains trompent lorsqu’on y arrive. Cette maladie a tué beaucoup de personnes raisons pour laquelle beaucoup la craignent.

**P16: Ktw_HopMtda_Audit_dcd-05_Proche-2.doc - 16:41 [R. Elles ont compris que c’est..] (217:217) (Super)**

Codes: [Ebola_considérations-opinions]

No memos

R. Elles ont compris que c’est une maladie après beaucoup de pertes des vies humaines. Actuellement elles insistent sur la propreté.

**P15: Ktw_HopMtda_Audit_dcd-05_Proche-1.doc - 15:73 [R. Je ne sais pas] (296:296) (Super)**

Codes: [Ebola_considérations-opinions]

No memos

R. Je ne sais pas

**P 8: Bbo_HgrKttmba_Audit_dcd-04_Proche-2.doc - 8:34 [R : Certains recevaient la nou..] (142:142) (Super)**

Codes: [Ebola_considérations-opinions]

No memos

R : Certains recevaient la nourriture d’autres de l’argents.

**P 8: Bbo_HgrKttmba_Audit_dcd-04_Proche-2.doc - 8:39 [R : Les autres dans la communa..] (160:160) (Super)**

Codes: [Ebola_considérations-opinions]

No memos

R : Les autres dans la communauté disent même chose que ce que je venais de vous dire, comme une maladie politisée. Nombreux les disaient à haute voix que ce n’était pas une maladie. Ces gens premièrement étaient des nouveaux dans le milieu et avaient recrutés les autorités locales pour surveiller dans la communauté en vérifiant celui qui présentera la fièvre et la toux il appel aux gens de la reposte. Même si vous vous reposez un peu à l’extérieur vous aller trouvez une ambulance devant votre porte pour vous prendre. Cela a prouvé que cette maladie était politisée. Et si quelqu’un meurt on vient on brule tous vos effets et celui qui lui a touché qui lui a vu, vous tous c’est la mort.

**P 6: Bbo_HgrKttmba_Audit_dcd-03_Proche-3.doc - 6:56 [R : Oui je savais que ITA c’es..] (222:222) (Super)**

Codes: [Ebola_considérations-opinions]

No memos

R : Oui je savais que ITA c’est l’argent et on doit y mourir, je suis allé au CTE récemment jadis je ne pouvais pas y aller. C’est ce qui faisait douter les gens, quand vous cachez on parvenait à guérir ; mais si vous allez à l’hôpital il y a risque de mourir. Alors on se demandait comment les gens meurt alors que ceux qui restent à la maison ne meurent pas.

**P 8: Bbo_HgrKttmba_Audit_dcd-04_Proche-2.doc - 8:35 [R : Ebola c’est une maladie po..] (146:146) (Super)**

Codes: [Ebola_considérations-opinions]

No memos

R : Ebola c’est une maladie politisée et je demande à Dieu de me pardonner par ce que cette maladie ne devrait pas être ici chez nous. Jadis on pouvait avoir une température de 40°c et on vous soignera, mais dans la période de l’épidémie avec 37°,38°c on appelait l’ambulance pour vous transporter au CTE même la fièvre ordinaire on doit vous référer. Alors qu’à l’époque on pouvait venir avec la diarrhée, la toux et même si vous faites le vomissement du sang les prestataires des soins étaient disposés à vous accueillir sans problème et s’arrangent pour que vous soyez guéri.

**P 6: Bbo_HgrKttmba_Audit_dcd-03_Proche-3.doc - 6:55 [R : la maladie a créé beaucoup..] (217:218) (Super)**

Codes: [Ebola_considérations-opinions]

No memos

R : la maladie a créé beaucoup de conflits dans la communauté, car il y avait un terme vulgaire « ITA » qui signifie appeler. C’était une façon d’alerter pour quelqu’un qui était malade et il fallait appeler pour qu’on l’amène aux soins ce qui n’était pas apprécié par la famille qui avait le malade. Maintenant si quelqu’un a appelé, il reste en conflit avec la communauté.

Si le malade mourrait à hôpital, la personne qui a alertée est considérée comme quoi elle a reçu l’argent pour l’alerte et pour que le malade y décède. Et le conflit reste jusqu’à présent et il n’y aura pas de réconciliation pour certains ; surtout les relais communautaires. Il arrivait de cachait même les malades du fait qu’on alerte pas ; moi-même j’étais malade j’étais resté da la maison sans informer à quelqu’un.

**P 6: Bbo_HgrKttmba_Audit_dcd-03_Proche-3.doc - 6:54 [R : C’est une maladie grave qu..] (213:213) (Super)**

Codes: [Ebola_considérations-opinions]

No memos

R : C’est une maladie grave qui a tué beaucoup de personnes.

**P16: Ktw_HopMtda_Audit_dcd-05_Proche-2.doc - 16:47 [R. Il y a ceux-là qui étaient ..] (236:236) (Super)**

Codes: [Ebola_considérations-opinions]

No memos

R. Il y a ceux-là qui étaient guéris mais on avait la peur de comprendre que l’on amène quelqu’un au CTE car on le considérait comme déjà mort. Mais certains rentraient étant guéris.

**P15: Ktw_HopMtda_Audit_dcd-05_Proche-1.doc - 15:70 [00 :20 :43 R. C’est une mauvai..] (282:283) (Super)**

Codes: [Ebola_considérations-opinions]

No memos

00 :20 :43

R. C’est une mauvaise maladie

**P 6: Bbo_HgrKttmba_Audit_dcd-03_Proche-3.doc - 6:53 [R : La communauté a compris qu..] (209:209) (Super)**

Codes: [Ebola_considérations-opinions]

No memos

R : La communauté a compris que c’est une maladie mais pas Ebola qu’ils avaient déjà entendu. Par ce qu’ils n’ont pas vus le sang qui coulait dans les orifices comme signe clinique selon des informations et des images des autres provinces.Pendant cette periode on avait pas publié un cas qui avait des signes similaire à Ebola. Aucun cas qui était décédé avec le sang comme signe. Ils disent que c’est un virus semblable au Covid 19 que les blancs fabriquent.

**P 8: Bbo_HgrKttmba_Audit_dcd-04_Proche-2.doc - 8:33 [R : Certains considéraient com..] (138:138) (Super)**

Codes: [Ebola_considérations-opinions]

No memos

R : Certains considéraient comme un business ; certains y partaient pour gagner de l’argent d’autres pour gagner la nourriture.

**P 8: Bbo_HgrKttmba_Audit_dcd-04_Proche-2.doc - 8:23 [R : Les Diabétiques ont leurs ..] (98:98) (Super)**

Codes: [Ebola_considérations-opinions]

No memos

R : Les Diabétiques ont leurs traitements en injection, le temps qu’elle était là je ne sais pas ce que on lui avait parlé comme moi je suivais les soignants pour son traitement et ce que j’avais.

**P19: Ktw_HopMtda_Audit_dcd-10_Proche-1.doc - 19:32 [R : Ils disent que ceux qui av..] (152:152) (Super)**

Codes: [Ebola_considérations-opinions]

No memos

R : Ils disent que ceux qui avaient des malades, quand ils arrivaient aux CTE, les soignants qui s’y trouvaient étaient retissant envers les patients, ils soignaient avec retard, ils ne faisaient pas vraiment l’entretien des patients et ils le laissaient souffrir. Ils se demandaient comment un soignant peut avoir peur d’un malade ?

**P 7: Bbo_HgrKttmba_Audit_dcd-04_Proche-1.doc - 7:48 [R : Par ce que c’est une malad..] (199:199) (Super)**

Codes: [Ebola_considérations-opinions]

No memos

R : Par ce que c’est une maladie qui est venue sous forme des maladies ordinaires comme la fièvre, la toux, la diarrhée etc...

**P 7: Bbo_HgrKttmba_Audit_dcd-04_Proche-1.doc - 7:50 [R : Les un disent que c’est un..] (207:207) (Super)**

Codes: [Ebola_considérations-opinions]

No memos

R : Les un disent que c’est une maladie surnaturelle.

**P 7: Bbo_HgrKttmba_Audit_dcd-04_Proche-1.doc - 7:49 [R : Elle considère que c’est u..] (203:203) (Super)**

Codes: [Ebola_considérations-opinions]

No memos

R : Elle considère que c’est une maladie mais d’origine inconnue.

**P14: Ktw_Hgr_Audit_dcd-09_Proche-1.doc - 14:40 [R : Dans notre cellule ils ont..] (169:169) (Super)**

Codes: [Ebola_considérations-opinions]

No memos

R : Dans notre cellule ils ont compris que c’était une épidémie dangereuse et ils avaient quitté l’ignorance, ils sont bien. Et maintenant nous leurs sensibilisons sur le Covid19, ils nous demandent de leurs aider pour lutter contre cette pandémie.

**P18: Ktw_HopMtda_Audit_dcd-06_Proche-1.doc - 18:73 [R. On disait que l’on tuait de..] (303:303) (Super)**

Codes: [Ebola_considérations-opinions]

No memos

R. On disait que l’on tuait des personnes au CTE, on les cassait à travers des marteaux, on les injectait au coup. C’étaient ça les rumeurs de la population

**P19: Ktw_HopMtda_Audit_dcd-10_Proche-1.doc - 19:31 [R : La communauté dit que c’es..] (147:147) (Super)**

Codes: [Ebola_considérations-opinions]

No memos

R : La communauté dit que c’est un virus fabriqué.

**P12: Ktw_Hgr_Audit_dcd-08_Proche-12.doc - 12:40 [R : Je ne peux pas connaitre p..] (171:171) (Super)**

Codes: [Ebola_considérations-opinions]

No memos

R : Je ne peux pas connaitre pour les autres car ici à Butembo KIPE YA YO (occupe de tes affaires) mais selon moi c’est une maladie.

**P20: Ktw_HopMtda_Audit_dcd-11_Proche-1.doc - 20:31 [R : La critique ne manque pas,..] (139:139) (Super)**

Codes: [Ebola_considérations-opinions]

No memos

R : La critique ne manque pas, certains disent que ce n’est ne pas une maladie, qu’on tuait seulement les gens soit disant que c’est créé mais moi je pense que c’est une épidémie.

**P 7: Bbo_HgrKttmba_Audit_dcd-04_Proche-1.doc - 7:51 [R : C’est très grave.] (211:211) (Super)**

Codes: [Ebola_considérations-opinions]

No memos

R : C’est très grave.

**P 4: Bbo_HgrKttmba_Audit_dcd-03_Proche-1.doc - 4:57 [R : Ebola c’est une maladie gr..] (236:236) (Super)**

Codes: [Ebola_considérations-opinions]

No memos

R : Ebola c’est une maladie grave, que je ne peux plus vouloir son retour dans notre milieu.

**P 7: Bbo_HgrKttmba_Audit_dcd-04_Proche-1.doc - 7:53 [R : Manque d’amour et l’incomp..] (219:219) (Super)**

Codes: [Ebola_considérations-opinions]

No memos

R : Manque d’amour et l’incompréhension.

**P 4: Bbo_HgrKttmba_Audit_dcd-03_Proche-1.doc - 4:58 [R : Les uns disent que l’Ebola..] (240:240) (Super)**

Codes: [Ebola_considérations-opinions]

No memos

R : Les uns disent que l’Ebola n’existe pas ; que c’est du mensonge

**P 4: Bbo_HgrKttmba_Audit_dcd-03_Proche-1.doc - 4:61 [R : Par ce que beaucoup de per..] (252:252) (Super)**

Codes: [Ebola_considérations-opinions]

No memos

R : Par ce que beaucoup de personnes ont perdu leurs vies.

**P 4: Bbo_HgrKttmba_Audit_dcd-03_Proche-1.doc - 4:59 [R : Les autres ont peur de cet..] (244:244) (Super)**

Codes: [Ebola_considérations-opinions]

No memos

R : Les autres ont peur de cette maladie comme moi.

**P19: Ktw_HopMtda_Audit_dcd-10_Proche-1.doc - 19:34 [R : Oui, elle était grave.] (161:161) (Super)**

Codes: [Ebola_considérations-opinions]

No memos

R : Oui, elle était grave.

**P 7: Bbo_HgrKttmba_Audit_dcd-04_Proche-1.doc - 7:52 [R : Il y a eu beaucoup de mort..] (215:215) (Super)**

Codes: [Ebola_considérations-opinions]

No memos

R : Il y a eu beaucoup de morts papa.

**P11: Ktw_Hgr_Audit_dcd-08_Proche-1.doc - 11:66 [R/ Comme dans ma cellule ici l..] (267:267) (Super)**

Codes: [Ebola_considérations-opinions]

No memos

R/ Comme dans ma cellule ici les gens se révoltent beaucoup contre Ebola,

**P11: Ktw_Hgr_Audit_dcd-08_Proche-1.doc - 11:69 [R : Avant Ebola notre centre i..] (279:279) (Super)**

Codes: [Ebola_considérations-opinions]

No memos

R : Avant Ebola notre centre ici avait trop de fréquentation mais quand Ebola est arrivé tout le monde s’est retiré, peut être comme on dit qu’on va proclamer la fin d’Ebola que la fréquentation peut recommencer.

**P11: Ktw_Hgr_Audit_dcd-08_Proche-1.doc - 11:67 [R/ Ebola est une maladie grave..] (271:271) (Super)**

Codes: [Ebola_considérations-opinions]

No memos

R/ Ebola est une maladie grave par ce qu’elle tue, c’est vrai on nous dit toujours qu’il ne faut pas toucher les cadavres comme nous le faisions avant car il ya déjà EBOLA.

**P18: Ktw_HopMtda_Audit_dcd-06_Proche-1.doc - 18:75 [R. Ils disent que l’EBOLA est ..] (311:311) (Super)**

Codes: [Ebola_considérations-opinions]

No memos

R. Ils disent que l’EBOLA est un montage ; Est une maladie qui n’existe pas. Et cette maladie est envoyée par quelques personnes.

**P19: Ktw_HopMtda_Audit_dcd-10_Proche-1.doc - 19:30 [R : la communauté dit que l’éq..] (143:143) (Super)**

Codes: [Ebola_considérations-opinions]

No memos

R : la communauté dit que l’équipe de riposte est venue avec des gens qui ne sont pas qualifiés dans le domaine de santé.

**P 5: Bbo_HgrKttmba_Audit_dcd-03_Proche-2.doc - 5:68 [R : Comme je te le dis, nous a..] (262:262) (Super)**

Codes: [Ebola_considérations-opinions]

No memos

R : Comme je te le dis, nous avons vu Ebola et la communauté ne savait pas et par surprise nous avons été informé que l’Ebola vient et nous on se demandait la ressemblance de cet Ebola on a pas trouvé. Mais les autres disaient que c’est un esprit qu’on jetait sur les gens pour qu’ils tombent malade.

**P18: Ktw_HopMtda_Audit_dcd-06_Proche-1.doc - 18:76 [R. Malgré qu’ils disaient que ..] (315:315) (Super)**

Codes: [Ebola_considérations-opinions]

No memos

R. Malgré qu’ils disaient que c’est une maladie montée par certains, ils se protégeaient contre elles car tout le monde suivait les instructions qui étaient données en ce sens que personne ne pouvait toucher un cadavre. Malgré ces rumeurs, ils se protégeaient

**P 8: Bbo_HgrKttmba_Audit_dcd-04_Proche-2.doc - 8:32 [R : Je ne sais pas. Vous allez..] (134:134) (Super)**

Codes: [Ebola_considérations-opinions]

No memos

R : Je ne sais pas. Vous allez trouver quelqu’un qui vient du CTE, les soignants se donnent pour sa prise en charge et son état de santé s’améliore mais après quelques jours il rechute et à la fin de compte il meurt.

**P18: Ktw_HopMtda_Audit_dcd-06_Proche-1.doc - 18:74 [R. Moi, je ne tenais pas compt..] (307:307) (Super)**

Codes: [Ebola_considérations-opinions]

No memos

R. Moi, je ne tenais pas compte de ces rumeurs. Ils disaient cela parce qu’ils n’étaient pas encore tombés malades.

**P 5: Bbo_HgrKttmba_Audit_dcd-03_Proche-2.doc - 5:72 [R : Ebola c’est une mauvaise m..] (278:278) (Super)**

Codes: [Ebola_considérations-opinions]

No memos

R : Ebola c’est une mauvaise maladie qui a tué beaucoup de personnes dans notre milieu et elle vient de finir une autre apparait, on se demande c’est quoi peut être c’est une maladie jetée sur la population.

**P 5: Bbo_HgrKttmba_Audit_dcd-03_Proche-2.doc - 5:71 [R : Je peux vous expliquer sel..] (274:274) (Super)**

Codes: [Ebola_considérations-opinions]

No memos

R : Je peux vous expliquer selon ma connaissance, je vois que cette maladie qui vient des instances supérieures et ils savent les jours où la maladie finira mais une maladie on ne peut pas savoir le jour où elle finira.

**P 5: Bbo_HgrKttmba_Audit_dcd-03_Proche-2.doc - 5:70 [R : C’est ma façon de comprend..] (270:270) (Super)**

Codes: [Ebola_considérations-opinions]

No memos

R : C’est ma façon de comprendre la situation.

**P 5: Bbo_HgrKttmba_Audit_dcd-03_Proche-2.doc - 5:69 [R : Moi personnellement étant ..] (266:266) (Super)**

Codes: [Ebola_considérations-opinions]

No memos

R : Moi personnellement étant chrétienne, je considère Ebola comme un mauvais esprit prêché dans l’église qu’il viendra dans les derniers jours des choses et maladies horribles. Alors moi je le comprends de cette manière que ces sont les écritures bibliques qui s’accomplissent.

**P 5: Bbo_HgrKttmba_Audit_dcd-03_Proche-2.doc - 5:73 [R : la population dit ce que j..] (282:282) (Super)**

Codes: [Ebola_considérations-opinions]

No memos

R : la population dit ce que je vous ai dit qu Ebola était amené et jadis cette maladie n’existait pas et les autres disaient qu’elle était.

**P17: Ktw_HopMtda_Audit_dcd-05_Proche-3.doc - 17:40 [R. Cela, je ne connais pas.] (168:168) (Super)**

Codes: [Equipe riposte_Considération_Opinions]

No memos

R. Cela, je ne connais pas.

**P 5: Bbo_HgrKttmba_Audit_dcd-03_Proche-2.doc - 5:74 [R : J’entendais dans l’avenue ..] (286:286) (Super)**

Codes: [Equipe riposte_Considération_Opinions]

No memos

R : J’entendais dans l’avenue on disait qu’on nous a amené des gens qui ont la maladie pour nous tuer et que les rwandais viennent s’installer.

**P11: Ktw_Hgr_Audit_dcd-08_Proche-1.doc - 11:68 [R : Ils sont venus chercher de..] (275:275) (Super)**

Codes: [Equipe riposte_Considération_Opinions]

No memos

R : Ils sont venus chercher de l’argent, c’est ce qu’ils disaient ou s’ils passaient les gens disaient « voici les gens d’Ebola »

**P 6: Bbo_HgrKttmba_Audit_dcd-03_Proche-3.doc - 6:58 [R : Peut-être tout début est d..] (232:232) (Super)**

Codes: [Equipe riposte_Considération_Opinions]

No memos

R : Peut-être tout début est difficile, mais leurs stratégies avaient échoué au début par ce que les gens qui étaient venus n’étaient pas familiers à la communauté et ils parlaient la langue non connue il fallait un interprète pour se rapprocher de la communauté. Ils se sont retrouvés un peu tard avec plusieurs dégâts en vies humaines et c’est après qu’ils commencé à recruter les agents locaux et c’est ainsi qu’avait commencé à voir les relais communautaires et chacun dans sa communauté. Mais cette stratégie de recruter au niveau supérieur les avait rendus un mauvais service et qui a entrainé la persistance de la maladie.

**P 6: Bbo_HgrKttmba_Audit_dcd-03_Proche-3.doc - 6:57 [Pour quoi vous souriez d’abord..] (226:228) (Super)**

Codes: [Equipe riposte_Considération_Opinions]

No memos

Pour quoi vous souriez d’abord ?

00 :34 :19

R : Je ne parle pas pour toute la communauté mais avec le peu que j’entends l’équipe de la riposte était venue pour nous aider mais en premier lieu ils sont venus pour leurs intérêts personnels. Comme il y avait beaucoup d’argent ils ne voulaient pas que la maladie finisse pour que cet argent ne retourne pas chez les partenaires. Mais l’épidémie devrait finir avant Par exemple hier sous la pluie je passais et j’entendais me voisins entrain de citer un autre voisin qui venais de construire trois maisons à cause de la riposte et ils énuméraient de la première jusqu’à la troisième. C’est un conflit avec l’équipe de la riposte par ce que pour certains s’enrichissaient au détriment de ceux qui ont perdu les leurs.

**P 4: Bbo_HgrKttmba_Audit_dcd-03_Proche-1.doc - 4:60 [R : Ils ont bien travaillé] (248:248) (Super)**

Codes: [Equipe riposte_Considération_Opinions]

No memos

R : Ils ont bien travaillé

**P 4: Bbo_HgrKttmba_Audit_dcd-03_Proche-1.doc - 4:62 [R : Ils ont bien travaillé, pa..] (256:256) (Super)**

Codes: [Equipe riposte_Considération_Opinions]

No memos

R : Ils ont bien travaillé, par ce qu’ils avaient créé des petits groupes de RECO pour la surveillance de l’épidémie dans la communauté pour la sensibilisation et voir celui qui a la fièvre. Celui qui présentera les signes d’Ebola.

**P 5: Bbo_HgrKttmba_Audit_dcd-03_Proche-2.doc - 5:76 [R : Tu m’as demandé ceux qu’il..] (294:294) (Super)**

Codes: [Equipe riposte_Considération_Opinions]

No memos

R : Tu m’as demandé ceux qu’ils disaient. Comme ils avaient échoué dans leurs plans c’est ainsi qu’ils ont amené la maladie.

**P 5: Bbo_HgrKttmba_Audit_dcd-03_Proche-2.doc - 5:77 [R : Ceux du milieu passaient d..] (298:298) (Super)**

Codes: [Equipe riposte_Considération_Opinions]

No memos

R : Ceux du milieu passaient dans les avenues et chaque parcelle en nous demandaient si nous avons déjà entendu de l’Ebola, ceux qui avaient déjà entendu acceptaient et ceux qui n’avait pas encore entendus refusaient. Même les grands-pères nous disaient que cette maladie était là mais cette maladie on pouvait le bloquer et ça ne va plus se propager.

**P13: Ktw_Hgr_Audit_dcd-08_Proche-2.doc - 13:37 [R : La communauté dit que Kins..] (153:153) (Super)**

Codes: [Equipe riposte_Considération_Opinions]

No memos

R : La communauté dit que Kinshasa avait envoyé les gens qui ne sont pas, pas expertise en médecine. Là on formatait les gens, c’était comme le dit souvent les congolais « un Cop » quelque chose de gain que devrait se partager entre connaissance ou famille. Alors on vous donne la personne pour travailler et c’est pour cette raison qu’il y a eu beaucoup de décès pendant cette période, ce n’était pas de vrais médecins qualifiés pour faire ce travail. C’est ce que la communauté dit jusqu’à présent. Et si Kinshasa pouvait considérer les prestataires locaux il n’aurait pas beaucoup de perte en vies humaines.

**P17: Ktw_HopMtda_Audit_dcd-05_Proche-3.doc - 17:41 [R. Ils passaient au quartier.] (172:172) (Super)**

Codes: [Equipe riposte_Considération_Opinions]

No memos

R. Ils passaient au quartier.

**P 7: Bbo_HgrKttmba_Audit_dcd-04_Proche-1.doc - 7:54 [R : Elle avait bien travaillé ..] (223:223) (Super)**

Codes: [Equipe riposte_Considération_Opinions]

No memos

R : Elle avait bien travaillé mais la famille qui a connu le décès ne peut pas les apprécier par ce qu’on ne croyait pas à la maladie et souvent c’était la jalousie ou la sorcellerie. A chaque décès il y avait toujours d’autres causes même si c’est Ebola.

**P18: Ktw_HopMtda_Audit_dcd-06_Proche-1.doc - 18:78 [R. Pour confirmer l’existence ..] (323:323) (Super)**

Codes: [Equipe riposte_Considération_Opinions]

No memos

R. Pour confirmer l’existence de la maladie pourtant contestée par la population.

**P 5: Bbo_HgrKttmba_Audit_dcd-03_Proche-2.doc - 5:75 [R : Oui pour que nous tous nou..] (290:290) (Super)**

Codes: [Equipe riposte_Considération_Opinions]

No memos

R : Oui pour que nous tous nous mourrons et que d’autre personne viennent s’installer dans leurs milieux.

**P 9: Bbo_HopScola_Audit_dcd-07_Proche-1.doc - 9:64 [R : Aujourd’hui ils se rendent..] (288:288) (Super)**

Codes: [Equipe riposte_Considération_Opinions]

No memos

R : Aujourd’hui ils se rendent comptent que si beaucoup des gens sont mort c’est à cause de l’ignorance, si la communauté pourrait comprendre dès le début les gens ne pouvaient pas mourir ainsi.

**P 8: Bbo_HgrKttmba_Audit_dcd-04_Proche-2.doc - 8:36 [R : Ils uns étaient content de..] (150:150) (Super)**

Codes: [Equipe riposte_Considération_Opinions]

No memos

R : Ils uns étaient content de leurs revenus pour le travail fait.

**P12: Ktw_Hgr_Audit_dcd-08_Proche-12.doc - 12:41 [R : Je ne sais pas car maman é..] (175:175) (Super)**

Codes: [Equipe riposte_Considération_Opinions]

No memos

R : Je ne sais pas car maman était décédée ils étaient venus pour faire l’examen de son corps mais c’est leurs droits de faire leur travail.

**P 9: Bbo_HopScola_Audit_dcd-07_Proche-1.doc - 9:63 [R : Les gens n’acceptent pas ç..] (284:284) (Super)**

Codes: [Equipe riposte_Considération_Opinions]

No memos

R : Les gens n’acceptent pas ça. Faire comprendre que le médecin ne peut pas arrêter la vie à cause de l’argent, c’est très difficile, il y’a des gens qui n’ont même pas étudier alors pour eux comprendre c’est très difficile.

**P16: Ktw_HopMtda_Audit_dcd-05_Proche-2.doc - 16:44 [R. Oui, parce qu’on aura un pr..] (225:225) (Super)**

Codes: [Equipe riposte_Considération_Opinions]

No memos

R. Oui, parce qu’on aura un problème de communication dans le traitement

**P14: Ktw_Hgr_Audit_dcd-09_Proche-1.doc - 14:42 [R : Ils ont compris par ce qu’..] (178:178) (Super)**

Codes: [Equipe riposte_Considération_Opinions]

No memos

R : Ils ont compris par ce qu’ils avaient vu une perte énorme en vie humaine, malgré que dans notre cellule il n’y avait pas de cas mais lorsque on recevait les information des autres cellules tel et tel ne sont plus, ils ont fini par comprendre la situation

**P16: Ktw_HopMtda_Audit_dcd-05_Proche-2.doc - 16:43 [R. On ne les connais pas (d’où..] (221:221) (Super)**

Codes: [Equipe riposte_Considération_Opinions]

No memos

R. On ne les connais pas (d’où ils sont venus). On ne savait pas identifier même un seul agent parmi l’équipe

**P14: Ktw_Hgr_Audit_dcd-09_Proche-1.doc - 14:41 [R : Dans la communauté il y a ..] (174:174) (Super)**

Codes: [Equipe riposte_Considération_Opinions]

No memos

R : Dans la communauté il y a toute catégorie des gens. Il y a les délinquants et les gens sensibilisés mais la majorité ont compris que la riposte a travaillé et continue de travailler. Alors que jadis elle ne comprenait pas la riposte, à l’époque lorsqu’une personne décédait on s’asseyait mais actuellement même si c’est un familier on lui laisse là en attendant l’équipe de la riposte vienne décontaminer même s’il n’y a plus les cas d’Ebola nous attendons toujours. Même si vous allez compatir vous devez s’éloigner du cadavre.

**P16: Ktw_HopMtda_Audit_dcd-05_Proche-2.doc - 16:48 [R. Cela n’est pas aussi un pro..] (240:240) (Super)**

Codes: [Equipe riposte_Considération_Opinions]

No memos

R. Cela n’est pas aussi un problème parce qu’on ne sait pas dire que ceux qui mouraient étaient causés par les gens de l’équipe de la riposte. Les soignants leur donner le traitement.

**P20: Ktw_HopMtda_Audit_dcd-11_Proche-1.doc - 20:32 [R : Ils n’ont pas à dire par c..] (143:143) (Super)**

Codes: [Equipe riposte_Considération_Opinions]

No memos

R : Ils n’ont pas à dire par ce que la population a besoin d’être guérie et aussi cette équipe de riposte vient de faire longtemps et elle a le souci que la population guérisse.

**P16: Ktw_HopMtda_Audit_dcd-05_Proche-2.doc - 16:46 [R. Cette équipe était formée d..] (232:232) (Super)**

Codes: [Equipe riposte_Considération_Opinions]

No memos

R. Cette équipe était formée des prestataires que la population ne connaissait pas leurs origines. Alors lorsqu’il y avait un transfert vers le CTE c’était un cas de décès, on disait que le défunt a été injecté de l’eau.

**P16: Ktw_HopMtda_Audit_dcd-05_Proche-2.doc - 16:45 [R. Je ne connais pas] (229:229) (Super)**

Codes: [Equipe riposte_Considération_Opinions]

No memos

R. Je ne connais pas

**P18: Ktw_HopMtda_Audit_dcd-06_Proche-1.doc - 18:77 [R. On disait que cette équipe ..] (319:319) (Super)**

Codes: [Equipe riposte_Considération_Opinions]

No memos

R. On disait que cette équipe a déjà eu sa part BALIRE ; Littéralement : « ils ont déjà mangé » pour dire qu’ils sont complices parce qu’ils gagnent de l’argent dans cette affaire.

**P16: Ktw_HopMtda_Audit_dcd-05_Proche-2.doc - 16:49 [R. On savait que lorsque quelq..] (244:244) (Super)**

Codes: [Equipe riposte_Considération_Opinions]

No memos

R. On savait que lorsque quelqu’un est amené au CTE, sa guérison était incertaine

**P19: Ktw_HopMtda_Audit_dcd-10_Proche-1.doc - 19:33 [R : La communauté disait qu’il..] (156:156) (Super)**

Codes: [Equipe riposte_Considération_Opinions]

No memos

R : La communauté disait qu’ils sont venus s’enrichir, en manipulant les faibles économiquement. Les gens qui n’avaient pas étudié ne touchaient qu’un salaire très minime. Alors qu’eux avaient un salaire très colossal. Et ils préféraient recruter les gens qui n’ont pas étudié pour le manipuler par rapport à la rémunération et de peur d’être traduit à la justice. Les gens qui n’ont pas une vision quelconque.

**P 9: Bbo_HopScola_Audit_dcd-07_Proche-1.doc - 9:62 [R : La population disait qu’il..] (279:279) (Super)**

Codes: [Equipe riposte_Considération_Opinions]

No memos

R : La population disait qu’ils sont venus tuer les gens pour gagner de l’argent.

**P 8: Bbo_HgrKttmba_Audit_dcd-04_Proche-2.doc - 8:12 [R : Son état était gravement m..] (50:50) (Super)**

Codes: [Etat_Arrivée_Hospitalisation]

No memos

R : Son état était gravement malade et déshydraté par ce ça faisait longtemps qu’elle restait dans la maison sans se déplacer. Elle ne pouvait pas manger la nourriture qui contient ni du sel et de l’huile. C’est la déshydratation qui était la cause d’aller à l’hôpital lorsqu’elle arriva à l’hôpital après la réhydratation sa santé se rétabli très vite.

**P 9: Bbo_HopScola_Audit_dcd-07_Proche-1.doc - 9:15 [R : Il marchait seul jusqu’à l..] (69:69) (Super)**

Codes: [Etat_Arrivée_Hospitalisation]

No memos

R : Il marchait seul jusqu’à l’hôpital et nous on l’avait juste accompagné.

**P 7: Bbo_HgrKttmba_Audit_dcd-04_Proche-1.doc - 7:21 [R : Je n’étais pas là lorsqu’o..] (86:86) (Super)**

Codes: [Etat_Arrivée_Hospitalisation]

No memos

R : Je n’étais pas là lorsqu’on l’avait amené à l’hôpital.

**P11: Ktw_Hgr_Audit_dcd-08_Proche-1.doc - 11:27 [R : Il avait beaucoup maigri.] (112:112) (Super)**

Codes: [Etat_Arrivée_Hospitalisation]

No memos

R : Il avait beaucoup maigri.

**P11: Ktw_Hgr_Audit_dcd-08_Proche-1.doc - 11:26 [R/ j’avais quitté la maison qu..] (108:108) (Super)**

Codes: [Etat_Arrivée_Hospitalisation]

No memos

R/ j’avais quitté la maison quand mon mari était déjà fâché disant que moi j’aime trop le traitement indigène plus que le traitement moderne, c’est là que je l’avais amené ici ; mais l’enfant était déjà trop mince.

**P11: Ktw_Hgr_Audit_dcd-08_Proche-1.doc - 11:29 [R/ En mangeant la nourriture ;..] (120:120) (Super)**

Codes: [Etat_Arrivée_Hospitalisation]

No memos

R/ En mangeant la nourriture ; par ce que je l’avais amené ici il ne mangeait plus, il triait la nourriture quand il y avait de la viande ou du poisson il mangeait mais lorsque c’était autre chose comme le feuille de manioc et qu’il n’y a rien de dans mangeait pas. (KULATA)

**P11: Ktw_Hgr_Audit_dcd-08_Proche-1.doc - 11:28 [R : Il avait récupéré un peu] (116:116) (Super)**

Codes: [Etat_Arrivée_Hospitalisation]

No memos

R : Il avait récupéré un peu

**P19: Ktw_HopMtda_Audit_dcd-10_Proche-1.doc - 19:11 [R : Elle avait déjà fait deux ..] (53:53) (Super)**

Codes: [Etat_Arrivée_Hospitalisation]

No memos

R : Elle avait déjà fait deux mois à la maison par ce qu’elle était encore prise en charge à Matanda antérieurement et après son hospitalisation elle retourna à la maison. Etant à la maison, elle avait encore rechuté, c’est ainsi qu’on l’amena encore à ce même l’hôpital.

**P 6: Bbo_HgrKttmba_Audit_dcd-03_Proche-3.doc - 6:23 [R : Son état était un peu bien..] (90:90) (Super)**

Codes: [Etat_Arrivée_Hospitalisation]

No memos

R : Son état était un peu bien mais quand elle est partie au CTE, c’est à ce moment-là qu’elle est revenue étant gravement malade son état était critique et on avait dit qu’elle n’avait pas d’Ebola.

**P14: Ktw_Hgr_Audit_dcd-09_Proche-1.doc - 14:7 [R : Son état de santé était tr..] (29:29) (Super)**

Codes: [Etat_Arrivée_Hospitalisation]

No memos

R : Son état de santé était très médiocre par ce qu’il avait maigri et ses plaintes étaient la céphalée et les douleurs abdominales. Ils nous avaient dit qu’il n’était pas déshydraté ni anémique mais qu’il souffre de la gastrite et le paludisme seulement. Nous leur avons demandé de nous aider qu’il fasse l’échographie. Mais pour la deuxième hospitalisation j’étais partis chercher un radiologue pour qu’il nous aide à lui faire passer à l’échographie comme nous il était hospitalisé le samedi et c’était le dimanche que j’étais allée chercher le radiologue et je lui avais dit que la santé du malade n’est pas bonne, il me répondu qu’il ne travaille pas ce jour et qu’il va lui programmer pour le lundi. Après échographie on nous dira qu’ils avaient vu en bas du foie quelque chose que est comme un cancer. J’ai trouvé que la gratuité ne sert à rien peut être pour le paludisme seulement par ce qu’on nous servait le médicament de cinq jours alors que les antibiotique la cure va sept jusqu’à dix jours.

**P13: Ktw_Hgr_Audit_dcd-08_Proche-2.doc - 13:17 [R : Il était gravement malade ..] (71:71) (Super)**

Codes: [Etat_Arrivée_Hospitalisation]

No memos

R : Il était gravement malade parce qu’il fallait l’ambulance pour qu’il arrive à l’hôpital, de la maison vers le centre de santé où l’ambulance devait lui récupérer on l’avait porté au dos. Mais quand on était allé au bureau central, sa sante n’était pas vraiment médiocre il marchait seul. Mais aussi, on avait déjà fait six mois au centre de santé et ils n’avaient toujours pas diagnostiqué la pathologie. C’est alors qu’ils sont parvenus à nous transférer après une longue durée.

**P12: Ktw_Hgr_Audit_dcd-08_Proche-12.doc - 12:11 [R : Elle était un peu bien par..] (50:50) (Super)**

Codes: [Etat_Arrivée_Hospitalisation]

No memos

R : Elle était un peu bien par ce qu’on collaborait sans problème et s’alimentait c’est en arrivant au soins intensif que sa santé s’était aggravée

**P12: Ktw_Hgr_Audit_dcd-08_Proche-12.doc - 12:12 [R : C’est l’endroit où on ajou..] (54:54) (Super)**

Codes: [Etat_Arrivée_Hospitalisation]

No memos

R : C’est l’endroit où on ajoute la respiration (soins intensifs)

**P 5: Bbo_HgrKttmba_Audit_dcd-03_Proche-2.doc - 5:27 [R : Lorsqu’elle était transfér..] (107:107) (Super)**

Codes: [Etat_Arrivée_Hospitalisation]

No memos

R : Lorsqu’elle était transférée, elle avait passé deux jours à la maison et après elle avait demandé qu’on lui ramène à l’hôpital alors qu’on lui avait dit d’aller immédiatement vers la structure où elle était référée et lui qui insista qu’elle doit passer chez elle, le taxi man l’amena à la maison.

**P 5: Bbo_HgrKttmba_Audit_dcd-03_Proche-2.doc - 5:33 [R : Elle était dans un état cr..] (123:123) (Super)**

Codes: [Etat_Arrivée_Hospitalisation]

No memos

R : Elle était dans un état critique et elle avait quitté la maison dans le même état et c’est ce qui avait fait qu’elle soit transférée.

**P 4: Bbo_HgrKttmba_Audit_dcd-03_Proche-1.doc - 4:18 [R : On lui avait envoyé à Kita..] (77:77) (Super)**

Codes: [Etat_Arrivée_Hospitalisation]

No memos

R : On lui avait envoyé à Kitatumba son état était grave et quand elle arriva à HGR Kitatumba on l’envoya au CTE

**P11: Ktw_Hgr_Audit_dcd-08_Proche-1.doc - 11:30 [R : Oui] (124:124) (Super)**

Codes: [Etat_Arrivée_Hospitalisation]

No memos

R : Oui

**P 1: Bbo_HgrKttmba_Audit_dcd-01_Proche-1.doc - 1:16 [RÉPONDANT : Il était malade et..] (50:50) (Super)**

Codes: [Etat_Arrivée_Hospitalisation]

No memos

RÉPONDANT : Il était malade et jadis on l’amenait il était traité et il retournait à la maison et lorsqu’on lui avait amené à l’hôpital on pensait qu’il allait retourner comme d’habitude parce qu’il nous disait que le ventre lui faisait mal.

**P11: Ktw_Hgr_Audit_dcd-08_Proche-1.doc - 11:37 [R/ L’enfant avait beaucoup mai..] (152:152) (Super)**

Codes: [Etat_Arrivée_Hospitalisation]

No memos

R/ L’enfant avait beaucoup maigri, trop fatigué avec la toux, il ne devait pas quitter ici jusque-là en marchant seul.

**P 2: Bbo_HgrKttmba_Audit_dcd-01_Proche-2.doc - 2:18 [R : Son Etat était gravement p..] (77:77) (Super)**

Codes: [Etat_Arrivée_Hospitalisation]

No memos

R : Son Etat était gravement par ce qu’il était d’abord dans un état de vieillesse et en plus la maladie, quand on lui avait soigné il avait récupéré un peu et comme la mort est inévitable c’est ainsi qu’il nous avait laissé.

**P 3: Bbo_HgrKttmba_Audit_dcd-01_Proche-3.doc - 3:31 [R : Il était dans un état grav..] (122:122) (Super)**

Codes: [Etat_Arrivée_Hospitalisation]

No memos

R : Il était dans un état grave très affaibli par la maladie « AMETOHA » on ne se souciait pas de lui soigner. Un malade décédait alors qu’il pouvait être guéri

**P19: Ktw_HopMtda_Audit_dcd-10_Proche-1.doc - 19:20 [R : A notre arrivée, on se lav..] (95:95) (Super)**

Codes: [Etat_Arrivée_Hospitalisation]

No memos

R : A notre arrivée, on se lavait les mains et en suite on prélevait la température et enfin on passait.

**P20: Ktw_HopMtda_Audit_dcd-11_Proche-1.doc - 20:9 [R : Elle était toujours dans s..] (43:43) (Super)**

Codes: [Etat_Arrivée_Hospitalisation]

No memos

R : Elle était toujours dans son état de paralysie mais elle collaborait, par ce que nous avons dit qu’on ne peut pas laisser le patient à la maison au moins qu’elle soit à côté des soignants

**P19: Ktw_HopMtda_Audit_dcd-10_Proche-1.doc - 19:12 [R : C’était toujours la même p..] (57:57) (Super)**

Codes: [Etat_Arrivée_Hospitalisation]

No memos

R : C’était toujours la même plainte, et c’était dans l’hôpital des anglicans pour la première fois. Après les traitements dans cet hôpital, on est rentré à la maison car sa santé s’améliorait. Après un temps elle portât encore une autre grossesse, encore elle rechuta à cause du fibrome. C’est ainsi qu’on l’amena à Matanda. Après presque deux semaines d’hospitalisation, on rentra encore à la maison.

**P18: Ktw_HopMtda_Audit_dcd-06_Proche-1.doc - 18:43 [R. Elle s’était sentie bien, g..] (170:170) (Super)**

Codes: [Fréquentation_Autre_Fosa]

No memos

R. Elle s’était sentie bien, guérie et sorti pour retourner à son domicile.

**P 5: Bbo_HgrKttmba_Audit_dcd-03_Proche-2.doc - 5:19 [R : Oui elle était transférée...] (75:75) (Super)**

Codes: [Fréquentation_Autre_Fosa]

No memos

R : Oui elle était transférée.

**P 8: Bbo_HgrKttmba_Audit_dcd-04_Proche-2.doc - 8:8 [R : Oui Avant elle était soign..] (34:34) (Super)**

Codes: [Fréquentation_Autre_Fosa]

No memos

R : Oui Avant elle était soignée à Beni et il y avait amélioration par ce qu’elle commencé à faire des travaux ménagers pendant une année. Comme il restait chez un petit frère de papa qui mourra et ils n’avaient plus à faire, ils sont rentrés à Butembo chez son beau-fils où ils résidèrent pendant un moment mais ils se trouvèrent mal à l’aise de rester dans le ménage de sa fille. Ils retournèrent chez eux et la vie devint de plus en plus chère pour trouver à manger alors que maman devrait être sous un régime alimentaire, et nous autre nous sommes séparés mais lorsqu’on trouve quelque chose on leur envoyait. Comme ils n’avaient pas de moyen la maman ne respectait plus son régime alimentaire, elle prenait tout pour l’alimentation ce qui faisait une complication pour elle. Sa santé commença à s’grava contrairement de la période qu’elle était à Beni jusqu’à ce qu’était arrivée à Kitatumba. A Kitatumba elle recevait le traitement et son état de santé s’améliora et on lui donna une prescription pour son régime alimentaire. Faute de moyen nous étions dans l’impossibilité de respecter son régime et elle rechuta et deux jours après elle décéda.

**P18: Ktw_HopMtda_Audit_dcd-06_Proche-1.doc - 18:45 [R. Oui] (178:178) (Super)**

Codes: [Fréquentation_Autre_Fosa]

No memos

R. Oui

**P 8: Bbo_HgrKttmba_Audit_dcd-04_Proche-2.doc - 8:11 [R : Elle été soigné dans la mé..] (46:46) (Super)**

Codes: [Fréquentation_Autre_Fosa]

No memos

R : Elle été soigné dans la médecine à base des plantes naturelles dans une organisation dénommée HELP et il y’avait amélioration. C’est par ce qu’il y avait plus moyen qu’elle poursuive les soins alors que là où ils résidaient le responsable était décédé

**P18: Ktw_HopMtda_Audit_dcd-06_Proche-1.doc - 18:44 [R. Deux ou trois mois environs..] (174:174) (Super)**

Codes: [Fréquentation_Autre_Fosa]

No memos

R. Deux ou trois mois environs. Vers le troisième mois, elle commençait encore à sentir mal à l’aise. C’est ainsi qu’il fallait l’amener à Kyondo où la situation s’aggrava jusqu’à être paralysée. Elle y avait suivi les soins, mais par après, les prestataires avaient décider de sa sortie en disant qu’elle sera guérie parce que sa paralysie n’était pas vraiment grave.

**P18: Ktw_HopMtda_Audit_dcd-06_Proche-1.doc - 18:42 [R. Oui c’est une Fosa] (166:166) (Super)**

Codes: [Fréquentation_Autre_Fosa]

No memos

R. Oui c’est une Fosa

**P 3: Bbo_HgrKttmba_Audit_dcd-01_Proche-3.doc - 3:8 [R : C’est au Graben dans une s..] (30:30) (Super)**

Codes: [Fréquentation_Autre_Fosa]

No memos

R : C’est au Graben dans une structure médicale

**P18: Ktw_HopMtda_Audit_dcd-06_Proche-1.doc - 18:37 [R. Oui] (147:147) (Super)**

Codes: [Fréquentation_Autre_Fosa]

No memos

R. Oui

**P18: Ktw_HopMtda_Audit_dcd-06_Proche-1.doc - 18:36 [R. Non, elle était passée par ..] (143:143) (Super)**

Codes: [Fréquentation_Autre_Fosa]

No memos

R. Non, elle était passée par là bien avant parce qu’’elle se connaissait avec les infirmiers de là.

**P18: Ktw_HopMtda_Audit_dcd-06_Proche-1.doc - 18:38 [R. C’était environ 2 mois] (150:150) (Super)**

Codes: [Fréquentation_Autre_Fosa]

No memos

R. C’était environ 2 mois

**P18: Ktw_HopMtda_Audit_dcd-06_Proche-1.doc - 18:41 [R. Oui, c’est un peu loin d’ic..] (162:162) (Super)**

Codes: [Fréquentation_Autre_Fosa]

No memos

R. Oui, c’est un peu loin d’ici. On a toujours dit que là, il y a des spécialistes.

**P 3: Bbo_HgrKttmba_Audit_dcd-01_Proche-3.doc - 3:7 [R : Il était soigné à l’Horizo..] (26:26) (Super)**

Codes: [Fréquentation_Autre_Fosa]

No memos

R : Il était soigné à l’Horizon.

**P18: Ktw_HopMtda_Audit_dcd-06_Proche-1.doc - 18:35 [R. C’était une façon d’essayer..] (139:139) (Super)**

Codes: [Fréquentation_Autre_Fosa]

No memos

R. C’était une façon d’essayer par ci, par là pour voir s’il aurait amélioration de sa santé. « TUTASHAYIRATUTYA » (Quoi faire encore ?où allons-nous encore ? Cela prouve à suffisance que le jour programmé par Dieu personne ne peut le modifier.

**P 5: Bbo_HgrKttmba_Audit_dcd-03_Proche-2.doc - 5:1 [R : C’est lorsqu’elle avait se..] (3:3) (Super)**

Codes: [Fréquentation_Autre_Fosa]

No memos

R : C’est lorsqu’elle avait senti affaibli par la maladie on avait cherché un taxi man qui lui avait amené dans une structure medicale on lui soigna mais elle avait sa maladie d’épilepsie et c’est celle qui lui dérangeait souvent.

**P 4: Bbo_HgrKttmba_Audit_dcd-03_Proche-1.doc - 4:14 [R : C’est un Poste de Santé] (61:61) (Super)**

Codes: [Fréquentation_Autre_Fosa]

No memos

R : C’est un Poste de Santé

**P 4: Bbo_HgrKttmba_Audit_dcd-03_Proche-1.doc - 4:15 [R : Oui elle avait fait les ex..] (65:65) (Super)**

Codes: [Fréquentation_Autre_Fosa]

No memos

R : Oui elle avait fait les examens et on lui dira qu’elle souffre la maladie de tomber par terre.

**P 4: Bbo_HgrKttmba_Audit_dcd-03_Proche-1.doc - 4:17 [R : Je ne me rappel plus de la..] (73:73) (Super)**

Codes: [Fréquentation_Autre_Fosa]

No memos

R : Je ne me rappel plus de la durée de son hospitalisation c’est après qu’on lui avait transférer à l’HGR Kitatumba.

**P 4: Bbo_HgrKttmba_Audit_dcd-03_Proche-1.doc - 4:13 [R : Cet hôpital s’appelle CHRI..] (57:57) (Super)**

Codes: [Fréquentation_Autre_Fosa]

No memos

R : Cet hôpital s’appelle CHRINOVITE

**P14: Ktw_Hgr_Audit_dcd-09_Proche-1.doc - 14:3 [R : Sept jours] (13:13) (Super)**

Codes: [Fréquentation_Autre_Fosa]

No memos

R : Sept jours

**P 1: Bbo_HgrKttmba_Audit_dcd-01_Proche-1.doc - 1:32 [RÉPONDANT : Comme c’était la n..] (109:109) (Super)**

Codes: [Fréquentation_Autre_Fosa]

No memos

RÉPONDANT : Comme c’était la nuit et ceux qui lui avez amené n’étaient pas informés de l’adroit ou il se soignait régulièrement.

**P14: Ktw_Hgr_Audit_dcd-09_Proche-1.doc - 14:2 [R : Nous avions fait six jours..] (9:9) (Super)**

Codes: [Fréquentation_Autre_Fosa]

No memos

R : Nous avions fait six jours.

**P16: Ktw_HopMtda_Audit_dcd-05_Proche-2.doc - 16:13 [R. Oui] (53:53) (Super)**

Codes: [Fréquentation_Autre_Fosa]

No memos

R. Oui

**P16: Ktw_HopMtda_Audit_dcd-05_Proche-2.doc - 16:12 [R. C’est tout près de l’église..] (49:49) (Super)**

Codes: [Fréquentation_Autre_Fosa]

No memos

R. C’est tout près de l’église de Kimemi

**P 3: Bbo_HgrKttmba_Audit_dcd-01_Proche-3.doc - 3:5 [R : Il était parti dans une au..] (18:18) (Super)**

Codes: [Fréquentation_Autre_Fosa]

No memos

R : Il était parti dans une autre structure ailleurs.

**P 4: Bbo_HgrKttmba_Audit_dcd-03_Proche-1.doc - 4:22 [R : Oui elle était hospitalisé..] (93:93) (Super)**

Codes: [Fréquentation_Autre_Fosa]

No memos

R : Oui elle était hospitalisée, je la visitais mais je ne sais pas que médicament qu’on lui donnait.

**P 3: Bbo_HgrKttmba_Audit_dcd-01_Proche-3.doc - 3:24 [R : Partout c’était le champs ..] (94:94) (Super)**

Codes: [Fréquentation_Autre_Fosa]

No memos

R : Partout c’était le champs d’Ebola même si on pouvait aller ailleurs on vous renvoyait vers le CTE pour la fiche du CTE

**P16: Ktw_HopMtda_Audit_dcd-05_Proche-2.doc - 16:15 [R. Je ne sais pas] (61:61) (Super)**

Codes: [Fréquentation_Autre_Fosa]

No memos

R. Je ne sais pas

**P16: Ktw_HopMtda_Audit_dcd-05_Proche-2.doc - 16:14 [R. Elle était bien soignée.] (57:57) (Super)**

Codes: [Fréquentation_Autre_Fosa]

No memos

R. Elle était bien soignée.

**P15: Ktw_HopMtda_Audit_dcd-05_Proche-1.doc - 15:22 [R. Je ne sais pas le nom de ce..] (88:88) (Super)**

Codes: [Fréquentation_Autre_Fosa]

No memos

R. Je ne sais pas le nom de cet hôpital. C’est cet hôpital qui admet les personnes qui ont les problèmes de muscles, des blessures ; C’est là où il y a le service de kinésithérapie.

**P15: Ktw_HopMtda_Audit_dcd-05_Proche-1.doc - 15:21 [R. Non, en descendant ici tout..] (84:84) (Super)**

Codes: [Fréquentation_Autre_Fosa]

No memos

R. Non, en descendant ici tout près de la banque.

**P11: Ktw_Hgr_Audit_dcd-08_Proche-1.doc - 11:9 [R : C’est là en ville sur Aven..] (37:37) (Super)**

Codes: [Fréquentation_Autre_Fosa]

No memos

R : C’est là en ville sur Avenue Makasi

**P11: Ktw_Hgr_Audit_dcd-08_Proche-1.doc - 11:10 [R : c’est aussi un grand centr..] (41:41) (Super)**

Codes: [Fréquentation_Autre_Fosa]

No memos

R : c’est aussi un grand centre où on test la tuberculose, quand j’y suis arrivé on l’a pas suivi comme je voulais.

**P15: Ktw_HopMtda_Audit_dcd-05_Proche-1.doc - 15:20 [R. C’est ici tout près, en des..] (80:80) (Super)**

Codes: [Fréquentation_Autre_Fosa]

No memos

R. C’est ici tout près, en descendant à la banque à côté de KIMEMI à coté de la banque

**P15: Ktw_HopMtda_Audit_dcd-05_Proche-1.doc - 15:19 [R. On l’a fait sortir à l’hôpi..] (76:76) (Super)**

Codes: [Fréquentation_Autre_Fosa]

No memos

R. On l’a fait sortir à l’hôpital de KITATUMBA pour l’amener à Kalugamakuba qui est un centre pour handicapé.

**P15: Ktw_HopMtda_Audit_dcd-05_Proche-1.doc - 15:13 [R. Non] (54:54) (Super)**

Codes: [Fréquentation_Autre_Fosa]

No memos

R. Non

**P 6: Bbo_HgrKttmba_Audit_dcd-03_Proche-3.doc - 6:17 [R : Oui j’y était arrivée auss..] (65:65) (Super)**

Codes: [Fréquentation_Autre_Fosa]

No memos

R : Oui j’y était arrivée aussi. Donc lors que vous arrivez à l’hôpital pour les soins il faut allait au CTE pour faire le test d’Ebola et après les examens vous retournez à l’hôpital et ce dernier peut vous recevoir.

**P 6: Bbo_HgrKttmba_Audit_dcd-03_Proche-3.doc - 6:16 [R : Non ça ne demandait pas le..] (61:61) (Super)**

Codes: [Fréquentation_Autre_Fosa]

No memos

R : Non ça ne demandait pas les signes cliniques, c’était une condition que pour recevoir un malade, il doit avoir un document du CTE même moi j’y étais passé aussi pour le prélèvement et ce sont ses résultats qui permet d’être reçu pour le traitement.

**P20: Ktw_HopMtda_Audit_dcd-11_Proche-1.doc - 20:7 [R : Nous étions à Mangrujipa c..] (35:35) (Super)**

Codes: [Fréquentation_Autre_Fosa]

No memos

R : Nous étions à Mangrujipa c’est là que son père (militaire)travaillait et nous l’avions amené à l’HGR Mangrujipa. En y arriva on me dira qu’elle n’avait pas reçu le vaccin contre la méningite. Au départ elle n’avait pas un problème c’est un jour qu’elle s’était rendu compte de la paralysie et lorsqu’on la réveilla elle dira que ses membres inferieurs ne font plus aucun mouvement. J’avais essayé de lui soulever mais c’était impossible, nous étions allés au Centre Santé proche, ce dernier nous avait référé à l’HGR. On lui préleva l’échantillon et envoyer dans un laboratoire spécialisé à Goma pour voir si ce n’est pas MAKONONGO (la méningite). Au retour le résultat était négatif. Seulement qu’on avait attendu le résultat pendant une semaine sans qu’elle bénéficie aucun traitement, c’est après qu’on nous transféra au Centre pour Handicapé à Butembo. Comme c’était dans une période de l’épidémie nous étions dans l’obligation d’aller au CTE et puis nous sommes allés à Matanda.

**P15: Ktw_HopMtda_Audit_dcd-05_Proche-1.doc - 15:29 [R. Oui, bien sur.] (115:115) (Super)**

Codes: [Fréquentation_Autre_Fosa]

No memos

R. Oui, bien sur.

**P15: Ktw_HopMtda_Audit_dcd-05_Proche-1.doc - 15:28 [R. C’est un hôpital chargée du..] (111:111) (Super)**

Codes: [Fréquentation_Autre_Fosa]

No memos

R. C’est un hôpital chargée du service de Kinésithérapie. Qui fait le massage des muscles.

**P15: Ktw_HopMtda_Audit_dcd-05_Proche-1.doc - 15:31 [R. Oui.] (122:122) (Super)**

Codes: [Fréquentation_Autre_Fosa]

No memos

R. Oui.

**P15: Ktw_HopMtda_Audit_dcd-05_Proche-1.doc - 15:30 [. Non, non. Elle était arrivée..] (118:118) (Super)**

Codes: [Fréquentation_Autre_Fosa]

No memos

. Non, non. Elle était arrivée à Matanda lorsqu’elle avait déjà le plâtre ; Maintenant une faiblesse générale du corps a commencé à apparaitre.

**P15: Ktw_HopMtda_Audit_dcd-05_Proche-1.doc - 15:27 [R. C’est à Kalungamakuha, en p..] (107:107) (Super)**

Codes: [Fréquentation_Autre_Fosa]

No memos

R. C’est à Kalungamakuha, en passant derrière l’ancienne banque. A coté de la SONAS

**P15: Ktw_HopMtda_Audit_dcd-05_Proche-1.doc - 15:24 [R. Elle était déjà presque gué..] (95:95) (Super)**

Codes: [Fréquentation_Autre_Fosa]

No memos

R. Elle était déjà presque guérie, c’est ainsi qu’on l’a transférée à Kalungamakuha pour faire des exercices (sport) dans la salle ; C’est ainsi qu’elle était tombée contre le bras qui connut la fracture. Il fallait alors mettre ce bras sous ciment (plâtre). Elle avait fait beaucoup de jours dans le lit pour cause de la blessure du bras et le problème du muscle. Elle était fatiguée jusqu'à ce qu’elle avait atteint l’anémie. Il fallait dans ce cas-là qu’elle soit transférée à l’officiel.

**P15: Ktw_HopMtda_Audit_dcd-05_Proche-1.doc - 15:23 [R. Non, c’est en descendant de..] (92:92) (Super)**

Codes: [Fréquentation_Autre_Fosa]

No memos

R. Non, c’est en descendant de l’autre coté en ville .

**P15: Ktw_HopMtda_Audit_dcd-05_Proche-1.doc - 15:26 [R. C’est là ! elle avait comme..] (103:103) (Super)**

Codes: [Fréquentation_Autre_Fosa]

No memos

R. C’est là ! elle avait commencé à avoir un problème de l’anémie à chaque moment, et recevait une transfusion sanguine qui n’a pas connu des résultats satisfaisants jusqu’à mourir. Elle était bien soignée à Matanda.

**P15: Ktw_HopMtda_Audit_dcd-05_Proche-1.doc - 15:25 [R. C’est à Matanda.] (99:99) (Super)**

Codes: [Fréquentation_Autre_Fosa]

No memos

R. C’est à Matanda.

**P 5: Bbo_HgrKttmba_Audit_dcd-03_Proche-2.doc - 5:32 [R : Je ne sais pas ce qui lui ..] (111:111) (Super)**

Codes: [Fréquentation_Autre_Fosa]

No memos

R : Je ne sais pas ce qui lui était venu dans sa tête pour insister d’aller chez lui et lui-même dira qu’on puisse l’amener à l’hôpital, toit même tu sais que parfois il faut marcher avec la volonté du malade de façon lorsqu’il vous dit qu’il part à la toilette on doit l’accompagner.

**P18: Ktw_HopMtda_Audit_dcd-06_Proche-1.doc - 18:11 [R. A Kalungamakuha, on l’a soi..] (41:41) (Super)**

Codes: [Fréquentation_Autre_Fosa]

No memos

R. A Kalungamakuha, on l’a soignée la colonne vertébrale parce qu’elle paraissait paralysée. Elle est arrivée à l’étape de mourir quand elle était déjà abattue. On a dit c’était maintenant de suite d’une tuberculose osseuse qu’elle avait succombé après avoir consommé autant de médicaments dans les différentes structures sanitaires visitées.

**P 5: Bbo_HgrKttmba_Audit_dcd-03_Proche-2.doc - 5:31 [R : Elle nous avait dit qu’ell..] (115:115) (Super)**

Codes: [Fréquentation_Autre_Fosa]

No memos

R : Elle nous avait dit qu’elle envie d’arriver chez lui.

**P 1: Bbo_HgrKttmba_Audit_dcd-01_Proche-1.doc - 1:13 [IL s’y rendait de temps en tem..] (41:41) (Super)**

Codes: [Fréquentation_Autre_Fosa]

No memos

IL s’y rendait de temps en temps lors rechute, lorsqu’il était soigné on retournait à la maison.

**P 1: Bbo_HgrKttmba_Audit_dcd-01_Proche-1.doc - 1:12 [RÉPONDANT : C’était la nuit qu..] (31:31) (Super)**

Codes: [Fréquentation_Autre_Fosa]

No memos

RÉPONDANT : C’était la nuit qu’on l’avait fait sortir de la maison et on m’avait appelé sur téléphone comme j’étais à la maison et on m’avait dit qu’on vient de l’amener à l’hôpital Kitatumba qu’on puisse s’y rencontrer. Y arriva comme c’était la nuit les prestataires avaient refusé de lui soigner, il fallait d’abord qu’il parte au CTE pour les examens du laboratoire. Il passa la nuit sans aucun traitement et le matin on l’amena au CTE où il sera observé pendant deux jours sans traitement. C’est ainsi qu’on leur demanda comment quelqu’un qui est venu étant gravement malade et ça fait deux jours sans être soigné il sera guéri comment ? on nous répondra qu’après le résultat il sera soigné et après ces deux jours on l’amena à Kitatumba où il avait fait deux jours et le troisième jour il décéda.

**P 5: Bbo_HgrKttmba_Audit_dcd-03_Proche-2.doc - 5:30 [R : Je ne sais pas, peut être ..] (119:119) (Super)**

Codes: [Fréquentation_Autre_Fosa]

No memos

R : Je ne sais pas, peut être trois jours et il n’avait pas d’amélioration et la structure décida qu’elle part dans une grande structure pour une meilleure prise en charge.

**P 5: Bbo_HgrKttmba_Audit_dcd-03_Proche-2.doc - 5:20 [R : il y avait un mouvement et..] (79:79) (Super)**

Codes: [Fréquentation_Autre_Fosa]

No memos

R : il y avait un mouvement et quand tu arrivais dans une structure sanitaire et que l’on te soigne tu ne guéris pas on devait te transférer à Kitatumba et ce dernier te transfert au CTE

**P 1: Bbo_HgrKttmba_Audit_dcd-01_Proche-1.doc - 1:11 [C’est un Hôpital de catholique..] (38:38) (Super)**

Codes: [Fréquentation_Autre_Fosa]

No memos

C’est un Hôpital de catholique 00 :04 :09

**P18: Ktw_HopMtda_Audit_dcd-06_Proche-1.doc - 18:20 [R. Elle était encore un peu bi..] (80:80) (Super)**

Codes: [Fréquentation_Autre_Fosa]

No memos

R. Elle était encore un peu bien. Elle avait seulement le problème de marcher ; Elle était partie là pour faire la consultation seulement. En y arrivant, on a dit qu’il y avait déjà écartement des muscles et elle a été obligée d’être internée.

**P11: Ktw_Hgr_Audit_dcd-08_Proche-1.doc - 11:60 [R : Oui, très minime, ils n’av..] (243:243) (Super)**

Codes: [Influence-MVE Sur les Evénements]

No memos

R : Oui, très minime, ils n’avaient pas soigné comme mon cœur voulais.

**P 9: Bbo_HopScola_Audit_dcd-07_Proche-1.doc - 9:55 [R : Il y’a de ces malades qui ..] (249:249) (Super)**

Codes: [Influence-MVE Sur les Evénements]

No memos

R : Il y’a de ces malades qui allaient toujours à l’hôpital et d’autres qui restaient à la maison.

**P15: Ktw_HopMtda_Audit_dcd-05_Proche-1.doc - 15:67 [R. C’est la propreté] (273:273) (Super)**

Codes: [Influence-MVE Sur les Evénements]

No memos

R. C’est la propreté

**P10: Ktw_Hgr_Audit_dcd-02_Proche-1.doc - 10:32 [R : A l’arrivée de l’épidémie ..] (128:128) (Super)**

Codes: [Influence-MVE Sur les Evénements]

No memos

R : A l’arrivée de l’épidémie Ebola les gens entaient dans une panique par ce que la sensibilisation n’était pas de l’avis de la population. Cette dernière était prête à accueillir, recevoir et collaborer avec l’équipe de la riposte ; mais la façon dont cette dernière avait commencé n’était pas bonne, par ce que l’équipe de riposte en rencontré le climat politique en place n’était pas bon. Où la population et les autorités locales il y avait certaines choses que la population se demandait s’il fallait continuer de leur faire confiance. Est-ce que ces gens ne sont pas quelque part ils ne savent pas ceux qui se passe ? La population vu le moyen qui était mobilisé pour Ebola, rien n’était fait du côté de la sécurité et il y avait quelques ’uns qui était venu pour politiser la question Ebola immédiatement il n’y avait plus confiance, elle n’a pas cru à l’équipe de la riposte. La population considérait l’hôpital comme un lieu de Crime (un lieu de mort) ; par ce que si tu partais à l’hôpital c’est fini pour toi. Cette conception a été captée par la population. Cette conception est quitte progressivement lorsqu’elle avait vu que même celui qui fouillait la maladie était victime toujours de celle-ci et celui qui est resté est victime de la maladie, il n’y avait plus moyen de faire autrement. C’est à ce moment qu’elle avait compris qu’il n’y avait plus de choit elle avait commencé à se faire soigner et grâce à la sensibilisation qui était intense. Pendant cette période il y avait une incertitude pour pouvoir se retirer dans la situation alors qu’on était informé, on avait peur que si je parts à l’hôpital on va m’amener au CTE, le gens voyais comme un tabou et que les soignants sont corrompus. Ce qui était l’opinion de la population.

**P13: Ktw_Hgr_Audit_dcd-08_Proche-2.doc - 13:35 [R : Non, ils ne fréquentaient ..] (145:145) (Super)**

Codes: [Influence-MVE Sur les Evénements]

No memos

R : Non, ils ne fréquentaient plus les structures ils avaient peur par ce qu’ils arrivaient à l’hôpital ils tombaient malade. Ils évitaient d’être contaminés car il y avait deux cas qui étaient malades et lorsqu’on les avait amenés à l’hôpital l’un d’eux étaient décédé après les avoir examiné on avait dit qu’ils étaient décédés d’Ebola et était contaminé à partir de la chambre où il était observé. Ces cas ont fait que les gens ne fréquentent plus la structure.

**P13: Ktw_Hgr_Audit_dcd-08_Proche-2.doc - 13:34 [R : Oui dans la structure on a..] (141:141) (Super)**

Codes: [Influence-MVE Sur les Evénements]

No memos

R : Oui dans la structure on avait enlevé les moustiquaires sur les lits des patients alors qu’il y a beaucoup de moustiques et lorsqu’on avait demandé on nous dira que ces sont les gens de la riposte qui avaient enlevé pour éviter la propagation de la MVE. Les moustiques dérangeaient et il y avait beaucoup de cas de paludisme.

**P10: Ktw_Hgr_Audit_dcd-02_Proche-1.doc - 10:22 [R : Oui, je pense qu’il n’aura..] (89:89) (Super)**

Codes: [Influence-MVE Sur les Evénements]

No memos

R : Oui, je pense qu’il n’aurait pas une grande différence par ce qu’avant cette épidémie il était encore hospitalisé ici. Bien qu’il avait une petite difficulté qui était justifié, moi-même j’avais compris et c’était bien passé.

**P13: Ktw_Hgr_Audit_dcd-08_Proche-2.doc - 13:38 [R : C’est la perte des vies hu..] (157:157) (Super)**

Codes: [Influence-MVE Sur les Evénements]

No memos

R : C’est la perte des vies humaines car il a eu beaucoup de décès.

**P13: Ktw_Hgr_Audit_dcd-08_Proche-2.doc - 13:32 [R : Non je ne pense pas, au co..] (132:132) (Super)**

Codes: [Influence-MVE Sur les Evénements]

No memos

R : Non je ne pense pas, au contraire l’épidémie a amené l’allégement de certaines tâches dans les structure qui étaient en difficulté de trouver.

**P15: Ktw_HopMtda_Audit_dcd-05_Proche-1.doc - 15:68 [Du point de vue aide ou perte ..] (277:277) (Super)**

Codes: [Influence-MVE Sur les Evénements]

No memos

Du point de vue aide ou perte ? La répondante n’ayant pas fourni des réponses à ces questions il fallait relancer en ceci : « Est-ce que l’avènement de l’Ebola a amélioré où perturber les services au sein de KITATUMBA ?

**P 9: Bbo_HopScola_Audit_dcd-07_Proche-1.doc - 9:56 [R : Ils restaient à la maison ..] (253:253) (Super)**

Codes: [Influence-MVE Sur les Evénements]

No memos

R : Ils restaient à la maison à de peur qu’on puisse les amener au CTE.

**P13: Ktw_Hgr_Audit_dcd-08_Proche-2.doc - 13:31 [R : Peut-être oui parce que ce..] (128:128) (Super)**

Codes: [Influence-MVE Sur les Evénements]

No memos

R : Peut-être oui parce que cette épidémie avait apporté la défaillance aux soignants tellement qu’ils se plaignaient qu’ils n’étaient pas rémunérés, ils traitaient avec méfiance.

**P15: Ktw_HopMtda_Audit_dcd-05_Proche-1.doc - 15:69 [R. Cela a augmenté seulement l..] (279:279) (Super)**

Codes: [Influence-MVE Sur les Evénements]

No memos

R. Cela a augmenté seulement la propreté des mains parce que tout le monde se laver

**P15: Ktw_HopMtda_Audit_dcd-05_Proche-1.doc - 15:66 [R. On forçait les gens à se la..] (269:269) (Super)**

Codes: [Influence-MVE Sur les Evénements]

No memos

R. On forçait les gens à se laver les mains.

**P 9: Bbo_HopScola_Audit_dcd-07_Proche-1.doc - 9:49 [R : Non, nous sommes créés pou..] (224:224) (Super)**

Codes: [Influence-MVE Sur les Evénements]

No memos

R : Non, nous sommes créés pour mourir un jour, peut être que son destin était ce jour-là, parce qu’on a tout fait mais ça n’a pas marcher. C’était son jour de voyage.

**P12: Ktw_Hgr_Audit_dcd-08_Proche-12.doc - 12:36 [R : Oui les soins gratuits on ..] (155:155) (Super)**

Codes: [Influence-MVE Sur les Evénements]

No memos

R : Oui les soins gratuits on ne soigne pas bien

**P20: Ktw_HopMtda_Audit_dcd-11_Proche-1.doc - 20:26 [R : avant l’arrivé d’Ebola les..] (119:119) (Super)**

Codes: [Influence-MVE Sur les Evénements]

No memos

R : avant l’arrivé d’Ebola les gens étaient malades et ils se soignaient et c’est une maladie grave on pouvait soigner toujours.

**P12: Ktw_Hgr_Audit_dcd-08_Proche-12.doc - 12:37 [R : Oui c’était la période de ..] (159:159) (Super)**

Codes: [Influence-MVE Sur les Evénements]

No memos

R : Oui c’était la période de gratuité on ne payait pas les soins. On payait seulement certains examens à peu près dix dollars

**P 9: Bbo_HopScola_Audit_dcd-07_Proche-1.doc - 9:52 [R : L’Ébola a ajouté le triage..] (236:236) (Super)**

Codes: [Influence-MVE Sur les Evénements]

No memos

R : L’Ébola a ajouté le triage parce qu’au paravent on passait directement par la réception puis à la consultation mais aujourd’hui avant d’y arriver on passe par le triage.

**P20: Ktw_HopMtda_Audit_dcd-11_Proche-1.doc - 20:27 [R : Ça pourrait arriver] (123:123) (Super)**

Codes: [Influence-MVE Sur les Evénements]

No memos

R : Ça pourrait arriver

**P14: Ktw_Hgr_Audit_dcd-09_Proche-1.doc - 14:28 [R : Je pense bien que c’est ça..] (120:120) (Super)**

Codes: [Influence-MVE Sur les Evénements]

No memos

R : Je pense bien que c’est ça, par ce que si on avait payé beaucoup d’argents les choses pouvaient marcher, je me demande si on ne paye pas les prestataires des soins lorsqu’il y a la gratuité ? S’ils savaient qu’ils auraient leurs salaire la fin de mois, ils seraient motivés, ils pouvaient soigner avec courage. Lorsqu’on le demandait un service de radiographie ou échographie, on sait que ces sont des services payables avant de les faires mais parfois on leurs demandait qu’on puisse le faire eux refusaient sans nous demander si nous somme prés avec les frais.

**P 9: Bbo_HopScola_Audit_dcd-07_Proche-1.doc - 9:53 [R : Il y’a aussi le lavage des..] (241:241) (Super)**

Codes: [Influence-MVE Sur les Evénements]

No memos

R : Il y’a aussi le lavage des mains.

**P12: Ktw_Hgr_Audit_dcd-08_Proche-12.doc - 12:35 [R : Je pense oui par ce que pe..] (151:151) (Super)**

Codes: [Influence-MVE Sur les Evénements]

No memos

R : Je pense oui par ce que pendant la période d’Ebola il y avait la gratuité, il y avait un traitement superficiel. Même si vous soyez hospitalisé vous serez déchargé sans être guéris. Mais à cause de l’épidémie beaucoup de gens sont morts. Que vous ayez Ebola ou pas les gens ne rentaient pas, ils mouraient tous.

**P 1: Bbo_HgrKttmba_Audit_dcd-01_Proche-1.doc - 1:31 [RÉPONDANT : Oui il y aurait ch..] (105:105) (Super)**

Codes: [Influence-MVE Sur les Evénements]

No memos

RÉPONDANT : Oui il y aurait changement et surtout si on pouvait l’amener dans la structure où il était habitué à être soigné il aurait guéri.

**P14: Ktw_Hgr_Audit_dcd-09_Proche-1.doc - 14:38 [L’épidémie nous a apporté la p..] (161:161) (Super)**

Codes: [Influence-MVE Sur les Evénements]

No memos

L’épidémie nous a apporté la propreté dans la communauté, et le changement du comportement par l’écoute et la mise en pratique d’une information communautaire. Par ce que nous en tant que relais nous étions mal compris lors de la sensibilisation sur la MVE. Actuellement ils nous félicitent pour cette activité de leurs conscientiser par rapport avec l’épidémie en disant que nous leurs avons aidé. Peut-être que nous serions morts, dans notre cellule on a pas connu le cas d’Ebola .

**P11: Ktw_Hgr_Audit_dcd-08_Proche-1.doc - 11:59 [R : Peut-être mon fils devrait..] (239:239) (Super)**

Codes: [Influence-MVE Sur les Evénements]

No memos

R : Peut-être mon fils devrait être en vie ; c’est ma réflexion mais les gens étaient en train de mourir toujours. Lorsqu’on a perdu on pense autrement.

**P14: Ktw_Hgr_Audit_dcd-09_Proche-1.doc - 14:37 [R : La MVE nous donné une autr..] (157:157) (Super)**

Codes: [Influence-MVE Sur les Evénements]

No memos

R : La MVE nous donné une autre connaissance par ce qu’au début on pensait que c’est de la blague, mais comme ça s’est aggravé si l’on savait. Tu sais qu’ici chez nous, on ne savait pas l’épidémie pour faire face. La population riveraine qui souffrait de l’épidémie du choléra ; un moment donné elle avait adopter un comportement des règles d’hygiène ce qui n’était pas le cas dans notre milieu. Mais ça nous a aidé parce que ça ne peut plus nous rattraper encore et rester dans l’ignorance.

**P14: Ktw_Hgr_Audit_dcd-09_Proche-1.doc - 14:39 [R : Nous leurs avons préparer ..] (165:165) (Super)**

Codes: [Influence-MVE Sur les Evénements]

No memos

R : Nous leurs avons préparer à faire face à cette épidémie en lavant les mains et avoir la propreté.

**P20: Ktw_HopMtda_Audit_dcd-11_Proche-1.doc - 20:29 [R : Le Virus Ebola a remonté l..] (131:131) (Super)**

Codes: [Influence-MVE Sur les Evénements]

No memos

R : Le Virus Ebola a remonté le niveau de la propreté au niveau de l’hôpital et nous a instauré le lavage des mains et la prise de température. Même au niveau du social c’est toujours la propriété qu’a modifié.

**P20: Ktw_HopMtda_Audit_dcd-11_Proche-1.doc - 20:30 [R : Comme je vous l’ai dit les..] (135:135) (Super)**

Codes: [Influence-MVE Sur les Evénements]

No memos

R : Comme je vous l’ai dit les gens ont commencé à laver les mains et les seaux sont éparpillé partout dans la communauté. C’est ce que nous avons vu comme changement la propriété.

**P15: Ktw_HopMtda_Audit_dcd-05_Proche-1.doc - 15:63 [R. Mourir c’est normal pour ch..] (257:257) (Super)**

Codes: [Influence-MVE Sur les Evénements]

No memos

R. Mourir c’est normal pour chaque personne si le moment est arrivé. Le soignant n’a que le rôle de soigner ; Celui dont le moment n’est pas encore arrivé, il guérira.

**P 9: Bbo_HopScola_Audit_dcd-07_Proche-1.doc - 9:54 [R : Les changements apportés p..] (245:245) (Super)**

Codes: [Influence-MVE Sur les Evénements]

No memos

R : Les changements apportés par l’Ebola sont : le lavage des mains, le fait de ne pas serrer la main lors d’une salutation aux personnes malades, surtout la propreté.

**P17: Ktw_HopMtda_Audit_dcd-05_Proche-3.doc - 17:35 [R. Je n’avais jamais amené un ..] (149:149) (Super)**

Codes: [Influence-MVE Sur les Evénements]

No memos

R. Je n’avais jamais amené un malade à Kitatumba ; Elle était la première que j’ai internée dans cette structure de santé.

**P17: Ktw_HopMtda_Audit_dcd-05_Proche-3.doc - 17:36 [Elle répond alors en disant qu..] (153:153) (Super)**

Codes: [Influence-MVE Sur les Evénements]

No memos

Elle répond alors en disant que l’Ebola a fait vraiment succomber beaucoup des personnes dans le milieu. Et Dieu nous a aidé pour que cela finisse ; et cela est fini. Mais nous regrettons qu’une notre épidémie vienne

**P19: Ktw_HopMtda_Audit_dcd-10_Proche-1.doc - 19:27 [R : Au point de vue organisati..] (126:126) (Super)**

Codes: [Influence-MVE Sur les Evénements]

No memos

R : Au point de vue organisation l’épidémie a apporté un développement surtout au niveau de la propreté de l’hôpital, Matanda est devenu propre, il y’a eu un grand changement du point de vu hygiène.

**P 6: Bbo_HgrKttmba_Audit_dcd-03_Proche-3.doc - 6:48 [R : Oui parce que s’il n’y ava..] (189:189) (Super)**

Codes: [Influence-MVE Sur les Evénements]

No memos

R : Oui parce que s’il n’y avait pas l’épidémie elle tomberait malade et aller aux soins spécialisés. A cause de la peur elle était obligée d’aller au poste de santé, De deux le temps qu’elle avait fait au CTE sans traitement lui avais affaibli davantage. Si on l’avait transféré à FEPSI comme c’est une structure qui s’occupe du VIH peut être qu’elle aurait reçu un traitement approprié et retrouver sa santé, la période d’Ebola nous avait mis mal alaise.

**P17: Ktw_HopMtda_Audit_dcd-05_Proche-3.doc - 17:28 [R. Oui, elle allait mourir tou..] (121:121) (Super)**

Codes: [Influence-MVE Sur les Evénements]

No memos

R. Oui, elle allait mourir toujours.

**P 5: Bbo_HgrKttmba_Audit_dcd-03_Proche-2.doc - 5:64 [R : Par ce qu’ils nous soignai..] (246:246) (Super)**

Codes: [Influence-MVE Sur les Evénements]

No memos

R : Par ce qu’ils nous soignaient bien.

**P 5: Bbo_HgrKttmba_Audit_dcd-03_Proche-2.doc - 5:63 [R : Il y avait les soins gratu..] (242:242) (Super)**

Codes: [Influence-MVE Sur les Evénements]

No memos

R : Il y avait les soins gratuits et ils nous avaient bien soignés ; et il n’y avait pas de cas de décès à l’hôpital. Tous les cas de décès provenaient de la CTE.

**P17: Ktw_HopMtda_Audit_dcd-05_Proche-3.doc - 17:39 [R. Cela, je ne connais pas.] (165:165) (Super)**

Codes: [Influence-MVE Sur les Evénements]

No memos

R. Cela, je ne connais pas.

**P17: Ktw_HopMtda_Audit_dcd-05_Proche-3.doc - 17:37 [R. Là je ne sais pas répondre] (157:157) (Super)**

Codes: [Influence-MVE Sur les Evénements]

No memos

R. Là je ne sais pas répondre

**P17: Ktw_HopMtda_Audit_dcd-05_Proche-3.doc - 17:38 [R. Pour moi aucun bienfait, pa..] (161:161) (Super)**

Codes: [Influence-MVE Sur les Evénements]

No memos

R. Pour moi aucun bienfait, par ce que toute ma famille était fini : ma fille, ma sœur, dans leur parcelle on a fermé la maison. De l’autre côté, on a aussi fermé des maisons.

**P 7: Bbo_HgrKttmba_Audit_dcd-04_Proche-1.doc - 7:43 [R : Non il n’y avait pas de la..] (179:179) (Super)**

Codes: [Influence-MVE Sur les Evénements]

No memos

R : Non il n’y avait pas de lamentation et il n’avait pas le service de tri quand vous arrivez on donnait la fiche et immédiatement vous partez à la consultation.

**P 7: Bbo_HgrKttmba_Audit_dcd-04_Proche-1.doc - 7:41 [R : Oui car la maladie a sa fi..] (171:171) (Super)**

Codes: [Influence-MVE Sur les Evénements]

No memos

R : Oui car la maladie a sa fin.

**P 7: Bbo_HgrKttmba_Audit_dcd-04_Proche-1.doc - 7:44 [R : C’est le retard au service..] (183:183) (Super)**

Codes: [Influence-MVE Sur les Evénements]

No memos

R : C’est le retard au service de tri ou on doit attendre pour faire les examens d’Ebola.

**P 7: Bbo_HgrKttmba_Audit_dcd-04_Proche-1.doc - 7:46 [R : Personnellement je dirais ..] (191:191) (Super)**

Codes: [Influence-MVE Sur les Evénements]

No memos

R : Personnellement je dirais qu’il n’y avait plus la charité ou la fraternité pourquoi ? Lorsqu’il y avait décès dans la communauté on pouvait plus assister au deuil et aux obsèques et même si c’est un membre de famille.

**P 7: Bbo_HgrKttmba_Audit_dcd-04_Proche-1.doc - 7:45 [R : Au niveau de la communauté..] (187:187) (Super)**

Codes: [Influence-MVE Sur les Evénements]

No memos

R : Au niveau de la communauté les gens avaient peur et si on tombait malade on parvenait de se cacher pour ne pas aller à l’hôpital. Mais moi je partais à l’hôpital et lorsque j’arrivais au tris je l’avais les mains et passer sans problème.

**P16: Ktw_HopMtda_Audit_dcd-05_Proche-2.doc - 16:37 [R. Elle était bien soignée. Ma..] (184:184) (Super)**

Codes: [Influence-MVE Sur les Evénements]

No memos

R. Elle était bien soignée. Mais lorsque le créateur a décidé sur le dernier jour de quelqu’un, il doit nécessairement mourir. Même s’il y avait Ebola elle était bien suivie et bien prise en charge.

**P16: Ktw_HopMtda_Audit_dcd-05_Proche-2.doc - 16:42 [R. L’Ebola a apporté la propre..] (213:213) (Super)**

Codes: [Influence-MVE Sur les Evénements]

No memos

R. L’Ebola a apporté la propreté. Il y avait jadis des hôpitaux sales mais avec l’avènement de l’Ebola, tous les hôpitaux sont devenus propres.

**P19: Ktw_HopMtda_Audit_dcd-10_Proche-1.doc - 19:21 [R : Non.] (99:99) (Super)**

Codes: [Influence-MVE Sur les Evénements]

No memos

R : Non.

**P 7: Bbo_HgrKttmba_Audit_dcd-04_Proche-1.doc - 7:40 [R : Non je ne pense pas que le..] (167:167) (Super)**

Codes: [Influence-MVE Sur les Evénements]

No memos

R : Non je ne pense pas que les choses pourraient se passer autrement parce que nous devons comprendre les choses, lorsque on vit avec une pathologie quelconque ; ce n’est pas l’arrivée d’Ebola qui doit mettre fin à ta pathologie.

**P19: Ktw_HopMtda_Audit_dcd-10_Proche-1.doc - 19:22 [R : Parce que c’était une autr..] (103:103) (Super)**

Codes: [Influence-MVE Sur les Evénements]

No memos

R : Parce que c’était une autre maladie, et elle ne présentait pas les signes de la maladie à virus d’Ebola, et on nous avait vite accueilli c’est pourquoi on n’avait même pas traine au triage.

**P 5: Bbo_HgrKttmba_Audit_dcd-03_Proche-2.doc - 5:62 [R : Non, c’est au CTE ou il y ..] (238:238) (Super)**

Codes: [Influence-MVE Sur les Evénements]

No memos

R : Non, c’est au CTE ou il y avait une information qu’on tuait les gens mais à Kitatumba non. Il n’y a pas de problème.

**P18: Ktw_HopMtda_Audit_dcd-06_Proche-1.doc - 18:64 [R. Non] (267:267) (Super)**

Codes: [Influence-MVE Sur les Evénements]

No memos

R. Non

**P18: Ktw_HopMtda_Audit_dcd-06_Proche-1.doc - 18:67 [R. Pour l’accueil, il y a eu u..] (279:279) (Super)**

Codes: [Influence-MVE Sur les Evénements]

No memos

R. Pour l’accueil, il y a eu un changement parce qu’’avant épidémie, les infirmiers couraient pour accueillir les malades et venaient secourir ceux-là qui l’amènent malgré le moyen utilisé, pendant l’épidémie cela a changé parce qu’il ne faut pas se toucher et les soignants traitaient selon les méthodes reçues dans la formation obtenue pour l’épidémie.

**P19: Ktw_HopMtda_Audit_dcd-10_Proche-1.doc - 19:28 [R : C’était devenu le lieu de ..] (134:134) (Super)**

Codes: [Influence-MVE Sur les Evénements]

No memos

R : C’était devenu le lieu de refuge des malades, ils s’y rendaient pour les soins par ce que la structure voulait s’abstenir de la riposte, c’est un peu tard qu’ils avaient accepté l’installation de la riposte. Et quand cette dernière est installer, les malades n’y arrivaient plus, tout le monde allait vers les centres de santé et les dispensaires.

**P18: Ktw_HopMtda_Audit_dcd-06_Proche-1.doc - 18:62 [R. Sa situation serait bien. M..] (259:259) (Super)**

Codes: [Influence-MVE Sur les Evénements]

No memos

R. Sa situation serait bien. Mais sa maladie n’était pas à craindre pour assimilation à Ebola, il suffisait seulement pour partir prendre les soins.

**P18: Ktw_HopMtda_Audit_dcd-06_Proche-1.doc - 18:63 [R. Oui, cela pouvait arriver] (263:263) (Super)**

Codes: [Influence-MVE Sur les Evénements]

No memos

R. Oui, cela pouvait arriver

**P18: Ktw_HopMtda_Audit_dcd-06_Proche-1.doc - 18:71 [R. Oui ; Certains y allaient m..] (295:295) (Super)**

Codes: [Influence-MVE Sur les Evénements]

No memos

R. Oui ; Certains y allaient mais d’autres qui sont ignorants refusaient catégoriquement de s’y rendre afin de ne pas être assimilés aux malades d’Ebola et être transféré. C’est ainsi que l’on pouvait souffrir de la maladie en restant à la maison, ce qui est à qualifier comme ignorance.

**P18: Ktw_HopMtda_Audit_dcd-06_Proche-1.doc - 18:72 [R. Au Centre de traitement d’E..] (299:299) (Super)**

Codes: [Influence-MVE Sur les Evénements]

No memos

R. Au Centre de traitement d’EBOLA (CTE). Il y a eu autant des rumeurs à cause du CTE

**P18: Ktw_HopMtda_Audit_dcd-06_Proche-1.doc - 18:70 [R. Il y avait plus recours aux..] (291:291) (Super)**

Codes: [Influence-MVE Sur les Evénements]

No memos

R. Il y avait plus recours aux soins les portes étaient fermées parce que pour accéder au passage c’était difficile. Un chemin passant par l’hôpital pour accéder à notre domicile était bloqué.

**P18: Ktw_HopMtda_Audit_dcd-06_Proche-1.doc - 18:68 [R. Je manque quoi dire par rap..] (283:283) (Super)**

Codes: [Influence-MVE Sur les Evénements]

No memos

R. Je manque quoi dire par rapport à cette question.

**P18: Ktw_HopMtda_Audit_dcd-06_Proche-1.doc - 18:69 [R. C’est une sorte de division..] (287:287) (Super)**

Codes: [Influence-MVE Sur les Evénements]

No memos

R. C’est une sorte de division entre les malades et le personnel soignant car celui-ci cherchait à tout prix comment se protéger lors de soins. Le malade se lamentait avec autant des questions pour l’accueil qu’affichait cette équipe soignante mais actuellement comme la maladie est en train de disparaitre, on assiste au retour progressif de la situation précédente.

**P 2: Bbo_HgrKttmba_Audit_dcd-01_Proche-2.doc - 2:31 [R : Par ce que dans cette péri..] (129:129) (Super)**

Codes: [Influence-MVE Sur les Evénements]

No memos

R : Par ce que dans cette période, les personnels soignants ne souciaient plus des patients. Peut-être que s’il tombait malade et que les soignants s’occupaient de lui il allait guérir ou soit qu’il n’y avait pas l’Epidémie il pourrait récupérer et vivre encore une année.

**P 4: Bbo_HgrKttmba_Audit_dcd-03_Proche-1.doc - 4:56 [R : Ils nous ont apporté l’emb..] (232:232) (Super)**

Codes: [Influence-MVE Sur les Evénements]

No memos

R : Ils nous ont apporté l’embouteillage.

**P 2: Bbo_HgrKttmba_Audit_dcd-01_Proche-2.doc - 2:30 [R : Oui les choses pourraient ..] (125:125) (Super)**

Codes: [Influence-MVE Sur les Evénements]

No memos

R : Oui les choses pourraient pu se passer bien

**P 5: Bbo_HgrKttmba_Audit_dcd-03_Proche-2.doc - 5:61 [R : Ils avaient fait ce qu’ils..] (234:234) (Super)**

Codes: [Influence-MVE Sur les Evénements]

No memos

R : Ils avaient fait ce qu’ils pouvaient mais ça n’a pas tenu Je n’ai **jamais** entendu un mauvais rapport sur eux.

**P 5: Bbo_HgrKttmba_Audit_dcd-03_Proche-2.doc - 5:60 [R : La façon dont son état éta..] (230:230) (Super)**

Codes: [Influence-MVE Sur les Evénements]

No memos

R : La façon dont son état était, l’amaigrissement du corps et même on lui disait d’aller à l’hôpital mais elle refusait. Elle même avait vu que son temps s’approchait. Le fait de manquer le sang aussi montre que c’était son moment pour dire que sans Ebola elle devrait mourir, même l’épilepsie de chaque jour ça pouvait épuiser le sang.

**P19: Ktw_HopMtda_Audit_dcd-10_Proche-1.doc - 19:35 [R : Il y’avait des problèmes a..] (166:166) (Super)**

Codes: [Influence-MVE Sur les Evénements]

No memos

R : Il y’avait des problèmes au niveaux des familles : par exemple nous qui avons les mamans qui sont des relais communautaires, quand elle remarque qu’il a un membre de famille ou un voisin qui est malade et qu’elle appelle l’équipe de la riposte pour lui récupérer et l’amener pour le traitement, les gens restaient en conflits avec cette femme, surtout si la personne malade mourrait. Par exemple notre voisin qui était mort, et sa famille avait dit que c’est nous qui sommes à la base de la mort de leur frère. C’était grave, de manière qu’on disait qu’après l’épidémie, ça sera nous les victimes.

**P 3: Bbo_HgrKttmba_Audit_dcd-01_Proche-3.doc - 3:49 [R : Oui s’il n’y avait pas Ebo..] (194:194) (Super)**

Codes: [Influence-MVE Sur les Evénements]

No memos

R : Oui s’il n’y avait pas Ebola, il pouvait encore vivre quelques mois mais quand tu parts à l’hôpital et on vous dit d’attendre jusqu’à ce que le résultat vient du CTE pour qu’on traite le malade et la maladie continue de s’aggraver. Si le soignant s’occupe de vous immédiatement on guérit

**P 4: Bbo_HgrKttmba_Audit_dcd-03_Proche-1.doc - 4:52 [] (216:216) (Super)**

Codes: [Influence-MVE Sur les Evénements]

No memos

**P 4: Bbo_HgrKttmba_Audit_dcd-03_Proche-1.doc - 4:55 [R : Ils nous ont apporté les m..] (228:228) (Super)**

Codes: [Influence-MVE Sur les Evénements]

No memos

R : Ils nous ont apporté les matériaux de l’hygiène

**P 4: Bbo_HgrKttmba_Audit_dcd-03_Proche-1.doc - 4:53 [R : S’il y avait continuité de..] (220:220) (Super)**

Codes: [Influence-MVE Sur les Evénements]

No memos

R : S’il y avait continuité de soins au CTE peut être qu’il y aurait un changement

**P 8: Bbo_HgrKttmba_Audit_dcd-04_Proche-2.doc - 8:31 [R : Oui par ce que selon moi, ..] (130:130) (Super)**

Codes: [Influence-MVE Sur les Evénements]

No memos

R : Oui par ce que selon moi, l’arrivée d’Ebola a perturbé tout. Toute personne qui était sortie de Itave (CTE), tout le monde mourrait. Aucune personne qui était guérie c’est ce que j’avais constaté. Et cela avait créer une psychose au niveau de la communauté.

**P 8: Bbo_HgrKttmba_Audit_dcd-04_Proche-2.doc - 8:30 [R : Ils arrivaient et ils deva..] (126:126) (Super)**

Codes: [Influence-MVE Sur les Evénements]

No memos

R : Ils arrivaient et ils devaient passer par Itave le lieu de Centre de Traitement Ebola CTE et ils y passaient deux jours puis retourner à l’hôpital. On vous amène dans les soins intensifs et après vous pouvez retourner dans la salle pour la continuité des soins.

**P19: Ktw_HopMtda_Audit_dcd-10_Proche-1.doc - 19:25 [R : A Matanda c’est une struct..] (117:117) (Super)**

Codes: [Influence-MVE Sur les Evénements]

No memos

R : A Matanda c’est une structure des religieux qui sont très rigoureux, mais aussi leurs factures sont très élevées. Elle est plus chère que les autres structures de la place. Cette structure est gérée par les Sœurs et ces dernières sont catégorique envers leurs biens.

**P 8: Bbo_HgrKttmba_Audit_dcd-04_Proche-2.doc - 8:37 [R : A cette époque je n’étais ..] (154:154) (Super)**

Codes: [Influence-MVE Sur les Evénements]

No memos

R : A cette époque je n’étais pas ici mais là où j’étais les gens n’acceptaient pas d’aller vers les structures sanitaires pour qu’on ne change pas la pathologie alors qu’on pouvait bien vous prendre en charge et guérir mais lorsque la pathologie change en Ebola il y a une petite chance de survivre. Certains préféraient rester à la maison et parfois on pouvait mourir à la maison sans aller se faire soigner au Centre ou à l’hôpital. Nombreux sont mort et d’autres sont restés des orphelins. C’est ce qui a fait que je dise que la MVE était une maladie politisée même le démon travail

**P 8: Bbo_HgrKttmba_Audit_dcd-04_Proche-2.doc - 8:29 [R : La propriété dans les stru..] (122:122) (Super)**

Codes: [Influence-MVE Sur les Evénements]

No memos

R : La propriété dans les structures c’est ce que j’ai vu.

**P 8: Bbo_HgrKttmba_Audit_dcd-04_Proche-2.doc - 8:26 [R : Les gens arrivaient, ils é..] (110:110) (Super)**

Codes: [Influence-MVE Sur les Evénements]

No memos

R : Les gens arrivaient, ils étaient reçus au triage moi j’étais dans une chambrette pour la prises de la température si la fièvre est très élevée on appelait l’ambulance et on vous amène au CTE pour les examens.

**P 8: Bbo_HgrKttmba_Audit_dcd-04_Proche-2.doc - 8:25 [R : Je ne pas de réponse] (106:106) (Super)**

Codes: [Influence-MVE Sur les Evénements]

No memos

R : Je ne pas de réponse

**P 8: Bbo_HgrKttmba_Audit_dcd-04_Proche-2.doc - 8:28 [R : Oui il y a eu des changeme..] (118:118) (Super)**

Codes: [Influence-MVE Sur les Evénements]

No memos

R : Oui il y a eu des changements.

**P 8: Bbo_HgrKttmba_Audit_dcd-04_Proche-2.doc - 8:27 [R : La prise en charge est bon..] (114:114) (Super)**

Codes: [Influence-MVE Sur les Evénements]

No memos

R : La prise en charge est bonne.

**P 7: Bbo_HgrKttmba_Audit_dcd-04_Proche-1.doc - 7:47 [R : L’hôpital soignait comme d..] (195:195) (Super)**

Codes: [Influence-MVE Sur les Evénements]

No memos

R : L’hôpital soignait comme d’habitude sauf que la population avait peur d’y fréquenter à cause d’Ebola. Parce qu’on n’avait pas accepté le mot Ebola.

**P 1: Bbo_HgrKttmba_Audit_dcd-01_Proche-1.doc - 1:34 [RÉPONDANT : Oui parce que si v..] (117:117) (Super)**

Codes: [Influence-MVE Sur les Evénements]

No memos

RÉPONDANT : Oui parce que si vous amenez un malade à l’hôpital et qu’il fasse deux sans recevoir les médicaments alors qu’il était malade il va mourir. Mais s’il était soigné immédiatement il allait guérir par ce qu’on dit qu’il y a un malade qui peut guérir même dans 30min.

**P11: Ktw_Hgr_Audit_dcd-08_Proche-1.doc - 11:58 [R : Ce n’est pas bon, par ce q..] (235:235) (Super)**

Codes: [Investigation_Ebola]

No memos

R : Ce n’est pas bon, par ce que jadis on en avait pas et on ne mourrait pas. Ce sont les histoires d’Ebola qui ont amené tout ça, quand on arrivait à l’hôpital on y trouvait l’urgence en premier lieu où les soignants s’occupaient de vous et c’est après qu’ils pouvaient vous trouver une chambre.

**P 5: Bbo_HgrKttmba_Audit_dcd-03_Proche-2.doc - 5:37 [R : Elle avait fait environs q..] (139:139) (Super)**

Codes: [Investigation_Ebola]

No memos

R : Elle avait fait environs quatre jours et le lendemain on lui renvoya à Kitatumba

**P11: Ktw_Hgr_Audit_dcd-08_Proche-1.doc - 11:56 [R/Là ils accueillent bien, dès..] (227:227) (Super)**

Codes: [Investigation_Ebola]

No memos

R/Là ils accueillent bien, dès que vous entrez à l’intérieur après vous avoir donné un lit vous allez rester là entrain attendre les médicaments et on peut y mourir alors qu’on est avec la foi que si on donne le médicament ça va soulager les douleurs on attend, on attend.

**P11: Ktw_Hgr_Audit_dcd-08_Proche-1.doc - 11:57 [R : c’est pour vérifier les te..] (231:231) (Super)**

Codes: [Investigation_Ebola]

No memos

R : c’est pour vérifier les températures des gens c’est ce que je pense.

**P 5: Bbo_HgrKttmba_Audit_dcd-03_Proche-2.doc - 5:36 [R : Je ne sais pas par ce que ..] (135:135) (Super)**

Codes: [Investigation_Ebola]

No memos

R : Je ne sais pas par ce que je n’y étais pas partie mais dit-on qu’on lui soignait.

**P18: Ktw_HopMtda_Audit_dcd-06_Proche-1.doc - 18:22 [R. Il n’y avait pas de grave. ..] (88:88) (Super)**

Codes: [Investigation_Ebola]

No memos

R. Il n’y avait pas de grave. On se lavait seulement les mains, en y arrivant.

**P18: Ktw_HopMtda_Audit_dcd-06_Proche-1.doc - 18:23 [R. Non] (92:92) (Super)**

Codes: [Investigation_Ebola]

No memos

R. Non

**P 5: Bbo_HgrKttmba_Audit_dcd-03_Proche-2.doc - 5:35 [R : Je ne sais pas, elle était..] (131:131) (Super)**

Codes: [Investigation_Ebola]

No memos

R : Je ne sais pas, elle était partie au soins.

**P 1: Bbo_HgrKttmba_Audit_dcd-01_Proche-1.doc - 1:33 [RÉPONDANT : A l’Horizon eux pr..] (113:113) (Super)**

Codes: [Investigation_Ebola]

No memos

RÉPONDANT : A l’Horizon eux prélèvent et amènent les échantillons au CTE sans faire déplacer les malades, contrairement de Kitatumba qui amène les malades au CTE.

**P 5: Bbo_HgrKttmba_Audit_dcd-03_Proche-2.doc - 5:59 [R : non je ne l’avais pas vu p..] (226:226) (Super)**

Codes: [Investigation_Ebola]

No memos

R : non je ne l’avais pas vu par ce qu’on était venu nous prendre à la maison qu’elle était déjà décédée et lorsqu’on y était arrivé on refusa que nous pourrons attendre le corps à la maison.

**P14: Ktw_Hgr_Audit_dcd-09_Proche-1.doc - 14:10 [R : Nous avions gardé ceux qu’..] (41:41) (Super)**

Codes: [Investigation_Ebola]

No memos

R : Nous avions gardé ceux qu’on avait donné pour la première fois et ces sont les mêmes que nous avions montré. Mais moi je n’ai pas un bon souvenir sur Katwa alors qu’il était mon hôpital.

**P20: Ktw_HopMtda_Audit_dcd-11_Proche-1.doc - 20:25 [R : Je ne vois pas son inconvé..] (115:115) (Super)**

Codes: [Investigation_Ebola]

No memos

R : Je ne vois pas son inconvénient, c’est un service qui est mise en place pour contrôler les personnes. Trier ceux qui peuvent avoir la MVE et c’est qui n’ont pas.

**P15: Ktw_HopMtda_Audit_dcd-05_Proche-1.doc - 15:56 [R. Elle était déjà dans le str..] (228:228) (Super)**

Codes: [Investigation_Ebola]

No memos

R. Elle était déjà dans le structure.

**P17: Ktw_HopMtda_Audit_dcd-05_Proche-3.doc - 17:31 [R. Ce service n’a pas d’inconv..] (133:133) (Super)**

Codes: [Investigation_Ebola]

No memos

R. Ce service n’a pas d’inconvénien

**P 9: Bbo_HopScola_Audit_dcd-07_Proche-1.doc - 9:45 [R : Oui et ça continue jusqu’à..] (207:207) (Super)**

Codes: [Investigation_Ebola]

No memos

R : Oui et ça continue jusqu’à maintenant.

**P15: Ktw_HopMtda_Audit_dcd-05_Proche-1.doc - 15:60 [R. Non] (248:248) (Super)**

Codes: [Investigation_Ebola]

No memos

R. Non

**P15: Ktw_HopMtda_Audit_dcd-05_Proche-1.doc - 15:61 [R. Oui.] (251:251) (Super)**

Codes: [Investigation_Ebola]

No memos

R. Oui.

**P15: Ktw_HopMtda_Audit_dcd-05_Proche-1.doc - 15:59 [R. Je suis arrivée dans la str..] (244:244) (Super)**

Codes: [Investigation_Ebola]

No memos

R. Je suis arrivée dans la structure d’accueil quand elle était déjà internée.

**P15: Ktw_HopMtda_Audit_dcd-05_Proche-1.doc - 15:57 [R. Oui, elle était passée par ..] (232:232) (Super)**

Codes: [Investigation_Ebola]

No memos

R. Oui, elle était passée par là.

**P15: Ktw_HopMtda_Audit_dcd-05_Proche-1.doc - 15:58 [R. Non c’est quoi ?] (240:240) (Super)**

Codes: [Investigation_Ebola]

No memos

R. Non c’est quoi ?

**P14: Ktw_Hgr_Audit_dcd-09_Proche-1.doc - 14:35 [R : IFRO on travaillait avec e..] (148:148) (Super)**

Codes: [Investigation_Ebola]

No memos

R : IFRO on travaillait avec eux ici, ils étaient les gens de suift, nous étions dans l’hygiène et assainissement en 2015 2016 comme je suis relais communautaire d’ici.

**P 4: Bbo_HgrKttmba_Audit_dcd-03_Proche-1.doc - 4:21 [R : Non elle n’avait pas reçu ..] (89:89) (Super)**

Codes: [Investigation_Ebola]

No memos

R : Non elle n’avait pas reçu aucun traitement et son état de santé se détériorait davantage.

**P18: Ktw_HopMtda_Audit_dcd-06_Proche-1.doc - 18:59 [R. C’est un bon service dans c..] (247:247) (Super)**

Codes: [Investigation_Ebola]

No memos

R. C’est un bon service dans cette entité, c’est un service qui nous a beaucoup aidé même si les gens murmuraient car il nous donnait de l’ordre.

**P18: Ktw_HopMtda_Audit_dcd-06_Proche-1.doc - 18:58 [R. Moi, je n’avais jamais eu d..] (243:243) (Super)**

Codes: [Investigation_Ebola]

No memos

R. Moi, je n’avais jamais eu de fièvre. Je passais seulement mais on nous disait que quelqu’un qui a une température élevée pouvait être isolé et avoir un petit repos après il pourra entrer.

**P18: Ktw_HopMtda_Audit_dcd-06_Proche-1.doc - 18:56 [R. Oui, ce service existe] (235:235) (Super)**

Codes: [Investigation_Ebola]

No memos

R. Oui, ce service existe

**P18: Ktw_HopMtda_Audit_dcd-06_Proche-1.doc - 18:57 [R. Il est chargé de lavage des..] (239:239) (Super)**

Codes: [Investigation_Ebola]

No memos

R. Il est chargé de lavage des mains à l’arrivé, le prélèvement de leurs températures pour de dénicher ceux qui peuvent avoir la fièvre élevée.

**P20: Ktw_HopMtda_Audit_dcd-11_Proche-1.doc - 20:21 [R : Nous étions venus avec l’a..] (95:95) (Super)**

Codes: [Investigation_Ebola]

No memos

R : Nous étions venus avec l’ambulance du CTE, et on l’avait fait entrer à la réception

**P 4: Bbo_HgrKttmba_Audit_dcd-03_Proche-1.doc - 4:23 [R : Elle était reçue dans une ..] (97:97) (Super)**

Codes: [Investigation_Ebola]

No memos

R : Elle était reçue dans une chambre et un jour après on l’amena au CTE

**P12: Ktw_Hgr_Audit_dcd-08_Proche-12.doc - 12:34 [R : Je n’avais pas vu son impo..] (147:147) (Super)**

Codes: [Investigation_Ebola]

No memos

R : Je n’avais pas vu son importance

**P18: Ktw_HopMtda_Audit_dcd-06_Proche-1.doc - 18:60 [R. Il n’y en a pas] (251:251) (Super)**

Codes: [Investigation_Ebola]

No memos

R. Il n’y en a pas

**P18: Ktw_HopMtda_Audit_dcd-06_Proche-1.doc - 18:61 [R. De ma part, pas de méfaits...] (255:255) (Super)**

Codes: [Investigation_Ebola]

No memos

R. De ma part, pas de méfaits. Mais certains disaient que c’était un mauvais service parce qu’on commence à vérifier l’état de santé de quelqu’un moyennant le thermo flash avant de visiter un malade à l’hôpital. Vraiment c’était bon parce que cela facilitait de laver les mains chaque fois à l’hôpital mais aussi mesurer ma température afin de savoir mon état de santé. Je n’ai pas vu ses méfaits ! L’épidémie nous défendus de se saluer avec la main et ce thermo flash permettait de connaitre la température de quelqu’un sans le toucher.

**P 4: Bbo_HgrKttmba_Audit_dcd-03_Proche-1.doc - 4:20 [R : Non je n’étais pas là ; el..] (85:85) (Super)**

Codes: [Investigation_Ebola]

No memos

R : Non je n’étais pas là ; elle avait fait deux jours au CTE.

**P20: Ktw_HopMtda_Audit_dcd-11_Proche-1.doc - 20:22 [R : On ne peut pas entrer sans..] (99:99) (Super)**

Codes: [Investigation_Ebola]

No memos

R : On ne peut pas entrer sans laver les mains et comme le papa était déjà là on y arrivait souvent et on y passait pour la prise de température et le lavage des mains.

**P14: Ktw_Hgr_Audit_dcd-09_Proche-1.doc - 14:31 [R : Non, on n’y était pas pass..] (132:132) (Super)**

Codes: [Investigation_Ebola]

No memos

R : Non, on n’y était pas passé par là.

**P14: Ktw_Hgr_Audit_dcd-09_Proche-1.doc - 14:32 [R : Le triage je le connais pa..] (136:136) (Super)**

Codes: [Investigation_Ebola]

No memos

R : Le triage je le connais par rapport à ceux qui y allaient comme par exemple la sœur-là c’était bon. Elle avait apprécié la façon dont les malades étaient reçus et d’ailleurs j’avais remercié les gens de la CTE par rapport à leurs accueils et il y avait un changement chez le malade lorsque nous étions partis à Katwa.

**P14: Ktw_Hgr_Audit_dcd-09_Proche-1.doc - 14:34 [R : Avec IFRO] (144:144) (Super)**

Codes: [Investigation_Ebola]

No memos

R : Avec IFRO

**P14: Ktw_Hgr_Audit_dcd-09_Proche-1.doc - 14:33 [R : je ne trouvais pas les inc..] (140:140) (Super)**

Codes: [Investigation_Ebola]

No memos

R : je ne trouvais pas les inconvénients du triage, là je mens si je dis quelques choses. Parmi ceux dont j’avais visités personne qui se plaignait du quelque chose au triage. Même mon enfant était parti au CTE aussi je n’avais pas vu d’inconvénient. Le lavage des mains nous qui sommes nés à Kyondo, c’est une habitude pour nous de laver les mains. Et dans notre milieu nous avons eu la grâce de travailler avec l’ONG IFRO en 2015 dans un projet hygiène et assainissement, chez nous il n’y avait pas aucun cas de MVE par ce qu’on avait placé le petit bidon d’eau aux toilettes pour lavage des mains. En quittant la toilette on piétinait un stick d’arbre et l’eau sortait et on l’avait les mains avec la cendre. Nous sommes habitué à cela.

**P 4: Bbo_HgrKttmba_Audit_dcd-03_Proche-1.doc - 4:51 [R : J’entends parler mais je n..] (212:212) (Super)**

Codes: [Investigation_Ebola]

No memos

R : J’entends parler mais je n’ai jamais été là pour l’expérimenter.

**P 4: Bbo_HgrKttmba_Audit_dcd-03_Proche-1.doc - 4:50 [R : J’étais venu et elle était..] (208:208) (Super)**

Codes: [Investigation_Ebola]

No memos

R : J’étais venu et elle était déjà dans la chambre, je ne sais pas s’elle y était passée.

**P15: Ktw_HopMtda_Audit_dcd-05_Proche-1.doc - 15:62 [R. Je ne sais pas.] (254:254) (Super)**

Codes: [Investigation_Ebola]

No memos

R. Je ne sais pas.

**P14: Ktw_Hgr_Audit_dcd-09_Proche-1.doc - 14:30 [R : Je n’ai jamais entré dans ..] (128:128) (Super)**

Codes: [Investigation_Ebola]

No memos

R : Je n’ai jamais entré dans le triage, au CTE où on passait

**P 4: Bbo_HgrKttmba_Audit_dcd-03_Proche-1.doc - 4:19 [R : Je ne sais pas si c’était ..] (81:81) (Super)**

Codes: [Investigation_Ebola]

No memos

R : Je ne sais pas si c’était comment.

**P15: Ktw_HopMtda_Audit_dcd-05_Proche-1.doc - 15:55 [R. On se lavait les mains et l..] (224:224) (Super)**

Codes: [Investigation_Ebola]

No memos

R. On se lavait les mains et la prise de température.

**P 6: Bbo_HgrKttmba_Audit_dcd-03_Proche-3.doc - 6:47 [R : Psychologiquement c’était ..] (185:186) (Super)**

Codes: [Investigation_Ebola]

No memos

R : Psychologiquement c’était notre première fois de vivre la situation épidémique et il serait mieux de mettre en place de prélèvement et disponibiliser un traitement en attendant les résultats comme le fait le CT, sinon ils nous avaient traumatisés parce que quelqu’un qui n’a jamais vécu l’épidémie on l’amène là où les gens meurt c’est lui traumatiser. Pour le triage il fallait mettre un dispositif intermédiaire entre le temps des examens et les résultats où on doit attendre. Si c’est négatif on traite le patient, le contraire c’est à ce moment qu’on devrait lui transférer à la CTE c’est l’inconvénient et son avantage est qu’il n’aurait plus contamination si quelqu’un était malade.

00 :24 :17

**P 8: Bbo_HgrKttmba_Audit_dcd-04_Proche-2.doc - 8:22 [R : Je ne sais pas] (94:94) (Super)**

Codes: [Investigation_Ebola]

No memos

R : Je ne sais pas

**P10: Ktw_Hgr_Audit_dcd-02_Proche-1.doc - 10:24 [R : Au niveau du triage de foi..] (97:97) (Super)**

Codes: [Investigation_Ebola]

No memos

R : Au niveau du triage de fois sa traine à cause des questions et de la complétude de fiche, cela lorsque vous avez une force, mais il arrivait le moment ou le malade arrive étant gravement on le fait passer et aller aux urgences et là il commence ses soins.

**P15: Ktw_HopMtda_Audit_dcd-05_Proche-1.doc - 15:54 [R. Je ne connais pas cela.] (220:220) (Super)**

Codes: [Investigation_Ebola]

No memos

R. Je ne connais pas cela.

**P20: Ktw_HopMtda_Audit_dcd-11_Proche-1.doc - 20:24 [R : Contrôler ou trier celui q..] (111:111) (Super)**

Codes: [Investigation_Ebola]

No memos

R : Contrôler ou trier celui qui peut avoir le virus ou la maladie qui peut être contagieuse.

**P20: Ktw_HopMtda_Audit_dcd-11_Proche-1.doc - 20:23 [R : C’est pour contrôler.] (107:107) (Super)**

Codes: [Investigation_Ebola]

No memos

R : C’est pour contrôler.

**P19: Ktw_HopMtda_Audit_dcd-10_Proche-1.doc - 19:23 [R : Le triage était important ..] (107:107) (Super)**

Codes: [Investigation_Ebola]

No memos

R : Le triage était important parce que il nous aidait à distinguer si le malade est un malade ordinaire ou s’il s’agit d’un malade d’Ebola. Différencier la maladie ordinaire et la maladie épidémique.

**P 8: Bbo_HgrKttmba_Audit_dcd-04_Proche-2.doc - 8:21 [R : Lorsque vous arrivez à l’h..] (90:90) (Super)**

Codes: [Investigation_Ebola]

No memos

R : Lorsque vous arrivez à l’hôpital, on vous reçoit au triage ou on doit faire les différents examens et lorsque vous n’avez pas la maladie contagieuse, c’est à ce moment là qu’on vous amène dans la structure pour les soins appropriés.

**P 7: Bbo_HgrKttmba_Audit_dcd-04_Proche-1.doc - 7:39 [R : Je n’avais pas vu l’inconv..] (163:164) (Super)**

Codes: [Investigation_Ebola]

No memos

R : Je n’avais pas vu l’inconvénient du tri par c’était question de savoir qui a Ebola et qui n’en a pas. Et lorsque vous y arriver et constater que vous avez la fièvre c’est là que vous pouvez dire que ce service est mauvais. Quelqu’un qui n’a pas de problème ne peut pas dire que c’est mauvais.

00 :13 :55

**P20: Ktw_HopMtda_Audit_dcd-11_Proche-1.doc - 20:8 [R : On nous avait déchargé san..] (39:39) (Super)**

Codes: [Investigation_Ebola]

No memos

R : On nous avait déchargé sans rien dire.

**P 8: Bbo_HgrKttmba_Audit_dcd-04_Proche-2.doc - 8:24 [R : Oui nous y étions passés e..] (102:102) (Super)**

Codes: [Investigation_Ebola]

No memos

R : Oui nous y étions passés et nous y avons fait trois jours.

**P13: Ktw_Hgr_Audit_dcd-08_Proche-2.doc - 13:30 [R : Nous sommes venus avec le ..] (124:124) (Super)**

Codes: [Investigation_Ebola]

No memos

R : Nous sommes venus avec le véhicule du CTE je n’y avais pas passé

**P17: Ktw_HopMtda_Audit_dcd-05_Proche-3.doc - 17:30 [R. C’est pour nous protéger co..] (129:129) (Super)**

Codes: [Investigation_Ebola]

No memos

R. C’est pour nous protéger contre certaines maladies parce que d’abord il faut la propreté.

**P16: Ktw_HopMtda_Audit_dcd-05_Proche-2.doc - 16:36 [R. J’étais lavé les mains puis..] (180:180) (Super)**

Codes: [Investigation_Ebola]

No memos

R. J’étais lavé les mains puis on avait prélevé ma température et en fin relâchée.

**P 9: Bbo_HopScola_Audit_dcd-07_Proche-1.doc - 9:46 [R : Le triage était bon, car a..] (211:211) (Super)**

Codes: [Investigation_Ebola]

No memos

R : Le triage était bon, car avec ce dernier on a confirmé trois cas d’Ébola, lorsqu’ ils trouvent un cas suspect, eux-mêmes appellent l’équipe de la riposte.

**P19: Ktw_HopMtda_Audit_dcd-10_Proche-1.doc - 19:24 [ : Le triage n’a pas des consé..] (111:111) (Super)**

Codes: [Investigation_Ebola]

No memos

 : Le triage n’a pas des conséquences parce que ça ne fait pas trainer le malade ou le traitement du malade.

**P 6: Bbo_HgrKttmba_Audit_dcd-03_Proche-3.doc - 6:18 [R : Le résultat était négatif...] (70:70) (Super)**

Codes: [Investigation_Ebola]

No memos

R : Le résultat était négatif.

**P17: Ktw_HopMtda_Audit_dcd-05_Proche-3.doc - 17:29 [R. A l’arrivée, tout le monde ..] (125:125) (Super)**

Codes: [Investigation_Ebola]

No memos

R. A l’arrivée, tout le monde se lavait les mains, on prélevait la température, puis orienté vers les malades à visiter. En cas de sortie, on était soumis au même geste.

**P 9: Bbo_HopScola_Audit_dcd-07_Proche-1.doc - 9:47 [R : Je ne vois aucun inconvéni..] (215:215) (Super)**

Codes: [Investigation_Ebola]

No memos

R : Je ne vois aucun inconvénient dans le triage, sauf qu’il a des fois où l’on faisait trainer le patient en lui posant plusieurs questions,c’est ce que les malades se plaignaient.

**P 7: Bbo_HgrKttmba_Audit_dcd-04_Proche-1.doc - 7:37 [R : Son existence pour moi je ..] (155:155) (Super)**

Codes: [Investigation_Ebola]

No memos

R : Son existence pour moi je ne vois pas un problème parce qu’on l’avait installé pendant la période de l’épidémie pour laver les mains et on lavait les mains puis on entre sans difficulté.

**P 7: Bbo_HgrKttmba_Audit_dcd-04_Proche-1.doc - 7:38 [R : c’est pour protéger.] (159:159) (Super)**

Codes: [Investigation_Ebola]

No memos

R : c’est pour protéger.

**P10: Ktw_Hgr_Audit_dcd-02_Proche-1.doc - 10:25 [R : Pour nous il n’y avait pas..] (101:101) (Super)**

Codes: [Investigation_Ebola]

No memos

R : Pour nous il n’y avait pas le problème et nous étions venus au triage je n’avais pas peur d’écouter les soignants. Il fallait que j’expliques la situation du malade s’il s’agit de la complétude on pourra le faire étant à l’intérieur pour la prise en charge. J’avais la facilité de m’expliquer sans attendre qu’ils puissent m’orienter

**P16: Ktw_HopMtda_Audit_dcd-05_Proche-2.doc - 16:35 [R. C’est un bon service parce ..] (173:173) (Super)**

Codes: [Investigation_Ebola]

No memos

R. C’est un bon service parce que grâce à lui, on fait la propreté en se lavant les mains à tout moment car certaines gens avaient l’habitude de toucher même la nourriture sans avoir lavé leurs mains.

**P18: Ktw_HopMtda_Audit_dcd-06_Proche-1.doc - 18:2 [R. C’est seulement suite à une..] (9:9) (Super)**

Codes: [Lien Répondant-Défunt(e)]

No memos

R. C’est seulement suite à une relation de voisinage

**P 7: Bbo_HgrKttmba_Audit_dcd-04_Proche-1.doc - 7:6 [Suis l’amie de sa fille nous v..] (6:6) (Super)**

Codes: [Lien Répondant-Défunt(e)]

No memos

Suis l’amie de sa fille nous vendons ensemble elle vivait

**P 5: Bbo_HgrKttmba_Audit_dcd-03_Proche-2.doc - 5:3 [R : C’est la femme de son oncl..] (11:11) (Super)**

Codes: [Lien Répondant-Défunt(e)]

No memos

R : C’est la femme de son oncle maternelle.

**P17: Ktw_HopMtda_Audit_dcd-05_Proche-3.doc - 17:6 [R. Je suis sa cousine la fille..] (25:25) (Super)**

Codes: [Lien Répondant-Défunt(e)]

No memos

R. Je suis sa cousine la fille de son oncle, ma mère c’est sa tente paternelle

**P 3: Bbo_HgrKttmba_Audit_dcd-01_Proche-3.doc - 3:1 [Je m’appelle KAVUGHO SIFA, pet..] (3:3) (Super)**

Codes: [Lien Répondant-Défunt(e)]

No memos

Je m’appelle KAVUGHO SIFA, petite fille de MASIKA du défunt et qui était son garde malade. Le grand père était âgé de 79ans il venait de souffrir de la maladie de prostate.

**P 7: Bbo_HgrKttmba_Audit_dcd-04_Proche-1.doc - 7:8 [R : J’oubli le nom de ce villa..] (30:30) (Super)**

Codes: [Lien Répondant-Défunt(e)]

No memos

R : J’oubli le nom de ce village, par ce quelqu’un peut te dire qu’il va dans la forêt mais on ne sait pas le nom de la forêt où il est parti. On nous avait dit qu’elle vient pour les soins et je me suis familiarisée avec eux et la maman était déjà là.

**P 3: Bbo_HgrKttmba_Audit_dcd-01_Proche-3.doc - 3:2 [R : Je suis sa petite fille (l..] (6:6) (Super)**

Codes: [Lien Répondant-Défunt(e)]

No memos

R : Je suis sa petite fille (la fille de son Maska)

**P18: Ktw_HopMtda_Audit_dcd-06_Proche-1.doc - 18:1 [R. Elle était ma voisine] (5:5) (Super)**

Codes: [Lien Répondant-Défunt(e)]

No memos

R. Elle était ma voisine

**P 3: Bbo_HgrKttmba_Audit_dcd-01_Proche-3.doc - 3:3 [R : C’est sa fille ainée] (10:10) (Super)**

Codes: [Lien Répondant-Défunt(e)]

No memos

R : C’est sa fille ainée

**P 6: Bbo_HgrKttmba_Audit_dcd-03_Proche-3.doc - 6:3 [R : Consolée était mon amie no..] (5:5) (Super)**

Codes: [Lien Répondant-Défunt(e)]

No memos

R : Consolée était mon amie nous avons grandi ensemble presque meme age dans la cellule jusqu’à ce qu’on est devenu toutes de mamans

**P13: Ktw_Hgr_Audit_dcd-08_Proche-2.doc - 13:8 [R : Je suis son père biologiqu..] (33:33) (Super)**

Codes: [Lien Répondant-Défunt(e)]

No memos

R : Je suis son père biologique.

**P 1: Bbo_HgrKttmba_Audit_dcd-01_Proche-1.doc - 1:4 [Répondant : Maternel] (16:16) (Super)**

Codes: [Lien Répondant-Défunt(e)]

No memos

Répondant : Maternel

**P 2: Bbo_HgrKttmba_Audit_dcd-01_Proche-2.doc - 2:1 [R : Je suis son petit fils] (5:5) (Super)**

Codes: [Lien Répondant-Défunt(e)]

No memos

R : Je suis son petit fils

**P15: Ktw_HopMtda_Audit_dcd-05_Proche-1.doc - 15:1 [R : Je suis sa belle sœur] (5:5) (Super)**

Codes: [Lien Répondant-Défunt(e)]

No memos

R : Je suis sa belle sœur

**P15: Ktw_HopMtda_Audit_dcd-05_Proche-1.doc - 15:15 [R. J’étais sa belle sœur] (61:61) (Super)**

Codes: [Lien Répondant-Défunt(e)]

No memos

R. J’étais sa belle sœur

**P11: Ktw_Hgr_Audit_dcd-08_Proche-1.doc - 11:14 [R/ Oui, suis sa mère.] (57:57) (Super)**

Codes: [Lien Répondant-Défunt(e)]

No memos

R/ Oui, suis sa mère.

**P20: Ktw_HopMtda_Audit_dcd-11_Proche-1.doc - 20:4 [R : Je suis sa mère] (23:23) (Super)**

Codes: [Lien Répondant-Défunt(e)]

No memos

R : Je suis sa mère

**P15: Ktw_HopMtda_Audit_dcd-05_Proche-1.doc - 15:2 [R. Oui, bien sur] (9:9) (Super)**

Codes: [Lien Répondant-Défunt(e)]

No memos

R. Oui, bien sur

**P 4: Bbo_HgrKttmba_Audit_dcd-03_Proche-1.doc - 4:1 [R : elle était ma tente matern..] (9:9) (Super)**

Codes: [Lien Répondant-Défunt(e)]

No memos

R : elle était ma tente maternelle

**P16: Ktw_HopMtda_Audit_dcd-05_Proche-2.doc - 16:2 [R. Elle était la sœur à mon pè..] (9:9) (Super)**

Codes: [Lien Répondant-Défunt(e)]

No memos

R. Elle était la sœur à mon père

**P16: Ktw_HopMtda_Audit_dcd-05_Proche-2.doc - 16:1 [R. Elle était ma tente paterne..] (5:5) (Super)**

Codes: [Lien Répondant-Défunt(e)]

No memos

R. Elle était ma tente paternelle

**P 8: Bbo_HgrKttmba_Audit_dcd-04_Proche-2.doc - 8:2 [R : C’est papa qui connait si ..] (10:10) (Super)**

Codes: [Lien Répondant-Défunt(e)]

No memos

R : C’est papa qui connait si je peux être le quantième parmi ses enfants, mais mon nom c’est KYAKIMWA (le sixième enfant de la famille) et nous tous nous sommes sept enfants.

**P19: Ktw_HopMtda_Audit_dcd-10_Proche-1.doc - 19:7 [R : Elle était ma petite sœur] (33:33) (Super)**

Codes: [Lien Répondant-Défunt(e)]

No memos

R : Elle était ma petite sœur

**P 7: Bbo_HgrKttmba_Audit_dcd-04_Proche-1.doc - 7:7 [R : A l’hôpital dans un villag..] (26:26) (Super)**

Codes: [Lien Répondant-Défunt(e)]

No memos

R : A l’hôpital dans un village où elle résidait que j’oubli le nom et elle était venue pour les soins.

**P19: Ktw_HopMtda_Audit_dcd-10_Proche-1.doc - 19:8 [R : Elle est la quatrième nais..] (37:37) (Super)**

Codes: [Lien Répondant-Défunt(e)]

No memos

R : Elle est la quatrième naissance dans la famille et elle vient troisième fois après mois

**P14: Ktw_Hgr_Audit_dcd-09_Proche-1.doc - 14:4 [R : sa femme de Mumbere est ma..] (17:17) (Super)**

Codes: [Lien Répondant-Défunt(e)]

No memos

R : sa femme de Mumbere est ma petite Sœur moi c’est KAVIRA et lui KAVUGHO successivement le deuxième et le troisième enfant.

**P 1: Bbo_HgrKttmba_Audit_dcd-01_Proche-1.doc - 1:3 [Répondant : C’était le grand p..] (14:14) (Super)**

Codes: [Lien Répondant-Défunt(e)]

No memos

Répondant : C’était le grand père.

**P 8: Bbo_HgrKttmba_Audit_dcd-04_Proche-2.doc - 8:1 [R : Je suis sa fille] (6:6) (Super)**

Codes: [Lien Répondant-Défunt(e)]

No memos

R : Je suis sa fille

**P 9: Bbo_HopScola_Audit_dcd-07_Proche-1.doc - 9:13 [R : J’étais son voisin proche...] (60:60) (Super)**

Codes: [Lien Répondant-Défunt(e)]

No memos

R : J’étais son voisin proche.

**P12: Ktw_Hgr_Audit_dcd-08_Proche-12.doc - 12:9 [R : Je suis sa fille, je fus s..] (38:38) (Super)**

Codes: [Lien Répondant-Défunt(e)] [Rôle_Repondant]

No memos

R : Je suis sa fille, je fus sa garde, je lessivais et préparais la nourriture pour elle

**P 5: Bbo_HgrKttmba_Audit_dcd-03_Proche-2.doc - 5:51 [R : C’était dans une mauvaise ..] (194:194) (Super)**

Codes: [Médicaments_Disponibilité]

No memos

R : C’était dans une mauvaise période où tout le monde avait eu peur de fréquenter l’hôpital pour ne pas être amené de force au CTE et en suite y mourir. Même pour lui visiter c’était toujours un problème et on ne pensait pas qu’il pourrait y avoir quelqu’un qui pouvait retourner du CTE, par ce qu’on nous disait que personne ne retournait pas au CTE.

**P 5: Bbo_HgrKttmba_Audit_dcd-03_Proche-2.doc - 5:50 [R : Oui tout le monde avait pe..] (190:190) (Super)**

Codes: [Médicaments_Disponibilité]

No memos

R : Oui tout le monde avait peur de fréquenter l’hôpital ; on avait appelé un garçon de lui donner le sang il avait refusé.

**P 5: Bbo_HgrKttmba_Audit_dcd-03_Proche-2.doc - 5:49 [R : Il n’y avait personne car ..] (186:186) (Super)**

Codes: [Médicaments_Disponibilité]

No memos

R : Il n’y avait personne car tout le monde avait eu peur d’aller à l’hôpital, que si on y arrivait on peut les amener au CTE et on vous injecte une injection qui va vous conduire à la mort.

**P 2: Bbo_HgrKttmba_Audit_dcd-01_Proche-2.doc - 2:24 [R : On ne nous avait pas donné..] (101:101) (Super)**

Codes: [Médicaments_Disponibilité]

No memos

R : On ne nous avait pas donné l’ordonnance.

**P 6: Bbo_HgrKttmba_Audit_dcd-03_Proche-3.doc - 6:40 [R : Oui, quelque fois si son f..] (156:156) (Super)**

Codes: [Médicaments_Disponibilité]

No memos

R : Oui, quelque fois si son frère n’a pas encore donne elle pouvait manquer.

**P 5: Bbo_HgrKttmba_Audit_dcd-03_Proche-2.doc - 5:52 [R : Tout le monde croyait à ce..] (198:198) (Super)**

Codes: [Médicaments_Disponibilité]

No memos

R : Tout le monde croyait à cela par ce que c’était une période où beaucoup de personnes sont morts et on pouvait avoir peur de **son** mari s’il est hospitalisé pour aller lui visiter.

**P11: Ktw_Hgr_Audit_dcd-08_Proche-1.doc - 11:65 [R : C’est comme le paracétamol..] (262:262) (Super)**

Codes: [Médicaments_Disponibilité]

No memos

R : C’est comme le paracétamol, ibuprofène c’est ce qu’ils donnent.

**P16: Ktw_HopMtda_Audit_dcd-05_Proche-2.doc - 16:55 [R. Oui on m’avait donné.] (271:271) (Super)**

Codes: [Médicaments_Disponibilité]

No memos

R. Oui on m’avait donné.

**P 1: Bbo_HgrKttmba_Audit_dcd-01_Proche-1.doc - 1:23 [RÉPONDANT : Non tous les médic..] (73:73) (Super)**

Codes: [Médicaments_Disponibilité]

No memos

RÉPONDANT : Non tous les médicaments étaient disponibles

**P11: Ktw_Hgr_Audit_dcd-08_Proche-1.doc - 11:64 [R : Par ce qu’il y a un médica..] (258:258) (Super)**

Codes: [Médicaments_Disponibilité] [Paiement_Soins_gratuité]

No memos

R : Par ce qu’il y a un médicament de Mueleleye (les médicaments génériques pas de spécialité par ce qu’ils sont chers).

**P 3: Bbo_HgrKttmba_Audit_dcd-01_Proche-3.doc - 3:20 [R : Non je n’étais pas au cour..] (78:78) (Super)**

Codes: [Médicaments_Provenance]

No memos

R : Non je n’étais pas au courant.

**P 3: Bbo_HgrKttmba_Audit_dcd-01_Proche-3.doc - 3:19 [R : Ces sont ses enfants qui a..] (74:74) (Super)**

Codes: [Médicaments_Provenance]

No memos

R : Ces sont ses enfants qui achetait ce médicament et nous nous n’étions pas programmé sur l’achat.

**P10: Ktw_Hgr_Audit_dcd-02_Proche-1.doc - 10:15 [R : Non il n’y avait pas un pr..] (61:61) (Super)**

Codes: [Médicaments_Provenance]

No memos

R : Non il n’y avait pas un problème des médicaments parce que la facture était payée vers la fin. Il n’y avait pas l’obligation de payer la caution de soins

**P 4: Bbo_HgrKttmba_Audit_dcd-03_Proche-1.doc - 4:40 [R : Oui on avait payé.] (164:164) (Super)**

Codes: [Paiement_Soins_gratuité]

No memos

R : Oui on avait payé.

**P 8: Bbo_HgrKttmba_Audit_dcd-04_Proche-2.doc - 8:38 [R : Oui on nous demandait l’ar..] (157:157) (Super)**

Codes: [Paiement_Soins_gratuité]

No memos

R : Oui on nous demandait l’argent pour les soins d’ailleurs nous avions payé **40 dollars** et nous restons avec une dette que nous pensons faire une réunion de famille pour discuter comment pouvoir payer ladite dette. Nous avons beaucoup dépensé lors de funéraire.

**P15: Ktw_HopMtda_Audit_dcd-05_Proche-1.doc - 15:46 [R. Je ne sais pas c’était comb..] (184:184) (Super)**

Codes: [Paiement_Soins_gratuité]

No memos

R. Je ne sais pas c’était combien d’argent.

**P19: Ktw_HopMtda_Audit_dcd-10_Proche-1.doc - 19:26 [R : On avait payé six-cent dol..] (121:121) (Super)**

Codes: [Paiement_Soins_gratuité]

No memos

R : On avait payé six-cent dollars (600$)

**P 4: Bbo_HgrKttmba_Audit_dcd-03_Proche-1.doc - 4:39 [R : Non les soins n’étaient pa..] (160:160) (Super)**

Codes: [Paiement_Soins_gratuité]

No memos

R : Non les soins n’étaient pas gratuits

**P 4: Bbo_HgrKttmba_Audit_dcd-03_Proche-1.doc - 4:38 [R : Oui c’était dans la gratui..] (156:156) (Super)**

Codes: [Paiement_Soins_gratuité]

No memos

R : Oui c’était dans la gratuité

**P15: Ktw_HopMtda_Audit_dcd-05_Proche-1.doc - 15:45 [R. Oui] (181:181) (Super)**

Codes: [Paiement_Soins_gratuité]

No memos

R. Oui

**P 3: Bbo_HgrKttmba_Audit_dcd-01_Proche-3.doc - 3:41 [R : Non on ne demandait pas l’..] (162:162) (Super)**

Codes: [Paiement_Soins_gratuité]

No memos

R : Non on ne demandait pas l’argent et c’est pourquoi on préférait là où on demandait l’argent pour vu qu’on soigne et on ne va pas vous envoyer au CTE

**P 6: Bbo_HgrKttmba_Audit_dcd-03_Proche-3.doc - 6:41 [R : Non durant cette période i..] (160:160) (Super)**

Codes: [Paiement_Soins_gratuité]

No memos

R : Non durant cette période il y avait la gratuité de soins et les examens pour ceux qui étaient hospitalisé mais avant l’éclosion de l’épidémie on payait.

**P13: Ktw_Hgr_Audit_dcd-08_Proche-2.doc - 13:28 [R : Oui, j’ai même la facture ..] (116:116) (Super)**

Codes: [Paiement_Soins_gratuité]

No memos

R : Oui, j’ai même la facture Un moment donné la période de gratuité était fini et moi j’étais arrivé après la gratuité.

**P13: Ktw_Hgr_Audit_dcd-08_Proche-2.doc - 13:16 [R : la radiographie c’était on..] (67:67) (Super)**

Codes: [Paiement_Soins_gratuité]

No memos

R : la radiographie c’était onze dollars mais l’échographie, je ne sais pas vraiment.

**P11: Ktw_Hgr_Audit_dcd-08_Proche-1.doc - 11:63 [R : Les Soins gratuits ne sont..] (254:254) (Super)**

Codes: [Paiement_Soins_gratuité]

No memos

R : Les Soins gratuits ne sont pas bons.

**P11: Ktw_Hgr_Audit_dcd-08_Proche-1.doc - 11:62 [R : 420 dollars américains.] (250:250) (Super)**

Codes: [Paiement_Soins_gratuité]

No memos

R : 420 dollars américains.

**P17: Ktw_HopMtda_Audit_dcd-05_Proche-3.doc - 17:21 [R. En famille, on a beaucoup i..] (89:89) (Super)**

Codes: [Paiement_Soins_gratuité]

No memos

R. En famille, on a beaucoup intervenu. Elle a une fille religieuse qui a intervenu jusqu’à la dernière minute en voulant sauver la vie de sa mère ; elle payait même les factures.

**P 5: Bbo_HgrKttmba_Audit_dcd-03_Proche-2.doc - 5:66 [R : Ils étaient en train de no..] (254:254) (Super)**

Codes: [Paiement_Soins_gratuité]

No memos

R : Ils étaient en train de nous aider par ce que Dieu leurs avaient donné cette grâce d’aider ceux qui sont en difficulté.

**P 5: Bbo_HgrKttmba_Audit_dcd-03_Proche-2.doc - 5:67 [R : Oui nous étions traités sa..] (258:258) (Super)**

Codes: [Paiement_Soins_gratuité]

No memos

R : Oui nous étions traités sans nous demander l’argent et on quittait l’hôpital(KUGOMBOLEWA) pour rentrer chez lui sans payer de l’argent.

**P 9: Bbo_HopScola_Audit_dcd-07_Proche-1.doc - 9:42 [R : Le patient payait les médi..] (195:195) (Super)**

Codes: [Paiement_Soins_gratuité]

No memos

R : Le patient payait les médicaments car la structure était privée

**P 5: Bbo_HgrKttmba_Audit_dcd-03_Proche-2.doc - 5:65 [R : les soins étaient gratuits..] (250:250) (Super)**

Codes: [Paiement_Soins_gratuité]

No memos

R : les soins étaient gratuits ; ils nous avaient aidé parce que nous étions en difficulté et il n’y avait plus moyen de pouvoir chercher de l’argent.

**P11: Ktw_Hgr_Audit_dcd-08_Proche-1.doc - 11:39 [R/ juste au début nous avions ..] (160:160) (Super)**

Codes: [Paiement_Soins_gratuité]

No memos

R/ juste au début nous avions payé 12.5 dollars américains

**P11: Ktw_Hgr_Audit_dcd-08_Proche-1.doc - 11:36 [00 :14 :20 R/ J’avais payé 4.5..] (147:148) (Super)**

Codes: [Paiement_Soins_gratuité]

No memos

00 :14 :20

R/ J’avais payé 4.5 dollars

**P11: Ktw_Hgr_Audit_dcd-08_Proche-1.doc - 11:35 [R/ Non, le prix était bien, la..] (144:144) (Super)**

Codes: [Paiement_Soins_gratuité]

No memos

R/ Non, le prix était bien, la première facture était payable mais la deuxième était gratuite.

**P15: Ktw_HopMtda_Audit_dcd-05_Proche-1.doc - 15:43 [R. Je ne sais pas.] (170:170) (Super)**

Codes: [Paiement_Soins_gratuité]

No memos

R. Je ne sais pas.

**P13: Ktw_Hgr_Audit_dcd-08_Proche-2.doc - 13:29 [R : Oui oui ça devrait dépasse..] (120:120) (Super)**

Codes: [Paiement_Soins_gratuité]

No memos

R : Oui oui ça devrait dépasser

**P16: Ktw_HopMtda_Audit_dcd-05_Proche-2.doc - 16:51 [R. J’avais payé 21 dollars amé..] (252:252) (Super)**

Codes: [Paiement_Soins_gratuité]

No memos

R. J’avais payé 21 dollars américains.

**P15: Ktw_HopMtda_Audit_dcd-05_Proche-1.doc - 15:44 [R. C’est à Matanda où on avait..] (177:177) (Super)**

Codes: [Paiement_Soins_gratuité]

No memos

R. C’est à Matanda où on avait demandé de l’argent.

**P14: Ktw_Hgr_Audit_dcd-09_Proche-1.doc - 14:29 [Comme c’était dans une période..] (124:124) (Super)**

Codes: [Paiement_Soins_gratuité]

No memos

Comme c’était dans une période de gratuité ils ne voulaient pas accepter pour certains services mais pour la seconde fois nous y avons fait sept jours et nous avons payé Cent vingt-huit point trente-quatre dollars.

**P 9: Bbo_HopScola_Audit_dcd-07_Proche-1.doc - 9:30 [R : Non, il n’a pas eu ce prob..] (136:136) (Super)**

Codes: [Paiement_Soins_gratuité]

No memos

R : Non, il n’a pas eu ce problème

**P10: Ktw_Hgr_Audit_dcd-02_Proche-1.doc - 10:16 [R : Nous avions payé Cent Nona..] (65:65) (Super)**

Codes: [Paiement_Soins_gratuité]

No memos

R : Nous avions payé Cent Nonante six dollars y compris le frais d’un jour pour la morgue payable à cinquante dollars.

**P18: Ktw_HopMtda_Audit_dcd-06_Proche-1.doc - 18:29 [R. Oui elle avait payé ] (115:115) (Super)**

Codes: [Paiement_Soins_gratuité]

No memos

R. Oui elle avait payé

**P18: Ktw_HopMtda_Audit_dcd-06_Proche-1.doc - 18:30 [R. Non, non ! Elle, avait payé..] (119:119) (Super)**

Codes: [Paiement_Soins_gratuité]

No memos

R. Non, non ! Elle, avait payé de l’argent

**P16: Ktw_HopMtda_Audit_dcd-05_Proche-2.doc - 16:54 [R. Oui, il y a un jour où j’av..] (264:264) (Super)**

Codes: [Paiement_Soins_gratuité]

No memos

R. Oui, il y a un jour où j’avais aussi peur d’y aller.

**P16: Ktw_HopMtda_Audit_dcd-05_Proche-2.doc - 16:53 [R. Oui, on demandait aux gens ..] (260:260) (Super)**

Codes: [Paiement_Soins_gratuité]

No memos

R. Oui, on demandait aux gens d’aller se faire soigner gratuitement. Certains y allaient, d’autres refusaient en disant que ces médicaments n’étaient pas de bonne qualité.

**P16: Ktw_HopMtda_Audit_dcd-05_Proche-2.doc - 16:52 [R. Cette offre de service n’ex..] (256:256) (Super)**

Codes: [Paiement_Soins_gratuité]

No memos

R. Cette offre de service n’existait plus.

**P18: Ktw_HopMtda_Audit_dcd-06_Proche-1.doc - 18:34 [R. Non. Non vraiment, étant do..] (135:135) (Super)**

Codes: [Paiement_Soins_gratuité]

No memos

R. Non. Non vraiment, étant donné que le moment où elle était partie à KYONDO, la famille a trouvé bon l’amener encore aux soins à Matanda.

**P13: Ktw_Hgr_Audit_dcd-08_Proche-2.doc - 13:27 [Ils n’étaient pas rémunérés et..] (112:112) (Super)**

Codes: [Paiement_Soins_gratuité] [Points_Positif-Négatif]

No memos

Ils n’étaient pas rémunérés et disaient que le traitement gratuit alors que l’Etat ne donne pas la motivation, on ne fait que les envoyer les patients sans prime. Ils disaient à haute voix

**P13: Ktw_Hgr_Audit_dcd-08_Proche-2.doc - 13:15 [R : C’était ça normalement. Co..] (62:62) (Super)**

Codes: [Paracliniques_Accès]

No memos

R : C’était ça normalement. Comme je vous ai dit, les soignants se plaignaient qu’ils ne sont pas rémunérés, alors pour faire la radiographie il fallait attendre beaucoup de jours, et aussi ils exigeaient d’abord l’argent, et sans ce dernier on ne pouvait pas le faire. Il y’a des fois où ils disaient qu’il passera à l’échographie, mais ça n’a pas été fait à cause de gens de la riposte qui avaient dit qu’on les soins doivent être gratuits et ils ne paient pas. Même dans l’hôpital ils nous disaient « malgré que vous êtes déchargé du CTE vous devez comprendre que les soins ne sont plus gratuits et commencer à chercher l’argent pour que l’enfant fasse la radiographie et l’échographie.

**P 3: Bbo_HgrKttmba_Audit_dcd-01_Proche-3.doc - 3:39 [R : Là il n’y avait pas d’Ebol..] (154:154) (Super)**

Codes: [Paracliniques_Accès]

No memos

R : Là il n’y avait pas d’Ebola.

**P 3: Bbo_HgrKttmba_Audit_dcd-01_Proche-3.doc - 3:38 [R : On ne fait plus les examen..] (150:150) (Super)**

Codes: [Paracliniques_Accès]

No memos

R : On ne fait plus les examens, même moi j’y étais un jour pour faire l’examen du selles mais on m’avait obligé d’aller à ITAVE (CTE de Butembo) et comme ils avaient refusé j’étais obligé d’aller chez les tradi praticien à Butuhye

**P 2: Bbo_HgrKttmba_Audit_dcd-01_Proche-2.doc - 2:25 [R : Oui il les avait faits] (105:105) (Super)**

Codes: [Paracliniques_Accès]

No memos

R : Oui il les avait faits

**P 6: Bbo_HgrKttmba_Audit_dcd-03_Proche-3.doc - 6:20 [R : Non à cette époque on ne f..] (78:78) (Super)**

Codes: [Paracliniques_Accès]

No memos

R : Non à cette époque on ne faisait plus les examens aux postes de santé et les gens y préféraient dans le but de ne pas être envoyé au CTE et si vous n’étiez pas gerris ils étaient obligés de vous transférer dans une grande structure qui va à son tour vous envoyer au CTE, et les soignants du poste de santé avaient peur de garder un malade plus de quatre jours comme il y avait une équipe de la riposte qui circulait dans les petites structures sanitaires pour le contrôle des malades.

**P11: Ktw_Hgr_Audit_dcd-08_Proche-1.doc - 11:33 [R/ Oui j’avais fait les examen..] (136:136) (Super)**

Codes: [Paracliniques_Accès]

No memos

R/ Oui j’avais fait les examens ici deux fois.

**P11: Ktw_Hgr_Audit_dcd-08_Proche-1.doc - 11:42 [R/ A Katwa nous y avons fait t..] (172:172) (Super)**

Codes: [Paracliniques_Accès]

No memos

R/ A Katwa nous y avons fait trop d’examens : l’échographie d’abord pour savoir la source de la maladie, puis la radiographie 4 fois.

**P11: Ktw_Hgr_Audit_dcd-08_Proche-1.doc - 11:34 [R/Nous avions fait l’examen de..] (140:140) (Super)**

Codes: [Paracliniques_Accès]

No memos

R/Nous avions fait l’examen de crachant.

**P 9: Bbo_HopScola_Audit_dcd-07_Proche-1.doc - 9:26 [R : Il avait fait des examens,..] (117:117) (Super)**

Codes: [Paracliniques_Accès]

No memos

R : Il avait fait des examens, il y avait un médecin dans cette structure ; même les gens de riposte étaient arrivés un jour et ils avaient fait des prélèvements.

**P 9: Bbo_HopScola_Audit_dcd-07_Proche-1.doc - 9:23 [R : On avait fait des examens ..] (103:103) (Super)**

Codes: [Paracliniques_Accès]

No memos

R : On avait fait des examens et il avait aussi les résultats seulement je n’étais pas curieuse de regarder ces résultats.

**P10: Ktw_Hgr_Audit_dcd-02_Proche-1.doc - 10:14 [R : Pour les examens c’était g..] (57:57) (Super)**

Codes: [Paracliniques_Accès]

No memos

R : Pour les examens c’était gratuit sauf qu’un examen ou il m’avait dit qu’on demande de payer avant de le faire. C’était un seul examen mais les reste d’examens c’était gratuit. Et ils avaient autorisé de le faire malgré qu’on m’avait dit cela, on l’avait payé après que ce dernier est déjà fait. Pour la collaboration nous famille est l’hôpital c’était bonne.

**P 6: Bbo_HgrKttmba_Audit_dcd-03_Proche-3.doc - 6:33 [R : Oui elle avait fait des ex..] (131:131) (Super)**

Codes: [Paracliniques_Accès]

No memos

R : Oui elle avait fait des examens et d’ailleurs après qu’on nous dira qu’elle était atteinte du VIH sida et il parait on avait informé sa grande sœur mais il y avait une confidentialité dans la famille restreinte.

**P 1: Bbo_HgrKttmba_Audit_dcd-01_Proche-1.doc - 1:24 [RÉPONDANT : Au CTE ? Non, à Ki..] (77:77) (Super)**

Codes: [Paracliniques_Accès]

No memos

RÉPONDANT : Au CTE ? Non, à Kitatumba on n’avait pas fait les examens.

**P11: Ktw_Hgr_Audit_dcd-08_Proche-1.doc - 11:43 [R : Ils avaient dit qu’ils ne ..] (176:176) (Super)**

Codes: [Paracliniques_Accès]

No memos

R : Ils avaient dit qu’ils ne trouvent pas la maladie mais l’enfant toussait d’une manière grave.

**P12: Ktw_Hgr_Audit_dcd-08_Proche-12.doc - 12:32 [R : Oui elle avait fait les ex..] (134:134) (Super)**

Codes: [Paracliniques_Accès]

No memos

R : Oui elle avait fait les examens

**P 4: Bbo_HgrKttmba_Audit_dcd-03_Proche-1.doc - 4:31 [R : Oui elle avait fait les ex..] (128:128) (Super)**

Codes: [Paracliniques_Accès]

No memos

R : Oui elle avait fait les examens.

**P14: Ktw_Hgr_Audit_dcd-09_Proche-1.doc - 14:14 [R : Echographie au premier tou..] (57:57) (Super)**

Codes: [Paracliniques_Accès]

No memos

R : Echographie au premier tours, il ne l’avait pas fait

**P14: Ktw_Hgr_Audit_dcd-09_Proche-1.doc - 14:15 [R : Oui j’étais partie cherche..] (61:61) (Super)**

Codes: [Paracliniques_Accès]

No memos

R : Oui j’étais partie chercher le radiologue et je lui avais trouvé là où il soignait en lui disant « Pardon papa, vous pouvez venir voir le malade. Après une heure il revient en me disant : je ne vais plus te cacher ton beau-frère est malade, vous pouvez rentrer à la maison pour attendre la décision divine » On était encore dans les soins intensifs. Je m’étais tu par ce que sa femme était moins âgée je ne pouvais pas lui donner cette information.

**P 5: Bbo_HgrKttmba_Audit_dcd-03_Proche-2.doc - 5:40 [R : Oui très bien par ce qu’el..] (151:151) (Super)**

Codes: [Paracliniques_Accès]

No memos

R : Oui très bien par ce qu’elle accompagnait les gens à chaque fois qu’on lui visitait on se disait qu’elle était guérie.

**P 5: Bbo_HgrKttmba_Audit_dcd-03_Proche-2.doc - 5:39 [R : Elle avait déjà fait les e..] (147:147) (Super)**

Codes: [Paracliniques_Accès]

No memos

R : Elle avait déjà fait les examens au CTE ; après trois jours de traitement comme Dieu est merveilleux et elle avait commencé à faire pas à ceux qui étaient venus lui visiter.

**P 2: Bbo_HgrKttmba_Audit_dcd-01_Proche-2.doc - 2:26 [R : Oui on avait demandé, quan..] (109:109) (Super)**

Codes: [Paracliniques_Accès]

No memos

R : Oui on avait demandé, quand on est à l’hôpital on doit toujours demander l’argent et on est sensé de donner.

**P 5: Bbo_HgrKttmba_Audit_dcd-03_Proche-2.doc - 5:41 [R : Oui] (155:155) (Super)**

Codes: [Paracliniques_Accès]

No memos

R : Oui

**P16: Ktw_HopMtda_Audit_dcd-05_Proche-2.doc - 16:33 [R. Vraiment tout était bien fa..] (147:147) (Super)**

Codes: [Points_Positif-Négatif]

No memos

R. Vraiment tout était bien fait.

**P15: Ktw_HopMtda_Audit_dcd-05_Proche-1.doc - 15:52 [R. Elle était bien suivie, c’é..] (212:212) (Super)**

Codes: [Points_Positif-Négatif]

No memos

R. Elle était bien suivie, c’était vraiment le moment pour mourir.

**P15: Ktw_HopMtda_Audit_dcd-05_Proche-1.doc - 15:53 [R. Chaque personne a son jour ..] (216:216) (Super)**

Codes: [Points_Positif-Négatif]

No memos

R. Chaque personne a son jour pour mourir. Ils ont soigné

**P20: Ktw_HopMtda_Audit_dcd-11_Proche-1.doc - 20:20 [R : Je dirais que tout marchai..] (91:91) (Super)**

Codes: [Points_Positif-Négatif]

No memos

R : Je dirais que tout marchait on nous donnait des ordonnances et on achetait les médicaments sauf les résultats qu’on avait attendus pour confirmer si c’est la méningite ou pas. Etant un soignant tu ne sais pas quel médicament qui va traiter le malade ?

**P17: Ktw_HopMtda_Audit_dcd-05_Proche-3.doc - 17:26 [R. Tout était bien parce que l..] (113:113) (Super)**

Codes: [Points_Positif-Négatif]

No memos

R. Tout était bien parce que lorsqu’on donnait une ordonnance médicale, on faisait tout pour acheter tous les médicaments prescrits.

**P17: Ktw_HopMtda_Audit_dcd-05_Proche-3.doc - 17:27 [R. Tout le monde a travaillé c..] (117:117) (Super)**

Codes: [Points_Positif-Négatif]

No memos

R. Tout le monde a travaillé ce qu’il devait, la famille, et les infirmiers. Et d’ailleurs les infirmiers de Matanda ont beaucoup soigné

**P14: Ktw_Hgr_Audit_dcd-09_Proche-1.doc - 14:27 [Ceux qui n’avaient pas marché ..] (114:117) (Super)**

Codes: [Points_Positif-Négatif]

No memos

Ceux qui n’avaient pas marché parmi ces quatre sont à deux il s’agit de :

- Sa famille biologique par ce qu’elle s’était penché aux autres enfant qui ont de moyen financier et surtout sa mère voulait que son fils quitte ou abandonne son foyer pour aller chez lui enfin de l’observer durant sa maladie chose que son fils et belle-fille n’ont pas accepter. Et surtout qu’il était déjà un père de famille. Cela avait affecté le patient lorsqu’il était amené chez eux en quittant chez et y laisser sa femme et ses enfants. Et sa femme lui avait demandé de rentrer à la maison chose que ce dernier n’avait pas accepter par ce que ses parents lui avaient demandé de ne plus retourné chez lui sinon il ne verra personne à sa porte pour lui visiter. La femme du défunt était enseignante alors que sa belle-famille tous sont des cultivateurs d’où une sorte de complexe à l’égard de cette femme qu’elle ne cultive pas. Et tout ce que on pouvait décider dans la famille il fallait demander son petit frère KAKULE comme c’est lui qui avait l’argent. Lui était Mumbere

- Lors de la gratuité et la période normale de soins on devrait uniformiser le même traitement par ce qu’il toujours les mêmes pathologies. Il y a eu une différence de soins pendant notre prise en charge, je ne sais pas si c’est à cause de la gratuité de soins qu’ils nous avaient traité de cette manière. En tant que professionnels de santé ils devraient la même déontologie professionnelle pendant la période de gratuité et celle de routine. Les patients qui sont dans les chambres privées étaient complètement prise en charge plus que ceux qui sont dans la salle commune. Comme ceux qui sont dans les privés payaient mieux ils étaient suivis mieux que ceux qui étaient dans les salles communes. Les soignants n’ont pas le même regard envers les malades. Alors que l’on est venu en ayant l’espoir d’être mieux accueillis, par ce que si on s’occupe de toi physiquement et spirituellement c’est le même. Et si on ne s’occupe pas de toi psychologiquement on se demande beaucoup de questions.

00 :19 :50

**P19: Ktw_HopMtda_Audit_dcd-10_Proche-1.doc - 19:19 [R : Je crois c’était au niveau..] (90:90) (Super)**

Codes: [Points_Positif-Négatif]

No memos

R : Je crois c’était au niveau du malade, qui ne voulait pas informer comme c’était une grossesse qu’elle avait eue en clandestinité. Cela lui touche psychologiquement en pensant que si la famille était au courant on va lui secouer en demandant comment elle peut porter une grossesse dans les âges avancées chose qu’elle n’avait pas fait pendant ses jeunes âges. Elle avait informé lorsque la maladie était déjà grave.

**P16: Ktw_HopMtda_Audit_dcd-05_Proche-2.doc - 16:34 [R. Oui parce que l’on achetait..] (151:151) (Super)**

Codes: [Points_Positif-Négatif]

No memos

R. Oui parce que l’on achetait autant des médicaments pour chercher seulement sa guérison. On se souciait d’elle durant l’époque de son état de maladie.

**P18: Ktw_HopMtda_Audit_dcd-06_Proche-1.doc - 18:55 [R. * De ma part en tant que vo..] (226:230) (Super)**

Codes: [Points_Positif-Négatif]

No memos

R. * De ma part en tant que voisine, la défunte était disposée pour son traitement.

* la famille a bien joué son rôle parce qu’ils ont tout fait pour la prise en charge du malade tout était bon.

* Pour les prestataires se donnaient à tout prix pour tenter de lui sauver

* Pour les structures fréquentées, vraiment c’était des hôpitaux renommés, dans les soins pour la meilleure prise en charge.

Vraiment tout aller bien. Elle était bien soignée, c’était seulement son moment de décéder.

**P 9: Bbo_HopScola_Audit_dcd-07_Proche-1.doc - 9:37 [R : Selon moi, c’est l’hôpital..] (174:174) (Super)**

Codes: [Points_Positif-Négatif]

No memos

R : Selon moi, c’est l’hôpital qui manque certain service comme d’échographie, et la radiographie

**P 9: Bbo_HopScola_Audit_dcd-07_Proche-1.doc - 9:38 [R : Peux être que c’était au n..] (178:178) (Super)**

Codes: [Points_Positif-Négatif]

No memos

R : Peux être que c’était au niveau de l’hôpital ou il y avait de problème. Peut-être que s’il faisait la radiographie ça pourrait lui aider.

**P 9: Bbo_HopScola_Audit_dcd-07_Proche-1.doc - 9:39 [R : Peut-être qu’ils disaient ..] (182:182) (Super)**

Codes: [Points_Positif-Négatif]

No memos

R : Peut-être qu’ils disaient ou qu’ils croyaient que c’étaient de la fatigue alors que l’on fond de lui étaient cachés d’autres maladies.

**P 8: Bbo_HgrKttmba_Audit_dcd-04_Proche-2.doc - 8:20 [R : Je ne trouve pas ce qui n’..] (86:86) (Super)**

Codes: [Points_Positif-Négatif]

No memos

R : Je ne trouve pas ce qui n’avait pas marché parmi les tous seulement ce que je trouve est que la maladie venait d’arriver à son terme. Il n’y avait plus à faire.

**P 7: Bbo_HgrKttmba_Audit_dcd-04_Proche-1.doc - 7:34 [R : Je disais que les prestata..] (143:143) (Super)**

Codes: [Points_Positif-Négatif]

No memos

R : Je disais que les prestataires ont fait ce qu’ils pouvaient faire.

**P 7: Bbo_HgrKttmba_Audit_dcd-04_Proche-1.doc - 7:35 [R : Pour la structure il n’y a..] (147:147) (Super)**

Codes: [Points_Positif-Négatif]

No memos

R : Pour la structure il n’y a pas de problème par ce que tous les examens et les services y sont.

**P 7: Bbo_HgrKttmba_Audit_dcd-04_Proche-1.doc - 7:36 [R : Dans tous je ne vois pas c..] (151:151) (Super)**

Codes: [Points_Positif-Négatif]

No memos

R : Dans tous je ne vois pas ce qui n’a pas marché

**P 9: Bbo_HopScola_Audit_dcd-07_Proche-1.doc - 9:40 [R : Je pense que c’est oui.] (186:186) (Super)**

Codes: [Points_Positif-Négatif]

No memos

R : Je pense que c’est oui.

**P10: Ktw_Hgr_Audit_dcd-02_Proche-1.doc - 10:21 [R : Je ne pas de connaissance ..] (85:85) (Super)**

Codes: [Points_Positif-Négatif]

No memos

R : Je ne pas de connaissance avec eux mais aussi je ne suis pas exigent ou timide car je m’adapte facilement par rapport à la réalité du milieu.

**P10: Ktw_Hgr_Audit_dcd-02_Proche-1.doc - 10:28 [R : Tous ces quatre paramètres..] (109:112) (Super)**

Codes: [Points_Positif-Négatif]

No memos

R : Tous ces quatre paramètres entaient réunis :

• Le malade était calme, il savait qu’on lui suivait de près et il avait confiance en moi. Tout activité que je prenne en responsable réussit toujours même s’il y avait déjà un désespoir souvent ça marche.

• Hôpital tout a marché même au niveau de l’organisation nous avons retenu une chose, la cause de son décès et tel que nous connaissons cette maladie il n’y avait pas moyen de faire autrement. Il fallait l’amener en Inde mais vu nos moyens et son âge ce n’était pas facile. C’était le jour où Dieu avait prévu de l’appeler.

• Au niveau de la famille nous avons fait le minimum de ce que nous pouvions faire par ce que même la maman qui est resté nous avait remercié de ce que nous avons fait. Bref les quatre éléments entaient positifs. Au niveau de l’hôpital on ne reproche rien ainsi que la famille.

**P11: Ktw_Hgr_Audit_dcd-08_Proche-1.doc - 11:55 [R/ Ce qui n’a pas marché c’est..] (223:223) (Super)**

Codes: [Points_Positif-Négatif]

No memos

R/ Ce qui n’a pas marché c’est au niveau de l’hôpital de KATWA avant d’entrer dans les soins intensifs, dès que nous y sommes arrivés on nous a conduit à la salle commune hommes où il y avait le retard des soins et lorsqu’il était entré aux soins intensifs c’est là qu’on avait commencé à lui traité par ce qu’il y avait des soignants en permanence à tout moment. Et je me disais que si on était accueilli ici pour la première fois mon fils allait être récupéré, par ce qu’il y avait seulement une jeune fille je ne sais si elle était stagiaire c’est elle seule qui faisait la tour de salle et lorsqu’elle entrait l’enfant dit aaaa maman encore elle vient encore, mon Dieu.

**P10: Ktw_Hgr_Audit_dcd-02_Proche-1.doc - 10:20 [R : C’est qui était de bon c’e..] (81:81) (Super)**

Codes: [Points_Positif-Négatif]

No memos

R : C’est qui était de bon c’est la collaboration de personnel soignant car pour les chambres cliniques on exige la caution pour les occuper, mais moi j’avais demandé la clé et la caissière m’avait donné sans aucune caution.

**P 9: Bbo_HopScola_Audit_dcd-07_Proche-1.doc - 9:50 [R : Personnellement je peux di..] (228:228) (Super)**

Codes: [Points_Positif-Négatif]

No memos

R : Personnellement je peux dire que l’accueil est bien ils font bien leurs travaux, ils écoutent les malades et sont très rapides et souple. Et les autres disent la même chose.

**P 9: Bbo_HopScola_Audit_dcd-07_Proche-1.doc - 9:51 [R : Eux aussi disent la même c..] (232:232) (Super)**

Codes: [Points_Positif-Négatif]

No memos

R : Eux aussi disent la même chose que moi.

**P10: Ktw_Hgr_Audit_dcd-02_Proche-1.doc - 10:19 [R : Je dirais qu’il n’y a rien..] (77:77) (Super)**

Codes: [Points_Positif-Négatif]

No memos

R : Je dirais qu’il n’y a rien qui n’a pas marché par ce que lorsque j’observais quelque chose je cherchais immédiatement les soignants et s’il y avait besoin de médecin on lui cherchait rapidement et il intervenait. Dans une semaine que nous avions faite je n’avais pas observé une mauvaise situation.

**P 7: Bbo_HgrKttmba_Audit_dcd-04_Proche-1.doc - 7:33 [R : Là je ne sais pas et je ne..] (139:139) (Super)**

Codes: [Points_Positif-Négatif]

No memos

R : Là je ne sais pas et je ne peux pas entrer dans ses détailles

**P 2: Bbo_HgrKttmba_Audit_dcd-01_Proche-2.doc - 2:43 [R : Au niveau de la famille il..] (177:177) (Super)**

Codes: [Points_Positif-Négatif]

No memos

R : Au niveau de la famille il n’y avait pas de problème, à tout moment qu’il avait besoin de médicament on achetait toujours.

**P 2: Bbo_HgrKttmba_Audit_dcd-01_Proche-2.doc - 2:44 [R : Pendant l’épidémie, on ava..] (181:181) (Super)**

Codes: [Points_Positif-Négatif]

No memos

**R : Pendant l’épidémie, on avait observé une certaine particularité à l’hôpital** si non avait quelqu’un comme connaissance parmi les personnels soignants, on devait vous accueillir et vous soigner sans condition.

**P 2: Bbo_HgrKttmba_Audit_dcd-01_Proche-2.doc - 2:45 [R : L’hôpital en soit n’avait ..] (185:185) (Super)**

Codes: [Points_Positif-Négatif]

No memos

R : L’hôpital en soit n’avait aucun problème

**P 2: Bbo_HgrKttmba_Audit_dcd-01_Proche-2.doc - 2:42 [R : Non il prenait les médicam..] (173:173) (Super)**

Codes: [Points_Positif-Négatif]

No memos

R : Non il prenait les médicaments sans problème.

**P 1: Bbo_HgrKttmba_Audit_dcd-01_Proche-1.doc - 1:35 [RÉPONDANT : Nous demandons aux..] (121:121) (Super)**

Codes: [Points_Positif-Négatif]

No memos

RÉPONDANT : Nous demandons aux responsables des structures de pouvoir recadrer les prestataires des soins par ce que même dans la période de la MVE il y a beaucoup de gens qui sont décédés pas par l’épidémie mais par la négligence et la peur des personnels soignants, à cause de faire attendre le malade les résultats du CTE alors que le malade est venu se faire soigner. Par ce que même ici chez moi il y avait un enfant qui était malade et lorsqu’on l’avait amené à l’hôpital on nous dira d’attendre l’équipe de la riposte parce qu’il ne sera pas soigné s’il ne part pas au CTE, je leurs avait demandé de nous consulter avant d’aller au CTE ils avaient refusé. Et nous étions rentré à la maison, comme la maladie continuait nous lui avons amené à Katwa et là comme on avait une petite connaissance. Cette dernière confirma auprès de soignants qu’il nous connaissait et que le prélèvement pouvait se faire en étant prise en charge. On nous soignait étant dans la chambre privée. A la sortie il n’était pas complètement guéri et on se demandait de faire quoi alors que nous venions de quitter une grande Fosa, mais quelqu’un nous conseilla d’aller encore dans une autre structure comme c’était le problème de d’anémie. Nous étions partis dans un laboratoire de la ville où on nous dira que son taux d’hémoglobine est à 6% et nous dira que le malade ne pouvait plus passer la nuit s’il ne prend pas les médicaments. Il nous donna une ordonnance des médicaments à acheter et on les avait achetés et on donna d’autres choses qui augmentent le sang et jusqu’à présent il est vivant. Si on pouvait continuer avec l’hôpital il allait décéder par ce qu’à l’hôpital on avait plus l’habitude de faire les examens du sang. Lorsque vous y arrivez c’était toujours Ebola et cela a causé beaucoup de décès.

**P 2: Bbo_HgrKttmba_Audit_dcd-01_Proche-2.doc - 2:39 [R : La première chose est que ..] (161:161) (Super)**

Codes: [Points_Positif-Négatif]

No memos

R : La première chose est que lorsqu’un malade arrive à l’hôpital il faut que les soignants lui prennent en charge d’abord.

**P 2: Bbo_HgrKttmba_Audit_dcd-01_Proche-2.doc - 2:40 [R : Son souci était qu’il guér..] (165:165) (Super)**

Codes: [Points_Positif-Négatif]

No memos

R : Son souci était qu’il guérisse et continuer avec ses activités courantes.

**P 3: Bbo_HgrKttmba_Audit_dcd-01_Proche-3.doc - 3:54 [R : Je n’ai pas de bonnes chos..] (214:214) (Super)**

Codes: [Points_Positif-Négatif]

No memos

R : Je n’ai pas de bonnes choses à se souvenir sur la maladie du grand père, comme le dit la loi des soignant par rapport aux malades la façon dont ils doivent se comporter face aux malades. Avant ils s’occupaient de malade, mais aujourd’hui ce n’est ne pas le cas on se demande si les malades ont un esprit qui fait qu’ils aient peur d’eux.

**P 6: Bbo_HgrKttmba_Audit_dcd-03_Proche-3.doc - 6:52 [R : Il y avait une période où ..] (205:205) (Super)**

Codes: [Points_Positif-Négatif]

No memos

R : Il y avait une période où les patients ne fréquentaient plus la structure à cause de l’hygiène.

**P 7: Bbo_HgrKttmba_Audit_dcd-04_Proche-1.doc - 7:31 [Lui-même était arrivée à l’hôp..] (131:131) (Super)**

Codes: [Points_Positif-Négatif]

No memos

Lui-même était arrivée à l’hôpital étant gravement malade de façon qu’elle n’avait plus la force, et le pouvoir sur lui-même. C’est les soignants lui donnaient les médicaments comme il fallait, la tâche était restée chez les prestataires qui s’occupaient d’elle.

**P 7: Bbo_HgrKttmba_Audit_dcd-04_Proche-1.doc - 7:32 [R : La famille s’était donné p..] (135:135) (Super)**

Codes: [Points_Positif-Négatif]

No memos

R : La famille s’était donné pour sa prise en charge en s’occupant d’elle pendant ses soins.

**P 6: Bbo_HgrKttmba_Audit_dcd-03_Proche-3.doc - 6:51 [R : L’hôpital en tant que stru..] (201:201) (Super)**

Codes: [Points_Positif-Négatif]

No memos

R : L’hôpital en tant que structure, organise tous les services mais dit-on qu’il n’y a pas d’hygiène dans les salles et dans les latrines. Les hygiénistes ne nettoient pas chaque jour c’est maintenant qu’ils sont en train de construire.

**P 3: Bbo_HgrKttmba_Audit_dcd-01_Proche-3.doc - 3:55 [R : La famille s’était donné p..] (222:222) (Super)**

Codes: [Points_Positif-Négatif]

No memos

R : La famille s’était donné pour l’accompagné à l’hôpital pendant ses soins.

**P 6: Bbo_HgrKttmba_Audit_dcd-03_Proche-3.doc - 6:45 [R : C’était dans une période c..] (176:176) (Super)**

Codes: [Points_Positif-Négatif]

No memos

R : C’était dans une période critique de l’épidémie, même si les examens étaient négatifs le prestataire avaient une réticence exagérée de peur d’être contaminé ; quand vous arrivez, la première de chose était la référence vers le CTE et leurs façons de transporter les malades n’étaient pas bonne car c’était sous forme de coffre où on y a pulvérisé le chlore et souvent on y vomissait. Et lorsque vous y arrivez on fait comme si on vous a oublié alors que la chlore vous dérange. Le transport des malades n’était pas bon

**P 6: Bbo_HgrKttmba_Audit_dcd-03_Proche-3.doc - 6:46 [R : Les prestataires ont conti..] (180:180) (Super)**

Codes: [Points_Positif-Négatif]

No memos

R : Les prestataires ont continué à lui traiter malgré son état de santé qui se détériorait, ils nous réconfortaient de temps en temps et ils continuaient à lui traiter malgré ce qui est arrivé.

**P13: Ktw_Hgr_Audit_dcd-08_Proche-2.doc - 13:26 [Ça veut dire qu’ils n’étaient ..] (110:110) (Super)**

Codes: [Points_Positif-Négatif]

No memos

Ça veut dire qu’ils n’étaient pas rémunérés ?

**P12: Ktw_Hgr_Audit_dcd-08_Proche-12.doc - 12:33 [R : Les soignants qui étaient ..] (143:143) (Super)**

Codes: [Points_Positif-Négatif]

No memos

R : Les soignants qui étaient le facteur par ce que leurs attitudes n’étaient pas bonnes, la négligence de malade, mais lors du traitement ils n’ont plus d’affection envers le malade en se rapprochant du malade pour lui donner le réconfort. Mais pour eux par ce qu’on lui programmer de placer la perfusion il vient exécuter seulement les prescriptions, le malade aura le traitement sans la conviction d’être guéris. Parce que le réconfort de soignants fait partie des médicaments lorsqu’ils s’approche de malade, les quelques paroles de réconfort leurs soulage. Parce que maman me disait que ses soignants cherchent ma mort et moi je lui disais que non tu vas guérir. Elle dit encore qu’on fait une journée entière sans un soignant qui vient visiter le malade dans ou voir leur évolution ?

**P13: Ktw_Hgr_Audit_dcd-08_Proche-2.doc - 13:24 [R : le premier facteur qui n’a..] (104:104) (Super)**

Codes: [Points_Positif-Négatif]

No memos

R : le premier facteur qui n’a pas marcher, ç’a été les soignants, ils doivent être capables de découvrir les maladies des enfants par ce qu’ils sont des spécialistes en matière et ils peuvent être souple pour le transfert, mais là au centre de santé où on avait commencé, tellement que l’épouse est relais communautaire là, on y a fait tant de jours sans qu’ils ne découvrent pas la maladie de l’enfant, il y’a de fois où ils disaient que l’enfant est venu nous vous avons dit qu’on ne trouve pas la pathologie et vous continuer de l’amener. Je suis arrivé au point de l’amener loin d’ici à cause de la légèreté de la structure. J’avais constaté aussi que leur laboratoire n’est pas bien équipé. C’est la raison qui a fait qu’on ne trouve pas la maladie ou soit leur laborantin n’est pas expérimenté sinon il pouvait référer l’enfant à temps.

**P13: Ktw_Hgr_Audit_dcd-08_Proche-2.doc - 13:25 [R : En commençant par le BCZS ..] (108:108) (Super)**

Codes: [Points_Positif-Négatif]

No memos

R : En commençant par le BCZS ils étaient expéditifs, le même jour que nous sommes arrivés aussitôt on passa aux examens de laboratoire, et nous donna un rendez-vous de revenir le lendemain avec un notre crachant pour réexaminer. Nous sommes revenus et les résultats étaient donnés à temps en disant qu’il n’avait pas la tuberculose pour moi je doutais en disant que l’enfant qui tousse beaucoup comme ça comment il n’a pas la tuberculose ? ils m’avaient dit que c’est une simple toux. On lui avait donné les comprimés bleus qui n’ont pas agi dans trois jours, j’étais obligé d’appeler les médecins de la riposte en disant que j’ai un malade son état de santé n’est pas bonne, ils arrivèrent on l’amena à Katwa où il y a une lenteur énorme et de fois ils nous disaient « EBOLA HAITUPATIYAKE FRANKA » Ebola ne nous donne pas de l’argent. C’est le langage qui était courant dans cette fosa.

**P 3: Bbo_HgrKttmba_Audit_dcd-01_Proche-3.doc - 3:40 [R : Oui nombreux préféraient y..] (158:158) (Super)**

Codes: [Pratiques_Soins]

No memos

R : Oui nombreux préféraient y aller comme on n’envoyait pas au CTE et on était bien traité mieux.

**P 8: Bbo_HgrKttmba_Audit_dcd-04_Proche-2.doc - 8:10 [R : Sept] (42:42) (Super)**

Codes: [Présentation_Défunt(e)]

No memos

R : Sept

**P 8: Bbo_HgrKttmba_Audit_dcd-04_Proche-2.doc - 8:9 [R : Elle avait 71ans.] (38:38) (Super)**

Codes: [Présentation_Défunt(e)]

No memos

R : Elle avait 71ans.

**P 8: Bbo_HgrKttmba_Audit_dcd-04_Proche-2.doc - 8:4 [R : Non elle n’était que culti..] (18:18) (Super)**

Codes: [Présentation_Défunt(e)]

No memos

R : Non elle n’était que cultivatrice, seulement qu’elle priait dans une église EERA

**P 9: Bbo_HopScola_Audit_dcd-07_Proche-1.doc - 9:1 [R : Il était cultivateur depui..] (5:5) (Super)**

Codes: [Présentation_Défunt(e)]

No memos

R : Il était cultivateur depuis sa jeunesse, il faisait une grande distance, il cultivait à Burondo en passant par Ruhwahwa. Ça fait Longtemps il cultiver à Burondo, il était toujours en bonne santé pendant des années, à son retour il amenait la nourriture pour les enfants, toujours sur la tête.

**P 9: Bbo_HopScola_Audit_dcd-07_Proche-1.doc - 9:4 [R : Non, le mari cultivait son..] (17:17) (Super)**

Codes: [Présentation_Défunt(e)]

No memos

R : Non, le mari cultivait son champ de sa famille et la femme aussi cultivait le sien de sa famille également, c’est comme ça qu’ils vivaient.

**P 9: Bbo_HopScola_Audit_dcd-07_Proche-1.doc - 9:3 [R : Sa femme cultivait un autr..] (13:13) (Super)**

Codes: [Présentation_Défunt(e)]

No memos

R : Sa femme cultivait un autre champ, ils ne cultivaient pas ensemble.

**P 9: Bbo_HopScola_Audit_dcd-07_Proche-1.doc - 9:2 [R : Il transportait la farine,..] (9:9) (Super)**

Codes: [Présentation_Défunt(e)]

No memos

R : Il transportait la farine, banane, colcase l’huile, toute la nourriture qu’il trouvait au champ, il transportait sur sa tête. Quand il a commencé à souffrir, nous membre de la famille avions remarqué qu’il souffrait du paludisme mais aussi la fatigue. Le fait de porter des choses sur la tête à son âge, ce n’était pas bon.

**P 8: Bbo_HgrKttmba_Audit_dcd-04_Proche-2.doc - 8:3 [R : Elle était cultivatrice] (14:14) (Super)**

Codes: [Présentation_Défunt(e)]

No memos

R : Elle était cultivatrice

**P 4: Bbo_HgrKttmba_Audit_dcd-03_Proche-1.doc - 4:2 [R : Elle était âgée de 46ans.] (13:13) (Super)**

Codes: [Présentation_Défunt(e)]

No memos

R : Elle était âgée de 46ans.

**P 5: Bbo_HgrKttmba_Audit_dcd-03_Proche-2.doc - 5:8 [R : Elle ne faisait rien meme ..] (31:31) (Super)**

Codes: [Présentation_Défunt(e)]

No memos

R : Elle ne faisait rien meme la maman la connaissait, elle passait son temps à la maison et de temps en temps elle se promenait dans les buvettes où on vendait la boisson alcoolique.

**P 7: Bbo_HgrKttmba_Audit_dcd-04_Proche-1.doc - 7:12 [R : Oui il est encore chez sa ..] (50:50) (Super)**

Codes: [Présentation_Défunt(e)]

No memos

R : Oui il est encore chez sa fille, vous allez lui voir

**P 5: Bbo_HgrKttmba_Audit_dcd-03_Proche-2.doc - 5:2 [R : Elle avait 42ans.] (7:7) (Super)**

Codes: [Présentation_Défunt(e)]

No memos

R : Elle avait 42ans.

**P13: Ktw_Hgr_Audit_dcd-08_Proche-2.doc - 13:5 [R : Il avait dix-sept ans.] (20:20) (Super)**

Codes: [Présentation_Défunt(e)]

No memos

R : Il avait dix-sept ans.

**P13: Ktw_Hgr_Audit_dcd-08_Proche-2.doc - 13:6 [R : Il était élève.] (24:24) (Super)**

Codes: [Présentation_Défunt(e)]

No memos

R : Il était élève.

**P 4: Bbo_HgrKttmba_Audit_dcd-03_Proche-1.doc - 4:3 [R : Elle était toujours maladi..] (17:17) (Super)**

Codes: [Présentation_Défunt(e)]

No memos

R : Elle était toujours maladive

**P 9: Bbo_HopScola_Audit_dcd-07_Proche-1.doc - 9:5 [R : Non, elle cultive toujours..] (22:22) (Super)**

Codes: [Présentation_Défunt(e)]

No memos

R : Non, elle cultive toujours chez elle, le champ qu’elle cultivait, elle n’a pas encore commencé à cultiver le champ que son mari lui a laissé.

**P 9: Bbo_HopScola_Audit_dcd-07_Proche-1.doc - 9:8 [R : Il n’avait pas d’autres ac..] (34:34) (Super)**

Codes: [Présentation_Défunt(e)]

No memos

R : Il n’avait pas d’autres activités à part cultiver.

**P16: Ktw_HopMtda_Audit_dcd-05_Proche-2.doc - 16:3 [R. Au total, elle avait huit e..] (13:13) (Super)**

Codes: [Présentation_Défunt(e)]

No memos

R. Au total, elle avait huit enfants (8)

**P 4: Bbo_HgrKttmba_Audit_dcd-03_Proche-1.doc - 4:7 [R : elle vendait de denrées al..] (33:33) (Super)**

Codes: [Présentation_Défunt(e)]

No memos

R : elle vendait de denrées alimentaires

**P 9: Bbo_HopScola_Audit_dcd-07_Proche-1.doc - 9:9 [R : Il vivait biens avec les g..] (38:38) (Super)**

Codes: [Présentation_Défunt(e)]

No memos

R : Il vivait biens avec les gens la famille parce qu’il était sage de l’Église.

**P 4: Bbo_HgrKttmba_Audit_dcd-03_Proche-1.doc - 4:8 [R : Elle revendait du légume.] (37:37) (Super)**

Codes: [Présentation_Défunt(e)]

No memos

R : Elle revendait du légume.

**P 4: Bbo_HgrKttmba_Audit_dcd-03_Proche-1.doc - 4:9 [R : Non elle n’était pas marié..] (41:41) (Super)**

Codes: [Présentation_Défunt(e)]

No memos

R : Non elle n’était pas mariée mais elle mettait au monde sans mari et elle était sociale envers les gens.

**P 9: Bbo_HopScola_Audit_dcd-07_Proche-1.doc - 9:10 [R : Non il était sage de l’Egl..] (42:42) (Super)**

Codes: [Présentation_Défunt(e)]

No memos

R : Non il était sage de l’Eglise CECA20 mais pas pasteur, c’est comme un conseiller du pasteur.

**P16: Ktw_HopMtda_Audit_dcd-05_Proche-2.doc - 16:4 [R. Non] (17:17) (Super)**

Codes: [Présentation_Défunt(e)]

No memos

R. Non

**P 4: Bbo_HgrKttmba_Audit_dcd-03_Proche-1.doc - 4:4 [R : Elle avait trois enfants.] (21:21) (Super)**

Codes: [Présentation_Défunt(e)]

No memos

R : Elle avait trois enfants.

**P 9: Bbo_HopScola_Audit_dcd-07_Proche-1.doc - 9:7 [R : Il y a trois filles et tro..] (30:30) (Super)**

Codes: [Présentation_Défunt(e)]

No memos

R : Il y a trois filles et trois garçons.

**P 9: Bbo_HopScola_Audit_dcd-07_Proche-1.doc - 9:6 [R : Ils sont au nombre de six,..] (26:26) (Super)**

Codes: [Présentation_Défunt(e)]

No memos

R : Ils sont au nombre de six, et ils allaient toujours avec leur mère là où elle cultivait.

**P16: Ktw_HopMtda_Audit_dcd-05_Proche-2.doc - 16:8 [R. Elle se comportait vraiment..] (33:33) (Super)**

Codes: [Présentation_Défunt(e)]

No memos

R. Elle se comportait vraiment bien. Elle venait nous rendre visite ici à la maison.

**P16: Ktw_HopMtda_Audit_dcd-05_Proche-2.doc - 16:5 [R. Cinq (5)] (21:21) (Super)**

Codes: [Présentation_Défunt(e)]

No memos

R. Cinq (5)

**P16: Ktw_HopMtda_Audit_dcd-05_Proche-2.doc - 16:6 [R. Elle vendait de la boisson] (25:25) (Super)**

Codes: [Présentation_Défunt(e)]

No memos

R. Elle vendait de la boisson

**P16: Ktw_HopMtda_Audit_dcd-05_Proche-2.doc - 16:7 [R. « Kindingi » (boisson tradi..] (29:29) (Super)**

Codes: [Présentation_Défunt(e)]

No memos

R. « Kindingi » (boisson traditionnelle)

**P 7: Bbo_HgrKttmba_Audit_dcd-04_Proche-1.doc - 7:11 [R : C’est là où elle était inh..] (46:46) (Super)**

Codes: [Présentation_Défunt(e)]

No memos

R : C’est là où elle était inhumée.

**P 6: Bbo_HgrKttmba_Audit_dcd-03_Proche-3.doc - 6:5 [R : Elle et moi prenions de l’..] (17:17) (Super)**

Codes: [Présentation_Défunt(e)]

No memos

R : Elle et moi prenions de l’alcool, la viande et le poisson frais.

**P 6: Bbo_HgrKttmba_Audit_dcd-03_Proche-3.doc - 6:6 [R : Sa famille je ne maitrise ..] (21:21) (Super)**

Codes: [Présentation_Défunt(e)]

No memos

R : Sa famille je ne maitrise pas bien mais je sais que sa grande sœur vit seule et elle-même avait connu une séparation et elle était venue rester avec sa grande sœur.

**P 6: Bbo_HgrKttmba_Audit_dcd-03_Proche-3.doc - 6:2 [R : Elle avait 48 à 50ans vers..] (9:9) (Super)**

Codes: [Présentation_Défunt(e)]

No memos

R : Elle avait 48 à 50ans vers là je ne pas de précision comme moi j’ai déjà 50ans on a grandi ensemble, on ne sait pas qui est grande et on ne demandait pas l’âge de chacune de nous.

**P 6: Bbo_HgrKttmba_Audit_dcd-03_Proche-3.doc - 6:4 [R : De fois elle travaillait d..] (13:13) (Super)**

Codes: [Présentation_Défunt(e)]

No memos

R : De fois elle travaillait dans un restaurant et parfois elle faisait l’agriculture.

**P 6: Bbo_HgrKttmba_Audit_dcd-03_Proche-3.doc - 6:9 [R : Oui elles sont nées ici et..] (33:33) (Super)**

Codes: [Présentation_Défunt(e)]

No memos

R : Oui elles sont nées ici et sont de grandes filles actuellement

**P 6: Bbo_HgrKttmba_Audit_dcd-03_Proche-3.doc - 6:10 [R : Elle était très bonne, cha..] (37:37) (Super)**

Codes: [Présentation_Défunt(e)]

No memos

R : Elle était très bonne, charmante envers tout le monde.

**P 6: Bbo_HgrKttmba_Audit_dcd-03_Proche-3.doc - 6:7 [R : A ma connaissance c’est de..] (25:25) (Super)**

Codes: [Présentation_Défunt(e)]

No memos

R : A ma connaissance c’est deux filles mais ceux qu’elle avait mis au monde avec son mari ils étaient retournés chez leurs père et je ne les connais pas.

**P 6: Bbo_HgrKttmba_Audit_dcd-03_Proche-3.doc - 6:8 [Q : Là je ne sais pas s’elle a..] (29:29) (Super)**

Codes: [Présentation_Défunt(e)]

No memos

**Q : Là je ne sais pas s’elle avait laissé combien d’enfant. Vous savez quand** **on se mari il y a un grand moment de séparation. Lors ce qu’elle revenue on** n’avait pas parlé sur ça.

**P18: Ktw_HopMtda_Audit_dcd-06_Proche-1.doc - 18:7 [R. Elle avait 5 enfants.] (25:25) (Super)**

Codes: [Présentation_Défunt(e)]

No memos

R. Elle avait 5 enfants.

**P18: Ktw_HopMtda_Audit_dcd-06_Proche-1.doc - 18:6 [R. Elle mangeait comme d’habit..] (21:21) (Super)**

Codes: [Présentation_Défunt(e)]

No memos

R. Elle mangeait comme d’habitude

**P18: Ktw_HopMtda_Audit_dcd-06_Proche-1.doc - 18:9 [R. C’est un lieu de prière, c’..] (33:33) (Super)**

Codes: [Présentation_Défunt(e)]

No memos

R. C’est un lieu de prière, c’est une petite église pour le catholique

**P18: Ktw_HopMtda_Audit_dcd-06_Proche-1.doc - 18:8 [R. Elle était accueillante, el..] (29:29) (Super)**

Codes: [Présentation_Défunt(e)]

No memos

R. Elle était accueillante, elle vivait bien avec tout le monde. Nous la regrettons. Elle fut notre Mama présidente de notre succursale catholique(Chapelle).

**P 5: Bbo_HgrKttmba_Audit_dcd-03_Proche-2.doc - 5:79 [R : Elle était mariée mais plu..] (55:55) (Super)**

Codes: [Présentation_Défunt(e)]

No memos

R : Elle était mariée mais plus tard ils se sont séparés avant par ce que le mari l’avait abandonné et c’ainsi qu’elle vivotait seule sans mari.

**P 6: Bbo_HgrKttmba_Audit_dcd-03_Proche-3.doc - 6:1 [R : Consolée était mon amie no..] (5:5) (Super)**

Codes: [Présentation_Défunt(e)]

No memos

R : Consolée était mon amie nous avons grandi ensemble presque meme age dans la cellule jusqu’à ce qu’on est devenu toutes de mamans.et puis elle était malade et hospitaliser au poste de santé chez Kapitula après trois jours son état de santé n’était pas bon et on l’amena à Kitatumba y arriva, on y avait rencontré leurs systèmes de traitement qui dit que pour recevoir un patient il doit passer par le CTE et apporter le résultat pour commencer le traitement. C’est ainsi qu’on lui envoya au CTE où elle passa trois jours sans changement. Et puis elle retourna à Kitatumba pour le traitement où elle décéda.

**P18: Ktw_HopMtda_Audit_dcd-06_Proche-1.doc - 18:5 [R. C’est cinquante-sept ans (5..] (13:13) (Super)**

Codes: [Présentation_Défunt(e)]

No memos

R. C’est cinquante-sept ans (57 ans).

**P18: Ktw_HopMtda_Audit_dcd-06_Proche-1.doc - 18:4 [R. Elle partait acheter des po..] (17:17) (Super)**

Codes: [Présentation_Défunt(e)]

No memos

R. Elle partait acheter des poissons à Kasindi qu’elle revendait au quartier.

**P 5: Bbo_HgrKttmba_Audit_dcd-03_Proche-2.doc - 5:18 [R : Non et ne faisait rien mai..] (71:71) (Super)**

Codes: [Présentation_Défunt(e)]

No memos

R : Non et ne faisait rien mais quelque fois elle vendait les morceaux des cannes à sucre à la maison, parce qu’était faible elle n’avait pas la force de faire autres choses.

**P17: Ktw_HopMtda_Audit_dcd-05_Proche-3.doc - 17:1 [R. Pour la vie de Justine, ell..] (5:5) (Super)**

Codes: [Présentation_Défunt(e)]

No memos

R. Pour la vie de Justine, elle venait de la ville puis elle avait trébuché. Le fait de trébucher avait occasionné une fracture au niveau de la jambe. Il fallait suivre les soins à Kitatumba. Deux semaines après, elle était transférée à Kalungamakuha où elle a fait trois mois de soins sans succès. Lorsqu’on a trouvé qu’elle était gravement malade, elle avait été transférées à Matanda où nous avons fait deux semaines puis elle est décédée.

**P 7: Bbo_HgrKttmba_Audit_dcd-04_Proche-1.doc - 7:1 [R : Suis l’amie de sa fille no..] (6:6) (Super)**

Codes: [Présentation_Défunt(e)]

No memos

R : Suis l’amie de sa fille nous vendons ensemble elle vivait avec Madeleine la défunte qui souffrait du diabète pendant longtemps qui avait bloqué sa vision et plu tard on l’amena à l’hôpital ou elle sera orientée vers l’urgence ou Soins intensifs.

**P17: Ktw_HopMtda_Audit_dcd-05_Proche-3.doc - 17:3 [R. Elle vendait de la boisson ..] (13:13) (Super)**

Codes: [Présentation_Défunt(e)]

No memos

R. Elle vendait de la boisson en ville

**P17: Ktw_HopMtda_Audit_dcd-05_Proche-3.doc - 17:2 [R. Elle a 63 ans.] (9:9) (Super)**

Codes: [Présentation_Défunt(e)]

No memos

R. Elle a 63 ans.

**P 7: Bbo_HgrKttmba_Audit_dcd-04_Proche-1.doc - 7:4 [R : Non ça je ne connaissais p..] (18:18) (Super)**

Codes: [Présentation_Défunt(e)]

No memos

R : Non ça je ne connaissais pas

**P 7: Bbo_HgrKttmba_Audit_dcd-04_Proche-1.doc - 7:10 [R : Oui, il est toujours en vi..] (42:42) (Super)**

Codes: [Présentation_Défunt(e)]

No memos

R : Oui, il est toujours en vie.

**P 7: Bbo_HgrKttmba_Audit_dcd-04_Proche-1.doc - 7:2 [R : Je ne peux pas connaitre p..] (10:10) (Super)**

Codes: [Présentation_Défunt(e)]

No memos

R : Je ne peux pas connaitre par ce qu’elle était venue pour les soins et même ses enfants je les avais vu aux obsèques.

**P 7: Bbo_HgrKttmba_Audit_dcd-04_Proche-1.doc - 7:3 [R : Je ne m’intéressais pas à ..] (14:14) (Super)**

Codes: [Présentation_Défunt(e)]

No memos

R : Je ne m’intéressais pas à cela.

**P 5: Bbo_HgrKttmba_Audit_dcd-03_Proche-2.doc - 5:15 [R : Il y a trois naissances qu..] (59:59) (Super)**

Codes: [Présentation_Défunt(e)]

No memos

R : Il y a trois naissances qu’elle avait accouchée en dehors de son mari et le autres avec son mari.

**P 5: Bbo_HgrKttmba_Audit_dcd-03_Proche-2.doc - 5:13 [R : Elle avait six naissances ..] (51:51) (Super)**

Codes: [Présentation_Défunt(e)]

No memos

R : Elle avait six naissances mais c’est cinq qui sont en vie.

**P 5: Bbo_HgrKttmba_Audit_dcd-03_Proche-2.doc - 5:17 [R : Oui, elle ne faisait pas a..] (67:67) (Super)**

Codes: [Présentation_Défunt(e)]

No memos

R : Oui, elle ne faisait pas aucune activité

**P 5: Bbo_HgrKttmba_Audit_dcd-03_Proche-2.doc - 5:16 [R : Malgré sa maladie elle les..] (63:63) (Super)**

Codes: [Présentation_Défunt(e)]

No memos

R : Malgré sa maladie elle les nourrissait ; parfois elle envoyait un enfant pour aller acheter les cannes à sucre chez les cultivateurs et le revendre encore. C’est dans cette façon qu’ils mangeaient.

**P17: Ktw_HopMtda_Audit_dcd-05_Proche-3.doc - 17:5 [R. Concernant sa vie social ou..] (21:21) (Super)**

Codes: [Présentation_Défunt(e)]

No memos

R. Concernant sa vie social ou avec ses amis, je ne peux pas le connaitre car je l’avais gardée seulement à l’hôpital.

**P17: Ktw_HopMtda_Audit_dcd-05_Proche-3.doc - 17:4 [R. Elle avait 7 enfants] (17:17) (Super)**

Codes: [Présentation_Défunt(e)]

No memos

R. Elle avait 7 enfants

**P13: Ktw_Hgr_Audit_dcd-08_Proche-2.doc - 13:7 [R : Souvent il ne réussissait ..] (28:28) (Super)**

Codes: [Présentation_Défunt(e)]

No memos

R : Souvent il ne réussissait pas à l’école, il était en deuxième année secondaire.

**P 5: Bbo_HgrKttmba_Audit_dcd-03_Proche-2.doc - 5:9 [R : Je ne sais pas mais elle f..] (35:35) (Super)**

Codes: [Présentation_Défunt(e)]

No memos

R : Je ne sais pas mais elle fréquentait ces gens-là. Sa maladie était toujours l’épilepsie.

**P14: Ktw_Hgr_Audit_dcd-09_Proche-1.doc - 14:1 [R : Dans sa vie il était motar..] (5:5) (Super)**

Codes: [Présentation_Défunt(e)]

No memos

R : Dans sa vie il était motard il faisait le taxi depuis sa jeunesse c’était son travail habitué. Il était parti faire le taxi à Mungwalu dans la province de l’Ituri et au retour comme sa femme était enceinte elle avait accouchée. Trois semaines après l’enfant décéda et dès lors son état de santé n’était plus bon, comme c’était dans une période de la MVE nous étions obligés de l’amener au CTE Chirimabolyo pour les examens ; ici on nous avait accueilli très bien pas de reproche. Après là nous sommes allés dans l’hôpital Katwa où on nous avait accueilli aussi et nous avions dit aux prestataires qu’il avait un problème de ventre qu’il serait mieux de passer à l’échographie les prestataires des soins n’avaient pas accepter, sept jours on nous donna la sortie pour rentrer à la maison. Et puis comme c’était la période de gratuité ils nous avaient dit qu’il faut payer 20 dollars pour les examens faits. Après les examens ils nous diront qu’il souffre du paludisme et la gastrite. Ils nous donnèrent le traitement et nous étions rentré à la maison mais il n’y avait pas d’évolution. Quelques jours après il sera gravement malade et il retourna à Katwa encore pour les soins, il été reçu dans les soins intensifs où il passa six jours et on retourna dans la salle et le lendemain et nous dira de rentrer à la maison par ce qu’il souffre du cancer de foie. Nous leurs avons dit que pour notre première hospitalisation nous vous avion demandé qu’il puisse passer à l’échographie pour mieux diagnostiquer sa pathologie vous n’aviez pas accepter ? ils nous répondirent de retourner à la maison pour y attendre la volonté divine. Nous étions retournés à la maison et dix jour après il nous quitta.

**P 2: Bbo_HgrKttmba_Audit_dcd-01_Proche-2.doc - 2:41 [R : Nous étions nombreux qui é..] (169:169) (Super)**

Codes: [Présentation_Défunt(e)]

No memos

R : Nous étions nombreux qui étaient sous sa responsabilité et il avait le souci de pouvoir nous laisser étant stables dans la vie.

**P11: Ktw_Hgr_Audit_dcd-08_Proche-1.doc - 11:6 [R : Oui, il était connu par to..] (25:25) (Super)**

Codes: [Présentation_Défunt(e)]

No memos

R : Oui, il était connu par tout le monde même si vous demandez à quelqu’un chez Kasereka on va vous orienter ; il était dynamique et celui d’ailleurs qui faisait trop des travaux dans la maison que les autres enfants.

**P15: Ktw_HopMtda_Audit_dcd-05_Proche-1.doc - 15:10 [R. Elle pratiquait de l’agricu..] (42:42) (Super)**

Codes: [Présentation_Défunt(e)]

No memos

R. Elle pratiquait de l’agriculture

**P20: Ktw_HopMtda_Audit_dcd-11_Proche-1.doc - 20:3 [R : elle la quatrième naissanc..] (19:19) (Super)**

Codes: [Présentation_Défunt(e)]

No memos

R : elle la quatrième naissanc

**P20: Ktw_HopMtda_Audit_dcd-11_Proche-1.doc - 20:2 [R : Elle avait 8 ans et elle é..] (15:15) (Super)**

Codes: [Présentation_Défunt(e)]

No memos

R : Elle avait 8 ans et elle était en troisième année primaire

**P11: Ktw_Hgr_Audit_dcd-08_Proche-1.doc - 11:2 [R : il se promener avec la pès..] (9:9) (Super)**

Codes: [Présentation_Défunt(e)]

No memos

R : il se promener avec la pèse pour peser les poids des gens.

**P11: Ktw_Hgr_Audit_dcd-08_Proche-1.doc - 11:1 [R : Il était ambulant de la pè..] (5:5) (Super)**

Codes: [Présentation_Défunt(e)]

No memos

R : Il était ambulant de la pèse

**P19: Ktw_HopMtda_Audit_dcd-10_Proche-1.doc - 19:29 [R : Elle vivait chez nous, au ..] (139:139) (Super)**

Codes: [Présentation_Défunt(e)]

No memos

R : Elle vivait chez nous, au centre-ville.

**P11: Ktw_Hgr_Audit_dcd-08_Proche-1.doc - 11:5 [R : il nourrissait les chèvres..] (21:21) (Super)**

Codes: [Présentation_Défunt(e)]

No memos

R : il nourrissait les chèvres et chercher les herbes pour les lapins.

**P11: Ktw_Hgr_Audit_dcd-08_Proche-1.doc - 11:4 [R :il était mon troisième garç..] (17:17) (Super)**

Codes: [Présentation_Défunt(e)]

No memos

R :il était mon troisième garçon et sixième de ma famille.

**P11: Ktw_Hgr_Audit_dcd-08_Proche-1.doc - 11:3 [R : il avait déjà fait deux an..] (13:13) (Super)**

Codes: [Présentation_Défunt(e)]

No memos

R : il avait déjà fait deux ans.

**P15: Ktw_HopMtda_Audit_dcd-05_Proche-1.doc - 15:9 [R. Dans l’église KITATUMBA(cha..] (38:38) (Super)**

Codes: [Présentation_Défunt(e)]

No memos

R. Dans l’église KITATUMBA(chapelle)

**P12: Ktw_Hgr_Audit_dcd-08_Proche-12.doc - 12:3 [R : elle ne faisait que cultiv..] (13:13) (Super)**

Codes: [Présentation_Défunt(e)]

No memos

R : elle ne faisait que cultiver, rien d’autre.

**P12: Ktw_Hgr_Audit_dcd-08_Proche-12.doc - 12:2 [R : Elle avait septante deux a..] (9:9) (Super)**

Codes: [Présentation_Défunt(e)]

No memos

R : Elle avait septante deux ans(72ans).

**P12: Ktw_Hgr_Audit_dcd-08_Proche-12.doc - 12:1 [R : Dans sa vie, elle ne faisa..] (5:5) (Super)**

Codes: [Présentation_Défunt(e)]

No memos

R : Dans sa vie, elle ne faisait que l’agriculture.

**P 1: Bbo_HgrKttmba_Audit_dcd-01_Proche-1.doc - 1:5 [RÉPONDANT : Avant il vivait ch..] (20:20) (Super)**

Codes: [Présentation_Défunt(e)]

No memos

RÉPONDANT : Avant il vivait chez moi et je l’avais gardé pendant cinq ans, un peu plus tard il retourna chez lui comme sa parcelle avait un problème on avait jugé bon qu’il retourne chez lui.

**P12: Ktw_Hgr_Audit_dcd-08_Proche-12.doc - 12:5 [R : Elle n’avait pas des probl..] (21:21) (Super)**

Codes: [Présentation_Défunt(e)]

No memos

R : Elle n’avait pas des problèmes avec les gens.

**P12: Ktw_Hgr_Audit_dcd-08_Proche-12.doc - 12:4 [R : Elle avait dix enfants don..] (17:17) (Super)**

Codes: [Présentation_Défunt(e)]

No memos

R : Elle avait dix enfants dont deux sont décédés.

**P15: Ktw_HopMtda_Audit_dcd-05_Proche-1.doc - 15:6 [R. Elle vendait de la boisson] (26:26) (Super)**

Codes: [Présentation_Défunt(e)]

No memos

R. Elle vendait de la boisson

**P15: Ktw_HopMtda_Audit_dcd-05_Proche-1.doc - 15:7 [R. Elle vendait de la bière Pr..] (30:30) (Super)**

Codes: [Présentation_Défunt(e)]

No memos

R. Elle vendait de la bière Primus et de la boisson traditionnelle « Rutuku »

**P15: Ktw_HopMtda_Audit_dcd-05_Proche-1.doc - 15:8 [R. Elle était chantre] (34:34) (Super)**

Codes: [Présentation_Défunt(e)]

No memos

R. Elle était chantre

**P15: Ktw_HopMtda_Audit_dcd-05_Proche-1.doc - 15:3 [R. Il vivait vraiment bien.] (13:13) (Super)**

Codes: [Présentation_Défunt(e)]

No memos

R. Il vivait vraiment bien.

**P15: Ktw_HopMtda_Audit_dcd-05_Proche-1.doc - 15:4 [R. Elle avait cinq naissances...] (17:17) (Super)**

Codes: [Présentation_Défunt(e)]

No memos

R. Elle avait cinq naissances. Tu sais, son mari qui était décédé avait aussi ses trois enfants avec une autre femme ; Mais ses enfants propres sont au nombre de cinq et dans l’ensemble c’est 8

**P15: Ktw_HopMtda_Audit_dcd-05_Proche-1.doc - 15:5 [R. Entre 54 ou 55 ans ; Je ne ..] (20:20) (Super)**

Codes: [Présentation_Défunt(e)]

No memos

R. Entre 54 ou 55 ans ; Je ne me souviens pas exactement. Mais elle n’a pas encore fait beaucoup de temps qu’elle est morte. Il y a de cela deux mois ou un mois et demi.

**P19: Ktw_HopMtda_Audit_dcd-10_Proche-1.doc - 19:6 [R : Elle était en bonne relati..] (29:29) (Super)**

Codes: [Présentation_Défunt(e)]

No memos

R : Elle était en bonne relation avec tout le monde, elle était trop gentille, très sociable,

**P10: Ktw_Hgr_Audit_dcd-02_Proche-1.doc - 10:2 [R : Jadis il était commerçant ..] (9:9) (Super)**

Codes: [Présentation_Défunt(e)]

No memos

R : Jadis il était commerçant puis il était retourné dans l’agriculture et parfois il faisait de petit commerce sur vélo en transportant les poissons il avait fait beaucoup d’activité et surtout les activités de fatigue.

**P10: Ktw_Hgr_Audit_dcd-02_Proche-1.doc - 10:3 [R : Il nous a quitté à l’âge d..] (13:13) (Super)**

Codes: [Présentation_Défunt(e)]

No memos

R : Il nous a quitté à l’âge de 75 ans.

**P10: Ktw_Hgr_Audit_dcd-02_Proche-1.doc - 10:4 [R : Au niveau de la famille il..] (17:17) (Super)**

Codes: [Présentation_Défunt(e)]

No memos

R : Au niveau de la famille il avait neuf enfant dont sept en vie et deux morts.

**P10: Ktw_Hgr_Audit_dcd-02_Proche-1.doc - 10:5 [R : Il était gentil envers tou..] (21:21) (Super)**

Codes: [Présentation_Défunt(e)]

No memos

R : Il était gentil envers tout le monde, il n’avait pas de problème avec son entourage et sur sa famille comme c’est lui qui était le responsable de la famille, sur lui tout le monde avait confiance en lui. C’est qui m’avait fort marqué est que pendant ses sept dernières années il s’était converti dans la vie spirituelle où il avait commencé à nous enseigné la parole de Dieu. J’ai compris qu’il ne voulait pas mourir et laisser ses enfants non croyants.

**P19: Ktw_HopMtda_Audit_dcd-10_Proche-1.doc - 19:5 [R : Elle n’avait pas encore mi..] (25:25) (Super)**

Codes: [Présentation_Défunt(e)]

No memos

R : Elle n’avait pas encore mis au monde un enfant.

**P13: Ktw_Hgr_Audit_dcd-08_Proche-2.doc - 13:1 [R : L’enfant est né à Banalia,..] (3:3) (Super)**

Codes: [Présentation_Défunt(e)]

No memos

R : L’enfant est né à Banalia, a cent vingt kilomètre (120km) de Kisangani dans la province orientale vers l’axe Buta. J’y travaillais avec mon petit frère. Après un temps, il a eu la maladie de tuberculose, heureusement que j’avais remarqué très tôt, on l’amena dans le grand hôpital de Banalia, il avait été soigne et guéri. Il est vivant jusqu'à maintenant. Et c’est lui qui veillait sur l’enfant qui était né. On quitta à Banalia quand l’enfant avait quatre ans. A dix ans, on remarqua une toux accentuée qui ne s’arrêtait pas chez l’enfant. C’est ainsi qu’on l’amena au centre de santé pour faire les examens, et après les soignant nous diront qu’il n’a rien. Mais après une longue durée, on remarqua qu’il présente les signes tels que la démangeaison de la peau. J’avais vite remarque que leur laborantin n’était pas vraiment qualifié, Parfois j’y suis les soins et quelque fois je vois qu’il reverse les échantillons de patient. Beaucoup de malades n’y fréquentent pas en disant qu’il n’est pas qualifié. Alors je décidais d’aller au bureau de la zone de sante parce que là, il y’a des spécialistes expérimentés pour soigner la tuberculose et autre. On y arriva, et l’enfant avait fait les examens. On nous dira qu’après deux jours on passera pour le résultat, on a eu le résultat montrant que l’enfant n’a pas la tuberculose et je les avais expliqués toute la situation que j’avais un petit frère qui avait souffert de la tuberculose, on lui donnant les **médicaments pour trois jours. Le troisième jour, l’enfant sera gravement** malade, on l’amena de nouveau au même Centre de santé. Y arriva, les soignants nous avaient dit cet enfant n’avait pas la maladie et qu’ils étaient incapables de soigner ce cas, c’est ainsi que j’avais appelé le docteur de riposte. Ils arrivèrent, et partirent avec l’enfant au CTE KATWA. Au CTE le patient était observé pendant deux jours, ils avaient remarqué que l’enfant n’a pas la maladie d’Ebola. Eux même l’amenèrent à l’hôpital Katwa. Un jour le médecin me dira qu’il ne comprend pas de quelle maladie souffre l’enfant. Il y’a des fois où il disait que c’était de la tuberculose osseuse, d’autres fois ils disaient que c’était du poison, on ne savait pas de quoi l’enfant soufrait-il ? On l’amena la radio mais pas de diagnostic. On est resté à l’hôpital l’enfant souffre et maigris mais pas d’évolution. Lorsqu’il est devenu agonissant étant dans les soins intensifs, ils avaient prescrit le même médicament de la tuberculose qu’on lui donnait à 5hoo du matin. On avait fait un mois dans les soins intensifs, l’enfant finira par décéder on ne savait pas si c’était quoi et ainsi que nous y avons quitté.

**P10: Ktw_Hgr_Audit_dcd-02_Proche-1.doc - 10:1 [R : La personne qui nous a qui..] (5:5) (Super)**

Codes: [Présentation_Défunt(e)]

No memos

R : La personne qui nous a quittée s’appelait KAKULE KAHYANA JOSEPH, c’est mon père biologique ; c’est moi qui s’occupait de lui pendant qu’il était à la maison et même pendant qu’il était malade hospitalisé

**P19: Ktw_HopMtda_Audit_dcd-10_Proche-1.doc - 19:3 [R : Pas d’autres activité qu’e..] (17:17) (Super)**

Codes: [Présentation_Défunt(e)]

No memos

R : Pas d’autres activité qu’elle faisait à part la vente de produits pharmaceutiques.

**P19: Ktw_HopMtda_Audit_dcd-10_Proche-1.doc - 19:4 [R : Elle préférait plus l’alco..] (21:21) (Super)**

Codes: [Présentation_Défunt(e)]

No memos

R : Elle préférait plus l’alcool.

**P19: Ktw_HopMtda_Audit_dcd-10_Proche-1.doc - 19:1 [R : Elle travaillait dans une ..] (9:9) (Super)**

Codes: [Présentation_Défunt(e)]

No memos

R : Elle travaillait dans une pharmacie, c’est elle qui avait la responsabilité des enfants comme les parents étaient déjà tous morts. Après un temps elle fut engrossée par un homme non connu et cette dernière avait des difficultés d’évoluer à cause du fibrome. C’est ainsi qu’elle déclara qu’elle était malade et on l’amena à l’hôpital des anglicans. Après quelques d’hospitalisation elle sortit et nous avions crus que ce fibrome était traité et guéris, on retourna à la maison. Elle avorta, et les médecins nous diront que c’était à cause du fibrome. Après, elle fut enceinte pour la deuxième fois par le même homme. Quand la grossesse atteignit le sixième mois, encore elle eut encore le même problème du fibrome. C’est ainsi qu’on amena à Matanda. Une fois arrivé à Matanda, les médecins avaient remarqué qu’il fallait l’opérer car l’enfant était déjà mort. Et pendant les réanimations, elle avait fait presque deux heures, elle aussi mourra.

**P 4: Bbo_HgrKttmba_Audit_dcd-03_Proche-1.doc - 4:25 [R : Oui elle y avait passé la ..] (105:105) (Super)**

Codes: [Prise en charge_Etapes l'hôpital]

No memos

R : Oui elle y avait passé la nuit.

**P 6: Bbo_HgrKttmba_Audit_dcd-03_Proche-3.doc - 6:28 [R : Il y avait une place où le..] (111:111) (Super)**

Codes: [Prise en charge_Etapes l'hôpital]

No memos

R : Il y avait une place où les agents de la riposte occupaient et qui étaient jadis une réception pour l’hôpital. Lorsque vous y arrivaient on vous dit de patienter, par après ils appellent leur ambulance et on vous embarque jusqu’au CTE. Sans que vous soyez en contact avec les soignant de Kitatumba.

**P 6: Bbo_HgrKttmba_Audit_dcd-03_Proche-3.doc - 6:27 [R : Oui] (107:107) (Super)**

Codes: [Prise en charge_Etapes l'hôpital]

No memos

R : Oui

**P 6: Bbo_HgrKttmba_Audit_dcd-03_Proche-3.doc - 6:25 [R : J’étais au Centre de Santé..] (98:98) (Super)**

Codes: [Prise en charge_Etapes l'hôpital]

No memos

R : J’étais au Centre de Santé on me dira d’aller à Kitatumba par ce que je venais de prendre deux cure sans soulagement. En y arriva on m’avait arrêté à la porte d’entrée que je dois aller au CTE, C’était environs 13hoo on nous avait amené au CTE à 17hoo et là on nous dira de patienter jusqu’à demain en me donnant 3 comprimés que j’avais pris à la même heure d’arriver. On m’avait prélevé à 7hoo et les résultats étaient sortis à 14hoo on nous dira d’attendre le deuxième prélèvement et depuis le 1^er^ prélèvement jusqu’au 2^ème^ on ne m’avait plus donné aucun médicament. il y avait eu beaucoup de malades qui étaient venus, ça fait qu’on me libère sans que je ne fasse le deuxième prélèvement et sans traitement à part ces trois comprimés que j’avais reçu à l’entrée. J’ai pensé que ça été le cas de Consolée qui a fait les trois jours sans aucun traitement. Si vous n’êtes pas un cas positif d’Ebola on ne donne pas le médicament malgré la gravité de la pathologie. C’est ainsi qu’elle deviendra gravement malade suite au retard de traitement et lorsqu’elle était arrivé à Kitatumba elle décéda dans une courte durée.

**P 6: Bbo_HgrKttmba_Audit_dcd-03_Proche-3.doc - 6:24 [R : Elle y avait fait trois jo..] (94:94) (Super)**

Codes: [Prise en charge_Etapes l'hôpital]

No memos

R : Elle y avait fait trois jours au CTE.

**P 2: Bbo_HgrKttmba_Audit_dcd-01_Proche-2.doc - 2:16 [R : On lui avait reçu et ne lu..] (69:69) (Super)**

Codes: [Prise en charge_Etapes l'hôpital]

No memos

R : On lui avait reçu et ne lui avait pas envoyé au CTE mais vous remarquez qu’ils ne s’occupaient pas de malade on leurs laissait là à cause de la peur de la MVE.

**P 2: Bbo_HgrKttmba_Audit_dcd-01_Proche-2.doc - 2:20 [R : Oui il était passé par là...] (85:85) (Super)**

Codes: [Prise en charge_Etapes l'hôpital]

No memos

R : Oui il était passé par là.

**P 4: Bbo_HgrKttmba_Audit_dcd-03_Proche-1.doc - 4:47 [R : La structure est bonne ave..] (192:192) (Super)**

Codes: [Prise en charge_Etapes l'hôpital]

No memos

R : La structure est bonne avec ses services, chacun est reçu dans chaque service

**P 4: Bbo_HgrKttmba_Audit_dcd-03_Proche-1.doc - 4:24 [R : OUI il y avait d’autres ma..] (101:101) (Super)**

Codes: [Prise en charge_Etapes l'hôpital]

No memos

R : OUI il y avait d’autres malades qui y étaient et c’était dans une période où on ne pouvait pas passer deux jours sans vous amener(BEBA) au CTE.

**P 9: Bbo_HopScola_Audit_dcd-07_Proche-1.doc - 9:25 [R : La prise en charge était b..] (113:113) (Super)**

Codes: [Prise en charge_Médicale]

No memos

R : La prise en charge était bonne, les soignants ont fait tout ce qu’ils pouvaient, car il y a des temps en temps que j’y arrivais je lui trouvais assis collaborant, mais un jour on était surpris d’entendre qu’il était décédé.

**P 9: Bbo_HopScola_Audit_dcd-07_Proche-1.doc - 9:28 [R : Oui, ils disaient aussi qu..] (128:128) (Super)**

Codes: [Prise en charge_Médicale]

No memos

R : Oui, ils disaient aussi qu’il sera bien et il n’y a pas nécessité de lui transférer dans une autre FOSA, car on demandait aux médecins le transfert s’ils voient que les choses n’évoluent pas.

**P18: Ktw_HopMtda_Audit_dcd-06_Proche-1.doc - 18:66 [R. Je voyais que les prestatai..] (275:275) (Super)**

Codes: [Prise en charge_Médicale]

No memos

R. Je voyais que les prestataires étaient souples dans la surveillance des malades ; seulement que lorsqu’on n’est pas soignant, on ne sait pas évaluer la qualité des soins, lorsqu’ils veulent soigner les patients, on demande aux visiteurs d’aller à l’extérieur.

**P 6: Bbo_HgrKttmba_Audit_dcd-03_Proche-3.doc - 6:44 [R : Qu’ils continuent de s’occ..] (172:172) (Super)**

Codes: [Prise en charge_Médicale]

No memos

R : Qu’ils continuent de s’occuper de nous seulement leurs réticences qui étaient justifiaient comme leurs mesures de protection à cause de l’épidémie.

**P 2: Bbo_HgrKttmba_Audit_dcd-01_Proche-2.doc - 2:22 [R : Oui il recevait] (93:93) (Super)**

Codes: [Prise en charge_Médicale]

No memos

R : Oui il recevait

**P 6: Bbo_HgrKttmba_Audit_dcd-03_Proche-3.doc - 6:50 [R : la prise en charge est bon..] (197:197) (Super)**

Codes: [Prise en charge_Médicale]

No memos

R : la prise en charge est bonne à l’intérieur

**P17: Ktw_HopMtda_Audit_dcd-05_Proche-3.doc - 17:14 [R : Je ne sais pas, ils venaie..] (61:61) (Super)**

Codes: [Prise en charge_Médicale]

No memos

R : Je ne sais pas, ils venaient avec les médicaments et il y avait des perfusions.

**P17: Ktw_HopMtda_Audit_dcd-05_Proche-3.doc - 17:15 [R. Deux semaines] (65:65) (Super)**

Codes: [Prise en charge_Médicale]

No memos

R. Deux semaines

**P 6: Bbo_HgrKttmba_Audit_dcd-03_Proche-3.doc - 6:43 [R : Oui tous les services étai..] (168:168) (Super)**

Codes: [Prise en charge_Médicale]

No memos

R : Oui tous les services étaient disponibles

**P17: Ktw_HopMtda_Audit_dcd-05_Proche-3.doc - 17:13 [R. On lui donnait des médicame..] (57:57) (Super)**

Codes: [Prise en charge_Médicale]

No memos

R. On lui donnait des médicaments mais je ne sais pas quels médicaments.

**P17: Ktw_HopMtda_Audit_dcd-05_Proche-3.doc - 17:19 [R. A Kitatumba, on semblait no..] (81:81) (Super)**

Codes: [Prise en charge_Médicale]

No memos

R. A Kitatumba, on semblait nous négliger beaucoup.

**P18: Ktw_HopMtda_Audit_dcd-06_Proche-1.doc - 18:24 [R. Oui, quand j’arrivais, je v..] (95:95) (Super)**

Codes: [Prise en charge_Médicale]

No memos

R. Oui, quand j’arrivais, je voyais dans la salle les soignants qui s’occupaient d’elle.

**P 9: Bbo_HopScola_Audit_dcd-07_Proche-1.doc - 9:36 [R : C’est ce qu’on demandait a..] (163:163) (Super)**

Codes: [Prise en charge_Médicale]

No memos

R : C’est ce qu’on demandait aux médecins, si ça ne marchait pas qu’ils fassent le transfert vers un autre hôpital, mais eux disaient que tout irait bien, que la situation était en train de s’améliorer.

**P 1: Bbo_HgrKttmba_Audit_dcd-01_Proche-1.doc - 1:19 [RÉPONDANT : Je dis que la pris..] (60:60) (Super)**

Codes: [Prise en charge_Médicale]

No memos

RÉPONDANT : Je dis que la prise en charge de l’époque est déférente de celle d’aujourd’hui. Par ce que jadis les soignants passaient dans les salles entrain de demander l’évolution du patient et s’il y avait une plainte de la part du malade le soignant s’arrangeait pour soulager le patient en le touchant mais actuellement il vous écoute et vous dit que ça va aller sans même s’approcher du patient.

**P18: Ktw_HopMtda_Audit_dcd-06_Proche-1.doc - 18:25 [R. On n’a pas la connaissance ..] (99:99) (Super)**

Codes: [Prise en charge_Médicale]

No memos

R. On n’a pas la connaissance de ce que font les soignants mais ils étaient toujours à côté d’elle en train de faire leur travail. Je les voyais la soigner seulement.

**P13: Ktw_Hgr_Audit_dcd-08_Proche-2.doc - 13:12 [R : Ce qui m’avait étonné est ..] (50:50) (Super)**

Codes: [Prise en charge_Médicale]

No memos

R : Ce qui m’avait étonné est qu’ils n’avaient pas pu trouver les résultats des examens de l’enfant, alors que la première chose qu’on doit chercher c’est de trouver la pathologie du patient. Et je me demandais s’ils sont vraiment qualifiés dans ce domaine ? C’est ce qui m’étonnait.

**P 5: Bbo_HgrKttmba_Audit_dcd-03_Proche-2.doc - 5:42 [R : Nous avons été surpris de ..] (158:158) (Super)**

Codes: [Prise en charge_Médicale]

No memos

R : Nous avons été surpris de son évolution, après trois de sa prise en charge elle avait rechuté de manière incompréhensive. Au lieu d’être guéris elle avait repris son état jadis, durant une semaine on l’amena dans les soins intensifs (KAZA ROHO). Quelques jours dans ce service on lui retourna étant morte.

**P 5: Bbo_HgrKttmba_Audit_dcd-03_Proche-2.doc - 5:43 [R : Kaza roho c’est un milieu ..] (162:162) (Super)**

Codes: [Prise en charge_Médicale]

No memos

R : Kaza roho c’est un milieu où on amène le malade pour voir comment lui récupérer et on lui avait ajouté l’oxygène.

**P 5: Bbo_HgrKttmba_Audit_dcd-03_Proche-2.doc - 5:38 [R : A Kitatumba sa prise en ch..] (143:143) (Super)**

Codes: [Prise en charge_Médicale]

No memos

R : A Kitatumba sa prise en charge a été bonne, le traitement était bon.

**P 5: Bbo_HgrKttmba_Audit_dcd-03_Proche-2.doc - 5:44 [R : Les prestataires n’ont jam..] (166:166) (Super)**

Codes: [Prise en charge_Médicale]

No memos

R : Les prestataires n’ont jamais été mauvais à l’égard des malades ils s’efforcent de sauver la vie des malades. Par ce que c’est ce qu’ils avaient étudié.

**P 1: Bbo_HgrKttmba_Audit_dcd-01_Proche-1.doc - 1:22 [RÉPONDANT : Oui ils se sont fo..] (70:70) (Super)**

Codes: [Prise en charge_Médicale]

No memos

RÉPONDANT : Oui ils se sont forcés pour son traitement par ce qu’ils lui donnaient les médicaments.

**P 6: Bbo_HgrKttmba_Audit_dcd-03_Proche-3.doc - 6:30 [R : La prise en charge était b..] (119:119) (Super)**

Codes: [Prise en charge_Médicale]

No memos

R : La prise en charge était bonne lorsque vous amenez les résultats du CTE, à l’intérieur il n’y avait pas de problème pour le traitement comme avant l’épidémie.

**P18: Ktw_HopMtda_Audit_dcd-06_Proche-1.doc - 18:28 [R. Cela pouvait l’aider à soig..] (111:111) (Super)**

Codes: [Prise en charge_Médicale]

No memos

R. Cela pouvait l’aider à soigner tous les malaises de la poitrine même si elle est décédée suite à une autre pathologie que celle-là. Il y a eu apparition d’un autre pathologie jusqu’ à ce qu’elle est morte parce qu’elle était guérie et sortie de cette structure pour rentrer dans son domicile. Arrivée à la maison, un autre malaise avait surgi puis amenée à Horizon (structure sanitaire).

**P17: Ktw_HopMtda_Audit_dcd-05_Proche-3.doc - 17:20 [R. Lorsqu’on nous avait demand..] (85:85) (Super)**

Codes: [Prise en charge_Médicale]

No memos

R. Lorsqu’on nous avait demandé d’aller faire la radiographie, et lorsque nous somme revenu il nous avait dit que s’ils lui traitaient elle risque d’être handicap alors on envoie ailleurs ; Je ne sais pas si c’était parce que le cas était difficile à prendre en charge. Il fallait alors nous transférée.

**P 6: Bbo_HgrKttmba_Audit_dcd-03_Proche-3.doc - 6:42 [R : Oui ils s’occupaient d’ell..] (164:164) (Super)**

Codes: [Prise en charge_Médicale]

No memos

R : Oui ils s’occupaient d’elle pour les soins.

**P18: Ktw_HopMtda_Audit_dcd-06_Proche-1.doc - 18:27 [R. Une forme de plâtre] (107:107) (Super)**

Codes: [Prise en charge_Médicale]

No memos

R. Une forme de plâtre

**P 2: Bbo_HgrKttmba_Audit_dcd-01_Proche-2.doc - 2:17 [R : Oui on lui avait traité.] (73:73) (Super)**

Codes: [Prise en charge_Médicale]

No memos

R : Oui on lui avait traité.

**P20: Ktw_HopMtda_Audit_dcd-11_Proche-1.doc - 20:15 [R : Je ne peux pas savoir par ..] (63:63) (Super)**

Codes: [Prise en charge_Médicale]

No memos

R : Je ne peux pas savoir par ce qu’à notre arrivée, quelques minutes elle était reçue aux soins intensifs et personne n’est autorisé d’y entrer. On reste à l’extérieur et s’il y a à donner on sonne une clochette ils viennent récupérer et on rentre encore. Ce sont eux qui s’occupe d’eux pour tous ses besoins.

**P18: Ktw_HopMtda_Audit_dcd-06_Proche-1.doc - 18:26 [R. Les médicaments oui. On ava..] (103:103) (Super)**

Codes: [Prise en charge_Médicale]

No memos

R. Les médicaments oui. On avait mis même une chose dans sa poitrine.

**P 2: Bbo_HgrKttmba_Audit_dcd-01_Proche-2.doc - 2:19 [R : Oui il était bien soigné m..] (81:81) (Super)**

Codes: [Prise en charge_Médicale]

No memos

R : Oui il était bien soigné mais avec retard comme je vous l’ai dit, c’était dans une période de l’épidémie ; à ce moment-là les prestataires ne pouvaient soigner un malade que lorsqu’ils reçoivent le document de résultat du CTE autorisant la prise en charge du patient.

**P 4: Bbo_HgrKttmba_Audit_dcd-03_Proche-1.doc - 4:34 [R : il n’y avait pas d’amélior..] (140:140) (Super)**

Codes: [Prise en charge_Médicale]

No memos

R : il n’y avait pas d’amélioration, peut être que ses jours venaient d’arriver à son terme.

**P 4: Bbo_HgrKttmba_Audit_dcd-03_Proche-1.doc - 4:41 [R : Les Personnels soignants l..] (168:168) (Super)**

Codes: [Prise en charge_Médicale]

No memos

R : Les Personnels soignants lui avaient bien soigner

**P 3: Bbo_HgrKttmba_Audit_dcd-01_Proche-3.doc - 3:23 [R : Oui il prenait bien le méd..] (90:90) (Super)**

Codes: [Prise en charge_Médicale]

No memos

R : Oui il prenait bien le médicament

**P16: Ktw_HopMtda_Audit_dcd-05_Proche-2.doc - 16:19 [R. On la soignait bien] (84:84) (Super)**

Codes: [Prise en charge_Médicale]

No memos

R. On la soignait bien

**P16: Ktw_HopMtda_Audit_dcd-05_Proche-2.doc - 16:20 [R. Le fait qu’elle avait connu..] (88:88) (Super)**

Codes: [Prise en charge_Médicale]

No memos

R. Le fait qu’elle avait connu une fracture à la main, l’avait conduit une énorme douleur mais elle avait quitté à KITATUMBA sa santé commençait à s’améliorer.

**P12: Ktw_Hgr_Audit_dcd-08_Proche-12.doc - 12:14 [R : La prise en charge n’était..] (62:62) (Super)**

Codes: [Prise en charge_Médicale]

No memos

R : La prise en charge n’était pas bon, par ce que sans l’influence du neveu qui avait appelé le médecin et ce dernier s’est donné pour son traitement, leurs façons de prendre en charge n’était pas bonne. Si le neveu n’avait pas appelé le médecin elle n’allait pas être bien reçu.

**P10: Ktw_Hgr_Audit_dcd-02_Proche-1.doc - 10:31 [R : Dans cette semaine-là je n..] (124:124) (Super)**

Codes: [Prise en charge_Médicale]

No memos

R : Dans cette semaine-là je n’avais pas eu de cas pareil que je peux décrire mais avant j’avais d’autres malades dans cet hôpital Katwa, j’avais vu un infirmier qui avais affiché un comportement qui n’était pas bon ; heureusement le malade avait un savoir vivre qui lui avait permis de maitriser la situation. Si c’était une autre personne il aurait déjà une mésentente. Entre les gens il y’a parfois des divergences.

**P 4: Bbo_HgrKttmba_Audit_dcd-03_Proche-1.doc - 4:43 [R : Ce que nous disons bien ch..] (176:176) (Super)**

Codes: [Prise en charge_Médicale]

No memos

R : Ce que nous disons bien chez les prestataires de soins c’est lors ce qu’ils viennent surveiller le malade et lui donnent le traitement.

**P 4: Bbo_HgrKttmba_Audit_dcd-03_Proche-1.doc - 4:42 [R : Tout était bon du coté de ..] (172:172) (Super)**

Codes: [Prise en charge_Médicale]

No memos

R : Tout était bon du coté de prestataire de soins

**P16: Ktw_HopMtda_Audit_dcd-05_Proche-2.doc - 16:28 [R. C’est entre 3 semaines ou u..] (118:118) (Super)**

Codes: [Prise en charge_Médicale]

No memos

R. C’est entre 3 semaines ou un mois

**P 3: Bbo_HgrKttmba_Audit_dcd-01_Proche-3.doc - 3:36 [R : On lui avait soigné et il ..] (142:142) (Super)**

Codes: [Prise en charge_Médicale]

No memos

R : On lui avait soigné et il était déjà affaibli par la maladie à cause de ces trois jours qu’il avait fait sans traitement il était revenu étant au bout de son souffle.

**P 3: Bbo_HgrKttmba_Audit_dcd-01_Proche-3.doc - 3:35 [R : Le rapport devait venir du..] (138:138) (Super)**

Codes: [Prise en charge_Médicale]

No memos

R : Le rapport devait venir du CTE

**P 3: Bbo_HgrKttmba_Audit_dcd-01_Proche-3.doc - 3:37 [R : Je n’ai pas apprécié comme..] (146:146) (Super)**

Codes: [Prise en charge_Médicale]

No memos

R : Je n’ai pas apprécié comme ils le faisaient jadis.

**P 4: Bbo_HgrKttmba_Audit_dcd-03_Proche-1.doc - 4:29 [R : Sa prise en charge était b..] (121:121) (Super)**

Codes: [Prise en charge_Médicale]

No memos

R : Sa prise en charge était bonne les personnels soignants lui avaient bien soigné.

**P 4: Bbo_HgrKttmba_Audit_dcd-03_Proche-1.doc - 4:30 [R : les prestataires lui avaie..] (125:125) (Super)**

Codes: [Prise en charge_Médicale]

No memos

R : les prestataires lui avaient bien soigné.

**P15: Ktw_HopMtda_Audit_dcd-05_Proche-1.doc - 15:33 [R. Je ne restais pas avec la m..] (132:132) (Super)**

Codes: [Prise en charge_Médicale]

No memos

R. Je ne restais pas avec la malade à l’hôpital. Oui, j’arrivais quand on la soignait vraiment bien en lui administrant les médicaments.

**P16: Ktw_HopMtda_Audit_dcd-05_Proche-2.doc - 16:18 [R. A KITATUMBA je ne l’avais p..] (76:76) (Super)**

Codes: [Prise en charge_Médicale]

No memos

R. A KITATUMBA je ne l’avais pas visité à Kitatumba

**P 3: Bbo_HgrKttmba_Audit_dcd-01_Proche-3.doc - 3:28 [R : Non ce n’était pas la rais..] (110:110) (Super)**

Codes: [Prise en charge_Médicale]

No memos

R : Non ce n’était pas la raison, les personnels soignants avaient mal travaillés ; ils n’avaient pas tenu compte de leurs déontologies et quand vous êtes malade pour eux c’est Ebola et ils vous laissent dans leurs bâches vous devez attendre. Sans se soucier de vous.

**P15: Ktw_HopMtda_Audit_dcd-05_Proche-1.doc - 15:36 [R. Ils sont bien parce que nou..] (144:144) (Super)**

Codes: [Prise en charge_Médicale]

No memos

R. Ils sont bien parce que nous y arrivons souvent.

**P 3: Bbo_HgrKttmba_Audit_dcd-01_Proche-3.doc - 3:34 [R : Sa prise en charge n’était..] (134:134) (Super)**

Codes: [Prise en charge_Médicale]

No memos

R : Sa prise en charge n’était pas bonne par ce que si tu pouvais dire au personnels soignants de traiter le malade de cette façon et eux disaient que « nous attendons le rapport » on se demandait si ce rapport viendra quel jour et quelle heure. Et le malade a quelle heure de guérir ou de mourir ?

**P 7: Bbo_HgrKttmba_Audit_dcd-04_Proche-1.doc - 7:26 [R : Nous l’appelons ainsi par ..] (106:106) (Super)**

Codes: [Prise en charge_Médicale]

No memos

R : Nous l’appelons ainsi par ce que c’est entre la mort et la vie

**P 7: Bbo_HgrKttmba_Audit_dcd-04_Proche-1.doc - 7:25 [R : Nous, nous l’appelons KAZA..] (102:102) (Super)**

Codes: [Prise en charge_Médicale]

No memos

R : Nous, nous l’appelons KAZA ROHO (fortifie le Cœur)

**P 7: Bbo_HgrKttmba_Audit_dcd-04_Proche-1.doc - 7:27 [R : Oui elle était prise en ch..] (110:110) (Super)**

Codes: [Prise en charge_Médicale]

No memos

R : Oui elle était prise en charge correctement, la mort est inévitable c’est pourquoi elle morte.

**P 4: Bbo_HgrKttmba_Audit_dcd-03_Proche-1.doc - 4:26 [R : Oui elle était soignée dur..] (109:109) (Super)**

Codes: [Prise en charge_Médicale]

No memos

R : Oui elle était soignée durant cette nuit.

**P10: Ktw_Hgr_Audit_dcd-02_Proche-1.doc - 10:13 [R : C’était moi qui lui gardai..] (53:53) (Super)**

Codes: [Prise en charge_Médicale]

No memos

R : C’était moi qui lui gardait et ma mère était à la cuisine, personnellement je m’adapte facilement et presque pour chaque personne et comme je venais de faire un moment en train de passer la journée à KATWA, je n’avais pas aucun complexe, je me suis familiarisé au près des soignants. Les choses avaient marché en pas de vitesse il n’y avait pas de problème.

**P10: Ktw_Hgr_Audit_dcd-02_Proche-1.doc - 10:12 [R : Nous avons constaté qu’il ..] (49:49) (Super)**

Codes: [Prise en charge_Médicale]

No memos

R : Nous avons constaté qu’il lui traitait bien, s’il y a autre chose donc c’est leurs secrets professionnels que nous ne pouvons pas connaitre.je me permet de dire cela.

**P 7: Bbo_HgrKttmba_Audit_dcd-04_Proche-1.doc - 7:24 [R : C’est ce que j’ai dit que ..] (98:98) (Super)**

Codes: [Prise en charge_Médicale]

No memos

R : C’est ce que j’ai dit que pour les pathologies ordinaires il y avait une bonne prise en charge comment jadis. Par ce que si quelqu’un est déjà aux soins intensifs on lui traite comme il faut.

**P10: Ktw_Hgr_Audit_dcd-02_Proche-1.doc - 10:11 [R : La prise en charge était b..] (45:45) (Super)**

Codes: [Prise en charge_Médicale]

No memos

R : La prise en charge était bonne sauf que le monde médical nous qui sommes profane c’est difficile de vous contrôler parce que nous ne connaissons pas le médicament que vous nous donnez en matière d’efficacité ou la qualité. Seulement qu’ils ont fait ce qu’ils pouvaient en lui ajoutant l’oxygène. Peut-être qu’ils pouvaient constater qu’il n’aura pas un changement chez le malade, ils commencent à lui traiter avec un placer bon pour nous montrer qu’il est en train de recevoir le traitement pour lui maintenir jusqu’au bout de son souffle. Par ce qu’après les examens ils savaient qu’avec le problème de reins il n’allait pas vivre eux ont gardé comme secret professionnel et nous autres on pensait qu’on était en train de lui traiter.

**P 7: Bbo_HgrKttmba_Audit_dcd-04_Proche-1.doc - 7:28 [R : Nous disons toujours merci..] (114:114) (Super)**

Codes: [Prise en charge_Médicale]

No memos

R : Nous disons toujours merci parce qu’ils ne cessent de se donner pour nous sans discrimination de qui que ce soit.

**P 2: Bbo_HgrKttmba_Audit_dcd-01_Proche-2.doc - 2:23 [R : Lors de ma visite je renco..] (97:97) (Super)**

Codes: [Prise en charge_Médicale]

No memos

R : Lors de ma visite je rencontrais sous perfusion mais je ne peux pas savoir quel médicament qu’on lui donnait.

**P14: Ktw_Hgr_Audit_dcd-09_Proche-1.doc - 14:11 [R : Non les soignants ne nous ..] (45:45) (Super)**

Codes: [Prise en charge_Médicale]

No memos

R : Non les soignants ne nous soignaient pas, par ce que l’état de santé du malade qui cri la tête et le ventre et lorsque vous vous rapprochez du soignant pour lui donner un médicament qui va soulager le ventre ainsi que la tête et que le médicament de la gastrite ne soigne pas les douleurs abdominales si vous pouvez ajouter un médicament pour les douleurs abdominales. Ils vous répondaient en disant qu’ils appliquent c’est qui est écrit sur la fiche. Et ce qu’il faut savoir et que pour mieux se faire soigner, il faut avoir une connaissance à l’hôpital il faut demander les autres. Si vous n’avez pas quelqu’un pour qui vous connaissez on ne vous soigne pas bien. L’Hôpital est devenue comme le commerce. Par ce que même pour la deuxième fois que nous sommes revenus ils lui ont traité à cause des médecins familiers que nous sommes venus avec. Ils leurs disaient ce qu’ils vont faire et ils avaient le fait vert d’arriver aux soins intensifs.

**P14: Ktw_Hgr_Audit_dcd-09_Proche-1.doc - 14:12 [R : Ils étaient à trois membre..] (49:49) (Super)**

Codes: [Prise en charge_Médicale]

No memos

R : Ils étaient à trois membres de familles

**P 4: Bbo_HgrKttmba_Audit_dcd-03_Proche-1.doc - 4:49 [R : Peut-être que c’est moi qu..] (200:200) (Super)**

Codes: [Prise en charge_Médicale]

No memos

R : Peut-être que c’est moi qui avait mal vu.

**P 4: Bbo_HgrKttmba_Audit_dcd-03_Proche-1.doc - 4:48 [R : On y écrivait chirurgie fe..] (196:196) (Super)**

Codes: [Prise en charge_Médicale]

No memos

R : On y écrivait chirurgie femme.

**P14: Ktw_Hgr_Audit_dcd-09_Proche-1.doc - 14:13 [R : Oui ce sont eux qui avaien..] (53:53) (Super)**

Codes: [Prise en charge_Médicale]

No memos

R : Oui ce sont eux qui avaient fait le suivi et ces sont eux qui nous avaient déclarés que c’est un cancer et c’est ainsi que nous étions rentrés à la maison.

**P13: Ktw_Hgr_Audit_dcd-08_Proche-2.doc - 13:13 [R : Il y’avait un soutien soci..] (54:54) (Super)**

Codes: [Prise en charge_Psycho-sociale]

No memos

R : Il y’avait un soutien social et psychologique au niveau de la famille. Les membres de la famille étaient mobilisés, elle se souciaient de lui, elle nous donnait tout le nécessaire dont on avait besoin.

**P 2: Bbo_HgrKttmba_Audit_dcd-01_Proche-2.doc - 2:27 [R : Oui les gens étaient nombr..] (113:113) (Super)**

Codes: [Prise en charge_Psycho-sociale]

No memos

R : Oui les gens étaient nombreux qui venaient lui rendre visite et les uns restaient à l’extérieur pour attendre que les autres sortent.

**P 3: Bbo_HgrKttmba_Audit_dcd-01_Proche-3.doc - 3:57 [R : Ils venaient lui réconfort..] (230:230) (Super)**

Codes: [Prise en charge_Psycho-sociale]

No memos

R : Ils venaient lui réconforter malgré qu’il ne parlait plus.

**P 3: Bbo_HgrKttmba_Audit_dcd-01_Proche-3.doc - 3:56 [R : Oui il recevait par ce que..] (226:226) (Super)**

Codes: [Prise en charge_Psycho-sociale]

No memos

R : Oui il recevait par ce que les pasteurs et le prêtre arrivaient pour prier.

**P13: Ktw_Hgr_Audit_dcd-08_Proche-2.doc - 13:14 [R : Spirituellement oui, il av..] (58:58) (Super)**

Codes: [Prise en charge_Psycho-sociale]

No memos

R : Spirituellement oui, il avait les représentants de l’église et les autres chrétiens venaient prier pour lui, mais aussi ils faisaient la réadaptation psychologique parce que nous africains, nous croyons toujours qu’à côté de la maladie il y’a toujours la sorcellerie. C’est pourquoi ils priaient pour lui, et lui rendaient toujours visite.

**P 3: Bbo_HgrKttmba_Audit_dcd-01_Proche-3.doc - 3:43 [R : Oui ils arrivaient pour le..] (170:170) (Super)**

Codes: [Prise en charge_Psycho-sociale]

No memos

R : Oui ils arrivaient pour le réconfort.

**P18: Ktw_HopMtda_Audit_dcd-06_Proche-1.doc - 18:33 [R. Non.] (132:132) (Super)**

Codes: [Prise en charge_Psycho-sociale]

No memos

R. Non.

**P 4: Bbo_HgrKttmba_Audit_dcd-03_Proche-1.doc - 4:46 [R : la famille a souffert pend..] (188:188) (Super)**

Codes: [Prise en charge_Psycho-sociale]

No memos

R : la famille a souffert pendant cette maladie, on était dérangé de sa maladie.

**P 4: Bbo_HgrKttmba_Audit_dcd-03_Proche-1.doc - 4:44 [R : La lui visitait chaque foi..] (180:180) (Super)**

Codes: [Prise en charge_Psycho-sociale]

No memos

R : La lui visitait chaque fois.

**P18: Ktw_HopMtda_Audit_dcd-06_Proche-1.doc - 18:32 [R. Nous l’avions vraiment assi..] (127:127) (Super)**

Codes: [Prise en charge_Psycho-sociale]

No memos

R. Nous l’avions vraiment assisté de la manière que nous pouvions, malgré que personne ne peut arrêter celui qui va mourir.

**P18: Ktw_HopMtda_Audit_dcd-06_Proche-1.doc - 18:31 [R. Sur le plan social, la défu..] (123:123) (Super)**

Codes: [Prise en charge_Psycho-sociale]

No memos

R. Sur le plan social, la défunte était une personne de bonne renommée parce qu’elle avait seulement 5 enfants mais on pouvait rencontrer chez elle plus de 13 personnes dans sa parcelle (pour dire qu’elle était une personne accueillante de la part de l’entourage).

**P 3: Bbo_HgrKttmba_Audit_dcd-01_Proche-3.doc - 3:42 [R : Oui la famille asseyait de..] (166:166) (Super)**

Codes: [Prise en charge_Psycho-sociale]

No memos

R : Oui la famille asseyait de lui encadrer pendant sa maladie.

**P 1: Bbo_HgrKttmba_Audit_dcd-01_Proche-1.doc - 1:21 [Répondant : Oui les gens de la..] (67:67) (Super)**

Codes: [Prise en charge_Psycho-sociale]

No memos

Répondant : Oui les gens de la communauté priaient pour lui lors de la visite. Et avant sa mort nous avions appelé le prêtre pour certains sacrements.

**P 1: Bbo_HgrKttmba_Audit_dcd-01_Proche-1.doc - 1:20 [RÉPONDANT : Non non cela n’exi..] (64:64) (Super)**

Codes: [Prise en charge_Psycho-sociale]

No memos

RÉPONDANT : Non non cela n’existait pas de la part des soignants.

**P 1: Bbo_HgrKttmba_Audit_dcd-01_Proche-1.doc - 1:25 [RÉPONDANT : Oui la famille l’a..] (80:80) (Super)**

Codes: [Prise en charge_Psycho-sociale]

No memos

RÉPONDANT : Oui la famille l’assistait par ce que pendant la journée on y était à trois.

**P 4: Bbo_HgrKttmba_Audit_dcd-03_Proche-1.doc - 4:33 [R : Oui elle était contente et..] (136:136) (Super)**

Codes: [Prise en charge_Psycho-sociale]

No memos

R : Oui elle était contente et parlait avec nous.

**P 4: Bbo_HgrKttmba_Audit_dcd-03_Proche-1.doc - 4:32 [R : Oui on lui soutenait penda..] (132:132) (Super)**

Codes: [Prise en charge_Psycho-sociale]

No memos

R : Oui on lui soutenait pendant sa maladie en lui amenant à manger ; de l’eau pour se laver et prier pour elle.

**P15: Ktw_HopMtda_Audit_dcd-05_Proche-1.doc - 15:40 [R. Non, mais les autres et les..] (159:159) (Super)**

Codes: [Prise en charge_Psycho-sociale]

No memos

R. Non, mais les autres et les responsables de l’église étaient arrivés.

**P12: Ktw_Hgr_Audit_dcd-08_Proche-12.doc - 12:16 [R : Elle était décédée le same..] (70:70) (Super)**

Codes: [Prise en charge_Psycho-sociale]

No memos

R : Elle était décédée le samedi et on avait arrivé le lundi.

**P15: Ktw_HopMtda_Audit_dcd-05_Proche-1.doc - 15:39 [R. Oui.] (156:156) (Super)**

Codes: [Prise en charge_Psycho-sociale]

No memos

R. Oui.

**P 8: Bbo_HgrKttmba_Audit_dcd-04_Proche-2.doc - 8:15 [R : Ils lui rendaient visite à..] (62:62) (Super)**

Codes: [Prise en charge_Psycho-sociale]

No memos

R : Ils lui rendaient visite à tout moment et lui apportait à manger

**P14: Ktw_Hgr_Audit_dcd-09_Proche-1.doc - 14:19 [R : Du côté spirituel il recev..] (77:77) (Super)**

Codes: [Prise en charge_Psycho-sociale]

No memos

R : Du côté spirituel il recevait de visite de différentes églises comme lui était protestant dans l’église de réveil EERA comme sa belle-famille est catholique elle amenait les prêtres pour venir prier pour lui à Katwa. Spirituellement il était bien préparé, seulement il y avait une petite incompréhension entre les deux familles. Lorsqu’il y a une maladie chronique dans la famille il y a toujours des mauvaises pensées. La famille du malade venait récupérer leur enfant en lui séparant de sa femme ce qui n’était pas acceptable par l’autre famille et même son épouse, car lorsqu’on l’amenait sa femme y aller pour lui récupérer encore.

**P17: Ktw_HopMtda_Audit_dcd-05_Proche-3.doc - 17:16 [R. Quand j’arrive à la maison ..] (69:69) (Super)**

Codes: [Prise en charge_Psycho-sociale]

No memos

R. Quand j’arrive à la maison après avoir lui laver je prépare la nourriture pour lui apporter, je passe la nuit jusqu’au matin peut-être et ses enfants nous aidaient.

**P17: Ktw_HopMtda_Audit_dcd-05_Proche-3.doc - 17:17 [R : Je n’avais pas vu un autre..] (73:73) (Super)**

Codes: [Prise en charge_Psycho-sociale]

No memos

R : Je n’avais pas vu un autre familier peut être qu’ils arrivaient à mon absence. Parfois j’y rencontrais le pain sans savoir qui l’apporté.

**P10: Ktw_Hgr_Audit_dcd-02_Proche-1.doc - 10:17 [R : Il faut être franc que les..] (69:69) (Super)**

Codes: [Prise en charge_Psycho-sociale]

No memos

R : Il faut être franc que les familles sont en difficultés et il peut y avoir qu’il y a quelqu’un que vous partagez les mêmes avis mais qui n’a pas le moyen. C’est un seul parmi les oncles paternels qui nous collaborons, les autres ne sont plus en vie et les autres ne vivent pas dans le lieu. Le Cadet des oncles paternel qui était venu de voyage et arriva au dernier moment où nous étions dans les soins intensifs et qui était obligé de passé la nuit et le matin à 11hoo le malade. Mais les familiers nous visitaient de temps en temps c’était bon.

**P12: Ktw_Hgr_Audit_dcd-08_Proche-12.doc - 12:18 [R : Non aucun soutien spiritue..] (78:79) (Super)**

Codes: [Prise en charge_Psycho-sociale]

No memos

R : Non aucun soutien spirituel avait eu mais avant d’arriver à l’hôpital les pasteurs arrivaient ici à la maison pour prier.

00 :17 :31

**P10: Ktw_Hgr_Audit_dcd-02_Proche-1.doc - 10:18 [R : Oui ils venaient nous rend..] (73:73) (Super)**

Codes: [Prise en charge_Psycho-sociale]

No memos

R : Oui ils venaient nous rendre visite.

**P15: Ktw_HopMtda_Audit_dcd-05_Proche-1.doc - 15:37 [R. Beaucoup plus] (148:148) (Super)**

Codes: [Prise en charge_Psycho-sociale]

No memos

R. Beaucoup plus

**P14: Ktw_Hgr_Audit_dcd-09_Proche-1.doc - 14:18 [R : Non] (73:73) (Super)**

Codes: [Prise en charge_Psycho-sociale]

No memos

R : Non

**P15: Ktw_HopMtda_Audit_dcd-05_Proche-1.doc - 15:38 [R. Oui bien sûr, on l’aidait b..] (152:152) (Super)**

Codes: [Prise en charge_Psycho-sociale]

No memos

R. Oui bien sûr, on l’aidait beaucoup.

**P16: Ktw_HopMtda_Audit_dcd-05_Proche-2.doc - 16:24 [R. Oui] (103:103) (Super)**

Codes: [Prise en charge_Psycho-sociale]

No memos

R. Oui

**P16: Ktw_HopMtda_Audit_dcd-05_Proche-2.doc - 16:25 [R. Oui, même les infirmiers la..] (106:106) (Super)**

Codes: [Prise en charge_Psycho-sociale]

No memos

R. Oui, même les infirmiers la prenaient bien en charge. La maladie qui va emporter quelqu’un ne compte pas sur le traitement (Tout était bien fait mais hélas !).

**P 7: Bbo_HgrKttmba_Audit_dcd-04_Proche-1.doc - 7:29 [R : Je dirais que psychologiqu..] (118:118) (Super)**

Codes: [Prise en charge_Psycho-sociale]

No memos

R : Je dirais que psychologiquement elle était assistée par ce qu’il y avait de gens qui lui visitaient pour lui réconforter en apportant la bouillie et autre mais spirituellement je ne sais pas.

**P12: Ktw_Hgr_Audit_dcd-08_Proche-12.doc - 12:15 [R : Son soutien psychologique ..] (66:66) (Super)**

Codes: [Prise en charge_Psycho-sociale]

No memos

R : Son soutien psychologique n’était pas à cause de la durée, on était arrivé le lundi et mardi la nuit on lui amena dans les soins intensifs et là elle ne parlait plus.

**P 7: Bbo_HgrKttmba_Audit_dcd-04_Proche-1.doc - 7:30 [R : Oui on priait souvent pour..] (122:122) (Super)**

Codes: [Prise en charge_Psycho-sociale]

No memos

R : Oui on priait souvent pour elle.

**P14: Ktw_Hgr_Audit_dcd-09_Proche-1.doc - 14:17 [R : C’était nous et les médeci..] (69:69) (Super)**

Codes: [Prise en charge_Psycho-sociale]

No memos

R : C’était nous et les médecins membres de la famille

**P14: Ktw_Hgr_Audit_dcd-09_Proche-1.doc - 14:16 [R : C’était nous de la famille..] (65:65) (Super)**

Codes: [Prise en charge_Psycho-sociale]

No memos

R : C’était nous de la famille.

**P17: Ktw_HopMtda_Audit_dcd-05_Proche-3.doc - 17:18 [R. Certaines mamans croyantes ..] (77:77) (Super)**

Codes: [Prise en charge_Psycho-sociale]

No memos

R. Certaines mamans croyantes arrivaient pour prier et nous laisser quelque fois une somme d’argent pour acheter du sucre.

**P11: Ktw_Hgr_Audit_dcd-08_Proche-1.doc - 11:46 [R/Ils ont peur d’être amené da..] (188:188) (Super)**

Codes: [Prise en charge_Psycho-sociale]

No memos

R/Ils ont peur d’être amené dans les maisons en bâches (tantes).

**P11: Ktw_Hgr_Audit_dcd-08_Proche-1.doc - 11:47 [R/ Lorsque la température est ..] (192:192) (Super)**

Codes: [Prise en charge_Psycho-sociale]

No memos

R/ Lorsque la température est élevée.

**P 5: Bbo_HgrKttmba_Audit_dcd-03_Proche-2.doc - 5:54 [R : Lorsqu’un malade arrive à ..] (206:206) (Super)**

Codes: [Prise en charge_Psycho-sociale]

No memos

R : Lorsqu’un malade arrive à l’hôpital il n’est plus à la charge de la famille automatiquement, la charge revient aux prestataires des soins.

**P 5: Bbo_HgrKttmba_Audit_dcd-03_Proche-2.doc - 5:55 [R : Il y avait d’autres qui ét..] (210:210) (Super)**

Codes: [Prise en charge_Psycho-sociale]

No memos

R : Il y avait d’autres qui étaient, mais moi je n’y étais pas à cause du travail de la maison.

**P11: Ktw_Hgr_Audit_dcd-08_Proche-1.doc - 11:45 [R/ Oui certain mais nombreux r..] (184:184) (Super)**

Codes: [Prise en charge_Psycho-sociale]

No memos

R/ Oui certain mais nombreux refusaient d’arriver par ce qu’ils ont peur d’entrer dans les structures sanitaires où il y a la prise en charge de cas d’Ebola.

**P 9: Bbo_HopScola_Audit_dcd-07_Proche-1.doc - 9:31 [R : le pasteur et les autres y..] (140:140) (Super)**

Codes: [Prise en charge_Psycho-sociale]

No memos

R : le pasteur et les autres y arrivaient chaque jour pour la prière .

**P14: Ktw_Hgr_Audit_dcd-09_Proche-1.doc - 14:20 [R : L’enfant que vous avez eng..] (81:81) (Super)**

Codes: [Prise en charge_Psycho-sociale]

No memos

R : L’enfant que vous avez engendré malgré sa vie conjugale, on a toujours de soucis pour son bien-être. Elle l’amenait pour voir ce qui ne va pas et chercher comment trouver d’autres moyens de soins appropriés.

**P 9: Bbo_HopScola_Audit_dcd-07_Proche-1.doc - 9:29 [R : Oui ils arrivaient.] (132:132) (Super)**

Codes: [Prise en charge_Psycho-sociale]

No memos

R : Oui ils arrivaient.

**P11: Ktw_Hgr_Audit_dcd-08_Proche-1.doc - 11:48 [R : les gens refusaient d’alle..] (196:196) (Super)**

Codes: [Prise en charge_Psycho-sociale]

No memos

R : les gens refusaient d’aller là où il y a le thermo flash

**P11: Ktw_Hgr_Audit_dcd-08_Proche-1.doc - 11:49 [R/Nous sommes de l’église cath..] (200:200) (Super)**

Codes: [Prise en charge_Psycho-sociale]

No memos

R/Nous sommes de l’église catholique chez nous les enfants reçoivent le baptême aux bas âges, mais n’avait pas encore eu le baptême. Il disait souvent qu’il sera baptisé le jour de son mariage. Le malade lui-même étant grand âgé de 17ans m’avait dit de lui appeler le prêtre pour qu’il vienne me baptiser ici aux soins intensifs, parce que vois comme si je ne veux pas guérir car les douleurs sont énormes, avant de l’appelé j’avais d’abord demandé la permission aux responsables de la structure en leurs disant que dans notre église on baptise quelqu’un avant de mourir, ils avaient accepté en disant qu’il ne fallait pas que le prêtre y traine et je leurs avais répondu qu’il n’allait pas trainé comme c’est un service qui a des malades qui sont graves. Le prêtre était arrivé pour lui baptiser dans les soins intensifs, il lui avait demander s’il voulait quel nom de baptême, il avait répondu qu’il voulait qu’on l’appelle Joseph et le prêtre a dit que depuis aujourd’hui tu t’appelles Joseph et après une semaine il est mort ; 00 :17 :57

**P 5: Bbo_HgrKttmba_Audit_dcd-03_Proche-2.doc - 5:56 [R : Oui nous avons échoué de l..] (214:214) (Super)**

Codes: [Prise en charge_Psycho-sociale]

No memos

R : Oui nous avons échoué de lui donner le sang et cela était causée par la peur et tout le monde était dans la même situation, on nous interdisait de ne pas toucher même ses habits par ce que j’avais demandé à un soignant de lui couvrir il avait refusé et lui-même était venu lui couvrir.

**P 6: Bbo_HgrKttmba_Audit_dcd-03_Proche-3.doc - 6:31 [R : Oui elle avait eu un souti..] (123:123) (Super)**

Codes: [Prise en charge_Psycho-sociale]

No memos

R : Oui elle avait eu un soutien social parce que ses frères ; sœurs ; ses belles sœurs étaient souvent à l’hôpital pour la soutenir et la réconforter.

**P11: Ktw_Hgr_Audit_dcd-08_Proche-1.doc - 11:44 [R/ Oui le matin pour voir comm..] (180:180) (Super)**

Codes: [Prise en charge_Psycho-sociale]

No memos

R/ Oui le matin pour voir comment s’est réveillé le malade, mais d’autres aussi refusaient de venir disant qu’à Katwa il contrôle Ebola qu’ils ont peur du lavage des mains et du thermo flash pour la température.

**P 6: Bbo_HgrKttmba_Audit_dcd-03_Proche-3.doc - 6:39 [R : Le soutien financier n’éta..] (151:151) (Super)**

Codes: [Prise en charge_Psycho-sociale]

No memos

R : Le soutien financier n’était pas là seulement son grand frère seulement qui lui assisté de temps en temps.

**P 6: Bbo_HgrKttmba_Audit_dcd-03_Proche-3.doc - 6:32 [R : Elle avait bénéficié de so..] (127:127) (Super)**

Codes: [Prise en charge_Psycho-sociale]

No memos

R : Elle avait bénéficié de soutien spirituel parce que dans la structure il y a un service de l’aumônerie de temps en temps il passait pour prier.

**P14: Ktw_Hgr_Audit_dcd-09_Proche-1.doc - 14:21 [R : Un humain est toujours hum..] (85:85) (Super)**

Codes: [Prise en charge_Psycho-sociale]

No memos

R : Un humain est toujours humain le fait de se regardait physiquement on ne peut pas connaitre ce qui est dans son cœur. Nous regrettons le fait de n’est pas nous donner les résultats à la première hospitalisation on pouvait prendre d’autre mesures. Et il n’avait pas accepté pour qu’on lui amène à Katwa pour les soins du deuxième tour. Pour qu’il accepte nous lui avons dit que nous y avons envoyé les soignants qui pourront lui soigner.

**P 9: Bbo_HopScola_Audit_dcd-07_Proche-1.doc - 9:27 [R : Il vivait en bon terme ave..] (123:123) (Super)**

Codes: [Prise en charge_Psycho-sociale]

No memos

R : Il vivait en bon terme avec les voisins, et on venait lui visiter à l’hôpital en dialoguant avec lui sans problème.

**P 1: Bbo_HgrKttmba_Audit_dcd-01_Proche-1.doc - 1:37 [RÉPONDANT : Aucune chose par c..] (129:129) (Super)**

Codes: [Repproche_Malade-Famille]

No memos

RÉPONDANT : Aucune chose par ce qu’il prenait les médicaments sans difficulté. A la famille il n’y a pas à reprocher par ce que Dieu a fini la création en créant l’homme il y a sa date de naissance ainsi que sa date de mourir et si cette dernière est arrivée, même le miracle ne peut pas changer le plan divin. Nous on avait compris que c’était son jour qui était arriver

**P19: Ktw_HopMtda_Audit_dcd-10_Proche-1.doc - 19:10 [R : Tout ce que je faisais, c’..] (45:45) (Super)**

Codes: [Rôle_Repondant]

No memos

R : Tout ce que je faisais, c’était attendre l’ordre des soignants pour exécuter leurs recommandations

**P19: Ktw_HopMtda_Audit_dcd-10_Proche-1.doc - 19:9 [R : oui, c’était moi.] (41:41) (Super)**

Codes: [Rôle_Repondant]

No memos

R : oui, c’était moi.

**P13: Ktw_Hgr_Audit_dcd-08_Proche-2.doc - 13:10 [R : Lui aider lorsqu’il voulai..] (41:41) (Super)**

Codes: [Rôle_Repondant]

No memos

R : Lui aider lorsqu’il voulait faire ses besoins intimes, changer sa position sur le lit, lui nourrir veiller sur lui comme il criait des douleurs intenses et son bain. Car il a fini par décédé après avoir fait deux mois sans sommeiller.

**P 9: Bbo_HopScola_Audit_dcd-07_Proche-1.doc - 9:44 [R : J’y allais à souvent le ma..] (203:203) (Super)**

Codes: [Rôle_Repondant]

No memos

R : J’y allais à souvent le matin et le soir et presque tout le monde au quartier c’est notre hôpital préfère.

**P20: Ktw_HopMtda_Audit_dcd-11_Proche-1.doc - 20:6 [R : Lorsqu’elle était tombé ma..] (31:31) (Super)**

Codes: [Rôle_Repondant]

No memos

R : Lorsqu’elle était tombé malade je l’avais amené à l’hôpital et la gardais.

**P 2: Bbo_HgrKttmba_Audit_dcd-01_Proche-2.doc - 2:3 [R : Je venais lui saluer seule..] (13:13) (Super)**

Codes: [Rôle_Repondant]

No memos

R : Je venais lui saluer seulement.

**P11: Ktw_Hgr_Audit_dcd-08_Proche-1.doc - 11:17 [R : Oui, je pensais que ça va ..] (69:69) (Super)**

Codes: [Rôle_Repondant]

No memos

R : Oui, je pensais que ça va lui aider mais ça n’a pas tenu.

**P20: Ktw_HopMtda_Audit_dcd-11_Proche-1.doc - 20:5 [R : je faisais le commerce] (27:27) (Super)**

Codes: [Rôle_Repondant]

No memos

R : je faisais le commerce

**P 1: Bbo_HgrKttmba_Audit_dcd-01_Proche-1.doc - 1:2 [Répondant : A l’hôpital, Nous ..] (10:10) (Super)**

Codes: [Rôle_Repondant]

No memos

Répondant : A l’hôpital, Nous avions fait la confusion de l’hôpital du malade. Car il longtemps souffert et généralement nous l’amenions à l’hôpital Horizon pour les soins. Nous avions vu que cette structure était trop loin. C’est comme ça que nous l’avions amené ici à Kitatumba. Et ce sont les soignants d’Horizon qui le connaissaient mieux ses pathologies. Et quand nous l’avons amené ici, il était un nouveau patient dans la structure. Et en plus nous l’avions amené dans cet hôpital pendant le mauvais moment de la période de l’épidémie à Ebola. La manière de soigner les malades était devenue difficile. Le malade pouvait arriver à l’hôpital dans un état critique, mais les soignants ne pouvaient pas se préoccuper de lui. Même l’accueil, ils n’accueillaient pas. Ils peuvent t’orienter où amener le malade mais ils ne pouvaient pas s’occuper suffisamment de lui. Et même si beaucoup de gens sont morts, c’était à cause de mêmes problèmes. Donc tu vas voir quelqu’un est malade, mais ils vont t’abandonner en disant que nous attendons les résultats, nous attendons la hiérarchie ou je ne sais quoi. Tu peux patienter jusqu’à trois heures du temps sans qu’on ne te soigne pas, ni te toucher.

**P 1: Bbo_HgrKttmba_Audit_dcd-01_Proche-1.doc - 1:1 [Répondant : Le malade, c’est m..] (6:6) (Super)**

Codes: [Rôle_Repondant]

No memos

Répondant : Le malade, c’est moi qui le gardais.

**P 2: Bbo_HgrKttmba_Audit_dcd-01_Proche-2.doc - 2:2 [R : Je lui rendais visite à l’..] (9:9) (Super)**

Codes: [Rôle_Repondant]

No memos

R : Je lui rendais visite à l’hôpital.

**P12: Ktw_Hgr_Audit_dcd-08_Proche-12.doc - 12:10 [R : Par ce que je vivais avec ..] (42:42) (Super)**

Codes: [Rôle_Repondant]

No memos

R : Par ce que je vivais avec la défunte ici à la maison.

**P11: Ktw_Hgr_Audit_dcd-08_Proche-1.doc - 11:16 [R/ Nous étions en train de fai..] (65:65) (Super)**

Codes: [Rôle_Repondant]

No memos

R/ Nous étions en train de faire la garde son père et moi, nous deux.

**P 3: Bbo_HgrKttmba_Audit_dcd-01_Proche-3.doc - 3:11 [R : Je ne faisais rein par ce ..] (42:42) (Super)**

Codes: [Rôle_Repondant]

No memos

R : Je ne faisais rein par ce qu’à cette période on interdisait de toucher le malade et ses objets il faut lui voir à distance. Il était à l’intérieur et nous on était à l’extérieur.

**P 3: Bbo_HgrKttmba_Audit_dcd-01_Proche-3.doc - 3:4 [R : Je lui visitais chaque foi..] (14:14) (Super)**

Codes: [Rôle_Repondant]

No memos

R : Je lui visitais chaque fois à l’hôpital et il souffrait de la prostate

**P 3: Bbo_HgrKttmba_Audit_dcd-01_Proche-3.doc - 3:21 [R : Oui même à la maison comme..] (82:82) (Super)**

Codes: [Rôle_Repondant]

No memos

R : Oui même à la maison comme mon grand père

**P 3: Bbo_HgrKttmba_Audit_dcd-01_Proche-3.doc - 3:12 [R : Non si tu te rapproches de..] (46:46) (Super)**

Codes: [Rôle_Repondant]

No memos

R : Non si tu te rapproches de lui on te fait payer une amande.

**P15: Ktw_HopMtda_Audit_dcd-05_Proche-1.doc - 15:17 [R. Non] (70:70) (Super)**

Codes: [Rôle_Repondant]

No memos

R. Non

**P15: Ktw_HopMtda_Audit_dcd-05_Proche-1.doc - 15:16 [R. Nous partions lui rendre vi..] (65:65) (Super)**

Codes: [Rôle_Repondant]

No memos

R. Nous partions lui rendre visite à l’hôpital et rentre à la maison. Quand on trouvait quelque chose à manger qu’on pouvait lui amener

**P11: Ktw_Hgr_Audit_dcd-08_Proche-1.doc - 11:15 [R/ je préparais pour lui l’euc..] (61:61) (Super)**

Codes: [Rôle_Repondant]

No memos

R/ je préparais pour lui l’eucalyptus blanc en dehors des médicaments qu’il prenait car je sais que l’eucalyptus blanc soigne aussi la toux.

**P15: Ktw_HopMtda_Audit_dcd-05_Proche-1.doc - 15:18 [R. Oui] (73:73) (Super)**

Codes: [Rôle_Repondant]

No memos

R. Oui

**P 6: Bbo_HgrKttmba_Audit_dcd-03_Proche-3.doc - 6:14 [R : Notre role était d’aller l..] (53:53) (Super)**

Codes: [Rôle_Repondant]

No memos

R : Notre role était d’aller lui visiter en lui apportant la nourriture sauf au CTE que je n’étais pas arrivée comme j’étais en train d’enseigner.

**P 6: Bbo_HgrKttmba_Audit_dcd-03_Proche-3.doc - 6:15 [R : Apres le CTE on ne dialogu..] (57:57) (Super)**

Codes: [Rôle_Repondant]

No memos

R : Apres le CTE on ne dialoguait plus par ce qu’elle devenait gravement malade son état était critique.

**P 7: Bbo_HgrKttmba_Audit_dcd-04_Proche-1.doc - 7:14 [R : Au soins intensifs on auto..] (58:58) (Super)**

Codes: [Rôle_Repondant]

No memos

R : Au soins intensifs on autorise pas tout le monde à visiter le malade, seulement le garde malade qui peut rester à côté d’elle.

**P 5: Bbo_HgrKttmba_Audit_dcd-03_Proche-2.doc - 5:23 [R : Non, je n’ai pas eu du tem..] (91:91) (Super)**

Codes: [Rôle_Repondant]

No memos

R : Non, je n’ai pas eu du temps parce qu’ici à la maison je suis surchargé et seule je prépare pour mon mari qui passe son temps ai travail.

**P16: Ktw_HopMtda_Audit_dcd-05_Proche-2.doc - 16:9 [R. Je partais lui rendre visit..] (37:37) (Super)**

Codes: [Rôle_Repondant]

No memos

R. Je partais lui rendre visite à l’hôpital pour une salutation

**P16: Ktw_HopMtda_Audit_dcd-05_Proche-2.doc - 16:10 [R : Elle criait toujours les d..] (41:41) (Super)**

Codes: [Rôle_Repondant]

No memos

R : Elle criait toujours les douleurs et moi-même je me sentais mal à cause d’elle.

**P 8: Bbo_HgrKttmba_Audit_dcd-04_Proche-2.doc - 8:5 [R : Je surveillais seulement l..] (22:22) (Super)**

Codes: [Rôle_Repondant]

No memos

R : Je surveillais seulement la perfusion.

**P13: Ktw_Hgr_Audit_dcd-08_Proche-2.doc - 13:9 [R : Quand j’avais vu les signe..] (37:37) (Super)**

Codes: [Rôle_Repondant]

No memos

R : Quand j’avais vu les signes que présentait l’enfant de la tuberculose, je me suis rappelé directement que ça peut être la tuberculose et je me suis fait dépisté, j’avais fait les examens à Katwa dans la même structure, lors de résultat ils m’avaient dit que je dois refaire les examens demain et après j’avais fait l’examen de crachant mais lors de résultat on me disait d’attendre. Et quand leurs avais dit que je garde l’enfant qui est aux soins intensifs ils me répondaient que je repasserai après. Ils m’avaient donné les résultats sauf le résultat du crachant jusqu’à présent ils n’ont pas donné, alors que moi et madame avons commencé à tousser ce qui était à la base de mon dépistage pour prévenir et c’est après moi que madame devait être dépistée aussi. Nous y avons quitté sans que j’obtienne les résultats et j’ai même le numéro de la fiche, mais je ne jamais y retourné car ça fait longtemps et ils avaient pris le crachant deux fois sans donner le résultat. Et l’enfant décéda dans cette circonstance sans que je reçoive le résultat.

**P 7: Bbo_HgrKttmba_Audit_dcd-04_Proche-1.doc - 7:13 [R : Je lui rendais visite seul..] (54:54) (Super)**

Codes: [Rôle_Repondant]

No memos

R : Je lui rendais visite seulement.

**P17: Ktw_HopMtda_Audit_dcd-05_Proche-3.doc - 17:7 [Q. Je la gardé à l’hôpital, je..] (29:29) (Super)**

Codes: [Rôle_Repondant]

No memos

**Q. Je la gardé à l’hôpital, je changeais sa position au lit car elle n’était plus à mesure de changer de position, je la faisais son bain, et aussi je lessivais pour elle.**

**P17: Ktw_HopMtda_Audit_dcd-05_Proche-3.doc - 17:10 [R : Elle était internée le soi..] (45:45) (Super)**

Codes: [Rôle_Repondant]

No memos

R : Elle était internée le soir et le matin on était venu m’appeler.

**P 6: Bbo_HgrKttmba_Audit_dcd-03_Proche-3.doc - 6:22 [R : J’y étais arrivée une fois..] (86:86) (Super)**

Codes: [Rôle_Repondant]

No memos

R : J’y étais arrivée une fois par ce que je n’avais pas du temps.

**P 5: Bbo_HgrKttmba_Audit_dcd-03_Proche-2.doc - 5:22 [R : Je l’avais visité et j’y a..] (87:87) (Super)**

Codes: [Rôle_Repondant]

No memos

R : Je l’avais visité et j’y avais passé à peu près une heure.

**P 5: Bbo_HgrKttmba_Audit_dcd-03_Proche-2.doc - 5:21 [R : Je lui gardais et surtout ..] (83:83) (Super)**

Codes: [Rôle_Repondant]

No memos

R : Je lui gardais et surtout pendant sa crise épileptique je l’arrangeais.

**P 9: Bbo_HopScola_Audit_dcd-07_Proche-1.doc - 9:14 [R : Je lui rendais visite à l’..] (64:64) (Super)**

Codes: [Rôle_Repondant]

No memos

R : Je lui rendais visite à l’hôpital, je lui donnais espoir et si j’ai trouvé à manger je lui amenais.

**P 4: Bbo_HgrKttmba_Audit_dcd-03_Proche-1.doc - 4:10 [R : Je lui visitais chaque foi..] (45:45) (Super)**

Codes: [Rôle_Repondant]

No memos

R : Je lui visitais chaque fois en apportant la nourriture et de l’eau.

**P18: Ktw_HopMtda_Audit_dcd-06_Proche-1.doc - 18:14 [R. Oui] (53:53) (Super)**

Codes: [Rôle_Repondant]

No memos

R. Oui

**P18: Ktw_HopMtda_Audit_dcd-06_Proche-1.doc - 18:13 [R. Non, je ne la gardais pas.] (49:49) (Super)**

Codes: [Rôle_Repondant]

No memos

R. Non, je ne la gardais pas.

**P18: Ktw_HopMtda_Audit_dcd-06_Proche-1.doc - 18:12 [R. Quand elle était internée à..] (45:45) (Super)**

Codes: [Rôle_Repondant]

No memos

R. Quand elle était internée à Kalungamakuha, je lui rendais visite : on lui apportait ce qu’on pouvait détenir comme la bouillie, autre et laissiver pour elle en tant que voisine.

**P 5: Bbo_HgrKttmba_Audit_dcd-03_Proche-2.doc - 5:24 [R : Oui il y avait une autre p..] (95:95) (Super)**

Codes: [Rôle_Repondant]

No memos

R : Oui il y avait une autre personne et elle qui était venue m’informé.

**P 4: Bbo_HgrKttmba_Audit_dcd-03_Proche-1.doc - 4:11 [R : Les autres le faisaient.] (49:49) (Super)**

Codes: [Rôle_Repondant]

No memos

R : Les autres le faisaient.

**P 1: Bbo_HgrKttmba_Audit_dcd-01_Proche-1.doc - 1:36 [RÉPONDANT : Qu’ils changent le..] (125:125) (Super)**

Codes: [Souhait_Changement]

No memos

RÉPONDANT : Qu’ils changent leurs comportements et prendre leurs anciennes habitudes qui consistait à accueillir les malades sans craintes en faisant tous les examens de routine et après ils peuvent vous soigner.

**P 1: Bbo_HgrKttmba_Audit_dcd-01_Proche-1.doc - 1:15 [RÉPONDANT : Rien] (47:47) (Super)**

Codes: [Traitement avant Hospitalisation]

No memos

RÉPONDANT : Rien

**P 1: Bbo_HgrKttmba_Audit_dcd-01_Proche-1.doc - 1:14 [RÉPONDANT : On lui soignait se..] (44:44) (Super)**

Codes: [Traitement avant Hospitalisation]

No memos

RÉPONDANT : On lui soignait seulement je ne savais pas quel médicament qu’on lui administrait.

**P11: Ktw_Hgr_Audit_dcd-08_Proche-1.doc - 11:23 [R : Oui et je mélangeais avec ..] (93:93) (Super)**

Codes: [Traitement avant Hospitalisation]

No memos

R : Oui et je mélangeais avec du miel

**P11: Ktw_Hgr_Audit_dcd-08_Proche-1.doc - 11:22 [R : C’est toujours Makulumbe] (89:89) (Super)**

Codes: [Traitement avant Hospitalisation]

No memos

R : C’est toujours Makulumbe

**P11: Ktw_Hgr_Audit_dcd-08_Proche-1.doc - 11:21 [R : C’est une plante qui pouss..] (85:85) (Super)**

Codes: [Traitement avant Hospitalisation]

No memos

R : C’est une plante qui pousse qu’on faisait cuir.

**P12: Ktw_Hgr_Audit_dcd-08_Proche-12.doc - 12:8 [R : Dans le foret qui se trouv..] (34:34) (Super)**

Codes: [Traitement avant Hospitalisation]

No memos

R : Dans le foret qui se trouve à Buinga. A mon retour, elle me demanda de lui chercher un soignant traditionnel. Je lui avais demandé pourquoi, elle m’avait dit que la façon dont elle sentait elle pourrait se sentir mieux si elle était soignée par la médecine traditionnelle. Alors on nous amena un soignant traditionnel qui traite les gastrites, il commença à lui soigner à la maison. Mais son traitement n’était pas efficace par ce qu’il n’y avait pas eu de changement. Alors le tradi praticien m’avait conseillé de l’amener à l’hôpital de Fukangi passer dans un appareil pour voir l’état de l’estomac. Apres examen on lui dira que son estomac avait déjà des ulcères on lui prescrit un médicament pour traiter ces ulcères. Elle continua son traitement qui n’aboutit à aucun résultat positif. Elle rechuta et était obligé de l’amener à l’hôpital Katwa pour les soins approprier. On arrêta les traitements de Fukangi d’abord comme c’était un traitement traditionnel moderne, nous étions arrivés à Katwa à 15h°° on chercha sa veine on avait pas trouvé jusqu’à 20h°° qu’on lui plaça un abord veineux, elle sera perfusée qui finira la nuit. Elle avait commencé à crier à cause des douleurs, sa garde était partie réveillé les soignants pour venir voir le cas du malade ces derniers n’avaient pas accepté de venir voir la malade, même pas pour ouvrir leurs portes enfin de nous écouter. Et la nuit était fini le matin j’étais rentré encore pour leurs demander s’ils peuvent quelque chose sur le patient ils me répondaient qu’ils traitent avec les médicaments prescrits. La fin de la journée on lui amena quelques comprimés alors qu’elle souffrait de la gastrite et les soignants de garde demanda si elle avait déjà fait les examens je répondis que c’est non. Ils s’étonnèrent de l’équipe soignante de la journée s’ils faisaient quoi sans traitement ni examens de laboratoire. On me demanda si on avait prélevé sa tension artérielle je leurs répondis qu’elle est souvent hypertendue. Après la prise de la tension et d’autres signes cliniques, on se rendra compte qu’elle était hypotendue et insuffisance respiratoire ce qui entraina qu’on l’amena aux soins intensifs pendant trois jours sans collaborer et le quatrième jour elle se réveilla. Lorsque je l’avais apporté la bouillie j’avais remarqué que dans ce service il n’y avait pas la prise en charge efficace. J’avais mon neveu qui avait étudié les sciences infirmières à Katwa et qui a son dispensaire dans le milieu et un autre à Béni, je lui avais appelé que maman on ne la traite pas comme il faut et lui me demanda si dans les soins intensifs il n’y avait un médecin ? je lui répondis que c’était des infirmiers seulement et ils ne lui mettent le respirateur seulement et il appela le médecin et lui informa que sa grand-mère était dans les soins intensifs qu’il puisse s’en occuper d’elle. Ce dernier est venu et lui examina de nouveau et demanda au garde malade de lui donner 10dollars pour la radiographie et après celle-ci le résultat concluait qu’elle avait un problème cardiaque. Le médecin s’occupa d’elle et celui que j’avais vu qu’il avait traité vraiment, il lui plaça deux perfusions au même moment avec des ampoules et le lendemain le malade se réveilla un certain vendredi et commença à collaborer, on lui donna la bouillie et même la nourriture en potage. C’était programme que demain elle quitte les soins intensifs la nuit à 19h°° elle rechuta, le médecin était parti, le respirateur manqua l’énergie pour le faire fonctionner comme il n’y avait pas d’électricité et à 20h°° elle décéda.

**P 5: Bbo_HgrKttmba_Audit_dcd-03_Proche-2.doc - 5:12 [R : Je ne sais pas mais lorsqu..] (47:47) (Super)**

Codes: [Traitement avant Hospitalisation]

No memos

R : Je ne sais pas mais lorsque vous leurs dites la maladie ; ils écrivent sur la fiche et eux même donnent le médicament.

**P11: Ktw_Hgr_Audit_dcd-08_Proche-1.doc - 11:20 [R/ Avant de l’amener ici à l’h..] (81:81) (Super)**

Codes: [Traitement avant Hospitalisation]

No memos

R/ Avant de l’amener ici à l’hôpital je le donnais un traitement indigène (makulumbe mélangé au miel).

**P 1: Bbo_HgrKttmba_Audit_dcd-01_Proche-1.doc - 1:8 [RÉPONDANT : Non,] (29:29) (Super)**

Codes: [Traitement avant Hospitalisation]

No memos

RÉPONDANT : Non,

**P 2: Bbo_HgrKttmba_Audit_dcd-01_Proche-2.doc - 2:9 [R : Avant son hospitalisation ..] (41:41) (Super)**

Codes: [Traitement avant Hospitalisation]

No memos

R : Avant son hospitalisation il était soigné à la maison.

**P 2: Bbo_HgrKttmba_Audit_dcd-01_Proche-2.doc - 2:10 [R : On lui achetait le médicam..] (45:45) (Super)**

Codes: [Traitement avant Hospitalisation]

No memos

R : On lui achetait le médicament dans une pharmacie qu’il prenait à la maison et lorsqu’il n’y a pas eu changement on l’avait amené à l’hôpital.

**P18: Ktw_HopMtda_Audit_dcd-06_Proche-1.doc - 18:17 [R. Je ne peux pas connaitre.] (68:68) (Super)**

Codes: [Traitement avant Hospitalisation]

No memos

R. Je ne peux pas connaitre.

**P18: Ktw_HopMtda_Audit_dcd-06_Proche-1.doc - 18:19 [R. Je ne sais pas. Mais il s’a..] (76:76) (Super)**

Codes: [Traitement avant Hospitalisation]

No memos

R. Je ne sais pas. Mais il s’agissait de la pommade anti inflammatoire

**P18: Ktw_HopMtda_Audit_dcd-06_Proche-1.doc - 18:18 [R. Lorsque j’arrivais, je voya..] (72:72) (Super)**

Codes: [Traitement avant Hospitalisation]

No memos

R. Lorsque j’arrivais, je voyais comment elle se massait.

**P18: Ktw_HopMtda_Audit_dcd-06_Proche-1.doc - 18:16 [R. Oui, elle prenait des calma..] (64:64) (Super)**

Codes: [Traitement avant Hospitalisation]

No memos

R. Oui, elle prenait des calmants à la maison et elle était massée avec différentes pommades.

**P11: Ktw_Hgr_Audit_dcd-08_Proche-1.doc - 11:25 [R/je lui avais donné d’autres ..] (104:104) (Super)**

Codes: [Traitement avant Hospitalisation]

No memos

R/je lui avais donné d’autres plante d’herbes qu’on appelle basilique en français et Songe en langue locale, je le faisais bouillir

**P11: Ktw_Hgr_Audit_dcd-08_Proche-1.doc - 11:24 [R/ la maladie s’était aggravée..] (97:97) (Super)**

Codes: [Traitement avant Hospitalisation]

No memos

R/ la maladie s’était aggravée, puis on m’avait dit qu’il faut lui donner le jaune d’œufs mélangé au miel

**P 2: Bbo_HgrKttmba_Audit_dcd-01_Proche-2.doc - 2:13 [R : Oui je lui avais demandé m..] (57:57) (Super)**

Codes: [Traitement avant Hospitalisation]

No memos

R : Oui je lui avais demandé mais il ne m’avait pas répondu.

**P 2: Bbo_HgrKttmba_Audit_dcd-01_Proche-2.doc - 2:11 [R : Non je n’avais pas vu.] (49:49) (Super)**

Codes: [Traitement avant Hospitalisation]

No memos

R : Non je n’avais pas vu.

**P 2: Bbo_HgrKttmba_Audit_dcd-01_Proche-2.doc - 2:12 [R : A chaque fois que je lui v..] (53:53) (Super)**

Codes: [Traitement avant Hospitalisation]

No memos

R : A chaque fois que je lui visitais, je lui demandais s’il prenait les médicaments et il acceptait.

**P16: Ktw_HopMtda_Audit_dcd-05_Proche-2.doc - 16:29 [R. Il parait qu’elle y avait f..] (122:122) (Super)**

Codes: [Traitement avant Hospitalisation]

No memos

R. Il parait qu’elle y avait fait 2 mois

**P 9: Bbo_HopScola_Audit_dcd-07_Proche-1.doc - 9:17 [R : Quand on avait remarqué la..] (78:78) (Super)**

Codes: [Traitement avant Hospitalisation]

No memos

R : Quand on avait remarqué la fièvre et la céphalée, il avait refusé qu’on lui soigne dans une pharmacie.

**P19: Ktw_HopMtda_Audit_dcd-10_Proche-1.doc - 19:15 [R : Non. Elle n’avait pas pris..] (69:69) (Super)**

Codes: [Traitement avant Hospitalisation]

No memos

R : Non. Elle n’avait pas pris des médicaments.

**P 3: Bbo_HgrKttmba_Audit_dcd-01_Proche-3.doc - 3:14 [R : C’était les comprimés par ..] (54:54) (Super)**

Codes: [Traitement avant Hospitalisation]

No memos

R : C’était les comprimés par ce qu’on voulait lui opérer mais ça n’a pas tenu.

**P 5: Bbo_HgrKttmba_Audit_dcd-03_Proche-2.doc - 5:4 [R : Lors que nous sommes arriv..] (15:15) (Super)**

Codes: [Traitement avant Hospitalisation]

No memos

R : Lors que nous sommes arrivés au Poste de santé et quand ils voyaient ses mouvements d’épilepsie on m’avait donné l’huile de palme pour lui parfumer.

**P 3: Bbo_HgrKttmba_Audit_dcd-01_Proche-3.doc - 3:48 [R : Non je ne sais pas.] (190:190) (Super)**

Codes: [Traitement avant Hospitalisation]

No memos

R : Non je ne sais pas.

**P13: Ktw_Hgr_Audit_dcd-08_Proche-2.doc - 13:4 [R : On lui donnait des bactrim..] (8:8) (Super)**

Codes: [Traitement avant Hospitalisation]

No memos

R : On lui donnait des bactrimes, paracétamol, et surtout l’amoxicilline qu’ils lui traitaient avec.

**P 3: Bbo_HgrKttmba_Audit_dcd-01_Proche-3.doc - 3:47 [R : Non pas d’autres médicamen..] (186:186) (Super)**

Codes: [Traitement avant Hospitalisation]

No memos

R : Non pas d’autres médicaments qu’il prenait en dehors.

**P 9: Bbo_HopScola_Audit_dcd-07_Proche-1.doc - 9:48 [R : Elle a le traitement pour ..] (220:220) (Super)**

Codes: [Traitement avant Hospitalisation]

No memos

R : Elle a le traitement pour soigner le poison car elle est spécialisée dans ce domaine.

**P13: Ktw_Hgr_Audit_dcd-08_Proche-2.doc - 13:3 [R : ils avaient donné les médi..] (12:12) (Super)**

Codes: [Traitement avant Hospitalisation]

No memos

R : ils avaient donné les médicaments. Mais je ne me souviens pas les noms. C’était un comprimé de la couleur bleue.

**P 5: Bbo_HgrKttmba_Audit_dcd-03_Proche-2.doc - 5:5 [R : Moi-même je ne savais pas,..] (19:19) (Super)**

Codes: [Traitement avant Hospitalisation]

No memos

R : Moi-même je ne savais pas, je suivais seulement les instructions des prestataires des soins comme je viens de leurs dire que c’est l’épilepsie. Ils m’avaient donné cette huile comme c’est l’épilepsie et moi j’avais exécuté leur recommandation

**P11: Ktw_Hgr_Audit_dcd-08_Proche-1.doc - 11:11 [R : ici on le suivait très bie..] (45:45) (Super)**

Codes: [Traitement avant Hospitalisation]

No memos

R : ici on le suivait très bien malgré que les médicaments n’avaient pas réussi , mais là leur façon de m’accueillir n’était pas bonne ; puis ils m’ont dit que les examens que je viens de faire je dois revenir prendre le résultat après cinq jours mais ils m’ont donné certains médicaments : Ibuprofène, antiramine ; nous sommes rentré ici chez moi à la maison ; mais l’enfant la toux s’est aggravée, après deux jours sans résultat l’enfant a continué sa gravité là j’ai appelé un infirmier d’ici à 2h, quand il est venu ici il a regretté puis il a appelé l’ambulance d’EBOLA, ils m’ont demandé s’il faut aller au CTE ? j’ai dit non il faut aller à KATWE quand nous avons fait deux jours dans le CTE de KATWE ils ont dit qu’il n’est pas malade de EBOLA, directement ils nous ont envoyé à l’hôpital général où nous avons passé les fêtes de Noel et nouvel ans. Nous y avons passé trop de jours car l’Enfant était mort le 22 janvier.

**P14: Ktw_Hgr_Audit_dcd-09_Proche-1.doc - 14:6 [R : Non comme c’était la pério..] (25:25) (Super)**

Codes: [Traitement avant Hospitalisation]

No memos

R : Non comme c’était la période Pique de l’épidémie on nous avait interdit de ne pas prendre aucun médicament, qu’il fallait aller au centre de santé proche pour qu’on vous amené au CTE avant d’être soigné et nous étions obligés de l’amener au CTE et après on nous transporta jusqu’à l’hôpital de Katwa. De ma part je vois que pour la première fois que nous étions arrivé à Katwa et si on avait fait l’échographie on devrait connaitre la maladie et trouver la solution même en dehors de Katwa. Par ce que souvent on fréquente les grandes Fosa en sachant qu’il y a disponibilité de tout l’équipement complet. Et là on peut venir en et étant malade(céphalée)tout sachant qu’on va faire les examens du paludisme et de la fièvre typhoïde. Mais quand on arrive à Katwa et qu’on leur demande de faire l’échographie il faudrait que les soignants nous comprennent. Dans cette période de la MVE je n’ai rien vu d’importance pour moi. Je n’ai plus du courage pour aller me faire soigner dans la gratuité.

**P 5: Bbo_HgrKttmba_Audit_dcd-03_Proche-2.doc - 5:7 [R : Oui eux même m’en avaient ..] (27:27) (Super)**

Codes: [Traitement avant Hospitalisation]

No memos

R : Oui eux même m’en avaient donné celle-là qui lessive pour eux.

**P 5: Bbo_HgrKttmba_Audit_dcd-03_Proche-2.doc - 5:11 [R : On lui traitait bien, quan..] (43:43) (Super)**

Codes: [Traitement avant Hospitalisation]

No memos

R : On lui traitait bien, quand elle était hospitalisé l’enfant qui lui gardait n’avait pas dit quelque chose sur cette maladie mais quand j’y étais arrivée je leurs avais informé qu’elle souffrait de l’épilepsie. C’est moi qui leurs avait donné l’éclaircissement par rapport à cette maladie.

**P11: Ktw_Hgr_Audit_dcd-08_Proche-1.doc - 11:13 [R : Oui dans Cinq jours] (53:53) (Super)**

Codes: [Traitement avant Hospitalisation]

No memos

R : Oui dans Cinq jours

**P11: Ktw_Hgr_Audit_dcd-08_Proche-1.doc - 11:12 [R : Je pense qu’ils devaient c..] (49:49) (Super)**

Codes: [Traitement avant Hospitalisation]

No memos

R : Je pense qu’ils devaient commencés les soins après la sortie de résultat et les résultats avaient trop trainés, 5 jours.

**P 3: Bbo_HgrKttmba_Audit_dcd-01_Proche-3.doc - 3:9 [R : Oui il s’était senti un pe..] (34:34) (Super)**

Codes: [Traitement avant Hospitalisation]

No memos

R : Oui il s’était senti un peu bien.

**P 3: Bbo_HgrKttmba_Audit_dcd-01_Proche-3.doc - 3:10 [R : Non, nous on ne connait pa..] (38:38) (Super)**

Codes: [Traitement avant Hospitalisation]

No memos

R : Non, nous on ne connait pas les médicaments.

**P 5: Bbo_HgrKttmba_Audit_dcd-03_Proche-2.doc - 5:6 [R : Je ne sais pas aussi mais ..] (23:23) (Super)**

Codes: [Traitement avant Hospitalisation]

No memos

R : Je ne sais pas aussi mais lorsqu’on a un malade on accepte tout ce qu’on nous dit comme médicament.

**P 3: Bbo_HgrKttmba_Audit_dcd-01_Proche-3.doc - 3:17 [R : A cette époque oui comme i..] (66:66) (Super)**

Codes: [Traitement pdt Hospitalisation_Difficultés]

No memos

R : A cette époque oui comme il n’y avait pas la gratuité mais lorsqu’on nous a dit qu’il y a la gratuité nous lui avons amené à Kitatumba voilà qu’il ne pas rentrer.

**P 3: Bbo_HgrKttmba_Audit_dcd-01_Proche-3.doc - 3:15 [R : Il y avait difficulté de t..] (58:58) (Super)**

Codes: [Traitement pdt Hospitalisation_Difficultés]

No memos

R : Il y avait difficulté de trouver l’argent pour payer.

**P 3: Bbo_HgrKttmba_Audit_dcd-01_Proche-3.doc - 3:16 [R : Je ne sais pas mais on nou..] (62:62) (Super)**

Codes: [Traitement pdt Hospitalisation_Difficultés]

No memos

R : Je ne sais pas mais on nous disait que c’est l’argent qui manquait.

**P 3: Bbo_HgrKttmba_Audit_dcd-01_Proche-3.doc - 3:60 [R : L’hôpital en soit est bon ..] (242:242) (Super)**

Codes: [Traitement pdt Hospitalisation_Difficultés]

No memos

R : L’hôpital en soit est bon par ce qu’il remplit les conditions ; il y a les soignants de toute catégorie. Ils ne peuvent pas être et qu’il ait un service qui manque. Ce sont les soignants qui avaient le problème de négliger les malades. Même une parturiente(MUKULE) pouvait accoucher au triage à cause de leurs négligences alors qu’elle fréquente la structure souvent et même les malades mouraient dans cette chambre par manque du traitement et d’attendre le résultat du CTE. Comme le papa d’ici à coté on l’avait amené là, il était décédé dans cette salle de triage sans aucun traitement reçu comme ils attendaient ses résultats du CTE

**P 9: Bbo_HopScola_Audit_dcd-07_Proche-1.doc - 9:22 [R : Non, il n’avait rien dit e..] (99:99) (Super)**

Codes: [Traitement pdt Hospitalisation_Difficultés]

No memos

R : Non, il n’avait rien dit et je ne lui avais pas posé cette question.

**P 9: Bbo_HopScola_Audit_dcd-07_Proche-1.doc - 9:21 [R : Je ne sais pas, parce que ..] (94:94) (Super)**

Codes: [Traitement pdt Hospitalisation_Difficultés]

No memos

R : Je ne sais pas, parce que je n’avais pas étudié la médecine.

**P 3: Bbo_HgrKttmba_Audit_dcd-01_Proche-3.doc - 3:18 [R : Il n’y avait pas moyen pou..] (70:70) (Super)**

Codes: [Traitement pdt Hospitalisation_Difficultés]

No memos

R : Il n’y avait pas moyen pour trouver l’argent de l’opération, nombreux étaient mobilisés pour trouver l’argent qui était dépensé pour l’achat de médicaments pour lui et qui était cher.

**P15: Ktw_HopMtda_Audit_dcd-05_Proche-1.doc - 15:35 [R. Non, je n’avais demandé !] (140:140) (Super)**

Codes: [Traitement pdt Hospitalisation_Difficultés]

No memos

R. Non, je n’avais demandé !

**P15: Ktw_HopMtda_Audit_dcd-05_Proche-1.doc - 15:34 [R. C’étaient des médicaments d..] (136:136) (Super)**

Codes: [Traitement pdt Hospitalisation_Difficultés]

No memos

R. C’étaient des médicaments des maladies de muscles. C’est ainsi que l’on a trouvé que ça ne promettait pas.

**P 9: Bbo_HopScola_Audit_dcd-07_Proche-1.doc - 9:18 [R : Peut-être l’anti-malaria p..] (82:82) (Super)**

Codes: [Traitement pdt Hospitalisation_Difficultés]

No memos

R : Peut-être l’anti-malaria parce que je ne regardais pas sur sa fiche, pour connaitre la prescription médicale.

**P 9: Bbo_HopScola_Audit_dcd-07_Proche-1.doc - 9:20 [R : C’est ce que moi je croyai..] (90:90) (Super)**

Codes: [Traitement pdt Hospitalisation_Difficultés]

No memos

R : C’est ce que moi je croyais.

**P10: Ktw_Hgr_Audit_dcd-02_Proche-1.doc - 10:23 [R : Avant il était malade et o..] (93:93) (Super)**

Codes: [Traitement pdt Hospitalisation_Difficultés]

No memos

R : Avant il était malade et on lui avait amené au Centre Hospitalier d’ambiance au centre-ville qui est une structure de la communauté CBCA comme cette hôpital et il était tombé dans une anémie et on avait manqué le sang et parfois dans les banques de sang il y avait rupture. Les soignants qui étaient sur place m’avaient dit de me débrouiller car le patient est dans un dernier tableau et s’il n’y a pas du sang on risque de lui perdre. A la dernière minute on me dira que ceux qui sont chargés de la banque de sang sont rentrés et je m’étais demandé comment les gens peuvent laisser ce service sans permanence alors que c’était un service important ? J’avais fait toute mes démarches pour l’obtention du sang, c’est ce qui était la difficulté liée à cette période.

**P 9: Bbo_HopScola_Audit_dcd-07_Proche-1.doc - 9:19 [R : Il était sous perfusion.] (86:86) (Super)**

Codes: [Traitement pdt Hospitalisation_Difficultés]

No memos

R : Il était sous perfusion.
