# Supplementary material for: Barriers and facilitators to healthcare facility utilization by non-Ebola patients during the 2018–2020 Ebola outbreak in the Democratic Republic of Congo
Source: Glob Health Res Policy. 2024 Nov 19;9:47. doi: 10.1186/s41256-024-00387-6 (PMC11575170; doi:10.1186/s41256-024-00387-6)
Supplement: Supplementary file 8 — Additional file 8. Key Informants Interviews Output files from Atlas-ti. [file 41256_2024_387_MOESM8_ESM.docx]

**All current quotations (1760). Quotation-Filter: All**

______________________________________________________________________

HU: Composantes Système Santé1

File: [C:\Users\Dr Gabriel Kyomba\Documents\Scientific Softwa...\Composantes Système Santé1.hpr7]

Edited by: Super

Date/Time: 2024-05-21 18:53:49

______________________________________________________________________

**P 1: IA_01_AS_IT1.rtf - 1:1 [Répondant : en fait, c’était u..] (4:4) (Super)**

Codes: [Début-Progression_Epidémie MVE]

No memos

Répondant : en fait, c’était une surprise. Ebola, on n’avait jamais entendu même un signe comme ce n’est pas tout le monde qui écouté les radios. C’était vraiment une surprise, en octobre quand on a constaté qu’Ebola était chez nous, en fait déjà déclaré en aout et c’est alors chez nous aussi on avait constaté en octobre qu’on avait le cas ça veut dire qu’on soignait les malades et en ce moment-là les gens étaient tellement malade et nos chambres d’observation étaient vraiment insuffisantes. On avait seulement constaté qu’un malade qu’on avait délivré, s’est amélioré comme on n’attendait pas et après 5 jours, il revient plus abattu qu’avant et puis maintenant avec les signes d’Ebola surtout la diarrhée ; les vomissements, l’anémie et quelques fois la fièvre et il y avait même d’autres qui étaient sortis d’ici et arrivaient à la maison peut-être ils ont dit ça doit être les effets des médicaments et parmi eux il y avait des relais communautaire qu’on avait dépisté positif. C’est ainsi que tous les malades qui nous arrivait et qui pendant cette période était ici chez nous qu’il ait des signes ou pas on a dit qu’il faut investiguer ; heureusement pendant tout ce temps, nous même on travaillait à main nue, c’est ce que nous avons dit que le seigneur vraiment nous a protégé par sa grâce parce qu’aucun de nos infirmiers n’a été suspect pendant cette période et pourtant il y avait la possibilité d’être contaminé par les malades

**P 1: IA_01_AS_IT1.rtf - 1:2 [Répondant : Ebola était vraime..] (6:6) (Super)**

Codes: [Considération_Ebola_population]

No memos

Répondant : Ebola était vraiment de la politique, vous savez avec des tueries, ils n’ont pas pu nous terminer par les machettes et maintenant ils nous ont envoyé la maladie, c’est comme ça qu’on a qualifié cette maladie mais c’est petit à petit qu’on est arrivé à comprendre mais pas en profondeur, la population n’est pas tellement convaincu que c’est une maladie et puis à part la politique, on a dit que les gens veulent s’enrichir. Il y avait ces deux versions-là, Ebola business, Ebola politique d’exterminer les gens enfin que l’ennemi prenait notre terre. C’était ça

**P 1: IA_01_AS_IT1.rtf - 1:3 [Répondant : oui, ceux qui sont..] (8:8) (Super)**

Codes: [Attitude_Equipe_riposte]

No memos

Répondant : oui, ceux qui sont dans la riposte et pourtant ils sont aussi en train de se donner

**P 1: IA_01_AS_IT1.rtf - 1:4 [Répondant : nous là même aujou..] (10:10) (Super)**

Codes: [Considération_prestataire_population]

No memos

Répondant : nous là même aujourd’hui, il y a ceux qui disent, ces gens-là après la riposte on va voir ceux qu’ils vont devenir, on va voir leur argent et leurs biens parce que quand on a quelques choses il faut investir, on verra s’ils vont jouir de leurs biens, ça c’est dans le parle mais les faits on en a pas encore vu, ce sont les rumeurs qui circulent dans la communauté. ils ne nous voient pas bien quand nous passons nous sommes indexés par les gens de la communauté. Ce sont eux qui ont eu de l’argent sur le sang de nos frères et sœurs qui sont morts à cause de cette maladie, ça n’a pas été facile.

**P 1: IA_01_AS_IT1.rtf - 1:5 [Répondant : pour eux leur souc..] (12:12) (Super)**

Codes: [Reproches_aux prestataire_population]

No memos

Répondant : pour eux leur souci était qu’on accueille les malades comme on les accueillait avant alors ils nous disent que vous ne vous inquiétez plus des gens parce qu’il faut d’abord le triage, se vêtir convenablement et dans tout ce temps, une personne peut mourir, il n’y a plus d’urgence, dans Ebola il n’y a pas d’urgence parce que toi et ton malade vous allez partir pour dire si vous partez qui va soigner les autres donc il faut se protéger d’abord, alors c’est cette guerre qui a eu lieu entre la population et les infirmiers

**P 1: IA_01_AS_IT1.rtf - 1:6 [Répondant : au fait il y a eu ..] (14:14) (Super)**

Codes: [Structure-Service_Fermeture_pdt MVE]

No memos

Répondant : au fait il y a eu des structures privées qui ont fermés parce qu’il y avait la gratuité des soins et tout le monde se dirigeait là om il y avait des soins gratuits, alors ils n’avaient pas des clients et puis nous avons beaucoup soufferts parce que d’autres structures qui ont persisté, ils ont séquestré les malades, c’est ainsi qu’il y a eu beaucoup des cas au niveau des structures privées et d’autres qui ont fermés parce qu’on leur avait obligé de fermer parce qu’il y a eu deux ou trois cas d’Ebola qui sont passés par là, les cas testés déjà positifs

**P 1: IA_01_AS_IT1.rtf - 1:8 [Répondant : c’était une instru..] (18:18) (Super)**

Codes: [Service_laboratoire_fonctionnement]

No memos

Répondant : c’était une instruction ; et c’était normalement presque tous les examens parce qu’on suppose qu’avec d’autres-là les malades étaient grave il fallait les transférer, c’est qu’on pouvait faire, c’était seulement l’hémoglobine et le sel et surtout avec la gratuité des soins avec 3,5 si on pouvait se permettre de faire aussi d’autres examens, on ne s’en sortirait pas mais la première chose, c’était d’abord la prévention

**P 1: IA_01_AS_IT1.rtf - 1:9 [Répondant : pendant la gratuit..] (20:20) (Super)**

Codes: [Service_maternité_fonctionnement]

No memos

Répondant : pendant la gratuité, la fréquentation avait haussé comme nous sommes une population démunies aussi et chez nous il y a presque des réfugiés même ceux d’autres zones rouge qui venaient chez nous, en un certain moment quand c’était gratuit, il y avait haussé de cas mais après ça, ça n’a pas tenu parce que tout le monde et aujourd’hui nous avons des difficultés comme cette personne qui a commencé, les veilles-là, ce sont eux qui viennent. Les autres là veulent les luxes, il faut aller chez les Bernardins, il faut aller là où il y a quand même la vie. Et puis avec les références, ils disent il faut aller là-bas on va vous référer et ça aussi ça diminuait les cas de nos patients à la maternité parce que directement si c’est la CPN, elle préférait là où on va l’accueillir facilement, il n’y a pas beaucoup des questionnements, il n’y a pas beaucoup des suivis et nous on a été victime, on n’a dit plus jamais un tel cas sera prise en charge chez nous

**P 1: IA_01_AS_IT1.rtf - 1:10 [Répondant : avec la sensibilis..] (22:22) (Super)**

Codes: [Structure-Service_Utilisation_population]

No memos

Répondant : avec la sensibilisation des relais communautaire, on a quand même bénéficié de quelques choses ou les malades au lieu de s’automédiquer à la maison pouvaient rejoindre la structure où il y avait la gratuité des soins et puis même s’il n’y avait pas moyen de se rendre là-bas on pouvait voir le relais qui passe et lui demandait si on pouvait avoir secours, on ne sait pas aller de soi, est-ce que vous pouvez nous appeler l’ambulance et là comme on ne pouvait pas envoyer l’ambulance sans investigation, on va d’abord investiguer la situation et après on va évaluer le cas et on va appeler m’ambulance pour amener le malade à l’hôpital et puis ils ont aussi reconnu qu’il faut aller au centre de santé de son aire, il ne fallait pas quitter Kasanga pour Malepe ou Malepe pour Kasanga parce que les lettres d’orientation ou de référence, il fallait se retrouver dans son aire de santé et cela aussi nous a aidé

**P 1: IA_01_AS_IT1.rtf - 1:11 [Répondant : le triage, on n’av..] (24:24) (Super)**

Codes: [Service_triage_existence dans structure]

No memos

Répondant : le triage, on n’avait pas

**P 1: IA_01_AS_IT1.rtf - 1:12 [Répondant : l’isolement non, p..] (26:26) (Super)**

Codes: [Service_isolement_existence]

No memos

Répondant : l’isolement non, plus

**P 1: IA_01_AS_IT1.rtf - 1:13 [Répondant : quand on a install..] (28:28) (Super)**

Codes: [Triage-Isolement-Chlore-thermoflash_Réaction_population]

No memos

Répondant : quand on a installé l’eau là avec le chlore, c’était la queue, d’autres qui disent que ça sent mauvais, d’autres qui disent avec votre chlore ici et comme on n’avait pas accueilli la maladie donc on était vraiment méfiant de tout ce qui arrivait dans ce cadre de l’Ebola et comme nous sommes dans une zone un peu minée il y a des forces négatives, ceux-là et quand la personne se disait que moi je ne me lavais pas il ne faut même pas insister, on était vraiment menace et il y a d’autres structures qui ont été brulées alors on avait peur de tout cela

**P 1: IA_01_AS_IT1.rtf - 1:14 [Répondant : chez nous on a eu ..] (30:32) (Super)**

Codes: [Triage-Isolement-Chlore-thermoflash_Réaction_population]

No memos

Répondant : chez nous on a eu la chance

Interviewer : parce que vous étiez libéral, vous n’insistez pas aux gens de se laver les mains ?

Répondant : pas certainement, peut-être nous avons eu beaucoup d’interface au niveau de la communauté, c’est la communauté qui nous a sauvé sinon on était ici dans l’insécurité

**P 1: IA_01_AS_IT1.rtf - 1:15 [Répondant : pour eux, le triag..] (34:34) (Super)**

Codes: [Triage_Considération_population]

No memos

Répondant : pour eux, le triage ça faisait d’abord trainer et puis on voyait le triage que c’était Ebola et quand c’est Ebola, c’était la mort, là vraiment c’est une zone noire, c’est vraiment la mort. C’est qui fait que convaincre la personne pour le triage était très difficile donc vous vous voulez que je puisse mourir donc on mourrait même des stress parce que quand on a dans sa tête le triage, c’est Ebola, la mort, c’est fini. On arrive là-bas on est d’abord fâché d’être transféré là-bas et quand on est transféré là-bas on peut te donner même de l’eau, tu ne sauras pas avaler et c’est ce qui a ramené beaucoup à la mort

**P 1: IA_01_AS_IT1.rtf - 1:16 [Répondant : le CTE, c’est le c..] (36:36) (Super)**

Codes: [Considération_CTE_population]

No memos

Répondant : le CTE, c’est le calvaire parce que là comme je venais de vous dire la maladie était arrivé comme une surprise et puis avec beaucoup des résistances, on y arrivait déjà trop tard et puis même ce qu’on pouvait faire là-bas et comme c’était l’expérimentation de tout et le vaccin et les produits, là c’était la mort. Vous allez au CTE, on va t’injecter, ils avaient un terme, on t’injecte de l’eau et puis tu meurs, là on t’injectait la mort, il ne fallait pas aller là-bas, c’était ça la population. En tout cas on a été très, très résistant

**P 1: IA_01_AS_IT1.rtf - 1:17 [Répondant : pour le thermo fla..] (38:38) (Super)**

Codes: [Considération_thermoflash_population]

No memos

Répondant : pour le thermo flash, on disait vous écouter même le thermo flash parle donc on est en train d’enregistrait vos noms dans ce thermo flash, on les envoie je ne sais pas où, c’était compliqué donc tout était la mort, très difficile, on n’avait âs d’espoir de vie avec Ebola. Même de l’eau avec le chlore, tout était compliqué

**P 1: IA_01_AS_IT1.rtf - 1:18 [Répondant : en fait la qualité..] (40:40) (Super)**

Codes: [Qualité_soins_Prestataires]

No memos

Répondant : en fait la qualité, elle était la qualité mais l’habitude est qu’on avait peur d’interne pour ne pas s’attirait un malade d’Ebola parce que là on pouvait se faire beaucoup de mal et puis il y avait trop des malades, par jour on pouvait même consulter 100 malades et si je consulte 100, il y en a autres sur les lits, 20, 30, vous trouvez les suivis, on se réservait il fallait faire des références alors on a donné les produits per os suivant l’instruction il faut plus donner les per os que les injectables pour éviter tout le risque de se contaminait, on ne saura pas vivre. C’est ce qui fait que la population a taxé de mauvais la qualité des soins parce qu’il n’était pas interne parce que la plupart s’il n’est pas interne donc pour lui il n’a pas reçu les soins et puis c’est déjà psychologique. C’est vrai les comprimés, on ne sait pas les avalent alors que les injections c’est beaucoup plus facile que les comprimés car il y a des gens qui n’arrivent pas d’avaler directement les comprimés. Et la guérison trainait à venir et d’abord la psychologie des gens pensent que les injections guérissent vite que les comprimes et aussi comme on ne pouvait interne les malades, ils étaient aussi déstabiliser et pouvait rechuter à tout moment et pour eux les soins n’était plus de bonne qualité. Et puis on disait que vos produits-là d’Ebola sont empoisonnés. Il en a d’autres après s’être soigné ici pour aller se rendre dans les dispensaires pour chercher des perfusions, la plupart, ils ont faite ça et c’est là où ils se sont infectés

**P 1: IA_01_AS_IT1.rtf - 1:20 [Répondant : aussi la gratuité,..] (44:44) (Super)**

Codes: [Gratuité_Conséquences_utilisation_services]

No memos

Répondant : aussi la gratuité, je ne sais pas s’il faut dire de la responsabilité de ceux qui ont parlé de la gratuité au lieu de se prendre en charge, ils ont baissé les bras mais d’autre part je dis ce n’est pas ça mais ce sont les déplacés qui n’ont pas où allé chercher, ils n’ont pas des moyens et ils ne savaient décider de la situation de la vie et de la maladie donc c’est un peu mélanger. D’autre part il y a cette irresponsabilité et d’autre part il y a la réalité de ce que nous sommes en train de vivre parce qu’il faut aller à Kalingana, c’est là où je travaille mais il y a les militaires et les mai-mai, on ne sait pas quand ils vont se battre, on a peur d’y aller et on meurt de faim ici. On n’a pas dix francs pour aller aux soins et la gratuité des soins pour moi c’est bénéfique

**P 1: IA_01_AS_IT1.rtf - 1:21 [Répondant : c’est le gouvernem..] (46:46) (Super)**

Codes: [Gratuité_Partenaire d'appui]

No memos

Répondant : c’est le gouvernement par PDSS

**P 1: IA_01_AS_IT1.rtf - 1:22 [Répondant : il y a eu un autre..] (48:48) (Super)**

Codes: [Gratuité_Partenaire d'appui]

No memos

Répondant : il y a eu un autre partenaire pour les déplacés parce que PDSS a pris en charge seulement les consultations ambulatoires. Au cas où vous êtes en observation, là vous vous prenez en charge, la maternité c’était avant tout au début là mais le second contrat on avait aussi omis la maternité donc, la CPN mêmement, ils sont venus pour compléter le paquet du PDSS, c’est ainsi que nous avons été dans la gratuité totale

**P 1: IA_01_AS_IT1.rtf - 1:23 [Répondant : Non, le contrat de..] (50:50) (Super)**

Codes: [Gratuité_Evolution]

No memos

Répondant : Non, le contrat de l’IRC était fini au mois de mars

**P 1: IA_01_AS_IT1.rtf - 1:24 [Répondant : ça commençait en o..] (52:52) (Super)**

Codes: [Gratuité_Evolution]

No memos

Répondant : ça commençait en octobre 2019 et jusqu’au mois de mars 2020

**P 1: IA_01_AS_IT1.rtf - 1:25 [Répondant : ça serait PDSS mai..] (54:54) (Super)**

Codes: [Gratuité_Evolution]

No memos

Répondant : ça serait PDSS mais nous avions eu la difficulté de signer le contrat parce que la condition d’avoir la carte d’électeur quoi ou bien c’est la carte d’électeur ou bien la photocopie de la carte de vaccination des enfants et avoir le numéro mais après un moment nous nous sommes dit que nous avons refusé mais vous trouvez que chez nous il n’y a pas des malades et ça fait problème et pourtant il y a beaucoup des malades dans la communauté, c’est ainsi que nous avons fait une lettre de recourt, on ne sait pas si on répondrait ou pas pour que PDSS revienne et nous allons essayer de convaincre la population et eux-mêmes seront témoins parce que quand ils nous amènent l’argent nous soignions les malades ou alors nous vous soignions et vous allez nous donner ceci et il en a d’autres qui sont en train de retourner ou soit s’il a deux enfants, il fait soigner un seule et retourner avec l’autre sans traitement et cela ça fait très mal

**P 1: IA_01_AS_IT1.rtf - 1:26 [Répondant : je ne me rappelle ..] (56:56) (Super)**

Codes: [Gratuité_Evolution]

No memos

Répondant : je ne me rappelle plus mais je pense que c’était de mois d’aout 2018 jusqu’au mois de décembre 2018

**P 1: IA_01_AS_IT1.rtf - 1:27 [Répondant : en aout 2019, il n..] (58:58) (Super)**

Codes: [Gratuité_Interruption]

No memos

Répondant : en aout 2019, il n’y avait pas de contrat avec PDSS

**P 1: IA_01_AS_IT1.rtf - 1:28 [Répondant : la gratuité comme ..] (60:60) (Super)**

Codes: [Gratuité_Rumeurs]

No memos

Répondant : la gratuité comme je vous ai dit avant ce n’était pas bien accueilli par tout le monde parce qu’ils se disent les médicaments-là qu’on est en train de nous donner, ce sont les médicaments d’Ebola, attendent seulement quelques mois ou quelques années et vous allez subir les conséquences et puis avec notre gouvernement avec les tueries, ils ont dit que c’est la manière de nous exterminer et quand il nous amène des produits, bientôt nous allons finir comme des mouches, c’était des rumeurs qui étaient dans la population.

**P 1: IA_01_AS_IT1.rtf - 1:29 [Répondant : ça s’est amélioré ..] (62:62) (Super)**

Codes: [Ressources humaines_Motivation_personnel]

No memos

Répondant : ça s’est amélioré mais ça ne répondait pas au travail qu’on rendait parce que par mois vous pouvez consulter une affaire de 3000 personnes et puis il faut voir comment les gens ont travaillé, c’est vrai il y avait beaucoup des fatigues mais ça n’a pas donné le prix du travail que les agents ont rendu durant le mois parce qu’il y avait beaucoup de travail et c’était fatiguant mais la motivation n’était pas à la hauteur avec les stress, il y a eu un peu des défaillances par rapport à ça. S’il y avait un forfait, c’allait être mieux mais on recevait selon qu’on consultait les malades et puis on était fatigué. Si vous avez reçu deux milles consultations et vous aurez aussi comme deux milles de frais et avec le fonctionnement, les médicaments et la motivation du personnel mais quand même ça relevait un peu la motivation du personnel

**P 1: IA_01_AS_IT1.rtf - 1:30 [Répondant : les médicaments, q..] (64:64) (Super)**

Codes: [Médicaments_disponibilité_fosa]

No memos

Répondant : les médicaments, qu’est-ce que je peux dire par rapport aux médicaments parce que la disponibilité, on pouvait mais avec beaucoup des nuances, avec PDSS on a 30% des recettes pour les médicaments mais à partir du mois d’avril ils nous ont retiré le 20% de nos factures pour les médicaments mais quand nous faisons les réquisitions, il y a un peu de souci donc le temps de la livraison, elle est vraiment compliqué mais on pouvait vraiment avoir autre chose pour se procurait d’abord ce dont on a besoin et puis le prix par rapport à d’autres officines, c’est un peu compliqué, c’est qui fait qu’on a beaucoup des difficultés par rapport à l’approvisionnement en médicaments

**P 1: IA_01_AS_IT1.rtf - 1:31 [Répondant : En fait nous peut-..] (66:66) (Super)**

Codes: [Ressources_humaines_Disponibilité et perturbations]

No memos

Répondant : En fait nous peut-être on n’a pas été consulté et nos demandes n’ont pas eu toutes les réponses positives, nous avons eu seulement une personne qui est allée travailler dans la riposte, elle a préféré rester là-bas parce que notre structure au niveau du BDOM a dit que si quelqu’un part à la riposte doit donner les 25% de son salaire pour essayer de contribuer à celles-là qui sont restées travailler dans la structure, mais celle qui était partie quand on lui avait donné cette note circulaire, elle n’a pas pu répondre positivement, c’est pourquoi elle est restée là-bas et puis comme on a été consulté ou utilisé, nous nous sommes dit qu’on ne saura pas avec deux personnes au triage et il fallait avoir plus des consultations par jour avec plus des 100 malades, ce n’était pas facile, c’est comme ça que nous avons cherché deux personnes de plus pour essayer de nous épauler dans la structure pour qu’on ait au moins un temps de se reposer parce que sinon on pouvait craquer quand la personne parle et il faut qu’une personne puisse aller se reposer à la maisons pendant une ou deux semaines ?

**P 1: IA_01_AS_IT1.rtf - 1:32 [Répondant : ils vont retourner..] (68:68) (Super)**

Codes: [Ressources_humaines_Disponibilité et perturbations]

No memos

Répondant : ils vont retourner chez elles donc c’est un contrat à durée déterminée et nous avons-nous tous enduré car personne n’est partie

**P 1: IA_01_AS_IT1.rtf - 1:33 [Répondant : ils ont compris qu..] (70:70) (Super)**

Codes: [Ressources_humaines_Disponibilité et perturbations]

No memos

Répondant : ils ont compris que c’est une période qui va passer et si on va perdre ce qu’on a déjà et allait là où on ne sait pas ce que l’avenir nous réserve ; ils ont été sage

**P 1: IA_01_AS_IT1.rtf - 1:34 [Répondant : ça était tellement..] (72:72) (Super)**

Codes: [SNIS_fonctionnement_Decrire_pdt MVE]

No memos

Répondant : ça était tellement lourd, au niveau des registres, ça pouvait se faire, la compilation des données ok et pour le remplissage des canevas, comme c’est obligatoire et l’envoie au bureau central se faisait dès fois avec beaucoup de retard parce que ça arrivait le 7 qui était même la date limite, on avait pas encore fini parce qu’on ne pouvait pas abandonner les malades et puis aller faire le rapport, c’était vraiment difficile et comme nous étions aussi dans l’épidémie, il y avait aussi des visites à temps et à contre temps et puis il faut contrôler le cas et je n’ai rien fait et tout cela c’est le temps qui se perd. Et puis pour l’analyse des données, on n’a pas analysé les données mais seulement si on voyait des incohérences donc pour se mettre et commencer à discuter on n’avait pas le temps donc on était coincé. Quand il y a un cas, c’est à ce moment que la riposte met ses yeux et tout se focalise là-bas, il faut suivre les contacts, la vaccination et puis avec le suivi des contacts ce n’était pas facile et les autres qui vint dire que nous on ne veut pas vous voir ici, il fallait aller soutenir cette équipe. Donc on était comme des folles, on n’était pas stable. On était vraiment toutes joyeuses quand on disait que c’est la fin de l’épidémie et on souffle un peu et cas on avait un cas positif, on était vraiment malade tous les 21 jours parce qu’on n’était pas stable, les appels d’ici et de partout de presque tout le monde donc on n’a pas le temps de se mettre et de réfléchir sur autre chose que c’est point-là de cas positif d’Ebola. Aussi avec la vaccination, c’était un peu compliqué donc la vaccination-là de routine

**P 1: IA_01_AS_IT1.rtf - 1:36 [Répondant : oui, au niveau de ..] (76:76) (Super)**

Codes: [PCI_Situation]

No memos

Répondant : oui, au niveau de la structure, la PCI a été vraiment renforcé même au niveau de la communauté

**P 1: IA_01_AS_IT1.rtf - 1:37 [Répondant : les mamans avaient..] (74:74) (Super)**

Codes: [Service_vaccination_Fonctionnement]

No memos

Répondant : les mamans avaient difficile d’amener leurs enfants parce qu’ils ont dit, on a d’abord refusé le vaccin d’Ebola, on avait d’abord cette difficulté, elles ont dit que ce sont ces mêmes vaccins-là, ils veulent injecter nos enfants et après six ans ou vingt ans vont devenir inutiles et en ce moment-là pour venir s’installer chez nous. C’est une haute politique, la stérilité et tout ça là, on a parlé de tout ça et on se dit d’où vienne toutes ses idées dans la tête des gens donc cette première blessures de la guerre que nous avons sur les esprits et nous sommes dans une zone de santé où il y a beaucoup des illettrés et ce sont des ruraux et une partie vit seulement en ville et même si on est en ville mais nous ne sommes pas de la ville

**P 1: IA_01_AS_IT1.rtf - 1:38 [Répondant : c’est là le grand ..] (78:78) (Super)**

Codes: [PCI_Comment_maintenir]

No memos

Répondant : c’est là le grand problème. En fait c’est l’idéal mais on ne sait pas comme les épis coutent chers. L’eau, on peut en avoir en achetant un tanker pour conserver l’eau de la pluie et là on aura un peu de savon pour se laver les mains, là ça peut aller comme avant on se lavait les mains, ce n’était pas à la porte mais dans les services, il en avait. Pour les gants, ce n’était seulement à la maternité et dans la laboratoire mais au niveau de l’hospitalisation et de l’observation, ce n’était pas tellement sauf en cas de diarrhée peut-être, c’est là où on pouvait se dire que je ne peux pas toucher ses affaires et on porte les gants. Aujourd’hui là on ne sait pas mais avec le plan d’action, on se dit qu’on va acheter autant des gants mais il faut avoir de l’argent, comment on va faire, est-ce qu’on va exiger aux malades de venir avec les gants au centre de santé or ce n’est pas possible, et ce cas nous dit que nous ne sommes pas épargner d’une nouvelle épidémie, c’est qui nous est arrivé ici c’est une leçon

**P 1: IA_01_AS_IT1.rtf - 1:39 [Répondant : c’est un grand déf..] (80:80) (Super)**

Codes: [PCI_Comment_maintenir]

No memos

Répondant : c’est un grand défis, on ne sait pas comment mais l’idéal serait de garder ça

**P 1: IA_01_AS_IT1.rtf - 1:40 [Répondant : en fait avant même..] (82:82) (Super)**

Codes: [Souhait_Changement_gestion_futures_épidémies]

No memos

Répondant : en fait avant même que cela n’arrive, il faut renforcer la communication par rapport aux risques et il faut aussi réunir les leaders communautaires pendant la gestion de l’épidémie, les leaders d’opinion, on les réuni et on leur dit voilà ce qu’on a constaté ceci qu’est-ce que nous pensons et les gens donnent leurs idées et celui qui a une idée très bien, on la capitalise et puis on les soumet à la communauté et moi je me dis c’est mieux comme on a initié les CAC mais malheureusement chez nous le tout a mal commencé parce qu’on mélangeait tout avec Ebola et donc dans nos CAC, on n’a pas des personnes qui sont vouées au bénévolat, là il n’y a pas des effets. Mais avec les élections de nouveaux membres, je pense que les choses vont aller de l’avant surtout avec la sensibilisation et puis la communauté aura un sens positif par rapport aux structures de CAC parce que maintenant là on est en train de dire que ce sont les gens d’Ebola et même ce qui vont dire ça n’a pas d’impact positif o la communauté parce que la majorité n’a pas encore donné foi à ces structures-là alors que ce sont ces structures qui vont nous aider à bien gérer des pareilles histoires.

**P 1: IA_01_AS_IT1.rtf - 1:41 [Répondant : ce que ne peux ajo..] (84:85) (Super)**

Codes: [Souhait_repondant]

No memos

Répondant : ce que ne peux ajouter à ce que je dis par rapport à ce que nous avons vécu par rapport à l’épidémie, c’est un mal mais un mal nécessaire parce que tous ce points de PCI, ce n’était pas objectifs dans nos structures et aujourd’hui chacun sait qu’on ne peut pas toucher les malades à main sans couvert et on ne peut plus réutiliser les trousses parce qu’avant on réutiliser parce que ça s’achète et même le brakster, on réutilisait. C’est vraiment l’acquis que nous avons eu malgré que c’est arrivé dans une période critique. C’est un acquis et nous espérons qu’on va continuer avec ces bonnes manières, cette même pratique pour sauvegarder notre propre vie et celle de notre communauté parce qu’il n’y a pas seulement Ebola mais il y a d’autres pathologies qui sont aussi transmissible par le sang et d’autres prescriptions corporelles

Ce que je peux dire si des pareilles épidémies venaient, il faut qu’il ait des fonds pour éviter ce que nous avons connu ici, il faut faire participer tout le monde parce que c’est arrivé comme si c’était une part d’autres personnes donc tout le monde n’était pas intéressé

**P 1: IA_01_AS_IT1.rtf - 1:42 [Répondant : quand je dis tout ..] (87:87) (Super)**

Codes: [Souhait_repondant]

No memos

Répondant : quand je dis tout le monde, nous sommes dans la structure mais nous avons aussi des structures privées parce que les soins gratuits étaient seulement chez nous et puis si les soins gratuits ont été chez nous, on pouvait prendre les structures privées et on le met dans les suivis des contacts pour le faire participer dans la riposte

**P 1: IA_01_AS_IT1.rtf - 1:43 [Répondant : la gratuité des so..] (42:42) (Super)**

Codes: [Gratuité_Accueil_population]

No memos

Répondant : la gratuité des soins, par les vulnérables, ça était bien accueilli mais ceux-là qui peuvent se procurer les soins, ils n’ont vraiment pas accueilli cette gratuité. Ils se sont soignés là où il n’y avait pas la gratuité

**P 1: IA_01_AS_IT1.rtf - 1:44 [Répondant : tous les services ..] (16:16) (Super)**

Codes: [Structures-Services_fonctionnement]

No memos

Répondant : tous les services n’ont pas fonctionné correctement parce qu’il y avait des précautions, il ne fallait pas par exemple la CPS, il ne fallait pas peser les enfants parce que c’est la même culotte que tous les enfants pourra porter et le risque de la contamination est possible donc c’était un peu compliqué, même le TDR on le faisait plus parce qu’avec le sang, il y avait un danger.

**P 2: IA_01_BCZS_AG.rtf - 2:1 [Répondant : juste au début la ..] (4:4) (Super)**

Codes: [Considération_Ebola_population]

No memos

Répondant : juste au début la population ne comprenait vraiment pas d’autant plus que la zone n’avait jamais connu une épidémie, ça faisait plus de 9 ans alors quand l’épidémie est venue tellement qu’on était confronté à un problème de l’insécurité, la population a vu une masse importante des partenaires qui arrivaient à la zone avec les véhicules et cela ça impressionnait la population, la population se dit nous sommes en train d’être massacré, nous n’avons vu une mobilisation pareille des partenaires maintenant aujourd’hui on nous parlait de Ebola mais il y a une mobilisation intense des partenaires, c’était des questions, qu’est-ce qu’il y a derrière Ebola. Autre chose, c’est ce qui a entrainé même des résistances juste au début. Et aussi la population a vu d’autres communautés, les gens qui venaient du niveau central et des expatriés qui ne maitrisaient d’abord pas la langue alors pour que ces gens avaient des difficultés pour s’entretenir avec la population, c’est pourquoi on avait récupéré les autochtones pour qu’ils interviennent dans la riposte contre l’épidémie et on avait pris l’équipe cadre de la zone de santé de Beni pour faciliter ça au moins chaque membre de l’équipe cadre avait reçu une commission et moi j’étais affecté comme président de la commission logistique, il y a Damien qui était dans la surveillance, il y a Ernest qui était dans la vaccination, le MCZ qui était aussi dans la surveillance et tant d’autres.

**P 2: IA_01_BCZS_AG.rtf - 2:3 [Répondant : le service avait f..] (8:8) (Super)**

Codes: [Structures-Services_fonctionnement]

No memos

Répondant : le service avait fonctionné par exemple la vaccination avait fonctionné mais au niveau de laboratoire, il y avait quelques examens qui ne pouvaient pas être réalisés tel que le TDR pour le paludisme ça était arrêté, on nous avait demandé d’utiliser le TDR sauf c’était utilisé maintenant au laboratoire de l’INRB et pour éviter la confusion entre le paludisme et la MVE, on avait fait une campagne de sensibilisation pour la distribution des médicaments contre le paludisme donc c’était des mesures préventives du fait que toute personne qui aurait le paludisme serait assimilé à la MVE

**P 2: IA_01_BCZS_AG.rtf - 2:4 [Répondant : la gratuité tout a..] (10:10) (Super)**

Codes: [Gratuité_Structures-Services_concernés]

No memos

Répondant : la gratuité tout au début juste pour préparer la gratuité, on nous avait demandé de préparer un état de besoin des médicaments. Avec l’appui de PDSS, les médicaments étaient arrivés et la gratuité avait commencé dans les structures intégrées, à part l’aire de santé de Sayo, le centre hospitalier de Beni, centre hospitalier évangélique Beni et de Nyakunde, les deux structures n’avaient pas bénéficié de la gratuité. Pour le CH Beni, ils n’ont pas donné les données financières alors le rapport qu’il nous avait transmis, il y avait des problèmes. Le montant qu’il avait estimé de réaliser de 4 millions de dollars et quand on avait envoyé les dossiers au niveau de la PDSS pour sélectionner les structures, on avait constaté qu’on ne pouvait pas réaliser cette somme par mois et pour Sayo, là c’était une erreur, on était en train de faire les plaidoiries mais ça n’avait pas tenu mais vers la fin qu’on avait récupéré Sayo dans la gratuité et Medair avait récupéré Sayo et le centre de santé Payida. Alors depuis aout 2018 jusqu’à décembre 2018, là la gratuité était vraiment totale, c’est vers janvier 2019 qu’on a commencé à supprimer certains actes et on a laissé d’autres donc la gratuité était pour certains actes et pour d’autres on avait supprimé la gratuité. Nos structures avaient dit elles-mêmes qu’avec la somme que PDSS nous donne, on peut continuer avec la gratuité et cela nous suffira et vous verrez certains centres de santé qui offrent la gratuité et certains actes ont été retirés mais ces structures avaient toujours continué à travailler

**P 2: IA_01_BCZS_AG.rtf - 2:6 [Répondant : ce n’est pas unifo..] (14:15) (Super)**

Codes: [Gratuité_Partenaire d'appui]

No memos

Répondant : ce n’est pas uniforme par exemple Halima, quand Halima est venu à l’hôpital général parce que PDSS était à l’hôpital général quand Halima est venu, PDSS s’est retiré même PDSS avant intervenait pour toutes les actions mais peu après il s’est retiré pour certains actes, il a dit que moi je veux intervenir pour la pédiatrie dans son ensemble et la gynécologie et la médecine interne alors la chirurgie restait en souffrance. Halima était en train-là croyant que PDSS allait rester là aussi et ils pouvaient maintenant travailler en synergie mais quand PDSS a constaté que Halima est déjà là il s’est retiré. Halima est là pour une gratuité globale et il fournit même la nourriture au malade alors qu’avant PDDS lui venait vous facturez les actes et il vous paie sur base de la facture tandis que Halima lui, il n’y a pas de facturation, ce sont des forfaits. Et pour l’enveloppe des prestataires, je veux payer autant, pour les médicaments, je veux amener les médicaments d’autant et pour les fournitures pour assurer le fonctionnement, je veux amener 2500 par exemple, ce sont des forfaits contrairement à PDSS lui qui payait sur base de la facture donc on achète les performances. Il y a des vérificateurs qui pouvaient se présenter là ils vérifient vraiment si cet acte avait eu lieu et le malade existe et dès qu’ils vont valider la facture, c’est alors qu’ils vont payer la facture.

MSF, c’est comme Halima lui c’est global il n’y a pas des performances, Medair aussi la même chaose

**P 2: IA_01_BCZS_AG.rtf - 2:7 [Répondant : la gratuité a été ..] (17:18) (Super)**

Codes: [Gratuité_Rumeurs]

No memos

Répondant : la gratuité a été bien accueillie par la population puisque vous allez constater dans les structures où la gratuité a été installée la fréquentation et l’utilisation des services avaient augmenté en flèche et même pour les structures elles-mêmes la gratuité était bien accueillie par les structures parce que les structures ont beaucoup profité de l’apport du PDSS pour réaliser quelques projets. Quand PDSS payait les factures pour les frais de fonctionnement et d’investissements, Kanzuli récupère cette somme pour acheter des briques pour en faire une clôture, Kanzuli a déboursé une somme de 7500$ pour acheter une parcelle, on a fait même une fondation en brique qui est là pour préparer la nouvelle aire de santé.

Si vous arrivez à Butingi, il y a un bâtiment en étage qui est l’œuvre de PDSS donc les fonds que PDSS amène quand il payait la facture, les structures elles-mêmes gèrent ces fonds de manière a ce que ça pouvait aider pour le fonctionnement et à l’investissement, achetait d’autres matériels et la prime du personnel, c’était versait vers le personnel et la parie médicaments vers les médicaments.

**P 2: IA_01_BCZS_AG.rtf - 2:9 [Répondant : pour le moment, il..] (22:22) (Super)**

Codes: [Gratuité_Défis]

No memos

Répondant : pour le moment, il y a des défis parce qu’avec PDSS, il y a une partie qui n’est plus prise en compte, la chirurgie n’est plus supportée et autre chose vous verrez par exemple l’hôpital général de Beni, c’est un grand hôpital et avec Halima seulement, il y a toujours des problèmes, les médicaments qui sont toujours consommés par les malades, même le fonctionnement qui est pénalisé et on donnait quelque chose comme quelques 2500$ alors si on pouvait avoir un autre partenaire qui pouvait se joindre à Halima pour supporter d’autres charges ça pouvait aider dans le fonctionnement de l’hôpital.

**P 2: IA_01_BCZS_AG.rtf - 2:10 [Répondant : souvent à la fin d..] (24:24) (Super)**

Codes: [Gratuité_Partenaire d'appui]

No memos

Répondant : souvent à la fin du contrat. Le problème de PDSS, c’est quoi ? PDSS lui avant il ne paie pas cache, c’était après quand il y a eu beaucoup des réclamations mais quand les prestataires disent que nous avons terminés déjà deux mois et c’est vers novembre que PDSS a pris la résolution de payer chaque fois les cautions exorbitants et les structures consomment progressivement de façon que le contrat pouvait même se clôturer et les structures devaient à PDSS. Avec ce système-là il a été constaté que PDSS n’amenait plus ces cautions, c’est pourquoi les structures se sont désengagées pour trouver d’autres partenaires et quand PDSS comme c’est à la fin du contrat, PDSS pouvait encore revenir mais il trouvait un autre partenaire dans sa politique comme il ne pouvait pas travailler avec un autre partenaire, il se désengage

**P 2: IA_01_BCZS_AG.rtf - 2:11 [Répondant : c’est comme si tou..] (26:26) (Super)**

Codes: [Gratuité_Partenaire d'appui]

No memos

Répondant : c’est comme si tout le fonds provenait de la banque mondiale alors que quand PDSS récupère ces fonds à la Banque Mondiale, Halima je pense aussi que c’est la Banque Mondiale, je ne sais pas donc une structure ne pouvait pas bénéficier de deux fonds qui venaient d’un même bailleur.

**P 2: IA_01_BCZS_AG.rtf - 2:12 [Répondant : par exemple pour c..] (28:28) (Super)**

Codes: [Gratuité_Existence_plan_désengagement]

No memos

Répondant : par exemple pour chaque partenaire, là on définit dans le contrat pour se désengager, par exemple pour PDSS pour désengager des structures qui étaient dans PDSS malheureusement on a un nouveau cas, il était prévu qu’ici au mois de juin, le contrat devait finir alors ces structures pouvaient être accompagnées par un autre partenaire dans le cadre de développement et ça ne serait plus dans le cadre des urgences

**P 2: IA_01_BCZS_AG.rtf - 2:13 [Répondant : dans les centres d..] (30:30) (Super)**

Codes: [Gratuité_Information_population]

No memos

Répondant : dans les centres de santé, il y a des Cosa, il y a des relais communautaires, à travers ceux-là et autre chose on avait informé la population à travers les médias qu’il y a la gratuité que les gens viennent se faire soigner gratuitement, si quelqu’un a des céphalées qu’il ne tarde pas de venir au soins parce que s’il tardait et si c’est le cas d’Ebola vous risquerez de le perdre mais s’il arrivait vite là on aurait le moyen de le récupérer

**P 2: IA_01_BCZS_AG.rtf - 2:14 [Répondant : ici chez nous tout..] (32:32) (Super)**

Codes: [Médicaments_disponibilité_fosa]

No memos

Répondant : ici chez nous toutes nos structures intégrées s’approvisionnent toujours auprès de la CDER mais aussi il y a des pharmacies que nous apprécions bien et en ce moment les structures venaient et acheter et nous aussi on partait et on acheter à la CDER

**P 2: IA_01_BCZS_AG.rtf - 2:15 [Répondant : je dirais que les ..] (34:34) (Super)**

Codes: [Médicaments_disponibilité_fosa]

No memos

Répondant : je dirais que les médicaments étaient disponibles parce que les médicaments qu’on ne trouve pas à la CEDR, on allait les récupérer dans les pharmacies locales

**P 2: IA_01_BCZS_AG.rtf - 2:16 [Répondant : donc nous-mêmes on..] (36:36) (Super)**

Codes: [Ressources_humaines_Disponibilité et perturbations]

No memos

Répondant : donc nous-mêmes on devait qu’on soit à la sous coordination et ici donc le temps de nous concentrer dans une activité nous était difficile, les conditions de travail ne sont pas réunies, on nous avait délogé de notre bureau pour récupérer ce bâtiment et il y a de personnel qui sont partis dans les ONG

**P 2: IA_01_BCZS_AG.rtf - 2:17 [Répondant : pour pallier à ça ..] (38:38) (Super)**

Codes: [Ressources_humaines_Disponibilité et perturbations]

No memos

Répondant : pour pallier à ça nous nous sommes organisés autrement et par exemple ici à la pharmacie, si le pharmacie n’est pas là, on avait affecté deux personnes pour nous remplacer donc il y avait toujours une personne pour récupérer la situation en cas d’une absence

**P 2: IA_01_BCZS_AG.rtf - 2:18 [Répondant : ça ne peut pas man..] (40:40) (Super)**

Codes: [Ressources humaines_Motivation_personnel]

No memos

Répondant : ça ne peut pas manquer mais on a fait s’il y a quelque chose que tout le monde puisse se retrouver parce qu’on avait fait que chaque personne soit représentée dans chaque commission.

**P 2: IA_01_BCZS_AG.rtf - 2:19 [Répondant : juste au début com..] (42:42) (Super)**

Codes: [Réunion_pdt-épidémie-Ebola]

No memos

Répondant : juste au début comme on n’avait pas d’expérience sur la gestion de l’épidémie, il y avait des soucis mais à partir de janvier, on a dit qu’il serait mieux qu’on puisse récupérer la situation jusqu’à nos jours, c’est pourquoi quand moi je ne suis pas là le MCZ est là et quand le MCZ n’est pas là, moi je suis là et s’il y a des réunions Coges ou ECZ là on s’organise

**P 2: IA_01_BCZS_AG.rtf - 2:20 [Répondant : juste au début-là,..] (44:44) (Super)**

Codes: [SNIS_fonctionnement_Decrire_pdt MVE]

No memos

Répondant : juste au début-là, c’était compliqué à partir du deux aout 2018 jusqu’à décembre là c’était compliqué et surtout qu’on avait des massacres qui se commettaient, là il y avait des souffrances mais à partir de janvier 2019, en tout cas on a récupéré la situation et avec le système d’information sanitaires avec des réunions avec la validation des rapports et on se présentait le rapport SNIS à la hiérarchie

**P 2: IA_01_BCZS_AG.rtf - 2:21 [c’est vers le 28 juillet que n..] (46:46) (Super)**

Codes: [Début-Progression_Epidémie MVE]

No memos

c’est vers le 28 juillet que nous MCZ Beni avait tenu une réunion extraordinaire quand on avait appris qu’à Mangina il y a des cas de mortalité élevée et qui présentaient de saignement et tout consort et là on avait cru que ça serait la maladie Ebola, c’est pourquoi il avait initié une réunion extraordinaire, on a appelé le maire de ville, les protections civiles, toutes les couches sociales de la ville étaient là et c’était un certain dimanche et tout le monde était parti à Mangina, heureusement qu’il y avait des épis et en ce moment on avait un véhicule qui était fonctionnel, on a investigué et on se dit que ça doit être Ebola, l’équipe est retourné et on a fait rapport au maire de la ville, c’était dimanche et le lundi on a fait une autre réunion et le mardi une équipe est venue de Butembo de DPS qui est arrivé ici et nous leurs avons dit qu’on avait déjà fait une descente et qu’on suspecte la maladie Ebola et ce mardi qu’on avait pris l’échantillon pour que ça soit parti à Kinshasa et le mercredi l’échantillon était parti à Kinshasa et dans la réunion, il y avait un seul partenaire, c’est le MSF. Sur le six échantillon, 4 étaient positifs et deux négatifs. Et c’est à partir dès là qu’on se dit que c’est l’épidémie d’Ebola et le 02, le ministre national avait déclaré l’épidémie, c’est à, partir du deux au quatre, il y a eu l’arrivée des partenaires et c’était comme cela qu’on avait accueilli l’épidémie dans la zone. Et le quartier général était implanté là à l’hôtel Okapi et c’est à partir de là que les gens se rendaient à Mangina. Quand les gens quittaient Mangina pour arriver ici, ils parlaient de la sorcellerie qu’on a consommé le chat d’autrui et toute autre chose et on avait déjà trois cas ici qui étaient aussi hospitalisés et nous avons eu l’intervention des partenaires et on les avait transféré au CTE, d’autres étaient récupère et d’autres étaient morts

**P 2: IA_01_BCZS_AG.rtf - 2:22 [Répondant : la grève avait com..] (46:46) (Super)**

No codes

No memos

Répondant : la grève avait commencé au mois de mai 2018, alors juin, juillet,

**P 2: IA_01_BCZS_AG.rtf - 2:23 [Répondant : par exemple à l’hô..] (50:50) (Super)**

Codes: [PCI_Comment_maintenir]

No memos

Répondant : par exemple à l’hôpital général là on n’a déjà un comité d’hygiène qui est chargé de suivre tous ces aspects, au niveau des centres de santé, les Recos et les CAC sont informés de tous ses aspects et les IT, on les rappelle que c’est qu’on nous a fait pendant cette période de l’épidémie quelque chose de positif, il faut que ça soit pérenne et ce qui est négatif, il ne faut pas que ça puisse revenir donc comme on n’a pas des moyens, nous faisons des rappels et quand on fait des supervisions, il y a le superviseur qui est chargé des affaires d’assainissements et tout consort, il est chargé de passer sur tous ces aspects

**P 2: IA_01_BCZS_AG.rtf - 2:24 [Répondant : moi je pense que s..] (52:52) (Super)**

Codes: [Souhait_repondant]

No memos

Répondant : moi je pense que selon l’épidémie qu’on vient de gérer toute suite, d’abord sur le plan barème donc le facteur de motivation, si vous analysez un peu ce barème, vous allez constater qu’il y avait un décalage, un écart significat entre les locaux, les provinciaux, les nationaux parce qu’un national touchait 150$ par jour, un provincial c’était 90$ mais un local quel que soit le chef, c’était 20$ par jour, le second 15$ et le dernier 10$ alors que vous partagez les mêmes risques, prochainement moi je pense qu’il serait mieux qu’on puisse penser à ça, c’est vrai qu’on ne peut pas être égal mais il faut que les écarts disparaissent et autre chose, si une autre épidémie pouvait se présenter mais il faut avoir le temps de prendre en considération les locaux donc il ne faut pas venir avec le style d’écraser les locaux parce que ce sont les autochtones qui maitrisent plus le terrain, s’il veut vous bloquer, il va aussi facilement vous bloquer et s’il veut vous aider, il peut aussi facilement vous aider parce qu’au début quand on disait que riposte business, c’est vrai parce que quand les partenaires étaient venus au lieu d’intégrer directement les locaux mais il y avait des petites frustrations, les locaux ont été intégré avec un petit retard, on avait constaté qu’il y avait des résistances parce que vous amenez quelqu’un qui ne maitrise pas la langue local et il va s’exprimer dans une langue étrangère devant une population qui n’a pas étudier ou qui ne comprend pas cette langue et ici par exemple chez nous on parle d’une manière douce mais il y a d’autres qui parlent à haute voix et la population peut croire qu’on est en train de tonner sur elle

**P 2: IA_01_BCZS_AG.rtf - 2:25 [Répondant : si j’ai quelque ch..] (54:54) (Super)**

Codes: [Souhait_repondant]

No memos

Répondant : si j’ai quelque chose à ajouter, c’est la prise en charge des agents après cette activité donc après Ebola, il y a des agents au BCZ et à l’hôpital général qui étaient restés en train de travailler donc eux n’ont pas bénéficié des avantages de la riposte si on peut songer à eux, si c’est une gratification, si c’est qui ça peut les motiver et voir les gens qui se sont vraiment donné dans la riposte si on peut les promouvoir pour étudier et si ils peuvent aller même à l’école de santé et même ailleurs pour des gens qui n’ont pas encore bénéficié de cette matière, ça peut nous encourager tous

**P 2: IA_01_BCZS_AG.rtf - 2:26 [Répondant : PDSS avait pris to..] (12:12) (Super)**

Codes: [Gratuité_Partenaire d'appui]

No memos

Répondant : PDSS avait pris toutes les structures qui n’avaient pas des partenaires puisqu’on avait Medair qui appuie aussi Payida et après il a récupéré aussi Sayo. Avec l’afflux des partenaires, là où les partenaires se présentaient, les PDSS se retiraient directement comme H Halima qui est au niveau de Bunzi donc il y a certaines structures que PDSS n’appuient plus parce qu’ils ont d’autres partenaires. Comme à Malebe, il y a MSF et Makanzulu, PDSS n’est plus là comme il y a maintenant Halima, à Wangoma PDSS n’est plus là il y a MSF, à Boekene PDSS n’est plus là, parce qu’il y a un partenaire là. Et le centre de santé de Kasana a écrit une lettre pour réintégrer le processus de la gratuité parce qu’avant ils ont dit qu’ils n’étaient pas d’accord avec la façon dont le PDSS les payait. Il voudrait qu’il soit intégré dans le projet PDSS

**P 2: IA_01_BCZS_AG.rtf - 2:27 [Répondant : il y a beaucoup d’..] (48:48) (Super)**

Codes: [Gouvernance_soins santé]

No memos

Répondant : il y a beaucoup d’expérience que moi j’ai beaucoup acquis à partir de là parce que je vous ai informé que depuis toujours on n’a jamais géré une épidémie. A partir d’eux, on a su comment gérer une épidémie mais quelque jour avec l’appui de ces partenaires et d’autres personnes, nous sommes maintenu jusqu’à ce jours et là actuellement on sait s’il y a un agent qui aurait fait une faute, on doit d’abord analyser dans quelle circonstance cette personne a commis une faute. Raison pour laquelle ensemble, nous avons dit qu’il faut qu’on enlève la suspension de notre chef. Parce que nous avons dit qu’on l’a suspendu mais il a fallu d’abord qu’on analyse les circonstances dans laquelle il a fait une faute dans la communication parce que ce n’était pas facile, nous étions d’abord dans la période de l’épidémie et maintenant l’épidémie dont on parlait maintenant se présente et en un seul coup il y avait des résistances des cas suspects qui ne voulaient pas venir au centre, c’est ainsi qu’on a trébuché un peu, on pensait qu’on allait récupérer la population pour qu’elle ne se contamine pas mais voilà qu’il est suspendu. Moi je me dis avec cette expérience, je ne pouvais pas prendre cette décision, on devait penser dans le cas social. On appelle la personne pour lui dire que ce n’est pas comme ça que vous alliez procéder, sachant bien que si vous le suspendez ça va créer des rumeurs au niveau de la population et avec cette rumeur, ça va maintenant créer une résistance et avec cette résistance, la maladie peut maintenant se propager dans la communauté. Alors que si ça devrait être géré d’une manière sociale, on pouvait tout simplement le reprocher ou faire un rappel à l’ordre, prochainement, il ne faut plus faire cela et c’est comme ça que nous cogérons ici. Dans cette période de l’épidémie, il est dit que l’agent doit se présenter chaque à 7h30’ à son lieu de travail, on sait bien que nous venons de réaliser plus d’une année dans l’épidémie, les gens sont stressés et autre chose, nous sommes en insécurité, on a massacré nos frères et on continue toujours à les massacrer, est-ce que tu vas exiger coute que coute à une personne de se présenter tous les jours à 7h30’ ? Alors qu’il est dans une insécurité et il vient de sortir dans une période de l’épidémie qui venait de faire plus d’une année, quelque part il est aussi fatigué et quand il se réveille, je ne crois pas qu’il va se réveiller chaque jour à 6h00’ pour prendre bain et s’apprêter pour être là à 7h30’, je ne sais alors quand il se présente, je ne sais pas à 8h00’, je m’entretiens avec lui pour savoir pourquoi il est arrivait à 8h en son lieu de travail et moi-même je sais qu’il est stressé, il est fatigué, on adouci le problème et on passe, c’est comme ça qu’on doit arrangeait les problèmes

**P 2: IA_01_BCZS_AG.rtf - 2:28 [Répondant : la gratuité avec P..] (20:20) (Super)**

Codes: [Gratuité_Structures-Services_concernés]

No memos

Répondant : la gratuité avec PDSS, non, mais il y a certains partenaires qui intervenaient pour ces structures privées, c’est par exemple PPSP qui intervenait dans au moins 16 structures et il payait des salaires aux agents pour installer les kits PCI et autres choses donc il y a des partenaires qui intervenaient même dans les structures privées parce qu’il avait été constaté que les cas positifs provenaient de ces structures privées alors il a fallu qu’on puisse prendre en charge ces structures privées pour qu’ils ne puissent pas séquestrer les malades dans leurs maisons

**P 2: IA_01_BCZS_AG.rtf - 2:29 [Répondant : au début de la cri..] (6:6) (Super)**

Codes: [Structures-Services_fonctionnement]

No memos

Répondant : au début de la crise, toutes les actions étaient convergées vers les actions intégrées, les structures privées étaient délaissées ce quand on a constaté que plusieurs cas positifs provenaient des structures privées, c’est ainsi on dit non, non il serait mieux qu’on puisse récupérer quelques structures privées donc qu’on les intéresse donc il fallait qu’on forme le personnel des structures privées, qu’on parlait avec eux des méfaits d’Ebola parce qu’il y a plusieurs structures vraiment où il y a eu des prestataires qui étaient morts par exemple la cliniques les Bernardins là où on a perdu des prestataires et il y a d’autres prestataires où les prestataires et le médecin directeur ont été atteint de cette maladie

**P 2: IA_01_BCZS_AG.rtf - 2:30 [Répondant : dans la riposte co..] (2:2) (Super)**

Codes: [Répondant_Responsabilité_pdt MVE]

No memos

Répondant : dans la riposte contre Ebola, j’étais président de la commission logistique au niveau de la zone de santé dans la sous coordination de Beni

**P 3: IA_01_BCZS_IS.rtf - 3:1 [Répondant : la population a d’..] (8:8) (Super)**

Codes: [Triage_Considération_population]

No memos

Répondant : la population a d’abord eu peur de la zone d’isolement comme il y a le triage, là où on sucrine les malades. Si quelqu’un est suspect là on le met dans l’isolement et cela a été difficile pour la population de comprendre pourquoi je dois être isolé et on me retire des autres malades et les autres malades fuient la bonne filière, la bonne route pour la consultation mais moi je suis arrêté au niveau du triage alors ça faisait peur à beaucoup des malades et ça fait que les structures n’ont pas été fréquentées. Heureusement les partenaires sont venus nous aider, les triages ont été partout même chez les tradipraticiens, on devait sucrine quelqu’un et on a construit des isolements partout

**P 3: IA_01_BCZS_IS.rtf - 3:2 [Répondant : les malades en cra..] (10:10) (Super)**

Codes: [Triage_Considération_population]

No memos

Répondant : les malades en craignant les isolements sont allés vers les tradipraticiens où on n’avait pas encore construits des isolements alors quand on a mis des isolements partout, il y a eu des points focaux au niveau de chaque structure, c’est maintenant que la population a fini par comprendre que le zone de triage est partout, si vous allez même chez un tradipraticien vous rencontrerez une zone de triage, si ça ne répond pas aux normes, il y avait quand même quelqu’un qui sucrine les gens avant qu’ils aient dans d’autres services de cette structure-là

**P 3: IA_01_BCZS_IS.rtf - 3:3 [Répondant : Ebola comme c’étai..] (12:12) (Super)**

Codes: [Considération_Ebola_population]

No memos

Répondant : Ebola comme c’était la toute première fois qu’à Beni nous puissions gérer l’épidémie d’Ebola, ça n’a jamais arrivé depuis que la RDC a commencé à déclarer les épidémies d’Ebola, c’était la toute première fois alors même si à l’école on nous demandait de porter les épis, c’était la première fois même pour nous prestataires de porter les épis. Alors la population quand ils voyaient les gens portaient les épis au niveau des structures, elle disait que cette maladie, elle est dangereuse mais comme elle était traumatisée par la guerre, les massacres, il y a eu aussi les évènements de décembre lors des élections, on nous a fait rater les élections à cause de cette maladie à cause de cette épidémie alors la population se dit que c’est une maladie politisée, on a vu que les ADF ne nous ont pas exterminé et maintenant on nous a amené cette maladie pour exterminer les gens qui ont résisté aux massacres des ADF donc c’était dans un milieu où la population était trop traumatisée, elle ne faisait plus confiance aux autorités des entités administratives

**P 3: IA_01_BCZS_IS.rtf - 3:4 [Répondant : la population, le ..] (14:14) (Super)**

Codes: [Agression-Menaces_Structure-Prestataire_raisons]

No memos

Répondant : la population, le jour où Nangaa a déclaré que les villes de Beni et Butembo vous n’allez pas voter à cause de la propagation de l’épidémie, la population s’est attaquée aux structures des soins et surtout aux zones de triage, il y a beaucoup des structures qui ont été vandalisées. Je peux citer dans la zone de santé de Beni, le poste de santé Malepe, le centre de santé Rwangoma qui a été rendu en cendre, il y a le centre de santé Ngongolio, centre hospitalier Kamende en tout cas c’était du catastrophe

**P 3: IA_01_BCZS_IS.rtf - 3:5 [Répondant : c’était premièreme..] (16:16) (Super)**

Codes: [Agression-Menaces_Structure-Prestataire_raisons]

No memos

Répondant : c’était premièrement à cause de la CENI et la présence de l’épidémie

**P 3: IA_01_BCZS_IS.rtf - 3:6 [Répondant : les prestataires, ..] (18:18) (Super)**

Codes: [Agression-Menaces_Structure-Prestataire_raisons]

No memos

Répondant : les prestataires, c’était avant que cette situation arrive surtout les équipes de surveillance, quand les équipes de surveillance arrivaient dans un milieu, ils venaient par exemple avec six véhicules Land Cruiser et la population se disait, on avait des massacres ici même un Kamaz, un camion des militaires n’était pas déployé au lieu des massacre pourquoi avec un seul cas d’Ebola dans un quartier, il y a six Land Cruiser pour ne fut-ce que poser une question, quand est-ce que la personne est tombée malade, la date de début des symptômes, c’était quand ? Soit en voulant convaincre le malade à accepter à être transféré au CTE

**P 3: IA_01_BCZS_IS.rtf - 3:9 [Répondant : l’accueil et la pr..] (24:24) (Super)**

Codes: [Qualité_soins_Prestataires]

No memos

Répondant : l’accueil et la prise en charge, ça était un peu difficile parce que nos centres de santé la plupart les bâtiments étaient vétuste, les malades venaient très nombreux, il y a eu même insuffisance des capacités au niveau de l’hôpital général, il y a une partie de l’hôpital général qui a été pris par le labo de l’INRB et une partie, c’était là où on avait pris le premier cas, où on avait isolé les deux premiers cas, cette partie-là a été inhabitée par les malades de l’hôpital, ça était occupé par le labo INRB, la vaccination là où on avait mis le congélateur et les frigo pour les vaccins contre la MVE. Dans les centres de santé, il y a le centre de santé comme Mabakanga ; Karl Becker, c’est un centre hospitalier, il arrivait dès fois où on mettait les malades par terre alors comme il y avait un balcon, on a essayé de couper ça en compartiments pour avoir de la place

**P 3: IA_01_BCZS_IS.rtf - 3:10 [Répondant : les malades ont ét..] (26:26) (Super)**

Codes: [Qualité_soins_Prestataires]

No memos

Répondant : les malades ont été géré suivant l’ordre, celui qui arrivait à temps recevait les soins aussi avant mais les soins de qualité, il est impossible pour quelqu’un d’examiner 60 cas par jour, on peut en moyenne consulter 20 à 30 personnes mais à partir de la 40^ème^ personne, celle-là ne sera plus consultée comme il faut donc les soins vont déjà chutés à partir de la 40^ème^ personne

**P 3: IA_01_BCZS_IS.rtf - 3:11 [Répondant : Bon ce qui ont été..] (30:30) (Super)**

Codes: [Structures-Services_fonctionnement]

No memos

Répondant : Bon ce qui ont été vandalisé en décembre 2018 n’ont pas fonctionné presque deux mois, ils ont récupéré à partir de mois de février avec des hangars qui sont restés au niveau des centres de santé

**P 3: IA_01_BCZS_IS.rtf - 3:12 [Répondant : pour la vaccinatio..] (32:32) (Super)**

Codes: [Structures-Services_fonctionnement]

No memos

Répondant : pour la vaccination, nous on a continue pour la vaccination, c’est au niveau du labo où on nous a interdit de faire le TDR pour le paludisme, ça nous a été toujours difficile de comparer les données du paludisme avec les médicaments distribués mais la vaccination nous avons continué en respectant les mesures de prévention donc on ne devait pas attrouper les mamans, on faisait la vaccination au contact, une maman qui venait avec son enfant, on vérifie s’il a besoin de quelle antigène et on lui donne cette antigène après la maman rentre ainsi de suite. On évitait les attroupements comme dans covid-19 comme on ne peut pas être plus de 20

**P 3: IA_01_BCZS_IS.rtf - 3:13 [Répondant : la chirurgie a con..] (34:34) (Super)**

Codes: [Structures-Services_fonctionnement]

No memos

Répondant : la chirurgie a continué, il y a eu des structures où on a opéré des cas qui ont été confirmés après intervention. Il y a une structure où on avait fait un curetage pour évacuer les restes placentaires mais après la maman avait trompé les infirmiers de cette structure disant que non l’enfant a connu un avortement, c’était une fille de 17 ans alors qu’il y avait des restes placentaires, après le curetage, le lendemain l’enfant a saigné de partout et on l’a envoyé au CTE et il a été confirmée positive, alors quand on interviewe les prestataires, on les a mis d’abord en quarantaine pendant 21 jours, sous surveillance pendant 21 jours, après les 21 jours, nous avons dit voyons le résultat du vaccin parce que tous ceux qui ont été vaccinés n’ont pas été contaminés.

**P 3: IA_01_BCZS_IS.rtf - 3:14 [Répondant : nous avons des act..] (36:36) (Super)**

Codes: [Structures-Services_fonctionnement]

No memos

Répondant : nous avons des activités de JS3, journée santé, sécurité, sexualité, c’est là où nous avions demandé aux jeunes de ne plus venir fréquentés les structures pour ne plus se contaminer de rester dans la communauté, de nous aider à sensibiliser la population en leur disant que la MVE était une réalité et que ça existe au niveau de la zone de santé de Beni mais le service de la planning pour mettre les implants, le dépôt provera ça continuait sans problème

**P 3: IA_01_BCZS_IS.rtf - 3:15 [Répondant : oui, sans problème..] (38:38) (Super)**

Codes: [Structure-Service_Utilisation_population]

No memos

Répondant : oui, sans problème, ils ne craignaient pas seulement à l’hôpital général là où les prestataires qui prenaient en charge les PVV, il est allé chez Halima sans pourtant faire la remise et reprise avec quelqu’un d’autre alors comme nous avons toujours parlé de guichet unique pour la tuberculose et VIH, il y a eu quelqu’un de bonne volonté qui a pris à charge ces malades-là malheureusement les CDV était à coté de là où on avait casé les premiers malades des cas confirmés alors quand on est venu décontaminé les chambres où étaient les malades, on a brulait toutes nos fiches, toutes les fiches de nos malades PVV, pour reconstituer les dossiers de tous ces malades, ça nous a donné beaucoup des difficultés

**P 3: IA_01_BCZS_IS.rtf - 3:16 [Répondant : il y a eu des pert..] (40:40) (Super)**

Codes: [Ressources_humaines_Disponibilité et perturbations]

No memos

Répondant : il y a eu des perturbations partout comme je venais de commencer à l’hôpital dans le service de VIH heureusement qu’il y a eu quelqu’un de bonne volonté qui s’est occupé de ces malades-là donc à l’hôpital il y a eu des prestataires qui ont quitté l’hôpital vers le CTE, ils doivent travailler pour un organisme qui payait bien. Au BCZ, il y a par exemple le plan d’action de 2019 où les activités de l’hygiène, eau et assainissement qui n’ont pas été planifiées parce que le TRD, l’AC tous étaient dans la riposte, j’étais l’unique infirmier superviseur qui était resté au BCZ en permanence. J’allais à la PCI quelque fois et la PCI ne me tenait pas rigueur raison pour laquelle su vous voyez nos données dans le DHIHD, nous avons quand même une complétude qui est de 95,8% suite à une structure qui est fermée à cause de la guerre. La promptitude, il y a un cadre de la DPS qui a touché à nos données et qu’il a rendu notre promptitude très bas surtout pour ce qui est de mois de juillet, je ne sais pas ce qu’il cherchait dans ces données-là mais surtout nous nous sommes efforcés en encoder nos données SNIS mais c’était une charge parce que j’étais seul au BCZ pour encoder, s’il y avait quelques stagiaires pour les encadrer afin qu’ils puissent nous aider à encodé ces données

**P 3: IA_01_BCZS_IS.rtf - 3:17 [Répondant : cette grève-là n’a..] (42:42) (Super)**

Codes: [Ressources_humaines_situation_grève]

No memos

Répondant : cette grève-là n’a jamais été levée et nous n’avons eu gain de cette grève donc la grève a été levée pour que nous puissions prendre en charge d’abord les malades, c’est notre population. La grève, nous suspendons ça et jusqu’à présent, il n’a jamais eu quelqu’un qui dit vous avez grevé et il y a eu l’épidémie et voici nous avons tenu compte de vos revendications jusqu’à présent alors si la maladie est arrivée, moi je condamne les médecins qui n’ont rien fait parce que c’est nous les infirmiers qui étaient lésés pour les médecins ne voulaient pas faire les MEPEPI donc les maladies à potentiels épidémiologiques, ils devraient surveiller ça aussi parce que nous on se dit que nous on n’envoie plus les rapports, c’était au niveau des structures mais pas au niveau du BCZ. On leur demandait envoyer nous et nous on va séquestrer au niveau du BCZ, ils ont dit non, nous gardons les MAPEPI donc tous les rapports, on les a gardés au niveau des structures. Alors quand l’épidémie est entrée nous leur avons dit non, nous allons continuer à encaisser les rapports, envoyé nous les rapports et la grève est à moitié lever à cause d’Ebola, envoyé nous d’abord quelques rapports afin qu’on commence en encoder alors quelques structures ont envoyé les rapports et cela était une lourde charge pour nous pour encoder tous les rapports et les faire remonter à la coordination

**P 3: IA_01_BCZS_IS.rtf - 3:18 [Répondant : par quelques-uns o..] (48:48) (Super)**

Codes: [Gratuité_Rumeurs]

No memos

Répondant : par quelques-uns oui, il y a des populations démunies qui n’ont pas la capacité de se payent les soins, ils se sont dit que ça c’est pour les pauvres alors comme on était appauvri par la guerre et les massacres, la population a accepté cette gratuité facilement. Actuellement, la gratuité est difficilement acceptée parce qu’on a dit qu’il faut venir avec une carte d’électeur, on prenait les soins gratuits chez PDSS là où les centres de santé sont appuies par PDSS

**P 3: IA_01_BCZS_IS.rtf - 3:19 [Répondant : là vous voulez par..] (50:50) (Super)**

Codes: [Médicaments_disponibilité_fosa]

No memos

Répondant : là vous voulez parler de la satisfaction de notre commande par rapport à la CEDR, la CERD ne nous a jamais satisfait, la satisfaction est de 50 à 60%, on n’a jamais atteint 80%. Alors, il y a eu un moment où PDSS avait retenu 30% donc c’est reparti uniquement pour les médicaments, il avait retenu ça et il avait donné cela à la CERD Astramex mais quand il nous avait envoyé les produits, c’était 5 fois supérieur que ceux du Astramex alors quand nous avions posé la question chez Astramex, on dira non les produits viennent de Kinshasa jusqu’à Goma et de Goma maintenant jusqu’à Musiemene alors on dit les mêmes produits comme le sérum glucosé et autres vous amenez de Kinshasa alors que ça eut à coté et à un prix très exorbitant, heureusement avant 2020, nous avions écrit une lettre au PDSS et à la CERD le Coges avait écrit une lettre que les médicaments sont très chers s’il peut voir comment envoyer les 30% à la CEDR et non lui envoyé des médicaments, alors qu’ils ont donné un prix qui est passable donc les factures sont déjà révisées

**P 3: IA_01_BCZS_IS.rtf - 3:20 [Répondant : on ne peut pas cha..] (52:52) (Super)**

Codes: [Traitement_Invasifs_Prestataire_changement]

No memos

Répondant : on ne peut pas changer les prescriptions, elles restent les mêmes. Au contraire comme il y a eu plusieurs formations sur la gestion des médicaments, il y a moins des centres de santé qui continuent à faire des prescriptions irrationnelles. La plupart des structures utilisent des ordinogrammes

**P 3: IA_01_BCZS_IS.rtf - 3:21 [Répondant : ce qui a beaucoup ..] (54:54) (Super)**

Codes: [SNIS_fonctionnement_Decrire_pdt MVE]

No memos

Répondant : ce qui a beaucoup souffert c’est l’analyse à la base donc on nous envoyait les données sans pourtant les analysées donc les gens n’avaient pas de temps, si vous avez un cas positif MVE dans votre aire de santé, vous IT vous allez souffrit les appels viendront de partout alors l’IT n’avait plus le temps de concentrer sur les consultations même le rapportage, analyser les rapports se faisaient difficilement

**P 3: IA_01_BCZS_IS.rtf - 3:22 [Répondant : la tenue des réuni..] (56:56) (Super)**

Codes: [Réunion_pdt-épidémie-Ebola]

No memos

Répondant : la tenue des réunions pour nous au BCZ, on a toujours fait violence, on a essayé d’analyser les données mois par mois et le Coges on a essayé aussi de se rattraper malheureusement ce qui nous a écœuré au bureau PDSS nous donnait un forfait de 4400$ comme il y avait la prime et le fonctionnement alors il y a eu un moment à partit juillet-aout 2019 donc septembre, octobre, novembre et décembre, il fallait qu’on fasse une performance, on vient nous coter selon les performances, si nous avions eu 10%, on nous donne le 10% de 4400$ alors ça nous a déjà découragé et ce dernier temps si vous venez au bureau, on se dit qu’il faut qu’on nous donner le zéro que nous méritons parce qu’on sait casser les jambes, on sait époumoner mais à la fin on nous dira que vous n’avez rien fait*

**P 3: IA_01_BCZS_IS.rtf - 3:23 [Répondant : normalement il fal..] (60:60) (Super)**

Codes: [Souhait_repondant]

No memos

Répondant : normalement il fallait que chaque membre de l’ECZ reste membre de l’ECZ alors qu’on lui accorde une prime comme faisant partir de la riposte parce que les gens ont fui les BCZ pour aller là où il y a l’argent et nous qui sommes restés nous regrettons pourquoi nous ne sommes pas allés vers la riposte totalement et moi je ne faisais que quelques heures mais je n’étais pas payé comme tous les autres. Alors si une telle épidémie comme ça vient de recommencer aujourd’hui qu’on ramène tous les gens du BCZ vers les BCZ et les gens qui supportent la riposte nous payer de la même façon que les gens qui sont dans la riposte. Au lieu de prendre les membres de l’ECZ et dès les considèrent comme membres effectifs de la riposte mais restent là chez vous et faites votre travail comme il faut selon votre plan d’action ou votre plan trimestriel comme ça à la fin du mois on nous payer comme les autres qui sont dans la riposte mais si on ne le fait pas, on risque tous de quitter les BCZ pour profiter dès là où il y a l’argent et on reviendra après

**P 3: IA_01_BCZS_IS.rtf - 3:24 [Répondant : autre chose, la ri..] (62:62) (Super)**

Codes: [Souhait_repondant]

No memos

Répondant : autre chose, la riposte a mal commencé. Les gens qui viennent de la Guinée, de Kinshasa et d’ailleurs ne connaissent pas la langue locale et s’il y avait plusieurs résistances, c’était à cause de ça, vous venez vacciner les gens qui parlent swahili ou kinandé alors vous vous s’adresser à eux a lingala ou en français et il y avait un langage de sourds et les malades étaient frustrés au lieu de les préparer comme vous avez présenté les signes comparable ou proche de la MVE nous proposons que nous appelons l’ambulance et ainsi de suite, en tout cas soyez calme, ,vous aurez rien, vous serez dans les bonnes mains quand vous serez au CTE mais ils sont venus comme des militaires même celui de la DPS celui qui est chargé de la surveillance, lui-même avait dit à la mairie quand les gens de Kinshasa vont venir je serai là trop loin, il était dans la PCI à Mangina et alors nous qui étions au BCZ

**P 3: IA_01_BCZS_IS.rtf - 3:25 [Répondant : le niveau sera mai..] (64:64) (Super)**

Codes: [PCI_Comment_maintenir]

No memos

Répondant : le niveau sera maintenu su les gens sont dotés des épis, si les gens sont dotés en intrants, il y avait des structures ou par exemple où le partenaire IRC qui prenait en charge et un partenaire ASSP qui prenait en charge et qui venait de se désengager d’ici le 11 avril, ces structures-là vont chuter si elles ne sont pas prise en charge et quand on va leur demander d’aller acheté les gants, c’est très difficile car l’argent viendra d’où ? Alors que nous sommes dans la gratuité, il faut que les partenaires continuer à nous appuyer pour maintenir ces performances que les structures ont obtenues durant la riposte

**P 3: IA_01_BCZS_IS.rtf - 3:28 [Alors ce que j’ai oublié dans ..] (70:70) (Super)**

Codes: [Riposte_Investissements]

No memos

Alors ce que j’ai oublié dans la riposte avec PDSS, la plupart de nos structures ont au moins un chantier même si c’est encore au niveau de la fondation mais c’est le 20% qui a été alloué au fonctionnement et le 10% à l’investissement donc le personnel prenait 40%, les médicaments 30%, investissement 10 et le fonctionnement 20%. Alors avec les 30%-là, il y a des aires de santé qui étaient en semi durable mais ils ont érigé des fondations, ils sont au niveau des linteaux. C’est ça que je peux souligner comme point positif partout où PDSS avait appuyé.

**P 3: IA_01_BCZS_IS.rtf - 3:29 [Il y a deux structures qui n’é..] (70:70) (Super)**

Codes: [Structure_appui]

No memos

Il y a deux structures qui n’étaient pas dans PDSS même trois, il y a dans l’aire de santé de Sayo qui était un peu éloignée de la ville, il y a l’aire de santé de Payida où il y avait un partenaire Medair et le centre de santé Mukulya, ce centre de santé Mukulya n’ont pas bénéficié de la gratuité de PDSS, il y a les deux qui étaient appuyé par Medair et l’autre qui était délaissé.

**P 3: IA_01_BCZS_IS.rtf - 3:30 [Répondant : la raison était qu..] (72:72) (Super)**

Codes: [Structure_appui]

No memos

Répondant : la raison était que comme il y avait un partenaire qui prenait les gens gratuitement, il fallait que celui-là continue alors que lui donnait peu d’argent, il donnait 1000 ou 1300$ aux prestataires et il amenait les médicaments alors que PDSS amenait les médicaments et il achète les prestations. Si vous avez 1000 nouveaux cas, vous avez 3500$ et dans les 3500$, vous avez le 40% pour le personnel, 20% pour le fonctionnement, 10% pour l’investissement et le 30% pour les médicaments

**P 3: IA_01_BCZS_IS.rtf - 3:31 [Répondant : par exemple qui a ..] (74:74) (Super)**

Codes: [Structure_appui]

No memos

Répondant : par exemple qui a été délaissé, celui de Sayo parce qu’en ce moment-là on kidnappait les gens alors quand on avait envoyé la liste à Kinshasa, on se dit que le vérificateur va se rendre chaque fois à Sayo ? Est-ce qu’on ne va pas le kidnapper alors cette structure-là a été omise. Payida et Mukulya comme il y avait un autre partenaire qui prenait en charge la population dès là, on ne devait pas avoir deux partenaires dans une même structure, raison pour laquelle Payida et Mukulya n’ont pas bénéficié de la gratuité de PDSS, il y a même des structures qui n’ont pas atteint 50% de taux d’utilisation à cause d’excentricité de ces centres de santé dont Ngongolio et Busindji donc ces structures à chaque réunion d’analyse des données mensuelles, on tonnait sur elles parce que comment ils n’arrivaient pas à atteindre 50% de taux d’utilisation alors qu’il n’y avait plus de centre de santé qui était à moins de 50%, il n’y avait que les deux-là et l’autre Sayo qui n’était pas appuyée. Actuellement, Halima va aller à sayo et quand Medair va se désengager mais je ne sais pas la date où Medair va se désengager de là.

**P 3: IA_01_BCZS_IS.rtf - 3:32 [Répondant : au contraire Ebola..] (68:68) (Super)**

Codes: [Agression-Menaces_Structure-Prestataire_raisons]

No memos

Répondant : au contraire Ebola a amené des conflits dans la communauté. Moi je suis infirmier, j’ai par exemple ma belle-sœur qui a présenté des signes et je dis à mon beau-frère amenons ce malade au CTE, on me présente une résistance et quand j’essaye de convaincre cette résistance, quand la personne arrive au CTE et elle meurt, on me dira que toi tu as tué ta belle-sœur parce que tu es entrain de recevoir beaucoup d’argent et d’ailleurs quand tu nous a convaincu, on t’a donné 200$ raison pour laquelle tu as amené notre sœur au CTE et voilà qu’elle vient de mourir. Il y a eu des conflits pareils dans la communauté.

**P 3: IA_01_BCZS_IS.rtf - 3:33 [Autre chose, nous qui sommes d..] (69:69) (Super)**

Codes: [Agression-Menaces_Structure-Prestataire_raisons]

No memos

Autre chose, nous qui sommes dans la riposte, nous sommes pointés du doigt et on nous dit qu’attendons que cette Ebola finisse, ils verront ce que nous leur ferons, est-ce qu’on va démolir nos petites maisonnettes, on ne sait pas soit on va nous envoyer les mains armées, on ne sait pas mais on nous dit cela chaque jours dans le quartier.

**P 3: IA_01_BCZS_IS.rtf - 3:34 [Répondant : les structures n’o..] (22:22) (Super)**

Codes: [Structures-Services_fonctionnement]

No memos

Répondant : les structures n’ont pas été complétement fermées surtout les structures privées, ils ont travaillé difficilement parce qu’elles n’ont pas eu l’occasion d’avoir un partenaire pour les soins gratuits mais pour la PCI, la prévention et le contrôle des infections, ils sont positionnés pour eider les formations privées à construire les zones de triage et d’isolement et à prendre à charge les personnes affectées dans ces structures

**P 3: IA_01_BCZS_IS.rtf - 3:35 [Répondant : je veux commencer ..] (20:20) (Super)**

Codes: [Structures-Services_fonctionnement]

No memos

Répondant : je veux commencer par les structures étatiques, dans les structures étatiques il y a eu des partenaires qui ont assuré les soins gratuitement alors les structures ont été utilisées par la population malgré la présence de l’épidémie était là, la population était déjà appauvrie et n’avait plus d’argent et quand la population tombait malade disait que même on refuse d’aller là où on a construit les zones de triage ou d’isolement nous n’avons pas d’argent alors allons dans les structures là où on a mis la gratuité. Par exemple la zone de santé avant l’épidémie, par mois, on faisait en moyenne on avait noté à 20% le taux d’utilisation alors en période d’épidémie nous sommes arrivés en moyenne de 75 à 80% de taux d’utilisation par mois. Même les accouchements on arrivait difficilement à 30% d’accouchements par rapport à la population de la zone. Pendant l’épidémie avec PDSS, le premier contrat que PDSS avait signé avec les structures, on prenait tout à charge alors les accouchements nous sommes allés à 40, à 50% de taux d’accouchements assistés au niveau des structures. Alors vers le milieu de 2019, ils se sont dit non, non on prend seulement les nouveaux cas, vers juillet 2019 PDSS prenait seulement en charge les nouveaux cas s’il était validé par les vérificateurs de PDSS, on devrait payer 3,5 par nouveau cas alors les accouchements et autres services étaient à la charge du malade, c’est en ce moment que le taux d’occupation a un peu chuté

**P 3: IA_01_BCZS_IS.rtf - 3:36 [Répondant : pendant la riposte..] (4:4) (Super)**

Codes: [Répondant_Responsabilité_pdt MVE]

No memos

Répondant : pendant la riposte, j’étais dans la commission PCI comme superviseur, c’est ce que j’ai fait pendant la riposte

**P 3: IA_01_BCZS_IS.rtf - 3:37 [Répondant : il s’agissait de l..] (6:6) (Super)**

Codes: [Répondant : il s’agissait de l..]

No memos

Répondant : il s’agissait de la prévention et contrôle des infections, on devait vérifier au niveau des structures, ils ont des épis donc des équipements de protection, s’ils ont des lavabos, s’il y a de la distance entre deux lits pour que les malades ne puissent pas se contaminer entre eux, est-ce qu’il y a un incinérateur pour la destruction des déchets, il y a le trou à ordures, trou à placenta, fosse à cendre ainsi de suite.

**P 4: IA_01_COMM_LR.rtf - 4:1 [Répondant : Le début de l’épid..] (4:4) (Super)**

Codes: [Début-Progression_Epidémie MVE]

No memos

Répondant : Le début de l’épidémie nous l’avons vécu de façon frustrante presque l’épidémie est tombée dans le contexte des massages et quand l’épidémie est venue, c’était comme une clou dans une plaie au niveau de la population même pour nous même qui gardons la population, c’est comme ça que ça était difficile à accueillir puisque les gens ont mal interprété le fait, et l’épidémie tuait et dans le milieu dans lequel elle est apparue, à Mangina, le milieu rural et aussi ce qui a créé une panique si pas comme une doute, c’est que jusqu’à présent on a jamais expliqué à la population l’origine vraie de cette épidémie d’Ebola au niveau de Mangina parce que, c’était avant dans la province de l’Equateur et puis d’un coup de Bikoro de l’Equateur jusqu’au Nord-Kivu, précisément encore à Mangina, une bourgade non loin de Beni ici. Alors on racontait, je dis bien on racontait mais jusqu’à un moment donné les gens n’avaient pas compris et jusqu’aujourd’hui on n’a jamais accepté. Les gens prennent ça comme une maladie importée ou bien même fondée et pourtant c’est une épidémie qui est là. Et aussi c’est une maladie qui est nouvellement arrivée ici au niveau de chez nous ici à Beni, d’abord ça faisait problème parce qu’il y a beaucoup d’illettrés te les gens ont difficile à comprendre une maladie pareille, épidémie quoi tout ça, il faut beaucoup d’explications pour que les gens comprennent. Et de deux, à part le contexte des guerres, des massacres des ADF ici, il y a aussi la misère et puis la zone là où la maladie est apparue, je dirais Beni et ses alentours, ça faisait longtemps que les gens étaient frustrés, je dis toujours dans le contexte de la guerre et aussi nous étions sur le plan politique dans un contexte très compliqué, on nous a privé des élections, on a fait quoi alors tout ça c’est ce qui a créé même la résistance. Pour accueillir tout au début cette histoire d’Ebola, comme ça crée une résistance chez la population sur le plan accueil et quand les gens sont venus, je v eux parler des experts, les experts dans le traitement de cette maladie, ils sont venus comme pour imposer le traitement et on prenait tout le monde comme des néophytes ne sachant rien, c’est comme ça les gens n’ont pas compris alors qu’on avait aussi des experts des médecins et tout ça et pourquoi on n’a pas utilisé nos médecins qui nous soignent à qui nous avons confiance, c’est comme ça qu’il y a eu comme un langage des sourds parce que ceux qui sont venus comme experts, ils parlent une autre langue, je parlais de lingala et toujours en français et vous comprenez que quand vous parlez une autre langue que le malade ne comprend pas, il va vous prendre pour un arrogant ou encore vous avez tendance à dominer sur lui et ainsi de suite or en médecine dès que le malade n’a plus confiance en vous, vous comprenez ce que ça produit comme résultat, nécessairement négatif donc tout au début ça était difficile comme ça parce que la méthode a été mauvaise. On est venu comme s’imposer, on vous importe des traitants, je parle des médecins de toute sorte ce qui n’est pas mal mais je crois qu’ils avaient mal procédé donc c’est comme ça que je dis le début a été comme la fin. Mais nous les grandes personnalités ont a compris cela et après avoir accueilli ont compris après surtout quand on voyait déjà des morts, on a compris et dès que les gens ont compris que nous avons compris, on a adopté tout ce qu’on nous a donné comme règle à suivre en ayant compris et adopté voici comment on nous pris comme leaders pour véhiculer les messages et je vous assure que ça n’a pas été facile, ça était très compliqué, on nous caillassent, on a dit tout ce qu’on pouvait dire, on a même échappé à des attentes pour cette riposte mais Dieu est dans nous en sommes sortir et les gens ont fini par comprendre

**P 4: IA_01_COMM_LR.rtf - 4:2 [Répondant : au niveau de la zo..] (2:2) (Super)**

Codes: [Répondant_Responsabilité_habituelles]

No memos

Répondant : au niveau de la zone de santé de Beni, je suis membre du Coges mais aussi je fais partir de l’équipe du CLC où je suis trésorier et je représente aussi l’église catholique, je participe aussi dans plusieurs réunions et même on peut m’appeler aussi pour d’autres questions sanitaires et là on peut intervenir pour faire comprendre une question au niveau des fidèles ou de la population pour faire circuler facilement les messages.

**P 4: IA_01_COMM_LR.rtf - 4:3 [Répondant : je jouais le rôle ..] (6:6) (Super)**

Codes: [Répondant_Responsabilité_pdt MVE]

No memos

Répondant : je jouais le rôle non seulement comme le leader local, j’étais aussi dans l’équipe d’intervention rapide donc au cas où il y a une résistance quelque part comme prêtre on pouvait m’envoyer pour être comme facilitateur pour faire passer le message afin de temporaliser et facilement on croit aux prêtres et les équipes de la ripostes pouvaient facilement faire leur travail donc j’étais comme un pont entre les équipes de la ripostes et la communauté et aussi comme des communicateurs aussi et même sensibilisateur, on encadrait et on rassemblait les gens facilement et le message passait pratiquement dans le milieu dans lequel nous sommes à Malepe. Ça pourtant se vanter, j’ai eu quelques poches de résistances qu’on a su démanteler ça par notre intermédiaire

**P 4: IA_01_COMM_LR.rtf - 4:4 [Répondant : dans le langage vu..] (8:8) (Super)**

Codes: [Considération_Ebola_population]

No memos

Répondant : dans le langage vulgaire des gens, les gens parlaient d’une maladie qu’on a imposée aux gens pour venir exterminer la population ici. Je vous ai dit qu’il faut lier les actions au contexte des ADF aussi. De deux, ils ont pris ça comme une épidémie là où on vient gagner de l’argent, c’est comme ça qu’on parlait de business et on dit qu’on mettait plus l’argent en avant que le traitement des gens et dès que les gens ont découvert ça, ils ont dit que vous voulez gagner de l’argent sur notre sang ou bien sur nos corps. A quoi ça sert ?

**P 4: IA_01_COMM_LR.rtf - 4:5 [Répondant : surtout ça quand i..] (10:10) (Super)**

Codes: [Agression-Menaces_Structure-Prestataire_raisons]

No memos

Répondant : surtout ça quand ils ont eu l’hypothèse qu’on vient nous exterminer par cette maladie, c’est comme ça qu’il y a eu quelques bagarres dans les quartiers donc ils ont pris ça comme une menace malheureusement ce n’était pas ça, on a brulé donc c’était compliqué

**P 4: IA_01_COMM_LR.rtf - 4:6 [Répondant : sur le plan accuei..] (12:12) (Super)**

Codes: [Qualité_soins_Prestataires] [Structure-Service_Fermeture_pdt MVE]

No memos

Répondant : sur le plan accueil, je crois que chacun s’efforçait de bien accueillir les patients qui pouvaient arriver tout en gardant les principes des structures où tout le monde doit être bien accueilli. Et de deux, quand il y a eu cette gratuité des soins, ça déséquilibrait certaines structures privées et ça aussi un autre déséquilibre au niveau de l’accueil dans les structures publiques donc il y a eu un afflux des malades donc vous comprenez, il y avait des malades qui n’étaient pas vite traités à cause de cet afflux mais je crois que chacun s’efforçait d’être à la hauteur des attentes des patients.

**P 4: IA_01_COMM_LR.rtf - 4:7 [Répondant : la plainte qui éta..] (14:14) (Super)**

Codes: [Reproches_aux prestataire_population]

No memos

Répondant : la plainte qui était là est que les résultats ne sortent pas tôt et quand on t’amène à l’hôpital tu dois attendre c’est comme si tu étais abandonné jusqu’ à ce que quelqu’un pouvait même mourir et comme les gens avaient en tête qu’une fois vous allez au CT ou au CTE, c’est pour aller mourir, je sais que certains mourraient de la psychose et d’un niveau élevé de s stress. Psychologiquement ; il est déjà abattu quand il arrive et alors dans ce contexte là je peux dire qu’il en a ceux qui rentraient et ça créent une résistance et aussi comme c’était brusque sans qu’il ait une préparation parce que c’est une pandémie qui arrive et on ne peut pas faire une préparation. Ce que je peux relever sur le plan laboratoire parce qu’on a directement dit qu’on ne peut pas enterrer un cadavre sans que le corps soit examiné dans le laboratoire et cela aussi a créé une résistance et puis comme impatience aussi et nous sommes dans un milieu où on respecte les morts, dès qu’on vous dit attende encore jusqu’au soir c’est ce qui créé effectivement des problèmes. On ne comprenait pas qu’est-ce que ces gens font avec les cadavres alors ça crée des soupçons inutiles et quand vous déclarez que le corps est positif quand les gens sont montés avec l’émotion du décès et encore on vient ajouter tout ce qui est racontars et à la fin on vous dit qu’on ne vous remet plus le corps que vous attendez et ceux nous qui enterrons le corps, avec tout ce qu’il y a comme soupçons, c’est comme ça que ça crée toujours des problèmes. Mais bon qu’est-ce que vous vous voulez qu’on fasse, jusqu’aujourd’hui quand j’essaie de faire le monitoring, plusieurs savent qu’Ebola n’existe pas, c’est une histoire montée de toute pièce et pourtant ça existe.

**P 4: IA_01_COMM_LR.rtf - 4:8 [Répondant : ces triages nous o..] (16:16) (Super)**

Codes: [Triage_Considération_population]

No memos

Répondant : ces triages nous ont aidés à décanter certaines situations et j’ai encouragé ça parce que c’est qui nous a aidé davantage encore à permettre aux gens de comprendre pendant qu’on doit prélever la température, passe par ici et tout ça et ça permit de temporaliser les malades dans les centres et effectivement nous avons encouragé ceux qui se sont donnés pour tout ça, tous les partenaires bien sûr. C’est tout à fait normal, c’est ce que j’ai retenu comme point positif et malgré quelques points négatifs qu’on a relevé, des positifs, il y en a. ce que je trouve comme positif c’est ce cette mobilisation des partenaires pour ne fur-ce que sauver la vie et de deux, les gens ont donné l’argent, chacun selon sa capacité, ,il y a eu l’engagement de la mobilisation du monde entier, je peux parler ainsi et chacun a donné le mieux et je comprends qu’il y a eu beaucoup des sacrifices malgré la résistance mais les gens sont allés jusqu’au bout, c’est comme ça qu’en mettant du triage, chacun, selon les partenaires en arrivant, il se pointe et dit moi je viens de tel part pour que tout le monde soit servi

**P 4: IA_01_COMM_LR.rtf - 4:9 [Répondant : c’est ça le problè..] (18:18) (Super)**

Codes: [Ressources_humaines_Disponibilité et perturbations]

No memos

Répondant : c’est ça le problème, je le dis tout au début. Je dis avec Ebola, comme il y avait un grand financement, ça emportait beaucoup des médecins, des infirmiers et tant d’autres jusqu’à ce que certaines structures sanitaires se sont retrouvées déséquilibrer ou bien même presque abandonner par certains médecins, infirmiers et certains malades aussi se retrouvent non encadrer encore et vous savez que chaque malade a toujours un médecin et un infirmier à qui il a confiance et dès que le médecin s’en va dans la riposte, c’est fini, il va prendre d’autres engagements, il crée un vide. Alors c’est comme ça qu’on appelle ça Ebola business parce qu’on cherchait plus là où il y a l’argent, bon c’est tout à fait normal avec la crise économiques que nous avons et chacun cherche là où il est mieux payé. Le cout de la vie ça amenait chacun là où il est allé parce que certains ont été viré carrément dès là om ils travaillaient et ils pouvaient aller travailler dans la riposte et on ne peut pas être au four et au moulin à la fois donc pratiquement ça créée des équilibres

**P 4: IA_01_COMM_LR.rtf - 4:10 [Répondant : oui, dans la commu..] (20:20) (Super)**

Codes: [Gratuité_Rumeurs]

No memos

Répondant : oui, dans la communauté, il y avait des rumeurs que ces médicaments sont soit périmés ou soit tout ça et tout au début déjà on refusait ça et après beaucoup d’explications, je sais qu’ils ont compris et surtout je vous ai dit que cette épidémie nous a trouvé dans un contexte très compliqué des tueries et dès qu’on vous amène encore les médicaments aujourd’hui, oh c’est pour vous tuer davantage mais les gens ont accepté après pour prendre ces médicaments mais au début, ce n’était pas facile mais ça aide finalement parce que quand vous arrivez à l’hôpital et on vous donne les mêmes médicaments-là, les produits qui doivent être gratuits surtout que nous sommes dans une ville paludique et c’était parmi les produits qu’on a octroyé gratuitement et quand vous quittez on vous dit parte car vous n’avez pas des factures à payer. C’est à partir de là que les gens ont compris qu’on est venu nous aidé donc c’est à partir de la facture que les gens ont compris qu’il y a la gratuité des soins et cela a permis à certains guéris de véhicules les messages qu’il faut accepter cela parce que nous ne payons plus les soins de santé et il n’y a pas des problèmes

**P 4: IA_01_COMM_LR.rtf - 4:11 [Répondant : je vous assure que..] (22:22) (Super)**

Codes: [Prestataire_Attitude_devant_malade]

No memos

Répondant : je vous assure que les prestataires n’ont pas eu tâche facile, bien sur certains s’efforçaient mais les malades ont trouvé que les médecins et les infirmiers sont devenus tous très réticents, en tout cas très réticents parce que ça faisait trainer donc cette longue procédure à suivre, il faut prélever la température, la mise en quarantaine, les histoires de triage, ça indignait plusieurs si bien qu’il y a qui rentrait sans être traité. Vous imaginez vous y allez le matin avec tous les signes que vous vous sentez, maux de tête, maux de ventre et ça vous créée déjà la psychose, diarrhée par exemple mais personne ne vous regarde bien sûr à cause de l’afflux ou bien on peut vous caser quelque part et avec tout le risque de vous oublier et vous rentrez affamer et quelqu’un peut piquer une crise ou convulsé et les gens peuvent penser que c’est ça alors que c’est autre chose

**P 4: IA_01_COMM_LR.rtf - 4:12 [Répondant : elles étaient régu..] (24:24) (Super)**

Codes: [Réunion_pdt-épidémie-Ebola]

No memos

Répondant : elles étaient régulières, très régulières et d’ailleurs à la fin de chaque journée, on devait donner le compte rendu de là où on était parti pour permettre demain d’avancer et bien sûr qu’il y avait une résistance quelque part, on nous appelait en urgence pour aller décanter la situation souvent avec le maire de ville ou avec le médecin chef de zone et d’autres équipes de la riposte souvent la coordination ici et on se réunissait plus régulièrement possible

**P 4: IA_01_COMM_LR.rtf - 4:13 [Répondant : Même dans le coges..] (26:26) (Super)**

Codes: [Réunion_pdt-épidémie-Ebola]

No memos

Répondant : Même dans le coges et parfois on travaillait dans la complémentarité parce qu’on est dans la même zone de santé

**P 4: IA_01_COMM_LR.rtf - 4:14 [Répondant : je pense qu’on doi..] (28:29) (Super)**

Codes: [Agression-Menaces_Structure-Prestataire_solution]

No memos

Répondant : je pense qu’on doit commencer par la conscientisation des petits groupes et qu’on y allait d’une façon graduelle. Je vous assure que même parmi les prestataires qu’il y avait des sceptiques et c’est ce qui est à la base même des plusieurs résistances, des plusieurs problèmes. Vous êtes en train de prester mais vous êtes le premier à douter de ce que vous êtes en train de faire et ça crée la contradiction et celui-là à qui vous êtes en train de parler vous croyez qu’il est naïf et c’est lui qui va aussi véhiculer le même message dans la communauté et ça crée des problèmes donc je veux dire pour commencer à créer d’abord la confiance que les prestataires eux-mêmes soient positifs dans leurs langages et que l’argent que les gens ont eu, c’est tout à fait normal que vous soyez payés, ne montrent pas que c’est à cause d’Ebola que vous avez eu tout ça ; tout d’un coup tu as cinq véhicule et tu as une maisons que tu n’avais pas et les gens savent d’om ça vient, ce que je propose que ceux qui ont travaillé dans les postes stratégiques contre Ebola qu’ils soient modestes, qu’ils ne donnent pas raison à ceux qui ont parlé très mal d’eux en disant que vous êtes partis chercher l’argent et voici les résultats. Nous te voyons, tu as fui l’hôpital en nous abandonnant parce que tu es allé chercher l’argent, effectivement les gens montrent ça et après ça vous tirez des ennuis donc c’est comme cela que je dis que les gens ne mettent pas l’argent en avant, mettez d’abord la raisons pour laquelle cous avez étudié pour offrir des soins aux malades que de leurs montrent ce que vous avez eu en travaillant dans la riposte contre Ebola.

Mais j’avais aussi parlé de la logique distributive, l’équilibre, il y avait seulement des grands décalages et cela créait des gros ennuis, ça créait même des soulèvements parce qu’il y a un montant sûr et on a travaillé ensemble mais tel a eu un gros montant mais un autre a un petit montant et cela créée un grand déséquilibré. Et aussi à leur proposait que s’il y a un cas, il faut venir le plus vite possible et regroupe la famille concernée, soyez proche de la famille que de déclarer et de parler d’une façon diplomatique pour éviter qu’il y ait des résistances effectivement parce que dès que la famille comprend le tôt que possible, les familiers vous leur parlez de façon simple, bien, le message va placer mais l’entourage suit la réaction de la famille restreinte d’abord

**P 4: IA_01_COMM_LR.rtf - 4:15 [Répondant : ce que je peux ajo..] (31:31) (Super)**

Codes: [Commentaire-Recommandation_Répondant]

No memos

Répondant : ce que je peux ajouter c’est de dire merci et remercie tout le monde qui s »est donné corps et âme pour que Ebola soit éradiquer malgré qu’il y a eu beaucoup des remous dans la communauté et dire merci avec les dons que chacun a eu. Nous sommes en train d’atterrir, nous remercions tous les partenaires. Nous remercions ce qui ont compris et même ceux qui résistent encore qu’ils comprennent que la maladie reste la maladie et elle finit par être éradiquer avec le concourt de tout le monde

**P 5: IA_01_HGR_DN.rtf - 5:1 [Répondant : ici pour vous répo..] (4:4) (Super)**

Codes: [Considération_Ebola_population]

No memos

Répondant : ici pour vous répondre, je veux donner les points faibles et les performances par rapport à Ebola ; par rapport aux points faibles, il y a d’abord la mort en cascade des beaucoup de cas qui passaient à une épidémie qui semblait d’abord inconnue au début et cette épidémie a été annoncé mais la population n’a pas bien accueilli cette épidémie c’est pourquoi les gens ont pensé à des empoisonnements, à des intoxications, c’est pourquoi ils ont passé d’abord chez les tradipraticiens et dans les dispensaires privées, c’est après avec la sensibilisation qu’on est parvenu à comprendre que c’est une épidémie étant donné que on a déjà vécu la même épidémie dans d’autres provinces de la RDC. Alors ça n’a pas été accepté directement par la population, il y a eu même des menaces de casses des structures, les gens de la riposte ont été menace jusqu’à ce que les gens ont compris tardivement qu’il y avait vraiment mort d’hommes en cascade donc la population n’a pas accepté au début cette épidémie-là, eux ont pensé à une autre chose qu’à une épidémie qui est scientifiquement compris par les autres

**P 5: IA_01_HGR_DN.rtf - 5:2 [Répondant : Oui, nous sommes p..] (2:2) (Super)**

Codes: [Répondant_Responsabilité_pdt MVE]

No memos

Répondant : Oui, nous sommes parmi les responsables de CHR Beni mais nous sommes aussi membre de la commission prise en charge et médicale dans la riposte contre la MVE. L’épidémie a débuté au début de mois d’aout 2018 et jusque maintenant nous sommes toujours dans l’épidémie et on était en train de penser que d’ici le 12 avril on allait clore cette épidémie-là qui devait être proclamé officiellement pour en finir mais subitement lorsqu’on était en un jour de la déclaration de la fin de l’épidémie, on a été encore surpris par un cas qui vient d’être annoncé qui a été découvert à la suite au swab donc c’est un cas d’un décès communautaire qui a eu lieu dans le quartier Botanuka en ville de Beni pour lequel lorsqu’on a fait le Swab trois fois, le résultat se sont révélés positif, c’est ce qui nous fait encore étonné. Voilà un peu docteur la situation actuelle ;

**P 5: IA_01_HGR_DN.rtf - 5:3 [Répondant : donc eux se sont a..] (6:6) (Super)**

Codes: [Agression-Menaces_Structure-Prestataire_raisons]

No memos

Répondant : donc eux se sont attaquent dans le sens de dire que ce n’est pas cette Ebola-là mais aussi ça surpris désagréablement étant donné qu’on attendait dans les vois des ondes que cette épidémie ravage beaucoup des gens maintenant quand il a fallu annoncer que l’épidémie est ici, les gens ont pensé à ce qui se passe ailleurs et eux ont trouvé que tout le monde sera décimer par cette épidémie, c’est pour cela qu’on a trouvé cette nouvelle comme une mauvaise nouvelle qui a été accueilli par cette population

**P 5: IA_01_HGR_DN.rtf - 5:4 [Répondant : oui, cette épidémi..] (8:9) (Super)**

Codes: [Structures-Services_fonctionnement]

No memos

Répondant : oui, cette épidémie a perturbé le fonctionnement des structures étant donné que d’emblée 50% des bâtiments qui servaient de la prise en charge des malades à l’hôpital ont été réquisitionner pour servir des installations par rapport à cette nouvelle épidémie, voyez par exemple nous avons un grand bâtiment de la pédiatrie qui a été ravi immédiatement et qui sert actuellement de laboratoire de l’INRB, c’est grand bâtiment-là est parti, il y a d’autres bâtiments qui servaient de cadre de la clinique de l’hôpital qui actuellement sert de cadre pour la crèche donc ça héberge les enfants séparaient de leurs mères, les cas suspects et c’est là où on garde les enfants jusqu’à ce que la maman sorte di CTE ou du CTI testé négatif ou positif. Tout ce temps-là ce bâtiment est en train de servir ces enfants-là. Il y a aussi un bâtiment qui servait du triage de l’hôpital qui est devenu le triage de l’hôpital de la riposte imputait déjà de l’hôpital donc beaucoup des bâtiments sont déjà partis et la prise en charge devient un problème avec la flambée des cas, on est déjà au-delà de 100%, là où on pouvait héberger ces malades, on a maintenant des difficultés, c’est un impact négatif mais il fallait quand même céder ces bâtiments-là en faveur de cette épidémie.

A part l’espace, nous avons aussi la morgue qui est là dont la capacité elle est petite, beaucoup de cas sont morts même des cas suspects non confirmés mais qui ont trainé longtemps dans les dispensaires, il fallait que tout ce monde soit acheminé à l’hôpital général même les décès communautaires pour qu’il soit remis entre les mains de leurs famille après avoir reçu les soins, ça aussi ça nous a donné un problème de capacité au niveau de la morgue.

**P 5: IA_01_HGR_DN.rtf - 5:5 [Répondant : les activités en q..] (11:11) (Super)**

Codes: [Structures-Services_fonctionnement]

No memos

Répondant : les activités en question continuent à fonctionner puisque ce sont des activités dévolues du paquent de l’hôpital général de référence mais on a travaillé en combinaison avec l’autre service pour essayer de maintenir ces services en faveur de ces malades nécessiteux donc on a essayé de combiner les services ensemble en faveur de ces bâtiments qui ont été réquisitionné mais la capacité a constitué un problème par rapport à ça

**P 5: IA_01_HGR_DN.rtf - 5:6 [Répondant : au début il y avai..] (13:14) (Super)**

Codes: [Structure-Service_Utilisation_population]

No memos

Répondant : au début il y avait un problème parce qu’Ebola se trouvait à l’hôpital maintenant cela a constitué un rejet tout le monde a été différent de venir utiliser les installations de l’hôpital, on disait non, ça c’est devenu hôpital Ebola et il y a eu vraiment une baisse de fréquentation à tous les niveaux, que ça soit en ambulatoire, que ça soit en hospitalisation puisqu’on craignait de la contamination quand on entre dans l’enceinte de l’hôpital étant donné qu’on pensait que tous les cas Ebola étaient hébergés à l’hôpital. C’est maintenant avec le sensibilisation et quand on a éloigné le CTE là où on admettait les cas suspects d’Ebola qui n’étaient pas encore confirmés à 500 mètres de l’hôpital que les gens ont compris que l’hôpital est là pour desservir les malades et Ebola, prise en charge ailleurs, c’est maintenant que la population est venu mais dans les trois premiers mois même les six premiers mois, il y a eu baisse de fréquentation croyant que et les soignants et la structure constitués déjà la prise en charge des cas Ebola, il y avait d’autres malades qui étaient investiguaient qui venaient des structures privées, de chez les tradipraticiens qui venaient en première intention à l’hôpital en terme de référence maintenant qui étaient validés et investiguaient dans l’hôpital ; quand les gens apprends que le malades était aussi à l’hôpital et c’est après qu’on a amené aux CTE, nous on pense qu’Ebola était déjà à l’hôpital et pourtant l’hôpital ne prenait pas en charge les cas suspects et les cas confirmés d’Ebola puisqu’il y avait leur structure mais ça n’a pas permis que cela soit d’emblée accepter par la population

Au début vraiment, c’était une résistance

**P 5: IA_01_HGR_DN.rtf - 5:7 [Répondant : par rapport à la q..] (16:16) (Super)**

Codes: [Qualité_soins_Prestataires]

No memos

Répondant : par rapport à la qualité des soins, il y avait d’abord cette promiscuité des cas où il valait donc délocaliser tous les lits qui étaient dans le bâtiment par exemple la pédiatrie pour ramener de là vers le bâtiment qu’on a érigé pour la pédiatrie mais dont la capacité était petite alors on a entassé les malades, ça alors constitue un obstacle maintenant avec les différents piliers de la riposte, il y avait ce qu’on appelle la commission PCI, prévention et contrôle des infections, là aussi on a donné des normes disant que les lits doivent être espacé d’au moins un mètre, là aussi il fallait désengorger les espaces pour mettre d’autres lits dehors enfin de faire en sorte que la contamination soit diminuée, ça aussi ça constitué un élément de problème par rapport à la qualité des soins mais pour ce qui est des soins d’une manière ordinaire il fallait respecter les ordinogrammes de prises en charge et le peu des malades qui venaient guérissent et c’est à la suite de ces guérisons-là que les gens ont compris que même si l’hôpital est là croyant qu’il y a l’épidémie les gens sont en train de guérir et ils ont continué à venir, c’est pourquoi vous voyez dehors il y a des tentes qui sont érigés là-bas pour essayer des désengorger mais en répondant à cette capacité, il fallait mettre des tentes additionnelles dehors pour recevoir d’autres cas qui étaient référés et qui demandaient des soins en dehors de l’hôpital

**P 5: IA_01_HGR_DN.rtf - 5:8 [Répondant : pour ce qui est de..] (18:18) (Super)**

Codes: [Triage_Considération_population]

No memos

Répondant : pour ce qui est de triage et comme on a décidé de décrire les zones donc d’emblée là au début il fallait décrire les zones de lanière à ce que tout maladie qui arrive soit testé en termes de thermo flash en termes des températures, il y avait aussi une dispositif de lavage des mains alors il fallait que les malades passent par un triage avec des formulaires pour voir ce que le cas répond à la définition de cas et cela a permis à ce qu’on puisse filtrer les cas pour voir si ça réponds à la définition puisse être orienté à la CTE mais le cas qui ne réponds pas à la définition soit remis à l’hôpital et cela n’a pas posé de problème parce que cela était la première méthodologie pour filtrer le cas de manière à séparer les cas suspects et les cas nos suspects donc le triage n’a pas constitué un problème sauf qu’il y avait certains malades qui pouvaient mentir. Quand certains malades comprennent la définition des cas même dans la communauté, il pouvait mentir au début et maintenant quand il est à l’hospitalisation que vous allez qu’il est dans la définition des acas que vous allez le retirer de là pour l’envoyer dans la CTE

**P 5: IA_01_HGR_DN.rtf - 5:9 [Répondant : il mentait parce q..] (20:20) (Super)**

Codes: [Triage_comportement_patient]

No memos

Répondant : il mentait parce qu’il y a l’isolement là-bas et qu’il croyait que le centre d’isolement est un mouroir quand quelqu’un entre là-bas peut-être il ne va plus survivre, il sera contaminé et il va mourir là-bas.

**P 5: IA_01_HGR_DN.rtf - 5:10 [Répondant : de la même manière..] (22:22) (Super)**

Codes: [Considération_prestataire_EPI_population]

No memos

Répondant : de la même manière qu’il y a eu des équipes de surveillance et des équipes de sensibilisation dans les hôpitaux, il y a eu aussi des équipes de sensibilisateurs et de communicateurs dans la communauté et on leur avait dit comment ils peuvent comprendre un peu la donne afin de comprendre qu’on ne peut pas se toucher main en main, corps à corps puisqu’il fallait croire que tout le monde est infecté. C’était d’abord ça la définition donc apparemment il faut considérer que toute personne est contaminée même si apparemment il est en bonne santé par conséquent il fallait limiter un certain nombre des gestes pour ne pas soit contaminer les malades soit être contaminé par les malades et tous les malades avaient compris et cela n’avait pas constitué un obstacle. Il a fallu que les gens comprennent que même le soignant peut me contaminer et aussi le soignant sache que le malade peut aussi le contaminer et cela n’a pas constitué un blocage pour nous à l’hôpital puisque le message est passé dans les hôpitaux et dans la communauté

**P 5: IA_01_HGR_DN.rtf - 5:11 [Répondant : oui, nous avons co..] (24:24) (Super)**

Codes: [Ressources_humaines_Disponibilité et perturbations]

No memos

Répondant : oui, nous avons connu des problèmes avec le personnel parce qu’il y a eu une instruction de la part du ministère qui a demandé à ce que les premiers auprès de cas suspects, des cas MVE positif soient les agents du ministère, c’est pourquoi l’hôpital a été amputé même à 50% de son personnel donc HGR Beni avait perdu 50% de son personnel qui est servir au niveau de centre de traitement, au niveau de centre de transit et même dans l’autre commission par exemple la vaccination alors face à ça nous nous sommes retrouvés diminuer de notre effectifs et cela nous a poussé à faire de réquisition parce qu’il y avait des médecins, des infirmiers, d’autre catégorie de personnel qui était dans la ville mais qui était sous équipé mais nous avons fait des réquisition, nous les avons fait un appel d’offre et on les a soumis à des test de concourt et à partir de ça nous avons amené d’autres qui étaient en ville qui sont en train de nous aider en attendant pour leurs dire que nous n’avons pas signé avec vous des contrats, nous attendons que les gens du ministère retournés de là où ils sont partis vers l’hôpital et en ce moment-là vous pouvez vous attendre aussi au retour c’est comme ça que vous avons essayé de résoudre ce problème de personnel, au moins à 50% notre personnel est parti

**P 5: IA_01_HGR_DN.rtf - 5:12 [Répondant : oui, avec l’aval d..] (26:26) (Super)**

Codes: [Ressources_humaines_Disponibilité et perturbations]

No memos

Répondant : oui, avec l’aval de la DPS Goma, Goma était informé voici la note circulaire du ministre de la santé national qui demande à ce que le premiers animateurs soient d’abord les agents du ministère maintenant au lieu de nous amputer de ceci voici notre proposition, ils ont donné leur aval pour qu’on puisse détacher ces gens-là de l’hôpital vers les différentes commission ;

**P 5: IA_01_HGR_DN.rtf - 5:13 [Répondant : bon chez nous ici ..] (28:28) (Super)**

Codes: [Ressources_humaines_situation_grève]

No memos

Répondant : bon chez nous ici à l’hôpital, on n’était pas encore en grève. C’était plus la grève médicale, les autres catégories des soignants, ils étaient toujours à l’hôpital, c’est pourquoi on avait répondu à ce problème. Peut-être c’était dans une autre zone de santé mais localement on avait répondu à ce problème.

**P 5: IA_01_HGR_DN.rtf - 5:14 [Répondant : dans notre provinc..] (30:30) (Super)**

Codes: [Ressources_humaines_solution-local_Disponibilité et perturbations]

No memos

Répondant : dans notre province, nous avons beaucoup d’écoles de santé, il y a des ITM, des ISTM même des universités pour former des médecins et il y a beaucoup qui étaient plus dans les structures privées et lorsqu’il fallait déployer ces gens là-bas, on a fait l’appel d’offre et tout le monde est arrivé, on n’a pas connu ce problème, quel que soit eu niveau des centres de santé de référence. En tout cas nous avons couvert le gap qui existait et ça n’a pas constitué un problème

**P 5: IA_01_HGR_DN.rtf - 5:15 [Répondant : avant l’épidémie, ..] (32:32) (Super)**

Codes: [Recouvrement_coûts-des-soins_avant gratuité]

No memos

Répondant : avant l’épidémie, il y avait les soins qui étaient payés par les malades mais cette épidémie malheureusement a coïncidé avec les massacres, les tueries, cette épidémie a coïncidé avec la période de la guerre qui est encore même présent actuellement alors avec ça on a trouvé que les gens n’avaient plus accès à leurs champs parce que la plupart de gens ici vivent de leurs champs, il se nourrissent des produits de champs et ils peuvent aussi vendre les produits de champs pour se procurer de quelques choses. Alors le ministère avec PDSS a initié le système de la gratuité des soins dans les formations sanitaires étant donné les deux crimes-là et la guerre et l’épidémie pourquoi ? Parce que tout le monde était déplacé et même s’il y avait des malades au centre de santé, il y avait des insolvables qui pouvaient rester sur les lits sans pourtant payer parce qu’ils n’avaient pas d’argent, c’est pourquoi PDSS est arrivé pour prendre les malades en charge sur le plan de la gratuité et cela nous a permis de désengorger et qui a permis qu’il puisse avoir des achats de services et cela a motivé le personnel et acheter les médicaments et faire fonctionner l’hôpital

**P 5: IA_01_HGR_DN.rtf - 5:16 [Répondant : oui, ils étaient t..] (34:34) (Super)**

Codes: [Gratuité_Accueil_population]

No memos

Répondant : oui, ils étaient très content parce que quand quelqu’un met au monde par exemple par césarienne et on lui demande de retourner chez elle et cela n’était pas facile, il a fallu glorifier pour dire que celui qui a initié ça, il est un salut pour nous permettre de rentrer chez nous à la maison alors qu’on n’avait rien qu’on ne pouvait pas payer les soins

**P 5: IA_01_HGR_DN.rtf - 5:17 [Répondant : non, non, ça était..] (36:36) (Super)**

Codes: [Gratuité_Rumeurs]

No memos

Répondant : non, non, ça était vraiment un salut

**P 5: IA_01_HGR_DN.rtf - 5:18 [Répondant : c’est PDSS pendant..] (38:38) (Super)**

Codes: [Gratuité_Partenaire d'appui]

No memos

Répondant : c’est PDSS pendant toute cette période-là maintenant il a été relayé en mi-novembre 2019 par le partenaire Halima. C’est lui maintenant qui a fait la relevé jusqu’à maintenant. C’était le 16 novembre 2019, celui qui continue maintenant à appuyer la gratuité au niveau de l’hôpital.

**P 5: IA_01_HGR_DN.rtf - 5:19 [Répondant : au début c’était j..] (40:40) (Super)**

Codes: [Gratuité_Evolution]

No memos

Répondant : au début c’était justement total mais PDSS aussi a commencé à se désengager à certains services, il a seulement maintenu la médecine interne et la pédiatrie pourquoi ? Parce que ce sont des services qui répondaient à la définition des cas de MVE, ils ont dit que nous appuyons ce volet parce que ça va permettre la plupart des cas de médecine interne et de pédiatrie viennent se faire consulté dans l’objectif peut-être de trouver des cas suspects qui répondent dans la définition des cas mais les autres catégories de chirurgie et des maternités étaient abandonnés par PDSS. Le total était jusque le mois d’avril, ça commençait en aout 2018. C’est après quoi on a quitté le total pour aller au partiel et considérait les deux services seulement.

**P 5: IA_01_HGR_DN.rtf - 5:20 [Répondant : Alima lui appuie l..] (42:42) (Super)**

Codes: [Gratuité_Partenaire d'appui]

No memos

Répondant : Alima lui appuie la totalité de puis donc le 16 novembre 2019 jusqu’à nos jours

**P 5: IA_01_HGR_DN.rtf - 5:21 [Répondant : oui, oui il y a eu..] (44:44) (Super)**

Codes: [Gratuité_Interruption]

No memos

Répondant : oui, oui il y a eu une longue période de trêve, avant cette période 16 là, une trêve d’environs deux mois comme ça où PDSS s’est désengagé, il y a eu un silence. On n’a pas compris et après il a semblé reprendre et après encore il a terminé pour voir Alima qui vient pour intervenir

**P 5: IA_01_HGR_DN.rtf - 5:22 [Répondant : oui, il y a eu des..] (46:46) (Super)**

Codes: [Gratuité_Interruption]

No memos

Répondant : oui, il y a eu des interruptions donc pour plus comprendre vous allez voir l’administration pour comprendre le moment des trêves au cours de ce processus de gratuité

**P 5: IA_01_HGR_DN.rtf - 5:23 [Répondants : pendant la périod..] (48:48) (Super)**

Codes: [Médicaments_disponibilité_fosa]

No memos

Répondants : pendant la période de PDSS comme lui payait les prestations donc il fallait compter le nombre des fiches et chaque acte était comptabilisé, c’est comme ça qu’il nous payait mais Alima lui, il a un paquet il vient appuyer en globalité, sans compter les fiches, une prime pour la motivation du personnel, une petite prime pour le fonctionnement et puis les médicaments et nous avons trouvé que le taux de satisfaction d’Alima est à 10% donc le taux de satisfaction en termes des médicaments, c’est à 10% dons c’est parmi le point faible, c’est la satisfaction en médicaments son apport est petit, c’est là où il y a le point faible par rapport à Alima

**P 5: IA_01_HGR_DN.rtf - 5:24 [Répondant : le problème de la ..] (50:50) (Super)**

Codes: [Ressources humaines_Motivation_personnel]

No memos

Répondant : le problème de la motivation du personnel était bien avec PDSS mais avec Alima, lui comme il a son plafond bon on a dit l’objectif capital, c’est d’encourager et puis soulager la population. On a dit que s’il fallait évoquer le cas du personnel, ça peut-être aussi mal interprété par cette population, pour lui avait trouvé qu’il a quand même une satisfaction et il y a quand même un partenaire qui vient à son rescousse maintenant si le personnel commence à s’attaquer à ce partenaire ça peut encore constituer un autre problème mais le personnel a dit autant mieux privilégier l’intérêt de la population même si le personnel ne va pas se retrouver, voilà un peu la politique qu’on a prise pour ne pas casser par ici par là.

**P 5: IA_01_HGR_DN.rtf - 5:25 [Répondant : on a signé avec eu..] (52:52) (Super)**

Codes: [Gratuité_Partenaire d'appui]

No memos

Répondant : on a signé avec eux depuis cette période du 15 et un contrat d’une année et nous pensons que jusqu’au 15 novembre, on va arrêter le contrat à moins qu’on puisse encore renouveler donc c’est d’abord un contrat d’une année.

**P 5: IA_01_HGR_DN.rtf - 5:26 [Répondant : le système continu..] (54:54) (Super)**

Codes: [SNIS_fonctionnement_Decrire_pdt MVE]

No memos

Répondant : le système continue et nous entant que responsable cherché d’encoder les données. Nous n’avons pas voulu d’aller dans la riposte contre Ebola, il a fallu maintenir le système de manière que les autorités soient toujours informées de la situation épidémiologique, c’est pourquoi la plupart des responsables de l’hôpital ne se sont pas déplacés, on a plus envoyé les autres dans la riposte de manière à faire la continuité des activités, c’est pourquoi il y a des données à récolte journalière quand il y a une épidémie déjà qui est sortie, il faut informer directement, il y a aussi des déclarations hebdomadaires, les données là des MAPEPI que nous produisons chaque semaine que nous devons balancer et il y a aussi maintenant les données du mois par rapport au canevas qui a été initié par le ministère à travers la DPS jusqu’au niveau des zones de santé et nous les tenons régulièrement donc toutes les fois qu’il y a une épidémie, il faut informer et nous sommes aussi membre de la BCZ et nous avons aussi d’autres situations donc ce système a continu

**P 5: IA_01_HGR_DN.rtf - 5:27 [Répondant : il y a eu quelques..] (56:56) (Super)**

Codes: [SNIS_fonctionnement_Decrire_pdt MVE]

No memos

Répondant : il y a eu quelques problèmes parce que la plupart de ceux qui étaient les chefs de services, ils ont aussi été réquisitionné pour aller là-bas. Il fallait maintenant mettre en place leurs remplaçants, d’autres leurs anciens collaborateurs qui devaient assurer la responsabilité de leurs services maintenant parmi eux il y avait des petites difficultés de remplissage maintenant lorsque ces gens sont partis, il a fallu ressembler ces gens-là, redéfinir le canevas et le montrer voici comment remplir tel rubrique, tel rubrique de manière à ce que les erreurs-là puissent finir et depuis tout ce temps-là, ça juste était deux mois d’apprentissage et ceux-là qui sont restés, ils ont compris la donne et le système a évolué donc il a fallu être à côté d’eux pour leur briefer le canevas de récolte et leur dire comment nous allons travailler en terme de remonter des alertes ou bien d’autres épidémies

**P 5: IA_01_HGR_DN.rtf - 5:28 [Répondant : d’abord, ils sont ..] (58:58) (Super)**

Codes: [Ressources humaines_Motivation_personnel]

No memos

Répondant : d’abord, ils sont restés pour dire plus on va là-bas, on peut s’exposer à l’épidémie, il y avait d’abord l’autre qui avait crainte d’aller à coté de ce cas là-bas, il y a même ceux qui étaient désignés mais ils disaient moi à ma place je voudrais mettre un tel qui peut aller là-bas et même s’il y a une motivation qui est supérieure, je préfère rester ici. D’abord ça était un sentiment pout quelques-uns, tout le monde n’a pas adhéré immédiatement à cause de l’argent, il y avait aussi la crainte de la mort de peur qu’on soit contaminé. Ça c’était un élément, l’autre élément, il ne fallait pas quand même laisser l’hôpital parce que l’épidémie est passagère et les autres vont revenir et les gens ont compris et on a évolué

**P 5: IA_01_HGR_DN.rtf - 5:29 [Répondant : en tout cas comme ..] (60:60) (Super)**

Codes: [Réunion_pdt-épidémie-Ebola]

No memos

Répondant : en tout cas comme je suis en train de vous dire tous les responsable membres du Codir d’abord sont restés et ne sont pas partis dans la riposte même si on pouvait constituer des membres de la sous-commission prise en charge, on faisait les réunions de la sous-commission localement dans l’hôpital et ça ne nous a pas empêché de continuer avec le même rythme donc le codir qui se tient chaque semaine avec des comptes rendu en appui, des réunions de staff médical qui se tenaient ; des réunions inter services qui se tiennent avec le compte rendu en appui et la DPS continue toujours à venir superviser et ils trouvent les éléments dont ils ont besoin ; nous on a pensé qu’on n’a pas délaisser l’aspect du ministère pour se focaliser plus à la riposte.

**P 5: IA_01_HGR_DN.rtf - 5:30 [Répondant : non, en tout cas j..] (62:62) (Super)**

Codes: [Gouvernance_soins santé]

No memos

Répondant : non, en tout cas je peux le dire qu’eux aussi étaient stratège puisque je me souviens au début le chef de division est arrivé, il fallait qu’il accompagne le ministère et les autres membres de l’OMS et tous les partenaires mais après un certain temps, il est encore rentré à Goma pour aller continué son travail régalien en rapport avec ces activités et toute l’équipe cadre de la province est resté là-bas et toutefois qu’il y avait supervision, il descendait en supervision en termes des piliers ou des commissions et on le recevait toujours localement puisqu’il y a des médecins encadreurs des zones de santé, il revenait toujours et même d’autres techniciens et d’autres coordinations

**P 5: IA_01_HGR_DN.rtf - 5:31 [Répondant : en tout cas comme ..] (64:64) (Super)**

Codes: [PCI_Comment_maintenir]

No memos

Répondant : en tout cas comme vous le dites, d’ailleurs lorsqu’on a eu à riposter positivement sur cette épidémie, ça maintenant réveille les esprits des gens parce que les gens pensent que le curatif peut être un élément clés pour les soins des malades mais avec cette riposte, on a compris l’importance de la santé publique pour dire que les soins ne s’attèlent pas seulement au curatif mais c’est la prévention, la PCI qui est vraiment une armée très efficace pour essayer d’endiguer ou de terminer les épidémie puisque ni t’était la PCI, la prévention et contrôle des infections et la vaccination, on n’allait pas s’en sortir, le curatif seul n’allait rien faire maintenant on a capitalisé et on a compris la place d’un agent de l’hygiène publique, la santé publique qui n’est pas seulement le curatifs mais les autres volets aussi de soins de santé, c’est pourquoi tout le monde même actuellement vous pouvez arriver à l’hôpital vous allez trouver quelqu’un qui est toujours en protection, n’est plus touché un malade quand il ne comprends plus les notions de PCI même les partenaires de la PCI sont en train de former régulièrement toutes les équipes de manière à maintenir et à pérenniser les actions de la PCI pour dire que la PCI n’est pas seulement Ebola mais toutes les malades infectieuse et contagieuses, alors ce qui peut nous être utile c’est comment nous approvisionner en équipements de protection, c’est là où il y a encore le grand problème puisque la plupart de ces équipements venaient de pays étranger et on ne sait pas comment on va survivre même pendant qu’on eut encore avoir le bon sens de la PCI, l’équipement de protection

**P 5: IA_01_HGR_DN.rtf - 5:32 [Répondant : c’est qui a changé..] (66:66) (Super)**

Codes: [Changements_depuis_Ebola]

No memos

Répondant : c’est qui a changé tel que je dis, c’est la PCI donc quelqu’un ne peut plus manger sans s’être lavé les mains, quelqu’un ne peut plus soigner les malades sans être lavé les mains, sans avoir porté les gants, sans avoir porté les éléments de protection donc chaque malade peut être contagieux pour les autres malades de la même manière, un malade ne peut pas vous voir travailler sur lui sans un équipement et nous avons compris le volet PCI, c’est ce qui est l’élément clés dans tout c’est la PCI

**P 5: IA_01_HGR_DN.rtf - 5:33 [Répondant : les liens sont plu..] (68:68) (Super)**

Codes: [Changements_depuis_Ebola]

No memos

Répondant : les liens sont plus forts dans quel sens, par exemple ici à l’hôpital vous voyez même avec le système de santé actuel, il est demandé à ce qu’il y ait ce qu’on appelle accessibilité géographique que le malade soit soigner à proximité de son domicile mais les malades sont en train de quitter les lieux le plus loin pour venir à l’hôpital, on se demande, il n’y a même une note circulaire qui a été initié disant que ne peut venir à l’hôpital, celui qui a la note de référence de l’IT pour éviter un peu l’engouement et ça n’a pas suffi même et la capacité est toujours au-delà de 100%, ils sont en train d’envoyer toujours les malades à l’hôpital et ou un malade qui menace en disant que je ne veux pas me faire soigner ici envoyé moi seulement à l’hôpital, voilà un peu ce que je peux dire donc il n’y a pas des tractations entre les malades et les soignants, on est bien et la déontologie, on est aussi de la mettre en pratique et on essayait d’encadrer le personnel pour redorer l’image de l’hôpital,

**P 5: IA_01_HGR_DN.rtf - 5:34 [Répondant : donc ce qui est là..] (70:70) (Super)**

Codes: [Souhait_Changement_gestion_futures_épidémies]

No memos

Répondant : donc ce qui est là d’abord, cette épidémie a fait de tout le monde comme des experts, tous les prestataires au niveau de la zone de santé sont devenus des experts pour dire que pour quelqu’un par rapport à sa commission là où il a évolué pendant ces deux ans de la riposte, il peut aussi aller coacher et organiser les autres qui n’ont pas encore des connaissances mais ce qui est là il faut doter les gens de matériels nécessaires et de réanimation puisqu’à chaque épidémie correspond des mesures de riposte. Par exemple pour ce qui est de cette maladie ici la corona, vous allez voir que les soins intensifs sont aussi importants par rapport à la prise en charge donc si on n’a pas des équipements de réanimation, les appareils en oxygène, le courant continu, ça sera difficile de prendre en charge les cas donc ce qui reste, c’est seulement doté les gens de moyen et des équipements nécessaires pour que même les autres épidémies soient juguler de la même manière qu’on a jugulé Ebola

**P 5: IA_01_HGR_DN.rtf - 5:35 [Répondant : je suis content de..] (72:72) (Super)**

Codes: [Commentaire-Recommandation_Répondant]

No memos

Répondant : je suis content de votre présence, ce que je vous dis ça vient de la réalité que nous avons vécue. Ce n’est pas que je suis en train de vous mentir ou de faire quoi mais nous avons compris avec le temps que nous avons fait que ça c’est la réalité des faits que nous avons vécue et nous sommes content de votre présence.

**P 6: IA_01_PROGRAM_MCP.rtf - 6:1 [Répondant : ici, nous sommes a..] (3:3) (Super)**

Codes: [Répondant_Responsabilité_habituelles]

No memos

Répondant : ici, nous sommes au bureau de la sous-coordination du PNLS, c’est le bureau qui accompagne les zones de santé, les associations et les partenaires en matière de lutte contre le VIH/Sida. Le bureau ici à Béni s’occupe de 17 zones de santé à partir de Kanyabayonga donc la zone de santé de Kayina sur la nationale n°2 jusqu’à Elengeti dans la zone de santé de Uwitsha sur la nationale n°4 à peu près 4 millions d’habitants

**P 6: IA_01_PROGRAM_MCP.rtf - 6:2 [Répondant : moi, dans ce burea..] (5:5) (Super)**

Codes: [Répondant_Responsabilité_habituelles]

No memos

Répondant : moi, dans ce bureau, je suis le coordonnateur et j’encadre une équipe de 5 agents, il y a le coordonnateur, il y a l’assistant technique, un assistant administratif, il y a un chargé de suivi et une sentinelle.

**P 6: IA_01_PROGRAM_MCP.rtf - 6:3 [Répondant : oui, nous avions é..] (7:7) (Super)**

Codes: [Début-Progression_Epidémie MVE]

No memos

Répondant : oui, nous avions été alerté par les amis de Mangina, le médecin traitant du centre de santé de Mangina qui se plaignait d’avoir de cas pour lui d’une pathologie incompréhensible et des épidémies types familiales, càd des épidémies dans une famille, trois ou quatre membres dans une famille qui ont présenté des signes un peu bizarre diarrhée, fièvre et c’est vers la fin qu’il a décrit aussi de rougeole donc des signes de conjonctive avec des selles coloré rouge et avec de problème au point de caqueter donc au point de site d’injection. Directement un de nos confrères, les docteur Jérémie avec le médecin chef de zone se sont déplacés vers Mangina pour aller se rendre compte parce qu’à notre niveau, on avait déjà suspecté Ebola. Alors cette situation est apparue alors que nous avions des cas d’Ebola à l’Equateur et au niveau du PNLS à Beni, nous avions déjà fait une formation des pairs éducateurs à la prison de Kangwayi, alors nous nous avions fait la formation sur le VIH et nos amis de la Monusco qui nous a suggéré d’incorporer Ebola dans les matières à présenter chez les pairs éducateurs compte tenu du fait qu’il y avait Ebola dans le pays. c’est ainsi que nous avons pensé que c’était important mais nous pensions que nous étions vraiment loin par rapport à la géographie et que c’était difficile de penser qu’Ebola pourrait atterrir à Beni donc dans les environs de Beni. Le premier cas qui sont venue de Mangina, c’est parce qu’il y avait des rumeurs faisant état de la consommation d’une viande par les familles qui étaient victime et nous on pense que c’était un paludisme peut-être ou une fièvre typhoïde, c’est alors qu’une équipe est descendu à Mangina était parti avec l’idée d’aller renforcé les amis qui sont à Mangina et peut-être de partager leurs expériences, c’est ainsi qu’au retour de Mangina, ils ont eu des prélèvements qu’on a expédié à l’INRB et il se fait que les résultats de l’INRB à révéler l’existence de Ebola. C’est comme ça que nous on a vécu Ebola ici. Dès que les premiers cas sont arrivés ici, c’est vers le bâtiment, pas le bâtiment bleu, c’était dans le bâtiment en matériaux durable, c’est là justement qu’il y avait notre box de counseling par rapport au VIH avec une équipe de prise en charge de VIH et les premiers cas sont arrivés là-bas et les équipes sont arrivés de Kinshasa lorsqu’on a eu la confirmation qu’il s’agissait bien de Ebola et la première équipe est arrivée et je n’ai pas la date en tête et nous avons participé à la première réunion à l’hôtel Okapi et nous étions appelés pour participer aux premiers investigations qu’on a réalisé effectivement dans le bâtiment et de ses premières investigations, il y a eu une fille qui était guérie mais les autres à peu près 5 comme ça étaient décédés, la seule fille était guérie bien que ça soit un cas positif mais elle était guérie. C’est dans ce contexte que moi j’ai vécu le début de la maladie.

**P 6: IA_01_PROGRAM_MCP.rtf - 6:4 [Répondant : dans la riposte, j..] (9:9) (Super)**

Codes: [Répondant_Responsabilité_pdt MVE]

No memos

Répondant : dans la riposte, j’ai accompagné les équipes d’investigations dans la surveillance, j’ai accompagné les équipes dans la ville de Beni, j’ai accompagné des missions dans la zone de santé de Owitsha, j’ai accompagné les équipes dans la zone de santé de Kalunguta et même jusqu’à Kiondo, en ce moment-là Ebola était du côté Beni et Mabalako. Mais bon, quelque temps après compte tenu de ma charge au niveau de PNLS, j’étais obligé de laisser la riposte pour continuer avec les activités du programme parce qu’effectivement notre hiérarchie nous a interpeller en nous demandant de ne pas baisser les bras par rapport au VIH, c’est ainsi que nous avons laissé les investigations pour rentrer au niveau du VIH pour renforcer les capacités de nos amis au niveau du VIH.

**P 6: IA_01_PROGRAM_MCP.rtf - 6:5 [Répondant : vous savez qu’il y..] (11:11) (Super)**

Codes: [Considération_Ebola_population]

No memos

Répondant : vous savez qu’il y a la rumeur, la rumeur étant donné que nous étions dans un contexte particulier, la rumeur avait pris le dessus, nous étions dans une période des attaques perpétrées par les groupes armés dans toute la partie Est de la ville et même dans la ville et vous voyez dans toute la partie de la RN4, toute la partie Mbawu, toute la partie Owitsha, Erengeti, il y avait des attaques des présumés ADF alors les gens avaient pensé que ces attaques étaient l’œuvre de certains politiciens mal intentionnés ; deuxième élément de contexte, c’est que nous étions en période électorale, on préparait des élections et effectivement les gens ont spéculé sur les implications des politiciens qui voulaient se positionner, c’est ainsi que cette rumeur a créé si vous voulez la résistance de la population qui ne voulait plus participer aux activités de santé qui n’obéissait plus aux instructions même aux recommandations de santé, c’est ainsi que Ebola est apparue dans un contexte vraiment de crise de confiance entre la population et l’Etat

**P 6: IA_01_PROGRAM_MCP.rtf - 6:6 [Répondant : par rapport aux re..] (13:14) (Super)**

Codes: [Ressources_humaines_Disponibilité et perturbations]

No memos

Répondant : par rapport aux ressources humaines, nous n’avions pas les capacités par rapport à la prise en charge même la recherche des cas et même nous n’avions pas un plan de contingence alors c’est ainsi que du côté capacités ça nous a fort manqué et même j’ai observé quand les activités de la riposte ont commencé, il n’y avait presque pas des formations. Je vous ai dit que j’ai participé à des investigations mais c’était juste une formation d’une ou deux heures que nous avions reçu avec les formateurs et après nous sommes passés directement à l’action donc on nous a demandé d’aller partout pour chercher les cas, c’était d’ailleurs une petite équipe qui avait commencé les investigations et puis après lorsque les cas ont commencé à venir ; on a vu qu’il y avait plusieurs commissions qui étaient mise en place et il y avait plusieurs formations qui sont venus après donc déjà nous étions en pleine épidémie en ce temps-là et on a commencé à former les gens pour aller travailler dans ce site, déjà il y avait des équipes de EDS qui étaient mise en place (enterrement digne et sécurisé), on a formé chez les Croix-Rouge et il y avait des équipes d’investigations sur la surveillance, il y avait des formations sur les points d’entrée mais du côté formations, on a pu récupérer mais c’était en pleine épidémie.

Par rapport à la gestion de ces ressources humaines, il y avait le traitement par rapport aux ressources humaines, il y avait des inégalités, on ne tenait pas compte des diplômes càd on donnait la même chose pratiquement à tous les intervenants pendant les quatre premiers mois jusqu’à ce qu’il y a eu des lamentations du côté des agents qui travaillaient parce qu’ils estimaient qu’ils prenaient quand même plus des risques, effectivement il y a eu plusieurs attaques dans la population chez les résistants mais on a constaté que les gens qui étaient engagés localement étaient les moins payés et ceux qui étaient engagés à Goma avaient un barème supérieur, ceux qui étaient engagés à Kinshasa étaient encore plus haut et les expatriés c’est encore plushaut mais alors cette différence de traitement était mal perçue parce que tout le monde estimait que nous étions tous soumis au même risque et que étant au même risque et par conséquent on devait être rémunérer d’une façon conséquente mais c’est cette différence de traitement qui n’était pas bien perçue et d’ailleurs il semble que moi j’étais déjà parti qu’on avait réajusté par la suite mais je pense que si prochainement, on doit réagir, il faut tenir compte de cet aspect, en fait le facteur humain c’est très important quand il y a une différence, il y a quelqu’un qui reçoit 15$ ou 20$ par jour alors que pour le même travail, un autre reçoit 100 ou 150 $ par jour ou 90$, voyez vous-même la marge est trop grande. Ça aurait été mieux que les gens puissent avoir un niveau acceptable

**P 6: IA_01_PROGRAM_MCP.rtf - 6:7 [Répondant : oui, la mobilisati..] (16:16) (Super)**

Codes: [Ressources_humaines_Disponibilité et perturbations]

No memos

Répondant : oui, la mobilisation dans la riposte avait effectivement pénalisé la routine parce que la plupart compte du fait que c’était quand même incitatif du fait qu’on donnait de l’argent. Je vous disais que pour la plupart c’était des gens qui n’étaient pas mécanisés et d’autres qui étaient mécanisés de non payés, d’autres avec la prime de risque et vous savez vous-même comment se présente le barème salarial et c’est ainsi que les gens ont trouvé que c’était quand même quelque chose et les gens n’hésitent pas d’abandonner leur poste pour aller travailler dans Ebola.

**P 6: IA_01_PROGRAM_MCP.rtf - 6:8 [Répondant : oui, nous avons re..] (18:18) (Super)**

Codes: [Ressources_humaines_Disponibilité et perturbations]

No memos

Répondant : oui, nous avons reçu plusieurs lamentations d’ailleurs les membres du COP sont passés plusieurs fois à la radio et nous aussi nous sommes passés plusieurs fois à la radio pour aller sensibilisé parce que nous avions d’abord parler aux médecins chef des zones, nous avions parlé aux prestataires pour qu’ils puissent un temps pour les PVV mais nous avions senti qu’il y avait des difficultés parce que tout le monde pensaient que c’était une activité de 3 ou 4 mois et que ça allait s’arrêter et qu’il fallait profiter un peu c’est pour ça c’était une période ou il y a la manne et il fallait en profiter et on s’est étonné que ça puisse durer pendant deux années durant mais au tout début-là tout le monde abandonnait le service et beaucoup ont abandonné le service pour aller dans la riposte et nous au niveau du PNLS nous nous travaillons à travers les structures et les prestataires qui sont établis parce que l’intégration obligés et c’est ainsi que c’est sur lesquels que nous comptions beaucoup étaient partis pour la riposte et les postes-là étaient restés presque vide, il y a beaucoup des malades que nous avions perdu, il était obligé d’aller dans une autre structure ou dans une autre zone pour pouvoir continué les soins parce que son prestataire donc son infirmier traitant est absent, il a été réquisitionné, il a été engagé dans la riposte Ebola

**P 6: IA_01_PROGRAM_MCP.rtf - 6:9 [Répondant : donc pour l’engage..] (20:21) (Super)**

Codes: [Ressources_humaines_Disponibilité et perturbations]

No memos

Répondant : donc pour l’engagement c’est ce que je vous disais que l’engagement ne suivait pas les procédures telles que le ministère le recommande, il y avait des offres par ici par-là, toutes les ONG engageaient, elles avaient besoin des gens. Vous allez attendre MSF est en train d’engagé, une autre ONG est entre d’engagé et ALIMA est en train d’engager, Unicef est en train d’engagé donc des offres un peu partout.

Au niveau du Ministère de la santé donc au niveau des équipes de la riposte du ministère de la santé qui se rabattaient sur les zone de santé et avaient demandaient au chargé des ressources humaines de la zone de santé qu’il puisse engager au niveau de la zone alors là aussi je vous ai dit on avait remarqué que ceux qui étaient engagé par les ONG avaient une rémunération supérieure par rapport à ceux qui étaient engagés par le ministère de la santé donc tout le monde voulait avoir les résultats, tout le monde voulait avoir la main d’œuvre et c’est ainsi que nous avons remarqué qu’il y a beaucoup des gens qui sont venus d’autres provinces, des gens qui sont venus du Sud-Kivu et de la province voisine de l’Ituri, les gens qui sont venus de Goma pour intervenir et tous ces gens sont venus soit ils étaient appelés par tel ou ils avaient passés le test bref il y a eu quand même un engouement ;il y a eu un flux important de la main d’œuvre mais dans le traitement de la main d’œuvre, il n’y avait pas d’uniformité.

**P 6: IA_01_PROGRAM_MCP.rtf - 6:10 [Répondant : oui, effectivement..] (23:23) (Super)**

Codes: [Structures-Services_fonctionnement]

No memos

Répondant : oui, effectivement, toutes les structures n’ont pas bien fonctionné, nous avons d’abord les structures privées. Les structures privées n’ont pas bien fonctionnés au tout début ils séquestrent eux-mêmes les malades parce que vous vous imaginez quand on a proposé la gratuité des soins tous les malades partaient là où il y a la gratuité des soins parce qu’il semblait que la gratuité des soins était une stratégie pour détecter les cas Ebola mais alors pendant ce temps-là quand on a mis la gratuité des soins, ces structures privées n’ont pas fonctionné mais avant qu’on puisse mettre la gratuité des soins, ils avaient hyper fonctionné parce que tout le monde fuyait les structures de l’Etat en s’imaginant que cela qu’on va les attraper comme cas Ebola et après quand il y a eu la gratuité, c’était maintenant l’inverse tout le monde voulait aller là où il y a la gratuité alors il y a eu des structures de l’Etat qui avaient réduit leur capacité parce que fonctionnant avec 5 ou 6 infirmiers, ils ont vu peut-être leurs effectifs réduits de moitié donc les infirmiers partaient disant que je n’ai pas encore pris mon congé, demain encore on vient chercher les infirmiers, ils partaient disant que je veux faire un mois, il y a eu des notes comme ça de mise en disponibilité bref il y a eu perturbation si je peux le dire dans le fonctionnement des structures

**P 6: IA_01_PROGRAM_MCP.rtf - 6:11 [Répondant : Bon, disons que pa..] (25:25) (Super)**

Codes: [Structure-Service_Utilisation_population]

No memos

Répondant : Bon, disons que par rapport à Ebola je veux vous répondre comme ceci, il y a eu quand même une résistance, c’est quand même un phénomène très important qui avait influencé beaucoup des choses alors d’où était venu la résistance, la résistance était venue des rumeurs. Cette rumeur était venue de la population, des commerçants et cette rumeur était entretenue par les structures privées voyant partir leurs malades, ils ont entretenu une certaine rumeur dans la population pour que la population puisse fuir les services étatiques en leur disant voilà si vous allez là-bas, on va vous capturez comme Ebola et puis on va vous mettre au CTE. Il en a même ceux qui ont exagéré dans la rumeur en disant que quand on vous amène au CTE on va-vous injecter de l’eau pour que vous puissiez partir, c’est une folle rumeur mais on l’a vécu ici alors cette résistance a fait que les gens qui pouvaient venir au centre de santé étatique ou bien intégré, ils ont préféré aller dans les structures privées, c’est ainsi que l’équipe c’est ainsi que l’équipe stratégique de la riposte était obligée d’intégrer certaines structures privées parce qu’on a remarqué que même les malades Ebola, des vrais cas allaient même dans les structures privées, il y a eu beaucoup de contamination dans les structures privées et il y a également dans la même résistance aussi les gens qui fuyaient les structures de santé pour aller se réfugier dans les églises, il en a ceux qui fuyaient les centres de santé pour aller se réfugier chez les tradipraticiens alors on était obligé d’intégrer tout ce monde-là pour qu’on puisse avoir accès aux malades donc la résistance a joué quand même un grand rôle dans les mouvements des malades dans un sens comme dans l’autre. La résistance est vraiment un élément important. Alors cette résistance est également entretenue par les commerçants, les commerçants qui mettent en doute la bonne foi du personnel soignant, les commerçants qui mettent en doute la bonne volonté, ils disent non les gens ne peuvent pas travailler gratuitement donc ils ont quelques choses, ce sont les commerçants qui entretenaient ça. Alors ces commerçants-là, on ne sait pas ce qu’ils ont gagné en entretenant cette résistance mais il y a des commerçants des médicaments càd les pharmacies qui voyant presque leur ruine avec la gratuité parce que ça n’a jamais eu lieu, c’est aujourd’hui que nous voyons la gratuité même à l’époque om il y avait des interventions, il y avait toujours un ticket modérateur mais là où on vous dit gratuit à 100%, c’est rare en tout cas les commerçants des médicaments surtout les pharmacies entretenaient de faux bruit pour que les gens ne puissent pas accéder gratuitement plutôt que d’aller acheté chez eux donc cette résistance, c’est un phénomène qui a perturbé la lutte

**P 6: IA_01_PROGRAM_MCP.rtf - 6:12 [Répondant : par rapport à l’ac..] (27:27) (Super)**

Codes: [Qualité_soins_Prestataires]

No memos

Répondant : par rapport à l’accueil, il y a eu des problèmes parce que les structures fonctionnent avec un nombre réduit du personnel, c’est ainsi que les gens qui venaient pour les soins devaient faire la queue parce qu’il y avait un grand monde qui attendait les soins, les autres infirmiers étaient affectés dans les commissions entre autre la surveillance, la recherche active , et autres les infirmiers qui étaient dans Ebola, ce qui faisait dans les structures, ceux qui prenaient en charge c’était un nombre réduit, il y a eu des réajustements plus tard mais en nombre réduit on a réajusté progressivement, ceci a influencé la qualité des soins parce que pour accéder à une consultation, il a fallu faire deux ou trois heures d’attente. Et puis là l’intérieur il y avait effectivement des médicaments donc on n’était pas en rupture, en tout cas je ne vois pas une structure qui avait connu des ruptures en médicaments, on était quand même bien couvert mais en un certain moment certains actes commençaient à poser problème, la chirurgie ou certaines maladies chroniques, ça commençaient à poser problème, vous voyaient quand on fait la gratuité, tous ceux qui avaient des problèmes de myomes, de hernies et qui n’avaient pas encore trouvé des moyens pour se faire opéré, c’était une occasion mais par rapport aux malades chroniques, au tout début on acceptait les diabétiques et les autres maladies chroniques, vers la fin on dit qu’on ne sait pas prendre en charge tous les malades chroniques parce que les couts étaient devenus trop importants

**P 6: IA_01_PROGRAM_MCP.rtf - 6:13 [Répondant : je pense que les p..] (29:29) (Super)**

Codes: [Prestataire_Attitude_devant_malade]

No memos

Répondant : je pense que les prestataires par rapport à leur travail, je pense qu’ils n’avaient quand même pas à se plaindre, ils se plaignaient seulement des inégalités de traitement par rapport aux émoluments mais ce qu’on a constaté est que le volume du travail ou la charge était devenue trop grande, quelqu’un qui était habitué à prendre en charge 10 à 15 malades par jours était obligé d’en prendre 50 à 60 malades par jour ; tous les lits étaient occupés, ça c’est pendant la gratuité mais alors les prestataires comme il y a les médicaments, comme il y a les intrants. Je crois qu’on n’a pas à reprocher beaucoup des choses, seulement on a reproché aux tradipraticiens qu’ils ont contaminé beaucoup des personnes, chez eux l’asepsie ils n’en ont pas la formation, c’est ainsi qu’on a constaté beaucoup des cas Ebola à cause de leurs séjours dans une structure de prise en charge tradi-moderne

**P 6: IA_01_PROGRAM_MCP.rtf - 6:14 [Répondant : bon par rapport à ..] (31:31) (Super)**

Codes: [Structures-Services_fonctionnement]

No memos

Répondant : bon par rapport à la lutte contre le VIH, nous avions été affectés, nous avons perdu à peu près 18 PVV et sur les 18, nous avons remarqué que la plupart était contaminé sur le lieu de deuil à cause de la résistance càd quand il y a résistance, on fait croire aux gens qu’il n’en a pas eu Ebola. Et les gens se permettaient même d’enterrer les cas suspect et une PVV qui arrive dans un endroit comme ça se contamine plus facilement et peut même mourir plus facilement, il y a des PVV qui recevaient des effets personnels de leurs proches qui sont décédés parce que la résistance était aussi la désinformation ou l’intoxication donc on déformait les informations, il y a eu beaucoup des PVV qui ont été victime de ça et évidemment ce que je venais de dire qu’il y avait de PVV qui n’avaient pas accès aux soins parce qu’ils tenaient à leur confidentialité alors ils voient que leur infirmier n’est pas là alors ils manquent où ils peuvent s’adressent pour des soins et c’est ainsi qu’on a perdu des PVV à cause de ces désordres-là. Les autres services qui ont eu des problèmes, c’est par exemple le service de chirurgie, le service de chirurgie avait connu beaucoup des problèmes en ce sens qu’une fois qu’on a commencé la gratuité, on a pris tous les cas des interventions spécialisées ou les interventions qui devaient recevoir des spécialistes n’étaient plus possibles parce qu’on faisait seulement des interventions de routine donc il y a beaucoup des cas qui n’avaient plus accès à ces interventions. La CPN donc la maternité était presque débordée alors qu’avec un nombre réduit ce n’était plus vraiment une CPN de qualité donc c’était une CPN vraiment à la sauvette quoi, on ne faisait pas la qualité parce qu’il y avait vraiment un débordement, c’est ainsi qu’il y a des grossesses à gros risque où on vous surprend grossesse à haut risque alors qu’on pouvait prévenir ça à la maternité, il y avait des difficultés pour les prévoir, c’est ce service qui a également souffert ;

**P 6: IA_01_PROGRAM_MCP.rtf - 6:15 [Répondant : oui excusait moi j..] (33:33) (Super)**

Codes: [Service_vaccination_Fonctionnement]

No memos

Répondant : oui excusait moi j’avais presque oublié. La vaccination, elle a également souffert parce qu’on ne pouvait pas pendant la période de Ebola faire une vaccination de routine en tout cas c’était difficile étant donné qu’on avait peur de toucher à tout le monde alors il y a eu un ou deux passages comme ça en tout cas on n’a pas fait la routine comme il fallait, il y a eu beaucoup d’enfants perdus non atteints, il y a eu beaucoup des difficultés pour réaliser des activités préventives et promotionnelles.

**P 6: IA_01_PROGRAM_MCP.rtf - 6:16 [Répondant : pour le suivi et é..] (35:35) (Super)**

Codes: [SNIS_fonctionnement_Decrire_pdt MVE]

No memos

Répondant : pour le suivi et évaluation, nous étions obligés nous-mêmes parfois de descendre dans les zones de santé. Nous avions trouvé qu’il y a des zones qui étaient défaillantes alors que nous avions donné des conseils aux zones de santé d’essayer d’engager des temporaires pour que ce système ne puisse pas souffrir mais on avait tout le temps de retard donc faible promptitude, faible complétude alors que c’était une recommandation qu’on puisse mettre de data manager pour que ce service ne puisse pas souffrir mais en tout cas on a trouvé que les données ne remontent pas, parfois même qu’il y a des zones de santé qui envoyaient des données sans avoir fait correctement la validation parce que nous nous avons trouvé dans les données qu’ils envoyaient qu’il y avait des incohérences qui devait être corriger à la base mais que c’est à notre niveau que nous étions obligés de détecter ça, c’est ainsi qu’on a eu de retard dans la production de nos rapports, effectivement ça c’est un service qui a eu des difficultés

**P 6: IA_01_PROGRAM_MCP.rtf - 6:17 [Répondant : nous on faisait de..] (37:37) (Super)**

Codes: [Réunion_pdt-épidémie-Ebola]

No memos

Répondant : nous on faisait des réunions, nous ne sommes pas nombreux mais c’était écourté parce que c’était tous les lundis qu’on devait faire des réunions nous au niveau du PNLS mais c’était vraiment écourté mais vous savez c’est au niveau des équipes cadre c’est là où ça posait des problèmes parce que les équipes cadre chez nous sont réduites alors pratiquement tous les membres des équipes cadre étaient dans des commissions, il y a l’un qui est dans le PCI, l’autre qui est dans psycho, l’autre est dans la logistique, l’autre dans la coordination. Il y avait beaucoup des commissions. C’est qui fait que les réunions propre de la zone de santé devenaient difficile donc pour rencontrer quelqu’un pour une réunion ou un entretien c’était pratiquement difficile et en même ce qui nous concerne parfois même pour la supervision des activités VIH, nous étions obligés d’avoir une certaine flexibilité càd vous planifiez deux jours et vous êtes obligés de passer un troisième jour supplémentaire parce que le temps que vous devez travailler avec l’infirmier superviseur de la zone, il était dans la riposte c’est ainsi que ça nous a affecté vraiment parce que pour les supervisions nous avons l’habitude d’envoyer l’agenda à l’avance et de discuter la faisabilité avec le prestataire de la zone et c’est ainsi qu’on se fixe un consensus pour trouver une date où on peut commencer l’activité alors c’est ainsi qu’on a eu des difficultés pendant la période Ebola pour fixer des dates mais toutes les supervisions étaient quand même bien tenues. Seulement les descentes dans les structures étaient un peu difficiles parce que vous pouvez arriver à la zone, la zone est disponible mais quand vous voulez descendre dans la structure, la structure n’est pas disponible ; parfois à cause de ses résistants, on était obligé de changer des itinéraires pour éviter d’être agressé même si vous n’êtes pas dans Ebola, on voit un véhicule où il y a un superviseur, on pense qu’il est dans Ebola alors on a eu comme ça des cas où on a été agressé et devait changer des itinéraires.

**P 6: IA_01_PROGRAM_MCP.rtf - 6:18 [Répondant : Oui, oui, la prése..] (39:39) (Super)**

Codes: [Gouvernance_soins santé]

No memos

Répondant : Oui, oui, la présence des acteurs, ce que moi j’ai constaté les acteurs surtout internationaux, je prends nos amis guinéens, africains de l’OMS, c’est une autre culture ils ne connaissent pas très bien le système congolais alors, j’ai remarqué » à plusieurs fois l’alourdissement, il y a eu lourdeur dans les interventions à cause de cette présence, vous savez quoi ? lui il est expatrié il ne peut pas descendre sur terrain s’il n’est pas escorté càd que les conditions dans lesquelles nous pouvons accepter de travailler eux n’acceptent pas et puis c’est une grande responsabilité on vous dit que s’il a des problèmes ici ça risque d’être un incident diplomatique, c’est ainsi que moi j’ai vu leur présence bien sûr, c’est peut-être nécessaire mais moi j’ai vue qu’il y a plusieurs fois ça vraiment alourdi le système et moi je recommanderai d’ailleurs que s’il y a un tel problème qui apparait qu’ils viennent juste en appui pour donner des capacités mais qu’ils laissent des descentes, ils peuvent descendre comme des superviseurs si c’est nécessaire si par exemple, il y a quelque chose qui poserait problème mais dire qu’ils doivent descendre sur le lieu d’intervention dans la famille ou dans le quartier ça posait des problèmes. Imaginez que nous sommes allés à Kangwayi avec un expatrié tchadien ou centrafricain, nous arrivons, ils ne parlent pas swahili et je devais lui interpréter donc tout ce que la population me dit, je suis obligé de le traduire pour lui mettre au parfum ça retardait le travail mais je suis investigateur et il est investigateur, je pose des questions qui me permettaient d’avance dans les investigations mais je dois en même temps faire le travail d’interprète et je ne peux pas redescendre quand il y a un élément que j’ai oublié ou retourné, il faut un escorte et je dois redescendre avec lui. J’ai constaté que cette présence internationale sur le lieu, moi j’ai trouvé que ça alourdi le système donc le souhait est que s’il y a un intervenant, un expatrié, c’est mieux qu’il reste au niveau du bureau pour renforcer les capacités, par exemple s’il y a une difficulté, on peut le consulter pour avoir des éclaircissements sur une situation donnée mais lui il tient à descendre dans le lieu, alors c’était un problème. Eux ils étaient mieux payés pour le même travail, il vous fait travailler durer mais pour le même travail où il était mieux payé. J’avais entendu une discussion, je vous fais part de ça, quelqu’un qui dit aux médecins directeurs, nous sommes venus nous aidés mais moi je dis oui, ils sont venus nous aidés et il y a l’autre qui a rétorqué, est-ce qu’il est venu nous aider gratuitement ? Alors qui aide qui finalement, cette présence n’était pas perçu comme un appui comme tel parce qu’à cause de cette lourdeur.

**P 6: IA_01_PROGRAM_MCP.rtf - 6:20 [Répondant : d’abord par rappor..] (41:41) (Super)**

Codes: [Commentaire-Recommandation_Répondant]

No memos

Répondant : d’abord par rapport à la préparation, je pourrais commencer par-là, je constate que notre système sanitaire a une forte dépendance extérieure, je vois tout le système, il y a une forte dépendance extérieure. Cette dépendance extérieure a tout le sens, sur le plan de connaissance, sur le plan des ressources humaines et sur le plan financier, sur le plan du matériel et des interventions, il y a une forte dépendance extérieure. Là cette épidémie nous a démontré cela, imagine comme une épidémie coronas virus vous pouvez voir un cas à Béni pour avoir la confirmation, les échantillons doivent faire 2000 km, il n’y a même ;pas la possibilité pour nous de dire que l’échantillon peut aller en Ouganda mais avant avec les autres épidémies, la choléra les échantillons pourraient aller en Ouganda autour de 500km, c’est plus proche de chez nous que Kinshasa mais aujourd’hui avec ce qu’on a vécu ici INRB devait installer un laboratoire ici et même dans une recommandation on souhaiterait que ce labo puisse rester ici et si vous voyais le pays vous verrez que c’est les deux extrêmes de notre pays, ça aurait été mieux qu’un tel labo soit installé dans toutes les provinces de notre pays pour que la province puisse avoir la possibilité d’avoir les résultats en un temps record

**P 6: IA_01_PROGRAM_MCP.rtf - 6:21 [Sur le plan d’équipements, je ..] (42:44) (Super)**

Codes: [Commentaire-Recommandation_Répondant]

No memos

Sur le plan d’équipements, je viens de parler du labo mais aussi sur le plan des capacités, il serait mieux que les gens, les médecins et les personnels soient régulièrement formés pour ces éventualités. Vous vous imaginez que le premier cas est apparu, on ne pouvait croire que c’est possible alors que nous sommes à côté de l’Ouganda, il y a eu Ebola à Bundubudjo mais nous on n’était pas formé, le personnel au niveau de la frontière, l’hygiène des frontières ou le personnel au niveau de la frontière n’était pas formé, tous nos points d’entrées n’étaient pas sécurisés alors que ça aurait été souhaitable qu’ils soient sécurisés mais avec Ebola, on avait sécurisé les points d’entrée de Beni, Butembo mais je ne sais pas si les mêmes mécanismes se passaient ailleurs donc tous nos points d’entrée ne sont pas sécurisés. C’est vraiment une recommandation que tous ces points d’entrée soient sécurisés et moi je pense que par rapport à l’aspect financier qu’il est intéressant qu’on puisse penser à tous les personnels, médecins, infirmiers qu’ils ont droit à la vie

J’ai oublié un parenthèse Ebola est apparue dans un contexte où il y avait grève, j’ai oublié et ça me revient comme nous sommes en train de parler de ça et même le personnel, les infirmiers étaient en train de séquestrer les rapports, ils avaient mais ils étaient en train de les séquestrer, les MAPPI alors que la maladie a commencé, on a passé un ou deux mois avant que tout le monde s’inquiète pour qu’on puisse faire une intervention alors moi je me dis que si on peut résoudre cette question, si on peut se pencher sur cette question, la prime ou le paie des infirmiers et médecins, moi je me dis que peut-être qu’on aurait évité Ebola parce qu’ils étaient en grève et c’était le quatrième ou le cinquième mois qu’ils étaient en grève et défendre toutes ces revendications n’avaient pas trouvé des solutions, alors vous comprendrez qu’on peut se dire qu’ils ont l’habitude de revendiquer mais peut-être qu’ils sont plus important, moi je me dis que pour affronter les autres épidémies, il faut tenir compte de tout ce personnel

Alors par rapport aux approvisionnements en médicaments, nous nous pensons que la CDER pour la province a quand même joué un rôle parce que quand nous disons les intrants ou les médicaments, nous n’avons pas connu des problèmes seulement ce que nous avons encore des difficultés pour réglementer la circulation de certains médicaments, il y a beaucoup des médicaments que vous trouvez dans les pharmacies privées et vous ne savez pas si ce sont des médicaments des bonnes qualités mais ça existe bon évidemment quand je parlais médicaments je regardais covid-19, on parlait des chloroquine mais il n’y a pas des chloroquine de ces côtés, j’ai essayé de circuler mais il n’y a pas donc on en a pas dans les zones de santé donc notre système de santé a une faible réactivité

**P 6: IA_01_PROGRAM_MCP.rtf - 6:22 [Alors enfin, je constatais qu’..] (45:45) (Super)**

Codes: [Commentaire-Recommandation_Répondant]

No memos

Alors enfin, je constatais qu’avec Ebola, il y a eu énormément des moyens qui étaient déversés et ces moyens pour le gros était consommé dans les véhicules qui circulent, le land cruiser à location, tout le monde a constaté qu’un jour dans un land cruiser c’est 100$, ils ont accepté de prendre des véhicules à location mais dans nos bureaux comme le nôtre ici, on n’a pas de véhicule, il y a beaucoup des services de l’Etat comme le service de l’hygiène aux frontières donc le PNHF, ils n’ont pas de véhicule même après Ebola, je ne sais pas s’ils vont recevoir quelque chose mais ils n’ont pas de véhicule ça veut dire que pour les intervention non seulement à la frontière à Kasindji mais aussi nous avons la limite avec la province voisine, que ça soit des points d’entrée importants qu’il faut sécuriser, la limite entre la ville et les territoires au niveau de l’aéroport donc ils ne peuvent pas se mouvoir ou ne peuvent pas intervenir rapidement s’il y a par exemple un cas ou s’il faut aller chercher les intrants, des épis ou autres choses en tout cas pour ce service, on n’a pas encore résolu cette question alors que ce sont des services qui entrent dans la sureté de l’Etat, c’est vraiment stratégique, si les gens ne l’ont pas compris c’est vraiment important.

**P 8: IA_02_AS_IT1.rtf - 8:1 [Répondant : Mon travail dans l..] (2:2) (Super)**

Codes: [Répondant_Responsabilité_habituelles]

No memos

Répondant : Mon travail dans la structure, je suis l’infirmier titulaire de l’aire de santé. J’ai comme attribution la planification et le suivi des activités. Mais aussi j’encadre les relais communautaires et les membres de CAC aussi,

**P 8: IA_02_AS_IT1.rtf - 8:2 [Répondant : Oui juste quand no..] (4:4) (Super)**

Codes: [Début-Progression_Epidémie MVE]

No memos

Répondant : Oui juste quand nous avons appris qu’il y a épidémie, c’était à Mangina, on avait un peu peur mais on a commencé à prendre des mesures. Et après la maladie est arrivée dans notre contrée et après nous avons quand même eu le premier cas mais c’est un cas qui est transité par le centre de santé de l’aire de santé et après il est allé au CTE et il est guéri et nous avons aussi connu un deuxième cas mais qui n’est pas passé par ici mais qui a été suspecté ailleurs malheureusement cette personne était décédée. Nous avons essayé de nous efforcer de mettre en place des mesures préventives et nous n’avions plus d’autres.

**P 8: IA_02_AS_IT1.rtf - 8:3 [Répondant : Dans la structure] (6:6) (Super)**

Codes: [Début-Progression_Epidémie MVE]

No memos

Répondant : Dans la structure

**P 8: IA_02_AS_IT1.rtf - 8:4 [Répondant : Oui, il en a eu] (8:8) (Super)**

Codes: [Début-Progression_Epidémie MVE]

No memos

Répondant : Oui, il en a eu

**P 8: IA_02_AS_IT1.rtf - 8:5 [Répondant : Nous avons assumé ..] (10:10) (Super)**

Codes: [Répondant_Responsabilité_pdt MVE]

No memos

Répondant : Nous avons assumé la fonction de coordonnateur de riposte dans l’aire de santé, on essayait e suivre la surveillance et la recherche active, on ne cesse de suivre les activités du triage, il y avait beaucoup d’activités-là donc c’était notre fonction pendant la riposte.

**P 8: IA_02_AS_IT1.rtf - 8:6 [Répondant : Dès le début parce..] (12:12) (Super)**

Codes: [Répondant_Responsabilité_pdt MVE_Depuis quand ?]

No memos

Répondant : Dès le début parce que juste au premier mois, je pense que c’était au mois d’aout, nous avons été formé et on nous a parlé de la maladie et de ce que nous devons faire et nous avons attendu quand les cas ont commencé à venir et vous avons fait ce qu’on devait faire

**P 8: IA_02_AS_IT1.rtf - 8:7 [Répondant : c’est sur parce qu..] (14:14) (Super)**

Codes: [Structures-Services_fonctionnement]

No memos

Répondant : c’est sur parce que c’était un moment, on faisait la surveillance et quand on a un cas, il faut alerter et la communauté n’a pas été d’accord avec ça et la communauté a commencé à nous agresser et quelques fois on n’est vacciné pas les enfants et de fois on fermait la structure pendant une certaine période alors des effets comme ça se sont manifestés donc il y a eu beaucoup des perturbations au niveau du fonctionnement de la structure même de l’aire de santé toute entière.

**P 8: IA_02_AS_IT1.rtf - 8:8 [Répondant : On nous agressait ..] (16:16) (Super)**

Codes: [Agression-Menaces_Structure-Prestataire_raisons]

No memos

Répondant : On nous agressait parce qu’il n’était pas d’accord avec l’existence de la maladie. Je me rappelle lorsque nous étions agressés pour la première fois, c’était un cas qui était venu la nuit et l’infirmier de garde m’a appelé, nous avons un cas suspect et j’ai alerté la base, la sous coordination, ils m’ont dit que nous on ne se déplace pas la nuit mais si vous pouvez garder le malade, on viendra prélever le matin ou le prendre le matin malheureusement c’est un cas et deux heures après il est décédé et comme il était prévu qu’on prélève même chez le cadavre, nous étions été obligé de demander à la famille de laisser le corps dans la structure pour l’avoir fermé dans une chambre mais malheureusement la famille n’était pas d’accord mais dans la suite il y a eu des troubles et la communauté est venue nous agresser, on nous a jeté des pierres donc tout découlait de ce que nous voulons faire comme notre travail

**P 8: IA_02_AS_IT1.rtf - 8:9 [Répondant : Oh, pour eux c’éta..] (18:18) (Super)**

Codes: [Considération_Ebola_population]

No memos

Répondant : Oh, pour eux c’était beaucoup des choses à cela parce que Ebola est arrivé, nous étions dans une période pénible où on devait égorger les gens vers Beni et là les gens disaient beaucoup des choses par rapport à ça, on non c’est le gouvernement qui veut nous exterminer, oh on a vendu notre milieu chez les Rwandais et on a beaucoup commenter, alors la maladie quand elle a commencé malheureusement elle a commencé là où on égorger les gens, c’est comme ça que les gens résistent et comme nous avons résisté aux gens qui nous égorger et maintenant nous avons la maladie pour nous exterminer, c’est pour cela qu’il n’était pas d’accord

**P 8: IA_02_AS_IT1.rtf - 8:10 [Répondant : Oh, oui pour la ri..] (20:22) (Super)**

Codes: [Considération_Riposte_Population]

No memos

Répondant : Oh, oui pour la riposte ils disent que comme ils ont envoyé la maladie au début là nous avions des expatriés et la population prenait ça comme des gens qui faisaient de trafiquer des organes des humains, c’est pour cela qu’ils disent qu’une fois qu’ils attrapent quelqu’un on l’envoie au CTE pour retirer les organes et ils n’étaient pas d’accord

Interviewer : Vous avez parlé des gens qui ne venaient pas, qui vous agressez et par moment vous avez fermé la structure parce que plus par rapport à l’insécurité qu’à Ebola ?

Répondant : Oui, c’est plus par rapport à Ebola parce que pendant cette période, il y a eu des inciviques qui sont entrés dans la brousse et qui ont commencé à chercher les IT, rechercher les seniors ainsi que beaucoup des gens qui travaillent dans la riposte. Moi je fais deux ou trois mois, le tout premier mois, j’étais parti définitivement et après ce mois je pouvais revenir pour jeter un coup d’œil parce qu’on était recherché

**P 8: IA_02_AS_IT1.rtf - 8:11 [Répondant : parce que nous col..] (24:24) (Super)**

Codes: [Agression-Menaces_Structure-Prestataire_raisons]

No memos

Répondant : parce que nous collaborons avec les gens qui nous amener la maladie et après on parlait de maladie fomentée qui n’existait pas. Alors toute personne qui collaborait avec l’équipe parce qu’en ce moment il y avait encore des expatriés et même les locaux qui sont venus d’autres milieux tel que Kinshasa et ailleurs raison pour laquelle ils ont dit que celui qui collabore avec ces gens et aussi parmi les gens qui nous amène la maladie

**P 8: IA_02_AS_IT1.rtf - 8:12 [Répondant : ça était juste au ..] (26:26) (Super)**

Codes: [Structures-Services_fonctionnement]

No memos

Répondant : ça était juste au début Nous avons été briefé qu’il ne fallait pas nous exposer au liquide biologique, c’est comme ça que les actes comme les injections ont été supprimés et les examens de laboratoire.

**P 8: IA_02_AS_IT1.rtf - 8:13 [Répondant : Non depuis le débu..] (28:28) (Super)**

Codes: [Structures-Services_fonctionnement]

No memos

Répondant : Non depuis le début nous étions informés par rapport à ça et c’est ce que nous appliquions jusque maintenant

**P 8: IA_02_AS_IT1.rtf - 8:14 [Répondant : Je veux aborder la..] (32:32) (Super)**

Codes: [Qualité_soins_Prestataires]

No memos

Répondant : Je veux aborder la question dans le sens négatif et dans un autre sens positif. Au début il y a eu un grand problème parce que les infirmiers avaient peur des malades, je me souviens encore que si un malade arrivait on ne devait pas le toucher et on ne devait que le garder sur le lit et là ça n’enchantait pas la population parce qu’elle disait qu’on commence à recevoir à l’extérieur, après quand on a maitrisé la protection individuelle, on n’ a plus eu peur des malades et chaque malade qui arrivait, on pouvait mettre des équipements et recevoir correctement le malade. La qualité maintenant moi je crois qu’elle a été améliorée aujourd’hui et les gens ont compris que ce ne sont pas seulement les injections qui guérissent et là quand on est obligé ou même qu’il faudra donner deux catégories de médicaments et que le malade va guérir, ça nous a donné quand même de l’assurance

**P 8: IA_02_AS_IT1.rtf - 8:15 [Répondant : Oui, ça aussi caus..] (34:34) (Super)**

Codes: [Référence-contre-référence_fonctionnement]

No memos

Répondant : Oui, ça aussi causé des problèmes et puis nous, on referait au CTE et la population ne voulait pas écouter parlé de CTE parce qu’ils pensaient qu’ils voulaient prélever les organes donc c’est un centre pour tuer les gens et prendre leurs organes

**P 8: IA_02_AS_IT1.rtf - 8:16 [Répondant : Oui, au début il y..] (36:36) (Super)**

Codes: [Triage_comportement_patient]

No memos

Répondant : Oui, au début il y avait un sérieux problème avec le trie parce que le grand problème c’était autour de lavage des mains, les gens n’étaient pas d’accord que le clan line, n’était pas les désinfectants et ils disaient que c’est Ebola qu’on est en train de se laver et nous on a présenté le virus et quand les gens se lavent et en rentrant à la maison ils vont présenter la maladie et le deuxième problème, c’était par rapport à l’isolement, les gens n’étaient pas d’accord avec cet isolement et c’était difficile pour un patient d’aller dans l’isolement. Par moment, nous étions obligé de créer un endroit où un malade suspect, on pouvait le mettre là que d’aller dans l’isolement et après ils ont compris qu’on peut être isolé, on peut prélever, on peut faire le test et ça ne pose pas un problème mais ce n’est pas encore le 100%

**P 8: IA_02_AS_IT1.rtf - 8:17 [Répondant : Il se posait la qu..] (38:38) (Super)**

Codes: [Considération_prestataire_EPI_population]

No memos

Répondant : Il se posait la question et surtout le temps qu’il fallait mettre en mettant les épis, les malades grabataires mais le prestataire lui disait que je dois me protéger alors les accompagnants étaient mécontents et les malades lui-même pouvait aussi être mécontent

**P 8: IA_02_AS_IT1.rtf - 8:19 [Répondant : Si quelque part il..] (42:42) (Super)**

Codes: [Ressources_humaines_charge_travail]

No memos

Répondant : Si quelque part il y a eu une petite difficulté parce qu’il fallait travailler dans les structures, il fallait notifier et investiguer les cas, il fallait suivre les contacts alors il y a eu un problème sérieux. Maintenait après quand on a commencé à utiliser les relais communautaires qui pouvaient nous aider dans les suivies des contacts, là ça nous a soulagé un peu. Mais au début, c’était vraiment difficile

**P 8: IA_02_AS_IT1.rtf - 8:20 [Répondant : Si je peux parler ..] (44:44) (Super)**

Codes: [Structure-Service_Utilisation_population]

No memos

Répondant : Si je peux parler par exemple de la consultation curative, juste quand la population a compris qu’au centre de santé, on est en train d’envoyer les gens au CTE, ils sont restés, ils ne se sont pas présentés alors cet indicateur a souffert et c’est vers la fin qu’ils ont compris et qu’ils ont commencé à fréquenter et surtout avec la gratuité des soins. Avec la gratuité des soins, ils ont trouvé que c’est bien de venir se faire soigner au centre de santé. Inducteur pour la CPN, il y a eu également des problèmes parce que nous-mêmes on se disait non, comme il n’y avait pas encore moyen d’avoir beaucoup des gants, on va porter combien des gants alors qu’il est question de manipuler, au début il y a eu aussi un problème à la CPN mais aussi à la CPS parce que quand on a commencé à vacciner contre Ebola, il y a des moments où on nous disait que vous voulez donner à nos enfants le vaccin contre Ebola, nous on ne viendra plus et il y a eu ces indicateurs qui sont aussi tombé

**P 8: IA_02_AS_IT1.rtf - 8:21 [Répondant : Je ne sais pas si ..] (46:46) (Super)**

Codes: [Recouvrement_coûts-des-soins_avant gratuité]

No memos

Répondant : Je ne sais pas si j’ai bien compris la question mais sinon nous on avait un tarif forfaitaire et le système de recouvrement des couts, était quand le malade pouvait venir et on lui fait la facture et s’il y a quelque chose il peut payer même en partie mais le constat était qu’on pouvait faire le recouvrement à 70% et le reste-là c’était des pertes bien que le comité de santé pouvait s’organiser pour essayer de rechercher les malades qui n’avaient pas payé. Malheureusement chez nous ici comme nous sommes à la périphérie de la ville, il y a ni numéro même si notre population n’est pas permanente, il est locataire et il pourra déménager à tout moment

**P 8: IA_02_AS_IT1.rtf - 8:22 [Répondant : Ce n’était pas du ..] (48:48) (Super)**

Codes: [Gratuité_Evolution]

No memos

Répondant : Ce n’était pas du tic au tac. Au début on travaillait comme d’habitude, il n’y avait pas de gratuité et là je peux dire comme il n’y avait pas des fréquentations étant donné que la population en voulait plus consulter le centre de santé et nous n’avons pas eu à dire par rapport à ça parce que le stock des médicaments qu’on avait, était resté dans la pharmacie et c’est quand on a commencé la gratuité qu’on a écoulé les médicaments qu’on avait

**P 8: IA_02_AS_IT1.rtf - 8:23 [Répondant : Oui, elle a été bi..] (50:50) (Super)**

Codes: [Gratuité_Accueil_population]

No memos

Répondant : Oui, elle a été bien accueillie après explication parce qu’il y a eu des rumeurs que vous ne voulez pas aller au CTE, on va vous attraper et vous coupez les organes et maintenant on veut vous exterminer par les médicaments. on en a parlé mais avec l’information de bouche à l’oreille, ils ont fini par comprendre mais jusqu’aujourd’hui, il en a de ceux qui ne veulent pas prendre de ces médicaments parce que c’est gratuit. Dernièrement, j’étais avec le groupe des jeunes, il y a de ceux qui commencent à dire que nous savons que vos médicaments sont intoxiqués, c’est juste quand on parlait de riz avarie de OCC et PAM et un jeune a dit voilà on vient de découvrir qu’il y a aussi le riz qui peut exterminer les gens mais nous avons quand même essayé d’expliquer et moi je leur ai dit d’ailleurs ces gens qui nous financent ne nous donner pas des médicaments, ils nous donnent l’argent et c’est nous qui allons acheter les médicaments donc il ne faut pas imaginer qu’ils nous amènent des médicaments qui peuvent faire je ne sais quoi à la population mais nous on nous donne l’argent par cas et nous allons acheter les médicaments et là ils ont quand même fini par comprendre.

**P 8: IA_02_AS_IT1.rtf - 8:24 [Répondant : Chez nous, c’est p..] (52:52) (Super)**

Codes: [Gratuité_Structures-Services_concernés]

No memos

Répondant : Chez nous, c’est presque toutes les structures mais ailleurs il y avait sélection des structures.

**P 8: IA_02_AS_IT1.rtf - 8:25 [Répondant : Peut-être que nous..] (54:54) (Super)**

Codes: [Gratuité_Structures-Services_concernés]

No memos

Répondant : Peut-être que nous nos structures ont eu cette chance là mais ailleurs je ne sais pas comment on pouvait sélectionner les structures mais subitement on se retrouve que toutes nos structures sont dans la gratuité

**P 8: IA_02_AS_IT1.rtf - 8:26 [Répondant : A cette époque, on..] (56:56) (Super)**

Codes: [Gratuité_Structures-Services_concernés]

No memos

Répondant : A cette époque, on en avait dix mais maintenant on en a onze et c’est la dernière structure qui n’est pas dans la gratuité parce qu’il n’y a pas longtemps elle a commencé

**P 8: IA_02_AS_IT1.rtf - 8:27 [Répondant : Depuis le début, n..] (58:58) (Super)**

Codes: [Gratuité_Evolution]

No memos

Répondant : Depuis le début, nous avons commencé avec la gratuité et la première chose que nous avons déploré, c’est le retard dans le paiement des prestations, ça nous a créé beaucoup des problèmes parce qu’il fallait remplacer les médicaments mais avec ce retard, on ne parvenait pas il fallait s’endetter par ici par-là mais après quand on a paie les prestations, ça nous a donné un nouveau souffle par exemple vous pouvez voir ce beau bâtiment, c’est un bâtiment que nous avons érigé grâce au fond de la gratuité donc nous avons pu achever cette maison, bien qu’on avait déjà commencé donc nous avons juste fait les travaux de finissage avec cet argent. En tout cas nous ne déplorons pas beaucoup des choses, seulement le retard dans le paiement. Dernièrement ici, il y a eu un changement, on a encore ajouté que le malade présente sa carte d’électeur ou sa carte de baptême, alors tellement que dans notre communauté, il y a des gens qui sont intoxiqués par des rumeurs, ils ne comprennent pas comment quelqu’un qui doit être soigné doit exhiber sa carte voir même laisser une photocopie de sa carte et c’est le plus grand problème que nous sommes en train de vivre maintenant parce que la plupart des malades se posent cette question-là

**P 8: IA_02_AS_IT1.rtf - 8:28 [Répondant : Ici les gens sont ..] (60:60) (Super)**

Codes: [Gratuité_Rumeurs]

No memos

Répondant : Ici les gens sont intoxiqués qu’il y a cette contrée de Beni, Butembo qui a été vendu par le gouvernement et qu’on cherche à tout prix de délocaliser la population d’ici pour que le Rwandais viennent occuper la contrée et c’est comme ça que quand ils voient qu’on égorge les gens et là ils imaginent que ces cartes-là, c’est pour chercher à nous exterminer pour qu’à la fin de compte ces gens viennent habiter notre contrée. Même quand on demande le numéro de la parcelle, ils pensent qu’on va attribuer cette parcelle à une autre personne qui viendra tout en connaissant que ma maison c’est le numéro autant  et aussi il y a eu un autre élément qu’on a ajouté, c’est l’identification des ménages. D’abord pour nous les soignants, ça nous a ajouté du travail parce que pour compléter la fiche ça prend du temps et là encore quand nous demandons au malade de nous expliquer ils sont à combien dans la maison et si possible leurs âges et même si on est en train de les expliquer, ils se demandent ces gens qu’est-ce qu’ils cherchent et là nous avons encore du mal à les faire comprendre mais nous sommes en train d’aller molo-molo et nous conscientisons les chefs de cellule et nous avons organisé des réunions de CAC et voilà que nous avons organisé des réunions avec les jeunes et nous sommes en train d’expliquer et voilà comment les gens commencent à venir

**P 8: IA_02_AS_IT1.rtf - 8:29 [Répondant : La gratuité concer..] (62:62) (Super)**

Codes: [Gratuité_Structures-Services_concernés]

No memos

Répondant : La gratuité concerne les consultations curatives et au début on a commencé comme ça et il y avait aussi la gratuité pour la maternité, les accouchements, on ne paie pas, la CPS, on ne paie pas et la CPN, on ne paie pas. Mais maintenant on est resté qu’avec la consultation curative

**P 8: IA_02_AS_IT1.rtf - 8:30 [Répondant : Je ne sais pas si ..] (64:64) (Super)**

Codes: [Gratuité_Conséquences_utilisation_services]

No memos

Répondant : Je ne sais pas si je peux parler des conséquences parce que c’est sûr que quand les agents ne sont pas payés à temps, ils essayent de murmurés mais quand ils ont leurs salaires, voilà ils sont encore content. Mais la seule conséquence, c’est la période qu’on fait sans paiement, là les gens souffrent parce qu’ils n’ont pas où toucher pour leur survie

**P 8: IA_02_AS_IT1.rtf - 8:31 [Répondant : Oui, c’était les r..] (66:66) (Super)**

Codes: [Ressources humaines_Motivation_personnel]

No memos

Répondant : Oui, c’était les recettes locales

**P 8: IA_02_AS_IT1.rtf - 8:32 [Répondant : Ils sont mieux mot..] (68:68) (Super)**

Codes: [Ressources humaines_Motivation_personnel]

No memos

Répondant : Ils sont mieux motivés qu’avant parce que quand il y a l’argent ils sont mieux motivés

**P 8: IA_02_AS_IT1.rtf - 8:33 [Répondant : Oui en tout cas il..] (70:70) (Super)**

Codes: [Médicaments_disponibilité_fosa]

No memos

Répondant : Oui en tout cas il y a eu un problème parce qu’on a connu des ruptures des médicaments, beaucoup des ruptures, de temps en temps parce qu’on avait commencé la gratuité et il n’y avait même pas un comprimé de démarrage et nous avions vidé notre stock et c’est comme ça que nous sommes entrés en rupture de stock presque total, c’est comme aujourd’hui, nous on s’est déjà préparé pour la fin de la gratuité, nous avons pris notre argent et nous avons renouvelés notre stock de la pharmacie, c’était vraiment préparé et subitement on nous dit que la gratuité recommence encore, c’était vraiment brusque et nous on est s’attendait même pas à ça et on pose la question, cette gratuité on va la démarrer avec quoi ? et on nous non commencé d’abord et on va vous envoyer un peu des médicaments pour vous renforcer et nous avons commencé la gratuité au mois de février, voilà qu’aujourd’hui nous sommes à la fin du mois de mars, on a rien jusque-là, nous n’avons ni argent ni médicaments. C’est avant-hier qu’on nous a dit qu’il semblerait qu’il y a un peu des médicaments au BCZ que nous n’avons pas encore.

**P 8: IA_02_AS_IT1.rtf - 8:34 [Répondant : Il y a eu changeme..] (72:72) (Super)**

Codes: [SNIS_fonctionnement_Decrire_pdt MVE]

No memos

Répondant : Il y a eu changement, c’était surtout au niveau de la surveillance donc il y a eu amélioration de la notification des maladies à déclaration immédiate hebdomadaire et mensuelle. Là en tout cas tout le monde a ouvert l’œil et tout moment il y a eu un cas, en tout dans l’immédiat il fallait transmettre l’information. Il y a eu un peu de problème par rapport à la notification mensuelle, dès fois on était submergé par les activités de la riposte de tel sorte qu’on n’avait plus le temps de compléter un canevas et ça crée un retard dans l’expédition des rapports, un rapport qu’on envoie le 6, on pouvait se retrouver le 10 alors que le rapport n’est pas encore fini

**P 8: IA_02_AS_IT1.rtf - 8:35 [Répondant : Je crois que vous ..] (74:74) (Super)**

Codes: [SNIS_fonctionnement_Solutions aux défis]

No memos

Répondant : Je crois que vous voulais parler de la décentralisation des activités ou même la délégation du pouvoir ou des activités mais le problème est que nous tous même ceux qui peuvent être délégués était aussi dedans et là chacun avec ses activités et il n’y avait personne qui pouvait dire ah je prends ce canevas et je le complète mais malgré ce retard on prenait quand même tu temps, quelques minutes de repos, on complète et on envoie, mais ce qui serait mieux s’il y avait certaines personnes qui n’étaient pas occupées, on pouvait leur charger d’ailleurs ici chez nous ce n’est pas seulement l’IT qui fait le rapport, c’est tout le monde, chaque service produit un rapport de ses prestations et à la fin on peut centraliser et on envoie donc ça veut dire que presque tout le monde est impliqué dans la collecte des données.

**P 8: IA_02_AS_IT1.rtf - 8:36 [Répondant : Non, c’est la comp..] (76:76) (Super)**

Codes: [SNIS_fonctionnement_Decrire_pdt MVE]

No memos

Répondant : Non, c’est la compilation parce que les registres-là nous servent pour la surveillance, là vraiment après chaque consultation, on enregistrait tous les malades. En tout cas les registres étaient à jour.

**P 8: IA_02_AS_IT1.rtf - 8:37 [Répondant : Si je peux voir ce..] (78:78) (Super)**

Codes: [Gouvernance_soins santé]

No memos

Répondant : Si je peux voir ce qui se passait dans l’aire de santé, je peux dire que la capacité de prendre des décisions n’a pas été tellement affectée néanmoins il pouvait avoir un groupe des personnes qui pouvait contester la décision mais du mois on pouvait prendre une décision pour améliorer les choses et ça pouvait aller. Mais aussi les organes, les organes fonctionnent normalement parce que le comité de santé se réunissait et pouvait analyser la situation et prendre des décisions. Néanmoins il y a eu un petit problème par rapport à la gestion du personnel, il en a ceux sont partis et qui se disent encore agents du centre de santé et quand nous avons intéressé notre hiérarchie par rapport à cette situation, en tout cas on n’a pas eu une solution favorable donc je peux vous dire qu’il y a pas eu beaucoup des problèmes par rapport à la gouvernance en générale

**P 8: IA_02_AS_IT1.rtf - 8:38 [Répondant : Peut-être du point..] (80:80) (Super)**

Codes: [Gouvernance_soins santé]

No memos

Répondant : Peut-être du point de vue financier, là oui parce que quelqu’un pouvait venir et dire moi je vous donne cet argent et vous allez l’utiliser dans un tel secteur alors il y a de fois où on vous amener de l’argent on vous dit juste pour le fonctionnement et les agents commencent à dire mais qui fait fonctionner le centre et là on se tiraille un peu parce que le bailleur lui dit c’est juste pour le fonctionnement et les agents disent le fonctionnement seul, comment il fallait qu’on ait aussi quelque chose

**P 8: IA_02_AS_IT1.rtf - 8:39 [Répondant : Merci, le grand ch..] (82:82) (Super)**

Codes: [Souhait_Changement_gestion_futures_épidémies]

No memos

Répondant : Merci, le grand changement qu’on doit faire, c’est le début de l’épidémie de commencer à travailler avec la base càd la communauté. Si ça bâclait c’est parce que les choses ont mal commencé parce que à cette époque-là on pouvait même voir sur terrain un communicologue mais qui a besoin d’un interprète, ça ne pouvait pas aller donc quand on a changé les choses, quand on a fait appel à la communauté même les résistances ont diminué sensiblement et voilà que l’épidémie est à la fin. Donc je veux parler des grands changements, c’est de travailler avec la communauté dès le début de l’épidémie

**P 8: IA_02_AS_IT1.rtf - 8:40 [Répondant : Les autres problèm..] (84:84) (Super)**

Codes: [Autres_Problèmes observés]

No memos

Répondant : Les autres problèmes, c’est toujours autour des rumeurs, des résistances, à part ça il n’y a pas eu d’autres problèmes mais seulement quand on a su gérer les rumeurs et les résistances, quand même, on se sentit dans notre bain.

**P 8: IA_02_AS_IT1.rtf - 8:41 [Répondant : Là si on peut dire..] (86:86) (Super)**

Codes: [Ressources humaines_Implication-agents-locaux]

No memos

Répondant : Là si on peut dire ça, c’est juste au début, cela rejoint ce que je venais de dire qu’il faudra commencer à travailler avec la base ou la communauté dès le début de l’épidémie. Si un IT peut dire que moi j’étais exclus, c’est au début parce qu’au début l’aire de santé qui n’avait pas des cas de contacts, l’IT ne pouvait pas se retrouver dans la riposte parce que si on dit que j’étais été exclu, c’est parce qu’on voit aussi l’argent donc l’aire de santé qui n’avait pas de cas de contact, je peux dire qu’il n’était pas prise en compte, c’est pourquoi au début il y a eu seulement trois IT qui étaient rémunérés parce qu’ils ont suivi les contact et après il y a eu d’autres qui se sont ajoutés et prise en compte et après quelques mois tout le monde a été prise en compte. Mais si ce sont les agents des structures, là c’est vrai parce qu’il y a eu ceux qui ont travaillé pendant seulement quelques jours et après ils ont arrêté par exemple il y a ceux qui ont fait les suivis des malades et après c’est suivi-là, ils devaient rester à la maison, il en a ceux qui ont travaillé dans la surveillance et surtout ici chez nous, quand nous avons placé certains infirmiers dans la surveillance et après nous avons commencé la gratuité et la gratuité nous a demandé une permanence au centre de santé et c’est comme ça que ça devenait un peu difficile que nous puissions soigner les malades et continuer la surveillance à base communautaire et même la sous coordination nous a dit que les infirmiers doivent rester dans le centre de santé et là ils ont encore une fois arrêter. Alors, c’est pourquoi ils disent qu’ils n’ont pas été impliqués, au début c’était ça

**P 8: IA_02_AS_IT1.rtf - 8:42 [Répondant : Encore une fois je..] (88:88) (Super)**

Codes: [Changements_depuis_Ebola]

No memos

Répondant : Encore une fois je veux vous parler dans le négatif et dans le positif. Si je peux commencer par le positif, maintenant les gens comprennent qu’on peut se protéger contre les maladies avec des gestes simples par exemple le lavage des mains, on peut faire la propreté pour se protéger contre les maladies mais le négatif, l’épidémie a créé beaucoup des conflits dans la société, ce n’est pas seulement un conflit entre soignant et un membre de la communauté mais dans la communauté elle-même il y a eu des conflits par exemple dans l’aire de santé Kyangike, une maman qui avait amené l’enfant de sa voisine aux soins ici pour la vaccination malheureusement quand l’enfant est rentré à la maison, sa mère était très mécontente et elle a fini par s’attaquer à sa voisine, pourquoi vous avez amené mon enfant recevoir le vaccin contre Ebola et cette affaire est allée même au niveau de la justice, c’est qu’un exemple alors il en a beaucoup, oh c’est tel qui avait appelé quand ma fille était malade et mon enfant est décédé au CTE, oh c’est elle qui m’avait dit d’amener l’enfant à tel endroit donc des conflits comme ça existent

**P 8: IA_02_AS_IT1.rtf - 8:43 [Répondant : Oui, un cas que no..] (90:90) (Super)**

Codes: [Considération_Survivants-MVE_population]

No memos

Répondant : Oui, un cas que nous avons eu, avant même que l’enfant revienne les gens ne voulaient plus rentrer dans la parcelle parce qu’ils se disaient au non nous serons contaminés et même quand l’enfant est revenu, il fallait qu’on ait expliqué à l’école qu’il ne constitue plus un danger pour les autres élèves donc ce qui fait que quelque part les vainqueurs étaient stigmatisés mais après beaucoup d’explications il pouvait être réintégré dans la communauté.

**P 8: IA_02_AS_IT1.rtf - 8:44 [Répondant : Bon, nous personne..] (92:92) (Super)**

Codes: [PCI_Comment_maintenir]

No memos

Répondant : Bon, nous personnellement ici nous avons l’habitude de faire de briefing chaque semaine, je crois qu’avec ce briefing, les gens ne vont pas oublier. De deux, nous avons pensé qu’il faudra déjà commencer à réfléchir comment on peut avoir des épis même après la fin de la riposte et cela on commençait à réfléchir comment on peut confectionner les masques, les bonnets, les histoires comme ça donc j’avais déjà posé la question au médecin ici qui est de la riposte disant que nous sommes presqu’à la fin mais à Butembo nous n’avons nulle part où on peut s’approvisionner en blouse par exemple, c’est pour cela qu’il me dira que juste après la riposte, il y aura quelque chose qu’on va organiser pour que les commerçants locaux puissent acheter ces habits et pour les revendre donc nous ici nous mettons plus l’accent sur le briefing chaque semaine.

**P 8: IA_02_AS_IT1.rtf - 8:45 [Répondant : Au début, ce n’éta..] (40:40) (Super)**

Codes: [Structure-Service_Utilisation_population]

No memos

Répondant : Au début, ce n’était pas toutes les structures qui étaient concernés, l’équipe de riposte a commencé à collaborer d’abord avec les centres de santé et quand les gens ont compris qu’au centre de santé, on est en train de convaincre les gens à aller au CTE, ils allaient au niveau des dispensaires, postes de santé et centres hospitaliers mais avec l’évolution, on a trouvé que ça constitue un autre risque et c’est là où on a commencé à avoir beaucoup des cas et on a intégré tous les dispensaires, centres hospitaliers et postes de santé, raison pour laquelle jusqu’aujourd’hui on a encore des malades qui viennent nous consulter parce qu’ils ont compris que partout vous allez trouver ce système mais au début ils pouvaient s’orienter au niveau des dispensaires

**P 9: IA_02_AS_IT2.rtf - 9:1 [Répondant : Je suis l’infirmie..] (2:2) (Super)**

Codes: [Répondant_Responsabilité_habituelles]

No memos

Répondant : Je suis l’infirmier titulaire de l’aire de santé Makasi et coordonnateur de l’aire de santé

**P 9: IA_02_AS_IT2.rtf - 9:2 [Répondant : D’une manière géné..] (4:4) (Super)**

Codes: [Répondant_Responsabilité_habituelles]

No memos

Répondant : D’une manière générale, chaque matin nous coordonnons les activités au niveau de l’aire de santé et placez chacun là où il doit assumer ses taches et au courant de la journée, surveiller et voir comment évoluer les activités

**P 9: IA_02_AS_IT2.rtf - 9:3 [Répondant : Dans notre aire de..] (6:6) (Super)**

Codes: [Début-Progression_Epidémie MVE]

No memos

Répondant : Dans notre aire de santé, il faut peut-être que je parlais de la façon dont Ebola est arrivé chez nous à Butembo, c’est un cas qui est venu d’une aire de santé voisine et l’aire de santé connue au nom de l’aire de santé Busongo, il serait un cas qui est venu de l’extérieur, d’une zone de santé affectée, la zone de santé de Mabalako et c’est delà que part le piste, le chemin, il est arrivé à Butembo et arrivé au centre de santé de Busongo mais la maladie n’était pas connue mais on entendait que la maladie à virus Ebola est déjà proche de chez nous. On l’a pris en charge comme d’habitude, on a pensé à d’éventuelles maladies que nous soignions tous les jours mais les prescriptions habituelles n’ont pas données des bons résultats et ce n’est qu’après quatre jours qu’on a dit referons ce cas ici et on l’a référé aux cliniques anniversaires, c’est là qu’on a tiré beaucoup des conclusions sur la maladie et c’est là qu’on a fait des prélèvements pour arriver à dire que c’est un tel cas que autre cas habituel. C’est comme ça que nous avons connu le premier cas à maladie à virus Ebola à Butembo. L’autre cas était arrivé de la zone de santé voisine, bon là on apprendra que c’était un cas qui est venu d’une femme qui a connu un épisode maladie ici et arrivée aux soins qu’on constatera que les signes d’une autre maladie que le cas courant, c’est comme ça que nous avons connu les cas de la maladie à virus Ebola dans la zone de santé de Butembo et la zone de santé de Katwa, la zone de santé voisine. Mais maintenant dans l’aire de santé, vous savez que la nouvelle n’était pas comprise étant donné qu’aucune notion d’épidémie n’était connue dans notre zone de santé, dire épidémie était une notion nouvelle et je dirai au niveau sanitaire et au niveau de la communauté, ça c’est la première chose. Deuxième chose, la politique a intervenue, ce qui a empiré la situation donc le fait que l’épidémie ne soit pas connue, la politique est entrée, la sécurité a intervenue, cela a abimé ou a mal avancé la riposte, c’est comme ça que je peux vous dire. Ces éléments-là on impactait d’une manière négative la riposte, c’est comme ça que les gens ont été contaminés et la population a cru à la fausseté que la véracité de la maladie qu’on allait souffrir

**P 9: IA_02_AS_IT2.rtf - 9:4 [Répondant : Je dirai que la po..] (8:8) (Super)**

Codes: [Politique-Sécurité_conséquences]

No memos

Répondant : Je dirai que la politique s’est mal tombée, c’est arrivé au moment où on faisait la compagne et les élections et pour se chercher les voix et pour attirer la population, les politiciens on dit que c’est faux, ce n’est pas une maladie, c’est un business, c’est ceci ou cela et la population a plus fait confiance aux politiciens qu’aux agents de santé

**P 9: IA_02_AS_IT2.rtf - 9:5 [Répondant : Après qu’il ait be..] (10:10) (Super)**

Codes: [Considération_Ebola_population]

No memos

Répondant : Après qu’il ait beaucoup des morts, l’attention a été attirée, les gens sont en train de mourir, après beaucoup des morts la conscience est enfin revenue avec la surveillance à base communautaire avec la sensibilisation quotidienne a amené la population à comprendre que c’est vrai, c’est une maladie

**P 9: IA_02_AS_IT2.rtf - 9:6 [Répondant : Avant que les gens..] (12:12) (Super)**

Codes: [Considération_Ebola_population]

No memos

Répondant : Avant que les gens qui partent à la sensibilisation, ils disent que la population n’avait pas le vrai message et ce qu’ils avaient c’était les ont dit, ce qu’on n’a pas sûr que ça existe, on y croit pas directement mais seulement le fait que les gens sont morts pour finir par comprendre que la maladie est là dès part le principe qu’on leur demandait de faire, les précautions préventives

**P 9: IA_02_AS_IT2.rtf - 9:7 [Répondant : dans la gestion de..] (14:14) (Super)**

Codes: [Répondant_Responsabilité_pdt MVE]

No memos

Répondant : dans la gestion de cette crise, nous avions du pain dans la planche étant donné que notre système avait des failles dans le cadre d’organisation. Je veux parlais de quoi ? Lorsque la riposte est venue, l’information est venue d’en haut que de venir de la base, de le communauté, alors quand c’est venu d’en haut, c’était d’abord mal perçu et il y a eu des nouveaux visages dans les milieux que de voir les visages habituels. Je veux parler des relais communautaire, dans notre système, ce que nous appelons cellule d’animation communautaire n’existe pas et c’est elle qui devait d’abord parler à la base et c’est dans les évaluations quotidienne qu’on a constaté qu’il fallait d’abord animer les cellules d’animations communautaires ou carrément mettre en place un système de surveillance quotidienne active, c’est comme ça que lorsqu’on est arrivé à mettre en place les enfants de chaque cellule, de chaque rue et qui passaient chaque jour en ne parlant que de la maladie, en cherchant les malades et en informant la communauté qu’on arrive à comprendre que c’est juste de voir les visages de gens qui pouvaient parler de la maladies dans leur communauté

**P 9: IA_02_AS_IT2.rtf - 9:8 [Répondant : Moi je veux dire q..] (16:16) (Super)**

Codes: [Répondant_Responsabilité_pdt MVE_Depuis quand ?]

No memos

Répondant : Moi je veux dire que tout ce que nous faisons vient de votre hiérarchie mais la hiérarchie nous a appelés quand les activités commençaient déjà. C’est pourquoi je disais qu’il y avait une certaine entorse quand l’information devait venir de nous vers la population, c’est plutôt venu d’une autre personne vers la population et cette personne n’était pas connue de la population

**P 9: IA_02_AS_IT2.rtf - 9:9 [Répondant : les choses ont cha..] (18:18) (Super)**

Codes: [Répondant_Responsabilité_pdt MVE_Depuis quand ?]

No memos

Répondant : les choses ont changé maintenant qu’on a commencé à éduquer et nous locaux, les chefs locaux, les leaders locaux, les enfants des différentes cellules et des différentes avenues

**P 9: IA_02_AS_IT2.rtf - 9:10 [Répondant : deux choses, là-ba..] (20:20) (Super)**

Codes: [Structures-Services_fonctionnement]

No memos

Répondant : deux choses, là-bas quand en ce qui concerne la santé, les infrastructures étaient toujours utilisées et le personnel était toujours présent en vue de servir toujours la communauté mais étant donné que les choses nouvelles qui sont venues dans le système, il n’y avait pas question de triage, triage est une chose qui n’existait pas et au lieu de comprendre que le thermo-flash est un appareil qui servait à prélever la température, on a transformé cette information en disant que c’est un moyen de transmettre la maladie à certains endroit ; c’est de un ; et quand c’était déjà arrivé dans la communauté, on ne venait plus à la structure à cause du triage et du lavage des mains et les gens faisait le contraire de ce qu’on leur demandait et cela impactait négativement nos activités quotidiennes

**P 9: IA_02_AS_IT2.rtf - 9:11 [Répondant : également, c’était..] (22:22) (Super)**

Codes: [Structure-Service_Utilisation_population]

No memos

Répondant : également, c’était le chlore qui dérangeait certaines personnes qui disaient qu’il y a la mauvaise odeur du chlore utilisé dans l’eau au début bien qu’on est changé en savon vers la fin parce qu’il y a de ceux qui ont connu des irritations a à cause de la mauvaise préparation du chlore ainsi de suite, tous ces éléments-là pouvaient amener la communauté à ne pas venir immédiatement venir demander les soins

**P 9: IA_02_AS_IT2.rtf - 9:12 [Répondant : je disais quoi ? Q..] (24:24) (Super)**

Codes: [Triage_Considération_population]

No memos

Répondant : je disais quoi ? Quand on craignait le tri, c’est pour être envoyer au CTE

**P 9: IA_02_AS_IT2.rtf - 9:13 [Répondant : Je dirais que tout..] (26:26) (Super)**

Codes: [Triage_Considération_population]

No memos

Répondant : Je dirais que toutes les structures ont intégrées le tri d’une manière progressive et là où on trouvait que les gens se retrouvent, il a fallu qu’on fasse attention pour placer le tri

**P 9: IA_02_AS_IT2.rtf - 9:14 [Répondant : ça continue même j..] (28:28) (Super)**

Codes: [Service_laboratoire_fonctionnement]

No memos

Répondant : ça continue même jusqu’au moment où nous parlons parce qu’on ne faisait plus certains examens par exemple au laboratoire comme le test de paludisme et d’ailleurs le bureau central nous avait retiré ces test et même la prise en charge du paludisme nous été défendue au dès part, ce n’est après que nous sommes revenus tout n’en a recherchant pas

**P 9: IA_02_AS_IT2.rtf - 9:16 [Répondant : on ne soignait pas..] (32:32) (Super)**

Codes: [Service_prise-en-charge_fonctionnement]

No memos

Répondant : on ne soignait pas d’abord, on l’envoyait immédiatement au CTE, c’est là où on le soignait

**P 9: IA_02_AS_IT2.rtf - 9:17 [Répondant : parce que les sign..] (30:30) (Super)**

Codes: [Service_prise-en-charge_fonctionnement]

No memos

Répondant : parce que les signes du palu peuvent cacher la maladie à virus Ebola

**P 9: IA_02_AS_IT2.rtf - 9:18 [Répondant : Bon étant donné qu..] (34:34) (Super)**

Codes: [Service_prise-en-charge_fonctionnement]

No memos

Répondant : Bon étant donné qu’on ne savait pas comment on prenait en charger Ebola là-bas et notre tâche consistait à envoyer les cas suspects

**P 9: IA_02_AS_IT2.rtf - 9:19 [Répondant : il y avait par exe..] (38:38) (Super)**

Codes: [Service_maternité_fonctionnement]

No memos

Répondant : il y avait par exemple même l’accouchement ou pendant les grossesses, dès qu’une femme pouvait présenter une perte sanguine, on ne pouvait pas hasarder à la toucher, il fallait carrément l’envoyer parce que Ebola sans douté peut faire fuir les grossesses des femmes alors cela pouvait être allé investiguer au CTE

**P 9: IA_02_AS_IT2.rtf - 9:20 [Répondant : nous avions égalem..] (40:40) (Super)**

Codes: [Service_maternité_fonctionnement]

No memos

Répondant : nous avions également peur parce qu’on avait peur d’entrer en contact avec le sang. Je peux dire durant presque une année nous n’avions dirigé que deux accouchements bien que nous n’avions pas beaucoup

**P 9: IA_02_AS_IT2.rtf - 9:21 [Répondant : oui] (42:42) (Super)**

Codes: [Service_maternité_fonctionnement]

No memos

Répondant : oui

**P 9: IA_02_AS_IT2.rtf - 9:22 [Répondant : ou carrément elle ..] (44:44) (Super)**

Codes: [Service_maternité_fonctionnement]

No memos

Répondant : ou carrément elle se dirigeait ailleurs croyant que nous allons immédiatement

**P 9: IA_02_AS_IT2.rtf - 9:23 [Répondant : si je peux parler ..] (46:46) (Super)**

Codes: [Structures-Services_fonctionnement]

No memos

Répondant : si je peux parler de la tuberculose, les malades pouvaient aussi craindre de venir au centre de santé et comme ça on avait parrainé les malades par les relais communautaires. Ce sont les relais qui pouvaient les suivre à domiciles et les voir chez eux pour s’assuraient qu’ils continuent à prendre leurs médicaments. Par rapport à la planification familiale, les clients avaient diminués et pour le dépistage et la prise en charge du VIH, notre structure n’était pas dans le programme

**P 9: IA_02_AS_IT2.rtf - 9:24 [Répondant : par rapport à la q..] (48:48) (Super)**

Codes: [Qualité_soins_Prestataires]

No memos

Répondant : par rapport à la qualité des soins moi en mon sens, je pense qu’on faisait notre tâche parce que les soins étaient ambulatoire et on prescrivait les médicaments tel qu’on fait le diagnostic et les soins étaient donné en totalité et on fixait un rendez-vous. Il nous était interdit d’observer du fait que nous sommes dans les structures de base

**P 9: IA_02_AS_IT2.rtf - 9:25 [Répondant : nous savons qu’il ..] (50:50) (Super)**

Codes: [Ressources_humaines_Disponibilité et perturbations]

No memos

Répondant : nous savons qu’il y avait un besoin maintenant qu’on avait instauré le système de triage et on avait ajouté certaines autres personnes pour aider les structures à répondre aux besoins de triage dans le cadre de maintenir une bonne hygiène et répondre au principe de la PCI mais le personnel était toujours suffisant.

**P 9: IA_02_AS_IT2.rtf - 9:26 [Répondant : dès fois ça pertur..] (52:52) (Super)**

Codes: [Ressources_humaines_Disponibilité et perturbations]

No memos

Répondant : dès fois ça perturbait parce que je suis infirmier titulaire et la formation j’étais pris comme formateur, mon absence impactait vraiment sur le service. Dès que j’étais absent, on disait est-ce que tu peux revenir alors que j’étais autorisé d’aller ailleurs donc mon absence n’était pas seulement une absence physique mais également à certaines exigences de la structures

**P 9: IA_02_AS_IT2.rtf - 9:27 [Répondant : non, nous nous ne ..] (54:54) (Super)**

Codes: [Ressources_humaines_Disponibilité et perturbations]

No memos

Répondant : non, nous nous ne sommes pas directement détachés mais on avait entendu qu’il y a de ceux qui ont choisi de rester carrément dans la riposte que dans leurs postes de routine

**P 9: IA_02_AS_IT2.rtf - 9:28 [Répondant : il y a trois mois ..] (56:56) (Super)**

Codes: [Structures-Services_fonctionnement]

No memos

Répondant : il y a trois mois que je suis ici mais dans l’aire de santé où j’étais, je connais une structure qui avait fermé parce qu’on l’a gêné de collaborer, il a vraiment fermé

**P 9: IA_02_AS_IT2.rtf - 9:29 [Répondant : avant la gratuité,..] (60:60) (Super)**

Codes: [Recouvrement_coûts-des-soins_avant gratuité]

No memos

Répondant : avant la gratuité, le mode était payant donc il y avait une certaine tarification qu’on respectait et au quelle était soumis la communauté pour recevoir les soins

**P 9: IA_02_AS_IT2.rtf - 9:30 [Répondant : je disais qu’il y ..] (62:62) (Super)**

Codes: [Recouvrement_coûts-des-soins_avant gratuité]

No memos

Répondant : je disais qu’il y avait un tarif forfaitaire donc tout le monde payer la même chose quel que soit la maladie donc les enfants autant quel que soit les actes, les adultes autant quel que soit les actes aussi

**P 9: IA_02_AS_IT2.rtf - 9:31 [Répondant : Non, chose nouvell..] (64:64) (Super)**

Codes: [Recouvrement_coûts-des-soins_avant gratuité]

No memos

Répondant : Non, chose nouvelle. Il y en a mais la communauté ne sait pas encore versée

**P 9: IA_02_AS_IT2.rtf - 9:32 [Répondant : la gratuité a conc..] (66:66) (Super)**

Codes: [Gratuité_Structures-Services_concernés]

No memos

Répondant : la gratuité a concerné à mon sens presque toutes les structures privées ou publiques.

**P 9: IA_02_AS_IT2.rtf - 9:33 [Répondant : oui, c’est le gouv..] (68:68) (Super)**

Codes: [Gratuité_Partenaire d'appui]

No memos

Répondant : oui, c’est le gouvernement à travers PDSS

**P 9: IA_02_AS_IT2.rtf - 9:34 [Répondant : oui, c’est très ju..] (70:70) (Super)**

Codes: [Gratuité_Interruption]

No memos

Répondant : oui, c’est très juste parce qu’en mon sens nous avons encore repris à partir du 16 février de cette année, il y a le mois d’aout 2019 où on n’a pas fait ça et peut-être c’était le temps pour la hiérarchie d’étudier encore comment reprendre ainsi de suite. C’est que nous déplorions est qu’il y avait un retard de payer mais aussi que nous trouvions la charge qui augmentait et peut-être qui ne correspondait pas selon nous ce qu’on devait recevoir vu la quantité de personnes qui venaient solliciter les soins

**P 9: IA_02_AS_IT2.rtf - 9:35 [Répondant : pour les choses de..] (72:72) (Super)**

Codes: [Gratuité_Information_population]

No memos

Répondant : pour les choses de la santé, normalement il est interdit qu’on fasse une publicité mais le message nous le passons par les relais communautaire, nous livrons les messages aux relais communautaires et les relais communautaires l’amenaient dans la communauté

**P 9: IA_02_AS_IT2.rtf - 9:36 [Répondant : oui] (74:74) (Super)**

Codes: [Ressources_humaines_charge_travail]

No memos

Répondant : oui

**P 9: IA_02_AS_IT2.rtf - 9:37 [Répondant : ça ne varie pas, d..] (76:76) (Super)**

Codes: [Ressources humaines_Motivation_personnel]

No memos

Répondant : ça ne varie pas, depuis le début c’est toujours 3,5$, peu ou plus c’est toujours 3,5$ et maintenant on se limité aux consultations curatives pour les malades ambulatoires selon le dernier avenant mais le tout premier, il y avait d’autres rubriques qui étaient concernés telles que accouchement, petite chirurgie

**P 9: IA_02_AS_IT2.rtf - 9:38 [Répondant : oui,] (78:78) (Super)**

Codes: [Ressources humaines_Motivation_personnel]

No memos

Répondant : oui,

**P 9: IA_02_AS_IT2.rtf - 9:39 [Répondant : nous trouvons que ..] (80:80) (Super)**

Codes: [Ressources humaines_Motivation_personnel]

No memos

Répondant : nous trouvons que c’est mieux s’il faut être franc que la tarification forfaitaire

**P 9: IA_02_AS_IT2.rtf - 9:40 [Répondant : moi je dis que les..] (82:82) (Super)**

Codes: [Gratuité_Accueil_population]

No memos

Répondant : moi je dis que les gens sont là bien qu’on nous ajoutait d’autres charges de remplir d’autre partie du registre, c’est la communauté qui nous voir en mal parce que nous ne savons pas les prendre en charge en totalité en remplissant plusieurs registres au même moment, c’est qui peut nous demander d’ajouter du personnel et nous pensons que nous y allons petit à petit

**P 9: IA_02_AS_IT2.rtf - 9:41 [Répondant : le projet là de gr..] (84:84) (Super)**

Codes: [Gratuité_Accueil_population]

No memos

Répondant : le projet là de gratuité vous savez quand c’est gratuit cela veut dire que venir présenter vos plaintes. Au début-là c’était mal reçu parce qu’ils disent qu’ils nous cherchent pour nous conduire dans leur CTE alors on a commençait à nous indexer dire que par exemple il y a des endroits où on a brulé le triage, on a battu l’infirmier titulaire mais moi je suis revenu parce que je n’ai pas trouvé un grand danger.

**P 9: IA_02_AS_IT2.rtf - 9:42 [Répondant : ils ont collaboré ..] (86:87) (Super)**

Codes: [Agression-Menaces_Structure-Prestataire_raisons]

No memos

Répondant : ils ont collaboré en faveur de la riposte qui était mal comprise par la communauté, au début croyant que c’était une manière de faire le carnage parce qu’il y a en ceux qui ont transformé la vérité en carnage

**Interviewer : parlons des médicaments, si vous pouvez comparer la disponibilité des médicaments**

**P 9: IA_02_AS_IT2.rtf - 9:43 [Répondant : je veux dire une c..] (88:90) (Super)**

Codes: [Médicaments_disponibilité_fosa]

No memos

Répondant : je veux dire une chose, je crois que par rapport aux médicaments PDSS pouvait, il avait un compte à rendre, il ne nous a pas donné les médicaments disponibles et s’il venait, ceux qui couteraient moins cher, c’est ce qui est à un prix moins élevé, nous n’oublions jamais une boite de mebendazole qui nous achèterons à 156$, cela nous a tiqué et qui nous a poussé à dire que la gratuité n’a plus de sens vraie

Interviewer : est-ce que cela a affecté le fonctionnement des services ?

Répondant : oui, au lieu d’avoir beaucoup, on a eu que peu. Imagine qi on vous donnez que la boité de mebendazole et si on vous faisait des médicaments, on ne répondra pas à toutes les réquisitions donc le taux de satisfaction ne dépassait pas le 50%, c’était toujours moins de 50%

**P 9: IA_02_AS_IT2.rtf - 9:44 [Répondant : il n’y avait pas d..] (92:92) (Super)**

Codes: [Médicaments_disponibilité_fosa]

No memos

Répondant : il n’y avait pas des médicaments chez le fournisseur pour nous qui était chez Stramex

**P 9: IA_02_AS_IT2.rtf - 9:45 [Répondant : au fait quand je d..] (94:94) (Super)**

Codes: [SNIS_fonctionnement_Decrire_pdt MVE]

No memos

Répondant : au fait quand je dis avant Ebola, on était calme tant soit peu on répondait aux exigences alors que c’est pendant l’épidémie qu’il a eu beaucoup des bouleversements tantôt on ne remettait pas le rapport, tantôt on ne le faisait pas à temps. On a manqué à beaucoup et dans ces manquements là il a fallu même qu’il y a eu même des grèves qui ont été déclenché parce qu’on n’a pas été bien répondu par la hiérarchie, certaines activités ont été coupée parce qu’on se sentait menacer par la communauté donc il y a des histoires qui n’allaient pas bien. Nous avons dit dans le cadre de cette histoire de la grève de ne plus faire certaines activités parce que nous avions dit que ce sont les femmes qui menacent les personnels, arrêtons la CPS, la CPN et la vaccination et cela pouvait empiéter sur le système d’information, ce qu’on attendait et pourtant le vaccin était là et tous les intrants étaient là, ça c’est pendant l’épidémie mais nous sommes revenus à ça quand nous sommes attendus et tant soi peu on continue

**P 9: IA_02_AS_IT2.rtf - 9:46 [Répondant : par rapport à la p..] (96:96) (Super)**

Codes: [SNIS_fonctionnement_Decrire_pdt MVE]

No memos

Répondant : par rapport à la promptitude, là j’ai dit vraiment on n’était pas prompt. Dans la complétude, on pouvait également manquer certaines données donc je ne saurai pas dire que c’était une négligence mais c’était une sorte de submersion par les activités d’autant plus que quand j’étais dans une formation je ne savais pas remplir les documents que je vais utiliser à la fin du mois pour la récolter des données

**P 9: IA_02_AS_IT2.rtf - 9:47 [Répondant : je crois que si un..] (98:98) (Super)**

Codes: [SNIS_fonctionnement_Solutions aux défis]

No memos

Répondant : je crois que si une autre crise pouvait arriver, je prierai d’abord qu’il n’y ait pas d’insécurité, il ne faut pas qu’il ait l’insécurité parce que cela affecte tout le monde. Si on dit ville morte et vous voulais vous hasarder, c’est chez vous qu’on va venir et vous faire du mal alors il ne faut pas qu’il y ait de l’insécurité pour essayer tant soi peu de faire quelque chose. Nous sommes en train de travailler porte fermée ici donc en craignant que comme la fois passait on a cassé beaucoup des vitres ici donc on a quand même le peur dans le vendre mais nous disons que l’insécurité affecte les activités

**P 9: IA_02_AS_IT2.rtf - 9:48 [Répondant : il faut que notre ..] (100:100) (Super)**

Codes: [SNIS_fonctionnement_Solutions aux défis]

No memos

Répondant : il faut que notre autorité pense à nous pour que chacun puisse rester à son poste. Un la prime de risque qui ne répond pas immédiatement à notre plan et tout le monde n’est pas salarié dans toutes les structures et services et c’est aussi insuffisant

**P 9: IA_02_AS_IT2.rtf - 9:50 [Répondant : ça revient surtout..] (106:106) (Super)**

Codes: [Agression-Menaces_Structure-Prestataire_solution]

No memos

Répondant : ça revient surtout à travers les relais communautaires quand nous avons des assises, on se fait accompagner par ces relais communautaire pour pouvoir parler à la communauté

**P 9: IA_02_AS_IT2.rtf - 9:51 [Répondant : lorsque la communa..] (104:104) (Super)**

Codes: [Agression-Menaces_Structure-Prestataire_raisons]

No memos

Répondant : lorsque la communauté a menacé le personnel de santé, le lien avec la communauté a été rompu, parler à la communauté s’était déjà difficile. Si vous le voulez, c’est vous exposer, c’était vraiment difficile que nous sommes en train d’y revenir encore petit à petit maintenant que nous allons vers la fin de la riposte. Vraiment le lien était vraiment rompu

**P 9: IA_02_AS_IT2.rtf - 9:52 [Répondant : je crois qu’ici il..] (108:108) (Super)**

Codes: [PCI_Comment_maintenir]

No memos

Répondant : je crois qu’ici il fallait de la négligence ou de l’ignorance, c’était des choses qui pourtant étaient prise à l’école donc ce n’était pas seulement contre Ebola qu’il fallait appliquer la PCI mais contre toutes les autres maladies infectieuses. S’il faut encore parler de la PCI, ça nous a encore remémoré beaucoup de bonne chose donc il nous faut les approprier et continuer à les appliqués vraiment. Il faut continuer à mettre l’eau et le savon pour la communauté et les prestataires continuent à mettre les épis, protéger la communauté pour éviter la propagation.

**P10: IA_02_BCZS_AG.rtf - 10:1 [Répondant : dans la zone de sa..] (2:2) (Super)**

Codes: [Répondant_Responsabilité_habituelles]

No memos

Répondant : dans la zone de santé de Butembo, je suis le gestionnaire où je gère les ressources et nous avons trois ressources : les ressources humaines, les ressources matérielles et les ressources financières. Ce n’est pas facile mais on essaie de s’en tenir pour regarder tout ce qui se passe dans nos différentes structures

**P10: IA_02_BCZS_AG.rtf - 10:2 [Répondant : on est à l’épidémi..] (4:4) (Super)**

Codes: [Répondant_Responsabilité_pdt MVE]

No memos

Répondant : on est à l’épidémie depuis septembre 2018, effectivement ici à Butembo, c’est zone de santé de Butembo qui avons enregistré le premier cas, c’était dans l’aire de santé de Bushundo et après détection de ce premier cas, il y a eu deux de nos infirmiers qui ont été affectés, Dieu est dans ils sont sortis guéris. Alors dans le cadre de la riposte, je fais la logistique entant que gestionnaire c’est normal donc j’ai participé à la gestion e certains biens qu’on a donné.

**P10: IA_02_BCZS_AG.rtf - 10:3 [Répondant : dans notre contrée..] (6:6) (Super)**

Codes: [Considération_Ebola_population]

No memos

Répondant : dans notre contrée, ça n’a pas été facile pour la population, pour notre population Ebola c’était quelque chose de fabriquer est injecté dans la population tout simplement pour éliminer la population de cette contrée et vous savez la situation que nous menons dans cette partie, nous sommes à l’Est du pays avec le massacre dans la ville et territoire voisin à Beni ici, pour eux c’était difficile de massacrer la population de Butembo c’est ainsi qu’ils ont choisi de nous éliminer à travers le virus d’Ebola

**P10: IA_02_BCZS_AG.rtf - 10:4 [Répondant : Bon, nous sommes à..] (8:8) (Super)**

Codes: [Considération_Ebola_population]

No memos

Répondant : Bon, nous sommes à la dixième épidémie comme vous le savez, Ebola est toujours vécu dans d’autres provinces comme à l’Equateur mais chez nous comme on est du domaine nous savons que c’est une maladie qui est causée par un virus, ça n’a pas été facile de faire comprendre la population, c’est ainsi qu’en un moment il y a eu une rupture totale de confiance entre la communauté et le personnel soignant

**P10: IA_02_BCZS_AG.rtf - 10:5 [Répondant : Pour la population..] (10:10) (Super)**

Codes: [Considération_Riposte_Population]

No memos

Répondant : Pour la population comme il en croyait pas à la maladie et il croyait que c’était quelque chose de fabriquer, qui n’existe pas. Ils savaient que tous les gens qui étaient dans les équipes, les soignants aussi qu’ils étaient payés pour juste les éliminer, c’était ça

**P10: IA_02_BCZS_AG.rtf - 10:6 [Répondant : en général toutes ..] (12:12) (Super)**

Codes: [Structures-Services_fonctionnement]

No memos

Répondant : en général toutes les structures que nous contrôlons dans la zone de santé n’ont pas fonctionnées correctement juste au début. Il y avait certaines personnes qui se disent soignant, ils sont des tradipraticiens moderne (charlatans) qui maintenant commençaient à récupérer toute la population au détriment des structures normales

**P10: IA_02_BCZS_AG.rtf - 10:8 [Répondant : Oui, l’offre de se..] (16:16) (Super)**

Codes: [Structures-Services_fonctionnement]

No memos

Répondant : Oui, l’offre de services était complet et les équipes étaient déjà formées, il y avait des partenaires qui nous apporter des épis, équipements de protection individuelle alors tout fonctionne normalement donc il n’y avait pas de rupture

**P10: IA_02_BCZS_AG.rtf - 10:9 [Répondant : dans notre zone de..] (18:18) (Super)**

Codes: [Service_prise-en-charge_fonctionnement]

No memos

Répondant : dans notre zone de santé, il y a eu certaines restrictions, c’est notamment les examens dans les centres de santé pour minimiser certaines contaminations, il y a eu l’interdiction de faire le TDR pour le paludisme et surtout que les signes du palu se confondaient aux signes de la MVE et d’ailleurs, c’est ce qui est arrivé au centre de santé Bushondo où il y a eu une dame avec des signes similaires au paludisme et eux ils soignaient, ils pensaient qu’ils soignaient un malade du paludisme alors que c’était Ebola, et on a interdit dans les centres de santé de faire quelques examens dans le cadre de limiter d’abord le risque de propagation mais aussi d’alerte afin de s’assurer que la MVE est d’abord épargnée avant de soigner autre chose.

**P10: IA_02_BCZS_AG.rtf - 10:10 [Répondant : Pour la vaccinatio..] (20:20) (Super)**

Codes: [Service_vaccination_Fonctionnement]

No memos

Répondant : Pour la vaccination, normalement il y avait pas d’instruction, seulement dans notre communauté la vaccination s’est arrêtée de soi. C’était comment, la communauté tout en sachant qu’Ebola, c’est quelque chose de fabriquer et qu’on fait des injections et les autres disaient que ce sont des comprimés qu’on jetait dans le WC pour infecter les habitants de cette parcelle et ce sont les mamans qui ont commencé elles-mêmes au personnel de santé qui se présentait aux séances de vaccination ; le taux de participation était aussi très faible, les mamans ne venaient plus alors suite aux menaces et aux attaques des structures parce qu’on a connu vraiment une période très difficile, les structures étaient détruites et tous les médicaments et le personnel soignant pourchassé, ils dormaient mêmes dehors et ils avaient peur de leurs vies. On a même connu des catastrophes lors de cette épidémie dans notre province avec l’assassinant de l’un des médecins qui venaient nous aider il était camerounais, vraiment on a vécu des situations horribles pendant cette épidémie. C’est ainsi que les syndicats des infirmiers avait jugé bon de suspendre les activités de vaccination ou bien les activités préventives mais il n’y avait pas une instruction venant du gouvernement interdisant les activités préventives.

**P10: IA_02_BCZS_AG.rtf - 10:11 [Répondant : c’est un document ..] (22:22) (Super)**

Codes: [Service_vaccination_Fonctionnement]

No memos

Répondant : c’est un document de l’OMS

**P10: IA_02_BCZS_AG.rtf - 10:12 [Répondant : c’est vrai, vous s..] (24:24) (Super)**

Codes: [Triage_Considération_population]

No memos

Répondant : c’est vrai, vous savez ici chez nous c’est la première épidémie bien que ça soit la dixième dans le pays mais ici chez nous à Butembi et Beni, c’est la première épidémie alors les gens ne comprenaient pas qu’un malade pouvait être isolé du reste de la famille et surtout ici chez nous quand quelqu’un est malade, il faut qu’on montrait qu’on est solidaire avec lui, qu’on exprime son amour envers lui et reste à côté de lui et maintenant comme il fallait trier et mettre les autres en isolement et les autres les intégrés dans les structures et c’est qui a compliqué les choses

**P10: IA_02_BCZS_AG.rtf - 10:13 [Répondant : le nombre exact ça..] (26:26) (Super)**

Codes: [Agression-Menaces_Structure-Prestataire_raisons]

No memos

Répondant : le nombre exact ça sera un peu compliquer mais nous avons 38 structures qui sont normées et presque toutes les 38 ont été attaquées d’une manière ou d’une autre. Il y a ceux qui ont vraiment suivi des dommages mais les tous ont été attaquées.

**P10: IA_02_BCZS_AG.rtf - 10:14 [Réponse : il n’y avait pas de ..] (28:28) (Super)**

Codes: [Qualité_soins_Prestataires]

No memos

Réponse : il n’y avait pas de problème de qualité de soins, tout allait très bien comme avant la riposte, seulement a un niveau il y a le gouvernement qui nous aidé et qui a fait que nos structures soient encore réutiliser par la même population, il nous a fait un système de gratuité des soins et au début c’était vraiment une gratuité intégrale et c’est avec le temps qu’on a essayé de réduire le paquet et même jusqu’aujourd’hui on est encore dans la gratuité mais avec des restrictions mais au début c’est tout le monde qui se présentait et était prise en charge gratuitement et les malades sont revenus très nombreux et on a commencé à récupérer.

**P10: IA_02_BCZS_AG.rtf - 10:15 [Répondant : avant la gratuité,..] (30:30) (Super)**

Codes: [Recouvrement_coûts-des-soins_avant gratuité]

No memos

Répondant : avant la gratuité, c’est le malade qui supportait ces soins et c’était par acte

**P10: IA_02_BCZS_AG.rtf - 10:16 [Répondant : du 1er novembre 20..] (32:32) (Super)**

Codes: [Gratuité_Structures-Services_concernés]

No memos

Répondant : du 1^er^ novembre 2018 au 15 janvier 2019, la gratuité était totale donc tout malade qui arrivait, il était soigné sans problème. Alors du 16 janvier presque jusqu’à nos jours, il y a eu des petites pauses, il y a seulement quelques services qui ont été prise en compte, il y a la médecine interne, et dans cette médecine interne, on ne prend pas les malades chroniques comme les diabétiques, les hypertendus, ceux-là sont exclus, il y a la pédiatrie qui est prise en compte et les consultations curatives dans les centres de santé. Mais dans les centres hospitaliers, les consultations n’étaient pas prise en compte mais dans le deuxième contrat, on avait limité, il y avait la médecine interne, la pédiatrie, les consultations dans les centres de santé et les consultations référées dans les structures hospitalières mais aujourd’hui on ne prend plus en compte même ces consultations référées, on prend seulement en compte la médecine interne exclus le cas chronique et la pédiatrie ;

**P10: IA_02_BCZS_AG.rtf - 10:17 [Répondant : au début pour que ..] (34:34) (Super)**

Codes: [Gratuité_Structures-Services_concernés]

No memos

Répondant : au début pour que la structure soit prise en compte, on demandait quelques données, on avait pris en compte quelques indicateurs techniques et quelques indicateurs financiers. Alors toutes les structures qui ont été prompt et qui ont amené les données ont été prise en compte, vraiment il n’y avait pas le choix, on n’avait pas dit que seulement les structures normées ou les structures de l’Etat, c’était vraiment tout le monde et les privées et l’Etat, c’était une grâce. La seule condition était seulement de présenter les quelques indicateurs qu’on leur avait demandé des six, malheureusement il y avait quelques structures qui ne documentent pas et ils n’avaient pas amené les données et ils n’étaient pas prise en compte. Nous avons eu 29 structures qui ont amené leurs données et ce sont les 29 qui ont été prise en compte. Et dans les 29 structures, il y a 15 centres de santé parce que la zone de santé compte 15 centres de santé et les 14 autres structures hospitalières. Les hôpitaux hospitaliers, il y a les privés, les étatiques et les conventionnés aussi

**P10: IA_02_BCZS_AG.rtf - 10:18 [Répondant : oui, il y a eu des..] (36:36) (Super)**

Codes: [Gratuité_Interruption]

No memos

Répondant : oui, il y a eu des interruptions de changement des modalités par exemple au mois aout 2019, il n’y avait pas de gratuité, du 1^er^ février au 15 février, il n’y avait pas des gratuités et elle a commencé du 16 février 2020 jusqu’à maintenant et elle prend fin le 30 juin 2020

**P10: IA_02_BCZS_AG.rtf - 10:19 [Répondant : de toute les façon..] (38:38) (Super)**

Codes: [Gratuité_Information_population]

No memos

Répondant : de toute les façons quand on signe le contrat comme on commence le 16, on a déjà informé les gens que la gratuité commence le 16 février et ça prend fin le 30 juin alors si le partenaire se prononce qu’avant le 30 juin qu’il prolonge le contrat de la gratuité, il amène d’autres contrat et on communique toujours parce qu’on est en contact avec notre population vue le comité de santé et avec cette épidémie, il y a eu des partenaires qui nous ont aidé à mettre en place les cellules d’animations communautaire, c’est ainsi que quand il y a une information la population est vite informé à partir de ces organes

**P10: IA_02_BCZS_AG.rtf - 10:20 [Répondant : bon c’est normal c..] (40:40) (Super)**

Codes: [Gratuité_Rumeurs]

No memos

Répondant : bon c’est normal comme je vous ai dit que la maladie elle-même n’a pas été acceptée. Au début aussi la gratuité n’a pas été acceptée parce que pour notre communauté ils n’ont jamais vu là où se passe une gratuité pour eux au début il croyait qu’ils veulent nous éliminer complètement, c’est ainsi qu’il nous amène leurs médicament et ils étaient le premier qui ont bénéficié de ses soins et qui ont témoigné que ce sont les mêmes médicaments qu’on nous donnait auparavant au centre de sante quand on est malade c’est pour cela que tout le monde a adhéré, c’est presque tout le monde qui est venu, il y avait des engouements impossibles, les malades venaient même à 5 heures pour quitter un peu tard et les infirmiers qui quittaient très tard, débordés et il y avait même dans d’autres structures où on avait mis le système de jeton et de rendez-vous. On vous consulte le lundi et on vous donne un jeton pour revenir le lundi prochain pour la nouvelle consultation

**P10: IA_02_BCZS_AG.rtf - 10:21 [Répondant : avec la gratuité, ..] (42:42) (Super)**

Codes: [Gratuité_Conséquences_motivation_prestataire]

No memos

Répondant : avec la gratuité, il y avait des clauses qui étaient claires donc dans la gratuité dans les structures hospitalières, il y avait le 50% des recettes qui revenaient aux prestataires, le 20% des recettes revenait au fonctionnement même de la structure et le 10% dans l’investissement. Dans les centres de santé, c’était le 40% aux prestataires, les 15% au fonctionnement et l’autre pourcentage à l’investissement. Alors quand vous faites beaucoup d’actes, vous avez beaucoup des malades votre 50% est automatiquement au-delà de ce que vous receviez avant quand vous receviez les malades. Vraiment ça eu un impact positif sur les revenus surtout quand on a eu cette gratuité totale.

**P10: IA_02_BCZS_AG.rtf - 10:22 [Répondant : premièrement la gr..] (44:44) (Super)**

Codes: [Gratuité_Défis]

No memos

Répondant : premièrement la gratuité est dans la zone de santé même les autres actions de la riposte mais quand vous regardez dans nos écritures, il y a nulle part où on a tout ce fond des structures. Les partenaires, ils ne collaborent pas avec la zone de santé, ils amènent directement leur aide au niveau de la base au du centre de santé pour assurer la gratuité même les matériels, il y a des matériels qui étaient venus dans le cadre de la riposte, ce sont les matériels de la zone parce que ces sont les structures qui sont dans la zone qui ont été dotées de ces matériels, le bureau même de la zone de santé n’est pas informé maintenant quand vous arrivez dans la structure vous trouve ceci cela, vraiment le circuit n’a pas été respecté par les différents partenaires.

**P10: IA_02_BCZS_AG.rtf - 10:23 [Répondant : bon c’est vraiment..] (46:46) (Super)**

Codes: [Médicaments_disponibilité_fosa]

No memos

Répondant : bon c’est vraiment un casse-tête parce qu’avant même la riposte, on nous a demandé de faire de commande selon les structures et les consommations qu’on estime qu’on peut consommer s’il y a gratuité. Les commandes ont été envoyés mais on a servi le 1/10 de ces commandes, on a servi seulement 10% de ces commandes des médicaments de démarrage. Au début de la gratuité, il n’y avait pas tellement des problèmes. Dans la répartition, je crois avoir sauté la situation des médicaments, c’était 50% personnel, 30% médicaments, 15% fonctionnement et 5% investissement ; ça c’est pour les structures hospitalières. Et dans les centres de santé, c’était 40% pour le personnel, 30% médicaments, 20% fonctionnements et 10% investissement. Maintenant juste au début le 30% ici on donnait tout le 100% à la structure et la structure avait la possibilité de prendre le 30% pour s’acheter les médicaments et nous avions vu que toutes les structures qui évoluent bien et qui avaient même de réserve des médicaments et les médicaments étaient vraiment suffisants dans ce système. Donc avec le premier contrat il n’y avait pas des problèmes maintenant le 2^ème^ contrat, on a été surpris, il y a une phrase qui disait une ligne de crédit médicaments est ouverts au centre de recherche Stamex et c’est là où à commencer notre calvaire, c’était le contrat qui allait du 16 avril au 31 juillet et jusqu’aujourd’hui on a reçu seulement une partie des médicaments du contrat qui part du 16 avril au 31 juillet 2019 donc jusqu’aujourd’hui cette livraison n’est pas encore terminée, les autres contrats, celui qu’on a commencé en septembre jusqu’à décembre, c’était la même chose donc non servit aussi et celui qui a commencé en février jusqu’aujourd’hui non servit alors quelques réserves qu’ils avaient pendant le premier contrat est déjà aussi consommés, alors on attends

**P10: IA_02_BCZS_AG.rtf - 10:24 [Répondant : je veux rentrer su..] (48:48) (Super)**

Codes: [SNIS_fonctionnement_Decrire_pdt MVE]

No memos

Répondant : je veux rentrer sur le personnel après mais si vous pouvez le parler sur le système d’information sanitaire pour que vous preniez des décisions au niveau du bureau central, vous avez besoin que les données vous arrivez et pour cela il faut que les rapports vous arrivent et il faut que les gens puissent remplir les registres et les fiches, remplir les canevas et vous l’envoyez et si nous regardons la promptitude et la complétude et la qualité des données, qu’est-ce que vous pouvez dire sur les conséquences de la riposte que le système d’information ?

**P10: IA_02_BCZS_AG.rtf - 10:25 [Répondant : en général et beau..] (49:49) (Super)**

Codes: [SNIS_fonctionnement_Decrire_pdt MVE]

No memos

Répondant : en général et beaucoup détaillé, il y a beaucoup d’indicateurs qui ont souffert parce que le personnel même étaient pris dans beaucoup des choses et on était là il fallait beaucoup de temps pour conscientiser les gens quand on pouvait faire autre chose alors presque beaucoup d’indicateurs n’ont pas marchés.

**P10: IA_02_BCZS_AG.rtf - 10:26 [Répondant : je crois que si vo..] (51:51) (Super)**

Codes: [SNIS_fonctionnement_Decrire_pdt MVE]

No memos

Répondant : je crois que si vous voyez le contexte même dans lequel on est entrain dans Ebola, il y avait déjà autre chose qui a commencé à déranger les indicateurs et Ebola est venu seulement envenimer la situation. Vous vous habitez à Kinshasa mais il y avait des mouvements des grèves ici chez nous ; d’ailleurs l’épidémie, nous autres on ne savait pas parce que les agents étaient en grève donc on avait un problème de prime de risque donc avant même Ebola il y avait déjà des petits quoique maintenant Ebola est venu enfonçait la situation

**P10: IA_02_BCZS_AG.rtf - 10:27 [Répondant : je veux dire oui p..] (53:53) (Super)**

Codes: [Ressources_humaines_Disponibilité et perturbations]

No memos

Répondant : je veux dire oui parce que la riposte a perturbé les horaires ; quelqu’un qui comptait être là à midi, à 6 heures, il est obligé à se réveiller très tôt pour aller suivre les contacts de peur qu’il trouve qu’ils sont partis aux champs ou ils sont vagués à d’autres occupations et maintenant c’est après qu’il doit venir pour commencer qu’il doit faire comme boulot et cela dans toutes les structures. Nous avons même observé quelques cas d’évasion des ressources parce qu’avec la MVE, il y a eu beaucoup des partenaires qui cherchaient aussi la main d’œuvre et ils offraient mieux alors certaines personnes qui se sont orientés vers les partenaires et ont abandonné leurs structures alors ça nous a perturbé.

**P10: IA_02_BCZS_AG.rtf - 10:28 [Répondant : en ce niveau, il y..] (55:55) (Super)**

Codes: [Ressources_humaines_solution-local_Disponibilité et perturbations]

No memos

Répondant : en ce niveau, il y a notre division qui nous a aidés, il a fait une instruction pour dire que le 1^er^ juillet 2019 pour dire que chacun devrai retourner dans son poste d’attache et les personnes qui ne sont pas retournées, ont été déclarée comme des déserteurs. Ça n’a pas été facile parce que les personnes avec qui vous travaillent, ils sont capacités, ils ont une certaines capacités, c’est vrai qu’ils ont été déclaré déserteurs mais on a encore commencé avec des nouvelles équipes et vous voyez ce que ça fait comme effet

**P10: IA_02_BCZS_AG.rtf - 10:29 [Répondant : ok, pendant cette ..] (57:57) (Super)**

Codes: [Gouvernance_soins santé]

No memos

Répondant : ok, pendant cette période, je ne peux pas dire que la gouvernance était au top compte tenu de la situation, seulement on essayait de se dépasser pour maintenir certaines choses. Les organes qui était avant sont restés et il y a même le CAC qui s’est ajouté puisque au niveau de la base nous avions de CODESA et il y a les CAC qui se sont ajoutés au niveau de la base, ils se réunissent et produisent leur rapport et font ce qu’ils ont à faire. A notre niveau, il y a des analyses de rapport qui continuent à se faire, il y a des réunions des équipes cadre, il y a des CAC ; il y a des COGES qui se font, bon on peut avoir un problème de nombre de participants parce qu’ils pourront ceux qui sont dans autre chose mais au moins on est là et on a maintenu tout ce que nous avons. Il y avait parfois des réunions qui avortent, ça arrivait mais ça ne nous a pas empêché de croiser les bras, on est resté là mais avec beaucoup des difficultés.

**P10: IA_02_BCZS_AG.rtf - 10:30 [Répondant : bon premièrement, ..] (59:59) (Super)**

Codes: [Souhait_Changement_gestion_futures_épidémies]

No memos

Répondant : bon premièrement, c’est juste voir l’organisation même, il y a certaines chose qu’on a sauté, il y a eu même le recrutement pendant cette période d’épidémie qui n’a pas respecté les normes et qui nous a posé des problèmes quelque part. il y a la situation des partenaires qui sont là, ils arrivent dans une zone de santé comme ils étaient dans leur zone de santé, il sont en train d’appuyer la zone de santé, ce sont des choses à corriger, aujourd’hui si quelqu’un de l’extérieur venait et demandait à la zone pour toutes les activités de la riposte, vous avez utilisé combien en termes d’argent, en termes du personnel. Ce sont les partenaires qui avaient l’argent et qui faisaient tout avec le personnel, ça c’est quelque chose que je pourrais corriger si ça dépendait que de moi si une autre épidémie arrivait.

**P10: IA_02_BCZS_AG.rtf - 10:31 [Répondant : oui, il y a eu des..] (61:61) (Super)**

Codes: [Changements_depuis_Ebola]

No memos

Répondant : oui, il y a eu des apports, on ne peut pas seulement s’atteler sur des points à améliorer, il y a des apports même dans les structures. Premièrement si vous pouvez même regarder en face vous voyez déjà un infirmier qui est habillé en épi, ça ce sont les habitudes qui n’étaient pas là, chacun venait avec ses habits donc il y a déjà un changement de comportement de la part même des prestataires et de la communauté aussi, si vous arrivez là-bas, il y a des lavabos où il y a de l’eau et chacun cherche de l’eau pour se laver les mains avant d’entrer dans la structure même dans beaucoup des parcelles il y a ceux qui ont mis des lavabos. Dans les structure, il y a eu beaucoup d’acquis que nous avions reçu des partenaires ; il y a beaucoup d’ouvrage à impact qualité, il y a des structures qui n’avaient pas des incinérateurs mais si vous passez partout vous verrez des incinérateurs, dans chaque structure, les gens ne jettent plus des déchets partout, ils connaissent même le circuit de déchets donc il y a beaucoup des thématiques que nous avons appris. Il y a des latrines donc il y a beaucoup des choses que nous avons acquis de cette épidémie ; on continue à communiquer que ces bonnes pratiques soient pérenniser, que ça ne disparaissent pas avec l’épidémie. On est en train de renforcer la communication avec nos organes qui sont à la base notamment le CODESA et les CAC pour que la population sache qu’à part Ebola, il y a d’autres maladies qui peuvent nous attaquer grâce à la saleté, il y a la fièvre typhoïde même le palu, il faut qu’il détruise le gites à l’envers donc ce sont des bonnes pratiques que nous aimerions que ça soit pérenniser même avec la disparition de l’Ebola

**P10: IA_02_BCZS_AG.rtf - 10:32 [Répondant : bon je pense que ç..] (63:63) (Super)**

Codes: [PCI_Comment_maintenir]

No memos

Répondant : bon je pense que ça va perdurait parce qu’on a déjà renforcé la communication surtout nous nous sommes attelés à la communication des risques et on croit qu’après Ebola, on ne va pas s’arrêter car on va toujours continuer avec le même élan que nous avons et nous pensons que les bonnes pratiques vont rester

**P10: IA_02_BCZS_AG.rtf - 10:33 [Répondant : bon le financement..] (65:65) (Super)**

Codes: [PCI_Comment_maintenir]

No memos

Répondant : bon le financement mais il y a certaines choses qui ne demandent pas le financement, il y a déjà des tabliers et des bottes qu’ils ont déjà, s’il n’y a pas des partenaires une structures qui fonctionne normalement peut s’acheter une botte quand l’autre là est usé, c’est vrai qu’il d’autres chose qui demandent beaucoup de moyens mais il y a quand même le minimum qui peut se faire sans moyen et c’est que nous pouvons faire sans beaucoup des moyens, on préfère le faire même avant Ebola, on avait déjà initié des AG dans les codesa presque chaque comité de santé a son AG et nous avons notre animatrice communautaire qui fait des suivis des états généraux. C’est juste pour garder ces organes de base pour qu’ils fonctionnent qu’ils ne manquent pas des stylos parce qu’avec les petits projets qu’on peut faire, je vois qu’on va toujours garder le même élan après la riposte.

**P10: IA_02_BCZS_AG.rtf - 10:34 [Répondant : je disais qu’il av..] (66:67) (Super)**

Codes: [Autres_Problèmes observés]

No memos

Répondant : je disais qu’il avait un aspect que je voulais ajoutais cette face à la résistance, suite à ce que je vous disais que la maladie était fabriqué dans le but d’éliminer. Et ce qui avait aussi envenimé la situation, le ministère et les partenaires avaient largués sur terrain les gens qui ne savaient pas parler en langue locale donc c’était des gens qui ne parlaient que le français et lingala, les langues que la communauté ne comprenait pas et alors ils ont eu du mal à s’approprier de la riposte, c’est ainsi que de leur part ils ont développé une contre-offensive donc une autre chose, à laquelle il faut travailler donc il faut associer les locaux dans la riposte

Il y a des postes par exemple pour la coordination et le bureau et autre ça peut se faire par les experts mais le terrain et la communication, il faut juste briefer les gens du milieu qui savent bien les langues locales et qui peuvent s’exprimer librement. C’est ce qui a fait que les gens disent non pourquoi ces gens sont là alors que nous avons des gens qui ont étudier donc il y a quelque chose qu’ils nous cachent

**P10: IA_02_BCZS_AG.rtf - 10:35 [Répondant : c’est le gouvernem..] (69:70) (Super)**

Codes: [Gratuité_Partenaire d'appui]

No memos

Répondant : c’est le gouvernement par le PDSS et EUP FASS qui est une institution qui canalise les fonds dans le domaine de santé. Il y a l’Union Européenne qui appui ses six zones qu’il appuie depuis longtemps, il passe par EUP face pour qu’il canalise les fonds, ce n’est pas un partenaire

D’ailleurs EUP signifie Entreprise d’utilité publique et le FASS : c’est le fonds d’achat de système sanitaire donc une entreprise publique qui canalise les fonds qui appuie le système de santé.

**P10: IA_02_BCZS_AG.rtf - 10:36 [Répondant : ce sont des gens q..] (14:14) (Super)**

Codes: [Structure-Service_Utilisation_population]

No memos

Répondant : ce sont des gens qui profitaient de la position de la communauté comme pour la communauté pour eux la maladie n’existait pas et eux ils abondaient dans le même sens en faisant comprendre la population que vraiment la maladie n’existait pas et s’ils venaient chez eux ils vont les épargner de ces équipes-là qui les tuent. Tout en disant qu’il y a la maladie. Pour la population, les équipes de la riposte et le personnel soignant qui sont dans des structures reconnues normées, pour la population, ils injectent une injection au coup pour transmettre la maladie et après la personne mourrait. Les autres structures, les charlatans disent que nous n’avons pas cette injection, venaient nombreux et ces équipes-là ne vont pas vous voir, ils soignaient dans les chambres, dans des maisons, dans les lieux cachés pour essayer de gagner la confiance de la population et comme c’était ça la position de la population, ils étaient obligés de s’y rentrer

**P11: IA_03_COMM_SOCI.rtf - 11:1 [Répondant : Je suis monsieur B..] (2:2) (Super)**

Codes: [Répondant_Responsabilité_habituelles]

No memos

Répondant : Je suis monsieur Bitangala Emmanuel, je suis le secrétaire exécutif permanent de la société civile santé Nord-Kivu. La société civile santé, c’est une faitière, une plateforme de toutes les organisations qui travaillent dans le domaine de santé. Organisations nationales et internationales qui travaillent dans le domaine de santé, c’est une plateforme nationale qui a dans chaque province, une coordination provinciale et nous coordonnons les activités des organisations non-gouvernementales en matière de santé, voilà ce que nous sommes.

**P11: IA_03_COMM_SOCI.rtf - 11:2 [Répondant : En rapport avec vo..] (4:4) (Super)**

Codes: [Structures-Services_fonctionnement]

No memos

Répondant : En rapport avec votre question, Ebola est arrivée là où il est arrivé, c’est parce qu’il a trouvé un système de santé fragile, il a été constaté malheureusement que notre pays n’a pas un système politique et d’organisation sanitaire qui est convainquant, en tout cas le système de santé est à revoir parce que quand Ebola est arrivé, il venait directement d’une autre épidémie dans l’Equateur et c’est après l’Equateur qu’on a déclaré l’épidémie du Nord-Kivu, bien que cette épidémie du Nord-Kivu était bien avant que celle de l’Equateur, c’est seulement qu’à l’Equateur, on avait identifié l’épidémie bien avant qu’ici pendant qu’ici ça tue des gens au niveau de Mangina là-bas. Et alors quand le gouvernement a envoyé les gens pour venir faire la riposte ici, on a vu que les gens n’avaient pas les deux pieds sur terre, on a vu encore que c’est comme-ci c’était les débutants et pourtant c’était la dixième épidémie, une dixième épidémie, on ne pouvait pas donner la réponse comme-ci on était des novices, non, on ne pouvait même pas recruter des étrangers parce que c’est nous qui avons beaucoup plus d’expériences et c’est dans ce pays qu’on a parlé plus d’épidémies d’Ebola que dans d’autres pays, voilà pourquoi on ne pouvait pas vraiment attendre que les expatriés viennent d’autres pays pour nous aider, ils pouvaient venir nous aider avec le moyen financier pas le technique et si nous avions attendu les gens viennent nous aider avec leurs techniques cela signifie que le système est vraiment au rabais, le système est telle faible dans notre pays. Nous avons beaucoup décrié et quand ils sont venus moi-même j’étais là, je vous dis franchement ils sont restés dans les hôtels au lieu d’aller directement sur terrain là où il y avait de cas. Moi-même je posais la question de savoir pourquoi vous étés encore là, il y a déjà deux semaines depuis que vous êtes arrivés ici à Goma au lieu de descendre à Beni, vous êtes encore ici à Goma, qu’est-ce que vous êtes en train de faire ? Nous attendons le plan de riposte. C’est qui tellement drôle, un expert qui vient de Kinshasa envoyé par le ministère pour faire la riposte, il attend encore quoi, pourquoi il est appelé expert ? Alors qu’on venait d’une autre épidémie et en venant ici on attend encore un plan, plan d’où ? Les experts qui sont venus sont incapables d’élaborer un plan de riposte ? Pourquoi ? Comment ? Parce que bien sûr le plan devait exister parce que c’est la dixième épidémie et toutes ces épidémies devraient avoir des défis qu’on devait résoudre pour arriver à la perfection et cette fois-ci, on ne devait plus parler du plan de la riposte, alors qu’on devait revoir comment résoudre ces défis-là et ça devait nécessairement constitue un plan de riposte très costaud et c’est ce plan qu’on devait amener ici et l’adapter sur terrain par rapport aux réalités du terrain donc je peux vous dire qu’il y a eu plusieurs failles d’abord le système de santé est à revoir, vraiment à revoir, de deux, et quand ils sont venus, la plupart on amène les bureaucrates, alors qu’en épidémie ce n’est pas comme ça, il faut amener les gens de terrain alors qu’eux étaient seulement dans les bureaux. Ils ont oublié aussi qu’il faut travailler avec la communauté. ils sont arrivés ici avec les gens venus de Kinshasa et ils ont recruté les ouest-africains, les burkinabais et les camerounais, les gens de ces coins-là et arrivent sur terrain, ils ne connaissent même pas parler la langue locale, le swahili et quand ils parlent en français, c’est un français transformait en leur dialecte et les gens qui sont sur terrain ont compris que celui-ci n’est le nôtre ; ils comprennent déjà et cela est devenu un problème donc c’est bien connu quand on soigne un malade, il a plus confiance en une personne qu’il connait bien qu’à un étranger, cela joue beaucoup sur le plan psychologique, il a plus confiance en quelqu’un avec qui il parlait le même dialecte, quelqu’un qui parle avec lui une langue qu’il va comprendre et quand vous allez poser des questions a votre malade qui ne connait pas le français, il est dépaysé d’office.

**P11: IA_03_COMM_SOCI.rtf - 11:3 [Répondant : Dans les zones aff..] (6:6) (Super)**

Codes: [Structure-Service_Fermeture_pdt MVE]

No memos

Répondant : Dans les zones affectés par l’épidémie Ebola, on n’a pas enregistré vraiment des structures sanitaires qui ont fermé à cause de l’importance qu’on a accordé à Ebola par rapport à d’autres services mais par rapport à l’insécurité qui a sévit dans certains milieux, on a constaté qu’il y a des structures qui ont fermé. Mais qu’est-ce qu’on a constaté, on a même décrié cela, quand on a parlé de l’épidémie d’Ebola, c’est tout le personnel qui se lançait dans l’épidémie d’Ebola parce que là il y avait quoi. ? L’argent et d’autres services ont souffert, ils n’ont pas fermé mais ils ont souffert. Prenons l’exemple au niveau de la vaccination, même tous les chefs de services ont quitté Goma pour aller resté à Beni et Butembo, tout le monde, ils ont quitté pour aller là-bas et certains sont encore là, pour dire que un chef de service lui-même quitte Goma pour aller ailleurs donc les activités dans ce service-là barde donc ça n’a pas bien fonctionné même aujourd’hui certains chefs de services sont encore là qui attendent qu’on leurs paie parce qu’il y a de ceux qui attendent encore leur paiement donc voilà, ils n’ont pas fermé parce qu’il y a eu Ebola mais au contraire les services n’ont pas bien fonctionné pendant cette période-là. Parce que la plupart du personnel s’est adonné plus à l’épidémie d’Ebola qu’à d’autres activités ou d’autres paquets d’activités au sein des structures sanitaires, ça en commençant par les médecins chef de zones qui sont sur terrain, les agents de la DPS qui sont là encore pour le moment, la coordination de services, on les interpelle pour leur dire que vous êtes devenus coordonnateur d’Ebola seulement au lieu de coordonner les autres services de santé. Nous on n’a pas caché comme on représente la population et c’est ça notre rôle, nous avons joué ce rôle-là de les interpeller mais on ne pouvait pas les enfermer ou les obliger parce qu’on n’a pas le pouvoir mais au moins le pouvoir qui nous a été donné par la population, on l’a utilisé pour leur dire que c’est que vous êtes en train de faire ce n’est pas bon, revenait parce qu’Ebola est là, nous tous nous devons entrer dans Ebola pour faire la riposte mais nous ne pouvant pas laisser à leur triste sort d’autres maladies. on a vu choléra qui a sévit ici, qui allait prendre la place de tout le monde ici par rapport à Ebola. Donc c’est ce que moi je peux vous dire, réellement les structures n’ont pas fermé mais les services ont connu des sérieux problèmes pour leur fonctionnement normal parce que les médecins et les autres personnels ont abandonné pour aller là-bas à l’épidémie d’Ebola. La plupart des médecins ont coupé leurs contrats pour aller là-bas.

**P11: IA_03_COMM_SOCI.rtf - 11:4 [Répondant : Bon, ici nous parl..] (8:9) (Super)**

Codes: [Structure-Service_Utilisation_population]

No memos

Répondant : Bon, ici nous parlons des endroits où il y avait Ebola, les services étaient fréquentés mais partiellement parce que la population vivaient dans la phobie, dans la peur de dire que quand nous allons là-bas pour des soins nous risquons d’aller attraper Ebola. C’est pourquoi il y a eu d’autres services clandestins, d’autres structures clandestines se sont fait créer pendant cette période où la population avait confiance d’aller fréquenté ces services au lieu d’aller fréquenté des structures qui assuraient aussi la prise en charge d’Ebola donc la population quand ils ont vu que les gens qui les soignaient parce qu’ils n’avaient pas confiance en eux parce que le recrutement qui a été fait, on a beaucoup plus recruté ailleurs que le personnel local, il a fallu plus recruter localement beaucoup plus des gens bien connus dans la communauté et en ce moment la population devait avoir confiance à ces gens-là que d’amener les gens qui viennent de Kinshasa et d’autres de l’Afrique de l’ouest, d’autres qui ne savent même pas parler la langue locale ;

Il y avait beaucoup des rumeurs autour d’Ebola, ça faisait aussi peur à la population de fréquenter les structures, il y avait beaucoup des rumeurs que quand on va là-bas on va aller attraper le sida et quand vous allez, on va vous injecter Ebola comme cela est devenu un business et les autres disaient qu’on va vous tuer là-bas et la population craignait ça et en craignant, ils n’ont pas beaucoup plus fréquenté ces structures-là ou ces services-là, c’était vraiment à moitié

**P11: IA_03_COMM_SOCI.rtf - 11:5 [Répondant : La qualité est res..] (11:11) (Super)**

Codes: [Qualité_soins_Prestataires]

No memos

Répondant : La qualité est restée la même parce qu’il y avait des médicaments et la plupart des structures étaient appuyées donc la qualité était restée la même mais les prestataires qui n’étaient pas disponibles et cela faisait défaut

**P11: IA_03_COMM_SOCI.rtf - 11:6 [Répondant : Oui, on a vécu la ..] (13:13) (Super)**

Codes: [Gratuité_Conséquences_motivation_prestataire]

No memos

Répondant : Oui, on a vécu la gratuité des soins et qu’avant la gratuité, il y avait un taux faible de la fréquentation des structures par la population mais avec la gratuité, il y a eu augmentation de taux d’utilisation des services mais quand ils ont encore laissé la gratuité, nous sommes revenus au même niveau pour dire que la gratuité n’a pas changé les choses. D’ailleurs, cette gratuité devait préparer les gens, on devait avoir un système où les gens trouvent les soins gratuitement mais les gens pouvaient payer quand même quelque chose et cela pouvait entre dans les esprits des gens pour dire que je suis dans l’obligation de payer mes soins de santé même s’il y a quelqu’un qui paie par le système de tiers payant ou d’achat des services mais il me faudra que je paie quelque chose mais là c’était totalement gratuit et après cette période de gratuité, le taux est revenu encore au rabais. Qu’est-ce qui se passe maintenant ? Ils n’ont pas pensé à la cause de la non fréquentation, c’est la pauvreté, les gens n’ont pas l’argent, les gens ne sont pas non plus sensibilisés qu’ils doivent fréquenter les services, voilà les deux choses qui vont en sorte qu’avant la gratuité égal après la gratuité mais pendant la gratuité, il y a eu augmentation du taux des services mais avant comme après, il y a réduction e taux de fréquentation des services, cette réduction-là est due à quoi ? Il fallait d’abord étudier ça. On a donné les soins aux malades, on a sauvé des vies pendant cette période-là, on a donné les soins gratuits mais après cette période, c’est vraiment chaotique donc il y a de l’impact négatif où il fallait étudier les défis et essayer de répondre à ces défis, il n’y a pas eu des projets intégrateurs parce qu’il fallait faire des projets intégrateurs, des projets où après la gratuité, comment la population va faire face à l’accessibilité aux soins

**P12: IA_03_DPS_ANALYSTE.rtf - 12:1 [Répondant : De manière général..] (2:2) (Super)**

Codes: [Répondant_Responsabilité_habituelles]

No memos

Répondant : De manière générale, je suis l’analyste chargé des ressources humaines à la division provinciale de la santé donc dans le bureau gestion des ressources, il y a quatre cellules. Il y a la cellule du partenariat, la cellule finances, la cellule gestions des matériels et la cellule gestion des ressources humaines donc mi je suis AG chargé des ressources humaines. Quand on parle des ressources humaines, je pense que vous connaissez nous sommes impliqués dans le recrutement du personnel, la gestion de la carrière du personnel

**P12: IA_03_DPS_ANALYSTE.rtf - 12:2 [Répondant : ce que je dis tant..] (4:4) (Super)**

Codes: [Répondant_Responsabilité_pdt MVE]

No memos

Répondant : ce que je dis tantôt dans le cadre de la riposte, ce que quand l’épidémie a commencé, nous n’étions pas impliqués ce n’est qu’en décembre lors du passage du Ministre Oly Ilunga, il nous réunit ici dans la grande salle et le Ministre a dit que l’épidémie tel que gère ce n’est pas bien gérée, il voudrait responsabiliser la DPS et ils ont créé des commissions et je me suis retrouvé comme président de la commission ressources humaines. Une commission qui n’a pas durée, on a fait trois à quatre mois et après on a supprimé cette commission, on dit que non peut-être ça ne le valait pas la peine, voilà ce que nous avions joué comme rôle pendant quelques mois dans la gestion de la riposte.

**P12: IA_03_DPS_ANALYSTE.rtf - 12:3 [Répondant : Vraiment j’avais u..] (6:6) (Super)**

Codes: [Répondant_Responsabilité_pdt MVE]

No memos

Répondant : Vraiment j’avais un grand rôle, au début-là je peux vous surprendre quand la riposte a commencé le recrutement se faisait par n’importe qui et à tel point que la sous coordination se retrouvait avec un effectif qu’on ne maitrisait pas, le nombre n’était même pas connu, alors la mission qu’on nous a confié, c’était d’abord d’identifier les prestataires et chercher à comprendre même le profil et qui les a recruté et c’est ce que nous avons fait et cela nous a pris plus d’une semaine et nous avons identifié plus de 637 personnes pour la sous coordination de Goma mais la question maintenant de savoir qui les a recruté, il y a un flou donc les choses n’étaient pas structurées tantôt c’est le président de la commission qui recrute, tantôt c’est le coordonnateur qui recrute, curieusement il y a des gens qui vous disent que moi je suis venu de moi-même, personne l’a recruté mais il est là donc c’était vraiment un désordre. Il fallait d’abord recenser et nous avons fini le recensement et nous avons présenté les résultats au cordon, l’expert qui était venu de Kinshasa, le Docteur Gaston Kapenda, nous lui avons présenté la situation et au vu de la situation, il fallait voir maintenant qui retenir suivant le profil, qui écartait et maitrisait aussi les effectif par commission et aussi arrêtait ce mouvement de recrutement anarchique, ce qui a été fait ;

**P12: IA_03_DPS_ANALYSTE.rtf - 12:4 [Répondant : Vous allez peut-êt..] (10:10) (Super)**

Codes: [Ressources_humaines_Disponibilité et perturbations]

No memos

Répondant : Vous allez peut-être le recadrer si je répondais autrement. Ce qui est vrai est qu’avec la riposte, ça déstabilisait le système, avant de penser aux structure, je commence par chez nous au niveau de la DPS. Au niveau de la DPS, nous avons un bureau qu’on appelle bureau SNIS, vous allez voir le point focal, le chargé de l’épidémiologie, il était parti et jusqu’aujourd’hui il est parti vers Mangina alors que c’est lui le point focal de toutes les maladies épidémiologiques. Quand vous allez déplacer un point focal qui va s’occupé de la riposte oubliant tous les cas de rougeole et d’autres épidémies, c’est déjà un déséquilibre. Non il est parti seul mais au niveau intermédiaire si vous allez chez Pronanut, il y a des gens qui sont partis même chez nous dans le service de l’hygiène, des gens qui sont vraiment partis jusqu’aujourd’hui pourquoi ? parce qu’il y a aussi l’aspect pecunia ; avec un taux important, Ebola paye mieux et tout le monde avait tendance de partir maintenant au niveau des structures, des zones de santé, ça aussi il y a eu de problème, il y a eu même un médecin chef de zone qui quitte sa zone de santé, on pouvait comprendre si un médecin chef de zone qui s’implique dans la riposte dans sa zone de santé parce que c’est dans son territoire, dans son rayon d’action mais un médecin chef de zone qui a abandonné sa zone de santé, le médecin de la zone de santé de KAMANGO et il est parti, ça bientôt deux ans parce qu’il est dans la riposte et il fallait chercher quelqu’un parce que quand il est parti la division n’était pas au courant, il dirigeait sa zone de santé à distance, le médecin chef de zone part, l’infirmier superviseur part, l’animateur communautaire part et comprenez un peu le système, la zone de santé de KAMANGO était déstabilisée. Quand nous entrons même dans la sécurité transfusionnelle pareille chose, le chef de centre de transfusion sanguine de Beni, il part o la riposte, le problème pour c’est quoi, la personne quitte son rayon et il va prester ailleurs, quelqu’un qui va quitter Beni pour aller presté à Butembo et il abandonne même le service de sécurité transfusionnel, il fallait réfléchir qui mettre à la place. Vous allez à Mabalako, c’est même catastrophique, un médecin directeur d’un hôpital de référence qui abandonne l’hôpital et qui va même dans une autre province vers Mambasa et jusqu’aujourd’hui et quand vous allez à Beni n’en parlons même pas, eux-mêmes ont créé un système, ce qu’ils appellent les médecins de remplacement et cela n’est pas même pas dans l’administration parce que tous étaient partis dans la riposte, ils ont recruté eux-mêmes alors qu’ils n’avaient pas la compétence de recruté, ils ont recruté des infirmiers de remplacement, des médecins de remplacement. En tout cas cela a vraiment déstabilisé le système parce qu’en un moment donné la division était obligé de demander à ces gens de faire le choix s’il faut rentrer dans le système s’il faut partir et ils avaient jugé de ne pas rentrer dans le système et ils disent qu’ils ont la couverture de la hiérarchie et beaucoup d’arguments mais ils sont partis.

**P12: IA_03_DPS_ANALYSTE.rtf - 12:5 [Répondant : ferme comme tel no..] (12:12) (Super)**

Codes: [Structure-Service_Fermeture_pdt MVE]

No memos

Répondant : ferme comme tel non, mais si vous y allez, vous sentez que l’offre n’est pas de qualité. Ça joue en tous cas ça déstabilisait le système. Je peux vous dire vous arrivez à Mabalako là où il y a trois médecins, vous trouvez que tous sont partis et ils ont recruté eux-mêmes des médecins, de quelle formation ? Malheureusement nous aussi nous ne faisons pas de supervision pour évaluer la qualité des soins, on ne sait pas comparer mais la situation avant et la situation pendant la riposte. Mais je sais à un moment donné, Médecin sans frontière avait besoin de 15 médecins et 45 infirmiers, j’étais associé à ça, juste quand il voulait installer le centre de traitement vers l’aéroport et le médecin directeur de l’hôpital était clair, il dit mais on me demande de tous les médecins, 15 et 45 infirmiers, il dit non et là nous avons assisté à un problème parce que tous ces médecins voulaient partir parce que là il y a l’argent et ils voudraient partir pour bénéficier de taux d’Ebola et de la prime de risque, vous voyez un peu oubliant que l’hôpital est dans un projet de gratuite et qu’il y avait aussi afflux des malades donc il fallait faire un choix, soit vous partez, on vous remplace soit vous restez

**P12: IA_03_DPS_ANALYSTE.rtf - 12:6 [Répondant : Bon, les condition..] (14:14) (Super)**

Codes: [Ressources_humaines_conditions_travail]

No memos

Répondant : Bon, les conditions de travail, je ne sais pas ce que vous voulais comme réponse

**P12: IA_03_DPS_ANALYSTE.rtf - 12:7 [Répondant : Bon ça varie d’une..] (16:16) (Super)**

Codes: [Ressources humaines_Motivation_personnel]

No memos

Répondant : Bon ça varie d’une structure à une autre parce que présentement ceux qui sont restés par exemple à l’hôpital provincial, ils vont vous dire qu’avec le financement de l’ULB coopération ils sont bien payés, il y en a ceux qui sont dans les 1000 dollars, c’est quand même beaucoup et si vous arrivez à l’hôpital de Beni avec ceux qui sont restés, avec l’intervention di PDSS, en tout cas ils étaient bien parce qu’il y a des gens qui vous arrivent même dans le deux mille et quelques dollars mais bien que c’est pour une durée éphémère mais il y a certains endroits ceux qui étaient restés, étaient presque des aigris, ils vivaient difficilement comme à Lubero par exemple, c’était une structure où un moment il n’y avait personne, tout le monde était presque parti et il n’y avait pas des malades et comme c’était une structure qui n’était pas encore subventionnée, les recettes ont baissé et les gens souffraient et comme je dis c’est d’une structure à une autre et si je prends mon quartier à IMBI là-bas parce qu’à un moment donné comme il, y avait un cas suspect d’Ebola dans la structure, il n’y avait aucun malade parce que tout le monde avait peur de fréquenter la structure et vous comprenez qu’ils sont souffert pendant quelques mois du manque des recettes

**P12: IA_03_DPS_ANALYSTE.rtf - 12:8 [Répondant : Il y a eu aussi de..] (18:18) (Super)**

Codes: [Ressources_humaines_Disponibilité et perturbations]

No memos

Répondant : Il y a eu aussi de départ dans le secteur privé, je vous ai donné l’exemple de MSF quand il avait besoin de 45 personnes bien qu’on n’a pas su les prendre chez nous à l’hôpital provincial, ça était compensé par les privés

**P12: IA_03_DPS_ANALYSTE.rtf - 12:9 [Répondant : Oui avant la décla..] (20:20) (Super)**

Codes: [Ressources_humaines_situation_grève]

No memos

Répondant : Oui avant la déclaration de l’épidémie, il y avait la grève et cette grève qui a été à la base de cette situation de l’épidémie. Il y a une grande partie, tout a commencé je crois dans la zone de santé de Mabalako, ils étaient en grève parce qu’ils réclamaient les primes de risque, les problèmes des omissions de noms et la grève actuelle, ils disent que c’est une grève administrative, on travaille mais on ne transmet pas le rapport à la hiérarchie, ce qui fait que quand cette épidémie a commencé comme les structures ne transmettaient pas même le rapport MAPEPI, vous comprenez un peu cette histoire avait bien commencé un peu bien avant. Le médecin chef de zone a découvert en retard quand les gens lui ont dit que les gens sont en train de mourir des maladies, je crois c’était deux ou trois mois après, c’est pour cela que ça pris cet ampleur-là, c’est vrai qu’il y a eu grève, une grève administrative des infirmiers qui réclamaient la majoration des primes et la mécanisation de tous les infirmiers et puis ils sollicitaient le repaiement des primes détournés, mais la grève a déjà pris fin.

**P12: IA_03_DPS_ANALYSTE.rtf - 12:10 [Répondant : Je dois vérifier p..] (22:22) (Super)**

Codes: [Ressources_humaines_situation_grève]

No memos

Répondant : Je dois vérifier pour vous donner de dates précises

**P12: IA_03_DPS_ANALYSTE.rtf - 12:11 [Répondant : C’est certains ser..] (24:24) (Super)**

Codes: [Gratuité_Structures-Services_concernés]

No memos

Répondant : C’est certains services mais je ne maitrise pas grand-chose, peut-être mon collègue pourra vous donner tous les détails.

**P12: IA_03_DPS_ANALYSTE.rtf - 12:12 [Répondant : Ce que je peux ajo..] (26:26) (Super)**

Codes: [Commentaire-Recommandation_Répondant]

No memos

Répondant : Ce que je peux ajouter je suis en train de voir la gestion des ressources humaines après la riposte parce que ceux-là qui sont partis vont chercher à revenir et je sais qu’ils vont rentrer et la réintégration, comme elle va se faire, c’est un peu flou parce que le hiérarchie ne vous a pas aidé étant donné que les textes sont très clairs, quand la personne est partie, il l’a déclaré comme déserteur et il est remplacé, ça n’a pas été fait cela a été fait avec complaisance mais maintenant que ça prend fin quand ils vont rentrer, je ne sais pas comment ça va se faire, est-ce qu’ils viendront remplacer ceux qui sont là, c’est un peu flou.

**P13: IA_03_DPS_CB1.rtf - 13:1 [Répondant : Ici, c’est le Bure..] (2:2) (Super)**

Codes: [Répondant_Responsabilité_habituelles]

No memos

Répondant : Ici, c’est le Bureau Inspection et Contrôle et alors le Bureau Inspection et Contrôle a pour mission d’effectuer des audits en interne au niveau de la DPS et il doit s’assurer que les normes sont bien d’application au sein de la DPS d’abord mais aussi dans les zones de santé. Les normes en matière de gestion des ressources, des normes en matière de la qualité des soins, des normes en matière de la qualité des médicaments sur toute l’étendue de la province et j’occupe la fonction de chef de bureau donc j’essaye de coordonner ces activités au sein du bureau donc je ne travaille pas seul, nous travaillons en équipe. Le bureau est constitué d’une équipe multidisciplinaire donc dans le bureau nous avons un pharmacien, un médecin, un administrateur gestionnaire, nous avons un laborantin et nous avons un infirmier

**P13: IA_03_DPS_CB1.rtf - 13:2 [Répondant : Pendant l’épidémie..] (4:4) (Super)**

Codes: [Répondant_Responsabilité_pdt MVE]

No memos

Répondant : Pendant l’épidémie, je peux dire que le bureau n’a pas joué beaucoup des rôles dans la gestion de l’épidémie, pourquoi ? Parce que je veux qu’il y avait une administration parallèle, au sein de la DPS, le système de la gestion n’était pas le système de santé tel que défini dans le texte, c’était vraiment une autre organisation et donc le Bureau Inspection et Contrôle n’a pas joué un grand rôle sauf au début où on a participé au déploiement des médicaments dans les formations sanitaires parce qu’au début quand l’épidémie a commencé dans une zone de santé Mabalako, il y avait deux autres zones de santé, Beni et Uwisha pour lesquelles il a fallu leurs doter des médicaments pour assurer la prévention de ceux qui viendront de Mabalako et au début je crois que c’est juste ça qu’on a fait. Le bureau a assuré, il y a eu la gratuité des soins dans les zones de santé et dans la gratuité des soins le bureau comme bureau d’inspection et contrôle a effectué quelques missions pour voir si les directives par rapport à cette gratuité des soins étaient respectés dans le cadre du PDSS

**P13: IA_03_DPS_CB1.rtf - 13:3 [Répondant : Non pas tel que dé..] (6:6) (Super)**

Codes: [Gouvernance_soins santé]

No memos

Répondant : Non pas tel que défini, je dis qu’il y a eu une administration parallèle et par administration parallèle, je veux dire quoi ? Vous voyez au niveau de la DPS, l’action sanitaire est pilotée parce que nous appelons le CPP et le CPP est dirigé par le gouverneur de province et dès fois il délègue le ministre, c’est ça l’organe suprême qui gère l’action sanitaire en province. Le CPP a des commissions et au Nord-Kivu, nous en avons six, donc nous appelons cela des groupes de travail qui sont des organes techniques du CPP et au Nord-Kivu, nous en avons six, nous avons groupe de travail gouvernance, groupe de travail médicaments, groupe de travail prestations et encadrement, groupe de travail infos sanitaire, groupe de travail financement et un groupe de travail ressources humaines. En principe comme Ebola n’était pas la première maladie que nous avons gère, il y a des épidémies que la DPS gère et quand il y a des épidémies dans chaque groupe de travail, il y a des thématiques par rapport à ça. Par exemple on a géré l’épidémie de choléra ; il y a le groupe de travail infos sanitaire qui a un rôle a joué, il y a le groupe de travail médicaments qui a un rôle à jouer pour s’assurer de tout ce qui est logistique et médicaments, il y a le groupe de travail prestations qui a un rôle à jouer et c’est de cette façon-là qu’on nous avons pensé que les choses allient marcher mais seulement ça n’a pas été le cas parce que parallèlement à ces organes qui existent bien sûr que c’était la première fois que nous avions eu cette épidémie d’Ebola et on n’avait pas assez d’expertise dans la gestion d’Ebola donc on avait des compétences dans la gestion des épidémies au niveau de la DPS mais pour ce qui est de l’épidémie d’Ebola, on n’avait pas des compétences comme telle. Mais ces compétences n’ont pas été associé comme ça devaient se passer

**P13: IA_03_DPS_CB1.rtf - 13:4 [Répondant : Bon pendant la pér..] (8:8) (Super)**

Codes: [Répondant_Responsabilité_pdt MVE]

No memos

Répondant : Bon pendant la période d’Ebola comme je vous ai dit le rôle puisque quand il y a eu Ebola le rôle devait rester le même mais comme je vous ai dit dans la gestion de l’épidémie était ailleurs, ce n’était pas à la DPS et donc nous, on n’était même pas sensé à parler

**P13: IA_03_DPS_CB1.rtf - 13:5 [Répondant : On nous a rapporté..] (10:10) (Super)**

Codes: [Structures-Services_fonctionnement]

No memos

Répondant : On nous a rapporté et aussi nous avons vécu, nous avons vécu certaines situations pas plus tard que la semaine passée et je reviens d’une mission d’inspection et contrôle dans une centre de santé de référence dans laquelle tous les médecins ont abandonné la formation sanitaire et sont tous devenus consultant chez l’OMS et d’autres ont été recruté dans d’autres organismes pour travailler en dehors de leurs zones de santé dans la riposte contre Ebola et donc il y a eu changement dans certaines formations sanitaires où il n’y avait pas assez des médecins et le peu des ressources qui étaient dans la province ont été recruté en dehors de leur zone de santé mais il faut dire que dans certaines formations sanitaires où il n’y avait pas d’Ebola, il y a eu quelques soucis de fonctionnement mais dans les aires de santé ou dans les zones de santé où il y a eu Ebola, bon là les prestations étaient de qualité parce que tous les médecins qui étaient dans les aires de santé et dans les formations sanitaires où il n’y avait pas Ebola sont allés travailler là où il y avait Ebola, dans les commissions de lutte contre Ebola

**P13: IA_03_DPS_CB1.rtf - 13:6 [Répondant : La consultation ça..] (12:12) (Super)**

Codes: [Structures-Services_fonctionnement]

No memos

Répondant : La consultation ça va parce que l’Ebola, c’était le curatif, il y avait le curatif, il y avait la surveillance épidémiologique, il y avait la vaccination et il y avait d’autres activités. Par rapport aux activités, prise en charge dans le cadre d’Ebola, c’était bien organisée et l) je vois par exemple la vaccination contre l’Ebola mais la vaccination contre les autres maladies, là il y a des soucis

**P13: IA_03_DPS_CB1.rtf - 13:7 [Répondant : D’abord il y a eu ..] (14:14) (Super)**

Codes: [Structure-Service_Utilisation_population]

No memos

Répondant : D’abord il y a eu un problème au niveau de la communauté parce que la communauté n’avait plus confiance au service de santé même jusqu’aujourd’hui la confiance n’a pas encore repris, je ne sais pas ce qu’on va faire pour que la communauté puisse refaire confiance aux services de santé que nous leurs offrons parce que dans les zones de santé touchées la population a refusé la distribution des kits familiaux donc il y a des médicaments qui sont périmés et que la population a dit que c’est dans vos kits là qu’il y a Ebola et nous n’allons pas les prendre. Par rapport à la vaccination de routine, c’est la même chose aussi la couverture OBC a chuté

**P13: IA_03_DPS_CB1.rtf - 13:8 [Répondant : Il y a les deux, l..] (16:16) (Super)**

Codes: [Service_vaccination_Fonctionnement]

No memos

Répondant : Il y a les deux, la personne qui devait prendre le vaccin, il refuse mais au même moment la personne qui devait sensibiliser pour l’amener à la vaccination n’a pas le temps matériel pour le faire, il est occupé à faire autre chose. Si je prends par exemple les équipes cadre des zones, normalement pendant l’épidémie ils devaient se réunir les plus régulièrement possible, si l’organisation était assurée comme nous organisons les choses, les comités des gestions des zones devaient se réunir les plus régulièrement possible, s’ils se réunissent une fois par mois alors pendant la période de l’épidémie, ils devaient se réunir deux à trois fois par mois et ce que nous avons constaté est que les équipes cadres ne se réunissent plus, vous arrivent dans une zone de santé et vous êtes en train d’évaluer la gratuité et dans la gratuité, il y a un contrat que les ECZ ont signé avec l’EIP face et on a constaté dans cette évaluation durant au moins une année, il y a des zones de santé qui ne se sont jamais réunies en comité de gestion, à l’équipe cadre et plus encore les encadrement des formations sanitaire s’était fait seulement pour la surveillance de l’Ebola et le reste, ce n’était plus vraiment la préoccupation des ECZ.

**P13: IA_03_DPS_CB1.rtf - 13:9 [Répondant : L’épidémie a eu un..] (58:58) (Super)**

Codes: [SNIS_fonctionnement_Decrire_pdt MVE]

No memos

Répondant : L’épidémie a eu un effet sur ça dans quel sens. Moi je suis ici au niveau de la DPS et comme l’épidémie paie mieux, il y a certains cadres du bureau information sanitaire qui sont partis dans la surveillance épidémiologique et dans le bureau, il n’est resté que le chef de bureau, les autres étaient déjà partis dans l’Ebola et donc vous comprenez que si dans un bureau, il n’y a que le chef de bureau qui d’ailleurs était sollicité par ici par-là donc sur le plan système, il y a eu un impact et qui nous poursuit jusqu’aujourd’hui donc il y a eu un impact.

**P13: IA_03_DPS_CB1.rtf - 13:10 [Répondant : La production de d..] (60:60) (Super)**

Codes: [SNIS_fonctionnement_Decrire_pdt MVE]

No memos

Répondant : La production de données pas de bonne qualité puisqu’on n’a pas eu pratiquement de faire l’analyse des données et faire le feedback aux zones de santé et les zones de santé ont produit leurs rapports un peu en retard. On a corrigé cela dans les zones de santé en instituant un système de performance et on a mis comme indicateur la promptitude pour que ces membres des équipes cadres soient payés par la riposte et ça augmentait un tout petit peu mais avant cela il y avait un problème de promptitude qui était criant.

**P13: IA_03_DPS_CB1.rtf - 13:11 [Répondant : La première chose,..] (52:54) (Super)**

Codes: [Souhait_Changement_gestion_futures_épidémies]

No memos

Répondant : La première chose, je suggérerais qu’il ait d’abord une évaluation qu’on comprenne qu’est-ce qu’il y a et ce qu’on doit apporter sur tout le plan, sur le plan ressources et dans les ressources, je vois les hommes, les infrastructures, je vois les équipements donc qu’on ne gère pas les épidémies dans la précipitation bien sûr que l’épidémie est une urgence mais l’urgence n’est pas une précipitation et c’est qui a caractérisé l’épidémie, c’était vraiment la précipitation et c’est cette précipitation qui a eu des conséquences. Je donnerai comme conseil de gérer l’urgence comme urgence et non se précipitait à trouver des solutions sans analyse préalable et en faisant l’analyse préalable on va voir l’existant pour évaluer qu’est-ce qu’il faut encore apporter donc nous avons besoins de telles ressources humaines et quelle est la disponibilité qu’il en a et qu’est-ce que nous pouvons encore apporter et dans la disponibilité je vois on peut avoir des hommes mais qui n’ont pas la connaissance voulue et là on évaluerait si les hommes sont là, les capacités pour faire le travail avant de réfléchir à la consultante, ça peut aussi faire que les hommes sont là mais ils n’ont pas la capacité mais ils n’apporteraient rien et dans ce cas alors on peut recourir à la consultance.

De deux, ce que je conseillerais il faut évaluer l’organisation des institutions qui sont mal organisées même s’il y a l’urgence, vous voulez vous appuyer sur ces institutions qui sont mal organisées avant de leur apporter l’assistance voulue alors dans ce cas il faut d’abord évaluer l’organisation existante et voir s’il faut renforcer cette organisation existante ou s’il faut créer quelque chose d’autres parce qu’on ne sait pas travailler avec l’organisation qui existe

De trois, la façon de gérer les apports parce qu’ici on a constaté qu’il y avait les mêmes choses qui se faisaient pas deux ou trois ou quatre partenaires dans une aire de santé alors qu’ailleurs il n’y avait absolument rien qui était fait donc ce serait de la meilleur de cas de coordonner les partenaires, les apports des partenaires puisqu’il y en a même ce qui se sont battu, sur terrain les partenaires se battaient et donc on a compris qu’il y avait des difficultés à ce niveau-là ; si on peut coordonner cet aspect-là par rapport au financement, on coordonne, on sait qu’un tel est à tel endroit et ici voici les besoins et toi tu dois faire un, deux, trois, quatre choses en dehors de l’aire de santé qui est déjà couverte

**P13: IA_03_DPS_CB1.rtf - 13:12 [Répondant : Planification des ..] (50:50) (Super)**

Codes: [Gouvernance_soins santé]

No memos

Répondant : Planification des activités, sur le rapportage dans le système. Oui, parce qu’au niveau de la DPS, la planification des activités de la riposte, il y a eu plusieurs commissions qui ont été mise en place et ce sont ces commissions qui travaillent mais par rapport à notre plan ici, plan d’action de la DPS, ça eu un impact, par exemple dans une zone de santé où on avait planifié, on n’a pas pu réaliser certaines informations mais il y a par exemple le monitorage, le MAA qui était prévu dans les zones de santé, on a pas pu réaliser parce que ce n’était pas un bon signe, à part ça il y a nos activités régaliennes qu’on n’a pas pu réaliser qui se figurait dans notre PAO, au début on n’a pas pu mobiliser tous les médecins chefs de zones pour les revus semestriels d’activités mais on sait ressaisi un mais au début c’était un peu difficile

**P13: IA_03_DPS_CB1.rtf - 13:13 [Répondant : Oui, ces fonctions..] (48:48) (Super)**

Codes: [Gouvernance_soins santé]

No memos

Répondant : Oui, ces fonctions ont été affectées par la riposte, comme je l’avais dit avant là où la décision devrait être prise, c’est là où la décision ne se prenait pas. Je dis au départ que c’est le CPP qui pilote l’action sanitaire et donc quand bien même on créerait cette coordination de riposte, la coordination de riposte serait un groupe de travail technique qui serait créer parce qu’il y a quelque chose d’anormale qui demandait à ce que le groupe de travail qui existe face au travail parce qu’il y a d’autres aspects pour lesquels le groupe de travail qui existait n’avait pas peut-être des capacités, cette coordination serait quelque chose de technique qui appuierait le CPP et toutes les décisions se prendraient à ce niveau-là mais ça n’a pas le cas, les décisions se prenaient dans cette coordination qui n’était même pas à Goma, la coordination était à Butembo et à Beni pas ici à Goma et après on a compris cette le chef-lieu, c’est à Goma et après on a ramenait cette coordination à Goma et donc à ce niveau-là vous comprenez que la personne attitrée qui devait prendre une décision, ce n’est pas seule-là qui prenait des décisions mais les décisions étaient prise ailleurs, je veux vous donner un exemple si la personne qui prenait des décisions était la personne qui devait prendre les décisions peut-être on capitaliserait les quelques moyens de location des véhicules qui ont utilisés parce que à ce niveau-là celui-là dirait au lieu d’aller loué les véhicule des particuliers achètent des véhicules pour les zones de santé qui resterait après l’épidémie et ça développerait le système mais comme la décision se prenait pas un lieu et où la personne qui prenait la décision n’était pas celle qui devait prendre des décisions, elle voyait juste cet aspect de l’épidémie, elle ne voyait pas le système donc à ce niveau déjà la coordination, donc le mécanisme de la gouvernance n’était pas respecté. Par rapport à la population, il y a eu quelques soucis, bien sûr qu’il y a des choses qui ont été corrigé progressivement, la province du Nord-Kivu est parmi les provinces mères, ce sont des provinces qui ont commencé sur le plan sanitaire qui avait un bon système sur le plan organisationnel de santé et donc dans les formations sanitaires, il y avait des médecins, il y avait tout mais début toutes les personnes qui travaillent dans la riposte, c’était des personnes, je ne sais pas dire importe, c’était des consultants mais a un moment donné comme nous sommes dans la coordination, ces consultants étaient recruté sur quelle base parce qu’il y avait des personnes qui ne comprenaient pas le système donc il ne savait pas, comment est-ce que le système est organisé, ils ne pouvaient pas savoir que quand vous arrivent au niveau de la zone de santé, il y a un médecin chef de zone et il y a une équipe cadre mais ils ne savaient pas ça. Ça était mal vu par la population

**P13: IA_03_DPS_CB1.rtf - 13:14 [Répondant : Bon, au fait il fa..] (46:46) (Super)**

Codes: [Médicaments_disponibilité_fosa]

No memos

Répondant : Bon, au fait il faut dire que dans les zones de santé où le système de santé était bien organisé, où les choses marchées déjà bien avant l’épidémie, ils n’ont pas eu assez de souci par rapport aux médicaments mais dans les zones de santé où le système n’était pas bien placé, ils ont connu des problèmes. Et les zones de santé qui étaient bien organisés, ce sont des zones de santé appuyées par l’Union Européenne et cela fait déjà plus des années, eux avaient déjà des systèmes plus solides, le système d’approvisionnement très solide et il faisait leur précommande avant, ils avaient des médicaments et leurs pharmacies étaient bien organisées et les formations sanitaires passent leurs commandes sans problèmes, là ils ont pas connu des problèmes des médicaments, même s’ils ont connu des ruptures de quelques molécules et ce n’était pas vraiment très criant mais les zones de santé comme Katwa, Butembo, Kalunguta et les autres qui n’avaient pas leur système bien organisé, ils ont vraiment connu des sérieux problèmes de distribution des médicaments et certaines formations sanitaires allaient même acheter les médicaments sur les marchés locaux avec la qualité qui n’étaient pas bonne par exemple Kiondo, Musenene, Vowoyi

**P13: IA_03_DPS_CB1.rtf - 13:15 [Répondant : Au niveau de la DP..] (44:44) (Super)**

Codes: [Gratuité_Existence_plan_désengagement]

No memos

Répondant : Au niveau de la DPS, il y a un plan qui a été discuté par les organes vraiment structurés de la division, vraiment je n’ai pas participé à une telle réunion dans laquelle on discute de tels éléments, je sais que je ne peux pas être dans toutes les réunions. Peut-être quand vous aller confronter avec la réalité du terrain, vous pourriez tomber dans certaines informations mais pour ce qui le concerne je n’ai pas participé dans une réunion où on discutait de ce plan

**P13: IA_03_DPS_CB1.rtf - 13:16 [Répondant : Bon, plusieurs for..] (42:42) (Super)**

Codes: [Gratuité_Conséquences_utilisation_services]

No memos

Répondant : Bon, plusieurs formations sanitaires privées ont fermé leurs portes et je crois que c’était sanitaire parce que le cas d’Ebola qu’on a notifié dans le majeur des cas venait de ces formations sanitaires. Au fait, quand les malades se méfiaient des structures publics parce qu’on sait que c’est là où on injectait la maladie aux gens, ils fuyaient dans les formations sanitaires privés où la qualité n’est pas bonne du tout, alors ces formations sanitaires ont contaminés beaucoup des malades mais beaucoup des prestataires comme vous êtes dans l’étude si vous analysez les statistiques des médecins qui sont décédés et des infirmiers et des laborantins qui sont décédés, ce sont des personnes qui travaillés dans des formations sanitaires privées. Si cette stratégie nous a permis de fermer tant soit peu certaines structures privées, personnellement je prendrai ça dans le sens positif ; ce n’est pas toutes les formations sanitaires privées qui ont fermés leurs portes, il y a ceux qui ont continué à fonctionnées et les malades paient régulièrement parce que comme je vous disais il y avait l’engouement dans les structures publiques et il y a des personnes ne pouvaient pas faire le fil d’attente et préféraient chez les privés bien organisés et pas dans les petits ligablos, ils ont fermés d’eux-mêmes

**P13: IA_03_DPS_CB1.rtf - 13:17 [Répondant : Il faut dire que c..] (40:40) (Super)**

Codes: [Gratuité_Conséquences_motivation_prestataire]

No memos

Répondant : Il faut dire que ce n’était pas vraiment la gratuité des soins mais plutôt les soins étaient subventionnés donc les soins étaient gratuits pour les malades mais pour les prestataires continuent à recevoir la subvention du PDSS pour les zones de santé que je citais et la subvention de l’Union Européenne pour les zones de santé qu’elle appuie et donc il n’y a pas eu de remous au contraire c’est le prestataire qui a gagné parce que le taux de recouvrement des recettes est de 100% donc c’est le prestataire qui a gagné, le malade a gagné et aussi le prestataire a gagné

**P13: IA_03_DPS_CB1.rtf - 13:18 [Répondant : Bon dans un premie..] (38:38) (Super)**

Codes: [Gratuité_Accueil_population]

No memos

Répondant : Bon dans un premier temps, il y a eu des résistances. C’est comme je vous ai dit qu’avec l’épidémie il y a eu un peu de méfiance entre le professionnel de santé et la population alors toute action bonne soit elle, la population n’avait pas de confiance en ça et au début il y avait vraiment des méfiances, c’était au deuxième mois et comme la pauvreté commençait à prendre de l’ampleur, c’est au deuxième mois après la sensibilisation que la population est venue en masse mais au premier mois non, ils étaient en train de voir ceux qui étaient partis s’ils allaient souffrir de la maladie et quand ils ont constaté que ce n’étaient pas le cas, ils sont venus

**P13: IA_03_DPS_CB1.rtf - 13:19 [Répondant : Il n’y a pas eu d’..] (36:36) (Super)**

Codes: [Gratuité_Interruption]

No memos

Répondant : Il n’y a pas eu d’interruption de cette gratuité

**P13: IA_03_DPS_CB1.rtf - 13:20 [Répondant : d’autres conséquen..] (34:34) (Super)**

Codes: [Gratuité_Conséquences_utilisation_services]

No memos

Répondant : d’autres conséquences de la gratuité, pas tellement. A part ça je ne vois pas d’autres conséquences

**P13: IA_03_DPS_CB1.rtf - 13:21 [Répondant : Présentement dans ..] (32:32) (Super)**

Codes: [Gratuité_Evolution]

No memos

Répondant : Présentement dans le PDSS, ils ont la consultation, là aussi il faut voir le document, le contrat qui définit clairement les services qui sont ciblés. Et le contrat vous pouvait les avoir à l’EIP qui est l’interface de ces deux partenaires

**P13: IA_03_DPS_CB1.rtf - 13:22 [Répondant : La gratuité des so..] (20:20) (Super)**

Codes: [Gratuité_Structures-Services_concernés]

No memos

Répondant : La gratuité des soins, c’était dans 7 zones de santé touchées par l’épidémie d’abord, et puis les 7 zones qui étaient financées par le PDSS puis l’union européenne a emboité le pas, il y a ces zones de santé qu’il appuie. Pour les 7 du PDSS, j’ai Uwisha, Beni, Mabalako, Butembo, Katwa, Kalunguta et Masereka, c’était les 7 qui ont commencé la gratuité et juste après on a ajouté Musenene, Kiondo, Mangoreshipa, Biena et Vowovi et dans ces zones de santé, toutes les formations sanitaires intégrées étaient prise en compte

**P13: IA_03_DPS_CB1.rtf - 13:23 [Répondant : Au début, c’était ..] (22:22) (Super)**

Codes: [Gratuité_Structures-Services_concernés]

No memos

Répondant : Au début, c’était tous les services pour les sept-là et à un moment on a commencé à cibler

**P13: IA_03_DPS_CB1.rtf - 13:24 [Répondant : Ah non, pour être ..] (24:24) (Super)**

Codes: [Gratuité_Structures-Services_concernés]

No memos

Répondant : Ah non, pour être beaucoup plus concret, il faut regarder les documents

**P13: IA_03_DPS_CB1.rtf - 13:25 [Répondant : mais probablement ..] (26:26) (Super)**

Codes: [Gratuité_Partenaire d'appui]

No memos

Répondant : mais probablement sur terrain, il y a d’autres qui ont appuyé la gratuité mais pas dans le format du PDSS et de l’Union Européenne mais quand vous serez dans les zones de santé essayer de poser la question

**P13: IA_03_DPS_CB1.rtf - 13:26 [Répondant : Oui, la gratuité c..] (28:28) (Super)**

Codes: [Gratuité_Evolution]

No memos

Répondant : Oui, la gratuité continue

**P13: IA_03_DPS_CB1.rtf - 13:27 [Répondant : C’est ciblé] (30:30) (Super)**

Codes: [Gratuité_Evolution]

No memos

Répondant : C’est ciblé

**P13: IA_03_DPS_CB1.rtf - 13:28 [Répondant : D’abord la plus gr..] (18:18) (Super)**

Codes: [Structures-Services_fonctionnement]

No memos

Répondant : D’abord la plus grande raison, c’est ce que je vous ai donné, il y a eu une gestion parallèle, l’épidémie était comme une autre institution où le médecin chef de zone ne dépendait plus du chef de division mais qui était un autre chef, je ne sais pas si c’était le ministre, là ne me demandait pas si le médecin chef de zone dépendait de qui exactement et par conséquent les activités de routine du médecin chef de zone, il ne les faisait plus parce que les réunions de coordination, c’était des réunions qui se tenaient tous les jours mais dès fois tous les membres de l’équipe cadre était là dans la riposte et au bureau, on pouvait trouver une personne juste l’administrateur gestionnaire qui était resté seul et il lui était impossible de tenir des réunions. Là je suis au niveau des équipes cadres des zones de santé. Si je dois aller au niveau des formations sanitaires, les prestations sont impeccables,, il n’y a pas eu beaucoup des difficultés sauf au niveau de la qualité des soins parce que quand on a institué la gratuité des soins, le nombre des cas on les a multiplié fois 20 ou fois 50 et l’équipe est resté le même parce qu’une partie de l’équipe était dans la riposte et en multipliant les cas donc la qualité des soins a baissé un peu

**P14: IA_03_DPS_CB2.rtf - 14:1 [Répondant : Notre bureau fait ..] (2:2) (Super)**

Codes: [Répondant_Responsabilité_habituelles]

No memos

Répondant : Notre bureau fait partie de six bureaux de la DPS, c’est le bureau appui technique aux zones de santé. Notre mission, c’est d’appuyer techniquement les zones de santé sur le plan logistique et managérial. Nous avons cinq services : nous avons l’approvisionnement et la logistique médicale donc tout ce qui est médicament, c’est bureau qui s’en occupe, nous avons le suivi et évaluation, c’est un service qui est dans notre bureau, nous avons l’encadrement des zones de santé, c’est un service qui est organisé à deux pools, le pool de Goma et le pool de Butembo, nous avons l’intégration des programmes spécialisés et enfin la qualité des soins. Ce sont les services que nous sommes en train de coordonner.

**P14: IA_03_DPS_CB2.rtf - 14:2 [Répondant : Ebola n’a pas été ..] (4:4) (Super)**

Codes: [Répondant_Responsabilité_pdt MVE]

No memos

Répondant : Ebola n’a pas été géré directement au sein de la DPS, il y avait une coordination nationale qui était gérée par Kinshasa qui a mis en place des sous coordinations qui étaient aussi gérées de manière externe donc certains agents de la DPS se sont retrouvés dans telle ou telle coordination vraiment avec des démarches que nous appellerons personnels maintenant avec ce terreur-là qui a désarticulé le système, on est en train de voir comment remettre la riposte au sein de la DPS et au niveau des zones, c’était la même chose étant qu’on pouvait piquer un ou deux qui vont travailler dans la riposte et bien rémunéré mais pas bien coordonner avec les médecins chefs des zones et ça était à la base de beaucoup des problèmes

**P14: IA_03_DPS_CB2.rtf - 14:3 [Répondant : Oui, le changement..] (6:6) (Super)**

Codes: [Structures-Services_fonctionnement]

No memos

Répondant : Oui, le changement, il en a eu parce qu’il y a eu pratiquement un relâchement de beaucoup d’activités des soins de santé primaire, la vaccination, la supervision des structures, parce qu’on se concentre uniquement sur la riposte. Les médecins chefs des zones ont été pris comme des superviseurs, des coordonnateurs au niveau de leurs zones de santé et le médecin chef de zone était à tout moment dans ces activités de riposte, il pouvait avoir derrière lui deux à trois de ses infirmiers superviseurs qui vont tous se retrouver dans la coordination organisée par la riposte donc beaucoup d’activités ont souffert di non suivi

**P14: IA_03_DPS_CB2.rtf - 14:4 [Répondant : Oui, j’ai même cit..] (8:8) (Super)**

Codes: [Structures-Services_fonctionnement]

No memos

Répondant : Oui, j’ai même cité la supervision donc il y a des structures qui pouvaient faire même six mois sans être supervisées parce que les personnes clés qui devaient les faires dépendaient d’autres personnes qui n’étaient pas de la DPS qui était cette organisation parallèle qui était créé à partir du Ministère de la santé

**P14: IA_03_DPS_CB2.rtf - 14:5 [Répondant : Oui, il y a des st..] (10:10) (Super)**

Codes: [Structures-Services_fonctionnement]

No memos

Répondant : Oui, il y a des structures, des centres de santé qui ont été pillées, endommagées. Il y a de personnel de la santé qui ont été tués parce qu’ils étaient dans la riposté donc ça amène certaines structure a ne pas fonctionné, il y a des dispensaires qui ont été pillées parce qu’ils sont dépistés peut-être un cas de la maladie à virus Ebola et la dispensaire fermée, il y a eu plusieurs structure comme ça. Dans la zone de Katwa, il y a eu l’infirmier qui a été tué parce qu’il s’est impliqué. A Katwa, il y a eu plusieurs structures qui ont été endommagé, il y a eu des menaces par ici par-là de tous les prestataires qui s’engagent dans la riposte

**P14: IA_03_DPS_CB2.rtf - 14:6 [Répondant : Quand l’épidémie a..] (12:13) (Super)**

Codes: [Structure-Service_Utilisation_population]

No memos

Répondant : Quand l’épidémie a commencé, la population était réticente au début pour utiliser les services parce que la communication n’était pas bonne. Au début, ce sont des gens qu’on ne connait pas dans l’aire de santé ou dans la ville qui venaient pour communiquer et un système parallèle mais parfois les infirmiers titulaires ne comprennent pas eux-mêmes qu’est-ce qui se passe, ils apprennent à travers les médias qu’il y a une maladie à virus Ebola et il y a des équipes mobiles qui circulent avec des grands moyens et les gens ont trouvent que c’est un danger public

Et comme ils commençaient à collaborer avec les infirmiers titulaires, ils craignaient à vernir à l’hôpital ou au centre de santé de peur qu’on les dépiste, il suffisait de trouver une fièvre, c’est fini. Déjà on est marginalisé, on est orienté quelque part et cela a amené la crainte pour la population de fréquenter les centres de santé ainsi toutes les activités curatives, la CPN, la vaccination afin tous les domaines des soins de santé primaires, toutes ces activités ont été sous utilisées parce qu’ils craignaient parce qu’on a instauré la gratuité des soins pour essayer d’amener les gens malgré cela il en a ceux qui sont venus, ceux qui avaient les fièvres, la paludisme alors que quelqu’un qui pouvait venir pour faire vacciner leurs enfants, ils sont restés à la maison.

**P14: IA_03_DPS_CB2.rtf - 14:7 [Répondant : La situation a évo..] (15:15) (Super)**

Codes: [Structure-Service_Utilisation_population]

No memos

Répondant : La situation a évolué autrement, il faut suivre la courbe épidémiologique au niveau de l’aire de santé pour telle ou telle autre activité parce qu’au début il y avait cette réticence d’aller au centre de santé maintenant il y a la gratuité, le taux d’utilisation est au-delà, le taux d’utilisation qui était à 30% arrive maintenant à 90%, il y a eu même des lits qu’on a manqué au niveau des hôpitaux mais d’autres indicateurs sont restés en souffrance. Alors avec le temps, les gens se sont habitués avec cette gratuité des soins et puis l’utilisation a baissé progressivement mais malgré ça on constate qu’on a accumulé beaucoup d’enfants qui ne sont pas vaccinés. Les mamans qui viendraient à la CPN et les enfants qu’on amènerait à la CPS pour le suivi nutritionnel donc tous ces enfants, on ne pouvait plus les avoir. L’utilisation s’améliore mais les autres indicateurs sont restés vraiment en souffrance

**P14: IA_03_DPS_CB2.rtf - 14:8 [Répondant : Nous étions dans l..] (17:17) (Super)**

Codes: [Recouvrement_coûts-des-soins_avant gratuité]

No memos

Répondant : Nous étions dans le tarif forfaitaire ; les privés sont dans la tarification parâtes même dans les services publics de l’Etat, dans les services intégrés, on conseille la tarification forfaitaire pour la plupart des aires de santé parce que la plupart des zones de santé étaient appuyées par les différents partenaires dans ce cadre-là maintenant ils ont commencé la gratuité et le recouvrement partiel donc cette gratuité, on est en train de l’abandonner progressivement pour introduire un recouvrement des couts

**P14: IA_03_DPS_CB2.rtf - 14:9 [Répondant : En fait, je n’ai p..] (19:19) (Super)**

Codes: [Gratuité_Structures-Services_concernés]

No memos

Répondant : En fait, je n’ai pas des précisions sur la vraie daté parce que l’introduction de la gratuité était progressive. Quelques trois mois après le dépôt de la déclaration de l’épidémie, le 1^er^ aout 2018, les quelques mois qui suivent, il y a eu une introduction progressive pour certaines structures qui étaient appuyées, qui étaient affectées parce qu’on a eu deux grands partenaires pour la gratuité des soins. Nous avons eu la Banque Mondiale avec le PDSS et on a eu l’Union Européenne donc le Prodes. Alors ils se sont subdivisés certaines zones de santé et les autres zones de santé ont été appuyées par d’autres partenaires qui viennent appliquer la gratuité des soins

**P14: IA_03_DPS_CB2.rtf - 14:10 [Répondant : Toutes les structu..] (21:21) (Super)**

Codes: [Gratuité_Structures-Services_concernés]

No memos

Répondant : Toutes les structures intégrées au niveau de la zone de santé. Quand la zone est affectée, toutes les structures intégrées sont concernées, les structures privées non intégrées n’étaient pas prise en charge

**P14: IA_03_DPS_CB2.rtf - 14:11 [Répondant : Quand nous disons ..] (23:23) (Super)**

Codes: [Gratuité_Structures-Services_concernés]

No memos

Répondant : Quand nous disons intégrer càd la zone reconnait à cette structure les capacités à exécuter la mission pour laquelle elle a été créée parce que les structures ont des nomenclatures, il y a des dispensaires, des centres médicaux, etc. et la zone de santé l’a intégrée dans son système d’information, la zone de santé s’engage à la superviser et la former et à collecter les données, à communiquer la politique nationale

**P14: IA_03_DPS_CB2.rtf - 14:12 [Répondant : La gratuité a eu b..] (25:25) (Super)**

Codes: [Gratuité_Conséquences_utilisation_services]

No memos

Répondant : La gratuité a eu beaucoup des effets positifs parce que pendant la mission des suivis de la gratuité au sein de la zone de santé parce que pour PDSS, l’argent passait quand même par la DPS pour les missions des suivies, nous avons constaté qu’il y a eu augmentation de la fréquentation des services, nous avons constaté que les recettes avaient triple même la prime du personnel est passé du simple au triple. Le fonctionnement, le montant était plus important et ces frais de fonctionnement ont permis aux structures d’investir plus des moyens, il en a ceux qui ont construit des salles, il en a ceux qui ont équipés, ils ont achetés des échographes et d’autres les équipements médicaux, il en a ceux qui ont renforcés leurs stock en médicaments, il en a ceux qui ont carrément réhabilité la structure parce que les recettes avaient augmentés mais aussi ils ont eu à notifiés plus des cas parce qu’avant la gratuité, les gens étaient réticents à venir, après la gratuité, il y a eu plus des cas suspects qui ont été  notifiés

**P14: IA_03_DPS_CB2.rtf - 14:13 [Répondant : Donc c’est l’achat..] (27:27) (Super)**

Codes: [Gratuité_Conséquences_utilisation_services]

No memos

Répondant : Donc c’est l’achat des services, nous avons commencé par des calculs au début avec PDSS. C’est un hôpital x qui fonctionne, quels sont les recettes mensuelles pour une période, on se dit cet hôpital fait entrer 20000$ par mois, la prime du personnel, c’est autant et le fonctionnement c’est autant, pour les médicaments c’est autant donc il faut leur donner ce montant-là mais quel est leur taux de recouvrement ? c’est 70% mais si on arrive à un taux de recouvrement de 100% voici le montant qu’ils auront et comme ils ont un clés de répartition, voici chaque rubrique et comment ça se ferait mais comme il aurait une fréquentation qui ne se limiterait pas à 100%, il peut aller même à 120%, il y a eu une marge qu’on a ajouté. Alors PDSS lui amène l’enveloppe en fonction de la fréquentation qu’il y a eu et si le malade devait payer 100$ de forfait maintenant ils amènent 100$ alors que le malade si c’est lui qui paie directement, il aurait donné peut-être 50$ et je veux amener les restes après donc ils ont trouvé que les recettes ont augmenté

**P14: IA_03_DPS_CB2.rtf - 14:14 [Répondant : Oui, il y a eu des..] (29:29) (Super)**

Codes: [Gratuité_Structures-Services_concernés]

No memos

Répondant : Oui, il y a eu des bénéfices comme au niveau des malades tout comme au niveau des structures de santé. Vous comprenez pour tous ces gens-là c’est une protection financière de ces malades, tu devais payer 100$, on te dit, tu rentré et tu ne paies rien et les gens pouvaient venir se faire consultés et ne payent rien, c’était quelque chose de bon

**P14: IA_03_DPS_CB2.rtf - 14:15 [Répondant : Il y a eu des chan..] (31:31) (Super)**

Codes: [Gratuité_Interruption]

No memos

Répondant : Il y a eu des changements, au début on pouvait prendre tous les actes, 100% des actes qui sont réalisés à l’hôpital après on a réduit parce que les couts devenaient de plus en plus grand. Ils ont réduit, ils ont dit que nous la gratuité c’est pour attirer les gens et ils ont enlevé de cas de la chirurgie et d’autres cas qui ne sont pas en rapport avec la maladie à virus Ebola et cela a fait que la fréquentation puisse diminuer au niveau des structures, c’est les analyses qu’on a fait au niveau des hôpitaux de Beni, ils ont même réclamé qu’on tienne compte de 100% des actes pour amener plus des gens et comme les financements étaient limités, ils ne pouvaient pas aller dans ce sens mais aujourd’hui la gratuité continue dans la nouvelle formule au niveau des centres de santé et pour certaines zones de santé, on ne prenait pas tout à 100% donc on ne prenait pas toutes les aires de santé et si PDSS trouve qu’il y a un autre partenaire qui appuie quel que soit le montant qu’il donne, il ne prend plus pour cette structure parce que PDSS, c’est l’Etat et il y a des structures qui ont été pris par PDSS et d’autres partenaires qui mettent plus d’argent que PDDS. Dans la zone de santé de Mitwanga, il y a une partie des aires de santé appuyée par PDSS et d’autre partie appuyée par les autres partenaires. Mais il y a eu quand même des difficultés, la disponibilité des médicaments puisque la fréquentation a vraiment triple, il y a des ruptures fréquents de stock des médicaments mais après PDSS a amené suffisamment des médicaments et ils ont été surabondés par les médicaments

**P14: IA_03_DPS_CB2.rtf - 14:16 [Répondant : Oui, un autre défi..] (33:33) (Super)**

Codes: [Gratuité_Défis]

No memos

Répondant : Oui, un autre défi est que quand la structure est fréquentée à 100%n c’est que le taux d’utilisation des lits est au-delà de 100%, cela signifie qu’il y a des malades qui passent la nuit par terre et là c’était un défis parce qu’on ne pouvait pas faire un projet rapide pour augmenter l’hôpital sur base d’une situation momentanée et là la qualité n’était plus respectée quand tu dors par terre, il y a deux ou trois personnes sur le lit et nous l’avons observés deux, trois personnes sur un seul lit. Surtout les enfants pédiatriques, pour les adultes, ce n’est pas possible mais les autres là, les hommes et les femmes, il valait chercher un lit

**P14: IA_03_DPS_CB2.rtf - 14:17 [Répondant : Les mutuelles n’ét..] (35:35) (Super)**

Codes: [Recouvrement_coûts-des-soins_mutuelle]

No memos

**Répondant : Les mutuelles n’étaient pas impliquées parce que quand il y a gratuité des soins aucun recouvrement des mutuelles n’est impliqué ?**

**P14: IA_03_DPS_CB2.rtf - 14:18 [Répondant : Ils sont là et ell..] (37:37) (Super)**

Codes: [Recouvrement_coûts-des-soins_mutuelle]

No memos

Répondant : Ils sont là et elles existent jusqu’aujourd’hui

**P14: IA_03_DPS_CB2.rtf - 14:19 [Répondant : Oui, il y a eu des..] (39:39) (Super)**

Codes: [Ressources_humaines_Disponibilité et perturbations]

No memos

Répondant : Oui, il y a eu des gaps puisque dans une zone de santé il y avait tout au début. Le médecin chef de zone de Kamango se retrouvait à Kayina pendant plusieurs mois donc vous pouvez quitter votre zone de santé et vous retrouvez dans une autre aine de santé parce que c’est là où vous avez été recruté donc les infirmiers superviseurs ont quitté des zones de santé pour aller se retrouver dans d’autres zones de santé. Des médecins chefs des zones de santé ont quitté leurs zones de santé pour se retrouver dans d’autres zones de santé. Le médecin chef de zone de Kalunguta comme la sous-coordination fonctionne à Butembo avec toute sa équipe, le médecin directeur, le médecin chef de zone dedans et tous étaient dans les réunions régulièrement à Butembo et donc il y avait un sérieux problème dans la mise en œuvre en termes d’effectif et ils ont acquis des ordres de mission au niveau du ministre provincial de la santé et la DPS n’était plus impliquée. Quand on évalue le système, on trouve que ça ne colle pas et les gens qui sont là sont démotivés, ils reçoivent et la prime de la riposte et la prime locale et nous nous sommes là en train de garder les bureaux et nous sommes très chargés, on a la même prime alors qu’il y a la démotivation des personnes qui sont restées dans les bureaux et qui ne reçoivent rien de la DPS. Il y a même beaucoup d’agents de la DPS qui sont partis dans la riposte avec la même situation et jusque-là ils ne sont pas rentrés pour le bon fonctionnement de la DPS.

**P14: IA_03_DPS_CB2.rtf - 14:20 [Tous les évaluateurs qui arriv..] (41:41) (Super)**

Codes: [Ressources_humaines_solution-local_Disponibilité et perturbations]

No memos

Tous les évaluateurs qui arrivaient du niveau central, du niveau international qui arrivaient, ils passaient par la DPS et la DPS leur montrait qu’il y a une procédure qui ne renforce pas le système plutôt qui est là pour détruire le système, c’était un copier-coller de ce qui était passé en Afrique de l’ouest et le ministre national qui a endossé ça et au niveau du système, il y avait une désarticulation totale du système

**P14: IA_03_DPS_CB2.rtf - 14:21 [Répondant : La DPS a fait des ..] (43:43) (Super)**

Codes: [Ressources_humaines_solution-local_Disponibilité et perturbations]

No memos

Répondant : La DPS a fait des plaidoyers auprès de tous les services qui pouvaient arriver pour faire des évaluations parce que l’….. était géré par le chef de la DPS qui est le ministre national, c’est lui qui venait avec des injonctions et la DPS ne pouvait que faire le suivis donc aucun moyen n’est passé par la DPS en termes de ce que PDSS a mis à notre disposition pour le suivi de la gratuité mais il n’y a pas des moyens d’autres partenaires pour aller superviser dans les zones de santé et surtout que dans le recrutement des agents la DPS n’a pas été impliqué et ce n’est par hasard que Kinshasa a changé la coordination, on a détaché cela du ministère pour mettre ça à la présidence avec Muyembe pour voir comment on devait intégrer la riposte dans le système parce qu’il y avait quelque chose qui ne va pas. Il y avait des échecs, un échec vraiment du système, on était en train de tuer le système. Maintenant avec le Prof. Muyembe, les histoires commencent à revenir à la normal petit à petit mais on n’a pas encore atteint ce qu’on veut

**P14: IA_03_DPS_CB2.rtf - 14:22 [Répondant : On a informé la po..] (45:45) (Super)**

Codes: [Gratuité_Information_population]

No memos

Répondant : On a informé la population, on a affiché au niveau de chaque hôpital, le tiers payant le partenaire va payer 10$ pour la consultation et le bénéficiaire va payer zéro et à la radio et les affiches et les gens ont eu l’information le plus rapidement possible

**P14: IA_03_DPS_CB2.rtf - 14:23 [Répondant : Il y a relance des..] (47:47) (Super)**

Codes: [Gratuité_Evolution]

No memos

Répondant : Il y a relance des activités. Par rapport à la gratuité, il y a les effets positifs que nous avons eu à citer, on a oublié la formation parce que on a renforcé la capacité sur la PCI qui est un acquis vraiment pour la structure et nous avions constaté ce qui était négatif, c’est le relâchement des activités de la zone de santé donc les supervisions même si dans le contrat a fait avec la zone de santé en tout cas la zone de santé ne respectait pas toutes les clauses, il fallait superviser une fois les structures alors que vous pouvez voir une structure qui n’a pas été supervisée pendant trois mois et les activités préventives même au niveau des aires de santé, si l’IT lui-même est récupéré quelque part, c’est compliqué mais aussi la population qui craignait

**P14: IA_03_DPS_CB2.rtf - 14:24 [Répondant : La grève n’a pas c..] (49:49) (Super)**

Codes: [Ressources_humaines_situation_grève]

No memos

Répondant : La grève n’a pas continué parce qu’il y a eu négociation parce que l’épidémie avait commencé en juillet 2018 dans la zone de santé de Mangina mais comme les infirmiers étaient en grève administrative càd ils ne pouvaient pas envoyer tout ce qui est rapport à la hiérarchie y compris les MAPEPI « maladies à potentielles épidémiologiques » et là l’épidémie aurait commencé en ce moment-là et on ne l’avait pas détecté et quelqu’un qui était parti pour une supervision détecte une maladie, on parle d’une maladie étrange qui tue les gens, toute une famille décimait et qui fait appeler à la DPS, on envoie quelqu’un pour prélever les échantillons et le 1^er^ aout 2018, le résultat est positif, c’est la maladie à virus Ebola et le Ministre déclare l’épidémie et tout le monde est alerté et voilà qu’il y a la maladie à virus Ebola. On n’a pas travaillé avec ces infirmiers qui étaient en grève mais on a recruté les gens rapidement et on a importé d’autres personnes et on pensait qu’en une semaine on pouvait contenir cette zone de santé de Mabalako et surtout que ce n’était pas dans toute la zone mais dans une cité qu’on appelle Mangina. Après on a négocié avec les infirmiers, on a dit que vous voyez une maladie qui peut vous tuer aussi, qui tue la population que vous êtes sensés protégés et maintenant vous grevez pour dire que vous n’avez pas la prime de risque, tous vous n’avez pas le salaire de l’Etat, est-ce que c’est la division qui a une réponse à ça mais les doléances sont canalisées à Kinshasa, la réponse, ce n’est pas aujourd’hui ou demain et tout le pays a le même problème que vous. On a négocié avec les syndicats ici à Goma et c’est difficilement qu’on a acquis cette suspension de la grève jusqu’à ce que la situation est redevenue calme, jusqu’aujourd’hui

**P14: IA_03_DPS_CB2.rtf - 14:25 [Répondant : Oui, affirmatif, p..] (51:51) (Super)**

Codes: [SNIS_fonctionnement_Decrire_pdt MVE]

No memos

Répondant : Oui, affirmatif, puisque quand les gens sont trop pris dans la riposte en commençant par le centre de santé qui doit produire un rapport mensuel, qui doit produire les MAPPI et il y a des programmes spécialisés qui demandent aussi quelques données. Vous arrivez au niveau du BCZ où l’équipe est réduite, c’est l’équipe qui doit encoder le rapport dans le DHCS mais cette équipe, c’est IS qui devait encoder mais qui n’est pas là qui est parti ailleurs, le médecin chef de zone qui devait assurer la coordination, lui-même est attiré là où il peut recevoir plus et il est très surchargé parce qu’il y a la gestion des alertes. A la DPS, il y a même l’agent qui s’occupait de la surveillance à la DPS devrait aller à Mangina, plus d’une année à Mangina en train de travailler là-bas donc comprenez que ça affecte la complétude, la promptitude du rapport SNIS au niveau de la DPS même au niveau national parce que c’est la zone qui encode, quand la zone n’a pas encodé même le niveau central est affecté

**P14: IA_03_DPS_CB2.rtf - 14:26 [Répondant : Au début de l’épid..] (53:53) (Super)**

Codes: [SNIS_fonctionnement_Decrire_pdt MVE]

No memos

Répondant : Au début de l’épidémie, ça se limitait au niveau d’une seule zone de santé, après ça s’élargit progressivement donc la situation ne faisait que s’aggraver mais à un certain moment la situation s’est stabilisée parce qu’il fallait faire pression au niveau des zones de santé pour leur dire que vous avez oublié l’essentiel et il y a eu des améliorations mais ce n’est pas encore fini. Mais cette information sanitaire n’a pas été affectée que par Ebola mais aussi par les massacres des civils dans le territoire de Beni, dans la zone de Wicha parce que c’est la moitié des structures qui fonctionnent, à Mabalako les gens sont en train de fuir

**P14: IA_03_DPS_CB2.rtf - 14:27 [Répondant : En tout cas la gou..] (55:55) (Super)**

Codes: [Gouvernance_soins santé]

No memos

Répondant : En tout cas la gouvernance locale a été impactée complètement parce que la zone de santé n’a pas des décisions, la DPS n’avait pas des décisions parce qu’on vient avec une stratégie pour lutter contre une maladie, on vous dit écouter vous, vous ne saurez pas gérer cette maladie, c’est nous au niveau national qui allons gérer cette maladie et donc nous c’est que nous sommes en train de faire, c’est l’organisation. Je veux vous donner un exemple, ils sont à l’hôtel Karibu, c’est une équipe qui est là, nous sommes des experts mais on peut piquer un parmi vous parce qu’il y a plusieurs contexte, vous ne connaissez pas les spécificités et la provinces alors ils ont besoin juste des gens pour les guider, pour les aider et là nous comme DPS, on n’a pas eu des décisions, même le ministère provinciale n’avait pas des décisions parce que c’est le chef qui chapotait tout donc vous êtes dans une famille où le papa décide de prendre toutes les charges sur lui mais vous vous êtes un enfant vous pouvez donner un point de vu mais il vous dit non, nous avons déjà siéger et nous avons trouvé que ça doit aller comme ça et en ce cas il faut éviter l’affrontement et la coordination n’en avait pas parce qu’elle était externalisée. L’implication de la population, il en avait pas parce qu’on n’est pas passé par ces gens qui sont en contact avec cette population et cela était à la base de beaucoup des conflits y compris des conflits armés, il y a eu même des morts, il en a ceux qui sont en prison parmi les prestataires des soins. Il y a eu des menaces physiques de certaines personnes qui ont même fui la région, etc. parce que la population ne comprenait pas de quoi il s’agissait, nos organes de gestion continuaient à fonctionner mais non pas fonctionner sur base de la riposte mais ils ont fonctionné sur base du système de la mutuelle, c’est tout ce qu’on fait toujours parce que quand tu vois quelqu’un qui te dit ah mon cher moi je ne dépends pas de toit mais du niveau central, bon qu’est-ce que vous avez encore à dire par rapport à celui-là. Les données sortent du terrain pour aller quelque part et même au niveau de la province pour avoir ces données, c’est un problème. Alors on dit qu’on les laisse avec leurs affaires et nous aussi nous continuons avec nos affaires. Et curieusement c’est la population qui s’occupait des équipes de la riposte

**P14: IA_03_DPS_CB2.rtf - 14:28 [Répondant : Nous allons consei..] (57:57) (Super)**

Codes: [Souhait_Changement_gestion_futures_épidémies]

No memos

Répondant : Nous allons conseiller à ce qu’on puisse intégrer la lutte dans le système, dans le système cela ne signifie pas qu’on va prendre tous les agents pour les amener sur terrain afin de laisser tout ce qu’on est en train de faire, nécessairement on aura besoin des personnes supplémentaires donc l’équipe des experts viennent en appui mais en sachant que notre chef dans cette province, c’est telle, toujours on reste dans le système mais comme il y a beaucoup d’alerte dans les communautés, beaucoup de va et viens dans les communautés maintenant nous avons pensé que nous pouvons engager périodiquement certaines personnes qui vont signer les contrats avec les partenaires qui vont nous aider à faire ceci ou cela, le système ne peut pas refuser cela néanmoins on connait le leadership et au niveau de la zone de santé, c’est la même chose mais on vous envoie des véhicules avec des logo mais le médecin chef de zone ne connait absolument rien. Et on ne peut pas recruter pour des ressources qui sont dans la province, vous recrutez en dehors de la province. Vous amenez de Kinshasa des gens pour mobiliser mais localement il y a un type de swahili

**P14: IA_03_DPS_CB2.rtf - 14:29 [Répondant : Je pense que le gr..] (59:59) (Super)**

Codes: [Autres_Problèmes observés]

No memos

Répondant : Je pense que le gros, c’est ça. Si on n’utilise pas surtout dans la communication, les gens qui sont connus dans la communauté, c’est un échec et si on le fait prochainement la situation risque d’être très grave que ce qu’on a vécu parce que c’était la première expérience dans les provinces du Nord-Kivu et de l’Ituri mais les gens ont eu suffisamment du temps pour réfléchir et si on recrutait quelqu’un de local, c’est un médecin, il a 15$ le jour et un autre médecin généraliste parce qu’il est venu de Kinshasa ou du Rwanda ou d’un pays voisin, il a dix fois plus, il a 150$ le jours mais vous êtes ensemble et celui qui est envoyé sur terrain avec tout le risque d’être attaqué par la population, c’est ce médecin local si bien qu’il y a eu un médecin qui est venu d’ici qui a reçu un coup de machette à la tête à Butembo et on l’amène ici pour des soins médicaux donc il y a eu quelques chose qui semblait à de business, des millions de dollars qui ont été dépensé et cela n’était pas intéressant.

**P14: IA_03_DPS_CB2.rtf - 14:31 [Répondant : Il n’y a pas une é..] (65:65) (Super)**

Codes: [Service_laboratoire_fonctionnement] [Service_prise-en-charge_fonctionnement]

No memos

Répondant : Il n’y a pas une évaluation mais j’espère que ça était respecté

**P14: IA_03_DPS_CB2.rtf - 14:32 [Répondant : C’est la même chos..] (63:63) (Super)**

Codes: [Service_laboratoire_fonctionnement]

No memos

Répondant : C’est la même chose, la goute d’épaisse, ces histoires-là, on a dit il déconseillait pendant qu’on peut soigner même la maladie avec des signes

**P14: IA_03_DPS_CB2.rtf - 14:33 [Répondant : Il y a eu quand mê..] (67:67) (Super)**

Codes: [Structures-Services_fonctionnement]

No memos

Répondant : Il y a eu quand même des indicateurs qui montraient que c’était respecté parce que dans le SNIS, il y a un indicateur palu simple présumé et dans le présumé c’est le diagnostic sur base des signes ou des symptômes qu’on a vu donc on n’a pas fait l’examen de laboratoire. Il y a eu quand même un montée des cas comme ça et on a compris que parce qu’il y a eu une restriction

**P14: IA_03_DPS_CB2.rtf - 14:34 [Répondant : Non] (69:69) (Super)**

Codes: [Structures-Services_fonctionnement]

No memos

Répondant : Non

**P15: IA_01_AS_CSR_01.rtf - 15:1 [Répondant : Ce que je suis en ..] (13:13) (Super)**

Codes: [Répondant_Responsabilité_habituelles]

No memos

**Répondant** : Ce que je suis en train de faire, c’est environs dans trois volets : le premier volet, c’est que je suis appelé à apprécier la qualité des soins ensemble avec le chef de staff dans la structure ; le deuxième volet, c’est que je dois faire la coordination du secteur des infirmiers vis-à-vis de la prise en charge des malades et le troisième volet est que, je suis appelé à faire l’encadrement des élèves et étudiants stagiaires futurs infirmiers.

**P15: IA_01_AS_CSR_01.rtf - 15:2 [Répondant : Je suis le Directe..] (11:11) (Super)**

Codes: [Fosa-Institution_Présentation]

No memos

**Répondant** : Je suis le Directeur de Nursing au sein de ce Centre Hospitalier Les vainqueurs, qui est une structure confessionnelle de la CEKA 20. Cette structure est intégrée par rapport à la politique nationale de la santé et regorge une capacité d’accueil de 55 lits et un total de 28 agents dont 2 médecins, 16 Infirmiers, 3 agents de surfaces et les autres qui peuvent nous accompagner dans le travail.

**P15: IA_01_AS_CSR_01.rtf - 15:3 [Répondant : C’est vrai nous av..] (15:15) (Super)**

Codes: [Début-Progression_Epidémie MVE]

No memos

**Répondant** : C’est vrai nous avons connu l’Ebola. Nous sommes dans la Zone de santé de Beni, voisine de la zone de santé de MABALAKO, une zone de santé voisine à partir de MANGINA et qui a été ravagé par EBOLA. Après qu’il y ait des cas d’Ebola, nous avions organisé de triage après lequel nous avons séparés les malades. Les uns nous les avions nommés « les suspects d’Ebola » et les autres qui pouvaient accéder aux différents services de la structure. Tel que nous les avions séparés, depuis qu’il y a eu l’Ebola ; il y a environ un an, nous avons enregistré plus de 1000 cas suspects. Parmi ces plus de 1000 cas suspects envoyés au CTE, 18 cas ont été confirmés à Ebola. C’est pour dire que nous avons connu et vécu avec l’Ebola.

**P15: IA_01_AS_CSR_01.rtf - 15:4 [Répondant : Au début, la commu..] (17:17) (Super)**

Codes: [Considération_Ebola_population]

No memos

**Répondant** : Au début, la communauté pensait que l’Ebola était une maladie montée par les prestataires des soins. C’est ainsi qu’il y a résistance de la communauté vis-à-vis des agents de la riposte. Au fur et à mesure que la sensibilisation et l’animation ont été organisées, tout le monde a compris que l’Ebola était une maladie comme toute autre. Malheureusement, certains ont compris que c’était une maladie ordinaire après des conséquences observées. Si la maladie parvenait à attraper un membre de la famille, on pouvait consigner après que 3 autres membres de la même famille soient contaminés.

**P15: IA_01_AS_CSR_01.rtf - 15:6 [Pour la communauté il fallait ..] (19:19) (Super)**

Codes: [Agression-Menaces_Structure-Prestataire_solution]

No memos

Pour la communauté il fallait faire un recrutement local des jeunes et de leaders locaux ; ce qui n’a pas été fait et qui a provoqué des attaques et résistance.

**P15: IA_01_AS_CSR_01.rtf - 15:7 [Répondant : Oui, ces attaques ..] (19:19) (Super)**

Codes: [Agression-Menaces_Structure-Prestataire_raisons]

No memos

**Répondant** : Oui, ces attaques ont été dues selon la population, premièrement au fait que dans l’équipe de riposte on n’a pas intégré les enfants du milieu.

**P15: IA_01_AS_CSR_01.rtf - 15:8 [Répondant : Personnellement je..] (21:21) (Super)**

Codes: [Répondant_Responsabilité_pdt MVE]

No memos

**Répondant** : Personnellement je suis resté Directeur de nursing et je me suis beaucoup donné au service de triage ; c’est par là que je devais m’acquérir de la situation entant qu’encadreur des autres en matière de la qualité des soins. A part le triage, je suis le président du conseil urbain de l’ordre des infirmiers. En un certain moment nous avons travaillé avec l’UNICEF où nous avons organisé des dialogues communautaires et avec les prestataires des soins.

**P15: IA_01_AS_CSR_01.rtf - 15:9 [Répondant : En ce que je peux ..] (23:23) (Super)**

Codes: [Structure-Service_Utilisation_population]

No memos

**Répondant** : En ce que je peux dire, au début les structures intégrées, parce que c’est dans lesquelles on a observé la qualité des soins par rapport à la gratuité des soins, ces structures ont été moins utilisées parce que la population pensait que les soins gratuits, égal derrière le recrutement des gens qui pouvaient accompagner la riposte ; c’était cela les structures intégrées. La population s’est réfugiée dans les structures privées d’autant plus qu’ils en ont trop par rapport aux structures intégrées. Après la compréhension de la population, elle est venue vers les structures intégrées publiques pour abandonner les structures privées. C’est ainsi qu’il y a beaucoup de malades ici chez nous et nous manquons parfois des lits pour les accueillir ; c’est ce que nous avons observé par rapport à l’utilisation.

**P15: IA_01_AS_CSR_01.rtf - 15:10 [Répondant : Nous avons dit qu’..] (25:25) (Super)**

Codes: [Agression-Menaces_Structure-Prestataire_raisons]

No memos

**Répondant** : Nous avons dit qu’il y a eu résistance parce que l’o n’a pas tenu compte du recrutement des locaux et les leaders communautaires n’étaient intéressés par rapport à la riposte mais aussi au même moment qu’il y avait Ebola, il y avait aussi la guerre d’ADF qui nous était en train de nous tuer. Vue cette confusion là, la population a eu à tout confondre ; Et pendant ce moment-là, la population de Beni ville et Territoire a été privée des élections sous motifs qu’il ya l’Ebola et la guerre. En nous privant ces élections, les acteurs de la prise en charge de l’bola ont été mal vus. Voilà les raisons qui ont à ce que les structures soient attaquées et le personnel aussi.

**P15: IA_01_AS_CSR_01.rtf - 15:11 [Répondant : Les structures pri..] (27:27) (Super)**

Codes: [Structures-Services_fonctionnement]

No memos

**Répondant** : Les structures privées n’ont pas bénéficié des soins gratuits ; cela a fait que certaines structures ferment carrément leurs portes ; D’autres ont fermé leurs portes parce qu’elles n’ont pas respecté les règles de PCI et dont les prestataires sont décédés d’Ebola et automatiquement le reste d’agents ont eu peur et ils ont fermé. Pour ce qui est des structures intégrées, il y a eu des conflits entre les prestataires et les clients. Ces conflits sont nés du fait qu’il y a eu amélioration des conditions salariales ou bien prime des agents pendant ce temps pendant que l’on a payé des prestations dans les structures de soins. Deuxièmement dans le cadre du fonctionnement, les structures ont évolué dans le sens où la recette a augmenté parce qu’on a payé même pour les malades qui ne pouvaient pas être à mesure de payer ; Les partenaires ont appuyé également les différentes structures ; ça créé une page de conflit car donnant aux structures la possibilité d’avoir les frais de prestation pour les malades vulnérables.

**P15: IA_01_AS_CSR_01.rtf - 15:12 [Répondant : Ce conflit c’est e..] (29:29) (Super)**

Codes: [Agression-Menaces_Structure-Prestataire_raisons]

No memos

**Répondant** : Ce conflit c’est entre prestataire et client (malade). Vous imaginez une maman qui amène son enfant de deux ou trois mois, vous alertez et pus on valide au CTE. Cette maman sera séparée de son enfant. Celle-là va rentrer à la maison sans enfant et ne saura pas comment l’enfant a passé sa nuit. Si l’enfant est trop rabatteur, la maman risquera de ne voir que le corps sur le lit. Il n’est pas tué mais il est décédé de l’Ebola où au CTE (où on devrait confirmer ou infirmer l’échantillon). Alors la maman va se poser la question de savoir qui m’a envoyé là bas : c’est le prestataire, qui est un ami à moi. C’est le prestataire avec qui nous avons vécu ensemble et qui m’envoi là où on va me séparer de mon enfant. Et cela avait lieu au début où la population n’avait pas encore compris.

**P15: IA_01_AS_CSR_01.rtf - 15:13 [Répondant : Cette intégration ..] (31:31) (Super)**

Codes: [Triage_Considération_population]

No memos

**Répondant** : Cette intégration a causé des petits problèmes au début car la population n’était pas familiarisée au tri. Et nous avons compris que c’est nous même qui n’avions pas familiarisé ces malades au système de trier : savoir qui est trop malade et qui ne l’est pas, qui est contagieux et qui ne l’est pas. C’est ainsi que cela a créé des difficultés au début mais peu après la population a été familiarisée.

**P15: IA_01_AS_CSR_01.rtf - 15:14 [Répondant : La qualité des soi..] (33:33) (Super)**

Codes: [Qualité_soins_Prestataires]

No memos

**Répondant** : La qualité des soins a été modifiée dans un sens positif parce que les prestataires ont observé les règles de la PCI. Quelqu’un qui ne se munissait pas de son gant avant de prendre en charge le malade, devait comment à le porter ; Et quelqu’un qui prenait en charge un malade sans tunique comment à le porter d’abord avant de prendre en charge ce malade. Mais la qualité des soins d’une part a bâclé vu le débordement ; donc on a su maintenir les mêmes nombres de prestataires mais les partenaires n’ont pas pensé à augmenter le nombre de prestataires. C’est ainsi qu’une structure qui prenait en charge 30 accouchements ; pendant le moment de la gratuité n’a pas su prendre en charge 100 accouchements avec le même nombre de prestataires.

**P15: IA_01_AS_CSR_01.rtf - 15:15 [Répondant : Oui cela a créé un..] (35:35) (Super)**

Codes: [Qualité_soins_Prestataires]

No memos

**Répondant** : Oui cela a créé une surcharge mais pour faire bâcler aussi la qualité des soins parce que lorsque vous travaillez beaucoup, vous perdez le contrôle.

**P15: IA_01_AS_CSR_01.rtf - 15:16 [Répondant : Oui cela a perturb..] (37:37) (Super)**

Codes: [Structures-Services_fonctionnement]

No memos

**Répondant** : Oui cela a perturbé parce que même lorsque l’on a fait le recrutement, on n’a pas tenu compte de la qualité de l’agent qui devait travailler dans le CP ou bien dans le CTE. C’est pourquoi vous constaterez que même certains n’avaient pas encore fait leur perfectionnement, ont eu à passer dans CTE, et nous on pensait que cela ne pouvait pas être efficace. C’est ainsi qu’il fallait former les gens au début de la riposte et la population a compris que les gens étaient effectivement dans la formation. C’est ainsi qu’il y eu échec aussi. Et nous prestataires, o a compris que l’échec serait dû à la qualité du personnel. En tout cas au début le recrutement n’était pas bon : il y avait même des élèves et des étudiants qui ont eu à avoir accès dans l’équipe de riposte (CTE), ce qui n’était pas bon. Malheureusement, quand on a pris certains acteurs qui travaillaient des structures ordinaires pour aller dans le CTE et dans le CT, derrière eux, il fallait engager des nouveaux qui n’étaient pas familiers au travail. Le travail qui demandait des acteurs formés a été effectué par peu d’acteurs formés et d’autres non formés ; c’est ce que nous avons observé.

**P15: IA_01_AS_CSR_01.rtf - 15:17 [Répondant : Oui ça nous a conc..] (39:39) (Super)**

Codes: [Ressources_humaines_situation_grève]

No memos

**Répondant** : Oui ça nous a concernés car nous-mêmes nous étions dans la grève administrative, avant, vis-à-vis du non payement de la prime de risque, malheureusement qui n’a toujours pas été payée. Je crois cette grève doit avoir commencé au mois de février pour s’étendre vers le mois de mai 2018 om on a commencé à observer quelques cas à MANGINA  pour aller vers juillet ; en août on a suspendu parce qu’on a déclaré l’épidémie chez nous.

**P15: IA_01_AS_CSR_01.rtf - 15:18 [Répondant : Je pense que tous ..] (41:41) (Super)**

Codes: [Structures-Services_fonctionnement]

No memos

**Répondant** : Je pense que tous les services ont bien fonctionné, tel que chez nous tous les services ont fonctionné normalement.

**P15: IA_01_AS_CSR_01.rtf - 15:19 [Répondant : Le laboratoire n’a..] (43:43) (Super)**

Codes: [Service_laboratoire_fonctionnement]

No memos

**Répondant** : Le laboratoire n’a pas effectivement fonctionné normalement parce qu’on a omis certains examens de laboratoire. On devait faire beaucoup plus la clinique que le para clinique. Par exemple en cas de la malaria qui est une endémie ici chez nous, on ne devait plus faire les examens.

**P15: IA_01_AS_CSR_01.rtf - 15:20 [Répondant : C’était par instru..] (45:45) (Super)**

Codes: [Service_laboratoire_fonctionnement]

No memos

**Répondant** : C’était par instruction ; il ya eu même une instruction écrite.

**P15: IA_01_AS_CSR_01.rtf - 15:21 [Répondant : Il ne fallait que ..] (47:47) (Super)**

Codes: [Service_chirurgie_fonctionnement]

No memos

**Répondant** : Il ne fallait que faire des chirurgies urgentes. Au début on devait faire la chirurgie sans problème, mais en un certain moment, on a exigé que nous ne puissions faire que des chirurgies urgentes et programmées.

**P15: IA_01_AS_CSR_01.rtf - 15:22 [Répondant : Il y en a eu trop ..] (49:49) (Super)**

Codes: [Service_maternité_fonctionnement]

No memos

**Répondant** : Il y en a eu trop parce que nous sommes allés d’une moyenne de 50 accouchements à 100.

**P15: IA_01_AS_CSR_01.rtf - 15:23 [Répondant : Oui c’était pendan..] (51:51) (Super)**

Codes: [Service_maternité_fonctionnement]

No memos

**Répondant** : Oui c’était pendant la période de la gratuité effectivement. Dans la gratuité nous étions dans la centaine d’accouchement par mois.

**P15: IA_01_AS_CSR_01.rtf - 15:24 [Répondant : C’est PDSS.] (53:53) (Super)**

Codes: [Gratuité_Partenaire d'appui]

No memos

**Répondant** : C’est PDSS.

**P15: IA_01_AS_CSR_01.rtf - 15:25 [Répondant : Jusqu’à maintenant..] (55:55) (Super)**

Codes: [Gratuité_Evolution]

No memos

**Répondant** : Jusqu’à maintenant, oui. Mais actuellement c’est une gratuité partielle car elle concerne seulement la médecine interne et la pédiatrie.

**P15: IA_01_AS_CSR_01.rtf - 15:26 [Répondant : Non, c’est rien qu..] (57:57) (Super)**

Codes: [Gratuité_Structures-Services_concernés]

No memos

**Répondant** : Non, c’est rien que l’hospitalisation. L’hospitalisation veut dire que si le malade est dans le circuit, dans les normes, il ne va pas payer ni la consultation ni le laboratoire, ni le dossier et ainsi de suite.

**P15: IA_01_AS_CSR_01.rtf - 15:27 [Répondant : Au début tout étai..] (59:59) (Super)**

Codes: [Gratuité_Structures-Services_concernés]

No memos

**Répondant** : Au début tout était vraiment gratuit.

**P15: IA_01_AS_CSR_01.rtf - 15:28 [Répondant : La gratuité totale..] (61:61) (Super)**

Codes: [Gratuité_Evolution]

No memos

**Répondant** : La gratuité totale est allée du mois d’aout 2018 jusqu’au mois d’avril 2019 si je ne me trompe pas.

**P15: IA_01_AS_CSR_01.rtf - 15:29 [Répondant : Oui cela a changé ..] (63:63) (Super)**

Codes: [Gratuité_Evolution]

No memos

**Répondant** : Oui cela a changé en gratuité partielle vis-à-vis de la médecine interne et de la pédiatrie et même maintenant, nous évoluons dans ce contrat.

**P15: IA_01_AS_CSR_01.rtf - 15:30 [Répondant : Oui il y en a eu. ..] (65:65) (Super)**

Codes: [Gratuité_Interruption]

No memos

**Répondant** : Oui il y en a eu. Je connais qu’en aout 2018, nous étions dans le payant à 100% parce que nous n’avions pas de contrat et en janvier 2020 et même février 2020, nous étions à 100% dans le payement.

**P15: IA_01_AS_CSR_01.rtf - 15:31 [Répondant : Malheureusement, n..] (67:67) (Super)**

Codes: [Gratuité_Interruption]

No memos

**Répondant** : Malheureusement, non. Les malades n’étaient préparés.

**P15: IA_01_AS_CSR_01.rtf - 15:32 [Répondant : Oui. Parce que nou..] (69:69) (Super)**

Codes: [Gratuité_Interruption]

No memos

**Répondant** : Oui. Parce que nous, o attendait de PDSS une précision ; On leur demandait ce qu’il pensait et nous disait qu’il attendait une précision de la hiérarchie. C’est alors que l’on se retrouve comme cela le 31 Décembre ; Alors, on est obligé de faire payer les clients. Cela veut dire qu’il n’y avait pas eu ni de l’annonce comme on l’a fait aujourd’hui n par qui que ce soit.

**P15: IA_01_AS_CSR_01.rtf - 15:33 [Répondant : Nous pensons que p..] (71:71) (Super)**

Codes: [Gratuité_Défis]

No memos

**Répondant** : Nous pensons que pour ce qui nous concerne, la gratuité avait sa raison d’être parce que quand on a commencé la gratuité, on pensait que l’on pouvait avoir de non malades. Mais on a constaté que la population qui a défilé derrière nous, était vraiment une population malade. Alors nous avons pensé que la gratuité avait sa raison d’être et pour le Gouvernement qui finançait, cela devait lui coutait car n’étant pas prévu pour ce moment-là. Mais si ça pouvait être possible, nous avons compris qu’il y avait des décès communautaires dû aux moyens de moyens pour accéder aux soins de santé et cette gratuité a sauvé.

**P15: IA_01_AS_CSR_01.rtf - 15:34 [Répondant : Oui, il y en avait..] (73:73) (Super)**

Codes: [Gratuité_Rumeurs]

No memos

**Répondant** : Oui, il y en avait. Il y eu des rumeurs au début disant que : égal gratuité, égal médicament de mauvaise qualité ; mais après tout la population a compris que c’est le même gouvernement qui donnait cela. Au début la population ne comprenait pas comme il y avait trop de malades et trouvait méfiante l’équipe de soins.

**P15: IA_01_AS_CSR_01.rtf - 15:35 [Répondant : La population a co..] (75:75) (Super)**

Codes: [Considération_prestataire_EPI_population]

No memos

**Répondant** : La population a compris par la sensibilisation qu’il y avait une nouveauté. Comme cela a été une nouveauté pour les prestataires, c’était encore plus une nouveauté pour la population. On ne comprenait pas comment un malade à moto et qu’il fallait effiler les gants, porter la blouse et le masque avant de récupérer le malade du lieu de la moto vers le lit. C’est là que la population a compris que l’équipe était méfiante et le malade se disait qu’il trainait sur la moto alors qu’il est arrivé sur le lieu des soins.

**P15: IA_01_AS_CSR_01.rtf - 15:36 [Répondant : Oui les médicament..] (77:77) (Super)**

Codes: [Médicaments_disponibilité_fosa]

No memos

**Répondant** : Oui les médicaments étaient disponibles par rapport à ce qu’on devrait payer mais pas disponibles à 100% suite à nos différents bureaux centraux de zone de santé. Pour une réquisition de 20 g de médicament on pouvait trouver soit 15 g ou 10; alors là c’était un problème.

**P15: IA_01_AS_CSR_01.rtf - 15:37 [Répondant : On disait que l’on..] (79:79) (Super)**

Codes: [Médicaments_disponibilité_fosa]

No memos

**Répondant** : On disait que l’on n’était pas encore approvisionné en médicaments. Et les antis malaria qui étaient toujours disponibles, un certain moment on a connu une rupture de stock pendant cette période.

**P15: IA_01_AS_CSR_01.rtf - 15:38 [Répondant : Pendant cette péri..] (81:81) (Super)**

Codes: [SNIS_fonctionnement_Decrire_pdt MVE]

No memos

**Répondant** : Pendant cette période de crise, comme tout le monde a été occupé, on a fourbi le rapport au moment opportun mais on a constaté que l’analyse des données au niveau de la zone de santé ne se faisait plus au moment opportun, fixe comme c’était avant. Avant on avait un calendrier qui disait quel tel jour, on devait se présenter au bureau de la zone de santé pour l’analyse des données. Mais depuis l’apparition de l’Ebola, on n’avait plus un calendrier fixe vu que nous étions trop pris voire mêmes les agents de la zone de santé. Pour ce qui est de la supervision, on n’avait plus un calendrier ; c’est pourquoi vous pouvez constater que l’on faisait des supervisions d’une façon circonstancielle. Quand on trouvait ce qui n’allait plus, l’équipe technique pouvait se concertait avec l’équipe du terrain.

**P15: IA_01_AS_CSR_01.rtf - 15:39 [Répondant : D’une façon généra..] (83:83) (Super)**

Codes: [Souhait_Changement_gestion_futures_épidémies]

No memos

**Répondant** : D’une façon générale, pour que ça ne puisse pas avoir de l’impact sur les services de routine, il fallait que nous puissions intéressés les leaders locaux comme on l’a fait à la fin ; avoir une bonne façon de recruter surtout dans les endroits sensibles comme le CTE et le CT. Moi j’étais en train de penser que le CT et le CTE au niveau du bureau central de la zone de santé, on aurait dû venir consulter la structure comme celle-ci pour dire au médecin directeur de proposer les meilleurs pour animer ces centres. Les meilleurs de la structure seront orientés au CT et au CTE et au niveau de la structure, on va engager d’autres qui vont suppléer ceux qui sont retenus comme ce n’est pas beaucoup sensible comme au CTE où un prestataire peut aussi facilement se contaminer. A Part cela, en cas d’une autre épidémie, je souhaiterais que le BCZ e le Ministère puisse penser à diminuer les structures qui pouvaient être à mesure de prendre en charge les malades parce que si nous avons connu des problèmes à Beni, c’est parce qu’on a environ 300 structures de santé sur toute l’étendu de la ville. Avec les 300 qui fonctionnent, l’équipe de riposte a eu du mal à contrôler tous les contacts de personnes contaminées. Le malade atteignait une structure comme celle-ci après avoir passé par 3 ou 4 structures. Malheureusement l’équipe n’était pas aussi capable de gérer les gens. Il faudra donc diminuer les structures de santé dans la ville de Beni. Pour les acteurs, qu’on puisse savoir qui peut faire quoi parce que l’on avait des agents qui n’étaient pas même infirmiers qui étaient dans le triage. Ils n’avaient pas la possibilité d’identifier les signes de l’Ebola si du mois ils sont trompés par les malades. Le Ministère doit insister à ce que l’on puisse continuer avec le service de triage et qu’il y ait une supervision régulière pour s’assurer de l’existence de ce service dans toutes les structures. Cela permettrait de connaitre les malades en cas d’épidémie et d’identifier ceux qui sont gravement malades et conclure que tel malade peut accéder aux soins plus que tel autre. C’est ce que j’aurais dû proposer.

**P15: IA_01_AS_CSR_01.rtf - 15:40 [Répondant : Vis-à-vis de la pr..] (87:87) (Super)**

Codes: [Service_prise-en-charge_fonctionnement]

No memos

**Répondant** : Vis-à-vis de la prise en charge, nous avions accompagné certains de nos prestataires au CT. Arrivé là, on pouvait constater du pire, on ne sait pas considérer la prise en charge de la base et qui pouvait continuer du CT ou du CTE. Si le malade avait déjà commencé son arthézinate, il fallait créer un système à ce qu’au CTE le malade puisse continuer avec cette cure. Mais nous avons observé que c’est aberrant. Ce sont les psychologues qui étaient appelés à donner les résultats. E n cas des résultats négatifs, il n’y avait pas de formations parce que parfois on retrouvait ici les malades que nous avions référés. C’est le malade qui nous informait qu’il avait déjà fait les examens et que les résultats étaient négatifs alors que l’on devait connaitre son évolution au CT. Ou on pouvait voir l’équipe de la décontamination ici dans la structure et qui pouvait confirmer le cas référé.

**P15: IA_01_AS_CSR_01.rtf - 15:41 [Répondant : Il y a eu au tant ..] (89:89) (Super)**

Codes: [Service_prise-en-charge_fonctionnement]

No memos

**Répondant** : Il y a eu au tant de défis par rapport à cela. Nous avons trouvé que peut-être la clinique n’est pas trop approfondie au CTE pour vérifier en dehors de ce que l’on a suspecté comme Ebola, quel est l’état en général ou quotidien du malade par rapport à ce qu’il a comme problème de santé .

**P15: IA_01_AS_CSR_01.rtf - 15:42 [Répondant : C’est ce qui a été..] (93:93) (Super)**

Codes: [Service_prise-en-charge_fonctionnement]

No memos

**Répondant** : C’est ce qui a été observé dans la plupart de cas en tout cas.

**P15: IA_01_AS_CSR_01.rtf - 15:43 [Répondant : En tout cas nous a..] (95:95) (Super)**

Codes: [Service_prise-en-charge_fonctionnement]

No memos

**Répondant** : En tout cas nous avons eu de feed-back des malades en disant qu’ils n’ont continué avec leur cure. One ne pouvait pas nous poser la question de savoir comment le malade était pris en charge avant de venir ici ou alors pour les diabétiques comme vous venez de le dire, on ne pouvait pas savoir que l’on a contrôlé une ou deux fois la glycémie et si on ne l’a pas fait, conclure que le malade a un diabète déséquilibré. Comment on a pris en charge le malade par rapport à son diabète ; on va voir seulement la suspicion de l’Ebola et ne pas voir les autres maladies.

**P15: IA_01_AS_CSR_01.rtf - 15:44 [Le mot à ajouter c’est dire qu..] (98:98) (Super)**

Codes: [Autres_Problèmes observés]

No memos

Le mot à ajouter c’est dire que nous sommes très contents que nous ayons eu cette grâce-là d’être assisté et moi-même et toute l’équipe nous ne savions pas si cette épidémie pouvait avoir cette ampleur. On se disait où est-ce que nous allons tirer une assistance ? Le gouvernement est venu payer toutes les prestations ; c’est là où nous nous sommes dit qu’il y aura de l’espoir que nous avons vécu effectivement. Nous encourageons qui s’investit dans cela d’aller en avant pour booster dehors cette épidémie d’Ebola et cette pandémie à coté de nous.

**P15: IA_01_AS_CSR_01.rtf - 15:45 [Répondant : Idéale était que n..] (100:100) (Super)**

Codes: [PCI_Comment_maintenir]

No memos

**Répondant** : Idéale était que nous maintenions ça, mais il y a toujours des défis par ce que nous avons été doté de cet équipement, malheureusement ces sont les intrants qui coutent extrêmement chers pour la structure, par exemple le gant comme nous avons beaucoup de malades nous utilisons en moyenne dix boites par jour, et une boite nous la payons quatre dollars, pour le moment le prix a haussé à six dollars comme la frontière ougandaise est fermée. Et cela sont de gant latex qui nous coute soixante dollars par jour, après dix jours ça fait six cents dollars et les prestataires ne savent même pas comptabiliser que j’ai utilisé autant de pair de gant chez un malade, c’est le cas de masque. Une boite de cinquante masques coute vingt dollars. Pendant ce moment c’est difficile, alors si ça complique on peut utiliser cette boite pour deux jours, alors idéalement il faut que nous maintenions ça mais financièrement c’est difficile.

**P15: IA_01_AS_CSR_01.rtf - 15:46 [Répondant : Pour le lavage des..] (102:102) (Super)**

Codes: [PCI_Comment_maintenir]

No memos

**Répondant** : Pour le lavage des mains je pense que c’est maintenu comme ça, nous sommes rassurés que nous avons de dispositifs de lavage des mains partout et même dans les locaux des malades et ça pourra continuer ainsi parce que nous ne pouvons pas manquer de l’eau et du savon

**P16: IA_01_AS_IT_02 .rtf - 16:1 [Répondant : Oui effectivement ..] (13:13) (Super)**

Codes: [Début-Progression_Epidémie MVE]

No memos

**Répondant** : Oui effectivement nous sommes permis les aires de santé qui a enregistré le 1er cas dans la zone de santé de Beni au besoin le dernier cas est venu dans notre aire de santé, en sommes nous avons enregistré plus de 260 cas confirmés avec une affaire de 60 dans l’aire de santé.

**P16: IA_01_AS_IT_02 .rtf - 16:2 [Répondant : Le 1er cas-là c’ét..] (15:15) (Super)**

Codes: [Début-Progression_Epidémie MVE]

No memos

**Répondant** : Le 1er cas-là c’était une famille qui a quittée Mangina, quand Mangina était la puissance elle a quittée Mangina de Mangina elle a séjournée a Sinzi une cellule de notre aire de santé. C’est comme ça que la maladie est entrée dans la ville Beni et particulièrement dans l’aire de santé de kanzulinzuli.

**P16: IA_01_AS_IT_02 .rtf - 16:3 [Répondant : Nous avons connu d..] (17:17) (Super)**

Codes: [Considération_Ebola_population]

No memos

**Répondant** : Nous avons connu de cession très énormes difficile à gérer des informations par ce que au début la population croyait a une maladie que le blanc nous on amener, c’est une maladie tueuse pour pouvoir tué le peuple Nande. c’est comme ça que nous avons eu de difficultés énorme au début pour gérer l’épidémie par ce que c’est maladie que le blancs nous on amener pour exterminer le peuple Nande et surtout les habitants de la province du Nord-Kivu en commença par Beni comme on a connue des massacres alors les massacres ont échoué alors on amène la maladie pour nous tuer c’est comme ça qu’on a eu vraiment de difficultés énormes pour gérer l’épidémie au début c’est vers la fin qu’on a essayé à surmonter toutes ces résistances.

**P16: IA_01_AS_IT_02 .rtf - 16:4 [Répondant : Bon les équipes de..] (19:19) (Super)**

Codes: [Considération_Riposte_Population]

No memos

**Répondant** : Bon les équipes de riposte normalement étaient mal vus par la population étant donné que ils ont des amis qui sont venus dans d’autres pays pour les appuyés, il y a même ce qui sont venus de Kinshasa, Goma ils étaient très mal vu très mal vu au début c’est au fur et à mesure que nous avons évoluer avec l’épidémie ou on commençait à combattre certaines choses mais au moins jusqu’à la fin de l’épidémie il avait toujours de résistances par rapport aux amis qui sont venus nous appuyés dans l’épidémie.

**P16: IA_01_AS_IT_02 .rtf - 16:5 [Répondant : Ce sont eux qu’on ..] (21:21) (Super)**

Codes: [Agression-Menaces_Structure-Prestataire_raisons]

No memos

**Répondant** : Ce sont eux qu’on a utilisé pour venir nous tué et comme à Beni il avait de prestataires qui étaient à mesure vraiment de prendre en charge les malades par ce que depuis qu’ils ont connus des épidémies telle que la rougeole on a jamais eu des expatriés qui viennent nous appuyés et pour quoi ils sont venus principalement pour Ebola c.-à-d. ils ont une mission de nous tuer.

**P16: IA_01_AS_IT_02 .rtf - 16:6 [Répondant : En fait nous on no..] (23:23) (Super)**

Codes: [Considération_prestataire_population]

No memos

**Répondant** : En fait nous on nous prenait comme corrompu par ce que elle disait nous les avons réussis ils nous ont amené l’argent effectivement nous sommes corrompus dalleurs au début là on nous disait à chaque alerte ont nous faisaient, cent dollars donc les expatriés nous faisaient cent dollars à chaque alerte donc au début là on nous prenait de corrompu par ce que à chaque appelle à chaque alerte, donc quand tu alertes tu reçois automatiquement cent dollars.

**P16: IA_01_AS_IT_02 .rtf - 16:7 [Répondant : En tout cas la maj..] (25:25) (Super)**

Codes: [Structures-Services_fonctionnement]

No memos

**Répondant** : En tout cas la majorité de services n’ont pas bien fonctionné par ce que on a changé la manière de prendre en charge les malades c.à.d. à chaque arrivé d’un malade on devrait prendre des précautions pour soigner le malade en fait, pour prendre en charge un malade il fallait d’abord se prévenir donc s’habillé, avoir d’équipement complet pour prendre un malade en charge. C’est comme ça qu’on a eu à perdre beaucoup des malades qui n’étaient même pas suspects mais par ce que nous sommes dans l’épidémie on ne devrait pas se précipiter pour prendre un malade en charge par exemple un dans le Kiévienne avec de saignement expliquer c’est peut-être même un avortement mais par ce que on était dans l’épidémie elle reste entre de saigné là entre temps toi tu prends le précautions pour se prévenir malheureusement le temps-là que tu pars t’habillé et pour revenir elle aura déjà perdue la vie ou bien carrément quand on la voit comme suspect au CTE et pour prendre place au CTE il faut au moins deux heures de temps le temps qu’elle va faire là entre de saigné elle va perdre directement sa vie donc, au fait la pris en charge totalement chargé et avec cette manière de prendre en charge le maladie on a eu à perdre beaucoup de malades à cause de retard pour vraiment répondre au besoin de malade.

**P16: IA_01_AS_IT_02 .rtf - 16:8 [Répondant : Elle était mauvais..] (27:27) (Super)**

Codes: [Qualité_soins_Prestataires]

No memos

**Répondant** : Elle était mauvaise aux malades, elle était vraiment mauvaise parce qu’au début là avant l’épidémie tout malade qui venait par exemple un enfant qui civils directement on le prend en charge sans même tarder mais le temps que il faut prendre les épis on va faire quoi on risque de perdre et pourtant l’accueil a effectivement diminué.

**P16: IA_01_AS_IT_02 .rtf - 16:9 [Répondant : Au début ça poser ..] (29:29) (Super)**

Codes: [Triage-Isolement-Chlore-thermoflash_Réaction_population]

No memos

**Répondant** : Au début ça poser de problème mais aujourd’hui maintenant les malades commencent à comprendre

**P16: IA_01_AS_IT_02 .rtf - 16:10 [Répondant : Au fait il n’y ava..] (31:31) (Super)**

Codes: [Triage-Isolement-Chlore-thermoflash_Réaction_population]

No memos

**Répondant** : Au fait il n’y avait pas des partenaires u début mais quand on a évolué avec l’épidémie c’est comme ça que nous avons maintenant les malades et en mettre en place tout ce que vous avez vu là-bas.

**P16: IA_01_AS_IT_02 .rtf - 16:11 [Répondant : En tout cas ils on..] (33:33) (Super)**

Codes: [Triage-Isolement-Chlore-thermoflash_Réaction_population]

No memos

**Répondant** : En tout cas ils ont critiqué ça par ce que ça ne permet pas d’être en contact avec les prestataires il faut d’abord.

**P16: IA_01_AS_IT_02 .rtf - 16:12 [Répondant : Donc le laboratoir..] (35:35) (Super)**

Codes: [Service_laboratoire_fonctionnement]

No memos

**Répondant** : Donc le laboratoire, pendant l’épic on ne fonctionné pas donc le laboratoire ne fonctionné pas quand il y avait baisse de cas ce comme ça qu’on accepter qu’on fasse certain examen mais pendant qui il avait de cas vraiment épic donc pendant l’épic de l’épidémie le laboratoire ne fonctionne pas.

**P16: IA_01_AS_IT_02 .rtf - 16:13 [Répondant : De la zone] (37:37) (Super)**

Codes: [Service_laboratoire_fonctionnement]

No memos

**Répondant** : De la zone

**P16: IA_01_AS_IT_02 .rtf - 16:14 [Répondant : C’était une instru..] (39:39) (Super)**

Codes: [Service_laboratoire_fonctionnement]

No memos

**Répondant** : C’était une instruction verbale

**P16: IA_01_AS_IT_02 .rtf - 16:15 [Répondant : Ça on ne faisait m..] (41:41) (Super)**

Codes: [Service_laboratoire_fonctionnement]

No memos

**Répondant** : Ça on ne faisait même pas de lors on n’a jamais fait ça

**P16: IA_01_AS_IT_02 .rtf - 16:16 [Répondant : Oui] (43:43) (Super)**

Codes: [Service_laboratoire_fonctionnement]

No memos

**Répondant** : Oui

**P16: IA_01_AS_IT_02 .rtf - 16:17 [Répondant : Ça on faisait quan..] (45:45) (Super)**

Codes: [Service_vaccination_Fonctionnement]

No memos

**Répondant** : Ça on faisait quand même

**P16: IA_01_AS_IT_02 .rtf - 16:18 [Répondant : C’est quand l’épic..] (47:47) (Super)**

Codes: [Service_vaccination_Fonctionnement]

No memos

**Répondant** : C’est quand l’épic effectivement comme j’étais entré de le dire quand il y avait l’épic les mamans ne venaient pas par crainte pour la maladie par ce que le fait d’être ensemble on peut être en contact d’un cas confirmer ou un cas probable, d’un cas suspect

**P16: IA_01_AS_IT_02 .rtf - 16:19 [Répondant : Il y avait une dif..] (49:49) (Super)**

Codes: [Service_vaccination_Fonctionnement]

No memos

**Répondant** : Il y avait une différence par ce que ceux qui venaient avec la vaccination contre Ebola ils venaient autrement que nous par ce que nous quand on va dans de séance et la population nous connaissent nous on n’a pas tellement d’équipement qui peut faire peur mais eux quand ils viennent pour la vaccination contre Ebola ils viennent avec de véhicule, tente, chaise effectivement la population se distinguer avec notre vaccination de routine

**P16: IA_01_AS_IT_02 .rtf - 16:20 [Répondant : Pour le confession..] (51:51) (Super)**

Codes: [Service_vaccination_Fonctionnement]

No memos

**Répondant** : Pour le confessionnel c’était la même chose pour ce que à l’église on ne sait saluer pas

**P16: IA_01_AS_IT_02 .rtf - 16:21 [Répondant : Oui on avait insta..] (53:53) (Super)**

Codes: [Structure-Service_Utilisation_population]

No memos

**Répondant** : Oui on avait instauré un système d’isolement une fois il y a un malade une fois on venait récupérer un confirmé ici pendant cette semaine il ne va pas vivre donc cette père-là est un père malade

**P16: IA_01_AS_IT_02 .rtf - 16:22 [Répondant : Ailleurs dans de s..] (55:55) (Super)**

Codes: [Structure-Service_Utilisation_population]

No memos

**Répondant** : Ailleurs dans de structures privées c’est pourquoi nous avons eu beaucoup des cas confirmés

**P16: IA_01_AS_IT_02 .rtf - 16:23 [Répondant : Non, je crois non ..] (57:57) (Super)**

Codes: [Structure-Service_Utilisation_population]

No memos

**Répondant** : Non, je crois non mais en fait nous qui appliquons correctement le mécanisme de triage, isolement la population avait peur ce comme ça qu’ils utilisaient les structures qui n’ont pas ce système ou ce mécanisme

**P16: IA_01_AS_IT_02 .rtf - 16:24 [Répondant : Elle a été bien ac..] (61:61) (Super)**

Codes: [Gratuité_Conséquences_utilisation_services]

No memos

**Répondant** : Elle a été bien accueillie dans la communauté par ce que nous par exemple ici avant l’épidémie nous étions à deux pourcent pour le curatif on était à deux pourcent pour le taux d’utilisation mais aujourd’hui nous sommes dans le 56, 57 pourcent et au début la avant l’épidémie on soigner 200 malades par mois mais aujourd’hui maintenant nous soignons 200 malades par jour pour dire que en tout cas cette gratuité a était bien accueillie.

**P16: IA_01_AS_IT_02 .rtf - 16:25 [Répondant : Non] (63:63) (Super)**

Codes: [Gratuité_Rumeurs]

No memos

**Répondant** : Non

**P16: IA_01_AS_IT_02 .rtf - 16:26 [Répondant : Il y a pas eu vrai..] (65:65) (Super)**

Codes: [Gratuité_Rumeurs]

No memos

**Répondant** : Il y a pas eu vraiment de rumeur au début là où la population ne savez pas comprendre mais au fur et à mesure que nous évoluons avec la sensibilisation là elle commence à comprendre par ce que aujourd’hui maintenant ils viennent En masse et donner que au niveau périphérique il y a de l’insécurité presque toute la population est concentrée dans la ville c’est comme ça que nous avons beaucoup de malade est cette population est diminué non rien maintenant

**P16: IA_01_AS_IT_02 .rtf - 16:27 [Répondant : Ici au début c’éta..] (67:67) (Super)**

Codes: [Gratuité_Partenaire d'appui]

No memos

**Répondant** : Ici au début c’était PDCC, avec fond mondial et aujourd’hui nous somme avec MSF

**P16: IA_01_AS_IT_02 .rtf - 16:28 [Répondant : Ça sa dépend maint..] (69:69) (Super)**

Codes: [Gratuité_Evolution]

No memos

**Répondant** : Ça sa dépend maintenant de chaque partenaire

**P16: IA_01_AS_IT_02 .rtf - 16:29 [Répondant : Ici chez nous tout..] (71:71) (Super)**

Codes: [Gratuité_Défis]

No memos

**Répondant** : Ici chez nous tout est gratuit avec notre partenaire effectivement mais par rapport à d’autre aire de santé il y a aussi d’autre réalité ou il dit par exemple il n’y a que l’accouchement que lui prend en charge et le reste les malades

**P16: IA_01_AS_IT_02 .rtf - 16:30 [Répondant : Oui] (75:75) (Super)**

Codes: [Gratuité_Evolution]

No memos

**Répondant** : Oui

**P16: IA_01_AS_IT_02 .rtf - 16:31 [Répondant : S’est venue seulem..] (73:73) (Super)**

Codes: [Gratuité_Evolution]

No memos

**Répondant** : S’est venue seulement avec Ebola avant il n’y avait pas

**P16: IA_01_AS_IT_02 .rtf - 16:32 [Répondant : Il y a n’a pas eu ..] (77:77) (Super)**

Codes: [Gratuité_Interruption]

No memos

**Répondant** : Il y a n’a pas eu seulement qui y a eu changement de partenaire

**P16: IA_01_AS_IT_02 .rtf - 16:33 [Répondant : Non ici chez nous] (79:79) (Super)**

Codes: [Gratuité_Interruption]

No memos

**Répondant** : Non ici chez nous

**P16: IA_01_AS_IT_02 .rtf - 16:34 [Répondant : De toute le façon ..] (81:81) (Super)**

Codes: [Gratuité_Conséquences_motivation_prestataire]

No memos

**Répondant** : De toute le façon il y a eu une amélioration par ce que avant la gratuité il y avait en fait ce sont les malades qui payés les prestataires mais aujourd’hui le partenaire arrive il dise bon moi je donne le soin gratuit et la motivation de prestataires sera à ma charge c’est comme ça que nous on est payé par les partenaires on est plus payé comme avant par ce que ont étaient payés à partir de ce que on recevait de malades

**P16: IA_01_AS_IT_02 .rtf - 16:35 [Répondant : Septembre la gratu..] (83:83) (Super)**

Codes: [Gratuité_Conséquences_motivation_prestataire]

No memos

**Répondant** : Septembre la gratuité 2018

**P16: IA_01_AS_IT_02 .rtf - 16:36 [Répondant : Chez nous non par ..] (85:85) (Super)**

Codes: [Gratuité_Conséquences_motivation_prestataire]

No memos

**Répondant** : Chez nous non par ce qu’on a avec de partenaire suffisamment fort

**P16: IA_01_AS_IT_02 .rtf - 16:37 [Répondant : Chez nous non] (87:87) (Super)**

Codes: [Ressources_humaines_Disponibilité et perturbations]

No memos

**Répondant** : Chez nous non

**P16: IA_01_AS_IT_02 .rtf - 16:38 [Répondant : Chez nous non] (89:89) (Super)**

Codes: [Ressources_humaines_Disponibilité et perturbations]

No memos

**Répondant** : Chez nous non

**P16: IA_01_AS_IT_02 .rtf - 16:39 [Répondant : Qu’est à la struct..] (91:91) (Super)**

Codes: [Ressources_humaines_Disponibilité et perturbations]

No memos

**Répondant** : Qu’est à la structure ailleurs je ne pas une idée par ce que ces des structures privées mais au moins ils ont connues aussi de perturbation par ce que les prestataires qui préférés aller dans la riposte

**P16: IA_01_AS_IT_02 .rtf - 16:40 [Répondant : Chez nous non] (93:93) (Super)**

Codes: [Ressources_humaines_Disponibilité et perturbations]

No memos

**Répondant** : Chez nous non

**P16: IA_01_AS_IT_02 .rtf - 16:41 [Répondant : Bon je suis tellem..] (141:141) (Super)**

Codes: [Commentaire-Recommandation_Répondant]

No memos

**Répondant** : Bon je suis tellement comptant de l’interview nous pensons que le gouvernement va nous aider à pérenniser tout ce que nous avons acquis dans l’épidémie donc dans le jour à venir nous pensons que on aura plus de victime, et on va nous payer par ce que avec l’épidémie on a eu à gagner suffisamment de l’argent et nous pensons que si ça restait le même, avec le partenaire on est payé sans problème mais si on retournait encore en arrière est ce que le gouvernement sera à mesure de nous payer.

**P16: IA_01_AS_IT_02 .rtf - 16:42 [Répondant : En tout cas l’idéa..] (139:139) (Super)**

Codes: [PCI_Comment_maintenir]

No memos

**Répondant** : En tout cas l’idéal est de maintenir tout ce que nous avons acquis par rapport à Ebola malheureusement nous sommes entre de pressentir qu’on sera toujours en rupture par ce que le partenaires sont entré de se désengagés ou est-ce que on aura de gant pour soigner la malade chaque jour voilà mais au moins l’idéal est de pérennise tout ce que Ebola nous a laisser comme acquis mais au moins le rupture de stock par ce que le partenaires sont entré de partir, on ne se pas comment on peut surmonter se rupture par ce que le pays aussi pour dire que il y arrive de fois où nous manquons même le médicament.

**P16: IA_01_AS_IT_02 .rtf - 16:43 [Répondant : Bon moi je crois q..] (137:137) (Super)**

Codes: [Agression-Menaces_Structure-Prestataire_solution]

No memos

**Répondant** : Bon moi je crois que, j’étais entré même de dire aux amis que nous sommes un peu comptant par ce que d’abord on est à la fin de l’épidémie a Ebola mais aussi cette pandémie nous a aussi renforcé par ce que au début là on disait que nous avons amené Ebola mais d’autant plus que cette population est entré de suivre à la radio que a chine, en Italie il y a de cas de décès par rapport à cette maladie elle commence à comprendre que ah doc avec Ebola c’est n’était pas une maladie jetée par ce que ailleurs aussi. Donc on peut dire que un moment donné nous somme un peu comptant par rapport à la survenue de corona virus jusque à ce que elle fait maintenant comprendre à la population que c’était réellement une maladie tueuse et qu’on devrait se protéger et réellement pour nous la maladie à commencer à hier pas chez nous par ce que si c’était chez nous ils étaient horrible.

**P16: IA_01_AS_IT_02 .rtf - 16:44 [Répondant : Dans la communauté..] (135:135) (Super)**

Codes: [Changements_depuis_Ebola]

No memos

**Répondant** : Dans la communauté d’abord nous sommes mal vu, on a pas gagné la confiance de la communauté pendant cette période donc il y a vraiment de conflits que nous devons gérés par ce que on a même eu de cas que nous avons référé au niveau du CTE pour confirmer et puis les cas sont décédés, leur familles nous portent à cœur on ne sait pas ce qui viendra après mais nous avons suffisamment de conflits dans la communauté. Bon au niveau inter personnelle ici comme soignant il y a de structures privées, nous n’avons pas vraiment de relation sur avec les structures privées ici chez nous nous avons eu a gérée la gratuité et cette gratuité a puis récupérée tous les patients qui se trouvent dans la communauté et eux ne font presque rien la bas c’est comme ça que on a de petit confit là.

**P16: IA_01_AS_IT_02 .rtf - 16:45 [Répondant : Bon il est vraimen..] (133:133) (Super)**

Codes: [Souhait_Changement_gestion_futures_épidémies]

No memos

**Répondant** : Bon il est vraiment idéal qu’on puisse respecter le système sanitaire normal donc si il aurait encore une autre crise qu’on respecte ce système-là donc que ça soit au moins nous comme infirmités titulaires de l’aire de santé qui gérons l’épidémie avec la zone de santé. Est-ce que on est suffisamment outiller et puis nous avons connus de problème avec les amis qui sont venus nous aider vraiment on a connu beaucoup de problème, nous ont pensé que si il aurait encore une autre crise pareille qu’on respecte le système de soin de santé primaire donc que ça soit l’épidémie, qu’elle soit gérée d’abord comme nous avons maintenant de cellule d’animation communautaire qui ont fonctionnés dans la communauté qu’on utilise celle-là et nous comme le gérant de l’aire de santé nous puissions vraiment remonter les formations de la zone à la province mais qui est encore d’autres personnes qui peuvent intervenir, effectivement ils peuvent venir nous accompagnés mais de toute façon on est suffisamment outiller par rapport à la gestion de l’épidémie.

**P16: IA_01_AS_IT_02 .rtf - 16:46 [Répondant : Je peux dire que ç..] (131:131) (Super)**

Codes: [Gouvernance_soins santé]

No memos

**Répondant** : Je peux dire que ça peut, c’est dans le deux sens par ce que on peut dire la que parmi les amis la qui sont venus intervenir dans la riposte il y avait effectivement de gens qui nous aidés c.à.d. qui sont venus vraiment pour nous aider mais il y avaient aussi ce qui sont venus pour l’agent par ce que on a eu avoir de vendeur de poisson qui ont étaient recrutés comme agents dans la riposte et quand ils arrivent sur terrain vous constaté que ils ne vous aidaient pas donc il y a d’autre qui sont venus nous aider et on a compris réellement qui sont venus nous aider donc d’autre qui sont venus seulement pour chercher l’argent.

**P16: IA_01_AS_IT_02 .rtf - 16:47 [Répondant : Chez nous non mais..] (95:95) (Super)**

Codes: [Fosa_Présence_Agent MVE]

No memos

**Répondant** : Chez nous non mais nous sommes passé au CT comme suspect par ce que à chaque fois qu’on présentait de signe

**P16: IA_01_AS_IT_02 .rtf - 16:48 [Répondant : De toute façon nou..] (97:97) (Super)**

Codes: [Ressources_humaines_conditions_travail]

No memos

**Répondant** : De toute façon nous personnellement nous avons constaté qu’il y a une amélioration par ce que au début là on ne sait protéger même pas mais l’épidémie nous a suffisamment essuie, par ce que depuis maintenant on est outillés on connait maintenant les mesures de protection ainsi de suite. Donc ça a amélioré vraiment la manière de prendre en charge les malades malheureusement pour le malades c’est le contraire mais nous personnellement on a compris que c’est comme ça qu’il fallait.

**P16: IA_01_AS_IT_02 .rtf - 16:49 [Répondant : Pour les malades e..] (99:99) (Super)**

Codes: [Considération_prestataire_EPI_population]

No memos

**Répondant** : Pour les malades eux ils constatent que c’est une façon pour le distraire par ce que au début ont soigné sans gant mais aujourd’hui on soigne avec gant.

**P16: IA_01_AS_IT_02 .rtf - 16:50 [Répondant : Nous aussi on étai..] (101:101) (Super)**

Codes: [Ressources_humaines_situation_grève]

No memos

**Répondant** : Nous aussi on était en grave effectivement par ce que on n’est pas payé jusque aujourd’hui nous sommes ici.

**P16: IA_01_AS_IT_02 .rtf - 16:51 [Répondant : Cette grève à comm..] (103:103) (Super)**

Codes: [Ressources_humaines_situation_grève]

No memos

**Répondant** : Cette grève à commencer au mois d’avril je crois jusqu’au mois de juin voir même juillet là effectivement on n’est pas payé jusque aujourd’hui on est reçoit même pas la prime de risque ici nous sommes au nombre de 35 mais il n’y a que 2 qui sont reconnus par l’état.

**P16: IA_01_AS_IT_02 .rtf - 16:52 [Répondant : C’est ce qui a déc..] (105:105) (Super)**

Codes: [Ressources_humaines_situation_grève]

No memos

**Répondant** : C’est ce qui a déclasser cette épidémie on ne savait plus rapporter le cas.

**P16: IA_01_AS_IT_02 .rtf - 16:53 [Répondant : En tout cas chez n..] (107:107) (Super)**

Codes: [Médicaments_disponibilité_fosa]

No memos

**Répondant** : En tout cas chez nous avec notre partenaire il y a pas de souci par rapport à ça, on est avec MSF on a toujours de stocks sur nous.

**P16: IA_01_AS_IT_02 .rtf - 16:54 [Répondant : Ils ont amélioré s..] (109:109) (Super)**

Codes: [Médicaments_disponibilité_fosa]

No memos

**Répondant** : Ils ont amélioré suffisamment par ce que avant on tomber dans de rupture, nous sommes entré de regretter par ce qu’ils vont partir bientôt.

**P16: IA_01_AS_IT_02 .rtf - 16:55 [Répondant : D’ici avril on dit..] (111:111) (Super)**

Codes: [Gratuité_Existence_plan_désengagement]

No memos

**Répondant** : D’ici avril on dit qu’on se désengager.

**P16: IA_01_AS_IT_02 .rtf - 16:56 [Répondant : Ce n’est pas encor..] (113:113) (Super)**

Codes: [Gratuité_Existence_plan_désengagement]

No memos

**Répondant** : Ce n’est pas encore détaillé

**P16: IA_01_AS_IT_02 .rtf - 16:57 [Répondant : Le fait de venir s..] (115:115) (Super)**

Codes: [Gratuité_Information_population]

No memos

**Répondant** : Le fait de venir se faire soigner gratuitement, et il y avait aussi de relais communautaire qui passé pour donner de message par rapport à la gratuité.

**P16: IA_01_AS_IT_02 .rtf - 16:58 [Répondant : Bon je veux dire q..] (117:117) (Super)**

Codes: [SNIS_fonctionnement_Decrire_pdt MVE]

No memos

**Répondant** : Bon je veux dire que ça ne fonctionner pas comme avant par ce que on était trop pris dans l’épidémie, ça affecté vraiment ce système par ce que le temps que vous devrez vous mettre pour le rapport il y a des cas dans la communauté donc ça n’a pas bien fonctionné ont n’étaient trop pris plus dans la communauté que au niveau de la cible par ce que il fallait renforcer le message s’il y a de cas listé décontaminé. En bref ça prenait beaucoup de temps dans la communauté donc le système nous avons de performance par rapport à la complétude de rapport en venant insuffisamment limité.

**P16: IA_01_AS_IT_02 .rtf - 16:59 [Répondant : Bon c.à.d. par exe..] (121:121) (Super)**

Codes: [SNIS_fonctionnement_Decrire_pdt MVE]

No memos

**Répondant** : Bon c.à.d. par exemple vous savez je vous ai dit tantôt que au début là par exemple 200 malades par mois mais aujourd’hui nous sommes à 200 malades par jour le temps de prendre tous ces malades dans le registre vous n’avez pas ce temps-là, donc il vous arrivez de fois que vous enregistrez 50 au lieu de 200, dans ces conditions-là les données directement.

**P16: IA_01_AS_IT_02 .rtf - 16:60 [Répondant : Oui certainement.] (119:119) (Super)**

Codes: [SNIS_fonctionnement_Decrire_pdt MVE]

No memos

**Répondant** : Oui certainement.

**P16: IA_01_AS_IT_02 .rtf - 16:61 [Répondant : Pas nécessairement..] (123:123) (Super)**

Codes: [Ressources_humaines_charge_travail]

No memos

**Répondant** : Pas nécessairement

**P16: IA_01_AS_IT_02 .rtf - 16:62 [Répondant : Par ce que on a av..] (125:125) (Super)**

Codes: [Ressources_humaines_charge_travail]

No memos

**Répondant** : Par ce que on a avec notre partenaire nous on était par exemple a 17 ici avant l’épidémie mais avec le partenaire il a ajouté le nombre de prestataire dont nous sommes passé de 17 à 35 c’est comme ça que on a était à mesure de bien gérer la situation.

**P16: IA_01_AS_IT_02 .rtf - 16:63 [Répondant : Non ça diminue.] (127:127) (Super)**

Codes: [Réunion_pdt-épidémie-Ebola]

No memos

**Répondant** : Non ça diminue.

**P16: IA_01_AS_IT_02 .rtf - 16:64 [Répondant : Par ce que on étai..] (129:129) (Super)**

Codes: [Réunion_pdt-épidémie-Ebola]

No memos

**Répondant** : Par ce que on était plus dans la communauté que au niveau de centre de santé.

**P17: IA_01_AS_IT_03.rtf - 17:1 [Répondant : Nous sommes l’aire..] (9:9) (Super)**

Codes: [Fosa-Institution_Présentation]

No memos

**Répondant** : Nous sommes l’aire de Santé de Mabolio une aire de santé qui compte 21.946 habitants, et neuf cellules qui sont dans deux quartiers différents il y a le quartier Nyankude qui a deux cellules et le quartier le reste de cellules Mabolio et puis nous sommes entourés de l’aire de santé Mukulia.

**P17: IA_01_AS_IT_03.rtf - 17:2 [Répondant : Quand je quitte la..] (11:11) (Super)**

Codes: [Répondant_Responsabilité_habituelles]

No memos

**Répondant** : Quand je quitte la maison la famille connait que je suis infirmier elle retient que je viens soigner le gens de l’aire de santé se Mabolio.

**P17: IA_01_AS_IT_03.rtf - 17:3 [Répondant : D’abord l’Ebola a ..] (13:13) (Super)**

Codes: [Début-Progression_Epidémie MVE]

No memos

**Répondant** : D’abord l’Ebola a Commencé vers Mangina à trente kilomètre de la ville de Beni, et lorsque l’Ebola est arrivée à Mangina j’étais dans l’aire de santé de Sayon qui est voisin de Mangina, il y avait beaucoup d’historiette les gens ne croyaient pas, à l’origine on disait de chat qui était consommé par une famille et ce par-là que le gens sont tombés malades et à la longue ils ont compris que c’était une maladie comme ils pensaient à une sorcellerie, on a compris après une longue recherche que c’était une maladie causé par le Virus et on a fini de dire qu’il s’agissait d’un virus d’Ebola. Alors là il y a eu beaucoup de décès, d’abord l’épidémie avait commencé sous coulisse, les étaient en grève et c’était à partir de mois de Mai 2018 que les gens avaient commencé à tomber malade, c’est à partir du 01 Aout qu’on a déclaré que c’est une épidémie. Depuis mai jusqu’en Aout c’était sous coulisse et on ne sait pas combien qui sont morts. Alors la maladie est arrivée à Beni c’était avec les gens qui fouillaient la maladie sont arrivés à Beni come les gens ne croyaient pas il y a eu trop de résistances, malgré les mesures qu’on demandait de respecter les gens ne croyaient pas à la maladie. Lorsque les gens de Beni commencé à mourir c’est à ce moment-là qu’on avait tiré attention qu’il faut observer les règles d’hygiènes pour la prévention. Une épidémie avec le contact, les gens parvenaient à désemballer les cadavres le monde était contaminé et la maladie s’est éparpillé comme ça dans toutes les aires de santé. Maintenant ici au niveau de Mabolio c’était vers le mois de septembre octobre comme j’étais encore dans l’aire de santé de Sayon, la maladie est arrivée dans cette de santé de Mabolio et comme nous avons un peuple autochtone, il y a eu trop de résistances, de décès, c’est plus tard que le gens se sont ressaisis dans l’aire de Kanzuli les gens ont décédés, ils ont compris que c’est une maladie cette a essayé de se stopper quelque part. Alors nous, qu’est-ce que nous faisons ? Avec la sensibilisation, les infirmiers, les agents communautaires et la population on a créé la surveillance communautaire et les cas ont diminué petit à petit jusqu’à s’arrêter au niveau de l’aire de santé et jusqu’à maintenant on enregistre plus de cas.

**P17: IA_01_AS_IT_03.rtf - 17:5 [Répondant : Connue malheureuse..] (17:17) (Super)**

Codes: [Considération_Ebola_population]

No memos

**Répondant** : Connue malheureusement elle était décédée on disait que c’est une maladie qui a été créer comme on vivait la période de massacre pour essayer d’éliminer le peuple Nande et donc c’était une histoire montée.

**P17: IA_01_AS_IT_03.rtf - 17:6 [Répondant : Ce qui se racontai..] (15:15) (Super)**

Codes: [Début_épidémie_Element déclencheur]

No memos

**Répondant** : Ce qui se racontait, d’abord je vous ai raconté l’histoire d’un chat qui était mangé par une famille, deuxièmement on disait qu’il s’agissait d’une femme qui était allé dans le deuxième monde comme on le dit et pour essayer de payer sur ce qu’on lui avait demandé de tuer les gens et maintenant il fallait créer une histoire qui va tuer les gens

**P17: IA_01_AS_IT_03.rtf - 17:7 [Répondant : C’est ça qui était..] (19:19) (Super)**

Codes: [Agression-Menaces_Structure-Prestataire_raisons]

No memos

**Répondant** : C’est ça qui était, quand on leurs disait de faire attention sur la maladie, ils répondaient que « Non nous savons que vous avez créé cette maladie, et comme l’épidémie n’a jamais été dans le milieu et lorsqu’ils voyaient les Experts venir et surtout qu’on avait envoyé les experts qui ne connaissent pas la langue locale, on disait qu’après avoir créé la maladie un nous avoie les promoteurs pour venir gagner l’argent et ce qui avait créer la résistance puisque quand on arrivait dans le ménage on ne s’adaptait pas facilement et surtout lorsque l’équipe de la riposte est arrivé on avait semblé ignorer les personnes cibles tel que les infirmiers qui restait auprès des malades, on prenait les relais communautaires qui ne restaient pas auprès de malade et comme nous vivons le monde de privé les gens n’acceptaient pas, et quand on demandait à un privé de référer les malades il vous disait, comment est-ce que je vais vivre. Ils gardaient les malades quelques soit les signes et les malades mourraient dans les structures comme ça et ce qui avait fait la résistance.

**P17: IA_01_AS_IT_03.rtf - 17:8 [Répondant : Nous aussi lorsqu’..] (21:21) (Super)**

Codes: [Agression-Menaces_Structure-Prestataire_raisons]

No memos

**Répondant** : Nous aussi lorsqu’on faisait la sensibilisation ou soit qu’il y a décès dans la communauté et qu’on leurs dit d’attendre l’équipe de la riposte on vous considère faisant parti de cette équipe de riposte et c’est comme ça qu’on s’attaquait aux prestataires et aux structures oubliant que c’est leurs structures.

**P17: IA_01_AS_IT_03.rtf - 17:9 [Répondant : Ici à Beni les str..] (23:23) (Super)**

Codes: [Structures-Services_fonctionnement]

No memos

**Répondant** : Ici à Beni les structure ne remplissent pas les conditions, les manque des matériels et les personnels non qualifiés c’était compliqué et avant même l’apparition de l’épidémie on travaillait difficilement, c’était le système de payer les soins mais avec la période de la riposte ç’a été un mal nécessaire. Mal par ce que nous avons perdu les amis, nécessaire par ce que on a eu à améliorer les structures par ce qu’il a eu la gratuité, même au début de la gratuité les gens refusaient ça, ils disaient que ce sont des soins d’Ebola qu’on veulent exterminer la population à partir de ces produits. Avec la sensibilisation, on a compris que c’était un acquis comme la population était déjà vulnérable elle devrait recevoir les soins gratuits. En suite les structures qui étaient dans la gratuité travail avec le PDSS on a quand même construit, par ce que nombreux de nos bâtiments étaient en usés. Il y a des partenaires qui étaient venus et qui avaient construit mais il y a d’autres structures qui n’ont pas eu de partenaire tel qu’à Sayon où je fus, il n’avait pas de cas d’Ebola. Au niveau des prestataires dans des structures étatiques on avait pris les charges de certains prestataires pas toujours tous dans la riposte et même dans la surveillance et d’autres dans la gratuité mais ce n’était pas comme ceux qui étaient dans la surveillance.

**P17: IA_01_AS_IT_03.rtf - 17:10 [Répondant : Ç’a n’a pas posé u..] (25:25) (Super)**

Codes: [Structures-Services_fonctionnement]

No memos

**Répondant** : Ç’a n’a pas posé un problème comme ce n’était pas un service étalé, c’était en secret et on prenait seulement les IT.

**P17: IA_01_AS_IT_03.rtf - 17:11 [Répondant : Non ça n’a pas per..] (27:27) (Super)**

Codes: [Ressources_humaines_Disponibilité et perturbations]

No memos

**Répondant** : Non ça n’a pas perturbé donc nous étions pris tout en étant dans la structure, on faisait la surveillance dans la structure. Par contre ceux qui étaient nomades au niveau de la surveillance n’étaient pas les prestataires de la structure.

**P17: IA_01_AS_IT_03.rtf - 17:13 [Répondant : Oui surtout la gra..] (31:31) (Super)**

Codes: [Service_maternité_fonctionnement]

No memos

**Répondant** : Oui surtout la gratuité et la maternité aussi.

**P17: IA_01_AS_IT_03.rtf - 17:14 [Répondant : Je prends l’exempl..] (29:29) (Super)**

Codes: [Service_maternité_fonctionnement]

No memos

**Répondant** : Je prends l’exemple d’ici selon les archives, avant la riposte, la gratuité on atteignait difficilement cinq accouchées mais pendant la riposte on arrivait à cinquante et actuellement on atteint les vingtaines donc c’a donné un impact positif.

**P17: IA_01_AS_IT_03.rtf - 17:15 [Répondant : Tout juste quand l..] (33:33) (Super)**

Codes: [Gratuité_Début]

No memos

**Répondant** : Tout juste quand l’épidémie avait commencé on avait instauré le système de la gratuité.

**P17: IA_01_AS_IT_03.rtf - 17:16 [Répondant : Le début comme il ..] (35:35) (Super)**

Codes: [Gratuité_Début]

No memos

**Répondant** : Le début comme il n’y avait pas de gratuité c’est difficile.

**P17: IA_01_AS_IT_03.rtf - 17:17 [Répondant : Tout au début on v..] (37:37) (Super)**

Codes: [Service_vaccination_Fonctionnement]

No memos

**Répondant** : Tout au début on voulait arrêter mais on s’était dit que si on arrêtait le PEV de routine on va risquer de sacrifier les enfants et c’ainsi qu’on continuait toujours.

**P17: IA_01_AS_IT_03.rtf - 17:18 [Répondant : Non, seulement ce ..] (39:39) (Super)**

Codes: [Service_vaccination_Fonctionnement]

No memos

**Répondant** : Non, seulement ce qui était dit, on avait suspendu le système de TDR contre le palu même jusqu’à présent on n’a pas encore autorisé ça mais la vaccination on continue.

**P17: IA_01_AS_IT_03.rtf - 17:21 [Répondant : Lorsqu’on a insatu..] (45:45) (Super)**

Codes: [Triage_comportement_patient]

No memos

**Répondant** : Lorsqu’on a insaturé le système du triage ils arrivaient à trainer comme c’était déjà dans la gratuité ils étaient nombreux, et comme ils étaient habitués à la période d’avant ; arriver étant moins nombreux ils allaient immédiatement à la réception chose contraire pendant l’installation du triage car ils arrivaient étant nombreux pour eux croyaient que c’est le triage qui causait leurs retards petit à petit ils compris que c’était à cause de l’épidémie et surtout que le triage était à côté de l’isolement, lorsque on venait avec la fièvre on doit vous vous mettre à coté pour l’observation .

**P17: IA_01_AS_IT_03.rtf - 17:22 [Répondant : L’isolement au dép..] (47:47) (Super)**

Codes: [Triage-Isolement-Chlore-thermoflash_Réaction_population]

No memos

**Répondant** : L’isolement au départ ce n’est pas tout le monde qui l’acceptait, lorsqu’on parlait de l’isolement on pensait que la personne est déjà positive de la maladie et là il avait tendance de faire une résistance, il y a même les gens qui évadaient. Avec la sensibilisation ils ont commencé à s’adapter.

**P17: IA_01_AS_IT_03.rtf - 17:23 [Répondant : Oui je peux dire p..] (49:49) (Super)**

Codes: [Triage_comportement_patient]

No memos

**Répondant** : Oui je peux dire partout par ce que lors de la sensibilisation qu’il disait que si on arrivait avec la fièvre on doit vous mettre dans l’isolement c’est ainsi qu’ils devaient prendre le paracétamol pour arrêter la fièvre, s’il s’agissait de la diarrhée on doit cacher la situation il disait qu’il souffrait de maux de tête et c’est tout. Lorsque vous l’obtempérer 36°C ou 36,5°maintenat étant au lit après un moment donné on revient pour la prise de température on trouve 38°C quand vous posez la question par rapport à cette température comme il est interné c’est à ce moment qu’il vous dira qu’il a quitté chez lui après avoir pris le paracétamol. Ils ne voulaient pas qu’ils soient isolés.

**P17: IA_01_AS_IT_03.rtf - 17:24 [Répondant : Là je dirai oui pa..] (51:51) (Super)**

Codes: [Prestataire_Attitude_devant_malade]

No memos

**Répondant** : Là je dirai oui par ce qu’au début s’il s’agissait d’un cas de fièvre automatiquement ça devrait être un cas d’alerte et quand c’est la fièvre on sous-entend la céphalée comme nous sommes dans la région du paludisme, soit douleur abdominale et là c’est un cas suspect. Avec la protection comme on était déjà au début on portait de masque à tout moment, même jusqu’à présent on ne peut pas toucher un malade sans porter le gant.

**P17: IA_01_AS_IT_03.rtf - 17:25 [Répondant : Avec les mesures d..] (41:41) (Super)**

Codes: [Prestataire_Attitude_devant_malade]

No memos

**Répondant** : Avec les mesures de précaution, ils ont été formés sur la prudence des injections, et la sécurité des injections surtout. C’est ce qui était.

**P17: IA_01_AS_IT_03.rtf - 17:26 [Répondant : D’habitude c’est u..] (43:43) (Super)**

Codes: [Prestataire_Attitude_devant_malade]

No memos

**Répondant** : D’habitude c’est une seringue par patient, le système là de par capuchonné par exemple l’aiguille une fois utilisé on la jette dans le réceptacle et là on est quand même protégé.

**P17: IA_01_AS_IT_03.rtf - 17:27 [Répondant : Oui, avec la coutu..] (53:53) (Super)**

Codes: [Prestataire_Attitude_devant_malade]

No memos

**Répondant** : Oui, avec la coutume et ici chez nous l’habitude qu’on avait quand un malade arrivait on devait lui toucher, avec l’épidémie quelqu’un qu’on soignait avant il savait comment vous lui consultait d’un coup il vient pendant l’épidémie vous l’observez et il se demande pourquoi on ne veut plus les toucher, il fallait expliquer le pourquoi.

**P17: IA_01_AS_IT_03.rtf - 17:28 [Répondant : Pendant la période..] (55:55) (Super)**

Codes: [Qualité_Soins_Patients]

No memos

**Répondant** : Pendant la période chaude de l’épidémie, il y a ceux qui ne voulaient pas se faire soigner là où il y avait la gratuité, ils voulaient partir là où ils seraient facturés. Après une longue période de sensibilisation ils ont compris que la gratuité c’est partout où il y a les structures étatiques y compris la maternité et ils ont commencé à retourner et surtout que les structures privées ont connus beaucoup de cas positives d’Ebola, les malades commençaient à avoir peur et là il y avait des prestataires qui prestaient sans porter l’EPI, C’est ainsi qu’ils ont trouvé qu’ils parcouraient un danger et ils retourné vers les structures étatiques.

**P17: IA_01_AS_IT_03.rtf - 17:29 [Répondant : Ça posé un problèm..] (57:57) (Super)**

Codes: [Considération_prestataire_EPI_population]

No memos

**Répondant** : Ça posé un problème par ce que les malades ne voulaient pas qu’on lui soigne avec l’EPI ils demandaient qu’on puisse enlever les EPI. Ils disaient que ce que nous portons comme EPI c’est l’Ebola.

**P17: IA_01_AS_IT_03.rtf - 17:30 [Répondant : La réponse c’est o..] (59:59) (Super)**

Codes: [Structure-Service_Utilisation_population]

No memos

**Répondant** : La réponse c’est oui par ce que toutes les structures étatiques les mesures étaient les mêmes.

**P17: IA_01_AS_IT_03.rtf - 17:31 [Répondant : Les mesures étaien..] (61:61) (Super)**

Codes: [Structure-Service_Utilisation_population]

No memos

**Répondant** : Les mesures étaient le triage, l’EPI et les soins gratuits et surtout dans les structures officielles on envoyait les gens au CTE alors que chez les privés n’était pas le cas car pour eux ils pouvaient hospitaliser un malade pendant 4jours sans penser en lui referer.002241

**P17: IA_01_AS_IT_03.rtf - 17:32 [Répondant : Tout au début les ..] (63:63) (Super)**

Codes: [Considération_CTE_population]

No memos

**Répondant** : Tout au début les gens décédaient en vrac par ce que le monde avait été totalement contaminé et avec cette habitude de se cacher nombreux étaient décédés. Lorsque les gens allaient au CTE confirmés, ils mourraient par ce qu’ils venaient avec retard et le gens pensaient que lorsqu’on va au CTE on devait décéder coute que coute « BAKISHA KUTUMA KULE UNAENDA KUFA »et les gens ne voulaient pas y aller. Même dans notre structure on pouvait recevoir un cas et lui connais qu’il répond au critère et lorsqu’il en entend l’équipe entré dans la structure il évadait par ce qu’on leurs explique tout et on ne saurait pas quand est ce qu’il est parti. Si vous n’avez pas son adresse complète c’était difficile de le retrouver et les malades commençaient à nous donner les faux noms et nous tromper avec les adresses. Si vous n’êtes pas du milieu, vous n’alliez pas le retrouver. S’il y avait là des gens suspects à haut risque c’est comme ça qu’ils se contaminaient là où il est allé se cacher il contamine ceux qui y sont, il va quitter là vers une autre structure ainsi de suite et ce comme ça qu’il y avait une chaine de contamination.

**P17: IA_01_AS_IT_03.rtf - 17:33 [Répondant : J’ai dit qu’avec E..] (65:65) (Super)**

Codes: [Gratuité_Rumeurs]

No memos

**Répondant** : J’ai dit qu’avec Ebola on veut exterminer les gens comme les massacres récents, qu’avec la gratuité on veut enrôler les gens, il faut connaitre les gens qui sont entrain de recevoir les soins comme ça on viendra les éliminer. Il y avait un décès dans la communauté, là c’était compliqué il fallait faire le lobbying avec les membres familiers, chercher le chef de cette entité pour vous accompagner pour convaincre la famille. Le faire voir que nous sommes dans une période contraire de ce qu’on vivait et là on nous exigeait ce qu’on voulait en nous disant que nous acceptons mais à condition que vos gens n’arrivent pas ici. C’est à ce moment qu’on avait dit que lorsqu’il y a décontamination il faudrait qu’il ait un membre de la communauté. Et au début on organisait les obsèques sans les membres de familles et ils se demandaient si on a réellement enterré leurs familiers, soit on a arraché une partie de son corps. Alors on a essayé d’apaiser les esprit en disant que chaque fois qu’il ait enterrement il faut chaque fois associé un membre famille de l’illustre disparu et c’est ce qui se fait maintenant.

**P17: IA_01_AS_IT_03.rtf - 17:34 [Répondant : Certaines structur..] (67:67) (Super)**

Codes: [Gratuité_Partenaire d'appui]

No memos

**Répondant** : Certaines structure étaient appuyé par PDSS, d’autres par MEDAIR, il y a MSF, dans cette structure il y avait IRC, et c’est cette dernière qui nous a appuyé totalement dans la gratuité mais PDSS est arrivé après.

**P17: IA_01_AS_IT_03.rtf - 17:35 [Répondant : C’était avec Ebola..] (69:69)	(Super)**

Codes:	[Gratuité_Début]

No memos

**Répondant** : C’était avec Ebola, avant Ebola il n’y avait rien à l’arrivée de l’Ebola qui IRC est arrivé.

**P17: IA_01_AS_IT_03.rtf - 17:36 [Répondant : IRC lorsqu’il est ..] (71:71)	(Super)**

Codes:	[Gratuité_Structures-Services_concernés]

No memos

**Répondant** : IRC lorsqu’il est arrivée je n’étais pas là, mais il avait commencé par la gratuité totale et même à son départ il avait laissé un bon stock de médicaments que nous continuons à utiliser jusqu’à présent or il y a longtemps qu’il est parti (en novembre 2019).

**P17: IA_01_AS_IT_03.rtf - 17:37 [Répondant : PDSS est arrivée a..] (73:73)	(Super)**

Codes:	[Gratuité_Partenaire d'appui]

No memos

**Répondant** : PDSS est arrivée au même moment que l’IRC, PDSS c’était la gratuité au début mai vers juillet 2019 qu’il a considéré la consultation externe jusqu’aujourd’hui. D’autres partenaires lorsqu’ils interviennent ils excluent ladite consultation externe.

**P17: IA_01_AS_IT_03.rtf - 17:38 [Répondant : Là je ne saurais p..] (75:75)	(Super)**

Codes:	[Gratuité_Partenaire d'appui]

No memos

**Répondant** : Là je ne saurais pas répondre comme je n’étais pas encore dans la structure.

**P17: IA_01_AS_IT_03.rtf - 17:39 [Répondant : La disponibilité j..] (77:77)	(Super)**

Codes:	[Médicaments_disponibilité_fosa]

No memos

**Répondant** : La disponibilité je le décris compte tenu des médicaments essentiels que nous utilisons souvent lorsqu’il n’y a pas de rupture on dit quand même ça va.

**P17: IA_01_AS_IT_03.rtf - 17:40 [Répondant : Non pas de problèm..] (79:79)	(Super)**

Codes:	[Médicaments_disponibilité_fosa]

No memos

**Répondant** : Non pas de problème.

**P17: IA_01_AS_IT_03.rtf - 17:41 [Répondant : Au niveau du triag..] (81:81)	(Super)**

Codes:	[SNIS_fonctionnement_Decrire_pdt MVE]

No memos

**Répondant** : Au niveau du triage là, lorsqu’un malade est déjà suspect on lui met à l’isolement, après investigation c’est soit au CTE, soit on invalide le cas il reste en observation. A la consultation ici tel que je vous l’ai dit un cas peut nous échapper arrivant ici vous constatez que le malade tend à la définition vous le ramenez à l’isolement pour être investiguer cela crée un problème, au niveau de la maternité on ne recevait pas les cas de menace d’accouchement prématuré, on les envoyait immédiatement au CT. On ne pouvait pas s’hasarder et même aujourd’hui on envoie directement. A la fin de la journée on devait faire la réunion de plateforme avec les agents de surveillance pour essayer de solidifier nos données et jusqu’à présent ça se fait et à la fin du mois chaque chef de service doit amener ses données, nous les analysons en équipe parce que tous les prestataires doivent apporter sa contribution dans ce rapport, on analyse et on fait un petit commentaire dans le rapport qu’on doit amener à la hiérarchie.

**P17: IA_01_AS_IT_03.rtf - 17:42 [Répondant : Nous nous faisons ..] (83:83)	(Super)**

Codes:	[SNIS_fonctionnement_Decrire_pdt MVE]

No memos

**Répondant** : Nous nous faisons là où j’étais et ici contions.

**P17: IA_01_AS_IT_03.rtf - 17:43 [Répondant : Là où j’étais il n..] (85:85)	(Super)**

Codes:	[SNIS_fonctionnement_Decrire_pdt MVE]

No memos

**Répondant** : Là où j’étais il n’y avait pas de cas pareil, on travaillait comme d’habitude.

**P17: IA_01_AS_IT_03.rtf - 17:44 [Répondant : Je souhaiterai qu’..] (91:91)	(Super)**

Codes:	[Souhait_Changement_gestion_futures_épidémies]

No memos

**Répondant** : Je souhaiterai qu’on prenne à compte tous les prestataires par ce qu’avec les tradis nous ont rendu un mauvais service, surtout que lorsque le malade souffrait de la maladie on les disait qu’il était empoisonné, on soigne alors que c’était Ebola. Si une autre épidémie arrivait je suggère qu’on prenne en compte tout le monde qui manipule un malade. Qu’ils soient dans la surveillance pour essayer de limiter la propagation de l’épidémie par ce que si on avait tenu compte de tous les prestataires et même privés et tradi on n’arriverait pas à cette situation qui était. Même dans les réunions ils nous disaient cela « que s’ils envoyaient les malades au CTE, ils vont vivre comment ? » Chose à changer ; la surveillance, il faut tenir compte des leaders communautaires par ce que se sont eux qui vivent avec la population, et savent comment maitriser la population ce qui a été contraire avec cette épidémie car il n’y avait pas l’implication de tout le monde. Lorsqu’on arrivait dans la communauté les chefs locaux n’étaient pas informés et c’était difficile de convaincre la communauté alors s’il était impliqué les choses passeraient sans difficulté.

**P17: IA_01_AS_IT_03.rtf - 17:45 [Répondant : Au début on avait ..] (93:93)	(Super)**

Codes:	[Organisation_travail-Circuit des malades_pendant MVE]

No memos

**Répondant** : Au début on avait impliqué certains IT, la sensibilisation est venue après, puisque les experts comme je l’ai dit étaient venus de tous les pays et ce sont eux qui allaient sur terrain, lorsqu’on ne voyait pas les prestataires locaux sur terrain avec les équipes de sensibilisations on se demandait d’où sont venus ces gens et c’est plus tard qu’ils ont compris qu’il fallait associer les locaux.

**P17: IA_01_AS_IT_03.rtf - 17:46 [Répondant : Il y a certaines s..] (95:95)	(Super)**

Codes:	[Commentaire-Recommandation_Répondant]

No memos

**Répondant** : Il y a certaines structures étatiques qui n’ont pas bénéficiées l’infrastructure comme celle-ci malgré la présence des partenaires, car ces derniers refusaient de construire en disant que c’était dans l’urgence mais d’autres arrivaient avec cette intention de transformer les structures pour répondre aux normes, ce que je suggère s’il arrivait une période qu’on ait des partenaires, que ces derniers gèrent de la même façon les structures. Je donne un exemple d’un partenaire comme ALIMA qui appui certaines structures, les malades mangent trois fois par jour, le frais de transport assuré et les soins sont gratuits. Dans d’autres structures où ALIMA n’intervient pas les malades ont tendance de se diriger vers les structures qui sont prise en charge par ALIMA et on se demandait que les structures prise en charge par le partenaire est étatique et les autres qui ne sont pas considérées sont étatiques, comment cette émulation alors que toutes ces structures sont publiques ?

**P17: IA_01_AS_IT_03.rtf - 17:47 [Répondant : Dans cette aire de..] (97:97)	(Super)**

Codes:	[Gratuité_Structures-Services_concernés]

No memos

**Répondant** : Dans cette aire de santé nous n’avons que ce centre de santé et dix-sept structure privées.

**P17: IA_01_AS_IT_03.rtf - 17:48 [Répondant : Pour maintenir ce ..] (99:99)	(Super)**

Codes:	[PCI_Comment_maintenir]

No memos

**Répondant** : Pour maintenir ce niveau nous souhaiterions que les partenaires continuent en nous aider en EPI par ce la population vulnérable que nous avons, c’est difficile de nous approvisionner dans les jours avenir en EPI mais si les partenaires continuaient à nous appuyer. Nous continuerons à garder ce niveau par ce qu’on est déjà habitué.

**P17: IA_01_AS_IT_03.rtf - 17:49 [Répondant : Il faut continuer ..] (101:101)	(Super)**

Codes:	[PCI_Comment_maintenir]

No memos

**Répondant** : Il faut continuer à capacité, car même les prestataires ne sont pas standard, il y a des nouveaux qui vont chaque fois venir des études, donc la capacitation est nécessaire.004058

**P17: IA_01_AS_IT_03.rtf - 17:50 [Répondant : Je ne peux que vou..] (103:103)	(Super)**

Codes:	[Commentaire-Recommandation_Répondant]

No memos

**Répondant** : Je ne peux que vous remercier car nous ne recevons pas de visiteurs de ces genres pour s’imprégner de ce qui se passe dans la contraint, c’est encourageant.

**P18: IA_01_AS_MDHPRIVE_02.rtf - 18:1 [Répondant : Je suis médecin gé..] (6:6)	(Super)**

Codes:	[Répondant_Responsabilité_habituelles]

No memos

**Répondant** : Je suis médecin généraliste bien sûr, je travaille ici depuis le mois d’octobre à cette structure centre médicaux chirurgical mapendo c’est depuis octobre que je suis ici mais avant cela j’étais en province d’ituri et cela que je travaillais bon comme je vous ai dit médecin généraliste c à d je fais tout je touche presque à tout ce ça.

**P18: IA_01_AS_MDHPRIVE_02.rtf - 18:2 [Répondant : Oui tout à fait je..] (8:8)	(Super)**

Codes:	[Répondant_Responsabilité_pdt MVE]

No memos

**Répondant** : Oui tout à fait je vécu Ebola depuis le mois de juillet le 15 juillet, c’est à ce moment-là qui suis venu m’imprégner de c’est qui ce passe dans le territoire avec Ebola par ce que en ituri on avait pas Ebola lorsque j’étais encore là c’était dans le territoire de Mambasa et Ebola est entré quand j’étais déjà sorti et à mon arriver dans c’est territoire j’ai commencé directement à travailler dans un centre de transit pour la prise en change de cas d’Ebola avant que je ne combine avec la prestation dans cette structure ici.

**P18: IA_01_AS_MDHPRIVE_02.rtf - 18:3 [Répondant : Bon au moment où j..] (10:10)	(Super)**

Codes:	[Considération_Ebola_population]

No memos

**Répondant** : Bon au moment où je suis arrivé je crois que la population avait déjà pris conscience sur la maladie Ebola mais, au début il y avait toujours de difficulté par ce que je me souviens j’avais fait un déplacement ici en pleine épidémie vraiment c’était compliquer chacun disait ce qu’il voulait sur l’épidémie et au début là on pensait que c’est une imagination, une création les agents de santé avaient amenés Ebola pour faire l’argent par ce que ça payé bien au début donc une année après, la population s’est rendue compte que vraiment c’est une maladie et toutes les normes que les agents de santé données à la population s’en est appropriée et on a constaté une diminution de cas et jusqu’au niveau ou nous sommes aujourd’hui on arrive à 0 cas après beaucoup de jour comme ça d’observation et je crois que c’est un effort qui est arriver quand la population s’était rendu compte que c’était une réalité par ce que à Butembo je crois que vous avez était à Butembo un moment donné les agents de la santé étaient accompagné par la police à Beni, ici ça se fait aussi il y a même dans certains quartier ici ou l’on a bruler les véhicules de l’OMS qui ne faisaient que de courses de service on a bruler par ce que il y avait une forte résistance on ne se pas de quelle origine, est ce que c’était politiser ou bien quelle était la base de cette intoxication on a pas bien visualiser cette histoire-là et voilà comment l’épidémie à trainer par ce que les gens ne s’étaient pas appropriés vraiment la maladie en question.

**P18: IA_01_AS_MDHPRIVE_02.rtf - 18:4 [Répondant : Tout à fait beauco..] (12:12)	(Super)**

Codes:	[Structures-Services_fonctionnement]

No memos

**Répondant** : Tout à fait beaucoup de structures n’ont pas bien fonctionnées, nous sommes parmi le rare donc je veux surtout mettre un accent sur les structures privées par ce que avec les structures de l’état toutes les interventions de la riposte se focaliser beaucoup plus sur les structures étatique mais, ce qui concerner les structures privées beaucoup on fermées nous sommes parmi le rares qui on survécut par ce que quand je suis arrivé ici avec 10 consultation par jour, 10 nouveaux cas disons même plus vous comptabilisé à la fin du mois 30 interventions 40 disons une moyenne de 28 interventions par mois, vous comprenez que on était entre de survivre quand même par rapport à d’autre. Mais beaucoup de structures avaient fermées par ce que comme vous le savez Ebola que nous avons vécu n’a pas était comme la fièvre hémorragique, on nous a différencier la maladie a virus Ebola et fièvre hémorragique comme nous on savaient Ebola avant donc dans la prise en charge cela a eu de conséquences, ça veut dire on va pas attendre que le patient aye l’hémorragie pour que on le notifier à la CTE ou bien dans la prise en chargé spécialiser il y avait les signes qui sont pratiquement les mêmes signes avec la fièvre typhoïde, avec le paludisme et automatiquement les patients ne voulaient pas nous fréquentés par ce que il vous fréquentes vous prélever tous ses signes là accompagner d’une fièvre vous notifier et vous envoyer au CT, on va dire vous êtes aussi complice tu veux nous faire mourir par ce que il nous envoie au agents de la riposte alors on va pas vous fréquenter et par conséquent vous allez fermer la porte ou en autre cas vos personnels ils vont aller se débrouiller dans la riposte, ils vont travailler la bas et vous-même responsable vous pouvez aussi trouver une place la bas vous fermer la porte vous travailler dans la riposte. Aussi donc c’est à ce point-là qu’on a eu un impact négatif sur les fonctionnements de structures dans cette prise en charge surtout dans cette période-là de la maladie à virus Ebola.

**P18: IA_01_AS_MDHPRIVE_02.rtf - 18:5 [Répondant : Ok la solution c’e..] (14:14)	(Super)**

Codes:	[Souhait_Changement_gestion_futures_épidémies]

No memos

**Répondant** : Ok la solution c’est la quelle, moi je pense que dans un 1^er^ temps il faut d’abord faire comprendre la population, lui montrer l’importance comment dirais-je donc la gravité de la pathologie il faut montrer la gravité de cette maladie-là qui arrive et la conscientiser pour que elle s’approprier les étapes, par ce que la prise en charge de la maladie à virus Ebola c’est à tout le niveau donc on parler de multisectorielle tout le monde intervient, la gestion de formation donc le journalistes sont là ils doivent bien joué leur rôle, il y a de psychologues il y a nous qui avons travaillés dans la prise en charge il y a d’autre dans la surveillance active alors tous ces domaines là il faut qu’ils parlent un même langage par ce que s’il y a un moindre contradiction alors tout reviennes à zéro, par ce que les agents de la riposte c’est sont attirés beaucoup d’ennuie seulement par ce que il y a eu une petite contradiction à leur façon d’intervenir quand il y a quelqu’un qui dit c’est positif l’autre dit non c’est ne pas encore positif et ça quand ça arrive au niveau de la population on dit oh ils sont entre de créer cette pathologie pour se faire l’argent c’est ça alors donc pour moi je pense que la coordination de cette riposte s’il y a une autre épidémie il faut que cette coordination soit vraiment solide et que ça soit multisectoriels, surtout employé ou bien utiliser des agents qui maitrise chacun son domaine par ce que ça aussi ça fait défaut ont prenaient de gens qui ne sont pas du domaine qui sont venus par ce que telle est mon frère et on lui met la bas c’était vraiment compliquer.

**P18: IA_01_AS_MDHPRIVE_02.rtf - 18:6 [Répondant : Bon je pense que p..] (16:16)	(Super)**

Codes:	[Structures-Services_fonctionnement]

No memos

**Répondant** : Bon je pense que pour répondre à cette question, je dirais que les services qui ont étaient vraiment touchés il y a la médecine interne, la pédiatrie oui surtout celle-là par ce que la maternité rouler encore bien par ce que ont voyaient rarement de femmes qui présentées de signes et qui pouvait faire à ce que on puisse les envoyées au niveau du CT. Rarement les femmes enceintes mais ça arrivait aussi qu’elles soient atteintes mais pas tellement. La chirurgie aussi fonctionnée bien, chirurgie très bien maternité bien mais pédiatrie et médicine interne là c’était compliquer.

**P18: IA_01_AS_MDHPRIVE_02.rtf - 18:7 [Répondant : Par ce que les sig..] (18:18)	(Super)**

Codes:	[Structures-Services_fonctionnement]

No memos

**Répondant** : Par ce que les signes sont plus lieu à cette catégorie de ce genre-là.

**P18: IA_01_AS_MDHPRIVE_02.rtf - 18:8 [Répondant : Bon juste au début..] (20:20)	(Super)**

Codes:	[Structures-Services_fonctionnement]

No memos

**Répondant** : Bon juste au début de l’épidémie si je me rappel bien au laboratoire on nous avez interdire de faire le TDR pour le paludisme par ce que là il faut minimiser vraiment la manipulation du sang, on avait interdire le TDR par ce que dans le programme national du paludisme le TDR pouvait se faire même ici dans mon bureau, je peux le faire sans problème ça on avait interdire et pour le structures qui transfusées par ce que comprenait ici nous avons même pas de banque de sang mais de transfusions se faisaient quand même dans beaucoup des structures et pour des structures qui transfusées on avait fermé la transfusion, donc la transfusion ne devrait que se passée à l’hôpital général de référence pas dans d’autres structures alors ça était aussi une handicape. Pour ces structures-là vraiment ces services là on avait frappé fort au début, c’est avec l’évolution de l’épidémie que on pouvait autoriser que l’on aller acheter du sang au niveau de la banque de sang et venir transfuser à la structure.

**P18: IA_01_AS_MDHPRIVE_02.rtf - 18:9 [Répondant : Je n’avais pas vu ..] (22:22)	(Super)**

Codes:	[Structures-Services_fonctionnement]

No memos

**Répondant** : Je n’avais pas vu un document mais quand on aller à la réunion de monitorage c’est là qu’on avait des instructions à partir de la zone de santé.

**P18: IA_01_AS_MDHPRIVE_02.rtf - 18:10 [Répondant : Oui nous pensons i..] (24:24)	(Super)**

Codes:	[Organisation_travail-Circuit des malades_pendant MVE_Effets sur la survie]

No memos

**Répondant** : Oui nous pensons impacter positivement peut être pour nous même aussi par ce que on pouvait aussi être affectés en manipulant le sang ça nous a aussi aidé à nous protéger oui je pense.

**P18: IA_01_AS_MDHPRIVE_02.rtf - 18:11 [Répondant : Bon je crois que a..] (26:26)	(Super)**

Codes:	[Prestataire_Pratique de Prévention contre MVE]

No memos

**Répondant** : Bon je crois que au début c’était une frustration tout le monde avait peur surtout qu’on savait que c’est une maladie mortelle mais quand on a compris que c’est ne pas tout le monde qui est entre de mourir par ce que ce qui nous avez fort réconforté au début là où la pathologie à commencer la maladie à virus Ebola il y avait 11 prestataires de santé qui étaient infectés on les a cantonner dans un lieu ils ont réussi le traitement que le professeur Muyembe lui-même a donné et personne entre eux ne morte d’Ebola certains ont développer la maladie mais d’autre n’ont même pas développer la maladie alors celle-là nous à réconforté et avec la multiplicité de formation que les partenaires données sur la protection par ce que au début là c’était d’abord la protection par ce que il y avait un principe qui disait se protéger d’abord avant de protéger l’autre alors c’est à ce point-là que nous avions vraiment donc le personnel était intéresser par beaucoup de formation et on s’était protégé pour essayer de protéger aussi d’autres gens c’était ça.

**P18: IA_01_AS_MDHPRIVE_02.rtf - 18:12 [Répondant : Non par ce que ava..] (28:28)	(Super)**

Codes:	[Triage_Considération_population]

No memos

**Répondant** : Non par ce que avant qu’on met celle d’ici il y avait seulement une installation en bâche la bas les gens ne se sont pas attaqué donc la 1^er^ conduite c’était quoi bien sûr on avait attaqué à hier, on a attaqué mais la 1^er^ réaction de la population c’était d’abord fuir cet hôpital quand on sent que on a mis un triage quelque part, on va vous fuir on commence à aller consister à hier là où il n’y a pas de triage mais quand ils ont constaté que on a mis le triage presque dans toutes les structures si c’est ne pas vraiment le vrai triage mais il y a quelque chose qui montre que las agents de l’Ebola qui passent par ici il y a juste à la porte lavage de mains et à l’époque il y avait même de spray pour sprayer la plante de pieds la chaussure tout ça pour combattre Ebola quand les gens ces sont rendu compte que c’est partout alors ils n’avaient pas à faire, ils ont adhéré facilement.

**P18: IA_01_AS_MDHPRIVE_02.rtf - 18:13 [Répondant : Au début non c’est..] (30:30)	(Super)**

Codes:	[Triage_comportement_patient]

No memos

**Répondant** : Au début non c’est par ce que ils avaient peur bien sûr de la maladie, non c’était il y avait une peur énorme vraiment par ce que les gens mouraient beaucoup moi-même j’ai été à la route quelque part chez un ami et dans une espace de trois heures de temps on pouvait compté facilement 15 croix qui passent vers la cimetière et dans l’espace de trois heures maintenant pour toute la journée ça pouvait être combien donc, les gens mouraient et cela était à la base de la peur qui était dans la population.

**P18: IA_01_AS_MDHPRIVE_02.rtf - 18:14 [Répondant : Bon je crois fonct..] (32:32)	(Super)**

Codes:	[Structures-Services_fonctionnement]

No memos

**Répondant** : Bon je crois fonctionnement ou bon dans les structures privées je crois qu’il faut différenciée 2, il y a ceux qui étaient partiellement ou bien totalement appuyés par bon est ce que c’est vraiment totale comme chez nous ici je ne pense pas ils nous appuie quand même un peu à ce qui concerne les intrants de la PCI contrôle des infections a cette aspect-là mais pris en charge c’est la structure elle-même qui intervient par ce que nous achetons nous même nos médicaments la différence il y a d’autre structures qui n’ont pas même cette appuie là sur l’aspect la prévention et contrôle des infections donc, eux-mêmes devraient achetée le gant par exemple je prends le gant par ce que pour chaque geste il faut utiliser le gant même nous sommes encore ici là où on a pas encore eu de cas suspect alors s’il faut placer vous voulez toucher un patient il faut avoir le gant au niveau de la salle de soins tout se fait avec le gant donc à la fin de la journée vous constatez que vous avez remplir tout un seau de gant, il fallait vraiment de soutient pour cela qui n’ont pas eu de soutient c’est sont ceux-là qui fermer facilement par ce que avec un grand risque de s’infecter. Alors dans les structures publiques chez eux tout était pris par l’état il y avait un de subvention chez le personnel on encourage le personnel on donne le médicament on donnait aussi des intrant pour la prévention au contrôle de réflexion la totalement tout était pris en charge et je peux souligné à ce point-là, que c’est pendant cette épidémie d’Ebola que nous avons compris l’importance de travaillé dans une structure étatique, nous avons étaient vraiment différent de ceux-là qui travaillent dans une structure étatique sur le point fonctionnement et pris en charge du personnel tout tout tout vraiment différent.

**P18: IA_01_AS_MDHPRIVE_02.rtf - 18:15 [Répondant : Ça nous ne savons ..] (34:34)	(Super)**

Codes:	[Gratuité_Structures-Services_concernés]

No memos

**Répondant** : Ça nous ne savons pas ça dépendait de partenaire.

**P18: IA_01_AS_MDHPRIVE_02.rtf - 18:16 [Répondant : Bon je suis arrivé..] (36:36)	(Super)**

Codes:	[Ressources_humaines_Disponibilité et perturbations]

No memos

**Répondant** : Bon je suis arrivé quand mes prédécesseurs m’ont rencontrés ce qui s’est passé ici aussi nous n’avons pas étaient épargner donc, au début de l’épidémie le deux premiers mois ou bien disons le trois premiers mois les gens avaient peur par ce que ça ne payé même pas mais quand il y a eu l’argent on est venu payer d’un coup comme ça on vous paye 1200 dollars ou bien combien de dollars donc sa dépendait de l’endroit où vous êtes positionné quand les gens ont compris ça il y a eu tendance tout le monde a eu tendance à aller travailler dans la riposte et laisser ou abandonner son poste et c’est ce qui se fait observer dans toute presque dans toute les structures mais il y avait quand même ce qui trainer toujours la charge était grande et mais comme il y a toujours et la riposte n’a pas pris tout le monde d’autres sont restés dans des structures pour continués avec le soins de routines dans des structures mais je croix que le partenaires avaient sentis aussi cette pression-là par ce que tout le monde voulait quitter alors ils ont privilégiés aussi par structure privée ou pas ils ont pris autant de personnes ici j’avais appris à mon arrivé avant moi on avait pris 8 personnes

**P18: IA_01_AS_MDHPRIVE_02.rtf - 18:17 [Répondant : nous sommes 22, il..] (38:38)	(Super)**

Codes:	[Ressources_humaines_Disponibilité et perturbations]

No memos

**Répondant** : nous sommes 22, ils avaient pris 8 qui signé dans leur cahier et pour être rémunéré par le partenaire la fin du mois cela pour éviter que ces gens-là puissent aller à hier alors après je ne sais pas combien de mois ils ont réduits ils sont revenu à 5 personnes selon quel critère nous on ne savait pas je crois que c’était leur stratégie pour essayer de garder encore plus de personnel dans les hôpitaux que d’allé se retrouvaient tous dans la riposte

**P18: IA_01_AS_MDHPRIVE_02.rtf - 18:18 [Répondant : Je pense que nous ..] (40:40)	(Super)**

Codes:	[Qualité_soins_Prestataires]

No memos

**Répondant** : Je pense que nous avons beaucoup amélioré cette période de l’épidémie à virus Ebola nous a permis d’amélioré beaucoup d’aspect dans notre façon de prendre en charge les patients et je crois que eux-mêmes le témoignent avant la riposte vous serez d’accord avec moi que l’homme noir si vous dit moins dans nos contrait si vous mettez le gant vous voulez lui soigné vous voulez lui mettre une injection vous mettez le gant il va se dire mais se monsieur il me prend comment il pense que je suis ou je veux mourir toute suite ou je quel maladie de quelle origine mais avec la maladie à virus Ebola que les gens ont compris que c’est maintenant la nouvelle façon je crois que vous êtes entre de circulé dans des hôpitaux vous allez comprendre que quand vous entré on a pas la difficulté de distinguer un infirmier ou un agent de santé qui travaille dans cette structure avec quelqu’un qui est venu visité ou rendre visite à un malade donc quand vous entrez vous sentez déjà que l’habillement à changer et dans la prise en charge aussi c’est la même chose par ce que ça nous a appris à développer vraiment un raisonnement quand nous prenons en charge les patients d’abord vous devrait mettre en évidence est ce que ce monsieur ici est ce que il n’a pas Ebola et le triage est là pour ça si il y a autant de signe on le met en isolement en attendant qu’on appelle les gens pour venir investigués après investigation on peut valider pour amener au CT , on peut invalider le cas on l’amène maintenant à l’hôpital il va prendre le soins et je crois vraiment que ça nous a aider.

**P18: IA_01_AS_MDHPRIVE_02.rtf - 18:19 [Répondant : Tout dépendait de ..] (42:42)	(Super)**

Codes:	[Qualité_Soins_Patients]

No memos

**Répondant** : Tout dépendait de la manière dont ont les abordaient nous sommes devenue doux avec l’épidémie à virus Ebola donc on a appris au moins comment on peut consoler, aborder un patient surtout dans de stress comme celle-là.

**P18: IA_01_AS_MDHPRIVE_02.rtf - 18:20 [Répondant : Donc il faut aller..] (44:44)	(Super)**

Codes:	[Structure-Service_Utilisation_population]

No memos

**Répondant** : Donc il faut aller bien sûr il y a un service qui travaillé sur ça et les accompagnateurs psychologique mais comment un monsieur un malade qui est dans votre structure qui reçoit souvent le soins chez vous quelqu’un viendra d’une autre aire de santé est ce que c’est celui-là qui va lui convaincre il faut d’abord que vous vous puissiez lui convaincre pour que celui-là puisse trouver un terrain bien lisse il y a plus de résistance comme ça il va lui récupérer facilement le patient donc c’est ce qu’ont faisaient.

**P18: IA_01_AS_MDHPRIVE_02.rtf - 18:21 [Répondant : Bon la gratuité es..] (48:48)	(Super)**

Codes:	[Gratuité_Structures-Services_concernés]

No memos

**Répondant** : Bon la gratuité est là, elle nous touche bien sûr par ce que la fréquentation ne reste pas la même il y a des gens quelqu’un peut venir en pensant que nous sommes aussi entre de donné de soins gratuit mais quand il va trouvait que nous nous soignons et nous demandons de l’argent il va dire non je pars là où il y a la gratuité mais ils ne sont pas nombreux par ce que en matière de santé quelqu’un veut être soigné par une personne à laquelle il met confiance surtout ça.

**P18: IA_01_AS_MDHPRIVE_02.rtf - 18:22 [Répondant : Bon les rumeurs co..] (50:50)	(Super)**

Codes:	[Gratuité_Rumeurs]

No memos

**Répondant** : Bon les rumeurs cours par ci par là mais on essaye de trouver un peu des solutions à cela pour des gens qui peuvent parler de cela donc qui peuvent exprimer librement leur votre présence par ce que quand on donne les soins gratuits c’est ne sont pas les vrais médicaments. C’est ça la rumeur autour de la gratuité de soins ils pensent que c’est sont de faux médicament alors on est là un peu pour les rassurés par ce que je me souviens je viens même d’envoyer un autre patient là à l’hôpital générale je lais seulement supplier il voulait résister je dis non non allais-y n’est pas peur il y a aussi de médecins comme nous il y a des infirmiers comme vos infirmiers qui vous soignes ici et en plus il y a la gratuité là-bas vous n’aurez pas a vous tourmenter à la fin concernant la facture et c’est comme ça qu’on essaye de le convaincre toujours .

**P18: IA_01_AS_MDHPRIVE_02.rtf - 18:23 [Répondant : Non chez nous non ..] (52:52)	(Super)**

Codes:	[Gratuité_Structures-Services_concernés]

No memos

**Répondant** : Non chez nous non nous ne sommes pas concerné nous on fait payer.

**P18: IA_01_AS_MDHPRIVE_02.rtf - 18:24 [Répondant : La diminution de l..] (56:56)	(Super)**

Codes:	[Gratuité_Conséquences_utilisation_services]

No memos

**Répondant** : La diminution de la fréquentation ça nous touche vous voyez depuis que vous êtes là je ne pas de patient mais avant c’est ne te pas comme ça.

**P18: IA_01_AS_MDHPRIVE_02.rtf - 18:25 [Répondant : Non il y a pas vra..] (58:58)	(Super)**

Codes:	[Médicaments_disponibilité_fosa]

No memos

**Répondant** : Non il y a pas vraiment eu de changement il n’y a que si je me rappel bien dipirone , le produit comme dipirone qu’on avait retiré au marché que les agents utilisaient en cachette mais qu’on a frappé vraiment d’interdiction quand Ebola est arrivait par ce que ça pouvait cacher la fièvre à la maison quelqu’un prend dipirone ou bien dans une structure elle fait la fièvre on le cache là-bas on lui donne dipirone et à la fin le test devient positif ça nous causer du tort alors. C’est seulement ce produit là qu’on avait frappé d’interdiction mais pour les autres…

**P18: IA_01_AS_MDHPRIVE_02.rtf - 18:26 [Répondant : Oui certains et po..] (60:60)	(Super)**

Codes:	[Médicaments_disponibilité_fosa]

No memos

**Répondant** : Oui certains et pour les autres produits c’était rester très bien.

**P18: IA_01_AS_MDHPRIVE_02.rtf - 18:27 [Répondant : Oui c’était dispon..] (62:62)	(Super)**

Codes:	[Médicaments_disponibilité_fosa]

No memos

**Répondant** : Oui c’était disponible sur le marché.

**P18: IA_01_AS_MDHPRIVE_02.rtf - 18:28 [Répondant : Avant la maladie d..] (64:64)	(Super)**

Codes:	[SNIS_fonctionnement_Decrire_pdt MVE]

No memos

**Répondant** : Avant la maladie donc l’épidémie c’était bien, le mapipi et autre ça s’est transmettre mais pendant l’épidémie non.

**P18: IA_01_AS_MDHPRIVE_02.rtf - 18:29 [Répondant : Il y a des équipes..] (66:66)	(Super)**

Codes:	[SNIS_fonctionnement_Decrire_pdt MVE]

No memos

**Répondant** : Il y a des équipes de recherche active qui passent chaque jour. Ils sont même ce matin d’autres comme vous êtes là ils sont venu pour le contrôle et autre chose, ils passent chaque jour c’est sont cela qui rapporte au niveau de partenaire et au niveau de la coordination de la riposte.

**P18: IA_01_AS_MDHPRIVE_02.rtf - 18:30 [Répondant : On ne ramène plus...] (68:68)	(Super)**

Codes:	[SNIS_fonctionnement_Decrire_pdt MVE]

No memos

**Répondant** : On ne ramène plus.

**P18: IA_01_AS_MDHPRIVE_02.rtf - 18:31 [Répondant : Non peut-être on v..] (70:70)	(Super)**

Codes:	[SNIS_fonctionnement_Decrire_pdt MVE]

No memos

**Répondant** : Non peut-être on va reprendre après l’épidémie.

**P18: IA_01_AS_MDHPRIVE_02.rtf - 18:32 [Répondant : Ok avec l’épidémie..] (74:74)	(Super)**

Codes:	[Souhait_Changement_gestion_futures_épidémies]

No memos

**Répondant** : Ok avec l’épidémie à maladie de virus Ebola du moins celle que nous avons vécu il y a eu un problème au début par ce que on a travaillé beaucoup plus avec les ex parts qu’avec les locaux c’est ça ce qui était à la base de la résistance par ce que quelqu’un je veux vous donner seulement un seul exemple c’était à mangina là ou l’épidémie à commencer j’avais un ami chauffer qui m’a dit que ce qui s’est fait ça ne devrait pas se faire on est entre de tué nos frères il y a un ministre qui vient de Kinshasa avec de soi-disant infirmiers ou docteurs entre gieme je ne connais pas leur vrais identité mais on met dit qu’ils s’appelaient toujours docteur entre eux est ce que c’est sont de médecins ou biens des infirmiers ou bien des agents de santé vraiment on les envois dans un village qui ne parle que swahili ou bien une dialecte locale le chauffer va arriver il ne sait même pas toquer à la porte pour s’exprimer alors ils ont arrivé dans une famille là où ils pouvaient enquêté à un fait difficulté de s’exprimer ils rentrent dans la voiture ils commencent à complété le papier là il créer de données et a 14h ils rentrent a la coordination alors que ça ne reflète pas la vraie réalité du terrain et celle-là par ce que ils utilisaient de gens qui sont venu ailleurs sans initient les autochtones les autochtones devraient normalement orienté tout ça on entre ici si il s’agit d’interpréter on va interprète mais au début c’est ne te pas ça , c’est ce qui a fait que la population doute de la réalité qui était dans cette épidémie. Alors donc si prochainement cela arrivait par ce que l’approche devrait être d’abord de convaincre la base donc les locaux travaillés avec eux pour bien gérer l’épidémie et que le cas soit vite pris en charge je pense ce mon point de vue.

**P18: IA_01_AS_MDHPRIVE_02.rtf - 18:33 [Répondant : Ça c’est une quest..] (76:76)	(Super)**

Codes:	[Souhait_Changement_gestion_futures_épidémies]

No memos

**Répondant** : Ça c’est une question difficile, quelque chose en termes de.

**P18: IA_01_AS_MDHPRIVE_02.rtf - 18:34 [Répondant : Bon sur ce point l..] (78:78)	(Super)**

Codes:	[Souhait_Changement_gestion_futures_épidémies]

No memos

**Répondant** : Bon sur ce point la moi je pense que le remède seulement c »est de se mettre ensemble avec tout le monde dans de situation comme celle-là , il fallait appeler tout le monde autour d’une table et que les gens s’attendent, on peut se dire non quelle est la meilleure façon de gérer l’épidémie qui viens de nous frapper le gens peuvent se mettre et échanger et tout ce qui devrait se vivre dans cette situation là ça ne devrait pas être mal interpréter soit par le privé soit par les gens de structures étatique mais quand seulement ce sont les structures de l’état qui sont invitée à la zone ou bien à la sous coordination pour décider ou bien discuter d’un point et concernant la riposte là c’est complique. Après la riposte normalement on devrait encore former les gens que nous sommes à la fin de l’épidémie comment devrons nous nous comporté après l’épidémie pour éviter qui ait une autre plan B dans le pays ou bien dans un coin normalement c’est ça la stratégie qui devrait avoir lieu malheureusement je ne sais pas si ils ont entré d’y penser par ce que on a dit qu’on v déclarer la fin de l’épidémie en Mai.

**P19: IA_01_AS_PCOSA_02.rtf - 19:1 [Répondant : D’abord le travail..] (8:8)	(Super)**

Codes:	[Répondant_Responsabilité_habituelles]

No memos

**Répondant** : D’abord le travail de la vie je suis enseignant à l’école mais dans l’aire de santé je suis président de l’aire de santé de kanzulinzuli.

**P19: IA_01_AS_PCOSA_02.rtf - 19:2 [Répondant : La maladie est ent..] (10:10)	(Super)**

Codes:	[Début-Progression_Epidémie MVE]

No memos

**Répondant** : La maladie est entrée en provenance de mangina là où il y a eu les 1^er^s cas hors notre centre est à proximité, notre aire de santé est tout proche de l’entrée de Beni donc c’est au centre ou les gens de mangina peuvent arrivaient est ce comme ça qu’Ebola est entré. Le 1^er^ cas à commencer à Ndindi et le gens n’étaient pas informer, ils ont refusé complètement, ils ne s’avaient pas que ça pourrait être Ebola par ce que il y avait trop de rumeur et ils ont dit que si Ebola était à Mangina ce par ce que il y a une personne qui avait mangé un chat d’un vieux homme, que c’était une sorcellerie alors les gens n’ont pas bien compris que c’était Ebola et c’est là a fait à ce que Ebola se propage trop par ce que surtout à l’aire de santé de Kanzulinzuli il y a eu beaucoup de cas.

**P19: IA_01_AS_PCOSA_02.rtf - 19:3 [Répondant : Les gens ont beauc..] (12:12)	(Super)**

Codes:	[Considération_Ebola_population]

No memos

**Répondant** : Les gens ont beaucoup pensaient par ce que jusque maintenant ils continuent toujours à pensés de choses non fondée, il pensait que c’est la sorcellerie et si c’est la sorcellerie autre disait que c’est quelque chose qu’on nous a envoyé ici d’autant plus que on était dans une guerre et massacre qui n’est pas connue on égorge le gens alors ils vus que c’est une autre forme de massacrés le gens et cela à causer la mort à beaucoup de gens par ce que ils ont mis dans leur tête que c’était autre chose ils n’ont pas compris que c’était une maladie.

**P19: IA_01_AS_PCOSA_02.rtf - 19:4 [Répondant : Et ces gens-là, te..] (14:14)	(Super)**

Codes:	[Considération_Riposte_Population]

No memos

**Répondant** : Et ces gens-là, tellement que c’était une épidémie je pourrais pensé que c’est la première épidémie qui est arrivé ici et le déploiement de gens, et il y a de docteurs qui sont venus de pays étrangers alors ils ont mystifiés et nous la population nous avons vu que c’est une business par ce que ils viennent cherchés de l’argent vu le montant qu’ils recevaient, le fait de loué un véhicule pour 100 dollars par jour c’était beaucoup et on disait que ça c’est ne pas normal ce que c’est quelque chose créée pour que ces gens puisse gagnés de l’argent beaucoup d’argent.

**P19: IA_01_AS_PCOSA_02.rtf - 19:5 [Répondant : Oui en tout cas le..] (16:16)	(Super)**

Codes:	[Structures-Services_fonctionnement]

No memos

**Répondant** : Oui en tout cas le service de santé à partir de cette période que Ebola est venue il y a eu beaucoup de changement, d’abord d’une manière générale les centres de santé étaient moins fréquentés. les gens n’ont pas voulus se faire soigner au centre de santé publique ils partaient aux structures privées et surtout il fréquentés le tradi-praticien ils pensaient que c’est là où il y a le médicament mais on a trouvé dans notre aire de santé les structures privées et les structures tradi-praticiens nous ont beaucoup causés tort par ce que c’est là où il y a eu contamination de toute sorte par ce que ils tiennent pas de mesure , pas de propreté donc rien pour le protéger et dalleurs beaucoup sont décèdes dans cette situation par ce qu’il manipule les gens sans s’avoir s’ils souffrent de l’Ebola mais ils ont étaient atteints et ils sont mort malgré le traitement que le tradi-praticiens le proposer et cela nous a déranger. Et autre chose qui n’a pas marcher et que lorsqu’ils ont commencés à dire que pendant l’épidémie il y a plus d’urgence donc si tu es malade personne ne peut s’occuper de toi par ce qu’il faut d’abord prendre beaucoup de mesures de précaution. beaucoup des malades mourraient dans des ambulances la plus part de fois pour dire qu’on commence à se préparer, donc il y avait plus urgence a de chose tous les cas étaient de suspicion il y avait personne pour dire c’est cas on connait on peut soigner non mais, dans un premier temps à la 1^er^ année les professionnels de santé sont mort, le fait d’accueillir un nouveau malade qui arrive sans mesure de précaution exemple ils n’ont pas le gant et si ils pouvaient en avoir leurs épis n’était pas suffisant pour que ils travaillent. Ça c’est parmi le méfait que nous avons vu et ce qui a fait que beaucoup de gens meurent, donc lorsqu’une personne est malade et comme ils étaient habitués à bien accueillir les malades mais lorsque cela est fini pendant cette période épidémiologique on ne toucher plus le malade comment avant on peut le voir, le tempérer et pas prélever les TA. Le fait de ne pas toucher n’importe comment le malade c’est ce qui a protégé le professionnels de santé, ils ont compris que c’est grave.

**P19: IA_01_AS_PCOSA_02.rtf - 19:6 [Répondant : La vaccination à c..] (20:20)	(Super)**

Codes:	[Service_vaccination_Fonctionnement]

No memos

**Répondant** : La vaccination à continuer mais il n’y a pas une chose importante que les gens ont accepté, donc depuis qu’on avait commencé avec la vaccination d’Ebola il n’y a pas vraiment de choses qu’on marchées. Ceux-là qu’on donner le vaccin de routine c’est les enfants qui étaient à la maternité puisqu’ils étaient facile à trouvés mais pour dire que les sites ont fonctionnés normalement en tout cas à un moment donné ça n’a pas marché.

**P19: IA_01_AS_PCOSA_02.rtf - 19:7 [Répondant : Le laboratoire éta..] (22:22)	(Super)**

Codes:	[Service_laboratoire_fonctionnement]

No memos

**Répondant** : Le laboratoire était opérationnel mais je parle de l’aire de santé plus précisément dans notre centre de santé le laboratoire n’a jamais fermé les portes, mais seulement il y a des choses qu’on a faits mais qui ne pas habituelle et avec la gratuité de soins il y a beaucoup de maladies qu’on a dit négliges comme par exemple l’hypertension, diabète etc… le corps médical ne se faisait plus souci de ça pendant cette période.

**P19: IA_01_AS_PCOSA_02.rtf - 19:9 [Répondant : Tout le monde ne p..] (26:26)	(Super)**

Codes:	[Qualité_Soins_Patients]

No memos

**Répondant** : Tout le monde ne peut pas être satisfait de la même façon, il y a celui qui vient en état très malade et les étapes qui suivent devient longues au point qu’ils se plaignent que les médecins ne se soucie plus d’eux, ils ne distinguent pas un cas grave ils voient comme si tout est normal. C’est ça la plainte que nous avons nous la population, la communauté nous voyons qu’ils ne s’occupent pas de ceux qui sont à l’agonie.

**P19: IA_01_AS_PCOSA_02.rtf - 19:10 [Répondant : L’accueil, d’abord..] (24:24)	(Super)**

Codes:	[Qualité_soins_Prestataires]

No memos

**Répondant** : L’accueil, d’abord il y avait beaucoup d’engouement lorsque ils ont amené la gratuité de soins, par ce que le soins était payant mais quand la gratuité à commencer les gens étaient nombreux alors ils ont changés les structures pour recevoir le visiteur car on a instauré le système de triage et pour le triage le malade s’exprime mais il n’avait pas le temps de touché son carnet donc après l’avoir testé et être tempérer, celui qui est au service de triage te guide et t’oriente dans un 1^er^ temps c’était bien avec PDSS, ceux-là qui avaient le soins c’était quand même bien et je peux dire que les prestataires étaient soulagés dans un 1^er^ temps par ce que leur salaire était entré de venir et ils étaient entré de maximisé le cas et c’est ce qui les a aider, ils maximisaient aussi le fiches perdue en faisant le suivis pour que tout se passe dans une voie normale et ceux-là nous a beaucoup aidé au niveau de centres de santé et aux structures.

**P19: IA_01_AS_PCOSA_02.rtf - 19:11 [Répondant : C’est ce que la co..] (28:28)	(Super)**

Codes:	[Triage_Considération_population]

No memos

**Répondant** : C’est ce que la communauté ne comprenait pas bien par ce que tu trouves que lorsqu’on parle de lavage de mains ça devient comme si on force la personne a se lavée les mains alors que l’eau est disponible mais ils trouvent ça comme une colonie et d’autres disent que le chlore le dérange et ça crée une démangeaison, mais ils n’étaient pas habitués et pour le moment quand je me promène sur la route il y a de point ou se lavé les mains mais tu vas trouver une personne qui se lave les mains comme si il n’a pas envie de le faire pour qu’il arrive au triage et les mains sont mouillée mais il ne se lave pas comme il se doit, c’est ce qui est lien de chose d’avant. Alors il y a ceux-là qui n’ont pas encore compris que Ebola c’est une maladie eux pense que c’est quelque chose qu’on a envoyé et surtout que si la maladie n’a pas encore arrivé touchée la famille ou s’il y a pas une personne morte avec la maladie dans la famille ou un proche, il ne peut pas comprendre que c’est une maladie.

**P19: IA_01_AS_PCOSA_02.rtf - 19:12 [Répondant : D’abord plusieurs ..] (30:30)	(Super)**

Codes:	[Prestataire_Attitude_devant_malade]

No memos

**Répondant** : D’abord plusieurs médecins lorsqu’ils ont remarqué qu’il y a l’argent dans la riposte certains ont abandonnaient leur poste pour aller là où il y a beaucoup l’argent ça c’était le 1^er^, lorsqu’ils ont trouvé qu’il y a quelque chose pour leur survie, ils ont cumulé de passé la nuit chaque jour au sein de leurs structures mais la journée ils travaillent à la riposte pour la présence mais au niveau du centre il change l’horaire pour travailler chaque nuit.

**P19: IA_01_AS_PCOSA_02.rtf - 19:13 [Répondant : Certainement.] (32:32)	(Super)**

Codes:	[Prestataire_Attitude_devant_malade]

No memos

**Répondant** : Certainement.

**P19: IA_01_AS_PCOSA_02.rtf - 19:14 [Répondant : Ceux qui sont au c..] (34:34)	(Super)**

Codes:	[Prestataire_Attitude_devant_malade]

No memos

**Répondant** : Ceux qui sont au centre commençaient à se lamenter que les autres sont payé à la riposte et au niveau de leurs structures. C’est le 1ere chose qui fait à ce qu’ils ne soignent pas avec une bonne conscience, ils disent que les autres sont entre de le volé et de fois ils se bagarrent entre eux donc tu vas trouver que ça dérange au niveau de la structure, ils se lamentaient pour l’horaire par ce qu’ils sont habitués travaillé la nuit alors que d’autre ne viennent pas la nuit donc ça créer de conflits entre eux dans le travail.

**P19: IA_01_AS_PCOSA_02.rtf - 19:15 [Répondant : Surtout la permane..] (36:36)	(Super)**

Codes:	[Ressources_humaines_Disponibilité et perturbations]

No memos

**Répondant** : Surtout la permanence par ce que tu peux être permanant aujourd’hui matin après un jour tu viens le soir, tu trouves que les autres sont pénalisés par ce que l’horaire est perturbé alors que c’est leur travail qu’ils faisaient dans la routine.

**P19: IA_01_AS_PCOSA_02.rtf - 19:16 [Répondant : Oui il y avait bea..] (38:38)	(Super)**

Codes:	[Ressources_humaines_situation_grève]

No memos

**Répondant** : Oui il y avait beaucoup de grevé donc avant Ebola c’était une catastrophe, nous sommes au centre général mais un médecin gradé comme IT trouvé 50 dollars c’était un problème hors c’est sont de responsable et tu vas remarquer que le dernier gagne 15 dollars, beaucoup d’année que les autres ne trouvaient plus leurs primes donc ils se contentaient de 20 dollars qu’ils gagnent ici alors ça donner de remords à tel point qu’ils détestaient même le travail et regrettés d’avoir choisi ce métier à cause de cette structure, mais à partir du moment que Ebola est venue ils se retrouvaient et gagnés quelque chose et ça les a beaucoup aidé.

**P19: IA_01_AS_PCOSA_02.rtf - 19:17 [Répondant : La gratuité à comm..] (40:40)	(Super)**

Codes:	[Gratuité_Début]

No memos

**Répondant** : La gratuité à commencer à la même période avec Ebola.

**P19: IA_01_AS_PCOSA_02.rtf - 19:18 [Répondant : Un moment donné lo..] (42:42)	(Super)**

Codes:	[Gratuité_Interruption]

No memos

**Répondant** : Un moment donné lorsque les projets des autres venaient, mais il y a toujours gratuité. Donc lorsque PDSS est passé par ce qu’il y a environs une année, il y avait tendance que les gens commencent à payer par ce qu’il y avait insuffisance financier et on se demander comment le centre va fonctionner ont disaient de payé même un cent franc mais ça n’avait pas dure par ce que les gens ne comprenaient pas puis on a eu une chance à notre centre on trouve MSF qui travaille avec nous et qui a pris la chose en main il a fait retourner la gratuité. Mais pour certains qui avait commencer la gratuité, ils amènent le cas comme une personne qui a le paludisme par ce que il présente le mêmes signes que celui qui a Ebola et là c’est gratuit mais pour les autres maladie tu dois payer mais ça n’a pas duré longtemps ils ont trouvés que les conditions dans lesquelles vivent les gens et comme ils avancent toujours d’arguments soit disant il y a massacre il y a pas moyen la population est démunie donc si on dit qui est de choses gratuites devienne payable la population ne sera pas en mesure.

**P19: IA_01_AS_PCOSA_02.rtf - 19:19 [Répondant : Il y avait de sens..] (44:44)	(Super)**

Codes:	[Gratuité_Information_population]

No memos

**Répondant** : Il y avait de sensibilisations et une émission à la radio qui disait au gens de ne pas se caché par ce que beaucoup se caché dans de maisons, on disait au lien de vous cacher dans de maisons, venez au centre le médicament et les soins sont gratuit et lorsque tu arrives et comme le gens sont compliqué ils disaient que ce qui est gratuit ne pas important

**P19: IA_01_AS_PCOSA_02.rtf - 19:20 [Répondant : Oui ils disaient q..] (46:46)	(Super)**

Codes:	[Gratuité_Rumeurs]

No memos

**Répondant** : Oui ils disaient qu’on le donné deux comprime et moi lorsque j’attends de rumeur on y va avec la personne pour qu’elle me montre celui qui la donner ce deux comprimer par ce que le fait que le gens étaient nombreux ils donnaient les soins même pour trois jours et après trois jours tu reviens pour voir s’il n’y a pas eu un changement mais, plusieurs pensaient ils ont conçu de façon qu’ils pensent que les comprimés ne guéris pas et c’est ça la guerre que nous avons ici chez nous. Les structures privées n’ont pas eux la notion de prescription rationnelle, un malade arrive on lui met une seringue il fait de mélange de multivitaminé on met dans le sérum ça donne une couleur, c’est là que le malade se dit qu’il a était soigné mais au centre le piquier sont très rares alors ils pensent que au niveau du centre il y a pas un bon traitement par ce que le patient ne voit pas la présence de sérum et beaucoup de seringue.

**P19: IA_01_AS_PCOSA_02.rtf - 19:21 [Répondant : Oui] (48:48)	(Super)**

Codes:	[Gratuité_Interruption]

No memos

**Répondant** : Oui

**P19: IA_01_AS_PCOSA_02.rtf - 19:22 [Répondant : Très peu, par ce q..] (50:50)	(Super)**

Codes:	[Gratuité_Interruption]

No memos

**Répondant** : Très peu, par ce que quand il y avait projet ce qui le soutenez pourrait dire bon, notre projet se termine par ici alors il se disait bon qu’est-ce qu’on va faire encore, les gens sont encore malade mais les plaidoiries passent toujours si la zone de santé travail sur ça elle dit la population ne saura pas payée là vous voulez dire que le gens meurent par ce que ils ne sont pas en mesure de payé il y a toujours les gens qui viennent lorsque le projet des uns se terminent d’autres commencent

**P19: IA_01_AS_PCOSA_02.rtf - 19:23 [Répondant : Le médicament étai..] (52:52)	(Super)**

Codes:	[Médicaments_disponibilité_fosa]

No memos

**Répondant** : Le médicament était vraiment disponible, puisque moi je suis dans la boite en tout cas le médicament était disponible par ce que à partir de fonctionnement pendant la période de l’Ebola quand même les structures qui gardées bien ces choses elles ont un stock qui est vraiment valable.

**P19: IA_01_AS_PCOSA_02.rtf - 19:24 [Répondant : Oui parce que s’il..] (54:54)	(Super)**

Codes:	[Gouvernance_soins santé]

No memos

**Répondant** : Oui parce que s’il y a une bonne gestion les choses pourraient être bien.

**P19: IA_01_AS_PCOSA_02.rtf - 19:25 [Répondant : Non il n’y a pas e..] (56:56)	(Super)**

Codes:	[Médicaments_disponibilité_fosa]

No memos

**Répondant** : Non il n’y a pas eu de rupture par ce que la rupture était ravitaillée on disait qui est alerte de stock, donc on disait d’alerté avant que le stock ne finisse

**P19: IA_01_AS_PCOSA_02.rtf - 19:26 [Répondant : Pour nous les tenu..] (58:58)	(Super)**

Codes:	[Réunion_pdt-épidémie-Ebola]

No memos

**Répondant** : Pour nous les tenues de réunions n’ont pas bien fonctionnée pourquoi je dis cela c’est par ce que dans de structures ou l’ont faisaient de réunions comme je le dis ici c’était l’épicentre souvent on commencer d’avoir peur d’arriver au centre donc tu trouves partout il y a le gens, donc il y avait beaucoup de réunions et il y avait plusieurs gens donc le centre était beaucoup risqué pendant l’épidémie mais on avait quand même le temps de nous appeler en tant que individu président représentant de la communauté pour prendre presque toutes les décisions qui devraient être prises puis ce que de fois je devrais être parmi le mandataire, même si c’est leur argent qui vont aller retirer, j’étais parmi le mandataires qui allés retirer.

**P19: IA_01_AS_PCOSA_02.rtf - 19:27 [Répondant : Il n’y avait pas e..] (60:60)	(Super)**

Codes:	[Réunion_pdt-épidémie-Ebola]

No memos

**Répondant** : Il n’y avait pas eu souvent de réunions mais, on était moins nombreux lorsque on se rassembler à cause de la peur et des beaucoup des rumeurs par ce que les gens avaient tendance d’organisé des marches pour juguler ces choses. Lorsqu’il y avait beaucoup de cas par ce que souvent ils brulés les structures alors on était là, et ce qui a fait à ce que notre structure ne puisse pas être brulée c’est par ce qu’elle était construite par l’état et notre chef de quartier il a était souple dans cette histoire comme il est encore trop jeune et il a parvenu à contourner beaucoup de choses qui pourrait nous arriver même ces gens venaient d’autre dénoncé leur plan et on les arrêtés avant qu’l ne fasse quelque chose.

**P19: IA_01_AS_PCOSA_02.rtf - 19:28 [Répondant : Donc si moi j’aler..] (62:62)	(Super)**

Codes:	[Agression-Menaces_Structure-Prestataire_raisons]

No memos

**Répondant** : Donc si moi j’alerte pour dire le patient se trouve quelque part, s’ils savent que c’est moi qui es alerté il faut qu’ils brûlent ma maison ou bien là où je donnais l’information.

**P19: IA_01_AS_PCOSA_02.rtf - 19:29 [Répondant : Non de structures ..] (64:64)	(Super)**

Codes:	[Structures-Services_fonctionnement]

No memos

**Répondant** : Non de structures privées, plusieurs structures privées sont fermées donc c’était après consensus. Donc si les gens deviennent nombreux après avoir donné un conseil, on n’amené pas des épis et autres choses ces structures ont le fermées.

**P19: IA_01_AS_PCOSA_02.rtf - 19:30 [Répondant : Plusieurs étaient ..] (68:68)	(Super)**

Codes:	[Structures-Services_fonctionnement]

No memos

**Répondant** : Plusieurs étaient de ce côté par ce que, vers Mangina ou il y avait beaucoup de cas, c’était de structures privées et ils ont tendances de soignées comme le tradi-praticien.

**P19: IA_01_AS_PCOSA_02.rtf - 19:31 [Répondant : Comme la propreté ..] (72:72)	(Super)**

Codes:	[Changements_depuis_Ebola]

No memos

**Répondant** : Comme la propreté et le lavage de mains ça on n’est te pas habituer les gens négligés ça mais, aujourd’hui ils connaissent déjà qu’il faut se laver les mains. Je pense que c’est la pauvrette qui fait à ce que on ne puisse pas avoir le lavabo chez soi mais, on est en train de sensibiliser pour que chaque maison, chaque ménage soit à mesure d’avoir un point de lave main chez soi je pense cela vas beaucoup nous aider.

**P19: IA_01_AS_PCOSA_02.rtf - 19:32 [Répondant : A part ça le fait ..] (74:74)	(Super)**

Codes:	[Changements_depuis_Ebola]

No memos

**Répondant** : A part ça le fait de se saluer ça diminué un peu mais, avec la présence de corona le gens ne se salués plus mais on peut le faire par distance et se déjà important.

**P19: IA_01_AS_PCOSA_02.rtf - 19:33 [Répondant : Depuis qu’il y a E..] (76:76)	(Super)**

Codes:	[Changements_depuis_Ebola]

No memos

**Répondant** : Depuis qu’il y a Ebola il y avait plus solidarité mais au moment de deuil ça posé problème pour dire aux gens de ne pas participé au deuil c’était encore un problème. Il faut beaucoup des sensibilisations pour que ça finisse et que les gens comprennent.

**P19: IA_01_AS_PCOSA_02.rtf - 19:34 [Répondant : Je pense non par c..] (78:78)	(Super)**

Codes:	[PCI_Comment_maintenir]

No memos

**Répondant** : Je pense non par ce que nos prestataires sont de négligent c’est eux qui vont commencer le non-respect de toutes ses mesures, par ce que certains prestataires commencés à soignés sans porter le gant d’autant plus que il y a aussi de rupture de gant au centre tellement que on était déjà habituer à des choses gratuites au niveau de centre, je sais que lorsque cette gratuité va se terminée les ONG vont quitter les aires de santé et tout ça va prendre fin par ce que ils vont manquer les épis et ou le trouvé car ils ne peuvent pas accepté donner leur argent pour en avoir par ce que il y aura déjà de calcul pour savoir il y a quelle recette je pense ça pourra pas continuer selon moi.

**P19: IA_01_AS_PCOSA_02.rtf - 19:35 [Répondant : Il faut une organi..] (80:80)	(Super)**

Codes:	[PCI_Comment_maintenir]

No memos

**Répondant** : Il faut une organisation qui sera à partir de nos bureaux centraux par ce que si le gens le comprennent et le mettent dans leur activités ça peut continuer mais, ce qui est grave est que eux aussi ne sont pas moindre et je ne sais pas comme faire cette histoire vous qui faites de recherche vous pouvez peut être les aidés par ce que au Congo notre l’état est fragile, même si tu demandes dalleurs pour nous les agents de l’état ils disent qu’ils n’ont pas d’intérêt dans ces histoires c’est eux qui sont presque pénaliser puis tout est contrôler par leur hiérarchie alors, ils sont là comme s’ils n’ont pas une pourcentage dans ces affaires car on se croiser souvent pour leur 10% qu’ils trouvent ici mais lorsque la gratuité avait commencée il y a de partenaire qui disaient de ne plus le donner le 10% par ce que eux aussi ont leur parts la bas mais, ils ne se contentent jamais ils ont l’habitude de détourné plusieurs projet pour que ils se retrouvent alors si ça ne continue pas ce que c’est l’état qui sera responsable.

**P19: IA_01_AS_PCOSA_02.rtf - 19:36 [Répondant : Ce vrai on ne sava..] (82:82)	(Super)**

Codes:	[Souhait_Changement_gestion_futures_épidémies]

No memos

**Répondant** : Ce vrai on ne savait rien c’est la 1^er^ épidémie alors ils importent presque toute la technicité , tous les techniciens sont venus d’ailleurs peut être nos professionnels de santé qui se trouvent ici pourraient s’en sortir mais, notre main d’œuvre était négliger donc s’il y a une nouvelle épidémie l’autre main d’œuvre peuvent venir aider mais que ça ne soit pas beaucoup privilégié plus que la nôtre, par ce que entre eux il y avait de mésentente soit disant moi aussi je peux être a mesure de gérée cette situation mais il y a un expatrié qui vient le faire et cela déranger beaucoup. Nous attendons de rumeurs qui y a un docteur assassiné à butembo, il se pourrait que ceux qui étaient dans le même circuit que lui c’est eux même qui l’avait assassiné.

**P19: IA_01_AS_PCOSA_02.rtf - 19:37 [Répondant : Je ne sais pas ce ..] (84:84)	(Super)**

Codes:	[Commentaire-Recommandation_Répondant]

No memos

**Répondant** : Je ne sais pas ce que je peux ajouter si c’est ne pas de remercier, même si Ebola est venu quand même la riposte nous aide ça nous apprend beaucoup de choses et je pense si un autre arrive les gens seront conséquent.

**P20: IA_01_AS_PCOSA_03.rtf - 20:1 [Répondant : Souvent je vois qu..] (11:11)	(Super)**

Codes:	[Répondant_Responsabilité_habituelles]

No memos

**Répondant** : Souvent je vois quelque problème de santé pour la population dans le cadre de soins, comment nous pouvons avoir de bon centre de santé et autre chose qui cadre avec la santé.

**P20: IA_01_AS_PCOSA_03.rtf - 20:2 [Répondant : Dans l’aire de san..] (15:15)	(Super)**

Codes:	[Début-Progression_Epidémie MVE]

No memos

**Répondant** : Dans l’aire de santé mabolio Ebola est venue ici suite au gens qui quittaient leurs aires de santé pour venir ici à l’aire de santé de Mabolio.

**P20: IA_01_AS_PCOSA_03.rtf - 20:3 [Répondant : Bon la communauté ..] (21:21)	(Super)**

Codes:	[Considération_Ebola_population]

No memos

**Répondant** : Bon la communauté pensait que Ebola c’était un montage, c’est après qu’Ebola est entrée dans l’aire de santé lorsqu’ils l’ont vu avec leurs propres yeux c’est à ce moment-là qu’ils ont compris que c’était réellement la maladie et parmi il y a ceux qui n’ont pas compris facilement et d’autres ont compris lorsqu’ils ont vu la maladie entrée dans la communauté.

**P20: IA_01_AS_PCOSA_03.rtf - 20:4 [Répondant : D’autres disaient ..] (23:23)	(Super)**

Codes:	[Considération_Ebola_population]

No memos

**Répondant** : D’autres disaient que peut-être ils sont en train de chercher l’argent c’est pour quoi ils font le montage d’Ebola

**P20: IA_01_AS_PCOSA_03.rtf - 20:5 [Répondant : C’était par exempl..] (25:25)	(Super)**

Codes:	[Agression-Menaces_Structure-Prestataire_raisons]

No memos

**Répondant** : C’était par exemple le relais communautaire orienter le malade au centre de santé lorsqu’il arrive au centre de santé on constate que le malade est un cas suspect, on l’envoi au CTE pour passer les examens et si ça passe positif et par mal chance le malade décédait, la communauté va attaquer le gens qui ont orienter le malade que c’est grâce a cette équipe que le malade trouve la mort.

**P20: IA_01_AS_PCOSA_03.rtf - 20:6 [Répondant : Ils ont continué à..] (27:27)	(Super)**

Codes:	[Agression-Menaces_Structure-Prestataire_raisons]

No memos

**Répondant** : Ils ont continué à faire leur travail par ce qu’ils savent bien que la communauté est dans l’ignorance ils se sont forcé pour continuer à travailler

**P20: IA_01_AS_PCOSA_03.rtf - 20:7 [Répondant : Non c’est ça surto..] (29:29)	(Super)**

Codes:	[Considération_Ebola_population]

No memos

**Répondant** : Non c’est ça surtout que la communauté développait, mais le relais non pas baisser le bras ils ont continué à sensibiliser.

**P20: IA_01_AS_PCOSA_03.rtf - 20:8 [Répondant : Ce que je trouvais..] (31:31)	(Super)**

Codes:	[Structures-Services_fonctionnement]

No memos

**Répondant** : Ce que je trouvais comme changement dans des activités que le service de riposte est venu avec c’est qui à changer et c’est qui nous a beaucoup aidé qui diminue certaines maladies c’est la propreté. J’ai trouvé les centres n’avaient pas la notion de propreté et surtout la communauté était contaminée là où elle avait souvent l’habitude de fréquenter, le centre et depuis l’arrive de l’Ebola avec le service de PCI j’ai trouvé qui y a eu un changement et ça diminue d’autres maladie de maux de ventre.

**P20: IA_01_AS_PCOSA_03.rtf - 20:9 [Répondant : Oui je vus un chan..] (33:33)	(Super)**

Codes:	[Structures-Services_fonctionnement]

No memos

**Répondant** : Oui je vus un changement lorsque leS relais sensibilisaient le gens et ils sont pris une décision de partie au centre de santé avant le temps est-ce que la présence d’Ebola na pas fait peur au gens de partir au centre de santé ?

**P20: IA_01_AS_PCOSA_03.rtf - 20:10 [Répondant : Avant ils avaient ..] (36:36)	(Super)**

Codes:	[Considération_CTE_population]

No memos

**Répondant** : Avant ils avaient peur par ce que lorsque la personne arrivait on l’envoie au CTE directement la communauté pense que cette personne souffre et elle va mourir de cette maladie d’Ebola et tout c’était par ignorance.

**P20: IA_01_AS_PCOSA_03.rtf - 20:11 [Répondant : Avant ils avaient ..] (34:34)	(Super)**

Codes:	[Considération_CTE_population]

No memos

**Répondant** : Avant ils avaient peur mais aujourd’hui ils ont compris

**P20: IA_01_AS_PCOSA_03.rtf - 20:12 [Répondant : Par ce qu’ils ont ..] (38:38)	(Super)**

Codes:	[Considération_CTE_population] [Référence-contre-référence_fonctionnement]

No memos

**Répondant** : Par ce qu’ils ont trouvé pendant la période de l’Ebola il y avait beaucoup de transferts qu’avant cette période, les centres faisaient beaucoup de transfert de malades par rapport à la période avant Ebola et si tu pars au centre on te transfert directement au CTE.

**P20: IA_01_AS_PCOSA_03.rtf - 20:13 [Répondant : C’est pour qu’un m..] (40:40)	(Super)**

Codes:	[Référence-contre-référence_fonctionnement]

No memos

**Répondant** : C’est pour qu’un malade arrive au CTE qu’il passe à l’examen pour avoir un résultat sur avant de le soigner.

**P20: IA_01_AS_PCOSA_03.rtf - 20:14 [Répondant : Ils n’ont pas aimé..] (42:42)	(Super)**

Codes:	[Référence-contre-référence_fonctionnement]

No memos

**Répondant** : Ils n’ont pas aimé c’est pourquoi nous avons continué avec de sensibilisations pour sensibiliser les familles.

**P20: IA_01_AS_PCOSA_03.rtf - 20:15 [Répondant : Oui comme le labor..] (44:44)	(Super)**

Codes:	[Service_laboratoire_fonctionnement]

No memos

**Répondant** : Oui comme le laboratoire on nous avait dit d’arrêter avec ce service et nous avons sensibilisé les malades par ce que s’il vient avec l’idée de faire l’examen on lui fait comprendre

**P20: IA_01_AS_PCOSA_03.rtf - 20:16 [Répondant : il n’y avait pas l..] (46:46)	(Super)**

Codes:	[Traitement_Invasifs_Prestataire_changement]

No memos

**Répondant** : il n’y avait pas le laboratoire et autre chose nous avons diminué les injections mais ce n’était pas un grand problème.

**P20: IA_01_AS_PCOSA_03.rtf - 20:17 [Répondant : Il y avait une aut..] (48:48)	(Super)**

Codes:	[Qualité_Soins_Patients]

No memos

**Répondant** : Il y avait une autre difficulté du côté de l’accueil par ce qu’avant lorsqu’un malade arrivait au centre de santé l’infirmier sautait sur le malade et s’occupait de lui directement mais lorsque il arrive aujourd’hui au centre l’infirmier commencer à se préparer et là il y aura un retard peut-être il trouve que le malade à beaucoup de signes dans c’est cas là l’infirmier na va pas lui approcher sans se protéger et pendant ce temps que l’infirmier se prépare le malade va se sentir mal et lorsque il va voir l’infirmier en tenue, il va dire que donc il va mourir. Donc le système d’accueillir le malade a changé.

**P20: IA_01_AS_PCOSA_03.rtf - 20:18 [Répondant : Oui mais à la fin ..] (50:50)	(Super)**

Codes:	[Considération_thermoflash_population]

No memos

**Répondant** : Oui mais à la fin de compte ils sont parvenus à comprendre, mais c’est qui à créer une résistance c’était le thermo flash, ils étaient habitués au thermomètre normal mais lorsqu’ils ont trouvé ça ils ont pensé que peut être lorsque on utilise le thermo flash ça peut absorber leur intelligence ils ont beaucoup commenté à cause de ça mais aujourd’hui ils on compris que c’est parmi aussi les mesures de protection.

**P20: IA_01_AS_PCOSA_03.rtf - 20:19 [Répondant : Ils ont pensé que ..] (52:52)	(Super)**

Codes:	[Considération_thermoflash_population]

No memos

**Répondant** : Ils ont pensé que ça va soutirer leur intelligence sur la tête, il n’y a pas d’autres choses à part ça.

**P20: IA_01_AS_PCOSA_03.rtf - 20:20 [Répondant : C’est qui posait b..] (56:56)	(Super)**

Codes:	[Triage_Considération_population]

No memos

**Répondant** : C’est qui posait beaucoup de problème c’était le triage et l’isolement, surtout l’isolement a posé beaucoup de problème par ce que lorsque tu mets une personne dans l’isolement, là il se sent mal il pense que vous l’avez déjà confirmé et qu’il va mourir de l’Ebola. Il reste là entre de souffrir et ce qui sont resté à la maison n’aura pas de l’espoir il va dire aussi qu’il va mourir d’Ebola, mais on n’a pas eu peur on a continué à les sensibiliser par ce qu’il fallait qu’ils comprennent la situation laquelle on traversait.

**P20: IA_01_AS_PCOSA_03.rtf - 20:21 [Répondant : On cause avec leur..] (58:58)	(Super)**

Codes:	[Triage_Considération_population]

No memos

**Répondant** : On cause avec leur famille on faisait ça souvent.

**P20: IA_01_AS_PCOSA_03.rtf - 20:22 [Répondant : S’il a Ebola on lu..] (60:60)	(Super)**

Codes:	[Triage_Considération_population]

No memos

**Répondant** : S’il a Ebola on lui soigne et s’il n’a pas Ebola on lui soigne pendant 4 jours il rentre à l’aire de santé et on lui envoi le résultat de l’aire de santé soit disant le malade que tu as envoyé on voit ça.

**P20: IA_01_AS_PCOSA_03.rtf - 20:23 [Répondant : Ils quittaient là-..] (62:62)	(Super)**

Codes:	[Considération_CTE_population]

No memos

**Répondant** : Ils quittaient là-bas avec un raisonnement différent que ceux qui sont laissé à la communauté, il va dire là il y a rien je suis parti on ma soigner le laboratoire à dit que je ne pas Ebola j’avais une malaria on m’avait soigné et je suis guéris, mais ceux qui sont resté à la maison pensent qu’on lui a donné de l’argent c’est pourquoi il ne veut pas dire la vérité ou le secret qui se passe là-bas.

**P20: IA_01_AS_PCOSA_03.rtf - 20:24 [Répondant : Et s’il y a un mor..] (64:64)	(Super)**

Codes:	[Considération_CTE_population]

No memos

**Répondant** : Et s’il y a un mort dans la communauté on appelle l’équipe de riposte et quand elle va arriver on prend 2 ou 3 personnes de la famille de la victime eux aussi s’habillent, par ce qu’ils entrent là-bas en pensant qu’on enlève un organe sur le cadavre et lorsqu’ils vont sortir tout en sachant que on n’enlève pas un organe à un cadavre. Alors leur penser par rapport avec ceux qui sont reste ne sera pas la même, ce qui sont restés vont penser qu’on les a donnés l’argent raison pour laquelle ils disent autre choses. Par ce que ces gens ont vu que ce n’est ne pas ce qu’on dit à l’extérieur qui se passe ici, après avoir vu la verité sur le travail que ce qui disent qu’on enlève l’organe au cadavre est faux, et les autres disent comme on vous a donné l’argent c’est pour quoi vous ne voulez pas dire la verité. Ces gens ne reculaient pas ils ont continué à sensibiliser.

**P20: IA_01_AS_PCOSA_03.rtf - 20:25 [Répondant : Pour la gratuité d..] (66:66)	(Super)**

Codes:	[Gratuité_Accueil_population]

No memos

**Répondant** : Pour la gratuité de soins au début ils avaient de doute, ils pensaient que ces sont les médicaments qui viennent dans le programme d’Ebola, alors nous avons envoyé le relais pour sensibiliser et enlève cette mauvaise mentalité à la communauté afin qu’elle comprend pour fréquenter le centre.

**P20: IA_01_AS_PCOSA_03.rtf - 20:26 [Répondant : Bon il y arriver s..] (68:68)	(Super)**

Codes:	[Qualité_Soins_Patients]

No memos

**Répondant** : Bon il y arriver surtout par ce qu’avant on ne demandait pas le numéro de téléphone et autre chose quand on est en train de demander, ils commencent à se demander où est-ce qu’on va amener mon nom ou mon numéro par ce qu’ils ont trouvé qu’avant on ne demander pas tout ça, tu arrives tu écris seulement ton nom et on te soigne directement tu rentres ou si tu seras hospitalisé on ne va pas te demander beaucoup de chose. Mais aujourd’hui on demande beaucoup de chose comme par exemple si tu n’avais pas été au deuil et cela lui fait perdre la mémoire et le sens où il vous donne un faux numéro, le nom qui n’est pas le leur vraiment c’était un travail difficile pour nous le fait de suivre le malade pour découvrir la vérité qu’il va vous dire. Et autre le malade cacher le signe de peur qu’on l’amener au CTE et s’il y a trois choses qui ne va pas chez lui il va te dire une seule chose.

**P20: IA_01_AS_PCOSA_03.rtf - 20:27 [Répondant : Vraiment ici à mab..] (70:70)	(Super)**

Codes:	[Ressources_humaines_Disponibilité et perturbations]

No memos

**Répondant** : Vraiment ici à mabolio ils ne sont pas partis si cela était fait a hier mais ici au structure ils sont resté mais ils ont beaucoup souffert par ce que il y avait beaucoup de travail.

**P20: IA_01_AS_PCOSA_03.rtf - 20:28 [Répondant : Ils ont beaucoup s..] (72:72)	(Super)**

Codes:	[Ressources_humaines_Disponibilité et perturbations]

No memos

**Répondant** : Ils ont beaucoup souffert comme pour le service qui quittait la sous coordination et la zone de santé, ces services entrait et les partenaires aussi venaient mais ils étaient en train de nous faciliter.

**P20: IA_01_AS_PCOSA_03.rtf - 20:29 [Répondant : Oui] (74:74)	(Super)**

Codes:	[Réunion_pdt-épidémie-Ebola]

No memos

**Répondant** : Oui

**P20: IA_01_AS_PCOSA_03.rtf - 20:30 [Répondant : Bon il y avait un ..] (76:76)	(Super)**

Codes:	[Réunion_pdt-épidémie-Ebola]

No memos

**Répondant** : Bon il y avait un moment ou nous étions trop pris dans le programme de riposte et les sensibilisations nous ont emportées vraiment on ne respectait pas le canevas de comite par ce que la maladie était au pic et on avait arrêté un moment donné.

**P20: IA_01_AS_PCOSA_03.rtf - 20:31 [Répondant : C’était une périod..] (78:78)	(Super)**

Codes:	[Réunion_pdt-épidémie-Ebola]

No memos

**Répondant** : C’était une période ou la maladie était maitrisée, mais dans une période notre aire de santé était attaqué et les gens sont morts, mais nous nous sommes mis dans le travaille pour sensibiliser mais la communauté nous a bloquer pour faire d’autres activités.

**P20: IA_01_AS_PCOSA_03.rtf - 20:32 [Répondant : Bon mon travail ét..] (80:80)	(Super)**

Codes:	[Répondant_Responsabilité_pdt MVE]

No memos

**Répondant** : Bon mon travail était d’orienter tous les services dans le terrain, s’il y aura un service qui s’occupe de l’eau propre et construire de tank d’eau. Lorsque OXFAM est venu, je m’occupais de tout ça. Si IRC vient je dois m’occuper de la propreté des structures, donc j’étais entre d’aider le gens dans tout ça et les équipes qui entrer au terrain je les orienté dans tout le domaine.

**P20: IA_01_AS_PCOSA_03.rtf - 20:33 [Répondant : C’est PDCC.] (82:82)	(Super)**

Codes:	[Gratuité_Partenaire d'appui]

No memos

**Répondant** : C’est PDCC.

**P20: IA_01_AS_PCOSA_03.rtf - 20:34 [Répondant : Bon PDCC et IRC au..] (84:84)	(Super)**

Codes:	[Gratuité_Partenaire d'appui]

No memos

**Répondant** : Bon PDCC et IRC aussi.

**P20: IA_01_AS_PCOSA_03.rtf - 20:35 [Répondant : PDC] (86:86)	(Super)**

Codes:	[Gratuité_Partenaire d'appui]

No memos

**Répondant** : PDC

**P20: IA_01_AS_PCOSA_03.rtf - 20:36 [Répondant : IRC surtout nous a..] (88:88)	(Super)**

Codes:	[Gratuité_Partenaire d'appui]

No memos

**Répondant** : IRC surtout nous a appuyé dans le cadre de la PCI depuis le début jusque maintenant il n’y avait aucun problème.

**P20: IA_01_AS_PCOSA_03.rtf - 20:37 [Répondant : Oui] (90:90)	(Super)**

Codes:	[Gratuité_Partenaire d'appui]

No memos

**Répondant** : Oui

**P20: IA_01_AS_PCOSA_03.rtf - 20:38 [Répondant : Ici il y avait de ..] (92:92)	(Super)**

Codes:	[Gratuité_Evolution]

No memos

**Répondant** : Ici il y avait de période ou les choses changeaient et il dit on va pas prendre un tel service comme par exemple la maternité ne sera pas dans le contrat et on se dit on lieu de facture le malade dans une période critique on les aidés comme ça mais il se force toujours pour aider la communauté.

**P20: IA_01_AS_PCOSA_03.rtf - 20:39 [Répondant : Mais aussi il y a ..] (94:94)	(Super)**

Codes:	[Gratuité_Structures-Services_concernés]

No memos

**Répondant** : Mais aussi il y a un problème qui nous est arrivé lorsque PDCC à amener une méthode ou on doit demander a tout le monde la carte d’électeur et le numéro de téléphone mais nous avons trouvé que suivre les gens dans cette situation ils aurons la peur mais on sait forcer travailler sans demander la photocopie de carte d’électeur pour éviter qui est panique mais lorsque PDSS est venu évaluer notre travail il la trouvait qu’il y a pas le copie de photocopie de la carte d’électeur il a annuler le travail que nous avons fait par ce que nous sommes arrivé à un niveau qu’on se dit acheter une photocopieuse pour éviter de faire déplacer le malade qui ne se pas l’importance d’une photocopieuse. Nous avons acheté une photocopieuse au centre de santé pour sensibiliser le gens de venir avec leur carte d’électeur on photocopie la carte au centre pour respect les conditions que PDSS nous a données.

**P20: IA_01_AS_PCOSA_03.rtf - 20:41 [Répondant : Non] (98:98)	(Super)**

Codes:	[Médicaments_disponibilité_fosa]

No memos

**Répondant** : Non

**P20: IA_01_AS_PCOSA_03.rtf - 20:42 [Répondant : Oui] (96:96)	(Super)**

Codes:	[Médicaments_disponibilité_fosa]

No memos

**Répondant** : Oui

**P20: IA_01_AS_PCOSA_03.rtf - 20:43 [Répondant : Surtout j’aurais a..] (100:100)	(Super)**

Codes:	[Positif_pdt MVE]

No memos

**Répondant** : Surtout j’aurais aimé certaines méthodes qu’on a placé pour l’épidémie qui est et qui pourra venir que ces méthodes ne puissent pas changer.

**P20: IA_01_AS_PCOSA_03.rtf - 20:44 [Répondant : Comme la méthode d..] (102:102)	(Super)**

Codes:	[Positif_pdt MVE]

No memos

**Répondant** : Comme la méthode de la propreté, lavage des mains et la propreté aux centres de santé et cela doit continuer pour nous protéger à d’autres menaces qui pourra attaquer la communauté, ça va aussi nous aider pour certaines maladies comme la diarrhée.

**P20: IA_01_AS_PCOSA_03.rtf - 20:45 [Répondant : Bon c’est qui peut..] (104:104)	(Super)**

Codes:	[Souhait_Changement_gestion_futures_épidémies]

No memos

**Répondant** : Bon c’est qui peut être changé c’est lorsqu’il y avait une résistance, c’était pour le visiteur, une personne quitte un endroit pour venir travailler dans une aire de santé ou il ne pas connu et cela poser beaucoup de problème. Et aujourd’hui nous sommes maintenant si c‘est relais communautaire que dans l’aire de santé avec une personne qui est connue à sa cellule, ça vraiment il faut que ça change, on peut être appuyé avec d’autres gens mais surtout qu’ils ne soient de personnes qui sont connu pas le gens de la communauté et s’il contenu il n’y aura pas de problème.

**P20: IA_01_AS_PCOSA_03.rtf - 20:46 [Répondant : Il y a n’a pas.] (106:106)	(Super)**

Codes:	[Commentaire-Recommandation_Répondant]

No memos

**Répondant** : Il y a n’a pas.

**P21: IA_01_AS_PCOSA_04.rtf - 21:1 [Répondant : Je suis le préside..] (6:6)	(Super)**

Codes:	[Répondant_Responsabilité_habituelles]

No memos

**Répondant** : Je suis le président de comité de santé d’abord à l’aire de santé de Malepe mais da la zone de santé de Beni je suis représentant de président du comité de santé dans le comité de gestion de la zone de santé de Beni.

**P21: IA_01_AS_PCOSA_04.rtf - 21:2 [Répondant : Bon étant que repr..] (8:8)	(Super)**

Codes:	[Répondant_Responsabilité_habituelles]

No memos

**Répondant** : Bon étant que représentant du comité de santé, mon travail est de récupéré des difficultés qui sont dans des aires de santé puis cherche une solution entre nous les présidents par ce que le dossier peut arriver à une personne, tellement que l’autre peut voir de nouvelle dans ce dossier et il lui donne une piste de solution. Et à part ça dans la communauté il y a des choses qu’on garde, nous savons que nous avons de médicament par ce que au centre de santé nous avons le gens et l’argent, c’est ça ce que nous voyons.

**P21: IA_01_AS_PCOSA_04.rtf - 21:3 [Répondant : D’abord dans notre..] (10:10)	(Super)**

Codes:	[Début-Progression_Epidémie MVE]

No memos

**Répondant** : D’abord dans notre zone de santé de Beni quand nous avons attendu l’arrivée d’Ebola dans la zone de santé de Mabalako, nous avons attendu aux informations à la radio qu’il y a une personne qui est morte à Mangina mais il a fait sortie le sang. Tellement que Mangina est dans la zone de Mabalako et il est à coté de Beni, nous ici à Beni on a pris ça comme une sorcellerie. Après cette personne nous avons vu un autre cas venir qu’une personne est morte. Après les experts ont apportaient un communique à la zone de santé, déjà le médecin chef de zone à convoque une réunion et ils ont appelé tout le monde à l’hôpital général ou ils nous à donner l’information qui disait que ce n’est ne pas la sorcellerie qui est dans la zone de santé de Mabalako mais nous sommes attaqués par une maladie qu’on appelle Ebola.

**P21: IA_01_AS_PCOSA_04.rtf - 21:4 [Répondant : Quand Ebola est en..] (12:12)	(Super)**

Codes:	[Début-Progression_Epidémie MVE]

No memos

**Répondant** : Quand Ebola est entré à Beni précisément dans la zone de santé de Beni, c’est l’aire de santé qui était touché et c’était l’aire de santé de Buchili précisément a une famille qu’on appelle famille Kapanza. C’est cette famille qui a amené Ebola à Beni, directement les équipes quand on a commencé le travail on nous a partagé et comme je suis président ils m’ont dit que je dois être au service de communication, déjà nous avons commencé à aller donner le message à la communauté que ce n’est ne pas une sorcellerie mais c’est une maladie qui entre ici chez nous et si on ne tient pas le garde ça peut nous exterminer dans l’ignorance.

**P21: IA_01_AS_PCOSA_04.rtf - 21:5 [Répondant : Communauté pour ac..] (14:14)	(Super)**

Codes:	[Considération_Ebola_population]

No memos

**Répondant** : Communauté pour accueillir Ebola d’abord, Ebola est nouveau pour nous dalleurs toute la communauté à attendue ça à la radio et télévision à Guinée qui a une maladie qu’on appelle Ebola. Puis on a attendu que Ebola arrive chez nous et contenue de la situation, on était en insécurité dans notre zone de santé et dans cette période la population n’avait pas confiance au leader surtout nos politiciens. Déjà la communauté a dit que Ebola ce n’est ne pas une maladie mais un montage de politiciens, par ce que comme le massacre n’arrive pas à finir la population de Beni a parement ils amènent Ebola pour tuer les gens, c’est pour ça que la population disait que c’est un montage politique. En quittant là-bas lorsque les activités de l’équipe de riposte ont commencé, la population avait dit que la façon dont elle voit Ebola que c’est un business le fait que la population trouve qui a présence de beaucoup de véhicules et presque tous les véhicules étaient très cher, la population a dit que ça c’est un business. La population à constater encore qu’il y a une étape qu’une personne part à la CTE on valide le cas mais derrière lui il remarque que on décharge son cas et puis PAM donne la nourriture à cette personne. La population s’est dit quel hôpital ou après les soins on donne la nourriture. On a trouvé que ça c’est un problème, mais comme on était dans le terrain le relais communautaire tout le monde se donner, nous avons dit non, si on vous donne cette nourriture c’est ne pas pour vous tuer mais cette nourriture montre que pendant le temps que tu étais suivi au CTE tu n’avais pas le moyen d’aller au champ et tu n’as pas la force mais comme tu n’as pas la force ce pourquoi on te donne cette nourriture pour que tu reviennes en forme. La population commencer à comprendre la situation lentement puis ils ont fait sortir une étape qu’on appelle engagement communautaire, déjà lorsqu’on a fait entre ça dans la communauté nous avons parlé de cette maladie et leur montré que c’est ne pas un commerce mais pour que ça finisse ici chez nous il faut que tout le monde enlève cette maladie au concerner mais qu’il se prenne comme si c’est bien lui qui est malade et puis il se pose une question de savoir qu’est-ce qu’il doit faire pour que cette maladie soit éradique par ce que si tout le monde se met dans l’engagement communautaire il doit commencer à surveiller son voisin, lorsque un voisin manifeste un signe on appelle directement le numéro vert et s’il y a celui qui est entre de vomir voir même si la personne a la diarrhée observe seulement si il va présenter deux signe . La population déjà à commencer à alerté on se dit ok , nous sommes arrivé à un niveau ou les malades deviennent nombreux au CTE mais le cas confirmer leur taux à baisser on sait dit vraiment que l’engagement communautaire tient le coup et lorsque on était là on les a montres cette stratégie qu’on appelle : vigilance toujours dans l’engagement communautaire, de que vous quitté hors de la zone de santé de Beni vous entré ici, les gens disent celui-ci est venu tel part déjà on informe le chef, on informe aussi l’équipe de riposte qu’il y a des visiteurs dans l’aire de santé, c’est comme ça que le taux était entre de baisser.

**P21: IA_01_AS_PCOSA_04.rtf - 21:6 [Répondant : Donc attaquer les ..] (18:18)	(Super)**

Codes:	[Agression-Menaces_Structure-Prestataire_raisons]

No memos

**Répondant** : Donc attaquer les centres de santé le premier tour, vous voyez lorsque la maladie arrive, elle est venue avec les signes de la fièvre déjà le partenaire on dit non à la fièvre, faisons la distribution de moustiquaire , quand ils ont fait la distribution de moustiquaire il fallait que les acteurs soient nombreux dans le communauté puis le fait d’appelé le gens à la ligne tellement que à la riposte pour fuir cette maladie Ebola on disait éviter le contacte mais quand ils vont aller aux lignes il y aura déjà contact et on ne sait pas d’où est venu chacun puis nous avons pris une stratégie de distribuer le moustiquaire ménage par ménage lorsque ils arrivent au rond-point ou il y a dix ménages tu arrives et tu distribue le moustiquaire. alors le partenaire avait fait un retard pour le payement de ces agents et cette équipes commençait à se demander pourquoi on ne nous paye pas notre argent et ça un peu perturber les têtes, tellement comme il y veut de t-shirt et il réclame l’argent alors il est arrivé un moment une personne est venu avec une idée pour dire comment ces gens veulent retenir notre argent il parait que c’est IT qui détient notre sommes, a parement c’est le président et l’IT qui font à ce que on ne puisse pas nous payé. Déjà ils viennent se décharger aux structures et après vous aller attendre qu’ils ont démolies un centre et le bruler directement, mais tout le monde s’est demandé ce qui se passe mais lorsque on les a donnés leur somme la situation aussi a changé.

**P21: IA_01_AS_PCOSA_04.rtf - 21:7 [Répondant : Quand Ebola était ..] (20:20)	(Super)**

Codes:	[Structures-Services_fonctionnement]

No memos

**Répondant** : Quand Ebola était entré la 1^er^ idéologie était la distance entre les gens et lorsque la communauté à eu cette information elle a eu peur d’ailleurs l’équipe cadre de la zone de santé avait dit directement que les gens attendent d’abord les histoires là de dire CPN vous pouvez le faire mais il faut voir la distance qui est entre le gens, que les gens ne puisse pas se toucher entre eux , comme on amène l’enfant a la CPS il faut que l’enfant soit protéger l’infirmier doit porter le gant et après avoir utilisé le gant il enlève pour porter d’autre gant par ce que l’enfant on sait pas si il a était en contact avec qui ou bien soit l’infirmier était en contact avec qui dans l’angle de protéger le concerné mais toutes ces activités continuées à fonctionner .

**P21: IA_01_AS_PCOSA_04.rtf - 21:8 [Répondant : Tous les centres d..] (22:22)	(Super)**

Codes:	[Structures-Services_fonctionnement]

No memos

**Répondant** : Tous les centres de santé de la zone de santé de Beni ont fonctionnés mais ceux qui n’ont pas fonctionnés c’était par rapport de l’insécurité comme c’est qui est dans la zone rouge comme aire de santé de supakalau il y a de trouble de groupe armée , tu vas trouver un autre centre de santé qui était entre de fonctionné mais il se force toujours comme Boikene ils étaient entre de travaillé malgré l’insécurité ils partaient la journée la nuit il y avait pas de garde comme le centre de santé de Paida ceux gens jusque aujourd’hui ils ne dorment pas en garde ils arrive le matin et le soir tout le monde rentre chez lui à la maison. Donc dans les 18 centres de la zone de Beni, il y avait trois centres de santé qui n’ont pas fonctionnés à 100% à cause de l’insécurité et parmi les trois il y a 1 centre qui n’a pas fonctionné totalement et jusqu’aujourd’hui, c’est le centre de supakalau.

**P21: IA_01_AS_PCOSA_04.rtf - 21:9 [Répondant : Bon les structures..] (24:24)	(Super)**

Codes:	[Structures-Services_fonctionnement]

No memos

**Répondant** : Bon les structures privées ont fonctionnées mais il y avait certaines structures ou sortez de cas puisque les structures privé la majorité sont de commerçant, par ce que quand on parle de la gratuité au centre de santé le privé lui doit faire sortir son argent dans la poche pour l’achat de médicament, contenue de c’est qui la dépensé avec l’idéologie dans la tête que l’Ebola c’est un montage il reste avec le malade et lui aussi tomber malade quand on prélever on trouve le concerné à Ebola on vient lui informer après un autre cas provient de là. On lui dit alors que on ferme ta structure malgré la construction, malgré la compétence des agents mais c’est par ce que vous avez oublié la mode de prévention et nous nous fermons la structure.

**P21: IA_01_AS_PCOSA_04.rtf - 21:10 [Répondant : Bon c’est qui ont ..] (26:26)	(Super)**

Codes:	[Structure-Service_Fermeture_pdt MVE]

No memos

**Répondant** : Bon c’est qui ont étaient fermées dans notre centre de santé de Malepe il y a une structure qui a était fermé et c’est une structure d’une autorité qui est à Kinshasa cette structure à fermer.

**P21: IA_01_AS_PCOSA_04.rtf - 21:11 [Répondant : Oui il n’y avait p..] (28:28)	(Super)**

Codes:	[Triage-Isolement-Chlore-thermoflash_Réaction_population]

No memos

**Répondant** : Oui il n’y avait pas le triage avant.

**P21: IA_01_AS_PCOSA_04.rtf - 21:12 [Répondant : La rumeur circuler..] (30:30)	(Super)**

Codes:	[Triage-Isolement-Chlore-thermoflash_Réaction_population]

No memos

**Répondant** : La rumeur circuler pour le thermo flash, c’est une chose qu’on n’est pas habituer ici chez nous, on était habitué au thermomètre, met ça on mettait en dessous de l’épaule et ça montre la température, mais pour le thermo flash on met ça devant les oreilles ? Et il a encore la forme de revolver, tout ça avec l’insécurité les gens commençaient à se demander s’il n’y a pas de cartouche de bale, mais on disait non c’est juste pour prélever la température et après une autre rumeur que le thermo flash enlève l’intelligence que les gens vont devenir de faux ils n’auront plus à réfléchir. C’est ce qui causer la peur aux gens pour le thermo flash, après quand la population était habituer au thermo flash la population a compris que c’est pour la température seulement mais ce qui crée une différence avec le thermomètre c’est la peinture qui se trouve là-bas.

**P21: IA_01_AS_PCOSA_04.rtf - 21:13 [Répondant : Par rapport à l’is..] (32:32)	(Super)**

Codes:	[Triage-Isolement-Chlore-thermoflash_Réaction_population]

No memos

**Répondant** : Par rapport à l’isolement, l’isolement c’était comment tu arrives on te fait une fiche après on te met dans une chambre, au niveau de l’hôpital on te met dans une salle d’urgence maintenant la population avait peur de l’isolement, que si on est isolé ce que on a Ebola comme on me garde ici les autres vont penser que j’ai Ebola, on lui fait voir que si on te met là c’est par ce que la température est élevé mais on attend que l’équipe arrive pour voir s’il s’agit de quoi. N’ayez pas peur on va pour le test au laboratoire, si le test est négatif de fois donc tu n’as pas Ebola mais si le test est positif ils vont te soigner n’ayez pas peur et la population se dit que l’isolement est venu nous aider à un niveau ou tu ne puisse pas te trouve là où il y a les malades et déjà ça nous protège par ce que si tu viens avec la maladie que tu ne puisse pas contaminer ça aux autres et lorsque tu vas quitter là-bas on te met là où il y a les autres malades et la communauté se dit la vraiment vous nous aidez mais le fait de quitté à la maison directement on te fait une chambre c’est là où la population était trop contaminée.

**P21: IA_01_AS_PCOSA_04.rtf - 21:14 [001737 R : Au début pour dire ..] (36:36)	(Super)**

Codes:	[Triage-Isolement-Chlore-thermoflash_Réaction_population]

No memos

001737 R : Au début pour dire que le gens on accueil, d’abord ils n’étaient pas habitués, les gens disaient que même passer pour aller quelque part il faut se laver les mains tu mets ton pied là où s’est trouvé l’agent sprayeur il commence à pomper te pieds et après on te met un thermoplane déjà c’est ce qui à causer la peur.

**P21: IA_01_AS_PCOSA_04.rtf - 21:15 [Répondant : Les autres malades..] (38:38)	(Super)**

Codes:	[Triage_comportement_patient]

No memos

**Répondant** : Les autres malades quand une personne arrive , il y a d’abord celui qui ne pas habituer à cette eau chloré , quand il trouve de l’eau qui est chloré et avec cette odeur, il dit votre eau dégage une odeur quand on lui met le thermo flash on lui que il a une température de 37 ou bien 38 il faut que tu entre dedans entre d’attendre que l’infirmier arrive, la personne dit si c’est comme ça moi je rentre chez moi à la maison et elle parte pour quoi par ce que elle pense que si la température est élevé donc elle a Ebola, c’est ce qui était un problème.

**P21: IA_01_AS_PCOSA_04.rtf - 21:16 [Répondant : Au début lorsqu’on..] (42:42)	(Super)**

Codes:	[Triage_comportement_patient]

No memos

**Répondant** : Au début lorsqu’on a construit le triage les malades avaient peur dans le sens ou si tu as une température de 38° , 39° et 40°C directement l’ambulance vient te prendre, alors si je pars là-bas avec une température élevé on va appeler une ambulance , hors au CTE celui qui part là-bas il meurt donc moi aussi on veut me mettre dans une ambulance pour aller me tuer et comme IT va appeler qu’ici il y a un malade qu’on vienne le prendre directement IT va recevoir de l’argent par ce que il a appelé , déjà quand la population avait ces idée derrière elle on avait compris ça et on sait dit qu’il faut combattre ça, on est partir informer la population que on est pas payer lorsque on appelle lorsque on appelle c’est pour dire que il y a un malade ici mais vu que on a pas de place au centre de santé que le malade part au CTE là où il y a une place .

**P21: IA_01_AS_PCOSA_04.rtf - 21:17 [Répondant : Lorsque les gens o..] (44:44)	(Super)**

Codes:	[Triage_comportement_patient]

No memos

**Répondant** : Lorsque les gens ont peur d’arrivé au triage hors ces gens sont malade déjà c’est l’équipe qui chercher comme approcher la personne.

**P21: IA_01_AS_PCOSA_04.rtf - 21:18 [Répondant : Si la personne ne ..] (46:46)	(Super)**

Codes:	[Triage_comportement_patient]

No memos

**Répondant** : Si la personne ne veut pas quitter chez elle alors que elle est malade, il y a une équipe qui doit partir voir la personne à la maison cette équipe parle avec la personne en ayant le thermo flash pour la température si elle élevé à combien de degré , après l’avoir tester avec le thermo flash vous lui dit que elle doit partir à l’hôpital comme tu es à la maison et tu es malade tu dois partir à l’hôpital mais on a pas dit que tu as Ebola et tu peux rester dans la maison et tu meurs gratuitement comme ça. Après avoir fait la visite domicile, la personne va quitter sa maison accompagner du relais communautaire ou la personne seule arrive à l’hôpital est dit si j’arrive ici c’est à cause de relais communautaire qui est venu me voir à la maison et ils m’ont orienté ici.

**P21: IA_01_AS_PCOSA_04.rtf - 21:19 [Répondant : Bon pendant cette ..] (48:48)	(Super)**

Codes:	[Qualité_Soins_Patients]

No memos

**Répondant** : Bon pendant cette période de crise par rapport à la prise en charge des malades aux structures , une personne est malade quand elle quitte chez elle pour n’est pas oublier que nos maman aussi sont devenues de médecins , à la maison elle a déjà pris de paracétamol mais ça ne marche pas elle manque quoi faire elle part à l’hôpital quand elle arrive à l’hôpital on lui donne le même paracétamol elle dit à l’infirmier que c’est le paracétamol qu’elle était entre de prendre à la maison déjà ça créer une doute par rapport sa guérison tu vas remarquer que quand elle quitte là-bas elle y passe beaucoup de temps à la réception lorsque elle traine elle-même commence à réclamer que elle partit à l’hôpital mais on lui fait trainer là-bas ça devient quoi un problème . Le problème c’étaient quoi les malades étaient nombreux et pour les soignés tous c’est ne te pas facile c’est pourquoi il y avait retard au centre de santé ils se lamenter surtout à cause de ça que après cinq jour qu’elle vient de recevoir le médicament ou soit il te dit qu’il arrive au centre de santé il y a même pas le médicament et un outre problème le malade arrive au centre l’infirmier n’est pas là , l’infirmier et au centre occuper par un autre travail mais alors que le malade est arrivé et il a dit que le médecin ne pas là. Tout ça c’est par ce que les personnels étaient moins nombreux.

**P21: IA_01_AS_PCOSA_04.rtf - 21:20 [Répondant : Les personnels éta..] (50:50)	(Super)**

Codes:	[Ressources_humaines_Disponibilité et perturbations]

No memos

**Répondant** : Les personnels étaient moins nombreux par ce qu’il est arrivé à un niveau ou les infirmiers avaient difficulté de maitrisé les malades, vous allez trouver par jour vous consulter beaucoup de malades voir 300 ou 600 personnes par jour alors qu’il y a une seule personne à la consultation, il va arriver un moment la personne est fatiguer et lui on est débordé là c’est dans la période de la gratuité.

**P21: IA_01_AS_PCOSA_04.rtf - 21:21 [Répondant : Bon lorsqu’on avai..] (52:52)	(Super)**

Codes:	[Ressources_humaines_Disponibilité et perturbations]

No memos

**Répondant** : Bon lorsqu’on avait appelle les gens à la riposte , ces gens étaient envoyé par les infirmiers titulaire et quand on avait envoyé les infirmiers titulaire et c’est infirmier commencé à parler avec cette personne et puis on prend d’autres par ce que il y a des gens qui sont venu de la communauté sont aussi des infirmiers soit des structures privées, on l’amène ici chez nous et on lui dit de travaillé à la place de celui qui est partie travail a la riposte, c’est là où il y avait perturbation par rapport à la compétence puisque la compétence de celui qui est parti travaillé à la riposte ne pas la même compétence avec celui qui est venu lui remplacer et c’est là où il y avait un problème. Autre qui y a perturbé c’est par rapport à l’argent par exemple celui qui travaille à la riposte touche soit 300 dollars et toi infirmiers qui est au centre de santé on te donne soit 200 dollars, 150 dollars tu te dis que tu devrais aussi partir à la riposte et déjà tu es démoralisé et tu as l’intention de partir aussi à la riposte.

**P21: IA_01_AS_PCOSA_04.rtf - 21:22 [Répondant : Oui le médicament ..] (54:54)	(Super)**

Codes:	[Médicaments_disponibilité_fosa]

No memos

**Répondant** : Oui le médicament était disponible au centre, on nous donné le médicament et comme les malades étaient nombreux le médicament aussi diminue.

**P21: IA_01_AS_PCOSA_04.rtf - 21:23 [Répondant : A la gratuité il y..] (56:56)	(Super)**

Codes:	[Gratuité_Information_population]

No memos

**Répondant** : A la gratuité il y avait à une période juste au début quant au cycle Ebola il est déjà entre quelque jour après , il y avait l’équipe de PDSS , cette équipe avait invité les infirmiers tutélaires et les présidents de comité de santé au BCZS partager pour le médicament et dire que depuis aujourd’hui le médicament devient gratuit, ne vend plus le médicament au malades et si ce système avait existé c’est par ce que plusieurs malades restaient dans leurs maisons, lorsque il est dans la maison et il n’a pas l’argent il reste là mais ils ont vu que avec la stratégie de la gratuité de soins tous ces malades-là vont venir au centre directement PDSS a dit utiliser d’abord le médicament que vous avez on a utilisé nos médicament et après PDSS on nous a apporté le médicament et on a vu que la pharmacie à vraiment le médicament et lorsque un malade arrive il trouve le médicament. Sauf lorsque PDSS a rompu le contrat à certaines structures, le partenaire commence à venir aux structures pour dire qu’ils vont les aider pour mettre la gratuité aux structures, c’est ça le problème même maintenant tu peux passer à Malepe et tu vas trouver qu’il n’y a pas le médicament même au niveau de pharmacie, tu vas seulement trouver le paracétamol.

**P21: IA_01_AS_PCOSA_04.rtf - 21:24 [Répondant : Pour la gratuité b..] (58:58)	(Super)**

Codes:	[Gratuité_Rumeurs]

No memos

**Répondant** : Pour la gratuité beaucoup de gens disaient que la gratuité c’est le 666, comment le médicament devient gratuit, nous sommes né ici on a jamais attendu le médicament gratuit ils se disent non ces gens veulent que l’Ebola continu et la population a dit ces médicament gratuits c’est pour 666 et d’autre disent que ces médicament gratuits c’est un médicament qui est presque dans le date de péremption c’est pourquoi ils profitent pour écouler leur médicament ici chez nous , on le dit non et puis on avait un système donc lorsque on donne a une personne le médicament on ouvre le carton pour lui montre la date d’expiration et voilà la date d’expiration pour le médicament qu’on te donne et la personne dit que le médicament a encore beaucoup de temps avant qu’il n’a soit expiré .

**P21: IA_01_AS_PCOSA_04.rtf - 21:25 [Répondant : Bon depuis il y a ..] (60:60)	(Super)**

Codes:	[Réunion_pdt-épidémie-Ebola]

No memos

**Répondant** : Bon depuis il y a épidémie jusque maintenant on est aussi, on fait une réunion chaque mois.

**P21: IA_01_AS_PCOSA_04.rtf - 21:26 [Répondant : Bon nous le secret..] (62:62)	(Super)**

Codes:	[Réunion_pdt-épidémie-Ebola]

No memos

**Répondant** : Bon nous le secret qu’on avait c’est un secret qui est à partir de BCZS, ce dernier demande un rapport chaque mois pour savoir si nous avons fait une réunion et quelle est les décisions que nous avions prises et combien qui était réalisée et qui n’a pas était réaliser, on se rendu compte que si on ne fait pas de réunions il y aura zéro rapport mieux vaut qu’on envoie un rapport.

**P21: IA_01_AS_PCOSA_04.rtf - 21:27 [Répondant : Dans la communauté..] (64:64)	(Super)**

Codes:	[Changements_depuis_Ebola]

No memos

**Répondant** : Dans la communauté

**P21: IA_01_AS_PCOSA_04.rtf - 21:28 [Répondant : Depuis que Ebola e..] (66:66)	(Super)**

Codes:	[Changements_depuis_Ebola]

No memos

**Répondant** : Depuis que Ebola est à Beni ce qui change c’est à partir de gens qui avaient l’unité de production, par exemple pour une personne qui a un atelier de menuiserie il produit de petite chaise les gens viennent acheter ça pour y placer le seau de lave mains, il construit de chaise déjà l’équipe vient acheter ça. Comme aussi c’est lui qui avait un dépôt de seau il amène aussi de robinet déjà il y a de lavabo, celui-là déjà s’est retrouver. Tu vas trouver une personne qui a un véhicule comme jeep land-cruiser ou pajero il met ça à location à la riposte celui-là aussi s’est retrouver, tu vas voir dans d’autres milieu qui sont presque reculer mais jeunes garçons achètent des parcelles là-bas avec l’argent qu’ils ont gagné dans la riposte. Et puis dans la communauté il y a des gens qui ne savaient même pas l’endroit où se trouve un centre de santé mais à partir de la riposte avec le système de la gratuité la personne dit c’est ici là où se trouve notre centre pour quoi à cause de l’Ebola.

**P21: IA_01_AS_PCOSA_04.rtf - 21:29 [Répondant : Comme il y a épidé..] (68:68)	(Super)**

Codes:	[Souhait_Changement_gestion_futures_épidémies]

No memos

**Répondant** : Comme il y a épidémie on prend soit ça devient une feuille de l’épidémie, vous voyez il y a une équipe de psychologues surtout à la riposte , dans cette équipe tout le monde était déjà préparer et tout le monde qui est dans la riposte si tu demandes il va te dire que la réflexion de tout qui là et après riposte c’est là ou est tout la monde , il fallait qu’il sache que si il est dans la riposte aujourd’hui un jour ça va prendre fin par ce que c’est ne pas une carrière donc ça va passer et la vie continue. C’est là que nous sommes, nous savons qu’après riposte le quelque chose que tu avais gagné là-bas tu peux même ouvrir une unité de production et tu fais d’autres activités.

**P21: IA_01_AS_PCOSA_04.rtf - 21:30 [003433 R : L’épidémie d’abord ..] (70:70)	(Super)**

Codes:	[Souhait_Changement_gestion_futures_épidémies]

No memos

003433 R : L’épidémie d’abord nous avons ça et on est entré de le combattre si aujourd’hui Dieu nous vienne en aide ça va se termine , et derrière ça provient un autre dossier une autre crise déjà l’équipe ou c’est qu’on peut faire au début c’est commencer à la base après avoir commencé à la base il y aura déjà de leader locaux alors quand on va attaquer avec le leader locaux on y va alors entre de monter pas à pas puisque tu ne peux pas combattre une maladie sans sponsor et quand on fait un recours à la base c’est déjà quelque chose qui aide le gens.

**P21: IA_01_AS_PCOSA_04.rtf - 21:31 [Répondant : Bon je ne pas une ..] (72:72)	(Super)**

Codes:	[Commentaire-Recommandation_Répondant]

No memos

**Répondant** : Bon je ne pas une autre chose à ajouter.

**P22: IA_01_AS_PCOSA_05.rtf - 22:1 [Répondant : Moi c’est le prési..] (9:9)	(Super)**

Codes:	[Répondant_Responsabilité_habituelles]

No memos

**Répondant** : Moi c’est le président du comité de santé dans l’aire de santé de BUTILI.

**P22: IA_01_AS_PCOSA_05.rtf - 22:2 [Répondant : Je suis le respons..] (11:11)	(Super)**

Codes:	[Répondant_Responsabilité_habituelles]

No memos

**Répondant** : Je suis le responsable de tous les travaux ici au centre de santé.

**P22: IA_01_AS_PCOSA_05.rtf - 22:3 [Répondant : La construction, e..] (13:13)	(Super)**

Codes:	[Répondant_Responsabilité_habituelles]

No memos

**Répondant** : La construction, et voir comment les travailleurs prestent. Nous nous entraidons avec l’IT pour le suivi des travailleurs dans cette aire de santé.

**P22: IA_01_AS_PCOSA_05.rtf - 22:4 [Répondant : L’aire de santé a ..] (15:15)	(Super)**

Codes:	[Début-Progression_Epidémie MVE]

No memos

**Répondant** : L’aire de santé a connu le cas d’Ebola à partir d’un lieu appelé Ndindi. Ce sont des gens immigrés de Mangina et qui s’étaient cachés là et om les individus ont commencé à mouri. C’est ainsi que la maladie s’est propagée dans cette aire de santé. C’est ainsi qu’on a mis le premiers en cas d’Ebola.

**P22: IA_01_AS_PCOSA_05.rtf - 22:5 [Répondant : Cela veut dire que..] (17:17)	(Super)**

Codes:	[Début-Progression_Epidémie MVE]

No memos

**Répondant** : Cela veut dire que nous avions beaucoup de cas

**P22: IA_01_AS_PCOSA_05.rtf - 22:6 [Répondant : Avant que les infi..] (19:19)	(Super)**

Codes:	[Répondant_Responsabilité_pdt MVE]

No memos

**Répondant** : Avant que les infirmiers soient arrivés, je sensibilisais les gens dans la communauté afin de croire à leurs conseils. On leur montrait que la maladie existait et n’était pas une irréalité afin de pouvoir prendre en considération les mesures édictées par le personnel de riposte et recevoir les vaccins contre cette épidémie.

**P22: IA_01_AS_PCOSA_05.rtf - 22:7 [Répondant : Dans la communauté..] (21:21)	(Super)**

Codes:	[Considération_Ebola_population]

No memos

**Répondant** : Dans la communauté, les gens disaient que l’Ebola était devenu du busines parce que la majorité des personnes affectées dans la riposte étaient des visiteurs ; On n’avait intégré les habitants de Beni dans l’équipe de riposte en premier lieu (ceux-là qui ont travaillé sont venus d’ici et là sans inclure Beni) ce qui avait conduit à ce que la communauté ne comprenne pas si vite.

**P22: IA_01_AS_PCOSA_05.rtf - 22:8 [Répondant : Après avoir dit qu..] (23:23)	(Super)**

Codes:	[Considération_Ebola_population]

No memos

**Répondant** : Après avoir dit que l’Ebola était du busines, d’autres disaient que c’était une force surnaturelle, satanique et que certains ont envoyé cette maladie afin d’en tirer profit ; D’autres disaient que c’était une force démoniaque pour que les complices puissent se taper de l’argent ; C’est ce qui avait provoqué la propagation de cette maladie ici. Les gens se partageaient des fausses informations quant à ce alors que nous qui avions des informations, nous avions déjà découvert que c’était vraiment une maladie mais les gens n’ont pas vite compris même jusqu’à présent ils n’ont pas encore compris. Quelqu’un peut arriver au centre pour la sensibilisation parce qu’il va gagner de l’argent mais sans croire à l’existence de cette épidémie et sans faire le travail lui demandeRépondant :

**P22: IA_01_AS_PCOSA_05.rtf - 22:9 [Répondant : Ici il y a eu un s..] (25:25)	(Super)**

Codes:	[Agression-Menaces_Structure-Prestataire_raisons]

No memos

**Répondant** : Ici il y a eu un seul cas et tous les jeunes sont arrivés au centre de santé et ont détruit les biens mais après l’équipe de riposte et le BCZ ont rassemblé les jeunes pour une sensibilisation et jusqu’à présent ces jeunes ne détruisent plus les biens et matériels du centre de santé.

**P22: IA_01_AS_PCOSA_05.rtf - 22:10 [Répondant : Ils pensaient que ..] (27:27)	(Super)**

Codes:	[Agression-Menaces_Structure-Prestataire_raisons]

No memos

**Répondant** : Ils pensaient que l’Ebola était un jeu pour que certains gagnent de l’argent et d’autres meurent ; c’est ce qui poussait ces jeunes à détruire. Ils disaient que si l’on chassait cette équipe et l’Ebola va disparaitre dans la communauté.

**P22: IA_01_AS_PCOSA_05.rtf - 22:11 [Répondant : Ce qui étaient fer..] (29:29)	(Super)**

Codes:	[Structure-Service_Fermeture_pdt MVE]

No memos

**Répondant** : Ce qui étaient fermés ce sont les postes de santé et les dispensaires qui cachaient les malades avant sans les dénoncer. Lorsque l’on trouvait qu’environ 3 ou 4 personnes sont mortes dans une structure sans dénoncer on vient la fermer même ceux-là qui n’ont suivi le programme de l’équipe de riposte.

**P22: IA_01_AS_PCOSA_05.rtf - 22:12 [Répondant : Ici, nous avons à ..] (31:31)	(Super)**

Codes:	[Structure-Service_Fermeture_pdt MVE]

No memos

**Répondant** : Ici, nous avons à peu près 5 personnes de santé et environs 15 structures.

**P22: IA_01_AS_PCOSA_05.rtf - 22:13 [Répondant : Oui. Il y a eu des..] (33:33)	(Super)**

Codes:	[Service_vaccination_Fonctionnement] [Structures-Services_fonctionnement]

No memos

**Répondant** : Oui. Il y a eu des services qui n’ont pas bien fonctionné. Et d’ailleurs il est arrivé un moment où il n ‘ y avait pas les vaccins pour les enfants parce que l’on avait peur de la contamination. Il fallait que les infirmiers se protègent. Après formation et dotation des matériels de protection aux infirmiers, ces services ont encore repris dans les structures de santé.

**P22: IA_01_AS_PCOSA_05.rtf - 22:14 [Répondant : Non, seulement cel..] (35:35)	(Super)**

Codes:	[Service_vaccination_Fonctionnement]

No memos

**Répondant** : Non, seulement celui-là qui a temporairement connu un suspens

**P22: IA_01_AS_PCOSA_05.rtf - 22:15 [Répondant : Même celui-là n’av..] (39:39)	(Super)**

Codes:	[Service_laboratoire_fonctionnement]

No memos

**Répondant** : Même celui-là n’avait pas bien fonctionné en un moment donné.

**P22: IA_01_AS_PCOSA_05.rtf - 22:16 [Répondant : C’était un program..] (41:41)	(Super)**

Codes:	[Service_laboratoire_fonctionnement]

No memos

**Répondant** : C’était un programme venu de la hiérarchie

**P22: IA_01_AS_PCOSA_05.rtf - 22:17 [Répondant : Tous les labos ; c..] (43:43)	(Super)**

Codes:	[Service_laboratoire_fonctionnement]

No memos

**Répondant** : Tous les labos ; c’était une instruction venue de la hiérarchie pour tous les centres de santé demandant à ces services de fermeRépondant :

**P22: IA_01_AS_PCOSA_05.rtf - 22:18 [Répondant : Les cas de la mate..] (45:45)	(Super)**

Codes:	[Service_maternité_fonctionnement]

No memos

**Répondant** : Les cas de la maternité étaient référés

**P22: IA_01_AS_PCOSA_05.rtf - 22:19 [Répondant : La façon dans laqu..] (47:47)	(Super)**

Codes:	[Service_maternité_fonctionnement]

No memos

**Répondant** : La façon dans laquelle on administrait les soins pouvait faire à ce qu’il y ait contamination. Il fallait alors référe

**P22: IA_01_AS_PCOSA_05.rtf - 22:20 [Répondant : Non] (49:49)	(Super)**

Codes:	[Triage-Isolement-Chlore-thermoflash_Réaction_population]

No memos

**Répondant** : Non

**P22: IA_01_AS_PCOSA_05.rtf - 22:21 [Répondant : Au début le triage..] (51:51)	(Super)**

Codes:	[Triage_comportement_patient]

No memos

**Répondant** : Au début le triage était attaqué. Mais actuellement on ne le fait plus parce que lorsque quelqu’un entre on prélève sa température, il se lave les mains et puis il est autorisé à entrer dans la structure de santé.

**P22: IA_01_AS_PCOSA_05.rtf - 22:22 [Répondant : Avant, on n’en éta..] (53:53)	(Super)**

Codes:	[Triage_comportement_patient]

No memos

**Répondant** : Avant, on n’en était contre

**P22: IA_01_AS_PCOSA_05.rtf - 22:23 [Répondant : Avant quand on dem..] (55:55)	(Super)**

Codes:	[Triage_Considération_population]

No memos

**Répondant** : Avant quand on demandait à quelqu’un de se laver les mains ou de prélever sa température, il refusait. Les gens n’avaient pas encore reçu des enseignements par rapport à la maladie, on en croyait pas vraiment. Il fallait au départ qu’ils soient sensibilisés pour connaitre cette maladie.

**P22: IA_01_AS_PCOSA_05.rtf - 22:24 [Répondant : Ce thermo flash a ..] (57:57)	(Super)**

Codes:	[Triage-Isolement-Chlore-thermoflash_Réaction_population]

No memos

**Répondant** : Ce thermo flash a un mauvais air et lorsqu’il est mis sur son corps, cela va occasionner des maladies parce que la communauté vivait dans l’ignorance.

**P22: IA_01_AS_PCOSA_05.rtf - 22:25 [Répondant : Au début ceux-là q..] (59:59)	(Super)**

Codes:	[Triage-Isolement-Chlore-thermoflash_Réaction_population]

No memos

**Répondant** : Au début ceux-là qui se lavaient étaient moins nombreux. Lorsqu’on disait à quelqu’un de se laver ses mains, il avait peur

**P22: IA_01_AS_PCOSA_05.rtf - 22:26 [Répondant : le fait que le chl..] (61:61)	(Super)**

Codes:	[Triage-Isolement-Chlore-thermoflash_Réaction_population]

No memos

**Répondant** : le fait que le chlore contenait du chlore, on disait que cela avait une mauvaise odeur et ce temps là on n’avait pas encore compris que c’était un désinfectant.

**P22: IA_01_AS_PCOSA_05.rtf - 22:27 [Répondant : Certains y fréquen..] (63:63)	(Super)**

Codes:	[Structure-Service_Utilisation_population]

No memos

**Répondant** : Certains y fréquentaient mais d’autres non parce que lorsque la température du malade était élevée, il était vite transféré ce qui avait créé une panique au niveau de la communauté ; lorsqu’on avait la température de 38 ou 39°C, on était référé au CTE et dans la communauté on disait que pour celui-là c’était la mort. Ceux-là qui savaient l’existence de cette maladie arrivaient et on les soignait mais d’autres disaient que la diarrhée et le vomissement sont entrain d‘occasionner les morts d’homme alors qu’avant ce n’était pas le cas et se disaient qu’ils ne seront plus à l’hôpital.

**P22: IA_01_AS_PCOSA_05.rtf - 22:28 [Répondant : Dans des dispensai..] (65:65)	(Super)**

Codes:	[Structure-Service_Utilisation_population]

No memos

**Répondant** : Dans des dispensaires privés.

**P22: IA_01_AS_PCOSA_05.rtf - 22:29 [Répondant : Il n’y avait pas d..] (67:67)	(Super)**

Codes:	[Triage-Isolement-Chlore-thermoflash_Réaction_population]

No memos

**Répondant** : Il n’y avait pas de triage et on avait là des lits qui pouvaient servir deux malades

**P22: IA_01_AS_PCOSA_05.rtf - 22:30 [Répondant : Oui parce qu’à tou..] (69:69)	(Super)**

Codes:	[Triage_comportement_patient]

No memos

**Répondant** : Oui parce qu’à tout moment on référait les malades.

**P22: IA_01_AS_PCOSA_05.rtf - 22:31 [Répondant : Oui, et puis il es..] (71:71)	(Super)**

Codes:	[Triage_comportement_patient]

No memos

**Répondant** : Oui, et puis il est arrivé un moment où un malade a été dans un état critique dans sa maison alors que les soins étaient gratuits pour éviter d’être référé. Et lorsque l’équipe de riposte constatait, on venait le récupérer et pouvait mourir même dans l’ambulance sans arriver au lieu des soins. Ou bien lorsqu’arrivé à l’hôpital, il pouvait mourir parce que les soignants devaient se chercher de matériels pour se protégeRépondant : Et cela ce message arrivait dans la communauté, on se disait pourquoi référer un malade et mouriRépondant : C’était parmi les cas qui était à la base des soulèvements des jeunes et qui pouvait faire à ce qu’ils incendient le centre de santé.

**P22: IA_01_AS_PCOSA_05.rtf - 22:32 [Répondant : Ils acceptaient le..] (73:73)	(Super)**

Codes:	[Qualité_Soins_Patients]

No memos

**Répondant** : Ils acceptaient les soins mais il arrivait de fois où un malade pouvait attendre même 30 minutes sans recevoir les soins ce qui décourageait les malades à fréquenter le centre de santé.

**P22: IA_01_AS_PCOSA_05.rtf - 22:33 [Répondant : Oui, et quand l’in..] (75:75)	(Super)**

Codes:	[Qualité_Soins_Patients]

No memos

**Répondant** : Oui, et quand l’infirmier le consultait en les touchant sur le corps mais actuellement, ce sont ceux qui défendaient un rapprochement avec les malades suite à la présence de l’Ebola. On ne parvenait plus à faire des injections aux malades, on leur donnait seulement des comprimés mais c’est à l’hôpital seulement où on injectait et où on faisait des examens de labo.

**P22: IA_01_AS_PCOSA_05.rtf - 22:34 [Répondant : Oui, car certains ..] (77:77)	(Super)**

Codes:	[Traitement_Invasifs_Prestataire_changement]

No memos

**Répondant** : Oui, car certains malades envisageaient être interné parce qu’ils auront des sérums. Mais comme on ne les donnait plus aux malades, ces derniers préféraient rester à la maison.

**P22: IA_01_AS_PCOSA_05.rtf - 22:35 [Répondant : Il y a deux mois q..] (79:79)	(Super)**

Codes:	[Traitement_Invasifs_Prestataire_changement]

No memos

**Répondant** : Il y a deux mois que les gens ont compris ; les malades commencent à arriver dans le centre de santé même à 14 heures, à 15 heures ils arrivent parce qu’ils ont compris l’existence de la maladie et de son médicament.

**P22: IA_01_AS_PCOSA_05.rtf - 22:36 [Répondant : Vivre avec les gen..] (83:83)	(Super)**

Codes:	[Gratuité_Accueil_population]

No memos

**Répondant** : Vivre avec les gens n’est pas chose facile. Il disait que ce qui est donné gratuitement doit être de mauvaise qualité et n’a pas d’ordre. Les malades préféraient être soignés là où ils vont payer au lieu bénéficier des soins gratuits qui ne vont pas l’aider à recouvrer sa santé parce qu’ils aiment être soignés de la façon voulue. Certains venaient demander des injections aux infirmiers ce qu’ils ne pouvaient pas bénéficier au profit des comprimés.

**P22: IA_01_AS_PCOSA_05.rtf - 22:37 [Répondant : Dans cette structu..] (85:85)	(Super)**

Codes:	[Ressources_humaines_Disponibilité et perturbations]

No memos

**Répondant** : Dans cette structure, tous les infirmiers étaient toujours disponibles pour les services.

**P22: IA_01_AS_PCOSA_05.rtf - 22:38 [Répondant : Non, tous étaient ..] (87:87)	(Super)**

Codes:	[Ressources_humaines_Disponibilité et perturbations]

No memos

**Répondant** : Non, tous étaient présents ici, seulement l’Infirmier Titulaire était parti dans un mois après lequel il est encore revenu.

**P22: IA_01_AS_PCOSA_05.rtf - 22:39 [Répondant : Non, seulement le ..] (89:89)	(Super)**

Codes:	[Ressources_humaines_Disponibilité et perturbations]

No memos

**Répondant** : Non, seulement le problème de communication entre les infirmiers et les malades qui était fréquent avant, mais ils étaient toujours disponibles.

**P22: IA_01_AS_PCOSA_05.rtf - 22:40 [Répondant : Les malades étaien..] (91:91)	(Super)**

Codes:	[Prestataire_Pratique de Prévention contre MVE]

No memos

**Répondant** : Les malades étaient mécontents de ce comportement nouveau parce que cela ne se faisait pas auparavant alors que les infirmiers étaient déjà informés que lorsqu’on pouvait touchait un malade d’Ebola sans se masquer, on pouvait être contaminé. C’est pourquoi ces infirmiers eux commençaient à se protéger avant de soigneRépondant : Mais le malade lui n’avait pas encore compris ce phénomène.

**P22: IA_01_AS_PCOSA_05.rtf - 22:41 [Répondant : Oui. Ils pensaient..] (93:93)	(Super)**

Codes:	[Prestataire_Pratique de Prévention contre MVE]

No memos

**Répondant** : Oui. Ils pensaient que lorsque l’infirmier partait par-ci par-là pour ses matériels de masque, c’était une perte de temps inutile ce qui impatientait les malades. Et par après on pouvait référer le cas et le malade partait en pleurant. En le conscientisant de ne pas pleurer, le malade disait qu’on voulait partir le tuer là où il est référé (CTE).

**P22: IA_01_AS_PCOSA_05.rtf - 22:42 [Répondant : Pour eux le CTE, c..] (95:95)	(Super)**

Codes:	[Considération_CTE_population]

No memos

**Répondant** : Pour eux le CTE, c’était question de tuer les personnes qui étaient référées. Cela veut dire que le malade disait qu’on voulait plus de lui dans la communauté mais plutôt, on voulait le tueRépondant :

**P22: IA_01_AS_PCOSA_05.rtf - 22:43 [Répondant : Oui, il y en a eu...] (97:97)	(Super)**

Codes:	[Gratuité_Structures-Services_concernés]

No memos

**Répondant** : Oui, il y en a eu. Pendant toute la période d’Ebola les soins étaient gratuits. On ne payait pas.

**P22: IA_01_AS_PCOSA_05.rtf - 22:44 [Répondant : Oui tous les soins..] (99:99)	(Super)**

Codes:	[Gratuité_Structures-Services_concernés]

No memos

**Répondant** : Oui tous les soins

**P22: IA_01_AS_PCOSA_05.rtf - 22:45 [Répondant : Oui, même jusqu’à ..] (101:101)	(Super)**

Codes:	[Gratuité_Structures-Services_concernés]

No memos

**Répondant** : Oui, même jusqu’à présent.

**P22: IA_01_AS_PCOSA_05.rtf - 22:46 [Répondant : C’est ALIMA qui fi..] (103:103)	(Super)**

Codes:	[Gratuité_Partenaire d'appui]

No memos

**Répondant** : C’est ALIMA qui financent cette gratuité actuellemen

**P22: IA_01_AS_PCOSA_05.rtf - 22:47 [Répondant : C’était PDSS] (105:105)	(Super)**

Codes:	[Gratuité_Partenaire d'appui]

No memos

**Répondant** : C’était PDSS

**P22: IA_01_AS_PCOSA_05.rtf - 22:48 [Répondant : Le PDSS a commencé..] (107:107)	(Super)**

Codes:	[Gratuité_Partenaire d'appui]

No memos

**Répondant** : Le PDSS a commencé n Aout 2028 le moment où l’Ebola a commencé dans la région jusqu’en Décembre 2019 et nous sommes avec ALIMA il y a seulement 2 mois.

**P22: IA_01_AS_PCOSA_05.rtf - 22:49 [Répondant : Non, c’était seule..] (109:109)	(Super)**

Codes:	[Gratuité_Interruption]

No memos

**Répondant** : Non, c’était seulement environ un mois il n’y avait pas gratuité des soins pour avoir le moyen du fonctionnement du centre.

**P22: IA_01_AS_PCOSA_05.rtf - 22:50 [Répondant : C’était au mois de..] (111:111)	(Super)**

Codes:	[Gratuité_Interruption]

No memos

**Répondant** : C’était au mois de décembre, janvier je pense

**P22: IA_01_AS_PCOSA_05.rtf - 22:51 [Répondant : Environ 3.5 dollar..] (113:113)	(Super)**

Codes:	[Gratuité_Interruption]

No memos

**Répondant** : Environ 3.5 dollars seulement que le malade payait pour ses soins.

**P22: IA_01_AS_PCOSA_05.rtf - 22:52 [Répondant : Ils en étaient con..] (115:115)	(Super)**

Codes:	[Gratuité_Accueil_population]

No memos

**Répondant** : Ils en étaient contents.

**P22: IA_01_AS_PCOSA_05.rtf - 22:53 [Répondant : Pour la nouvelle g..] (117:117)	(Super)**

Codes:	[Gratuité_Rumeurs]

No memos

**Répondant** : Pour la nouvelle gratuité, il n’y a plus des rumeurs.

**P22: IA_01_AS_PCOSA_05.rtf - 22:54 [Répondant : Il y avait beaucou..] (119:119)	(Super)**

Codes:	[Gratuité_Rumeurs]

No memos

**Répondant** : Il y avait beaucoup de rumeurs.

**P22: IA_01_AS_PCOSA_05.rtf - 22:55 [Répondant : On pensait que les..] (121:121)	(Super)**

Codes:	[Gratuité_Rumeurs]

No memos

**Répondant** : On pensait que les médicaments apportés pour cette fin étaient de mauvaise qualité. Et le fait que les prestataires ne recevaient plus las malades comme avant faisait à ce que les malades refusent de venir au centre pour les soins alors que c’étaient des bons médicaments donnés gratuitement.

**P22: IA_01_AS_PCOSA_05.rtf - 22:56 [Répondant : Oui, nombreux ont ..] (123:123)	(Super)**

Codes:	[Gratuité_Accueil_population]

No memos

**Répondant** : Oui, nombreux ont accepté.

**P22: IA_01_AS_PCOSA_05.rtf - 22:57 [Répondant : Nous avons de rela..] (125:125)	(Super)**

Codes:	[Gratuité_Information_population]

No memos

**Répondant** : Nous avons de relais communautaire qui ont cette activité.

**P22: IA_01_AS_PCOSA_05.rtf - 22:58 [Répondant : Oui] (127:127)	(Super)**

Codes:	[Médicaments_disponibilité_fosa]

No memos

**Répondant** : Oui

**P22: IA_01_AS_PCOSA_05.rtf - 22:59 [Répondant : Nous faisions des ..] (131:131)	(Super)**

Codes:	[Réunion_pdt-épidémie-Ebola]

No memos

**Répondant** : Nous faisions des réunions avec une équipe restreinte avec l’IT et ses agents. Mais pour le comité de santé, non.

**P22: IA_01_AS_PCOSA_05.rtf - 22:60 [Répondant : On n’avait pas int..] (133:133)	(Super)**

Codes:	[Réunion_pdt-épidémie-Ebola]

No memos

**Répondant** : On n’avait pas interdit. Mais on avait beaucoup de travaux : participer des réunions, faire des sensibilisations mais certains travaux s’effectuaient.

**P22: IA_01_AS_PCOSA_05.rtf - 22:61 [Répondant : Au centre de santé..] (135:135)	(Super)**

Codes:	[Changements_depuis_Ebola]

No memos

**Répondant** : Au centre de santé il y a eu des nouveautés : existence des douches, les latrines, la salle que tu vois là, le triage et ce bâtiment a été construit par l’aide de PDSS. Mais vraiment avant, nous étions dans des mauvaises conditions qui n’étaient adaptés pour faire des soins de santé.

**P22: IA_01_AS_PCOSA_05.rtf - 22:62 [Répondant : Nous, on veut que ..] (137:137)	(Super)**

Codes:	[PCI_Comment_maintenir]

No memos

**Répondant** : Nous, on veut que cette pratique soit quotidienne et que les formations des gens soient également continuelles. Que l’on ne dise pas que la maladie a été éradiquée et laisser ces pratiques d’hygiènes. Que les gens reçoivent même des lavabos pour aider la communauté. Le lavage des mains on ne peut pas le laisser au niveau du centre de santé.

**P22: IA_01_AS_PCOSA_05.rtf - 22:63 [Répondant : Si cela revenait, ..] (139:139)	(Super)**

Codes:	[Souhait_Changement_gestion_futures_épidémies]

No memos

**Répondant** : Si cela revenait, que l’on prépare des centres de soins pour la maladie en question dans chaque aire de santé et ne pas référer encore les malades ailleurs.

**P22: IA_01_AS_PCOSA_05.rtf - 22:64 [Répondant : Si l’Ebola revenai..] (141:141)	(Super)**

Codes:	[Souhait_Changement_gestion_futures_épidémies]

No memos

**Répondant** : Si l’Ebola revenait.

**P22: IA_01_AS_PCOSA_05.rtf - 22:65 [Répondant : D’abord ceux-là qu..] (143:143)	(Super)**

Codes:	[Souhait_Changement_gestion_futures_épidémies]

No memos

**Répondant** : D’abord ceux-là qui ne sont pas confirmés peuvent faire le labo ici et envoyer l’Echantillon ailleurs ; en cas de confirmation, on le réfère maintenant.

**P22: IA_01_AS_PCOSA_05.rtf - 22:66 [Répondant : Oui, parce que lor..] (145:145)	(Super)**

Codes:	[Souhait_Changement_gestion_futures_épidémies]

No memos

**Répondant** : Oui, parce que lorsqu’on le teste là où il a été référé et rentrer dans deux jours créer des polémiques. Mais li doit falloir transférer seulement les cas confirmé au centre de santé.

**P22: IA_01_AS_PCOSA_05.rtf - 22:67 [Répondant : Il faudra engager ..] (147:147)	(Super)**

Codes:	[Souhait_Changement_gestion_futures_épidémies]

No memos

**Répondant** : Il faudra engager l’équipe de riposte dans la communauté ; que les membres de l’équipe soient identifiables chez eux parce que lorsque l’on envoi quelqu’un qui n’est pas identifiable, on suppose que c’est la politique de tuer les gens. Le fait qu’il ya eu massacre ici et puis l’Ebola, les gens supposent que c’est une façon d’exterminer la communauté. Si cela revenait, cherchez comment informer la hiérarchie pour que les gens ne paniquent plus afin d’obtenir des soins dans des stratégies.

**P22: IA_01_AS_PCOSA_05.rtf - 22:68 [Répondant : C’est un remerciem..] (149:149)	(Super)**

Codes:	[Commentaire-Recommandation_Répondant]

No memos

**Répondant** : C’est un remerciement parce que l’équipe qui a soutenu la gratuité avant a aidé à ce que le centre de santé ait une figure nouvelle ; qu’ils continuent à nous soutenir parce que nous n’avons rien comme moyen alors que notre parcelle est encore petite. Que la hiérarchie puisse nous aider pour le centre ait au moins deux parcelles et continuer avec la construction pour ne pas manquer où interner les malades.

**P23: IA_01_HGR_AG.rtf - 23:1 [Répondant : Dans la gestion de..] (2:2)	(Super)**

Codes:	[Répondant_Responsabilité_pdt MVE]

No memos

Répondant : Dans la gestion des MVE, l’HGR de Beni a joué le rôle de triage d’abord à ce niveau donc à l’entrée de l’hôpital, il y a eu un triage et dans son enceinte, le centre de traitement de cette maladie a été érigé depuis donc Aout 2018,

**P23: IA_01_HGR_AG.rtf - 23:2 [Répondant : dans cette riposte..] (4:4)	(Super)**

Codes:	[Répondant_Responsabilité_pdt MVE]

No memos

Répondant : dans cette riposte comme administrateur général de l’hôpital, je jouais le rôle de logisticien dans l’hôpital général de Beni par rapport à cette maladie et puis je fais aussi la gestion des ressources humaines mais je fais aussi partir de la commission prise en charge médicale donc le bureau commission prise en charge médicale, je jouais le rôle de data

**P23: IA_01_HGR_AG.rtf - 23:3 [Répondant : au début de l’épid..] (6:6)	(Super)**

Codes:	[Considération_Ebola_population]

No memos

Répondant : au début de l’épidémie, la population a considéré Ebola comme un démon qui st arrivé, juste au début on disait que c’est un poison qu’on donnait à la population et après ils ont dit que c’est un démon qui est venu et les autres ont dit que ce sont les blancs qui ont crée cette maladie pour venir travailler dans cette partie du pays et ils ont aussi dit que cette maladie a été créée pour éliminer la population de l’Est parce que cette situation a échoué avec les massacres. Voilà un peu ce qui était la vision de la population par rapport à cette maladie.

**P23: IA_01_HGR_AG.rtf - 23:4 [Répondant : les attaques que l..] (8:8)	(Super)**

Codes:	[Agression-Menaces_Structure-Prestataire_raisons]

No memos

Répondant : les attaques que les structures ont subies, c’est toujours liés à ce que je venais de dire là donc une maladie qui est importée par l’extérieur pour exterminer la population de l’Est du pays et pour chercher le travail, les autres appellent ça Ebola business

**P23: IA_01_HGR_AG.rtf - 23:5 [Répondant : pendant la période..] (10:10)	(Super)**

Codes:	[Structures-Services_fonctionnement]

No memos

Répondant : pendant la période d’Ebola, il y a eu beaucoup de problèmes parce que juste au début nous avions consacré tout un service pour prendre le suspect Ebola parce que nous on savait pas comment Ebola fonctionne donc tous ces services que nous avons pris pour prendre les suspects a été directement mise en quarantaine et après ce service a été réfectionnée pour être réutiliser comme labo INRB et service des suivis des guéries de la MVE. Ça perturbait la capacité d’accueil de l’hôpital donc elle était de 202 lits et elle a chuté jusqu’à 150 lits mais aussi avec l’installation de la PCI parce qu’on avait beaucoup des lits qui étaient entassés dans les salles, on avait beaucoup des malades à cause de la gratuité des soins et quand nous avons arrangé les lits selon la PCI, nous avons encore perdu 35 lits parce que jusque-là nous avons travaillé avec 115 lits à l’hôpital

**P23: IA_01_HGR_AG.rtf - 23:6 [Répondant : oui, ça perturbait..] (12:12)	(Super)**

Codes:	[Qualité_Soins_Patients]

No memos

Répondant : oui, ça perturbait l’accueil des malades parce qu’on était dans la gratuité des soins et tout le monde qui se cachait avec sa maladie à la maison qui voulait venir aux soins et on n’avait pas une place pour garder tous ces malades et à un moment on a même refusait d’accueillir certains malades parce qu’on voulait respecter la PCI

**P23: IA_01_HGR_AG.rtf - 23:7 [Répondant : pendant la période..] (14:14)	(Super)**

Codes:	[Service_laboratoire_fonctionnement]

No memos

Répondant : pendant la période d’Ebola, tous les services techniques ont fonctionné normalement, ce sont les services d’hospitalisation qui ont été perturbé

**P23: IA_01_HGR_AG.rtf - 23:8 [Répondant : on a interdit d’ut..] (16:16)	(Super)**

Codes:	[Service_laboratoire_fonctionnement]

No memos

Répondant : on a interdit d’utiliser le TDR pour la malaria et c’est seulement ça qu’on a interdit dans tous les services de l’hôpital

**P23: IA_01_HGR_AG.rtf - 23:9 [Répondant : bon ici chez nous ..] (18:18)	(Super)**

Codes:	[Triage_comportement_patient]

No memos

Répondant : bon ici chez nous à l’installation de la gratuité, les malades n’ont pas résisté par rapport au triage mais comme d’habitude, il y a toujours ceux qui s’évadent des services de l’hôpital et c’est arrivé aussi au niveau du triage mais ça n’a pas vraiment causé des problèmes parce qu’il  y avait le service de psychosocial qui parlait aussi avec des malades et il y avait aussi la communication qui était déjà installée au niveau de l’hôpital pour rassurer les malades de ce triage.

**P23: IA_01_HGR_AG.rtf - 23:10 [Répondant : il y avait des rés..] (20:20)	(Super)**

Codes:	[Structure-Service_Utilisation_population]

No memos

Répondant : il y avait des résistances parce que les malades considéraient l’hôpital général de référence de Beni, c’est l’hôpital qui héberge Ebola alors si quelqu’un vient à l’hôpital ; il va directement infecter de cette maladie mais au fur et à mesure qu’ils ont compris qu’il y avait un bon suivi des malades, il y a la PCI qui est relevé, ils sont venus pour les soins

**P23: IA_01_HGR_AG.rtf - 23:11 [Répondant : concernant l’accue..] (22:22)	(Super)**

Codes:	[Qualité_Soins_Patients]

No memos

Répondant : concernant l’accueil dans la structure tel que je l’ai dit, qu’il y a eu diminution des places pour l’hospitalisation

**P23: IA_01_HGR_AG.rtf - 23:12 [Répondant : eh pour les malade..] (24:24)	(Super)**

Codes:	[Qualité_Soins_Patients]

No memos

Répondant : eh pour les malades ambulatoires, bon je veux y arriver, il y avait une diminution de l’accueil pour l’hospitalisation et les malades de l’accueil étaient un peu en difficulté, il ne voulait plus venir à l’hôpital mais parce qu’il y a eu la gratuité des soins qui est venue, on a vu un afflux des malades qui est venu pour cette gratuité des soins, la population de cette contrée est vraiment déminue, paupériser par les massacres et différentes guerres

**P23: IA_01_HGR_AG.rtf - 23:13 [Répondant : bien sûr la façon ..] (26:26)	(Super)**

Codes:	[Prestataire_Pratique de Prévention contre MVE]

No memos

Répondant : bien sûr la façon de prendre en charge les malades à changer pour les prestataires parce qu’ils ont senti que c’est une maladie qui est contagieuse et en touchant les malades, on ne sait pas qui peut avoir cette ma maladie juste à l’arrivée mais quand on pose la question, on peut avoir un malade qui n’a pas encore des symptômes dans le service d’hospitalisation, mais au fur et à mesure qu’on on est en train de le soigner, il peut arriver qu’il commence à présenter les symptômes donc les prestataires se sont mis à se protéger parce qu’au moins deux fois cet hôpital de Beni, on a eu à détecter des cas confirmés de MVE.

**P23: IA_01_HGR_AG.rtf - 23:14 [Répondant : au niveau de notre..] (28:28)	(Super)**

Codes:	[Ressources_humaines_Disponibilité et perturbations]

No memos

Répondant : au niveau de notre structure par rapport aux ressources humaines, juste quand la maladie a surgi dans cette contrée tellement qu’on était moins informé de la maladie et c’est tous les prestataires qui ont eu peur d’affronter ces prestations mais quand ils ont mis que d’autres prestataires se sont mis à soigner les malades, ils ont vu qu’ils n’ont eu aucun problème par rapport à l’infection. Ils se sont aussi orientés pour aller soigné les malades MVE aussi parce que les prestataires qui soignent les malades MVE étaient mieux payés que ceux qui étaient dans les hôpitaux donc si vous pouvez voir un prestataire du CTE ou de CT donc il avait un 15$ pour un infirmier par jour mais pour un infirmier qui est à l’hôpital, il pouvait avoir moins de 5$ par jour alors cela a fonctionnement de l’hôpital parce que c’est tout le monde qui voulait être un prestataire dans les activités de la riposte contre la MVE

**P23: IA_01_HGR_AG.rtf - 23:15 [Répondant : tous les services ..] (30:30)	(Super)**

Codes:	[Ressources_humaines_Disponibilité et perturbations]

No memos

Répondant : tous les services ont soufferts, en tout cas il n’y a pas un service qui n’a pas souffert parce que même le service de l’administration, c’est tout le monde qui voulait aller dans la riposte contre la MVE

**P23: IA_01_HGR_AG.rtf - 23:16 [Répondant : la situation de l’..] (32:32)	(Super)**

Codes:	[Ressources_humaines_solution-local_Disponibilité et perturbations]

No memos

Répondant : la situation de l’hôpital et comme on était en difficulté et plusieurs ne sont pas payés par l’Etat, qui n’ont pas le salaire, ils n’ont pas la prime de risque et nous avons vu qu’il ne fallait pas mettre la pression sur ceux qui voulaient dans la riposte et comme il y avait beaucoup des chômeurs qui avaient fini leurs études, on a pris par contrat quelques personnes qui couvraient les places qui étaient laissées par les autres prestataires mais aussi c’était une épidémie qui était dans notre contrée et on ne pouvait pas laisser les autres prestataires d’autres contrées venir apprendre ce que nous devons avoir comme acquis dans notre situation de prise en charge

**P23: IA_01_HGR_AG.rtf - 23:17 [Répondant : tel que nous avons..] (34:34)	(Super)**

Codes:	[Ressources_humaines_solution-local_Disponibilité et perturbations]

No memos

Répondant : tel que nous avons constaté la performance a été maintenu pourquoi ? Parce que ceux-là qui étaient pris comme agents de relèves, ils étaient déjà passé comme des stagiaires dans l’hôpital avant même que la maladie n’apparaisse donc il n’a pas eu des problèmes par rapport à ça

**P23: IA_01_HGR_AG.rtf - 23:18 [Répondant : l’épidémie est ven..] (36:36)	(Super)**

Codes:	[Ressources_humaines_situation_grève]

No memos

Répondant : l’épidémie est venue dans un contexte de grève et nous-mêmes ont été en grève mais c’était une drève qui n’était pas vraiment très suivie dans cet hôpital parce que plusieurs personnes dans cet hôpital ne recevaient même pas cette prime non pas de salaire et comme on vit d’une manière quotidienne dès ce que nous gagnons dans l’hôpital et même si on faisait la grève, on n’avait pas où on pouvait aller travailler pour trouver un sous enfin de nouer les deux bouts du mois. Comme c’était à l’hôpital, les malades paient quand même quelque chose, on n’a pas vraiment senti cette grève à notre niveau.

**P23: IA_01_HGR_AG.rtf - 23:19 [Répondant : avant la crise d’E..] (38:38)	(Super)**

Codes:	[Recouvrement_coûts-des-soins_avant gratuité]

No memos

Répondant : avant la crise d’Ebola, il y avait une tarification par acte

**P23: IA_01_HGR_AG.rtf - 23:20 [Répondant : et avec l’arrivée ..] (40:40)	(Super)**

Codes:	[Recouvrement_coûts-des-soins_avant gratuité]

No memos

Répondant : et avec l’arrivée de la riposte, nous avions été mis dans le système de subventionnement des soins et jusque-là on n’a pas encore changé le système donc depuis le début de la riposte nous sommes toujours dans ce subventionnement des soins par les partenaires

**P23: IA_01_HGR_AG.rtf - 23:21 [Répondant : c’est la même chos..] (42:42)	(Super)**

Codes:	[Gratuité_Evolution]

No memos

Répondant : c’est la même chose ici, nous avons fait trois mois de subvention totale et après nous avons passé dans les subventions partielles pour les restes de temps

**P23: IA_01_HGR_AG.rtf - 23:22 [Répondant : cette subvention e..] (44:44)	(Super)**

Codes:	[Gratuité_Partenaire d'appui]

No memos

Répondant : cette subvention est appuyée par PDSS à travers EIP face qui est le vérificateur

**P23: IA_01_HGR_AG.rtf - 23:23 [Répondant : dans la communauté..] (46:46)	(Super)**

Codes:	[Gratuité_Rumeurs]

No memos

Répondant : dans la communauté, il n’y a pas eu des rumeurs et à notre niveau vraiment la population a été soulagé par rapport à cette gratuité alors on n’a pas eu beaucoup des rumeurs

**P23: IA_01_HGR_AG.rtf - 23:24 [Répondant : on est passé du su..] (48:48)	(Super)**

Codes:	[Gratuité_Evolution]

No memos

Répondant : on est passé du subventionnement total au subventionnement partielle parce qu’on a constaté que tous les services ne pouvaient pas remontés des alertes de MVE alors on a choisi des services qui auraient des symptômes avec les MVE

**P23: IA_01_HGR_AG.rtf - 23:25 [Répondant : il n’y a pas eu te..] (50:50)	(Super)**

Codes:	[Gratuité_Conséquences_motivation_prestataire]

No memos

Répondant : il n’y a pas eu tellement des changements mais nous avons constaté qu’une petite amélioration de la motivation du personnel par rapport à la période d’avant épidémie. Une petite amélioration

**P23: IA_01_HGR_AG.rtf - 23:26 [épondant : quand nous avons ca..] (52:52)	(Super)**

Codes:	[Gratuité_Conséquences_motivation_prestataire]

No memos

épondant : quand nous avons calculé, nous pensons que c’était de 5% par rapport à ce qui était avant l’épidémie

**P23: IA_01_HGR_AG.rtf - 23:27 [Répondant : jusqu’à présent no..] (54:54)	(Super)**

Codes:	[Gratuité_Partenaire d'appui]

No memos

Répondant : jusqu’à présent non, nous avons été avec PDSS jusqu’au 15 novembre 2019 et après nous sommes maintenant avec le partenaire Alima, une ONG international qui appuie la gratuité totale dans la structure

**P23: IA_01_HGR_AG.rtf - 23:28 [Répondant : ils n’ont pas la m..] (56:56)	(Super)**

Codes:	[Gratuité_Structures-Services_concernés]

No memos

Répondant : ils n’ont pas la même politique, PDSS lui faisait l’achat des prestations et Alima lui payait le forfait pour les prestataires et un forfait pour le fonctionnement de l’hôpital mais aussi il fait la réhabilitation des quelques bâtiments qui sont délabrés et aussi donnés le repas à tous les malades à l’hôpital. PDSS, lui faisait l’achat des prestations avec une tarification forfaitaire

**P23: IA_01_HGR_AG.rtf - 23:29 [Répondant : si on nous demande..] (58:58)	(Super)**

Codes:	[Gratuité_Structures-Services_concernés]

No memos

Répondant : si on nous demande de choisir les deux appuis, nous on choisirait l’appui PDSS puisque là nous avons essayé de réaliser quelques projets qui étaient en souffrance dans l’hôpital

**P23: IA_01_HGR_AG.rtf - 23:30 [Répondant : pendant la période..] (60:60)	(Super)**

Codes:	[Médicaments_disponibilité_fosa]

No memos

Répondant : pendant la période de crise comparée à la situation avant, la disponibilité des médicaments a été de la même façon parce qu’avant que l’épidémie n’arrive, on était toujours approvisionné par le BCZ et nous on achetait les médicaments et pendant l’épidémie, nous étions subventionnés par PDSS et on achetait aussi les médicaments au niveau du bureau central de la zone de santé mais avec le partenaire Alima lui il nous fournit en médicaments, c’est là où nous avons un peu des problèmes parce qu’il ne parvient pas à couvrir tous les besoins de l’hôpital en médicaments

**P23: IA_01_HGR_AG.rtf - 23:31 [Répondant : le défi que nous a..] (62:62)	(Super)**

Codes:	[Gratuité_Défis]

No memos

Répondant : le défi que nous avons affronté, c’était d’abord la disponibilité des médicaments au niveau du BCZ qui n’était pas vraiment à même de suffire à tous les malades qui venaient à l’hôpital pour avoir les soins gratuits et on était obligé de chercher d’autres intrants dans les pharmacies de la place.

**P23: IA_01_HGR_AG.rtf - 23:32 [Répondant : en décrivant le sy..] (64:64)	(Super)**

Codes:	[SNIS_fonctionnement_Decrire_pdt MVE]

No memos

Répondant : en décrivant le système d’information sanitaire, nous avons un système qui est national donc le SNIS qui est compilé à tous les niveaux depuis la structure de base jusqu’à la zone de santé et à chaque niveau, il y a une analyse qui est faite et à notre niveau à l’hôpital quand nous faisons la compilation des données sur le canevas SNIS et il nous arrive aussi de faire chaque mois les analyse de nos données pour prendre des décisions qui peuvent nous aider à avancer dans la prise en charge des malades

**P23: IA_01_HGR_AG.rtf - 23:33 [Répondant : cela a bien foncti..] (66:66)	(Super)**

Codes:	[SNIS_fonctionnement_Decrire_pdt MVE]

No memos

Répondant : cela a bien fonctionné et il n’y a pas eu des défis par rapport à ce système d’information. Nous avons fonctionné comme avant

**P23: IA_01_HGR_AG.rtf - 23:34 [Répondant : nous n’étions pas ..] (68:68)	(Super)**

Codes:	[SNIS_fonctionnement_Decrire_pdt MVE]

No memos

Répondant : nous n’étions pas vraiment surchargés jusqu’à manquer le temps de faire le rapport. En tout cas nous avons fait tous les rapports comme tels

**P23: IA_01_HGR_AG.rtf - 23:35 [Répondant : pendant cette péri..] (70:70)	(Super)**

Codes:	[Réunion_pdt-épidémie-Ebola]

No memos

Répondant : pendant cette période, nous à notre niveau on tient la réunion du comité de direction de l’hôpital et on n’a pas vraiment eu des problèmes par rapport à la tenue de ces réunions et nous participons aussi à des réunions de l’équipe cadre. Juste au début comme c’est la première épidémie, on n’avait pas vraiment tenus des réunions comme tel mais après les réunions ont été relancé sans problème et même les réunions de coges ont été relancé donc toutes les réunions ont été relancées sans problèmes même au niveau de l’équipe cadre de la zone de santé mais au niveau de l’hôpital on avait toujours des réunions de CODIR

**P23: IA_01_HGR_AG.rtf - 23:36 [Répondant : par rapport à la g..] (72:72)	(Super)**

Codes:	[Gouvernance_soins santé]

No memos

Répondant : par rapport à la gouvernance, nous sommes membre de l’équipe cadre de la zone de santé et aussi du Coges, la coordination est faite à ce niveau pour que nous puissions exécuter les taches de cette structure qui nous gère et cette structure surtout le Coges, il y a des appâts qui sont des membres pour faire participer la communauté mais aussi il y a le conseil d’administration de la zone de santé qui réunit une grande partie de la communauté, c’est là où la communauté participe à la gestion de tout ce qui a comme structures de santé, dans ses organes la communauté est représentée et c’est là où nous présentons tout ce qui a comme situation de l’HGR de Beni.*

**P23: IA_01_HGR_AG.rtf - 23:37 [Répondant : elle n’a pas été m..] (74:74)	(Super)**

Codes:	[Gouvernance_soins santé]

No memos

Répondant : elle n’a pas été modifié parce qu’on a vraiment été toujours très proche de la communauté pour faire comprendre la maladie qui nous est arrivée

**P23: IA_01_HGR_AG.rtf - 23:38 [Répondant : les supervisions o..] (76:76)	(Super)**

Codes:	[Supervision_pdt-épidémie-Ebola]

No memos

Répondant : les supervisions ont diminué mais nous avons fait beaucoup des supervisions par rapport à la riposte mais les supervisions n’ont pas été bien fait, on a fait seulement des supervisions par rapport à cette maladie qui nous est arrivée

**P23: IA_01_HGR_AG.rtf - 23:39 [Répondant : le changement qu’o..] (78:78)	(Super)**

Codes:	[Souhait_Changement_gestion_futures_épidémies]

No memos

Répondant : le changement qu’on aura voulu pour que les choses avancent comme tel il faudra mettre d’abord les prestataires au même niveau, les prestataires qui travaillent dans les activités d’épidémie et ceux-là qui resteront en train de faire d’autres activités de routine dans les structures de santé, il faudra qu’ils aient la même motivation pour que la perturbation ne soit pas visible dans cette communauté

**P23: IA_01_HGR_AG.rtf - 23:40 [Répondant : autre chose, comme..] (80:80)	(Super)**

Codes:	[Commentaire-Recommandation_Répondant]

No memos

Répondant : autre chose, comme nous n’avons pas des structures qui ne sont pas vraiment équipées et qui sont mal construits, nous souhaiterions que l’on puisse vraiment améliorer le système de santé pour que nous soyons capables de prendre en charge totalement les malades. La prise en charge holistique des malades qui nous viennent parce que nous avons toujours des situations qui nous arrivées et qu’il y a un malade qui nécessite des soins appropriés mais à cause d’une épidémie, on peut nous refuser de le référer vers les structures supérieurs vue ce que nous avons comme matériels dans nos structures de base.

**P23: IA_01_HGR_AG.rtf - 23:41 [Répondant : nous avons été vra..] (82:82)	(Super)**

Codes:	[PCI_Comment_maintenir]

No memos

Répondant : nous avons été vraiment outillés par rapport à la prévention des infections dans nos services et pour pérenniser ces acquis nous souhaiterions que le gouvernement puisse vraiment appuyer le système de santé parce que cette prévention demande beaucoup des moyens pour être pérenniser. Ce que nous avons vu, ce que les partenaires nous ont apporté nous ont permis d’arriver à un niveau qui ne pas à négliger et nous souhaiterions que le gouvernement puisse appuyer réellement les structures parce que les structures sont vraiment délaissées et on ne sait pas comment on peut arriver à maintenir la PCI si on n’a pas des moyens suffisants

**P23: IA_01_HGR_AG.rtf - 23:42 [Répondant : nous remercions la..] (84:84)	(Super)**

Codes:	[Commentaire-Recommandation_Répondant]

No memos

Répondant : nous remercions la personne qui est venue pour nous poser ces questions et nous croyons que ça sera prise en compte dans les différents rapports qu’il va établir et nous aurons vraiment des bons résultats dans l’avenir.

**P24: IA_01_HGR_MCS.rtf - 24:1 [Répondant : Merci de la parole..] (7:7)	(Super)**

Codes:	[Début-Progression_Epidémie MVE]

No memos

**Répondant** : Merci de la parole, quand l’épidémie s’est déclenchée nous sommes mal partis au départ, la preuve en est que même pour organiser le triage il fallait recruter les gens qui ne sont pas de l’hôpital et ç’a posé de problème puis que le triage devrait être former par les gens de l’hôpital, avec le temps nous avons été obligés de voir comment changer l’équipe pour qu’on puisse remettre le service à l’hôpital.

**P24: IA_01_HGR_MCS.rtf - 24:2 [Répondant : Ça n’a pas été cho..] (9:9)	(Super)**

Codes:	[Considération_Ebola_population]

No memos

**Répondant** : Ça n’a pas été chose facile pour la population et surtout que c’est un évènement qui n’a jamais été dans la contraint et on n’avait rien compris, à tel enseigne que dans le milieu périphérique on n’a pas adhéré facilement à la maladie, il y a eu résistance il fallait un long moment pour comprendre ce qu’il y a, en plus ceux qui sont venus en appui, étaient impressionnant avec beaucoup d’argent qui avait attiré l’attention de la population. Les gens commençaient à se demander « depuis longtemps qu’on est en train de nous tuer on n’a jamais vu les gens qui viennent nous assister, comment explique ça ? Avec le temps ils ont fini par comprendre.

**P24: IA_01_HGR_MCS.rtf - 24:3 [Répondant : C’est après qu’on ..] (11:11)	(Super)**

Codes:	[Structures-Services_fonctionnement]

No memos

**Répondant** : C’est après qu’on avait commencé à ferme certaines FOSA qui ne répondaient pas aux normes puis qu’il y avait des malades qui pavaient transiter là et eux les gardent sans donner l’information, cela a favorisé la propagation de l’épidémie par ce que les gens étaient dans l’ignorance de ceux qui se passait.

**P24: IA_01_HGR_MCS.rtf - 24:4 [Répondant : En tout cas toute ..] (13:13)	(Super)**

Codes:	[Structure-Service_Fermeture_pdt MVE]

No memos

**Répondant** : En tout cas toute structure qui n’avait pas leur document d’ouverture était obliger de fermer.

**P24: IA_01_HGR_MCS.rtf - 24:5 [Répondant : Autour d’une dizai..] (15:15)	(Super)**

Codes:	[Structure-Service_Fermeture_pdt MVE]

No memos

**Répondant** : Autour d’une dizaine.

**P24: IA_01_HGR_MCS.rtf - 24:6 [Répondant : Non, tous les serv..] (17:17)	(Super)**

Codes:	[Structures-Services_fonctionnement]

No memos

**Répondant** : Non, tous les services ont fonctionné malgré la présence de l’épidémie.

**P24: IA_01_HGR_MCS.rtf - 24:7 [Répondant : Ça n’a pas été fac..] (19:19)	(Super)**

Codes:	[Structure-Service_Utilisation_population]

No memos

**Répondant** : Ça n’a pas été facile que la communauté vienne se faire soigner d’autant plus que les gens savaient que le centre de traitement d’Ebola été ici et ils avaient l’idée que l’ils venaient à l’hôpital on pouvait les faire passer là. Alors ça été inquiet pour certains de venir se faire soigner ils préféraient aller dans les structures périphériques.

**P24: IA_01_HGR_MCS.rtf - 24:8 [Répondant : Oui c’est vrai, no..] (21:21)	(Super)**

Codes:	[Triage-Isolement-Chlore-thermoflash_Réaction_population]

No memos

**Répondant** : Oui c’est vrai, nous l’avions observé même ici chez nous.

**P24: IA_01_HGR_MCS.rtf - 24:9 [Répondant : Ils avaient en eux..] (23:23)	(Super)**

Codes:	[Triage_Considération_population]

No memos

**Répondant** : Ils avaient en eux que lorsqu’on arrive à l’hôpital on doit nécessairement les transférer au CTE pour eux ils savaient que c’est le mouroir.

**P24: IA_01_HGR_MCS.rtf - 24:10 [Répondant : Non les gens ont c..] (25:25)	(Super)**

Codes:	[Triage-Isolement-Chlore-thermoflash_Réaction_population]

No memos

**Répondant** : Non les gens ont compris ça.

**P24: IA_01_HGR_MCS.rtf - 24:11 [Répondant : En tout cas du cot..] (27:27)	(Super)**

Codes:	[Qualité_soins_Prestataires]

No memos

**Répondant** : En tout cas du coté accueil c’était la même manière de routine qu’on faisait ça. Sauf que l’idée des gens pensaient que s’ils arrivaient on devrait les envoyer au CTE c’était là leurs craintes.

**P24: IA_01_HGR_MCS.rtf - 24:12 [Répondant : Moi suis resté à l..] (29:29)	(Super)**

Codes:	[Répondant_Responsabilité_pdt MVE]

No memos

**Répondant** : Moi suis resté à l’hôpital, je n’ai pas été dans la riposte comme tel je suis resté en train de gérer l’hôpital, mais il y avait des confrères qui y étaient, pour que l’hôpital ne puisse pas être sacrifié moi je jugé bon de rester avec les confrères qui ont voulu rester aussi. Puisque les autres ont trouvé que c’est là qu’on paie mieux.

**P24: IA_01_HGR_MCS.rtf - 24:13 [Répondant : En tout cas en un ..] (31:31)	(Super)**

Codes:	[Ressources_humaines_Disponibilité et perturbations]

No memos

**Répondant** : En tout cas en un moment donné on n’était pas à la hauteur de fonctionner normalement comme d’habitude, on était obligé de prendre certaines personnes de relève pour palier à cette situation et c’est ainsi que nous avions continué à travailler dans cette façon.

**P24: IA_01_HGR_MCS.rtf - 24:14 [Répondant : Personnellement je..] (33:33)	(Super)**

Codes:	[Ressources_humaines_Disponibilité et perturbations]

No memos

**Répondant** : Personnellement je ne suis pas médecin chef de service, je fais l’intérim, le médecin chef de staff est parti dans la riposte, pour ne pas laisser le poste vacant la direction a été obligé de me mettre sur ce poste, comme il y avait d’autres médecins qui étaient partis là aussi, pour bien fonctionner nous avons été obligés de recruter les médecins et les infirmiers des relèves. C’est avec ceux-là que nous avons fait fonctionner dans l’hôpital pour cette période de la riposte.

**P24: IA_01_HGR_MCS.rtf - 24:15 [Répondant : Comme on était dan..] (35:35)	(Super)**

Codes:	[Ressources_humaines_solution-local_Disponibilité et perturbations]

No memos

**Répondant** : Comme on était dans une période de l’épidémie, la première chose était de former tous sur la protection et contrôle des Infections (PCI), tout infirmier qui était à l’hôpital était formé pour savoir comment se protéger, là ça n’a pas posé de problème. Bref toute personne qui était resté ici était formé sur la PCI.000811

**P24: IA_01_HGR_MCS.rtf - 24:16 [Répondant : Oui c’est là où il..] (37:37)	(Super)**

Codes:	[Ressources_humaines_solution-local_Disponibilité et perturbations]

No memos

**Répondant** : Oui c’est là où il y avait un peu de quoique, avec le temps on a trouvé le moyen pour soigner.

**P24: IA_01_HGR_MCS.rtf - 24:17 [Répondant : Je n’ai pas regret..] (39:39)	(Super)**

Codes:	[Ressources humaines_Regret de non implication dans la riposte]

No memos

**Répondant** : Je n’ai pas regretté de n’avoir pas parti, j’avais resté ici à l’hôpital si tout le monde devait partir on ne devait pas faire fonctionner l’hôpital avec les gens de relèves. Vous voyez ce que ça pouvait donner comme image. J’avais le souci de sauvegarder l’image de l’hôpital. Tout en sachant que la riposte finira un jour et si on	avait tous abandonné l’hôpital ça ne donnerait pas une bonne connotation.

**P24: IA_01_HGR_MCS.rtf - 24:19 [Répondant : En tout cas la qua..] (43:43)	(Super)**

Codes:	[Gratuité_Conséquences]

No memos

**Répondant** : En tout cas la qualité n’a pas été bonne puisque le flux de malades ne permettait pas qu’on puisse offrir une bonne qualité des soins.

**P24: IA_01_HGR_MCS.rtf - 24:20 [Répondant : En tout cas je sou..] (45:45)	(Super)**

Codes:	[Souhait_Changement_gestion_futures_épidémies]

No memos

**Répondant** : En tout cas je souhaiterai la situation pareille ne revienne plus, mais avec l’expérience nous saurons comment surmonter si cela arrivait encore.

**P24: IA_01_HGR_MCS.rtf - 24:21 [Répondant : En tout cas nous a..] (41:41)	(Super)**

Codes:	[Gratuité_Conséquences]

No memos

**Répondant** : En tout cas nous avons été très très submerger à cette époque-là, on pouvait consulter même plus de cent malades, on a fait ce qu’on pouvait faire à cette période.

**P24: IA_01_HGR_MCS.rtf - 24:22 [Répondant : C’est normal que c..] (47:47)	(Super)**

Codes:	[Considération_prestataire_EPI_population]

No memos

**Répondant** : C’est normal que cette population puisse trouver que ce n’était pas bon. C’est la toute première fois que nous vivons l’évènement, avec la modernisation ils de devaient comprendre ça mal. Ils ne sont pas habitués de voir les gens s’habiller de la sorte. Quant à nous les prestataires c’était une façon de nous protéger, on ne devait pas se laisser faire et se mettre à la portée de la maladie.

**P24: IA_01_HGR_MCS.rtf - 24:23 [Répondant : En tout cas nous s..] (49:49)	(Super)**

Codes:	[Traitement_Invasifs_Prestataire_changement]

No memos

**Répondant** : En tout cas nous suivons le programme national toujours, on utilise les médicaments essentiels génériques et comme nous sommes un hôpital de l’Etat. Nous avons déjà notre protocole thérapeutique, on n’a pas tendance de quitter le protocole.

**P24: IA_01_HGR_MCS.rtf - 24:24 [Répondant : En tout cas ces so..] (51:51)	(Super)**

Codes:	[Traitement_Invasifs_Prestataire_changement]

No memos

**Répondant** : En tout cas ces sont les per os c’est ce qui est privilégié pour le moment ici, les injections on ne donne pas tellement.

**P24: IA_01_HGR_MCS.rtf - 24:25 [Répondant : C’est un peu un ch..] (53:53)	(Super)**

Codes:	[Traitement_Invasifs_Prestataire_changement]

No memos

**Répondant** : C’est un peu un choix, nous avons trouvé mieux pour ne pas se piquer, pour ne pas créer des accidents.

**P24: IA_01_HGR_MCS.rtf - 24:26 [Répondant : Pas nécessairement..] (55:55)	(Super)**

Codes:	[Traitement_Invasifs_Prestataire_changement]

No memos

**Répondant** : Pas nécessairement, ici chez nous privilégions le per os 001324

**P24: IA_01_HGR_MCS.rtf - 24:27 [Répondant : En tout cas à l’ép..] (57:57)	(Super)**

Codes:	[Traitement_Invasifs_Prestataire_changement]

No memos

**Répondant** : En tout cas à l’époque de la riposte on avait réduit les cas de sergium, on n’opérait pas tout le temps, on prenait seulement les cas qui étaient urgent, les surgis programmés on avait cherché isave on a pas continué avec ça pour éviter tout éventualité de contamination.

**P24: IA_01_HGR_MCS.rtf - 24:28 [Répondant : Oui avec le PDSS ç..] (59:59)	(Super)**

Codes:	[Médicaments_disponibilité_fosa]

No memos

**Répondant** : Oui avec le PDSS ça marchait bien, c’est avec ALIMA où on avait un petit souci pour les médicaments.001423

**P24: IA_01_HGR_MCS.rtf - 24:29 [Répondant : Depuis que nous so..] (61:61)	(Super)**

Codes:	[Médicaments_disponibilité_fosa]

No memos

**Répondant** : Depuis que nous sommes avec ALIMA il y a beaucoup de médicaments qu’on ne parvenait pas à nous fournir pour donner aux malades, puis qu’il y a une gratuité partielle avec ALIMA

**P24: IA_01_HGR_MCS.rtf - 24:30 [Répondant : Oui] (63:63)	(Super)**

Codes:	[Médicaments_disponibilité_fosa]

No memos

**Répondant** : Oui

**P24: IA_01_HGR_MCS.rtf - 24:31 [Répondant : Pas encore, avec P..] (65:65)	(Super)**

Codes:	[Gratuité_Rumeurs]

No memos

**Répondant** : Pas encore, avec PDSS il n’y avait pas de soucis tout le monde était satisfait.

**P24: IA_01_HGR_MCS.rtf - 24:32 [Répondant : Avec PDSS on était..] (67:67)	(Super)**

Codes:	[Gratuité_Conséquences_motivation_prestataire]

No memos

**Répondant** : Avec PDSS on était bien primé, tout le monde était satisfait.

**P24: IA_01_HGR_MCS.rtf - 24:33 [Répondant : C’a été perturbé p..] (69:69)	(Super)**

Codes:	[SNIS_fonctionnement_Decrire_pdt MVE]

No memos

**Répondant** : C’a été perturbé par l’Ebola, il avait des malades qui devait quitter le CTE non cas, tant tôt on les amenait dans un état critique arriver ici ils décédaient alors ça augmenté le taux mortalité inter hospitalière et ça ne nous a pas arranger. Puis qu’avec tout cas qui pouvait présenter des signes en rapport avec Ebola partait là(CTE), arriver là-bas on peut les garder longtemps et la santé va encore se détériorer alors ç’a perturbé notre complétude, on a connu beaucoup de cas pareils.

**P24: IA_01_HGR_MCS.rtf - 24:34 [Répondant : Nous avons été obl..] (71:71)	(Super)**

Codes:	[Organisation_travail-Circuit des malades_pendant MVE]

No memos

**Répondant** : Nous avons été obligés d’aller vers eux et leurs dire que s’il faut transférer il faut le faire à temps et ne pas nous amener les malades qui sont déjà morts, on a connu beaucoup de cas de décès.

**P24: IA_01_HGR_MCS.rtf - 24:35 [Répondant : Tant tôt de mycoti..] (73:73)	(Super)**

Codes:	[Organisation_travail-Circuit des malades_pendant MVE]

No memos

**Répondant** : Tant tôt de mycotique, des hypertendus qu’on a amené là-bas, voire même les cas des ascites, on se demandait s’il avait de rapport avec la riposte pour qu’ils puissent se retrouver là.

**P24: IA_01_HGR_MCS.rtf - 24:36 [Répondant : Moi je crois que c..] (75:75)	(Super)**

Codes:	[Organisation_travail-Circuit des malades_pendant MVE]

No memos

**Répondant** : Moi je crois que c’était le problème de gens qui faisaient l’investigation, ils n’étaient pas allés aux cibles.

**P24: IA_01_HGR_MCS.rtf - 24:37 [Répondant : Oui puis que quelq..] (77:77)	(Super)**

Codes:	[Organisation_travail-Circuit des malades_pendant MVE]

No memos

**Répondant** : Oui puis que quelqu’un lui voyait qu’il est hypertendu ou diabétique et vous l’assimilez aux gens qui doivent être là-bas vous voyez ce que ça donne ? Par ce qu’il y avait des gens qui arrivaient en comment et comme tous comment doivent y aller, ça n’arrangeait pas les choses.

**P24: IA_01_HGR_MCS.rtf - 24:38 [Répondant : C’est de vous reme..] (79:79)	(Super)**

Codes:	[Commentaire-Recommandation_Répondant]

No memos

**Répondant** : C’est de vous remercier et s’il y a à améliorer ça va venir avec le temps, que ça ne soit pas la dernière fois de faire un travail pareil.

**P24: IA_01_HGR_MCS.rtf - 24:39 [Répondant : S’il faut qu’il ai..] (81:81)	(Super)**

Codes:	[Souhait_Changement_gestion_futures_épidémies]

No memos

**Répondant** : S’il faut qu’il ait changement, il faut qu’on mette au triage les gens qui méritent et non mettre n’importe qui ; par ce qu’on était mal parti au début quand l’épidémie s’était déclenchée. Par ce qui il avait des gens qui étaient mis là par ce que leurs frères appellent et ça ne nous avait pas arrangé.

**P24: IA_01_HGR_MCS.rtf - 24:40 [Répondant : Il y a eu même les..] (83:83)	(Super)**

Codes:	[Souhait_Changement_gestion_futures_épidémies]

No memos

**Répondant** : Il y a eu même les non professionnels de santé qui y étaient, mais prochainement si cela arrivait qu’on mette quand même les gens qui méritent.

**P24: IA_01_HGR_MCS.rtf - 24:41 [Répondant : Il faut les corps ..] (85:85)	(Super)**

Codes:	[Souhait_Changement_gestion_futures_épidémies]

No memos

**Répondant** : Il faut les corps soignants qu’on connait très bien. Puis que à l’époque on recrutait n’importe qui.

**P24: IA_01_HGR_MCS.rtf - 24:42 [Répondant : Malheureusement av..] (87:87)	(Super)**

Codes:	[PCI_Comment_maintenir]

No memos

**Répondant** : Malheureusement avec l’arrivé d’ALIMA lorsqu’il a créé la gratuité on a encore rentré à la case de départ. 002012

**P24: IA_01_HGR_MCS.rtf - 24:43 [Répondant : Déjà commencer à p..] (89:89)	(Super)**

Codes:	[PCI_Comment_maintenir]

No memos

**Répondant** : Déjà commencer à passer nuit par terre. Hors c’était déconseillé, c’est avec l’avènement du Covid 19 qu’on a créé un autre mécanisme en disant que toute personne qu’arrivera ici doit avoir une note de référence de son centre. C’est ainsi que vous ne voyez pas d’engouement ici.

**P24: IA_01_HGR_MCS.rtf - 24:44 [Répondant : Nous sommes en tra..] (91:91)	(Super)**

Codes:	[PCI_Comment_maintenir]

No memos

**Répondant** : Nous sommes en train de nous forcer, nous continuons à appliquer ça, on se protège toujours. Peut-être on aura les difficultés s’il arrivait que l’équipement manque. Par ce que si on a encore d’équipements c’est puisqu’il y a encore les partenaires. Lorsque les partenaires vont se retirer on ne serait pas à hauteur d’en trouver, d’autant plus se sont des choses qui coutent extrêmement cher.

**P25: IA_01_ONG-PTF_COORDO.rtf - 25:1 [Répondant : Au début, je savai..] (6:6)	(Super)**

Codes:	[Fosa-Institution_Présentation]

No memos

**Répondant** : Au début, je savais bien à commencer depuis 2018 alors ADR était déjà dans la zone, alors c'est à partir du 2014 les événements se sont progressés dans la zone de santé alors à ce qui est des structures qu'on a supportées. Je ne me suis même pas présenté je suis le manageur de santé. Alors quand l'évènement à commencer l'épidémie à virus Ebola qui s'est ajouter parmi les 7 évènements, on a aussi fait de plaidoyer de façon que en dehors de ce que nous faisons et qu'on puisse aussi mener certaines études par rapport aussi à la riposte alors comme vous êtes plus intéresser par le soins de santé primaire nous sommes parvenu aussi à décrocher un projet, donc en dehors de ce qu'on peut faire dans la PCI mais aussi le soins de santé s'est ajouter mais pour ça on a soutenu seulement trois structures dans la zone de santé de Kalunguta et une structure dans la zone de santé de Beni qui est dans la zone de santé de Kahamba

**P25: IA_01_ONG-PTF_COORDO.rtf - 25:2 [Répondant : A Beni c'est la st..] (10:10)	(Super)**

Codes:	[Fosa-Institution_Présentation]

No memos

**Répondant** : A Beni c'est la structure de Beni, alors en dehors de cette structure le soins de santé primaire vient maintenant au besoin de la population et ce qu'on faisait d'habitude on a 8 structures que nous soutenons en soins de santé primaire et le 4 sont dans la zone de santé de avec l'évènement qui se passe là il y a la population qui fuit et on ne peut pas intervenir là-bas donc là nous avons 4 structures qui sont: la structure de zone de santé la... nous intervenons aussi à Eringeti par ce que le mois passer on a changé la structure kansanga.

**P25: IA_01_ONG-PTF_COORDO.rtf - 25:3 [Répondant : Depuis, et à Beni ..] (18:18)	(Super)**

Codes:	[Fosa-Institution_Présentation]

No memos

**Répondant** : Depuis, et à Beni nous avons le structure sayon et à Kalunguta nous avons maintenant par ce que le 4 nous avons une structure à Beni et le trois à Kalunguta par ce que vous savez le deux que nous soutenons le centre de santé de mapuka comme on était aussi plus da Oicha c'est à partir du mois d'octobre qu'on s'est désengager pour raison d'insécurité le milieu était inaccessible il y avait de difficulté malgré qu'il y a n'a besoin on s'est désengagé.

**P25: IA_01_ONG-PTF_COORDO.rtf - 25:4 [Répondant : c'est la gratuité ..] (26:26)	(Super)**

Codes:	[Gratuité_Structures-Services_concernés]

No memos

**Répondant** : c'est la gratuité totale.

**P25: IA_01_ONG-PTF_COORDO.rtf - 25:5 [Répondant : Exactement.] (30:30)	(Super)**

Codes:	[Gratuité_Structures-Services_concernés]

No memos

**Répondant** : Exactement.

**P25: IA_01_ONG-PTF_COORDO.rtf - 25:6 [Répondant : Nous donnons d’abo..] (34:34)	(Super)**

Codes:	[Gratuité_Structures-Services_concernés]

No memos

**Répondant** : Nous donnons d’abord le médicament essentiel et à part le médicament essentiel nous donnons aussi au personnel.

**P25: IA_01_ONG-PTF_COORDO.rtf - 25:7 [Répondant : Nous soutenons aus..] (38:38)	(Super)**

Codes:	[Gratuité_Structures-Services_concernés]

No memos

**Répondant** : Nous soutenons aussi le gouvernement par le médicament, nous soutenons aussi en équipement.

**P25: IA_01_ONG-PTF_COORDO.rtf - 25:8 [Répondant : Bon le structures ..] (42:42)	(Super)**

Codes:	[Structures-Services_fonctionnement]

No memos

**Répondant** : Bon le structures qui ont connues d'abord nous avons la structure de kahama c'est une structure ou nous avons aussi besoin de mais c'est une structure qui est moins utiliser et la population ne l'utilise pas vraiment mais de qui a gratuité il utilise ça difficilement et par totalité ils ont compris que nous sommes venu le aider pour que la maladie ne puisse pas se propager mais par les équipes de sensibilisation les gans comprennent petit à petit, en tout cas il n'utilise pas suffisamment la structure.

**P25: IA_01_ONG-PTF_COORDO.rtf - 25:9 [Répondant : A Mukuya on a eu d..] (46:46)	(Super)**

Codes:	[Gratuité_Conséquences_utilisation_services]

No memos

**Répondant** : A Mukuya on a eu de la chance par ce que c'est une structure qu'on utilisée et qu'on appuyer avant Ebola et c'était sur utiliser aussi mais à un certain niveau quand on est venu avec Ebola tout le monde actuellement commence à comprendre et puis-je dire depuis janvier qui y a quand même provoquer l'utilisation qui a augmenter aussi par rapport à la sensibilisation mais le gens semble être un peu retissant d'utiliser seulement la structure comme ça se faisait avant par ce que Ebola le gens pensaient que c'est autre chose ainsi de suite ils voulaient lié ça à la politique. En dehors de ça il y a eu d'autre structures alors d'abord comme le mois de septembre, octobre 2018 novembre, décembre presque toutes les structures qu’on a appuyé dans la zone de santé de Beni et Kalunguta il y a eu vraiment diminution d'utilisation de service interne par ce que c'était au moins à un moment où il faillait installer le système de triage au sein des structures alors ça toucher vraiment beaucoup de service et les gens se sont rétracté.

**P25: IA_01_ONG-PTF_COORDO.rtf - 25:10 [Répondant : Rien que par ce qu..] (50:51)	(Super)**

Codes:	[Triage_Considération_population]

No memos

**Répondant** : Rien que par ce qu’il pensait que si je développe une fièvre on m'amène directement au niveau du CTE, il pensait que de qu'on est suspect c'est pour aller à la structure de traitement ou de transit. C'est comme ça qu'ils avaient un peu de réserve pour fréquenter la structure.

001452

**P25: IA_01_ONG-PTF_COORDO.rtf - 25:11 [Répondant : Les structures des..] (54:54)	(Super)**

Codes:	[Structure-Service_Utilisation_population]

No memos

**Répondant** : Les structures des activités préventives d'abord en octobre, septembre, octobre et novembre il y a eu un courant là où on se disait même que c'est ne te pas facile d'aller les grouper les mamans ainsi de suite on a commencé à vacciner là il y a eu presque le gens ne s'est sont pas intéressé à ces activités même au niveau de prestataires on avait même de confusion que tel est comme ça et l'autre ne pas comme ça, c'est ne que vers décembre ou les gens ont commencé à se ressaisir pour dire non on va prendre de précaution et commencer à réunir les gens, ou on pourrait même commencer à vacciner et prendre de mesures de précaution pour qu'on ne puisse pas attraper la maladie et c'est vers novembre et décembre que les gens n'ont repris leurs activités. Alors les gens même actuellement se rare de trouver le gens qui n'ont pas compris que non on va même pas donner ce vaccin-là par ce que le milieu à connue beaucoup d'histoire ils pensaient que quand on commençait les appelle venait il y a déjà de mesure qu'on va prendre il va dire non on va nous donner le vaccin contre Ebola et par conséquent c'est un vaccin qui vient pour nous tuer, c'est comme ça que la population pensait avant mais a partir de sensibilisation et on a un service d'engagement communautaire, ils sont entré dans les villages et essayer de sensibiliser le gens et ils doivent comprendre que les activités préventives doivent continuées et le vaccin qu'on le donne c'est son de vaccins de routine donc là les activités étaient comme ça.

**P25: IA_01_ONG-PTF_COORDO.rtf - 25:12 [Répondant : vous savez la situ..] (58:58)	(Super)**

Codes:	[Qualité_Soins_Patients]

No memos

**Répondant** : vous savez la situation même aujourd'hui c'est un peu complexe l'accueil juste au départ les prestataires avaient beaucoup de réserver au profit des utilisateurs de service alors parfois par ce que les malades pouvaient faire beaucoup de temps sans être pris en charge par ce que on se réservé. Ça aussi c'est un élément qui pouvait faire à ce que les gens se disent par ce qu’il faut que l'infirmier porte des épis et bien sur les épis légers par ce que c'est n'était pas l'habitude alors les gens ou la population avait vu, voilà ça ressemble à autre chose là ça change de carte donc ils disent que nous sommes venus les exterminer et moi quand j'arrive on me dit on faisait pas ce ci mais voilà on commence à se cagouler et porter de gant quand on veut toucher un malade. c'était ça une idée qui a fait qui est un engagement communautaire par ce que même sans Ebola on ne pouvait pas soigner le gens comme ça mais n'est fixe porter des épis et des éléments de protection donc c'est un peu ça il y a eu un petit souci par rapport et les gens qui craignaient et surtout s'il s'agit de faire passer le gens dans le triage et leur thermomètre là le gens se disaient que ça devient autre chose et quand tu vas tenir voilà tu vas passer de l'autre côté ça créer beaucoup de frustration à la population et on a commencé à prendre de réserver donc ils ont beaucoup utilisé bon consulter les professionnels privés et c'est comme ça que d'autres sont contaminer donc quand il a peur d'aller là mais il va aller a hier chez leur membre de famille ou chez les tradi-praticiens et il s'est fait contaminer donc c'est comme ça qu'il y a eu développement de l'épidémie .

**P25: IA_01_ONG-PTF_COORDO.rtf - 25:13 [Répondant : C'est une situatio..] (62:62)	(Super)**

Codes:	[Ressources_humaines_Disponibilité et perturbations]

No memos

**Répondant** : C'est une situation qui à perturber suffisamment c'est quelque chose que nous allons même pas négliger quand l'épidémie est arriver on a travaillé avec, il y a de gans qui sont venu par ce que c'est une maladie ou on avait pas assez d'information alors il fallait qui est le gens d'a hier par ce que c'est un défis mais toute parti au bon moment ou on était dans de structures et lorsque les aires de santé étaient entre d'être progressivement intègre dans le système ou il y a de cas où il fallait passer à introduire le soins gratuité dans de structures ça créer beaucoup d'ennuie par ce que a un moment donner même si je dirais d'abord que le système de santé était déjà un peu par terre par ce que il y a des structures ou il peut faire toute une journée sans qui est même un malade un nouveau cas qui se présente alors à partir de l'accueil qu'on a donné et les gens ont commençaient avenir fréquenter les structures. A ce qui est de ressources humaines on retrouve même jusqu'aujourd'hui si tu entres dans une structures tu vas trouver soit IT on va te dire il est soit tel part donc il est utilisé par plusieurs partenaires surtout dans le cadre de la riposte jusqu'à ce que même les acteurs par ce que nous nous faisons un appui institutionnel et quand il arrive tu peux même faire une correspondance pour le répondant par ce que il est soit avec l'organe de sensibilisation ils sont partie avec tel organisation par ce que là il y a de gens qui paye mieux plus que nous si on donne un petit forfait là ce que on va partager et le jardinier tout le monde la mais là c'est juste pour lui, alors bon nombre de staff avait tendance à s'aligner derrière ça et il pouvait faire toutes les activités et par conséquent ça beaucoup influencer l'accueil dans le structure par ce que tu vas trouver quelqu'un faire l'horaire de la journée et les malades va trouver qu'il n'y a qu'une personne qui s'occupe de ces gens et parfois aussi fatigué il va à hier et il fait autre chose et il y a de ces gens qui faisaient l'activité partout et d'autres aussi ont démissionné ils ont laissé les structures pour aller se faire recruter dans des organisations et parfois nous qui avons vécu fait de fois on regrette par ce que on te forme pour la prise en charge de soins de santé primaire et on va aller retrouver ils s'intéressent à autres chose et ça créer de fois un désagrément peut être on amène quelqu'un autre qui a un niveau peut être bas alors nous avons sentir nous avons trouvé que pour la structure le prestataires pour les histoire de qualité comme on faisait avant par ce que vous allez trouver que les jeunes qu'on avait investi dans ne sont plus la ils arrivent à une structure comme un visiteur et il rentre il est entre de chercher à hier.

**P25: IA_01_ONG-PTF_COORDO.rtf - 25:14 [Répondant : Le système sanitai..] (66:66)	(Super)**

Codes:	[SNIS_fonctionnement_Decrire_pdt MVE]

No memos

**Répondant** : Le système sanitaire il y a un temps par rapport à ce qui ont vérifié vraiment il y a eu un retard par exemple la transmission de mapipi on a trouvé que bon à un moment il faut rappeler le gens qui n'ont pas encore transmit leur mapipi et qu'il devrait envoyé le lundi pourquoi par ce que ils sont occupés ils font autres choses ainsi de suite dans de structure donc sa c'est un dossier même nous avons besoin de canevas SINIS tu vas retrouver qu'il arrive le 10 et la structure n'a pas encore complétée et parfois nous parvenons avec nos superviseurs on se dit bon il faut investir pour que tu es aussi le donnée et transmettre le donnée aussi à nous et que aussi il puisse avoir leur donnée qu'il va transmettre au niveau de bureaux centraux et tu trouves que c'est un peu complexe ou soit tu peux trouver un canevas et tu trouves que ça était compléter avec légèreté et quand tu rechercher ce que tu voulais voir intérieurement tu vas te demander s'il a puisait ces données où et c'était vraiment un peu superficiel par ce que il ne sont pas assis dans un consensus et tous les aires de santé ont étaient vraiment sollicité dans ces histoires, et ça ne peut pas dire même franchement le bureaux centraux manque de données jusqu'à présent comme des humanitaires tu verras jamais le médecin chef de zone il y a parfois de superviseurs conjointes rien du tout là où vous avez était donnée nous le rapport

**P25: IA_01_ONG-PTF_COORDO.rtf - 25:15 [Répondant : Les réunions de mo..] (70:70)	(Super)**

Codes:	[Réunion_pdt-épidémie-Ebola]

No memos

**Répondant** : Les réunions de monitorage ça commence encore mais à parement ça s'est tiennent il y a là où on participe et là où on ne participe pas comme c'est sont beaucoup plus investi dans les histoires interne a hier par rapport au centre là on participer et par après bien sûr il y a aussi comme il y a eu des cas chez eux ils tiennent des réunions ou tu peux voir peut-être il y a de délégations mais l'IT ne va pas venir il est à hier dans de formation ainsi de suite, de formation liée au riposte ainsi de suite on ne condamne plus personne chaque aussi à sa part de responsabilité .

**P25: IA_01_ONG-PTF_COORDO.rtf - 25:16 [Répondant : De façon global la..] (74:74)	(Super)**

Codes:	[Médicaments_disponibilité_fosa]

No memos

**Répondant** : De façon global la disponibilité de médicament on dirait essentiel oui était disponible mais vous savez un élément qui est capital c'est par ce que les gens la conception et si demande a un malade il te diras non le injection par exemple hors nous avons eu de mesure limiter de médicament essentiel et chaque devra aussi respecter ce qu'il devrait faire mais dans le temps on faisait de perfusion même quelqu'un qui est entre de se promener tu sens qu'on est juste limiter et restreindre toutes ces injections alors si vous évaluer au niveau de la communauté vous risquerez de créer pretence mais comme vous êtes technicien vous pouvez entre dans les structures pour les gammes qui sont essentiels vous allez trouver qu'il n'y a na et la population semblait sentir comme si situation à changer et en tout cas les perfusions on s'est dit non au niveau de centre de santé mais il faut que ça soit à l'hôpital mais ce qu'il faisait avant c'est tout le monde qui devrait fréquenter la structure et on aller piquer toujours mais ce changement-là a fait sentir que le bénéficier semble comme si il n'y a plus le médicament hors il y a le médicament par ce que en dehors de mon structure que nous appelons aussi la banque mondial à appuyer les autres structures par ce que la banque mondial était à gauche et à droite donc partout il y avait l'équilibre différemment de ce qu'on trouver avant dans les appuis des urgences liée au mouvement de la population. On a appuyé ici l'aire de santé voisine et ainsi de suite.

**P25: IA_01_ONG-PTF_COORDO.rtf - 25:17 [Répondant : Bon peut-être je p..] (82:82)	(Super)**

Codes:	[Souhait_Changement_gestion_futures_épidémies]

No memos

**Répondant** : Bon peut-être je pourrais dire que la situation que nous avons un peu connue au moment donner je retrouvais que les ONG, mais nous comme organisation qui vient appuyer le gouvernement on se dit que on doit toujours s'aligne derrière le gouvernement mais quelque part on a manqué cette coordination par ce que avant la coordination des ONG était chez OCHA mais quand la maladie est arriver on a dit non tout le monde qui part là c'est ne plus chez OCHA ça devient maintenant la coordination de la maladie et tout le monde donc OCHA n'avait plus la même mis sur les organisation donc tu allais quitter tel est autre part demain il ne plus là toi aussi tu peux venir et après on va te dire que l'autre est déjà là on se dirait que c'est ne plus de OCHA mais la coordination de la maladie maintenant c'est le gouvernement qui aller nous accompagné et je pense que il y a une crise aussi au bureaux centraux et qui sont sensé aussi conduire les activités et les gens semblaient de démissionner aux ordres que nous avons dit en disant que nos activités restent à routine pour quoi par ce que s'il ne se lave pas les mains les épidémies vont venir, la rougeole alors gagnons le temps de mettre ce ci et aussi les crises qui arrivent. les acteurs qui serons visibles il faut que ça soit le acteurs qui sont connu mais quand la situation est arrivée et c'est ça qui a un peu perturber les têtes des gens la population devrait voir quelqu'un qui vient piquer une personne il vient pour la vaccination c'est un quelqu'un qu'on a jamais vu c'est un quelqu'un qui parle une langue étrangère il ne s'est comprend même pas il parle français à un villageois ils ont dit c'est gens sont venu nous tué vraiment donc à créer une résistance et on se dit bon vous pouvez prendre des acteurs de terrain qui connait injecter que tout le monde connait et voilà protéger vous comme tout l monde se protège et donc ça était un peu compliquer en tout cas ça créer beaucoup de résistance.

**P26: IA_01_ONG-PTF_SUP.rtf - 26:1 [Répondant : je suis dans une o..] (2:2)	(Super)**

Codes:	[Répondant_Responsabilité_habituelles]

No memos

Répondant : je suis dans une organisation internationale qui intervient dans le secteur de la santé et nous avons plusieurs volets que nous appuyons dans le secteur de santé. Nous avons d’abord les soins de santé primaire que nous appuyons dans certaines structures et dans le paquet soins de santé primaire, vous voyez qu’il y a beaucoup d’activités, ce que je dirai que ce qui est essentiel, nous octroyons les soins gratuits à la population dans les aires de santé ciblées et nous appuyons les prestataires en capacitation pour des thèmes qui concerne la santé. Il y a certains thèmes que nous pouvons donner et nous faisons aussi la réhabilitation des infrastructures au niveau des structures que nous avons ciblées et nous appuyons aussi, il y a le paquet Wash et dans le paquet Wash, nous pouvons faire la construction des sources, faire le captage des sources amenées au niveau des structures, nous pouvons faire des forages dans les endroits où on ne peut pas trouver des sources aménagées et nous pouvons doter la structure en rapport avec tout ce qui est comme l’hygiène, les latrines donc tout ce qui est en rapport avec l’hygiène, c’est le Wash dans nos structures de santé et à part ça il y a cash que nous donnons pour l’appui personnel qui s’occupe des bénéficiaires et nous pouvons donner le lot des médicaments aux structures pour que la population accède aux soins de santé gratuit.

**P26: IA_01_ONG-PTF_SUP.rtf - 26:2 [Répondant : Oui, et comme je d..] (4:4)	(Super)**

Codes:	[Répondant_Responsabilité_pdt MVE]

No memos

Répondant : Oui, et comme je disais que nous sommes dans les soins de santé primaire et comme l’épidémie a touché le secteur de la santé, nous avons fait beaucoup des choses en donnant d’abord l’accès gratuit aux soins à toute la population dans les structures que nous avons ciblées pour permettre à ce que les gens ne restent pas à la maison car nous livrons le paquet de soins à toute la population même celui qui resterait à la maison avec une maladie aura tendance de venir dans une structure et là on va dépister qu’en tout cas vous mériter d’être isoler parce qu’il y a ceci ou cela ; donc dans nos structures nous avons déjà mis des dispositifs pour faire le dépistage, tout malade qui entre doit se faire dépisté pour voir son état de santé et si on trouve qu’il a certains symptômes qui peuvent être relatifs à l’épidémie, aussitôt on peut le mettre à l’isoloir et faire appel à l’équipe spécialisée pour que l’ambulance le prenne et l’amène au centre de traitement. Nous avons aussi une équipe qu’on appelle « engagement communautaire » qui dans la communauté, dans les aires de santé que nous appuyons pour essayer de leur parler de cette maladie, il y a eu beaucoup des barrières, au début l’épidémie n’a pas été bien gérer et ça fait qu’il y ait trop des barrières dans la communauté et c’est l’une des difficultés que j’allais dire quand on allait pour les soins gratuits on disait est-ce que ces soins, ce n’est pas Ebola que vous voulez nous amener, pourquoi pendant combien des jours, on n’a pas eu la gratuité des soins et c’est aujourd’hui que vous nous amenez la gratuité des soins avec tous ces médicaments, est-ce que ce n’est pas Ebola que vous nous amenez, c’est ce que la communauté était en train de dire. Pour briser ça, il a fallu avoir des équipes pour aller sensibiliser et expliquer qu’est-ce que la maladie et qu’est-ce que ça peut amener, alors au fur et à mesure nos équipe qu’on appelle engagement communautaire passait dans la communauté, ça pouvait déjà diminuer les mauvaises informations dans la communauté et la population commençait déjà à fréquenter les structures

**P26: IA_01_ONG-PTF_SUP.rtf - 26:3 [Répondant : c’est ce que je di..] (6:6)	(Super)**

Codes:	[Considération_Ebola_population]

No memos

Répondant : c’est ce que je disais au début la stratégie n’était pas bonne, il y avait des gens qui devaient venir de Kinshasa qui parlent d’abord moins la langue locale et qui ne maitrisent pas le milieu et alors ils vont parachuter dans la communauté avec le lingala et le français là très compliqué, vous venez dans la communauté et vous parlez d’Ebola, on va dire que c’est vous qui nous amenez votre Ebola ici. Il fallait maintenant chercher les gens locaux pour accompagner les équipes des experts qui sont venus alors ce sont ces gens locaux qui sont capables de persuader la communauté, peut-être que lui ne connait pas la langue mais il est là pour nous assister parce que c’est nous qui sommes victime et c’est nous qui perdons nos frères et c’est nous qui mourrons et lui son rôle est de venir nous appuyer, svp aider nous a accepté que c’est arrivé donc au début la situation n’était pas facile à gérer parce que la stratégie n’était pas bonne au début parce qu’on a amené des gens qui sont venus d’ailleurs, les gens qui ne maitrisaient pas et c’est ce qui a fait que les gens créent un peu des résistances mais au fur et à mesure qu’on a changé la stratégie et intégrer les personnes qu’on connait mais avec les leaders communautaire, les gens ont commencé à comprendre que c’est une situation qui existe et ça peut faire à ce que nous puissions perdre les membres de nos familles et après les gens se sont approprié l’affaire et ils ont compris que c’est sérieux

**P26: IA_01_ONG-PTF_SUP.rtf - 26:4 [Répondant : voilà, ce que je d..] (8:8)	(Super)**

Codes:	[Structures-Services_fonctionnement]

No memos

Répondant : voilà, ce que je dirai au début comme on avait déjà cet aspect de soins gratuits bien que ce n’était pas pour longtemps, on pouvait faire trois mois de projet de gratuité de soins et là la population comme il n’y avait pas encore cet aspect Ebola, la population était contente que nous leur apportons un soutien vraiment. Quand Ebola est venu quand nous c’était déjà l’accoutumance et ça fait que d’ailleurs que nous soyons vite accepté même dans la suite de la riposte alors là il n’y avait pas trop des problèmes pour nous mais on ne pouvait faire seulement trois mois mais quand Ebola est arrivé, la gratuité a fait pendant tout le temps qu’on était dans les structures, au lieu de faire cette phase de trois mois, gratuité, on réduit et c’est qui est comme impact positif sur les infrastructures pour le personnel même la communauté, il y avait des habitudes, qui étaient presque se laver les mains et on pouvait voir quelqu’un qui pouvait dire que je veux me laver les mains et qu’est-ce que je veux manger ? mais au fur et à mesure qu’ils ont été sensibilisé maintenant les gens comprennent que se laver les mains, c’est une habitude qu’il faut acquérir non seulement quand on veut manger mais c’est pour se protéger soi-même, ça c’est vraiment quelque chose que nous avons acquis mais sur le plan infrastructure, il y a des infrastructures qui existaient où on trouvait que deux portes des toilettes mais avec la maladie, avec le Wash, toutes les structures sont maintenant appuyer et vous pouvez voir qu’il y a 5 ou dix portes pour les installations hygiéniques et il y a même d’autres où on a fait des constructions qui n’étaient pas envisagés mais pour éviter que les gens soient contaminés mais on a essayé d’élargir des endroits pour l’hospitalisation, pour la maternité, ça c’est un avantage et vous allez voir que le personnel qui avait des petites primes là, presque maudite mais avec l’arrivée de la MVE, la prime a presque triplée et d’ailleurs c’est ça même la crainte après l’épidémie, les gens qui étaient déjà habitués à des grandes sommes vont revenir que la gamme du petite prime, est-ce que les gens seront encore motivés, voilà encore un autre défi

**P26: IA_01_ONG-PTF_SUP.rtf - 26:5 [Répondant : les changements tr..] (10:10)	(Super)**

Codes:	[Structure-Service_Utilisation_population]

No memos

Répondant : les changements très, très énormes, c’est ce que je disais, le taux d’utilisation qui était à 20% est allé à plus de 100%, c’est ce qui est un avantage

**P26: IA_01_ONG-PTF_SUP.rtf - 26:6 [Répondant : parce que c’est gr..] (12:12)	(Super)**

Codes:	[Structure-Service_Utilisation_population]

No memos

Répondant : parce que c’est gratuit

**P26: IA_01_ONG-PTF_SUP.rtf - 26:7 [Répondant : au début, les gens..] (14:14)	(Super)**

Codes:	[Structure-Service_Utilisation_population]

No memos

Répondant : au début, les gens ne voulaient pas venir dans les structures parce qu’on pensait que là où il y a gratuité c’est là où il y a Ebola mais à forcer de sensibiliser les gens, c’est en ce moment-là que les gens ont compris que ça c’est pour nous, c’est pour venir nous aider parce qu’on pouvait rester à la maison tant bien qu’on est malade parce qu’on a peur d’aller là où il y a Ebola. Je me souviens que même au début notre projet avait des problèmes, quand vous venez dire que gratuité des soins, on va dire voilà Ebola vient d’arriver là-bas. Désormais on change, on va aller même payer les soins ailleurs pourvu qu’on ne soit pas victime d’être pris au CT où on a été dépisté ou on a Ebola ou ceci ou cela. Et vous allez voir qu’en un certain temps les structures privées gagnées beaucoup des malades par rapport là où il y avait de la gratuité parce qu’ils avaient peur que quand ils seront là on va les dépister et quand on va les dépister on appelle l’ambulance pour aller se retrouver au centre de traitement Ebola et au début, c’était très compliqué même quelqu’un vous appelait l’ambulance parce que son enfant a une fièvre, c’est déjà un conflit que vous créez dans la communauté. Ce n’est qu’après la sensibilisation que les gens ont commencé à comprendre qu’il faut toujours aller et d’ailleurs pour appuyer ça nous sommes allés trop loin car nous avons commencé à faire des centres de traitement transit plus proche dans la communauté parce que les gens disent bon, je suis par exemple dans le fin fonds, très loin, vous amenez de Uwitsha pour aller me faire soigner à Beni et il n’y a même pas un membre de ma famille qui viendra me voir là-bas donc il y a une grande distance donc autant mieux ne pas aller là-bas même si vous dites qu’il y a la gratuité des soins, laissez-moi mourir à la maison. Donc on a compris qu’il faut alors chercher à rapprocher ce traitement près de la population pour essayer de résoudre ce qu’il présentait comme difficulté

**P26: IA_01_ONG-PTF_SUP.rtf - 26:8 [Répondant : au début la présen..] (16:16)	(Super)**

Codes:	[Triage-Isolement-Chlore-thermoflash_Réaction_population]

No memos

Répondant : au début la présence des signes signifiait que vous venez d’implanter Ebola là-bas, il suffisait seulement de voir le dispositif de lavage avec une zone d’isolation de triage, on disait qu’Ebola est déjà là et là d’office les gens ne pouvaient plus venir et même quelqu’un comme on le dit lavage avec l’eau chlorée, ils vont dire que cette odeur c’est ça Ebola et alors on était obligé de mettre un peu d’eau avec du savon et l’eau chlorée pour que celui qui ne pouvait pas accepter de se laver les mains aves l’eau chlorée avec qu’il sait qu’il y a Ebola alors on va contourner cela et on lui dit qu’il y a l’eau avec du savon là-bas comme tu ne vaux pas l’eau chlorée parce que c’est ça Ebola, il y a alors l’eau au savon ici, curieusement vous allez voir quelqu’un laisse l’eau chlorée pour aller se laver là où il y a l’eau avec le savon

**P26: IA_01_ONG-PTF_SUP.rtf - 26:9 [Répondant : oui au début ça po..] (18:18)	(Super)**

Codes:	[Triage-Isolement-Chlore-thermoflash_Réaction_population]

No memos

Répondant : oui au début ça pouvait faire peur parce que c’est humain parce que toute personne qui est entrée là-bas, on pouvait être en forme mais après deux jours on va sortir un cadavre et alors donc entrer au CTE signifie mourir. C’était ça une conclusion active pour la communauté. Il suffit qu’on entre au CTE, on ne va plus sortir dès là, ça sera la mort d’office donc je dirais que le taux de guérison était vraiment très faible alors la communauté avait compris que si vous entrez là-bas, c’est votre fin.

**P26: IA_01_ONG-PTF_SUP.rtf - 26:10 [Répondant : A part les privés,..] (20:20)	(Super)**

Codes:	[Structure-Service_Utilisation_population]

No memos

Répondant : A part les privés, on peut se retrouver chez les tradipraticiens, dans des maisons de prière où il y a même certains pasteurs qui se sont enfermés avec les malades, curieusement le pasteur va mourir et le malade aussi va mourir et part après les gens vont comprendre que le truc existe et plus le taux de guérisons augmentait, les gens ont commencé à avoir de l’espoir, et même si quelqu’un en a, il y a aussi des vainqueurs qui commencent à sortir et on pouvait faire des sensibilisations avec des gens qui sont des vainqueurs pour persuader les gens que là-bas on nous prend en charge donc il y a une bonne prise en charge

**P26: IA_01_ONG-PTF_SUP.rtf - 26:11 [Répondant : oui comme je disai..] (22:22)	(Super)**

Codes:	[Ressources_humaines_Disponibilité et perturbations]

No memos

Répondant : oui comme je disais tantôt avant l’épidémie, les gens étaient habitués à des petites primes curieusement dans l’épidémie, on a trouvé que les primes de gens ont été triple si combien de fois je ne sais pas, curieusement beaucoup de nos structures ont été vidés et les gens qui pouvaient aller à la riposte parce que là il y avait une bonne cagnotte parce que c’était idéal. Ou ça pouvait perturber parce qu’une structure qui n’avait pas d’appuie pouvait être vide de son staff parce que les gens étaient partis à la riposte.

**P26: IA_01_ONG-PTF_SUP.rtf - 26:12 [Répondant : la complétude, on ..] (26:26)	(Super)**

Codes:	[SNIS_fonctionnement_Decrire_pdt MVE]

No memos

Répondant : la complétude, on pouvait voir des registres qui ont été vides parce qu’on n’avait pas assez de temps de concentration et beaucoup sollicité alors vous allez prendre le registre qui est presque incomplet, le canevas des rapport qui manque certaines données qu’on n’a pas eu le temps de faire des suivis pour avoir ces données. Nous avons instauré notre système où on a mis un agent commis pour le data parce que nous avons trouvé que si on ne fait pas ça nous aurons de problème sérieux alors autant mieux mettre un staff qu’on responsabilise et maintenant que nous l’appuyons et quand nous serons sorti, est-ce qu’on va encore l’appuyer, c’est ça la crainte. On le fait parce qu’on a besoin des données et pour nous les données, c’est ce qui est capital alors on se dit parmi tout le staff, on sait que l’IT est trop sollicité et vous-là on vous prend comme point focal sur le plan des données et on lui donne des stratégies comme récolter les données et faire le reportage mais alors quand on sera parti peut-être la petite prime qu’on lui donne pour qu’il soit motivé, la crainte est que quand il ne sera plus motivé, est-ce qu’il va continuer mais nous les problèmes est que nous puissions avoir le données et les mêmes données seront transmises au bureau central, c’est pourquoi on demande son accompagnement dans tout ce qu’on fait. Et quand nous allons partir est-ce que vous allez fidéliser et pérenniser ces innovations ou bien ça va partir avec les partenaires, ça sera compliqué.

**P26: IA_01_ONG-PTF_SUP.rtf - 26:13 [Répondant : oui, je dirais que..] (24:24)	(Super)**

Codes:	[SNIS_fonctionnement_Decrire_pdt MVE]

No memos

Répondant : oui, je dirais que c’est une période qui a été très complexe pour le rapportage, on pouvait voir même certaines activités qui n’étaient même plus rapportées par exemple les activités vaccinales, il n’y avait plus des données même vous pouvez arriver à la structure et vous voulez aller voir l’infirmier titulaire, on dira qu’il n’est pas disponible car il est avec la commission de surveillance quelque part parce qu’il y a eu un cas suspect. Vous pouvez aller voir quelqu’un, on vous dit qu’il est avec les équipes de la riposte et nous qui restons pendant longtemps dans la structure et vous voulez peut-être faire des remarques mais il y a le téléphone qui sonne, oh IT venait d’urgence, il y a un cas suspect, il vous laisse là-bas donc pour nous comme nous avons l’habitude de rester presqu’un mois dans la structure, on pouvait se débrouiller avec les staff qui sont là. Sur le plan rapportage, il y a eu beaucoup des lacunes, beaucoup vraiment

**P26: IA_01_ONG-PTF_SUP.rtf - 26:14 [Répondant : les supervisions e..] (28:28)	(Super)**

Codes:	[Supervision_pdt-épidémie-Ebola]

No memos

Répondant : les supervisions et les monitorages, ça c’est encore une bête noire d’ailleurs c’est le défi que nous sommes en train de changer avec la DPS et même avec le bailleur. Nous avons eu l’appui de la DPS, nous avons dans nos équipes deux types d’évaluations par trimestre avec l’appui de la DPS et là la DPS vient et on fait la supervision conjointe avec la DPS et au niveau des zones de santé et des BCZ et comme ils sont trop sollicités, c’est là où il y a des problèmes. Vous pouvez établir le calendrier des supervisions conjointes mais vous serez là et quand vous voulez planifier l’activité et il est pris par autre activité. Au non, il y a eu un cas suspect quelque part et il faut faire des investigations donc c’est très compliqué mais il y a des réunions d’analyse des données chaque mois, à notre niveau et au niveau de la BCZ et en ce moment-là, on essaye de voir qu’est-ce qu’on a trouvé et est-ce que ça marche et on se met d’accord pour les données. Ça c’est sur le plan de monitoring et évaluation des données mais supervision conjointe, certaines zones se disponibilisent mais certaines, non

**P26: IA_01_ONG-PTF_SUP.rtf - 26:15 [Répondant : oui, parce que cel..] (30:30)	(Super)**

Codes:	[Supervision_pdt-épidémie-Ebola]

No memos

Répondant : oui, parce que celles qui sont disponibles parce que par rapport à leur supervision, nous nous accompagnons les structures, nous sommes là pour un temps et quand nous serons partis, ce que nous avons relevé comme défi, c’est supposé que l’équipe cadre de la zone de santé va continuer à accompagner ces structures et celles qui sont disponibles pour nous accompagner sont en train de s’approprier les activités à tel enseigne que quand ils ont des difficultés ils sont d’ailleurs les premiers à déceler les problèmes et nous faire participer mais ceux qui s’en fous, ceux nous qui venons vers elles pour leur montrer ceux que nous avons trouvé comme problème et vous sentez que les gens s’intéressent moins, il s’intéressent la riposte parce que ça paient mieux

**P26: IA_01_ONG-PTF_SUP.rtf - 26:16 [Répondant : de manière général..] (32:32)	(Super)**

Codes:	[Gratuité_Accueil_population]

No memos

Répondant : de manière générale, c’est ce que je disais pour nous quand Ebola est arrivé nous nous avions déjà le projet donc pour nous quand on arrive et nous sommes acceptés, je donne l’exemple de deux structures que nous appuyions et quand nous sommes sortis pour aller dans d’autres, jusqu’aujourd’hui il y a des lettres et des lettres de communautés qui ne sont pas encore en mesure de se prendre en charge en ce qui concerne les soins. C’est pour dire que la gratuité est bien reçue dans la communauté.

**P26: IA_01_ONG-PTF_SUP.rtf - 26:17 [Répondant : oui, c’est ce que ..] (34:34)	(Super)**

Codes:	[Souhait_Changement_gestion_futures_épidémies]

No memos

Répondant : oui, c’est ce que je disais, il faut d’abord faire participer la population tout au début que d’importe une main d’œuvre. Il faudrait que cette main d’œuvre importe vienne en appuie mais dans la communauté comme ça quand vous importez une main d’œuvre, ça créée toujours des résistances ; à tel enseigne que les gens vont dire pourquoi vous nous amenez les gens de très loin alors que nous avons nos enfants qui ont de gros diplômes et actuellement ils sont en train de nous aider et que nous comprenions mieux que ces gens qui nous parler lingala que nous ne comprenions pas, ça dépend de milieux, nous qui sommes dans les fins fonds on prend le lingala comme la langue des gens mais quand il y a quelqu’un qui me perle dans ma langue là je le comprends mieux que l’anglais ou le français qui demande encore un interprète.

**P26: IA_01_ONG-PTF_SUP.rtf - 26:18 [Répondant : dans les villages,..] (36:36)	(Super)**

Codes:	[Souhait_Changement_gestion_futures_épidémies]

No memos

Répondant : dans les villages, quand on te voit parler le lingala, on dit que c’est la langue des militaires donc ce sont les gens qui ne sont pas presque les autres. Alors ça, ça pouvaient être un défi. Vous m’amenez les gens qui parlent anglais et il me faut un interprète pour penser que ce qu’il est en train de dire, est-ce que c’est ça qu’il dit textuellement donc il faut s’appuyer sur le gens recrutent localement et c’est la stratégie qu’on a adopté vers la fin et on a fait des bons résultats.

**P26: IA_01_ONG-PTF_SUP.rtf - 26:19 [Répondant : il y avait aussi q..] (38:38)	(Super)**

Codes:	[Souhait_Changement_gestion_futures_épidémies]

No memos

Répondant : il y avait aussi question de, je ne sais pas si c’est notre ministère ou un autre et comme il y a eu plusieurs intervenants, il y avait des équipes qui pouvaient être rémunérées comme il se doit et les autres qui jusqu’à présent on des arriérés et vous voyez ce que ça fait. Je suis à la ligne d’attaque mais j’ai trois mois d’arriéré, je ne sais pas mon comportement face aux malade et cela n’est pas facile. Et quelqu’un d’autre peut avoir 5$ par jour, l’autre a 250$ par jour, vous voyez encore le décalage mais je ne sais pas ça c’est leur termes de référence alors nous on n’a pas eu trop des problèmes parce que nous étions déjà dans la communauté et en plus de cela dans les activités de la riposte, il ne faut pas seulement s’appuyer dans les activités de la riposte mais il faut penser à d’autres petits projets de développement, c’est qui fait que la communauté puisse accepter vos activités dans la communauté. Mais quand vous venez seulement porter vos masques, mais la population veut entendre après la riposte qu’est-ce qui va rester comme nous, je disais on fait des sources et des infrastructures, ils savent que ce gens avant la riposte, ils ont fait une source quelque part, avant la riposte, ces gens nous ont fait la maternité quelque part donc à côté de tout ce que nous sommes en train de faire comme d’habitude mais attention maintenant il y a corona et là la population va vite m’accepter parce qu’elle connait qu’à part cette corona-là, lui m’a déjà construit une maternité et ma femme est en train d’accoucher là-bas, nos enfants sont en train de puiser de l’eau quelque part là-bas donc il faut des projets de développement à part le projet d’urgence dans la riposte.

**P26: IA_01_ONG-PTF_SUP.rtf - 26:20 [Répondant : c’est à cause de l..] (44:44)	(Super)**

Codes:	[Agression-Menaces_Structure-Prestataire_raisons]

No memos

Répondant : c’est à cause de la stratégie et je veux d’ailleurs vous dire que plusieurs partenaires sont venus nous côtoyer, comment vous entrez même dans les endroits que nous ne pouvons pas entrer, beaucoup des partenaires sont venus vers nous comme ça dans les commissions et même là où on pouvait sentir qu’il y a des résistances et la commission pouvait dire que venez car vous êtes les mieux écouter, pouvez-vous nous aider à aller vers cette communauté donc ça c’est vraiment une bonne stratégie.

**P26: IA_01_ONG-PTF_SUP.rtf - 26:21 [Répondant : non,] (42:42)	(Super)**

Codes:	[Agression-Menaces_Structure-Prestataire_raisons]

No memos

Répondant : non,

**P26: IA_01_ONG-PTF_SUP.rtf - 26:22 [Répondant : non, pas même un s..] (40:40)	(Super)**

Codes:	[Agression-Menaces_Structure-Prestataire_raisons]

No memos

Répondant : non, pas même un seul

**P26: IA_01_ONG-PTF_SUP.rtf - 26:23 [Répondant : merci pour ce temp..] (46:47)	(Super)**

Codes:	[Commentaire-Recommandation_Répondant]

No memos

Répondant : merci pour ce temps d’écoute et de partage, j’ose croire que ça ne va pas rester une lettre morte. Peut-être certaines recommandations vous pouvez les véhiculer et comme nous n’avons pas la culture de lire, j’ose croire que cela pouvez aider à faire quelques choses dans les jours à venir pour les futurs cadres de notre pays même au niveau mondial.

**P27: IA_02_AS_IT_03.rtf - 27:1 [Répondant : Je suis infirmier ..] (8:8)	(Super)**

Codes:	[Répondant_Responsabilité_habituelles]

No memos

**Répondant** : Je suis infirmier titulaire de l’aire de santé butchumu, j’ai 8 ans ici en qualité IT mais je suis venu à une autre aire de santé

**P27: IA_02_AS_IT_03.rtf - 27:2 [Répondant : Le travail d’un IT..] (10:10)	(Super)**

Codes:	[Répondant_Responsabilité_habituelles]

No memos

**Répondant** : Le travail d’un IT normalement c’est faire les consultations de malades, organisation des services au sein de l’aire de santé et puis superviser les activités et rédiger le rapport mensuel il est interprète entre la communauté et le BCZS

**P27: IA_02_AS_IT_03.rtf - 27:3 [Répondant : Bon l’apparition d..] (12:12)	(Super)**

Codes:	[Début-Progression_Epidémie MVE]

No memos

**Répondant** : Bon l’apparition de l’Ebola normalement à commencer à mangina au mois d’Aout 2018 lorsqu’il y a eu de cas, le 1^er^ est apparu au centre de santé c’était le 4 septembre 2018. Le malade est arrivé ici, il présentait les signes semblables au paludisme et la fièvre typhoïde. Après on l’avait observé après trois jours. Trois jours après, il n’y avait pas de changement le 4 jour il a présenté le signe de l’asthénie et des vomissements et après on a jugé par ce que la fièvre résistait nous avons mieux jugé de l’envoyer au clinique universitaire, quand on lui a envoyé au clinique universitaire à 00h il est mort. C’est ça le 1^er^ cas. Il y a une personne qui avait perdu son enfant a mangina, il était parti au deuil après il est retourné à Beni, lorsqu’il s’est senti mal il est venu ici et nous a dit qu’ils étaient dans une circonstance de deuil pour un cas confirmer de l’Ebola. Nous les prestataires nous avons consulté le malade avec beaucoup de précaution mais celui qui l’avait reçu et l’autre qui avait monté garde nuit ces deux étaient atteints et ils sont parti à la CTE, Dieu merci ils ont été vainqueurs et ils travaillent.

**P27: IA_02_AS_IT_03.rtf - 27:4 [Répondant : Ok tout le mois de..] (14:15)	(Super)**

Codes:	[Répondant_Responsabilité_pdt MVE]

No memos

**Répondant** : Ok tout le mois de septembre à partir du 6 le centre de santé était fermé, septembre, octobre le centre de santé était fermé selon les mesures venues de la hiérarchie.

Je suis reste seul dans le centre de santé, je fais le suivi de contact et puis j’ai été répondant à toute question provenant de chef de la riposte

**P27: IA_02_AS_IT_03.rtf - 27:5 [Répondant : Butembo ville par ..] (17:17)	(Super)**

Codes:	[Début-Progression_Epidémie MVE]

No memos

**Répondant** : Butembo ville par ce que 1^er^ cas dans butembo ville c’est ici au centre de santé Butsundo.

**P27: IA_02_AS_IT_03.rtf - 27:6 [Répondant : Une autre extensio..] (19:19)	(Super)**

Codes:	[Début-Progression_Epidémie MVE]

No memos

**Répondant** : Une autre extension ou un autre cas est venu de Beni après il est entré dans la zone de santé de Katwa précisément l’aire de santé mutsanga et c’est là où provient l’extension.

**P27: IA_02_AS_IT_03.rtf - 27:7 [Répondant : Dans l’aire de san..] (21:21)	(Super)**

Codes:	[Début-Progression_Epidémie MVE]

No memos

**Répondant** : Dans l’aire de santé le 1^er^ cas il y avait un petit fils qui qui était garde de cette grand-mère et le petit fils à présenter des signes mais il a caché ça pour une période après le décès de sa grand-mère puis on l’avait trouvé a une poste de santé bora uzima d’où est venu le 2ième cas. au poste de santé bora uzima on lui a observer pendant 5 jours mais Dieu merci lorsque je faisais des visites dans la communauté je croisais deux garçons qui disaient que ils viennent du centre de santé pour rendre visite leur ami qui est hospitaliser là-bas mais son état de santé ne pas bonne , puis j’ai eu l’esprit de partir au centre de santé et j’ai posé une question au responsable du poste de santé et on a pris le dossier du malade et je trouvais tous les signe qui sont préétablis au cas suspect d’Ebola . J’ai appelé l’équipe et j’ai alerté l’équipe est venue pour faire l’investigation et l’équipe à transférer le malade au CTE et au niveau de CTE ils ont confirmé et le cas est devenu positif, après 2 à 3 jours par ce qu’il est arrivait au poste de santé 4 jours après qu’il est malade puis il a fait encore 4jours au poste de santé mais au CTE il n’a pas fait plus de 48 heures il est décédé, à partir de ces deux cas là.

**P27: IA_02_AS_IT_03.rtf - 27:8 [Répondant : Pour parler d’Ebol..] (23:23)	(Super)**

Codes:	[Considération_Ebola_population]

No memos

**Répondant** : Pour parler d’Ebola la communauté n’a pas compris que c’est une maladie, par ce qu’ils étaient entre de dire que c’est une maladie inventée (iko Rungu)

**P27: IA_02_AS_IT_03.rtf - 27:9 [Répondant : ça veut dire fonde..] (25:25)	(Super)**

Codes:	[Considération_Ebola_population]

No memos

**Répondant** : ça veut dire fonder quelque chose pour avoir un bénéfice, au départ c’est ça et c’est ce qui avait causer une résistance, a hier il y avait de casse mais dans l’aire de santé de Vutsundo il n’y avait pas de casse mais il y avait de rumeur que on va détruire le centre de santé par ce que c’est nous qui avons envoyé le 1^er^ et le deuxième qui était au poste de santé pour partir au CTE. Dieu merci nous avons réuni les jeunes, nous les avons expliqués, nous avons étaient vacciné le 6 aux cliniques universitaires et après la sensibilisation de jeune, ils ont compris que c’est une maladie. Mais la résistance était surtout dans la zone de santé de Katwa et surtout aire de santé wayene et Mutsanga ou il y avait beaucoup de casses mais au niveau de l’aire de santé de Vutsundo la communauté à bien compris

**P27: IA_02_AS_IT_03.rtf - 27:10 [Répondant : Affirmatif par ce ..] (27:27)	(Super)**

Codes:	[Structure-Service_Fermeture_pdt MVE]

No memos

**Répondant** : Affirmatif par ce que lorsque nous avons fermé la structure en septembre et octobre en un moment toutes les activités étaient suspendue : la vaccination, CPN, CPS et l’activité curative donc l’apparition d’Ebola a perturbé les activités et par après nous avons ouvrir les activités aussi ont commencée par ce que les gens ne voulaient plus adhérer aux activités du centre par ce qu’il y avait beaucoup de rumeur et beaucoup de résistance.

**P27: IA_02_AS_IT_03.rtf - 27:11 [Répondant : Autre rumeur, ils ..] (29:29)	(Super)**

Codes:	[Considération_prestataire_population]

No memos

**Répondant** : Autre rumeur, ils disaient que nous sommes déjà corrompus

**P27: IA_02_AS_IT_03.rtf - 27:12 [Répondant : Les prestataires a..] (31:32)	(Super)**

Codes:	[Considération_prestataire_population]

No memos

**Répondant** : Les prestataires avec l’équipe de riposte, ils disaient que nous avons avalé.

Une autre rumeur est celle-ci lorsque l’équipe de riposte est arrivée elle n’a pas associé la base dans les activités, c’est ça une autre résistance.

**P27: IA_02_AS_IT_03.rtf - 27:13 [Répondant : En général la qual..] (34:34)	(Super)**

Codes:	[Qualité_soins_Prestataires]

No memos

**Répondant** : En général la qualité de soins pendant la période d’Ebola, il y avait beaucoup de changement. Par ce qu’il y avait de thème qu’on ne savait pas pendant la période de l’épidémie, après formation on nous a appris beaucoup de chose comme PCI prévention contrôle des infections tout ça on ne savait pas, la tenue de service il n’y avait pas et avec tout ça il y a eu un grand changement et utiliser le gant et faire le lavage de mains nous avons vu que par la qualité de soins ça nous à amener un apport nécessaire. Le PCI était vraiment instauré au niveau de l’aire de santé et tous les prestataires ont subir plusieurs formations et ça nous a aidés. Et au niveau de activités lorsque nous partons vacciné nous portons tous de gant et nous utilisons de seringue à usage unique et tout c’était bien respecté dans la mesure de prévention.

**P27: IA_02_AS_IT_03.rtf - 27:14 [Répondant : Bon se protéger am..] (36:36)	(Super)**

Codes:	[Qualité_soins_Prestataires]

No memos

**Répondant** : Bon se protéger amener la peur aux malades, comment tu peux me toucher et tu portes de gant alors que tu ne portais pas ça avant et comment tu peux porter un masque donc tu croix que je suis une personne qu’on doit isoler, dans la période de soins ça c’était une crainte dans la communauté. Deuxièmement, lorsqu’ils entrent au centre de santé par ce qu’il fallait instaurer le système de triage et par le triage tu commencer à prendre la température s’il l’a la fièvre tu lui donne une chaise et celui qui n’a pas la fièvre il passe et on lui met dans le transit par ce que on n’était pas entre de dire salle d’isolement mais salle de transit pour qu’ils n’aient pas peur. Ça aussi à amener la crainte au niveau de la communauté mais par après ils ont compris, après dans nous avons instauré ce système dans toutes les structures. Même si le triage n’est pas construit là-bas on disait que vous pouvez même isoler un malade sur un banc pour ceux qui ont d’autre maladie.

**P27: IA_02_AS_IT_03.rtf - 27:15 [Répondant : Pour l’accouchemen..] (38:38)	(Super)**

Codes:	[Structures-Services_fonctionnement]

No memos

**Répondant** : Pour l’accouchement en tout cas au centre de santé la maternité n’est pas fréquentée par ce que les femmes enceintes préfèrent partir au centre hospitalier pour ça on ne peut pas parler d’Ebola par ce qu’avant Ebola on avait 2 à 3 accouchements par mois ça on ne peut pas parler. Pour la CPS a fonctionné normalement pendant la période il n’y a pas de changement ni perturbation. Pour le service de tuberculose on fait de dépistage et on soigne le malade pour ça il n’y a pas de problème il y n’a pas de perturbation. Mais du côté VIH on réserve à d’autres structures.

**P27: IA_02_AS_IT_03.rtf - 27:16 [Répondant : Les indicateurs ét..] (40:40)	(Super)**

Codes:	[Structures-Services_fonctionnement]

No memos

**Répondant** : Les indicateurs étaient bien avant Ebola mais dans deux premiers mois après l’ouverture de centre, les indicateurs des activités étaient entre de baissé et dans 4ieme mois les indicateurs sont revenu au normal

**P27: IA_02_AS_IT_03.rtf - 27:17 [Répondant : Nous avons intensi..] (42:42)	(Super)**

Codes:	[Structures-Services_fonctionnement_Solution]

No memos

**Répondant** : Nous avons intensifié la sensibilisation et l’adhésion de la communauté dans des activités de la riposte.

**P27: IA_02_AS_IT_03.rtf - 27:18 [Répondant : Je vérifier la pér..] (44:44)	(Super)**

Codes:	[Gratuité_Evolution]

No memos

**Répondant** : Je vérifier la période par ce que je ne me rappelle plus la date ou la gratuité à commencer, juste 2 minutes. La gratuité à commencer en novembre 2018 ? À était suspendue en février 2019 et aout 2019, en 2020 elle a repris le 15 février jusque maintenant nous sommes dans la gratuité

**P27: IA_02_AS_IT_03.rtf - 27:19 [Répondant : Ok la gratuité qui..] (46:46)	(Super)**

Codes:	[Gratuité_Structures-Services_concernés]

No memos

**Répondant** : Ok la gratuité qui a commencé, d’abord au niveau des structures, ils ont ciblé les centres de santé étatique. Comme à Vutsundo ils ont pris clinique universitaire et le centre de santé, d’autres structures privées n’étaient pas concernées ni impliquées dans la gratuité et la rubrique qui était considérer au centre de santé c’était la consultation curative et la référence. Mais pour le contrat de 2020 il considère seulement au centre de santé la consultation curative. Pour les centres hospitaliers il considère les malades qui étaient hospitalisés en médecine interne et dans la pédiatrie, Les autres activités sont payantes.

**P27: IA_02_AS_IT_03.rtf - 27:20 [Répondant : Pour moi le 1er co..] (48:48)	(Super)**

Codes:	[Gratuité_Conséquences]

No memos

**Répondant** : Pour moi le 1^er^ contrat avait une conséquence positive par le fait que dans cette période on devrait faire les surveillances épidémiologiques et c’était une occasion on voit tout le monde et on a orienté plusieurs malades au CTE en plus il a renforcé la morale de centre de santé lorsqu’ils étaient en train de venir encore ils ont considéré leurs centres par ce qu’avant l’entrée était rejeter. Une autre conséquence avec la gratuité on nous a doté beaucoup de matériels et on nous a donnés de l’argent et on est parvenu à acheter une parcelle du poste de santé communautaire avec une annexe que nous allons utiliser dans l’avenir avec 10 000 dollars.

**P27: IA_02_AS_IT_03.rtf - 27:21 [Répondant : Les agents aussi é..] (50:50)	(Super)**

Codes:	[Gratuité_Conséquences_motivation_prestataire]

No memos

**Répondant** : Les agents aussi étaient bien satisfait par ce que on le payé pour la gratuité là d’avant mais pour cette gratuité de 2020 jusqu’à maintenant on n’a pas encore viré l’argent dans le compte et il y a pas la motivation par ce que depuis janvier jusqu’aujourd’hui les agents ne sont pas motivés.

**P27: IA_02_AS_IT_03.rtf - 27:22 [Répondant : Bon 30% peut refus..] (52:52)	(Super)**

Codes:	[Gratuité_Rumeurs]

No memos

**Répondant** : Bon 30% peut refuser et 70% acceptent par ce que c’est une partie dans toutes les activités mais 70% étaient d’accord.

**P27: IA_02_AS_IT_03.rtf - 27:23 [Répondant : Le 30% disaient qu..] (54:54)	(Super)**

Codes:	[Gratuité_Rumeurs]

No memos

**Répondant** : Le 30% disaient que ce médicament que nous sommes en train de soigner avec ça contient de virus d’Ebola, comme ils refusent le vaccin soit disant c’est un vaccin fabriquer au niveau du Rwanda c’était ça de rumeur mais 70% étaient d’accord.

**P27: IA_02_AS_IT_03.rtf - 27:24 [Répondant : C’est le gouvernem..] (56:56)	(Super)**

Codes:	[Gratuité_Partenaire d'appui]

No memos

**Répondant** : C’est le gouvernement à travers EPFACE ;

**P27: IA_02_AS_IT_03.rtf - 27:25 [Répondant : Bon le 1er contrat..] (58:58)	(Super)**

Codes:	[Gratuité_Défis]

No memos

**Répondant** : Bon le 1^er^ contrat n’a pas eu de problème par ce que on tenait tous les documents compte tenu de la recommandation qu’ils nous avaient donné vraiment il y a pas de problème.

**P27: IA_02_AS_IT_03.rtf - 27:26 [Répondant : Le problème qui ét..] (60:60)	(Super)**

Codes:	[Gratuité_Défis]

No memos

**Répondant** : Le problème qui était là, 1^er^ contrat là où il y avait difficulté ils n’ont pas répondu à l’approvisionnement de médicament par ce que nous avons une réserve de médicament au dépôt comme ils étaient entre de retirer 20% de médicament et jusque-là nous avons 3300 dollars et ils n’ont pas encore honoré la livraison de nos médicaments. C’est ça le point négatif ou point faible

**P27: IA_02_AS_IT_03.rtf - 27:27 [Répondant : Bon ça dépend de l..] (62:62)	(Super)**

Codes:	[Gratuité_Défis]

No memos

**Répondant** : Bon ça dépend de leur gestion par ce que nous avons envoyé nos requêtes sans réponse.

**P27: IA_02_AS_IT_03.rtf - 27:28 [Répondant : La gratuité est al..] (64:64)	(Super)**

Codes:	[Gratuité_Existence_plan_désengagement]

No memos

**Répondant** : La gratuité est allée au 15 février jusque au 30 juin c’est ça la période qu’ils ont annoncé.

**P27: IA_02_AS_IT_03.rtf - 27:29 [Répondant : Bon ils sont entre..] (66:66)	(Super)**

Codes:	[Gratuité_Existence_plan_désengagement]

No memos

**Répondant** : Bon ils sont entre de dire qu’après le 30 juin il y a un problème qui peut entrer pour les soins gratuits comme à musosa une personne adhère et paye 10 dollars par tête au niveau du ménage donc c’est comme un mutuel.

**P27: IA_02_AS_IT_03.rtf - 27:30 [Répondant : On faisait passer ..] (68:68)	(Super)**

Codes:	[Gratuité_Information_population]

No memos

**Répondant** : On faisait passer de message ou information à la radio qu’il y a gratuité dans toutes les structures sanitaires et lorsqu’ils ont interrompu il n’y avait pas de messages. C’est nous qui disions aux malades ou on fait des affiches pour dire que la période qui était garantie était de soit novembre jusque x mois et la population à compris.

**P27: IA_02_AS_IT_03.rtf - 27:31 [Répondant : Ça ne peut pas man..] (70:70)	(Super)**

Codes:	[Gratuité_Information_population]

No memos

**Répondant** : Ça ne peut pas manque mais il n’y a pas eu de problème par ce qu’il était d’abord satisfait de cette gratuité mais lorsqu’ils sont surpris ils avaient une inquiétude et c’est normal.

**P27: IA_02_AS_IT_03.rtf - 27:32 [Répondant : Pendant la période..] (72:72)	(Super)**

Codes:	[Ressources_humaines_Disponibilité et perturbations]

No memos

**Répondant** : Pendant la période d’Ebola le nombreux de prestataire était insuffisant par ce que on a créé d’autres services, il n’y avait pas le PCI alors il fallait affecter au moins deux hygiénistes, il fallait affecter une personne qui s’occupe du triage. On a aussi ajouté deux personne par ce que nous étions 12 et on a ajouté 2 déjà 14 personnes pendant la période d’Ebola.

**P27: IA_02_AS_IT_03.rtf - 27:33 [Répondant : Bon pour les prest..] (74:74)	(Super)**

Codes:	[Ressources_humaines_solution-local_Disponibilité et perturbations]

No memos

**Répondant** : Bon pour les prestataires de centre il y avait deux vainqueurs mais ils sont partis ils étaient dans les activités du centre, ils ont entré dans la riposte automatiquement le BCZ les a remplacées. Et d’autres prestataires qui travaillé dans l’activité de la riposte ils faisaient le suivi de contact c a d le matin avant 7h lorsqu’ils terminent leurs activités il présente le rapport à 8 heures ils continuent avec leur activité normale vraiment ça n’a pas déséquilibré le service.

**P27: IA_02_AS_IT_03.rtf - 27:34 [Répondant : Avant Ebola il y a..] (76:76)	(Super)**

Codes:	[Ressources_humaines_situation_grève]

No memos

**Répondant** : Avant Ebola il y avait une grève au mois de juin pour quoi par ce qu’il n’y avait pas la prime de risque partout dans la ville de Butembo mais pas ici. Dans la ville de Butembo le prime de risque était coupé à plus de 2000 personnes, c’était ça le premier élément avant la riposte et le prime de risque était couper au niveau de la province et on ne s’est pas si ce processus et fait pourquoi donc c’était ça le problème.

**P27: IA_02_AS_IT_03.rtf - 27:35 [Répondant : La conséquence si ..] (78:78)	(Super)**

Codes:	[Ressources_humaines_situation_grève]

No memos

**Répondant** : La conséquence si je vérifie mes canevas le mois de juin il n’y avait pas des activités préventives vaccin CPN tout on avait fermé le centre de santé ça une conséquence par ce que toute maladie qui pourrait nous attaque pendant cette période. Et le mécontentement des agents à décoller ensemble.

**P27: IA_02_AS_IT_03.rtf - 27:36 [Répondant : Pendant la période..] (80:80)	(Super)**

Codes:	[Ressources_humaines_conditions_travail]

No memos

**Répondant** : Pendant la période d’Ebola deux mois sont passé le mois de septembre et octobre lorsqu’on avait fermé le centre de santé. Le personnel n’était pas pris en charge donc il a passé deux mois à la maison sans toucher quelque chose et c ça c’est un mécontentement. Ce pendant lorsqu’ils ont mis la gratuité quand même le personnel était motivé et dans la période d’Ebola tout le monde était satisfait grâce à la gratuité. Mais un autre mécontentement à part Ebola, dans mes prestataires il n’y a qu’un seul qui à la matricule sur 14 personnes seulement une seule personne qui touche l’argent de l’état mais 13 autres sont ne touchent rien lorsque la recette diminue au centre de santé et on ne travaille pas alaise.

**P27: IA_02_AS_IT_03.rtf - 27:37 [Répondant : Bon la disponibili..] (82:82)	(Super)**

Codes:	[Médicaments_disponibilité_fosa]

No memos

**Répondant** : Bon la disponibilité de médicament si ce n’est ne pas sortie là où on envoyait le 20%, ça dépend de la gestion de fournisseur, c’est là où il y a un problème.

**P27: IA_02_AS_IT_03.rtf - 27:38 [Répondant : Bon le système nat..] (84:84)	(Super)**

Codes:	[SNIS_fonctionnement_Decrire_pdt MVE]

No memos

**Répondant** : Bon le système national d’information sanitaire a fonctionné normalement, avant Ebola et dans la période d’Ebola il n’y avait pas de problème qui pouvait empêcher le système de fonctionné par ce que on faisait de rapport régulièrement et de rapport hebdomadaire chaque semaine et tous ces rapports étaient envoyé. Et tous les analyses et revues mensuelles et au niveau de BCZS on était entre de participer.

**P27: IA_02_AS_IT_03.rtf - 27:39 [Répondant : Dans mon aire de s..] (86:86)	(Super)**

Codes:	[SNIS_fonctionnement_Decrire_pdt MVE]

No memos

**Répondant** : Dans mon aire de santé sauf la période de la grève par ce qu’on ne devrait pas envoyer de rapport et après grève nous avons continué à envoyer de rapports.

**P27: IA_02_AS_IT_03.rtf - 27:40 [Répondant : CODESA dans la pér..] (88:88)	(Super)**

Codes:	[Réunion_pdt-épidémie-Ebola]

No memos

**Répondant** : CODESA dans la période d’Ebola par ce qu’eux travaillent bénévolement nous avons vu la priorité est dans le mettre dans les surveillances à base communautaire ils ont travaillé pendant la période d’Ebola et ils gagnés aussi quelque chose et d’orienté les malades, tenir de réunions et certaines décisions qu’ont prenaient fin du mois, début de mois elles étaient réalisé donc le CODESA n’était pas démotivé.

**P27: IA_02_AS_IT_03.rtf - 27:41 [Répondant : Je pense nous avon..] (90:90)	(Super)**

Codes:	[Gouvernance_soins santé]

No memos

**Répondant** : Je pense nous avons répondu à plus de 100 questions au membre de la riposte et les enquêteurs plus de 100 personnes on travailler avec eux on répond avec eux sans problème et ils n’ont pas influencé à l’autorité de l’aire de santé qui est IT. Nous avons travaillé en collaboration et je pense sans se venter ils ont apprécié l’aire de santé, par ce que lorsque Ebola est arrivé s’il était arrivé à une personne qui ne pas bien expérimenter ils n’allaient pas avoir les données comme ils étaient entre de le récolté.

**P27: IA_02_AS_IT_03.rtf - 27:42 [Répondant : Chez nous non par ..] (92:92)	(Super)**

Codes:	[Gouvernance_soins santé]

No memos

**Répondant** : Chez nous non par ce que dès le départ dans de suivi de contact j’avais pris le relais communautaire et le CODESA, c’est eux qui faisaient le suivi de contact. Après nous avons pris le jeune et les intégré dans la surveillance des bases communautaires, dans mon aire de santé il n’y a pas de problème.

**P27: IA_02_AS_IT_03.rtf - 27:43 [Répondant : Bon nous nous avon..] (94:94)	(Super)**

Codes:	[PCI_Comment_maintenir]

No memos

**Répondant** : Bon nous nous avons que cette épidémie d’Ebola peut encore apparaitre, si on néglige les mesures de la PCI encore on peut être victime alors nous nous allons maintenir le niveau de performance de la CPI comme nous le gardons que ça soit pérenne.

**P27: IA_02_AS_IT_03.rtf - 27:44 [Répondant : Bon nous si la gra..] (96:96)	(Super)**

Codes:	[PCI_Comment_maintenir]

No memos

**Répondant** : Bon nous si la gratuité continue nous allons prendre un pourcentage nous achetons nos propres matériels de la PCI les intrants de PCI comme ça on continue à garder ça. Dans le pourcentage de fonctionnement nous allons aussi acheter de gant et les autres matériels de la PCI pour que nous puissions maintenir.

**P27: IA_02_AS_IT_03.rtf - 27:45 [Répondant : D’abord ils que le..] (98:98)	(Super)**

Codes:	[Souhait_Changement_gestion_futures_épidémies]

No memos

**Répondant** : D’abord ils que les gens puissent considérer quelques formations qu’ils ont réussi et le mettre en application qu’ils ne rejettent pas la formation et tout ce qu’ils ont acquérir. Et si Ebola arrive à un autre endroit nous allons aller là-bas pour les appuyés avec les expériences que nous avions acquises.

**P27: IA_02_AS_IT_03.rtf - 27:46 [Répondant : Contenue des rumeu..] (100:100)	(Super)**

Codes:	[Souhait_Changement_gestion_futures_épidémies]

No memos

**Répondant** : Contenue des rumeurs et des résistances nous allons intégrer les locaux, les expatrier peuvent venir et il y a présence de locaux déjà dans des activités pour éviter de résistance et des casses.

**P27: IA_02_AS_IT_03.rtf - 27:47 [Répondant : Le commentaire et ..] (102:102)	(Super)**

Codes:	[Autres_Problèmes observés]

No memos

**Répondant** : Le commentaire et que notre gouvernement doit prendre en charge le prestataire de santé, vous allez trouver une personne qui fait 20 ans sans numéro matricule, c’est notre inquiétude pour tout le jour. Comme moi le prime de risque qu’on me donner dans cette période d’Ebola ils ont freiné mon prime de risque ça c’est mon inquiétude. Pour le prestataire et pour moi-même c’est une inquiétude.

**P27: IA_02_AS_IT_03.rtf - 27:48 [Répondant : Je pense à mon niv..] (104:104)	(Super)**

Codes:	[Stigmatisation_Survivant Ebola]

No memos

**Répondant** : Je pense à mon niveau dans mon aire de santé il n’y a pas de stigmatisation, ils travaillent ensemble il y a pas de discrimination. Par ce que le service de psychologie fait de réunions il est entre d’aider dans de CV, communauté et église il n’y a pas de problème.

**P27: IA_02_AS_IT_03.rtf - 27:49 [Répondant : les mesures hygién..] (106:106)	(Super)**

Codes:	[Changements_depuis_Ebola]

No memos

**Répondant** : les mesures hygiéniques par ce que les gens mangeaient sans se laver les mains, ça vraiment c’est un point fort. Et nous les prestataires porté les tenues de service et laisser nos tenues c’est déjà un élément capital.

**P27: IA_02_AS_IT_03.rtf - 27:50 [Répondant : Sur le plan social..] (108:108)	(Super)**

Codes:	[Changements_depuis_Ebola]

No memos

**Répondant** : Sur le plan social la communauté aussi connait lors de l’épidémie comment se protéger, ils ont attendu de mesure hygiénique et si corona arrive je pense il n’y aura pas de résistance

**P27: IA_02_AS_IT_03.rtf - 27:51 [Répondant : Surtout se saluer ..] (110:110)	(Super)**

Codes:	[Changements_depuis_Ebola]

No memos

**Répondant** : Surtout se saluer c’est une bonne mesure et se laver les mains à tout le temps, ça c’est les deux mesures que la population met en application.

**P28: IA_02_AS_IT_04.rtf - 28:1 [Répondant : Je suis IT de l’ai..] (9:9)	(Super)**

Codes:	[Répondant_Responsabilité_habituelles]

No memos

**Répondant** : Je suis IT de l’aire de santé de Katia, je fais de consultations et planning familial et d’autres travails. Dalleurs pour le moment nous sommes dans SGBV nous sommes entre de faire l’écoute et ce projet nous l’avons déjà nous suivons de gens ici à la structure et nous faisons l’écoute puis on les envoie à une espace sur qui est en cours. Nous faisons encore les activités de CPS, aux soins de santé primaire les données sont descendue suite à l’épidémie, donc le gens du quartier étaient traumatisé beaucoup dans ce traumatisme ici il y avait beaucoup de cas confirmés et beaucoup de décès c’est pourquoi la population est beaucoup stressé. Et il y avait beaucoup de résistance et pour le réussir à la vaccination ça nous a couté beaucoup d’effort et beaucoup de sensibilisation, même jusqu’aujourd’hui la résistance n’est pas encore fini voir même la population continue toujours à nous menace dans la structure. Pour le même problème lorsqu’ils voient de beaucoup de véhicules à la structure, le visiteur. Ils disent qui y a beaucoup d’argent là-bas, Spécialement toi IT c’est toi qui avait accueil ces gens ici, et c’est à cause de toi qu’ils sont venus à la structure vraiment c’est de la peine.

**P28: IA_02_AS_IT_04.rtf - 28:2 [Répondant : 1er cas c’est les ..] (11:11)	(Super)**

Codes:	[Début-Progression_Epidémie MVE]

No memos

**Répondant** : 1^er^ cas c’est les enfants qui étaient dans la zone de santé de Katwa, puis ils sont venus ici suite à la mort de leur grand-mère dans la zone de santé de Katwa. Puis ils sont venus se réfugie ici, ils étaient des enfants d’un policier je crois et directement un enfant est tombé malade après quelque jour dans l’aire de santé. Ils étaient deux enfants mais le 1^er^ lorsqu’il est tombé malade, il est parti à l’hôpital puis directement référence au CTE, après référence un jour après il était mort au CTE. Celui qui était avec lui à la maison directement il était tombé malade, lui aussi après il était parti à la référence mais il avait survécu. Lorsque ces deux cas étaient dépistés, après ont avaient d’autres cas ont avaient un poste de santé privée là aussi il y avait une personne venue de zone endémique puis il est venu ici au poste de santé et il était observer

**P28: IA_02_AS_IT_04.rtf - 28:3 [Répondant : Zone de Beni à Mab..] (13:13)	(Super)**

Codes:	[Début-Progression_Epidémie MVE]

No memos

**Répondant** : Zone de Beni à Mabalako, après il était sortie là-bas en étant malade ils ont pensé que c’est le paludisme il a était observer et on a fait de perfusion. Tous les visiteurs qui étaient venu voir le malade, étaient tous contaminer et dans ce poste de santé on a eu beaucoup de cas et beaucoup de décès et c’est même poste à était celer donc la majorité de cas était sorti là-bas

**P28: IA_02_AS_IT_04.rtf - 28:4 [Répondant : Premièrement le pr..] (15:15)	(Super)**

Codes:	[Considération_Ebola_population]

No memos

**Répondant** : Premièrement le propriétaire de cette structure disait qu’il n’avait pas compris c’est pourquoi il y avait une résistance. Le propriétaire de cette structure pensait que c’est le gens de centre de santé qui les envoient une malédiction par ce qu’ils sont beaucoup fréquentés que le centre de santé, que le gens de poste de santé et de centre de santé en collaboration avec les agents de la riposte pour qu’il puisse déclarer un cas positif. J’avais eu deux postes de santé ou il y avait ces cas donc tous les cas et les nombreux de décès d’ici étaient venu dans ces deux postes de santé.

**P28: IA_02_AS_IT_04.rtf - 28:5 [Répondant : Ils ont pensé que ..] (17:17)	(Super)**

Codes:	[Considération_Ebola_population]

No memos

**Répondant** : Ils ont pensé que nous qui sommes sur les structures étatique nous les envions, mais la maladie n’existe pas.

**P28: IA_02_AS_IT_04.rtf - 28:6 [Répondant : Donc nous mettons ..] (19:19)	(Super)**

Codes:	[Considération_Ebola_population]

No memos

**Répondant** : Donc nous mettons de cas chez eux par ce qu’ils sont trop fréquentés et les agents de la riposte disent que c’est positif.

**P28: IA_02_AS_IT_04.rtf - 28:7 [Répondant : La communauté disa..] (21:21)	(Super)**

Codes:	[Considération_Ebola_population]

No memos

**Répondant** : La communauté disait que c’est un commerce, le mensonge Ebola n’existe pas c’est faux.

**P28: IA_02_AS_IT_04.rtf - 28:8 [Répondant : Dans la communauté..] (23:23)	(Super)**

Codes:	[Changements_depuis_Ebola]

No memos

**Répondant** : Dans la communauté la population est très étonner par ce qu’elle ne s’attendait pas un nombre élevé de décès, puis elle avait compris que c’est une maladie contenue du nombre de décès, elle commençait à comprendre mais pas tout le monde.

**P28: IA_02_AS_IT_04.rtf - 28:9 [Répondant : Moi j’étais da la ..] (25:25)	(Super)**

Codes:	[Répondant_Responsabilité_pdt MVE]

No memos

**Répondant** : Moi j’étais da la recherche active.

**P28: IA_02_AS_IT_04.rtf - 28:10 [Répondant : Donc premièrement ..] (27:27)	(Super)**

Codes:	[Répondant_Responsabilité_pdt MVE]

No memos

**Répondant** : Donc premièrement lorsqu’Ebola avait commencé il y avait des équipes qui ne connaissaient pas le terrain, puis ils avaient dit comme je suis IT que je puisse les orienter, s’il y a un malade dans la communauté, je dois les montrer ce malade et là où il y a un cas de décès on doit partir là-bas par ce que nous avons une équipe pour aller investiguer dans cette axe. Quand ils avaient maitrisé le terrain et la langue, moi je suis retourne à la structure dans la recherche active pour chercher le cas dans la structure. Un cas qui se présenté vous voyez la définition de cas et si ça répond directement à la définition on envoi au CTE.

**P28: IA_02_AS_IT_04.rtf - 28:11 [Répondant : Au niveau de centr..] (29:29)	(Super)**

Codes:	[Structures-Services_fonctionnement]

No memos

**Répondant** : Au niveau de centre de santé il y avait baisse de fréquentation pour la CPS ; ça concerne la pèse des enfants, les gens avaient peur.

**P28: IA_02_AS_IT_04.rtf - 28:12 [Répondant : Ils avaient peur, ..] (31:31)	(Super)**

Codes:	[Structure-Service_Utilisation_population]

No memos

**Répondant** : Ils avaient peur, dalleur c’était un problème par ce que l’épidémie c’est une maladie contagieuse car avant on pèse à travers une culote alors il fallait changer la stratégie pour peser. Par ce que tout le monde n’avait pas l’argent pour payer sa culote alors celui qui n’avait pas la possibilité restait chez lui, en plus de celle-là il y avait introduction d’un autre vaccin dans la période de l’épidémie. Ils se disaient que voilà ont nous amène aussi le vaccin il y avait aussi beaucoup de résistance par ce qu’ils ont pensé que c’est encore le vaccin d’Ebola c’est pourquoi ils avaient peur, et le taux de CPS était toujours inferieur.

**P28: IA_02_AS_IT_04.rtf - 28:13 [Répondant : La maternité comme..] (33:33)	(Super)**

Codes:	[Service_maternité_fonctionnement]

No memos

**Répondant** : La maternité comme elle était avant il n’y avait pas beaucoup de changement.

**P28: IA_02_AS_IT_04.rtf - 28:14 [Répondant : C’était comme ça d..] (35:35)	(Super)**

Codes:	[Traitement_Invasifs_Prestataire_changement]

No memos

**Répondant** : C’était comme ça dans l’aire de santé, les observations, le laboratoire et le test en tout cas on avait suspendu tout ça.

**P28: IA_02_AS_IT_04.rtf - 28:15 [Répondant : C’était une instru..] (37:37)	(Super)**

Codes:	[Instructions_pdt_MVE]

No memos

**Répondant** : C’était une instruction.

**P28: IA_02_AS_IT_04.rtf - 28:16 [Répondant : Oui] (39:39)	(Super)**

Codes:	[Instructions_pdt_MVE]

No memos

**Répondant** : Oui

**P28: IA_02_AS_IT_04.rtf - 28:17 [Répondant : En tout cas lorsqu..] (41:41)	(Super)**

Codes:	[Traitement_Invasifs_Prestataire_changement]

No memos

**Répondant** : En tout cas lorsqu’il y a communication on ne trouve pas de conséquence par ce que nous avons intensifié notre communication. Le malade venait pour tester la tension on lui dit que la maladie est contagieuse et il y a de risque pour utiliser la tension mettre, vraiment il n’y avait pas d’impact.

**P28: IA_02_AS_IT_04.rtf - 28:18 [Répondant : Non on soignait se..] (43:43)	(Super)**

Codes:	[Service_laboratoire_fonctionnement]

No memos

**Répondant** : Non on soignait seulement le suspect, on soignait de signe donc suspect seulement par ce que si vous converser car sa définition ressemble comme celle d’Ebola, donc tu commences à faire le 1^er^ traitement puis tu peux le référé s’il présente la fièvre avec appui on lui référé directement au CTE.

**P28: IA_02_AS_IT_04.rtf - 28:19 [Répondant : Au niveau de la zo..] (45:45)	(Super)**

Codes:	[Traitement_Invasifs_Prestataire_changement]

No memos

**Répondant** : Au niveau de la zone.

**P28: IA_02_AS_IT_04.rtf - 28:20 [Répondant : Je peux vérifier p..] (47:47)	(Super)**

Codes:	[Instructions_pdt_MVE]

No memos

**Répondant** : Je peux vérifier par ce que j’ai déjà oublié.

**P28: IA_02_AS_IT_04.rtf - 28:21 [Répondant : C’est par le docum..] (49:49)	(Super)**

Codes:	[Instructions_pdt_MVE]

No memos

**Répondant** : C’est par le document.

**P28: IA_02_AS_IT_04.rtf - 28:22 [Répondant : Non] (51:51)	(Super)**

Codes:	[Structure-Service_Fermeture_pdt MVE]

No memos

**Répondant** : Non

**P28: IA_02_AS_IT_04.rtf - 28:23 [Répondant : Non, seulement les..] (53:53)	(Super)**

Codes:	[Structure-Service_Fermeture_pdt MVE]

No memos

**Répondant** : Non, seulement les deux structures par ce qu’il y avait de nosocomiale, beaucoup de cas provenaient de la et c’est à cause de ça qu’on avait fermé mais les autres sont fonctionnels.

**P28: IA_02_AS_IT_04.rtf - 28:24 [Répondant : Oui ils étaient en..] (55:55)	(Super)**

Codes:	[Structure-Service_Utilisation_population]

No memos

**Répondant** : Oui ils étaient entre de venir.

**P28: IA_02_AS_IT_04.rtf - 28:25 [Répondant : Oui dalleurs ils o..] (57:57)	(Super)**

Codes:	[Structure-Service_Utilisation_population]

No memos

**Répondant** : Oui dalleurs ils ont augmenté à cause de la gratuité.

**P28: IA_02_AS_IT_04.rtf - 28:26 [Répondant : Je pense l’épidémi..] (59:59)	(Super)**

Codes:	[Gratuité_Début]

No memos

**Répondant** : Je pense l’épidémie est venue au même moment avec la gratuité, seulement dans les interruptions de message de la gratuité il y a directement une interruption et les malades ne viennent plus. Mais ils sont habitués à la gratuité mais dans cette interruption en tout cas il y a une grande différence.

**P28: IA_02_AS_IT_04.rtf - 28:27 [Répondant : La qualité par ce ..] (61:61)	(Super)**

Codes:	[Qualité_soins_Prestataires]

No memos

**Répondant** : La qualité par ce que on nous a donné beaucoup de critères par rapport à la communauté, pour instruire la communauté par ce que d’une part nous au centres de santé, ont nous avaient donné une recommandation pour les lettres de référence sur le centre hospitalier et ça déjà celui qui ne savait pas le centre de santé il avait si ça à cette période. Donc les normes qu’ils avaient amenées avait fait à ce qui est beaucoup de fréquentation aux aires de santé et aux centres de santé et au avait trouvé beaucoup des gens, ce qui ne voulait pas aller au CH par ce qu’il y avait de critère pour allait là-bas il faut saisir et pour être soigner aux structures. On avait vu que cette stratégie nous avait aidé et les gens avaient compris qu’un centre de santé c’est aussi un hôpital.

**P28: IA_02_AS_IT_04.rtf - 28:28 [Répondant : Au début par ce qu..] (63:63)	(Super)**

Codes:	[Prestataire_Pratique de Prévention contre MVE]

No memos

**Répondant** : Au début par ce que on était doté des épis mais comme c’était étrange à la communauté, lorsqu’ils nous voyaient porté des épis les gens disaient que c’est terrible. Juste au début ils étaient effrayés de nous voir en tenue d’ailleurs pour le triage c’était grave mais aujourd’hui ils sont déjà habitués et ils trouvent ça normal.

**P28: IA_02_AS_IT_04.rtf - 28:29 [Répondant : Nous pour le triag..] (65:66)	(Super)**

Codes:	[Triage_comportement_patient]

No memos

**Répondant** : Nous pour le triage on était le 1^er^ par ce que c’est ici ou on avait commencé à construire le 1^er^ triage, la communication était très efficace par ce que on savait que la salle qu’il vient de construire ici et la nôtre et ça va rester ici par ce que c’est nous qui va l’utilisé.

Nous vraiment pour le triage on n’avait pas de problème, seulement là où on avait eu de problème c’est pour la salle de réunion par ce que lorsqu’ils sont venu construire la comme la communauté à penser que c’est le CTE c’est là où il y avait un problème et on avait trouvé peu d’incivique.

**P28: IA_02_AS_IT_04.rtf - 28:30 [Répondant : Chez eux ils ont p..] (68:68)	(Super)**

Codes:	[Considération_CTE_population]

No memos

**Répondant** : Chez eux ils ont pensé que le CTE c’est un mouroir.

**P28: IA_02_AS_IT_04.rtf - 28:31 [Répondant : Par ce que au débu..] (70:70)	(Super)**

Codes:	[Considération_CTE_population]

No memos

**Répondant** : Par ce que au début il y avait beaucoup de décès et les gens partaient là-bas tardivement c’est pourquoi il y avait beaucoup de décès, aujourd’hui 5 morts demain 3 alors ils avaient eu peur au début.

**P28: IA_02_AS_IT_04.rtf - 28:32 [Répondant : Dans cette période..] (73:73)	(Super)**

Codes:	[Organisation_travail-Circuit des malades_pendant MVE]

No memos

**Répondant** : Dans cette période que nous avons vécu au centre de santé il n’y avait pas les soins qui était avant par ce que nous on avait dit à la consultation au début on avait pris presque tous les services. Et on n’a pas beaucoup de service, nous avons le service curatif, CPN, accouchement, au début on avait pris donc on ne pourrait pas bénéficier même un service. On bénéficie seulement aux services de partenaires qui nous prenaient en charge et c’est ce que on trouve, lorsque ils étaient entre de venir ils diminuaient les activités et dire on va vous payer ce ci et ce qu’ils étaient entre de nous enlever, les malades avaient aussi difficulté de nous payer par ce que ils étaient déjà habitué d’arriver là-bas et jusque aujourd’hui nous avons ce problème par ce que ils ont déjà interrompu beaucoup d’activités comme la CPN, la CPS, fiche et accouchement par ce que il fallait que le malade paye mais ils ne payes pas par ce que les malades disent que c’est un centre de l’état ils ne peuvent pas payés par ce que tout est gratuit, le médicament est gratuit pourquoi partir avec l’argent.

**P28: IA_02_AS_IT_04.rtf - 28:33 [Répondant : Non nous on travai..] (75:75)	(Super)**

Codes:	[Ressources_humaines_Disponibilité et perturbations]

No memos

**Répondant** : Non nous on travaillait et on était parti nulle part.

**P28: IA_02_AS_IT_04.rtf - 28:34 [Répondant : Oui la grève de mé..] (77:77)	(Super)**

Codes:	[Ressources_humaines_situation_grève]

No memos

**Répondant** : Oui la grève de médecins.

**P28: IA_02_AS_IT_04.rtf - 28:35 [Répondant : Un mois.] (79:79)	(Super)**

Codes:	[Ressources_humaines_situation_grève]

No memos

**Répondant** : Un mois.

**P28: IA_02_AS_IT_04.rtf - 28:36 [Répondant : Pour toutes les do..] (81:81)	(Super)**

Codes:	[Ressources_humaines_situation_grève]

No memos

**Répondant** : Pour toutes les données de CPS donc par ce que on n’avait pas vacciné pendant ce mois, pèse, vaccination donc tout le PV dans ce mois c’était en juin avant l’épidémie oui c’était avant l’épidémie.

**P28: IA_02_AS_IT_04.rtf - 28:37 [Répondant : On facturait les s..] (83:83)	(Super)**

Codes:	[Recouvrement_coûts-des-soins_avant gratuité]

No memos

**Répondant** : On facturait les soins.

**P28: IA_02_AS_IT_04.rtf - 28:38 [Répondant : Nous on avait une ..] (85:85)	(Super)**

Codes:	[Recouvrement_coûts-des-soins_avant gratuité]

No memos

**Répondant** : Nous on avait une tarification forfaitaire.

**P28: IA_02_AS_IT_04.rtf - 28:39 [Répondant : Donc cette tarific..] (87:87)	(Super)**

Codes:	[Recouvrement_coûts-des-soins_avant gratuité]

No memos

**Répondant** : Donc cette tarification forfaitaire était venue dans l’épidémie mais avant on facturait de service comme CPN si c’est 1 dollars.

**P28: IA_02_AS_IT_04.rtf - 28:40 [Répondant : Il n’y a pas à pay..] (89:89)	(Super)**

Codes:	[Recouvrement_coûts-des-soins_avant gratuité]

No memos

**Répondant** : Il n’y a pas à payer, mais il met sa tarification forfaitaire.

**P28: IA_02_AS_IT_04.rtf - 28:41 [Répondant : Et aujourd’hui c’e..] (91:91)	(Super)**

Codes:	[Recouvrement_coûts-des-soins_actuel]

No memos

**Répondant** : Et aujourd’hui c’est comme ça, il y a certaines activités qu’elle prend en charge et d’autres activités normalement on doit la facture mais les gens ne payent pas.

**P28: IA_02_AS_IT_04.rtf - 28:42 [Répondant : Ils disent qu’ils ..] (93:93)	(Super)**

Codes:	[Recouvrement_coûts-des-soins_actuel]

No memos

**Répondant** : Ils disent qu’ils n’ont pas le moyen.

**P28: IA_02_AS_IT_04.rtf - 28:43 [Répondant : Tu peux lui factur..] (95:95)	(Super)**

Codes:	[Recouvrement_coûts-des-soins_actuel]

No memos

**Répondant** : Tu peux lui facturer 2.5 dollars il te dit qu’il n’a pas l’argent.

**P28: IA_02_AS_IT_04.rtf - 28:44 [Répondant : Quand même la grat..] (97:97)	(Super)**

Codes:	[Gratuité_Conséquences]

No memos

**Répondant** : Quand même la gratuité dans notre structure ça nous a aidé par ce que on trouve quelque chose, il y a un pourcentage de médicament et si on a beaucoup de malades ça nous aide nous le personnel et le fonctionnement, nous avons vu que ça nous a déjà aidé par rapport à la période d’avant la gratuité.

**P28: IA_02_AS_IT_04.rtf - 28:45 [Répondant : Le médicament donc..] (99:99)	(Super)**

Codes:	[Gratuité_Structures-Services_concernés]

No memos

**Répondant** : Le médicament donc la consultation et le médicament.

**P28: IA_02_AS_IT_04.rtf - 28:46 [Répondant : Ils doivent payer...] (101:101)	(Super)**

Codes:	[Gratuité_Structures-Services_concernés]

No memos

**Répondant** : Ils doivent payer.

**P28: IA_02_AS_IT_04.rtf - 28:47 [Répondant : A la fin, c’est le..] (103:103)	(Super)**

Codes:	[Gratuité_Conséquences_motivation_prestataire]

No memos

**Répondant** : A la fin, c’est le partenaire qui paye au compte de malades.

**P28: IA_02_AS_IT_04.rtf - 28:48 [Répondant : Oui] (105:105)	(Super)**

Codes:	[Gratuité_Conséquences_motivation_prestataire]

No memos

**Répondant** : Oui

**P28: IA_02_AS_IT_04.rtf - 28:49 [Répondant : ça n’a pas de cons..] (107:107)	(Super)**

Codes:	[Gratuité_Conséquences]

No memos

**Répondant** : ça n’a pas de conséquence nous avons déjà trouvé un travail et si le travail augmente c’est bien pour nous quand nous avons les malades par ce que notre structure est fréquenter.

**P28: IA_02_AS_IT_04.rtf - 28:50 [Répondant : Ils ont augmenté e..] (109:109)	(Super)**

Codes:	[Gratuité_Conséquences_utilisation_services]

No memos

**Répondant** : Ils ont augmenté et nous on se forçait pour partager quelque attribution et quelque activité donc on décentralise.

**P28: IA_02_AS_IT_04.rtf - 28:51 [Répondant : Avant l’épidémie o..] (111:111)	(Super)**

Codes:	[Médicaments_disponibilité_fosa]

No memos

**Répondant** : Avant l’épidémie on n’avait pas beaucoup de médicament et les malades aussi n’étaient pas nombreux, on avait le médicament qui mérité la structure. Avec l’épidémie les nombreux de malades à augmenter et au début de l’épidémie on nous avait donné le médicament Fond Mondial, ça on avait doté les aires de santé après quand le partenaire sont venu dalleurs, l’épidémie était venu et nous avons les partenaires ils nous ont beaucoup aidé en nous donnant beaucoup de médicament en plus ces partenaires nous à aider avec cet argent ou il y avait 30% de médicament dans ce montant il faut qu’on nous amène , ils ont commencé à avoir de retenir d’argent directement au niveau de la base, comme ils ont le retenu de l’argent au début ils nous avaient amené le médicament qui ne correspond pas de commande après ça je crois jusqu’aujourd’hui si ils avaient donné un lot dans l’épidémie et notre argent est là. De retenu à l’ASRAMES puis ils avaient dit que c’est ça le partenaire qui va nous donner le médicament jusqu’aujourd’hui le médicament ne pas encore venu et on fait souvent de commande, et il y a une retenu d’argent de 20% pour chaque mois donc le retenu d’argent et le médicament aussi n’est pas encore là. Puis on fait tout le moyen puis on a dit pour cet argent vous allez faire une retenu de 20% et 10% vous pouvez faire l’achat local pour le médicament local, donc au BCZ on part se ravitailler pour l’argent qui est cash et cet argent d’ASRAMES n’est pas encore arrive.

**P28: IA_02_AS_IT_04.rtf - 28:52 [Répondant : Mi-juillet on étai..] (113:113)	(Super)**

Codes:	[Gratuité_Interruption]

No memos

**Répondant** : Mi-juillet on était venu du 1^er^ au 15 et janvier du 1^er^ au 15 ça fait deux fois, il y a eu deux reprises.

**P28: IA_02_AS_IT_04.rtf - 28:53 [Répondant : Il n’y avait pas p..] (117:117)	(Super)**

Codes:	[SNIS_fonctionnement_Decrire_pdt MVE]

No memos

**Répondant** : Il n’y avait pas par ce qu’avant Ebola on faisait toujours le rapport hebdomadaire, chaque semaine il faut que tu envoies le rapport, à moins que tu tombes malade notification immédiate ça tu peux directement relever et envoyer à l’immédiat. Même aujourd’hui par ce que si c’est un cas suspect il faut envoyer directement par ce que c’est une maladie qui est immédiat.

**P28: IA_02_AS_IT_04.rtf - 28:54 [Répondant : Nous approchons ve..] (119:119)	(Super)**

Codes:	[SNIS_fonctionnement_Decrire_pdt MVE]

No memos

**Répondant** : Nous approchons vers la fin de l’épidémie,

**P28: IA_02_AS_IT_04.rtf - 28:55 [Répondant : Au début pour tout..] (121:121)	(Super)**

Codes:	[SNIS_fonctionnement_Decrire_pdt MVE]

No memos

**Répondant** : Au début pour tout cas il fallait alerter

**P28: IA_02_AS_IT_04.rtf - 28:56 [Répondant : Donc on avait peu ..] (123:123)	(Super)**

Codes:	[SNIS_fonctionnement_Decrire_pdt MVE]

No memos

**Répondant** : Donc on avait peu de temps pour faire l’analyse de donnée par ce que soit les gens sont à des formations, soit le forum n’est pas là mais quand même on s’est donner pour dire un mot. 1, on était surchargé avec de rapport et formation soit l’un part à la formation du CPI soit le PRESICO n’est pas là avec l’IT mais on se forçait toujours pour envoyer le rapport, mais ça peut arriver et tu es en retard c’est là où il y avait un moment pour la promptitude les gens arrivent en retard mais ils vont toujours remettre.

**P28: IA_02_AS_IT_04.rtf - 28:57 [Répondant : Et on faisait les ..] (125:125)	(Super)**

Codes:	[SNIS_fonctionnement_Decrire_pdt MVE]

No memos

**Répondant** : Et on faisait les analyses.

**P28: IA_02_AS_IT_04.rtf - 28:58 [Répondant : Nous normalement o..] (127:127)	(Super)**

Codes:	[SNIS_fonctionnement_Decrire_pdt MVE]

No memos

**Répondant** : Nous normalement on part on bureau central pour analyser les rapports, lorsque on les envoie eux aussi partent les encode là-bas puis on nous appelle nous les IT pour que chacun défend son rapport chaque mois par ce qu’eux ne sont pas surcharger mais in fait les réunions mensuelles.

**P28: IA_02_AS_IT_04.rtf - 28:59 [Répondant : Il y avait une col..] (131:131)	(Super)**

Codes:	[Gouvernance_soins santé]

No memos

**Répondant** : Il y avait une collaboration par ce que l’équipe qui est venu était collaborant vraiment lorsqu’ils viennent il se rabaisse au niveau de l’aire de santé par ce que lorsqu’ils viennent ils nous disent qu’eux dépendent de l’IT c’est le titulaire de l’aire de santé et l’IT va tout le montre, ils étaient vraiment simples à ce niveau.

**P28: IA_02_AS_IT_04.rtf - 28:60 [Répondant : Mois je ne peux pa..] (133:133)	(Super)**

Codes:	[Gouvernance_soins santé]

No memos

**Répondant** : Mois je ne peux pas dire mal, peut-être d’autres disaient cela par ce qu’ils pensaient que ces gens-là vont les engagés, hors c’est n’était pas comme ça par ce qu’eux aussi sont engagé et ils sont déjà arrivé à l’aire de santé. Ils ne peuvent pas engager une personne à l’aire de santé, c’est là où ils ne s’attendaient pas bien et ils ne savaient pas que l’engagement était au niveau supérieur. Nous ici on avait vu que l’équipe de personnel était insuffisante donc personne ne pouvait aller à un niveau de riposte par ce que celui qui va partir déjà il y aura un trou au centre c’est ce que on avait vu que celui qui va partir là-bas il perd directement sa place à la structure par ce que tu ne sauras pas servir en circulant alors que l’équipe n’est pas suffisante. Mais a hier aux équipes les prestataires et nombreux et c’est qui avait fait à ce qu’ils entrent dans d’autres activités, mais moi je ne peux pas dire par ce qu’ils arrivent ici et on collabore avec en tout cas.

**P28: IA_02_AS_IT_04.rtf - 28:61 [Répondant : Oui chaque mois.] (135:135)	(Super)**

Codes:	[Réunion_pdt-épidémie-Ebola]

No memos

**Répondant** : Oui chaque mois.

**P28: IA_02_AS_IT_04.rtf - 28:62 [Répondant : Nous avons leur fe..] (137:137)	(Super)**

Codes:	[Réunion_pdt-épidémie-Ebola]

No memos

**Répondant** : Nous avons leur feed-back et on se forçait par ce qu’eux aussi étaient dans leurs activités au centre mais ils vont profiter une journée pour se mettre ensemble.

**P28: IA_02_AS_IT_04.rtf - 28:63 [Répondant : Donc lorsque l’épi..] (139:139)	(Super)**

Codes:	[Leçons apprises_Problèmes_pdt MVE]

No memos

**Répondant** : Donc lorsque l’épidémie était venue c’était comme une chose étrange dans la communauté on ne savait pas que cette maladie pourrait s’accentué comme ça par ce que lorsque on partait à des formations on pensait que c’est, alors lorsque ils étaient venu ce vrai ils étaient avec les prestataires par ce que tous venaient de la hiérarchie, vous allez travailler avec ce gens et on pensait que par ce que on avait pas encore vu d’autre on pensait qu’ils sont spécial, que là-bas ils sont formé d’une autre manière mais on avait vu par après que, par exemple la vaccination ils amènent une équipe pour la vaccination on pensait qu’ils sont venu de Kinshasa ou de la Guinée mais on était surpris de voir qu’ils ont étaient formés au niveau local. Cela qu’on avait vu que ces gens s’ils allaient nous associer à l’équipe de vaccination la communauté ne pourrait pas refuser le vaccin. Là nous avons remarqué une première erreur.

**P28: IA_02_AS_IT_04.rtf - 28:64 [Répondant : Donc pour la vacci..] (141:141)	(Super)**

Codes:	[Leçons apprises_Problèmes_pdt MVE]

No memos

**Répondant** : Donc pour la vaccination par exemple, la vaccination lorsqu’il y avait le 1^er^ cas confirmé dans l’aire de santé, normalement ce qui causer une résistance dans la communauté c’est le fait de ne pas associer les infirmiers locaux qui s’occupent de la vaccination. Normalement ils devraient faire ce ci soit là nous allons mettre un ring vous les infirmiers qui faites là-bas la vaccination, d’abord les infirmiers devraient être briffé de la manière dont ils vont utiliser le vaccin d’Ebola comme ça lorsqu’il y a un cas ces mêmes infirmiers s’associes pour passer à la vaccination au même moment.

**P28: IA_02_AS_IT_04.rtf - 28:65 [Répondant : Bon d’abord la vac..] (143:144)	(Super)**

Codes:	[Leçons apprises_Problèmes_pdt MVE]

No memos

**Répondant** : Bon d’abord la vaccination ne marche pas, donc c’est la non intégration des agents de l’aire de santé dans la vaccination, on avait trouvé ça mal t c’est ce qui avait causé une forte résistance. Ils avaient peur par ce que les gens disaient comment il y a deux vaccins pour des différentes équipes un pour la routine et l’autre pour Ebola.

Bon une autre chose, pour le cas d’alerte je ne peux pas parle mal par ce qu’il y avait une convention, par ce qu’ils commençaient à demander s’il pourrait envoyer une personne au CTE vous tous si vous êtes d’accord et que la personne réponde à la définition.

**P28: IA_02_AS_IT_04.rtf - 28:66 [Répondant : Ça d’abord c’est p..] (146:146)	(Super)**

Codes:	[Leçons apprises_Problèmes_pdt MVE]

No memos

**Répondant** : Ça d’abord c’est parmi le bien, un autre mal qu’est-ce que je peux dire dalleurs principalement c’est le vaccin qui avait causé qui est une très forte résistance vraiment ça c’est un point qui nous avait laissé tomber.

**P28: IA_02_AS_IT_04.rtf - 28:67 [Répondant : Non cela suffit.] (148:148)	(Super)**

Codes:	[Commentaire-Recommandation_Répondant]

No memos

**Répondant** : Non cela suffit.

**P28: IA_02_AS_IT_04.rtf - 28:68 [Répondant : Moi si on appelle ..] (150:150)	(Super)**

Codes:	[Souhait_Changement_gestion_futures_épidémies]

No memos

**Répondant** : Moi si on appelle pour dire qu’il y a une épidémie qui arrive je déjà compris que je dois commencer dans la communauté, parler au leader de la communauté au bas niveau ce vrai on peut venir mais en commença par le bas niveau par ce que c’est ces gens-là qui sont mieux placer pour faire comprendre les autres en disant de bonnes ou mauvaises choses, par ce que on doit commencer d’abord à le contacter après c’est eux qui vont parlaient à la population c’est qu’on va faire. Par ce que lorsque ça commencer au-dessus il était difficile de s’adapter par ce qu’ils se demandaient ces gens nous amène quoi, ils viennent d’où.

**P29: IA_02_AS_PCOSA_03.rtf - 29:1 [Répondant : Nombreux sont ceux..] (9:9)	(Super)**

Codes:	[Gratuité_Accueil_population]

No memos

**Répondant** : Nombreux sont ceux qui disaient qu’ils ne voulaient pas de cette gratuité parce qu’elle était une forme de vouloir exterminer tout le monde qui en bénéficiera. Certains voulaient d’ailleurs être soignés là où ils vont payer dans les structures de santé privées.

**P29: IA_02_AS_PCOSA_03.rtf - 29:2 [Répondant : Oui, certains ont ..] (11:11)	(Super)**

Codes:	[Gratuité_Accueil_population]

No memos

**Répondant** : Oui, certains ont accepté et avaient suivi ces soins gratuits.

**P29: IA_02_AS_PCOSA_03.rtf - 29:3 [Répondant : Ils pensaient que ..] (13:13)	(Super)**

Codes:	[Triage_Considération_population]

No memos

**Répondant** : Ils pensaient que lorsque quelqu’un passe par le triage, on aura tendance qu’il a Ebola et le transférer au CTE et arrivé là il va vite mourir cat tout le monde qui arrive ici succombe.

**P29: IA_02_AS_PCOSA_03.rtf - 29:4 [Répondant : Oui] (15:15)	(Super)**

Codes:	[Triage_comportement_patient]

No memos

**Répondant** : Oui

**P29: IA_02_AS_PCOSA_03.rtf - 29:5 [Répondant : Oui] (17:17)	(Super)**

Codes:	[Structure-Service_Utilisation_population]

No memos

**Répondant** : Oui

**P29: IA_02_AS_PCOSA_03.rtf - 29:6 [Répondant : Les gens n’étaient..] (19:19)	(Super)**

Codes:	[Considération_prestataire_EPI_population]

No memos

**Répondant** : Les gens n’étaient pas habitués à voir ces genres de pratique où les infirmiers se masquent durant les heures de travail.

**P29: IA_02_AS_PCOSA_03.rtf - 29:7 [Répondant : Oui] (21:21)	(Super)**

Codes:	[Considération_Riposte_Population]

No memos

**Répondant** : Oui

**P29: IA_02_AS_PCOSA_03.rtf - 29:8 [Répondant : Il y en avait asse..] (23:23)	(Super)**

Codes:	[Agression-Menaces_Structure-Prestataire_raisons]

No memos

**Répondant** : Il y en avait assez

**P29: IA_02_AS_PCOSA_03.rtf - 29:9 [épondant : Le fait que l’on in..] (25:25)	(Super)**

Codes:	[Agression-Menaces_Structure-Prestataire_raisons]

No memos

**épondant** : Le fait que l’on interdisait aux gens de ne pas toucher une personne décédée d’Ebola et que c’étaient les secouristes qui devaient l’enterrer ; le fait de venir désinfecter les maisons, tous cela faisait que les gens résistent parce que certaines pratiques étaient anéanties dans la société.

**P29: IA_02_AS_PCOSA_03.rtf - 29:10 [Répondant : Oui, ceux qui arri..] (27:27)	(Super)**

Codes:	[Considération_prestataire_EPI_population]

No memos

**Répondant** : Oui, ceux qui arrivaient en étaient contents.

**P29: IA_02_AS_PCOSA_03.rtf - 29:11 [Répondant : Ils disaient qu’il..] (29:29)	(Super)**

Codes:	[Traitement_Invasifs_Prestataire_changement]

No memos

**Répondant** : Ils disaient qu’ils ne bénéficiaient que des comprimés et non des injections comme avant car certains aiment seulement ces injections.

**P29: IA_02_AS_PCOSA_03.rtf - 29:12 [Répondant : Les soignants disa..] (31:31)	(Super)**

Codes:	[Traitement_Invasifs_Prestataire_changement]

No memos

**Répondant** : Les soignants disaient que c’était seulement cela qui était disponible

**P29: IA_02_AS_PCOSA_03.rtf - 29:13 [Répondant : Quand on demandait..] (33:33)	(Super)**

Codes:	[Traitement_Invasifs_Prestataire_changement]

No memos

**Répondant** : Quand on demandait cette question, on nous disait que cela dépendait de la maladie de chacun parce que certains peuvent être injectés et leurs donner des comprimés et d’autres non. Surtout c’est lorsque le malade est hospitalisé qu’il reçoit des injections.

**P29: IA_02_AS_PCOSA_03.rtf - 29:14 [Répondant : Nombreux sont ceux..] (35:35)	(Super)**

Codes:	[Gratuité_Accueil_population]

No memos

**Répondant** : Nombreux sont ceux qui en avaient peur.

**P29: IA_02_AS_PCOSA_03.rtf - 29:15 [Répondant : Oui] (37:37)	(Super)**

Codes:	[Réunion_pdt-épidémie-Ebola]

No memos

**Répondant** : Oui

**P29: IA_02_AS_PCOSA_03.rtf - 29:16 [Répondant : Oui] (39:39)	(Super)**

Codes:	[Réunion_pdt-épidémie-Ebola]

No memos

**Répondant** : Oui

**P29: IA_02_AS_PCOSA_03.rtf - 29:17 [Répondant : Non.] (41:41)	(Super)**

Codes:	[Réunion_pdt-épidémie-Ebola]

No memos

**Répondant** : Non.

**P29: IA_02_AS_PCOSA_03.rtf - 29:18 [Répondant : Nous avions voulu ..] (43:43)	(Super)**

Codes:	[Réunion_pdt-épidémie-Ebola]

No memos

**Répondant** : Nous avions voulu rester avec une connaissance quant à l’Ebola parce que c’est dans cette entité où cette épidémie a commencé.

**P29: IA_02_AS_PCOSA_03.rtf - 29:19 [Répondant : Oui. Certaines str..] (49:49)	(Super)**

Codes:	[Structure-Service_Fermeture_pdt MVE]

No memos

**Répondant** : Oui. Certaines structures ont fermé leurs services par peur

**P29: IA_02_AS_PCOSA_03.rtf - 29:20 [Répondant : Ils avaient peur d..] (51:51)	(Super)**

Codes:	[Structure-Service_Fermeture_pdt MVE]

No memos

**Répondant** : Ils avaient peur de la maladie d’EBOLA pour que la communauté ne dise pas qu’ils étaient complices dans la mort de personnes. On disait dans la communauté que c’est l’infirmier qui était entrain de tuer les personnes qui mouraient d’Ebola.

**P29: IA_02_AS_PCOSA_03.rtf - 29:21 [Répondant : On disait que l’in..] (53:53)	(Super)**

Codes:	[Structure-Service_Fermeture_pdt MVE]

No memos

**Répondant** : On disait que l’infirmier était devenu un assassin parce que lorsque le malade arrivait, on l’injectait pour qu’il meure.

**P29: IA_02_AS_PCOSA_03.rtf - 29:22 [Répondant : Au CTE ou bien ail..] (55:55)	(Super)**

Codes:	[Référence-contre-référence_fonctionnement]

No memos

**Répondant** : Au CTE ou bien ailleurs ?

**P29: IA_02_AS_PCOSA_03.rtf - 29:23 [Répondant : Lorsque les soins ..] (57:57)	(Super)**

Codes:	[Qualité_Soins_Patients]

No memos

**Répondant** : Lorsque les soins étaient gratuits, on ne suivait pas les malades convenablement parce qu’il ne va rie payer. Par exemple quelqu’un qui va subir une opération et qui est arrivé au matin, sera masqué à treize heures par quelques petits comprimés jusqu’au lendemain. Les garde-malades étaient alors curieux de poser la question de savoir si leur malade n’allait bénéficier des soins ; c’est ce qui se passait dans les hôpitaux.

**P29: IA_02_AS_PCOSA_03.rtf - 29:24 [Répondant : Non, là on soignai..] (59:59)	(Super)**

Codes:	[Qualité_Soins_Patients]

No memos

**Répondant** : Non, là on soignait

**P29: IA_02_AS_PCOSA_03.rtf - 29:25 [Répondant : C’est parce qu’il ..] (61:61)	(Super)**

Codes:	[Qualité_Soins_Patients]

No memos

**Répondant** : C’est parce qu’il y avait la gratuité et l’existence de l’Ebola dans le zone.

**P29: IA_02_AS_PCOSA_03.rtf - 29:26 [Répondant : Les soignants avai..] (63:63)	(Super)**

Codes:	[Prestataire_Attitude_devant_malade]

No memos

**Répondant** : Les soignants avaient peur d’être contaminés de cette épidémie ce qui créait en eux une réticence de toucher les malades.

**P29: IA_02_AS_PCOSA_03.rtf - 29:27 [Répondant : Oui. On disait que..] (65:65)	(Super)**

Codes:	[Prestataire_Attitude_devant_malade]

No memos

**Répondant** : Oui. On disait que les infirmiers ne se souciaient plus de la prise en charge des malades.

**P29: IA_02_AS_PCOSA_03.rtf - 29:28 [Répondant : De tous les malade..] (67:67)	(Super)**

Codes:	[Prestataire_Attitude_devant_malade]

No memos

**Répondant** : De tous les malades oui étant donné qu’ils ne s’occupaient plus de leur prise en charge comme c’était auparavant.

**P29: IA_02_AS_PCOSA_03.rtf - 29:29 [Répondant : On disait aux gens..] (69:69)	(Super)**

Codes:	[Prestataire_Attitude_devant_malade]

No memos

**Répondant** : On disait aux gens que les infirmiers cherchaient aussi à se protéger de l’épidémie d’Ebola parce qu’on ne pouvait pas savoir que tel malade souffre de telle ou telle autre maladie ; Et que c’était une maladie qui ne fait de choix que tel est infirmier et ne peut être contaminé.

**P29: IA_02_AS_PCOSA_03.rtf - 29:30 [Répondant : Oui, certaines dén..] (71:71)	(Super)**

Codes:	[Prestataire_Attitude_devant_malade]

No memos

**Répondant** : Oui, certaines dénonçaient parce qu’il fallait que les soignant puissent d’abord se protéger avant de toucher la maman qui voulait accoucher.

**P29: IA_02_AS_PCOSA_03.rtf - 29:31 [Répondant : Oui] (73:73)	(Super)**

Codes:	[Prestataire_Attitude_devant_malade]

No memos

**Répondant** : Oui

**P29: IA_02_AS_PCOSA_03.rtf - 29:32 [Répondant : Je voudrais dire i..] (75:75)	(Super)**

Codes:	[Considération_RECO_depuis MVE]

No memos

**Répondant** : Je voudrais dire ici que les relais communautaires ont un travail arasant mais sans salaire de leur part. Ils ont un travail dur et ce sont à qui on a toujours jeté des pierres dans la communauté.

**P29: IA_02_AS_PCOSA_03.rtf - 29:33 [Répondant : Oui les gens sont ..] (77:77)	(Super)**

Codes:	[Considération_RECO_depuis MVE]

No memos

**Répondant** : Oui les gens sont devenus des impolis dans la communauté. Lorsqu’ils voient un relai communautaire, on l’appelle « Ebola ». Ils sont beaucoup injuriés

**P29: IA_02_AS_PCOSA_03.rtf - 29:34 [Répondant : Si l’Ebola revenai..] (79:79)	(Super)**

Codes:	[Souhait_Changement_gestion_futures_épidémies]

No memos

**Répondant** : Si l’Ebola revenait, je peux demander à la communauté de se protéger beaucoup en se lavant les mains, en évitant de toucher les cadavres des personnes mortes d’Ebola, en cas d’un malade l’orienter vite aux soins de santé à l’hôpital afin d’éviter qu’il ne meure à la maison.

**P30: IA_02_BCZS_IS_01.rtf - 30:1 [Répondant : Au niveau de ce bu..] (8:8)	(Super)**

Codes:	[Répondant_Responsabilité_habituelles]

No memos

**Répondant** : Au niveau de ce bureau qui est le bureau central de la zone de sante de Bembo je suis un superviseur et aujourd’hui nous travaillons d’une manière polyvalente mais spécifiquement je suis dans des activités de la vaccination pour les enfants de notre zone de sante c. a. d la cible et de 0 à 11 mois

**P30: IA_02_BCZS_IS_01.rtf - 30:2 [Répondant : Si on dit polyvale..] (10:10)	(Super)**

Codes:	[Répondant_Responsabilité_habituelles]

No memos

**Répondant** : Si on dit polyvalente ça signifie que en faisant la vaccination des enfants nous nous occupons à même temps de la surveillance épidémiologique donc pour dire que tous les malades sous surveillance, du moment qui a un cas on doit investiguer, notifier et prélever les échantillon que nous envoyons au niveau de l’antenne PEV pour que ces échantillons soient envoyé au niveau de laboratoire nationale pour voir si c’est ne pas l’une de maladie sous surveillance qui sera entre le service dans le milieu

**P30: IA_02_BCZS_IS_01.rtf - 30:3 [Répondant : Ok merci pour la q..] (12:12)	(Super)**

Codes:	[Début-Progression_Epidémie MVE]

No memos

**Répondant** : Ok merci pour la question, Ebola est entré dans Butembo en provenance de la zone de santé de Mabalako et plus précisément de Mangina, et quand la maladie a commencé à Mangina on a informé la population. nous avons commencé à sensibiliser la population, et comme il n’y avait pas de mesures de quarantaine pour que les gens ne puissent pas quitter Mangina, alors que Butembo et alimenter par ce coin la, ce comme ça que quand la maladie a commencé les gens ont commençaient à fuir Mangina pour se réfugier ici à Butembo et c’est comme ça que nous avons eu à partir du mois d’aout 2018 le 1^er^ cas dans notre aire de santé de Vutsundo, et c’était une maman qui était venue de Mangina et qui a amener la maladie ici à Butembo

**P30: IA_02_BCZS_IS_01.rtf - 30:4 [Répondant : Nous sommes là dep..] (14:14)	(Super)**

Codes:	[Considération_Ebola_population]

No memos

**Répondant** : Nous sommes là depuis le début de l’épidémie et jusqu’à ce que la maladie est entre d’être contrôlée, et la population n’a pas compris que réellement Ebola existe mais a définitive la population a compris quand elle a vue de portes fermées et quand il y a eu au moins 4 à 5 personnes décédées dans une ménage, si non quand la maladie est arrivée le gens ne croyaient pas surtout que dans notre milieu ils n’ont jamais vaincu aucune épidémie et il non jamais vu comment le gens meurent d’une maladie quelconque et c’est comme ça que les gens ont acceptaient difficilement surtout que dans la prise en charge était vraiment très vertical donc, les gens sont venues dans haut pour venir former à la base, les gens qu’on ne comprenaient même pas et qu’on ne connaissaient même pas alors il a était difficile de le recevoir. C’est comme ça que vers la fin quand la maladie était déjà là, quand on a impliqué les locaux c’est comme ça que le gens ont commençaient à comprendre que réellement la maladie existe

**P30: IA_02_BCZS_IS_01.rtf - 30:5 [Répondant : Oui le problème c’..] (16:16)	(Super)**

Codes:	[Agression-Menaces_Structure-Prestataire_raisons]

No memos

**Répondant** : Oui le problème c’est ce que je disais, je dirais pas peut être que pas tellement de finance, donc ils ont compris que les gens qui prestaient dans la riposte contre Ebola, ils ont étaient rémunérés de la manière qu’ils n’ont jamais compris qu’on pouvait être énuméré de cette manière, la population croyait qu’on est entre de fabriquer la maladie pour que les gens aient vraiment le sous et c’est comme ça que la population s’attaquer un moment au prestataire jusqu’à ce que le prestataire on commencer aussi à prendre l’Eilat et chercher à se retirer dans la riposte

**P30: IA_02_BCZS_IS_01.rtf - 30:6 [Répondant : L’offre de service..] (18:18)	(Super)**

Codes:	[Structures-Services_fonctionnement]

No memos

**Répondant** : L’offre de service comme vous venez de le dire a était tellement affecter de telle sorte que la population ne voulait plus fréquenter ces services, donc les gens ont vu que non, le symptôme qu’on enseignait à la population qui sont les signes de la maladie cette dernière n’avait pas compris comment ces signes peuvent devenir les symptômes d’Ebola et comme ça les gens ont abandonné les structures de santé, ils ont commencé à se faire soignes et a s’acheter eux même le médicament dans l’Officine. Ils ont commencé à fuir vers les structures privées et autre chose maintenant on a instauré le système de triage. Le triage qui n’existait pas ici chez nous et quand on a expliqué à la population que c’est ça la norme qui devrait être les gens ne comprenaient plus ils ont lié triage à Ebola donc il suffit que vous alliez au niveau d’une structure de santé et que vous présentez les signes on vous amener au CTE, et là c’est pour aller vous tuer vous n’allez plus revenir. C’est comme ça que les gens ont abandonnés pour ce qui est des soins curatifs. Pour ce qui est par exemple vaccination la population s’est acharné auprès des prestataires de telle sorte que les infirmiers ont dits non, on ne saura plus travailler avec la population qui est entré de s’attaquer aux prestataires, il y a plus moyen que nous puissions rendre service à cette population qui n’est pas reconnaissante de ce que nous faisons à son regard. Ce comme ça qu’un moment les activités de vaccination aussi ont dégringolées, on a même stoppé un moment la vaccination jusqu’à ce que la population a compris et elle la commencée à se présenter au niveau des autorités urbaines pour négocier encore cette activité pour le bénéfice de leurs enfants.

**P30: IA_02_BCZS_IS_01.rtf - 30:7 [Répondant : Il y a eu de struc..] (20:20)	(Super)**

Codes:	[Structure-Service_Fermeture_pdt MVE]

No memos

**Répondant** : Il y a eu de structures qui ont étaient fermées par ce que si j’ai parlé du début de la maladie, par exemple la maladie a commencé au niveau de l’aire de sante de Vutsundo ou une maman qui était venue de Mangina a était observer au niveau de la structure pendant au moins 4 jours et après elle est revenue maintenant dans un état très avance elle a était référée au clinique universitaire et cela qu’elle est décédée et quand on a fait swab c’est là qu’on a constaté que c’était déjà la maladie. Il y a eu deux prestataires de ce centre de sante qui ont étaient atteint, ce sont d’ailleurs les deux premiers cas de Butembo et ç a été l’objet de la fermeture direct de cette structure jusqu’à ce que le contact qui était lié à ce cas soit sortie pour qu’on voit comment on peut décontaminer la structure, changer quelques matériels pour qu’on ré ouvre la structure. La 2ieme structure a était aussi dans cette même ère de santé, c’était un cas contact la maman mais qui se cacher et qui a était retrouver aussi dans une poste de santé dans cette même ère de santé. C’est une structure qui a était fermée aussi pendant plus au moins deux mois et ça repris le travail donc il y a eu des structures qui ont étaient affectées et qui ont fermées momentanément par ce qu’elles ont été victimes des attaques de la population face à cette maladie.

**P30: IA_02_BCZS_IS_01.rtf - 30:8 [Répondant : Bon en fait, il n’..] (22:22)	(Super)**

Codes:	[Instructions_pdt_MVE] [Service_vaccination_Fonctionnement]

No memos

**Répondant** : Bon en fait, il n’y avait pas eu des instructions comme telle mais il y avait seulement de précaution à prendre par ce que quand on reprit même avec la vaccination après la sensibilisation de la population qui réclamée le service, il y a eu de précaution qui on était prise pour que, quand vous vacciner les enfants il fallait prendre toutes les mesures de précaution donc les mesures de contrôle. Donc c’était pour chaque enfant il fallait qu’on ait tous les nécessaires pour qu’il soit vacciner dans des bonnes conditions pour qu’il ne soit pas contaminer par le prestataire, notamment le port de gant et le seringue d’usage unique et par l’enfant et tout d’une manière très appropriée

**P30: IA_02_BCZS_IS_01.rtf - 30:9 [Répondant : Tout à fait.] (24:24)	(Super)**

Codes:	[Instructions_pdt_MVE]

No memos

**Répondant** : Tout à fait.

**P30: IA_02_BCZS_IS_01.rtf - 30:10 [Répondant : Donc tous ces acte..] (26:26)	(Super)**

Codes:	[Traitement_Invasifs_Prestataire_changement]

No memos

**Répondant** : Donc tous ces actes devaient se faire d’une manière, il fallait que la nécessité soit là parce que si j’entre par exemple au niveau de nos laboratoires, c’est n’était que de liquide biologique qui pouvait facilement transmettre la maladie qu’on manipuler à ce niveau-là. C’est comme ça qu’il y a un certain examen par exemple qui ne pouvait plus être réaliser, je veux prendre par exemple TDR pour le diagnostic de la malaria et ça on a défendue à ce qu’on puisse plus faire, si la personne présente déjà le signe de la malaria il sera soigner comme présumer malaria sans plus manipuler le sang, par ce que vous ne saviez pas quelle type de malaria vous êtes entre de soigne. Et si au niveau des interventions aussi il fallait que la nécessité soit là pour qu’on ne puisse pas manipuler le liquide qui peut contaminer facilement les prestataires.

**P30: IA_02_BCZS_IS_01.rtf - 30:11 [Répondant : Du point de vu pri..] (28:28)	(Super)**

Codes:	[Qualité_soins_Prestataires]

No memos

**Répondant** : Du point de vu pris en charge je dirais que l’accueil a était plus amélioré mais c’est la population qui ne voyait pas, il croyait qu’ont amélioré peut-être certaine prestation par ce que on veut la capture pour qu’on les amenés à la CTE. Des histoires comme ça, mais si non l’accueil a était amélioré par ce que à partir du triage qui on était installé presque dans toutes les structures, ça nous a permis d’ouvrir l’œil avec beaucoup plus d’attention et ce qui nous a aider aussi à la gratuite de ce qui est venue. Alors les gens ont compris que non je peux fréquenter telle structure et bénéficier d’un soin à un moindre cout et ça amener à ce que le gens soit plus attiré vers le service de santé.

**P30: IA_02_BCZS_IS_01.rtf - 30:12 [Répondant : Oui à certains end..] (30:30)	(Super)**

Codes:	[Prestataire_Attitude_devant_malade]

No memos

**Répondant** : Oui à certains endroits c’était observé mais on a corrigé les plus vite possible par ce qu’avec les épis que les gens prenaient donc quand un malade arriver vous avez vos EPI, les équipements de protection individuelle vous le portez mais d’une manière spécifique, si vous voyez qu’avec le symptôme que le malade présente, vous allez lui orienter vers un service d’isolement ou les soins vont se poursuivre.

**P30: IA_02_BCZS_IS_01.rtf - 30:13 [Répondant : La mobilisation pa..] (32:33)	(Super)**

Codes:	[Ressources_humaines_Disponibilité et perturbations]

No memos

**Répondant** : La mobilisation pas tellement mais les gens ont été plus vigilant, on a été comme si c’est Ebola seulement qui existé alors que les autres pathologies aussi existaient. Si non je dire que ça perturber mais pas tellement par ce qu’après sensibilisation de la population les gens ont été reçu au niveau de structure, vraiment il n’y a pas eu tellement de problème.

C’est l’attaque de la population aux structures qui a était vraiment le plus grand problème.

**P30: IA_02_BCZS_IS_01.rtf - 30:14 [Répondant : Ce que je peux dir..] (35:35)	(Super)**

Codes:	[Ressources_humaines_conditions_travail]

No memos

**Répondant** : Ce que je peux dire et que les gens sont revenues maintenant à la raison, ils sont revenus à ce qu’ils ont étudiés à l’école donc, il y avait certaines précautions que les gens pouvaient prendre. Cette épidémie a permis aux gens de faire ce qu’ils devraient faire et nous espérons que c’était vraiment une leçon et ça permettra aux gens de revenir sur leur déontologie après cette épidémie.

**P30: IA_02_BCZS_IS_01.rtf - 30:15 [Répondant : Pendant la période..] (37:37)	(Super)**

Codes:	[Médicaments_disponibilité_fosa]

No memos

**Répondant** : Pendant la période de l’Ebola, ça n’a pas était un problème par ce que tous les services étaient disponible. Dont le vaccin et le médicament pour la maladie courante étaient disponible, même le sens qui nécessite le sang ils en’ont bénéficié avec toute précaution donc tout était disponible.

**P30: IA_02_BCZS_IS_01.rtf - 30:16 [Répondant : Tout à fait, penda..] (39:39)	(Super)**

Codes:	[SNIS_fonctionnement_Decrire_pdt MVE]

No memos

**Répondant** : Tout à fait, pendant la période de l’épidémie, pendant qu’il y avait une forte éclosion ça affecter le service. Quand l’épidémie a commencée on a recruté certain prestataire pour qu’ils aillent rendre service ailleurs et au niveau de structure, il y a eu je dirais insuffisance de personnel ou bien même le répondant qui est IT et son adjoint qui était appeler tout le temps dans de formation. Ils étaient appelés par ci par là pour aller aussi former d’autre, et ç’a affecter de telles sortes qu’on peut arriver au niveau de structure, les différents registres ne sont pas tellement remplis. S’il n’y a pas vraiment une bonne organisation automatiquement ça va affecter le rapportage ce qui dit rapportage donc au niveau de bureau central nous avons aussi le rapport en retard nous sommes obligées de descendre sur le terrain pour aller voir qu’est ce qui ne va pas, les analyses parfois qui ne se faisaient plus et même au niveau de bureau central ça un moment affecter. Et par après on se retrouver que non on doit continuer avec les activités d’une manière normale en faisant le suivi des activités de la riposte. Mais il fallait qu’on puisse voir aussi les activités de routine qui ne devraient pas souffrir au même moment.

**P30: IA_02_BCZS_IS_01.rtf - 30:17 [Répondant : Au départ la tenue..] (41:41)	(Super)**

Codes:	[Réunion_pdt-épidémie-Ebola]

No memos

**Répondant** : Au départ la tenue de réunion a aussi était affecter mais après on se ressaisi et aujourd’hui ça continue d’une manière normale.

**P30: IA_02_BCZS_IS_01.rtf - 30:18 [Répondant : Pas tellement par ..] (45:45)	(Super)**

Codes:	[Gouvernance_soins santé]

No memos

**Répondant** : Pas tellement par ce que la zone de santé pouvait prendre de décisions pour ce qui ne marche pas, mais un moment c’était je dirais pas affecter c’était vraiment bon je ne sais pas comment le dire, il y a eu de moment où il y a les internationaux commençaient à apprendre en otage les infirmiers de la zone par ce qu’ils ont étaient formes soit à PCI, soit dans d’autres thématique, pour qu’ils aillent former davantage dans d’autres zones de santé sans informer le bureau central. C’est la qui y a eu vraiment quelque chose qui déséquilibré un peu la zone mais on est revenue à la raison pour que les choses puissent marchées d’une manière normale.

**P30: IA_02_BCZS_IS_01.rtf - 30:19 [Répondant : Par rapport à ça j..] (48:48)	(Super)**

Codes:	[Gouvernance_soins santé]

No memos

**Répondant** : Par rapport à ça je ne dirais pas que ça soit vrai par ce que ces toutes le structures qui étaient concernées par la riposte et dans toutes les aires de sante on a eu de cas il y a eu de formation, au moins chaque prestataire avait un rôle à jouer pour qu’on puisse booster vraiment la maladie a virus Ebola.

**P30: IA_02_BCZS_IS_01.rtf - 30:20 [Répondant : Le changement norm..] (50:50)	(Super)**

Codes:	[Souhait_Changement_gestion_futures_épidémies]

No memos

**Répondant** : Le changement normalement qu’on voudrait que ça puisse être amener et que il y a eu l’épidémie bon c’est qu’on a eu comme problème ce que pour nous autre qui doivent être mis en place pour que on puisse contrôler la maladie, c’est pour ça que nous disons avec tout ce que nous avons vu, nous voudrions à ce que lorsqu’il y a une épidémie qui arrive il faudrait que ceux que les choses commences a la base donc au niveau de la communauté, au niveau de structure de sante que le prestataire qui soit la , qu’il soit appuyé par les gens qui viennent de l’extérieure par rapport à ce que nous avons vaincu donc ce sont les gens de l’extérieur qui ont apportés, ils ont d’abord former le locaux pour que a la base il n’y ait rien maintenant quand il fallait revenir à la base c’est ce qui a créer problème alors que cela qu’on devrait commencer donc le système a commencer en aval au lieu de commencer en amont.

**P30: IA_02_BCZS_IS_01.rtf - 30:21 [Répondant : Une épidémie c’est..] (52:52)	(Super)**

Codes:	[Commentaire-Recommandation_Répondant]

No memos

**Répondant** : Une épidémie c’est quelque chose qui ne peut pas être gérer par une personne, il faut qui est l’implication de tous et avec le système actuel de santé primaire surtout la population qui doit d’abord s’approprier ce qui est entré d’être fait pour que les choses puissent marchée normalement.

**P30: IA_02_BCZS_IS_01.rtf - 30:22 [Répondant : Comme rôle dans la..] (43:43)	(Super)**

Codes:	[Répondant_Responsabilité_pdt MVE]

No memos

**Répondant** : Comme rôle dans la riposte, quand les activités ont commencé moi j’ai travaillé surtout dans la recherche active, la recherche active ça signifier qu’il fallait que je fasse la cartographie de toutes les structures de la zone de sante. Il fallait qu’on mette de gens qui doivent vérifiés au niveau de chaque structure chaque jour s’il n’y a pas eu de cas suspect et qui n’a pas était orienter ou bien qui a était observé au niveau d’une autre structure, et par après on retrouve que c’est un cas qui par après va contaminer les gens, personnellement je fais la recherche active au niveau de structure de la zone de santé et il y a eu d’autre cas à part Ebola qu’on trouver, il fallait qu’on poursuive le deux au même moment. Et la surveillance des autres maladies dans la riposte, pendant l’épidémie de la maladie a virus Ebola on a aussi détecté que on était à double épidémie par ce que nous avons même eu le cas de la rougeole confirmer.

**P31: IA_02_HGR_AG.rtf - 31:1 [Répondant : Je travaille dans ..] (6:6)	(Super)**

Codes:	[Répondant_Responsabilité_habituelles]

No memos

**Répondant** : Je travaille dans le domaine de la gestion, je vois l’utilisation des ressources : matérielles, humaines, financières, je me charge de faire respecte la mesure de l’application des normes.

**P31: IA_02_HGR_AG.rtf - 31:2 [Répondant : Cette période n’a ..] (8:8)	(Super)**

Codes:	[Répondant_Responsabilité_pdt MVE]

No memos

**Répondant** : Cette période n’a pas été facile pour nous, dès que l’on a annonce l’épidémie, le gouvernement ainsi que les organismes avaient eu besoin des ressources surtout humaines, ou l’on fait appel aux personnels pour appuyer les équipes de la riposte. Comme il s’agissait de l’épidémie, il fallait isoler les cas, mais avant d’isoler, tous les cas passent par les structures. Alors on a eu des difficultés au niveau de la structure, il fallait équilibrer les services au niveau de la structure vu le personnel qui est allé. Il fallait accueillir les malades à l’hôpital, et transférer les cas qui répondent à la définition. On a eu aussi le rôle de stabiliser le fonctionnement normal des activités, sinon on serrait fragile, et les cas devraient dégénérer de l’hôpital vers la population

**P31: IA_02_HGR_AG.rtf - 31:3 [Répondant : J’ai eu du personn..] (10:10)	(Super)**

Codes:	[Ressources_humaines_Disponibilité et perturbations]

No memos

**Répondant** : J’ai eu du personnel qui est presque totalement aller à l’extérieur, il fallait recourir à une ressource extérieure, c’était le moyen de maintenir l’équilibre des activités. Au début on n’a pas eu la formation pour gérer le cas déclare suspect, mais au deuxième mois de l’épidémie, on a forme tous les personnels pour que nous puissions maitriser et contrôler cette maladie a l’hôpital.

**P31: IA_02_HGR_AG.rtf - 31:4 [Répondant : Cette grève était ..] (12:12)	(Super)**

Codes:	[Ressources_humaines_situation_grève]

No memos

**Répondant** : Cette grève était causée par la prime de risque du personnel de santé, comme ont envoyé pas le rapport, beaucoup des choses ont échappes au traitement et analyse des données. La grève a été plus à caractère administratif, le personnel lui travaillait et les activités se déroulaient dans la normale.

**P31: IA_02_HGR_AG.rtf - 31:5 [Répondant : Je pense que ça n’..] (14:14)	(Super)**

Codes:	[Ressources_humaines_situation_grève]

No memos

**Répondant** : Je pense que ça n’a pas dépassé trois mois.

**P31: IA_02_HGR_AG.rtf - 31:6 [Répondant : Avant Ebola chacun..] (16:16)	(Super)**

Codes:	[Ressources_humaines_conditions_travail]

No memos

**Répondant** : Avant Ebola chacun se mettait au travail en sachant qu’il travaille pour sa structure. Pendant l’épidémie, beaucoup avaient tendance d’aller travailler pour les organismes, les gens ont délaissé les activités dans les structures au profit de la riposte.

**P31: IA_02_HGR_AG.rtf - 31:7 [Répondant : En rapport avec l’..] (18:18)	(Super)**

Codes:	[Structures-Services_fonctionnement]

No memos

**Répondant** : En rapport avec l’offre, il y’a eu des moments qui sont diffèrent, au début de l’épidémie, on a annoncé la gratuite. Les activités sont devenues intenses au niveau de la consultation, de la médecine interne et au niveau de la pédiatrie, de novembre en février, on a annulé la maternité, la gynéco obstétrique et la chirurgie, on peut dire que pendant cette période, la maternité a régressé, elles ne venaient plus et la cause est qu’on les envoyait accoucher au CTE une fois qu’elles arrivaient ici. En avril on est reste avec la consultation des référés. Seule la maternité a régressé pendant cette période mais en aujourd’hui à la fin de l’épidémie, la maternité est en train de revenir à la normale.

**P31: IA_02_HGR_AG.rtf - 31:8 [Répondant : Quand on est dans ..] (20:20)	(Super)**

Codes:	[Service_maternité_fonctionnement] [Structure-Service_Utilisation_population]

No memos

**Répondant** : Quand on est dans l’image de l’HGR, on doit remplir toutes les conditions, nous avons eu triage au niveau de l’entrée mais il y’a eu des privées qui n’avaient pas ce système-là, c’est la ou les femmes voulaient accoucher, dans les structures qui n’ont pas le système de contrôle.

**P31: IA_02_HGR_AG.rtf - 31:9 [Répondant : Elles disaient que..] (22:22)	(Super)**

Codes:	[Triage_comportement_patient]

No memos

**Répondant** : Elles disaient que si elles arrivaient à l’hôpital, on les envoyait directement au CTE.

**P31: IA_02_HGR_AG.rtf - 31:10 [Répondant : Non, c’est reste s..] (24:24)	(Super)**

Codes:	[Prestataire_Attitude_devant_malade]

No memos

**Répondant** : Non, c’est reste statuquo, mais la qualité a diminué vu qu’il a eu trop de malades que d’habitude, au niveau de l’accueil on a pas eu de changement sauf que notre charge de la réception est aussi partie, il ne fallait pas perdre les gens, celui qui partait dans la riposte s’il pouvait avoir le temps de venir prester, on capitalisait ça. Au début, les gens qui étaient partis la y sont restes, on a été obligé de recruter.

**P31: IA_02_HGR_AG.rtf - 31:11 [Répondant : Les structures qui..] (26:26)	(Super)**

Codes:	[Structure-Service_Fermeture_pdt MVE]

No memos

**Répondant** : Les structures qui ont fermées, je ne pense pas, seulement pendant l’épidémie, il avait des évaluations dans le cadre de voir la source de contamination, il y’a des structures qui présentaient beaucoup des cas positifs. C’est dans ce cadre du protocole de l’OMS, de chaque fermer les structures qui présentaient beaucoup des cas mais il n’y a pas une structure qui a fermée parce qu’il a Ebola. Les structures qui n’étaient pas dans le programme de la gratuite étaient plus contre la sensibilisation en cadre d’Ebola.

**P31: IA_02_HGR_AG.rtf - 31:12 [Répondant : Toutes les activit..] (28:28)	(Super)**

Codes:	[Structures-Services_fonctionnement]

No memos

**Répondant** : Toutes les activités étaient normales dans le cadre de planification, mais c’est au niveau des exécutions qu’il avait problème à cause de l’absence surtout des ressources humaines. Chez nous la chirurgie était toujours dans la normale, comme je l’ai dit au début de la gratuite, même ceux qui avaient des problèmes qui dataient de très longtemps avaient tendance à venir, et les cas qui ont été maintenu, ont été intervenu. Après ce programme, on est retourné à la routine.

**P31: IA_02_HGR_AG.rtf - 31:13 [Répondant : Avant l’épidémie n..] (30:30)	(Super)**

Codes:	[Médicaments_disponibilité_fosa]

No memos

**Répondant** : Avant l’épidémie notre stock arrivait toujours à satisfaire les malades, on a évolué jusque pendant l’épidémie ou l’on a disponibilisé la gratuite, notre capital a monté, et a un certain moment dans la gratuite, on parvenait plus à honorer les factures, on renforçait le stock avec les réserves qu’on avait. La grande difficulté dans la gestion des médicaments, on nous avait dit qu’il avait 20% pour la gestion des médicaments dans le compte d’ASRAMESS et le 10% qu’on donne normalement à la structure. Mais on a fait 4 mois à partir du mois d’avril, entrain de payer avec uniquement le 10% alors qu’il fallait satisfaire les malades. On était en train de consomme le stock sans pour autant le renforcer. Et jusqu’à maintenant le 20% n’a jamais été livré à la structure.

**P31: IA_02_HGR_AG.rtf - 31:14 [Répondant : C’est l’EUP-FASS a..] (32:32)	(Super)**

Codes:	[Médicaments_disponibilité_fosa]

No memos

**Répondant** : C’est l’EUP-FASS avec l’ASRAMESS, car le contrat dit que l’EUP-FASS doit remettre le 20% à l’ASRAMESS pour qu’ils nous fournissent les médicaments, mais depuis avril, on n’a jamais livré

**P31: IA_02_HGR_AG.rtf - 31:15 [Répondant : Ça nous a perturbe..] (34:34)	(Super)**

Codes:	[Médicaments_disponibilité_fosa] [Structures-Services_fonctionnement]

No memos

**Répondant** : Ça nous a perturber dans le fonctionnement parce que le compte qu’on réservait pour l’investissement, on a été obligés de l’utiliser pour acheter les médicaments. Quand ça serra paye par le partenaire avec le tableau de bord qui peut nous renseigner quel est le compte qui a consommé pour l’autre, nous aurons la capacité d’équilibrer les affaires.

**P31: IA_02_HGR_AG.rtf - 31:16 [Répondant : Pour les malades l..] (36:36)	(Super)**

Codes:	[Médicaments_disponibilité_fosa]

No memos

**Répondant** : Pour les malades les médicaments c’est un besoin, et nous, nous offrons les services, et les médicaments, et les examens du labo et tous les nécessaires aux malades. Avec la gratuite, nous offrons les médicaments aux malades gratuitement mais la disponibilité des médicaments c’est au niveau de la pharmacie. Gratuite signifie donner les médicaments gratuitement mais il y’a quelqu’un qui doit acheter ces médicaments pour que nous ayons ça au niveau du dépôt.

**P31: IA_02_HGR_AG.rtf - 31:17 [Répondant : Avec le moyen de b..] (38:38)	(Super)**

Codes:	[Médicaments_disponibilité_fosa]

No memos

**Répondant** : Avec le moyen de bord que nous avons localement, nous avons tout fait pour acheter les médicaments et satisfaire la gratuite du malade.

**P31: IA_02_HGR_AG.rtf - 31:18 [Répondant : À notre niveau, il..] (40:40)	(Super)**

Codes:	[SNIS_fonctionnement_Decrire_pdt MVE]

No memos

**Répondant** : À notre niveau, il n’y a pas trop de changement au niveau de notre système, on faisait l’évaluation service par service, on évaluait la disponibilité des responsables des services. Si le responsable était parti à la riposte, on cherchait un responsable collaborateur a la personne qui est allé.

**P31: IA_02_HGR_AG.rtf - 31:19 [Répondant : Toutes les réunion..] (42:42)	(Super)**

Codes:	[Réunion_pdt-épidémie-Ebola]

No memos

**Répondant** : Toutes les réunions étaient en train de se tenir, voilà un peu la collecte des données, la transmission des rapports qui ont eu des problèmes.

**P31: IA_02_HGR_AG.rtf - 31:20 [Répondant : Nous avons ce qui ..] (44:44)	(Super)**

Codes:	[Gouvernance_soins santé]

No memos

**Répondant** : Nous avons ce qui était attendu, notamment toutes nos planifications ont été faite et les exécutions ont été faites. On a évalué l’année 2018 et 2019 car l’épidémie a commencé en 2018. On a toujours évalué, et avec le programme nous tenons toujours les réunions, nous avons le CODIR, comité de direction qui tient chaque semaine une réunion, le staff médical, le staff infirmière qui tient chaque semaine une réunion, et chaque mois nous faisons l’analyse des rapports. A notre niveau, la performance pour cette tache a été maintenu.

**P31: IA_02_HGR_AG.rtf - 31:21 [Répondant : Bien qu’il a des a..] (46:46)	(Super)**

Codes:	[Gouvernance_soins santé]

No memos

**Répondant** : Bien qu’il a des acteurs qui sont venus dans l’imprévu, il avait déjà des programmes établis et qu’ils soumettaient à la structure. Au niveau de la structure, on était en train de voir comment répondre à leur demande. C’est pourquoi il y’a eu des points focaux pour certaines activités, ces derniers nous permettaient de travailler avec le partenaire mais aussi nous aidaient à assumer nos responsabilité internes.

**P31: IA_02_HGR_AG.rtf - 31:22 [Répondant : Le mode était l’au..] (48:48)	(Super)**

Codes:	[Recouvrement_coûts-des-soins_avant gratuité]

No memos

**Répondant** : Le mode était l’autofinancement, on rendait service aux malades et après cela, on facturait ces services et les malades étaient appelés à payer ces factures par acte.

**P31: IA_02_HGR_AG.rtf - 31:23 [Répondant : La gratuite a été ..] (50:50)	(Super)**

Codes:	[Gratuité_Partenaire d'appui]

No memos

**Répondant** : La gratuite a été financée par le gouvernement lui-même via le programme PDSS, EIP-FACE a servi pour le dépôt ou l’acheminement de fond vers les structures.

**P31: IA_02_HGR_AG.rtf - 31:24 [Répondant : Dans la gratuite n..] (52:52)	(Super)**

Codes:	[Gratuité_Partenaire d'appui]

No memos

**Répondant** : Dans la gratuite non, c’est uniquement le gouvernement.

**P31: IA_02_HGR_AG.rtf - 31:25 [Répondant : Je ne sais pas vra..] (54:54)	(Super)**

Codes:	[Gratuité_Structures-Services_concernés]

No memos

**Répondant** : Je ne sais pas vraiment la définition, mais la plupart des structures on regardait les services intégrés, il fallait que les structures soient reconnues dans la zone et peut être ceux-là aussi qui sont créées à partir d’une autorité.

**P31: IA_02_HGR_AG.rtf - 31:26 [Répondant : les rimeurs étaien..] (56:56)	(Super)**

Codes:	[Gratuité_Rumeurs]

No memos

**Répondant** : les rimeurs étaient énormes parce que la population ne digérait pas le mot Ebola, ils ont pensé qu’Ebola est arrivé pour qu’on élimine la population d’ici. Aussi Ebola est apparu pendant la période électorale, ils ont alors dit qu’on a amené Ebola pour écarter la population de ce milieu. C’est pourquoi il avait une grande résistance, même les activités étaient menacées. C’est pourquoi il y’a des militaires dans toutes les structures.

**P31: IA_02_HGR_AG.rtf - 31:27 [Répondant : C’était difficile ..] (58:58)	(Super)**

Codes:	[Gratuité_Accueil_population]

No memos

**Répondant** : C’était difficile au début, les gens disaient qu’on a amené ces médicaments pour alors tuer les gens. Mais avec le temps, quand ils évaluaient que ceux qui le prenaient guérissaient, ils ont compris que ce n’était pas pour le tuer.

**P31: IA_02_HGR_AG.rtf - 31:28 [Répondant : Pendant la période..] (60:60)	(Super)**

Codes:	[Gratuité_Conséquences_motivation_prestataire]

No memos

**Répondant** : Pendant la période de gratuite, on a eu plus des malades et leurs factures ont été honorées par le gouvernement, bien que l’on donnait avec des retards, comme le début on nous donnait des avances, quand ces avances étaient données les personnels se sentaient mieux car ça devrait doubler ou tripler, une prime qu’on a jamais eu dans les recettes locales dans ce domaine. Seulement la démotivation est arrivée quand on a passé beaucoup de temps sans honorer les factures.

**P31: IA_02_HGR_AG.rtf - 31:29 [Répondant : Au début c’était p..] (62:62)	(Super)**

Codes:	[Gratuité_Conséquences_motivation_prestataire]

No memos

**Répondant** : Au début c’était presque égale a la riposte, mais on ne savait pas quel était le principe des bailleurs de fond, on a commencé avec la gratuite globale, quand on constant que les structures se retrouvaient bien, on a changé le contrat, ce que l’on aspirait à maintenir, c’est cassé encore. Si on pourrait laisser la gratuite globale, il n’aurait pas la déstabilisation.

**P31: IA_02_HGR_AG.rtf - 31:30 [Répondant : La première chose ..] (64:64)	(Super)**

Codes:	[Souhait_Changement_gestion_futures_épidémies]

No memos

**Répondant** : La première chose c’est de ne pas oublier les ressources humaines, le personnel et reste de changement dans le sens positif ça va aller mais si ont délaissé les personnels, il n’aura pas ce qui va s’améliorer par exemple si on me laisse seul sur cette structure et que les autres partent, je ne saurai pas m’en sortir. Pendant cette épidémie, il ne faut avoir des agitations dans les structures. Il faut aussi mètre les mesures qui peuvent sécuriser les structures, comme pendant l’épidémie la population ne comprend pas au même moment. Il a la sécurisation dans plusieurs sens, mettre par exemple le triage, ce dernier n’a que deux lits mais on accueil six malades que nous faisons trainer en attendant le transfert vers le CTE.

**P31: IA_02_HGR_AG.rtf - 31:31 [Répondant : Je demanderai une ..] (66:66)	(Super)**

Codes:	[Commentaire-Recommandation_Répondant]

No memos

**Répondant** : Je demanderai une observation par rapport avec certains partenaires, quand nous sommes dans les activités de routine de la structure, certains partenaires arrivent et n’attendent pas à ce que vous puissiez finaliser vos activités et vous demandent de commencer ce qu’ils vont vous demander. Aussi un constant, c’est l’affectation des ressources qui se passait de n’importe comment, on n’affectait pas des gens a des postes qu’ils méritent. Il a certaine personne, quelqu’un qu’on analysait et qu’on trouvait qu’il n’a pas fait le domaine qu’on lui a affecté. Une personne qui n’a jamais fait la psychologie mais c’est lui qui fait l’activité de psycho-social. Ça nous a créé beaucoup des problèmes. Ceux qui sont au niveau de prendre les décisions qu’ils essayent d’analyser tous ces paramètres avant d’affecter une personne qu’on voit si elle mérite le poste.

**P32: IA_02_HGR_DN.rtf - 32:1 [Répondant : Ok Merci je suis l..] (6:6)	(Super)**

Codes:	[Répondant_Responsabilité_habituelles]

No memos

**Répondant** : Ok Merci je suis le Directeur de Nursing, je m’occupe de la planification des soins dans la Structure en suite je suis le point focal de la surveillance épidémiologique dans la structure.

**P32: IA_02_HGR_DN.rtf - 32:2 [Répondant : Pendant la riposte..] (8:8)	(Super)**

Codes:	[Répondant_Responsabilité_pdt MVE]

No memos

**Répondant** : Pendant la riposte je fus dans la condition de prise en charge.

**P32: IA_02_HGR_DN.rtf - 32:3 [Répondant : Dans la condition ..] (10:10)	(Super)**

Codes:	[Répondant_Responsabilité_pdt MVE]

No memos

**Répondant** : Dans la condition je fus superviseur, dans le suivi des malades sortis non cas du CTE en suite l’écurie et enfin je m’occupe de la collecte des données du CTE Butembo.

**P32: IA_02_HGR_DN.rtf - 32:4 [Répondant : Ces données porten..] (12:12)	(Super)**

Codes:	[Répondant_Responsabilité_pdt MVE]

No memos

**Répondant** : Ces données portent sur la prise en charge : il faut voir les entrées, les admissions du jour, les soties et s’il eut un décès on peut planifie un audit par rapport à ce décès.

**P32: IA_02_HGR_DN.rtf - 32:5 [Répondant : Oui nous avons con..] (14:14)	(Super)**

Codes:	[Ressources_humaines_Disponibilité et perturbations]

No memos

**Répondant** : Oui nous avons connus un problème d’instabilité du personnel cela est réel.

**P32: IA_02_HGR_DN.rtf - 32:6 [Répondant : D’abord on peut co..] (16:16)	(Super)**

Codes:	[Ressources_humaines_Disponibilité et perturbations]

No memos

**Répondant** : D’abord on peut commencer sur les Médecins : sur cinq nous étions restés avec deux pérennants dans la structure, maintenant pendant la gratuité nous étions obligé de prendre un médecin de la zone de sante de Kayina mais qui travaille dans nos Œuvres Médicales de la communauté CEBCE durant les trois mois de gratuités. Ceux qui nous ont aidés sont de Médecins trainingueurs.

**P32: IA_02_HGR_DN.rtf - 32:7 [Répondant : Ils sont dans la r..] (18:18)	(Super)**

Codes:	[Ressources_humaines_Disponibilité et perturbations]

No memos

**Répondant** : Ils sont dans la riposte, les uns qui ne sont plus pérennants sont dans la vaccination et les autres dans la prise en charge. Pour les infirmiers nous avons eu de soucis et cela au niveau de service : il y a ceux qui sont dans la prise en charge au CTE, c’est qui sont au niveau du triage, ceux qui sont dans la recherche active dans l’aire de santé, ceux qui sont dans la recherche active dans les aires des santés voisines et ceux qui sont dans la surveillance dans la zone voisine. Ça nous crée de problème pour la rotation au niveau du service. Pour le garde nuit ils sont là mais pour la journée ils ne sont pas là. Ceux qui sont présent supporte la charge des absents.

**P32: IA_02_HGR_DN.rtf - 32:8 [Répondant : En tout cas la dir..] (20:20)	(Super)**

Codes:	[Ressources_humaines_Disponibilité et perturbations]

No memos

**Répondant** : En tout cas la direction n’a pas été impliqué, on n’avait pas planifié leur départ. Ils sont partis de leurs volontés, seulement ceux qui sont au niveau du triage la direction avait planifié leurs activités et comme le triage est déjà dans la structure ça ne pose plus de problème mais aussi ça déséquilibre également les services comme ils sont au nombre de vingt-deux sur soixante-douze vous remarquez qu’il y a un écart.

**P32: IA_02_HGR_DN.rtf - 32:9 [Répondant : Tout ce que nous v..] (22:22)	(Super)**

Codes:	[Ressources_humaines_Disponibilité et perturbations]

No memos

**Répondant** : Tout ce que nous vivons aujourd’hui a commencé dès le départ, par ce qu’on n’avait pas pris en compte les prestataires du milieu(locaux). Je dirai que c’est le niveau stratégique qui décidait à notre place. On n’avait pas considéré les équipes cadres du milieu puisqu’avec ces dernières savent tel travaille dans telle structure et s’il peut aider dans les actions de la riposte il peut travailler dans son rayon, ça pouvait faire avancer le travail malheureusement c’est ne pas ce qui était fait ; c’est pour quoi vous trouverai tel dans la zone de santé de Katwa alors qu’il œuvre dans la zone de santé de Butembo. Il y avait une lettre qui était venu de la DPS disant que ceux qui travaillent dans la riposte peuvent y rester et ce qui veulent travailler restent dans leurs services mais ça toujours crée de problème puisqu’on ne pouvait pas remplacer ceux qui était dans la riposte par ce que la DPS a dit de ne pas engager ça été un problème. Bref il n’y avait pas l’implication de l’équipe cadre. Le recrutement des agents étaient liés par la connaissance sans tenir compte du profil de quelqu’un.

**P32: IA_02_HGR_DN.rtf - 32:10 [Répondant : On était obligé d’..] (24:24)	(Super)**

Codes:	[Ressources_humaines_Disponibilité et perturbations]

No memos

**Répondant** : On était obligé d’adapter l’horaire par rapport à l’état d’emploi, ceux qui sont dans la zone de santé voisine ils étaient obligé de faire les gardes nuits, pendant la journée ils posent problème et même s’ils viennent pour les gardes nuits, ils sont toujours fatigués c’est pourquoi nous rencontrons des erreurs.

**P32: IA_02_HGR_DN.rtf - 32:11 [Répondant : Les erreurs qu’on ..] (26:26)	(Super)**

Codes:	[Ressources_humaines_Disponibilité et perturbations]

No memos

**Répondant** : Les erreurs qu’on trouvent il y a des malades qu’ils peuvent oublier. C’est ça le problème.

**P32: IA_02_HGR_DN.rtf - 32:12 [Répondant : La journée ça ne p..] (28:28)	(Super)**

Codes:	[Ressources_humaines_Disponibilité et perturbations]

No memos

**Répondant** : La journée ça ne pose pas tout problème, comme il y a ceux qui travaillent au triage on fait recours à eux. Là il n’y a pas de soucis seulement la nuit. Pendant la journée on pouvait avoir deux personnes dans chaque service et pratiquement c’est une personne dans chaque service. Et c’est seule personne qui va faire : la réquisition ; l’administration des médicaments et elle va recevoir les malades. Elle est surchargée.

**P32: IA_02_HGR_DN.rtf - 32:13 [Répondant : Oui ça des conséqu..] (30:30)	(Super)**

Codes:	[Qualité_soins_Prestataires]

No memos

**Répondant** : Oui ça des conséquences sur la qualité des soins, quelqu’un qui est fatigué ne peut pas bien travailler car à part sa fatigue il est seul aussi.

**P32: IA_02_HGR_DN.rtf - 32:14 [Répondant : Il y a des oublis ..] (32:32)	(Super)**

Codes:	[Qualité_soins_Prestataires]

No memos

**Répondant** : Il y a des oublis dans nos prestations à cause de cela, par exemple le médecin peut faire le tour de salle, peut recommander tel médicament mais il peut oublier de faire la réquisition et pendant la garde nuit le malade sera sacrifié à cause de cette oublie.

**P32: IA_02_HGR_DN.rtf - 32:15 [Répondant : Avant l’épidémie i..] (34:34)	(Super)**

Codes:	[Ressources_humaines_conditions_travail]

No memos

**Répondant** : Avant l’épidémie il n’avait pas de problème je peux le dire, tout le monde était là ; il n’y avait pas de soucis. Comme chacun court derrière l’argent c’est normal et c’est le grand problème. Est-ce on peut dire qu’on puisse mettre ce qu’ils ont gagné dans une corbeille commune je ne sais pas. Nous on leurs avait demandé d’apporter le dix pour cent de ce qu’ils gagnent dans la riposte (10%) et le mettre dans une corbeille commune pour suppléer aux autres qui n’était pas dans la riposte. Ce qu’on avait constaté si on les disait d’apporter la somme en globalité ça pourrait créer de problème. De toute la façon ils amènent ce qu’on leurs avait dit le dix pour cent de ce qu’ils gagnent là-bas (10%).

**P32: IA_02_HGR_DN.rtf - 32:16 [Répondant : Si je peux évaluer..] (36:36)	(Super)**

Codes:	[Ressources_humaines_conditions_travail]

No memos

**Répondant** : Si je peux évaluer d’abord les infirmiers ; nous avons en médecine interne une personne qui fait seulement des gardes nuits, il s’efforce et pour lui pas de soucis même s’il est dans la zone de santé de Katwe la ZS voisine dans la recherche active. Seulement quand il est programmé la journée le dimanche c’est là que pose problème, elle est obligée à faire recourt à une autre personne de son service, il peut accepter tout comme il peut refuser. Pour la salle d’urgence : les soins intensifs ne posent pas problème. Globalement pour les infirmiers ça ne pose pas de problème, c’est seulement chez les médecins où il y a des soucis.

**P32: IA_02_HGR_DN.rtf - 32:17 [Répondant : Non il n’y a pas e..] (38:38)	(Super)**

Codes:	[Structure-Service_Fermeture_pdt MVE]

No memos

**Répondant** : Non il n’y a pas eu un service qui a fermé.

**P32: IA_02_HGR_DN.rtf - 32:18 [Répondant : En dehors de l’hôp..] (40:40)	(Super)**

Codes:	[Structure-Service_Fermeture_pdt MVE]

No memos

**Répondant** : En dehors de l’hôpital il y a des structures qui ont fermé comme elles ne voulaient pas appliquer la PCI, elles ont été résistantes par rapport aux actions de la riposte. Par exemple dans notre aire de santé il y a une structure ici, un CH environs 300m qui était obligé de fermer.

**P32: IA_02_HGR_DN.rtf - 32:19 [Répondant : Non c’est le BCZS ..] (42:42)	(Super)**

Codes:	[Structure-Service_Fermeture_pdt MVE]

No memos

**Répondant** : Non c’est le BCZS qui avait proposé qu’on puisse fermer par ce que c’est une structure qui gardé longtemps un cas confirmé et on ne voulait pas que l’équipe de la riposte puisse venir investiguer. C’est à la fin qu’on a constaté un décès et quand on a swappé c’était positif. Maintenant tous les malades y étaient internés et avec ce cas de décès ils avaient développé la MVE à cause de ce cas. C’est une structure qui changeait les malades comme elle avait une autre structure dans la zone de sante de Katwa lorsqu’elle constatait que l’équipe de riposte a suspecté un malade la nuit on changeait le malade vers l’autre structure dans l’autre zone de santé comme nous sommes deux zones voisines. C’est ainsi que dans les deux zones on a fermé leurs structures. Il y a eu beaucoup de structures qui étaient fermées même dans l’aire de santé de Vulamba qui avait une structure où on avait écrit une lettre de fermeture mais cette dernière n’avait pas appliqué la décision hiérarchique Comme le responsable c’est un incivique on a peur de lui, la structure est restée opérationnelle.

**P32: IA_02_HGR_DN.rtf - 32:20 [Répondant : Dans notre structu..] (44:44)	(Super)**

Codes:	[Structures-Services_fonctionnement]

No memos

**Répondant** : Dans notre structure nous avons eu la grâce de Dieu, au début de l’épidémie nous pouvons remercier l’ONG IRC qui donné la formation lors de la déclaration d’un cas de MVE à Beni à propos de la PCI. Tous les personnels ont bénéficié ladite formation, c’est un point fort pour notre structure. Pendant l’épidémie nous n’avons jamais eu un cas positif, en suite les accoucheuses ont appliqué les précautions universelles et le port des EPI étaient systématiques il n’y avait pas de problème même si les accouchements ont diminué à cause de l’épidémie par ce qu’avant l’épidémie on dirigeait cent à cent – vingt et même cent cinquante (100-150) accouchements le mois. Ce qui a été très remarquable pendant l’épidémie parce que la moyenne a été de quatre-vingt accouchement par mois. On n’a pas eu de de problème des infections nosocomiales pendant l’épidémie à la maternité. Sauf qu’en chirurgie pendant le mois de décembre on avait eu un cas de contact qui avait fui dans l’aire de santé de Matanda, il est parti dans la zone de santé de Katwa, après il avait fui da la zone de santé de Vuhovi et après il est revenu vers 17hoo dans un tableau d’un abdomen aigue. C’est celui-là qui voulait nous créer de problème par ce qu’on l’avait fait entrer aux soins intensifs, vu son état de choc ; on avait commencé à lui réanimer en plaçant le sonde urétral, sonde nasogastrique, une heure après il est décédé. C’est celui-là qu’on préparait pour une éventuelle intervention. Après le swape c’était positif et comme c’était un évadé connu qui était dans la base, on s’est retrouvé avec un cas confirmé dans la structure et on n’avait pas compris comment il était passé au triage.

**P32: IA_02_HGR_DN.rtf - 32:21 [Répondant : Je dirais non, not..] (46:46)	(Super)**

Codes:	[Structure-Service_Utilisation_population]

No memos

**Répondant** : Je dirais non, notre population depuis qu’on a instauré le système de triage elle n’arrive pas à cause de ce triage. Elle a peur du triage et cela avait pris du temps par ce qu’au début elle ne voulait pas passer par le triage ; même le lavage de main était difficile parce qu’elles disaient que cette eau contenait le virus d’Ebola. Plus tard elle avait compris qu’il fallait y passer toujours.

**P32: IA_02_HGR_DN.rtf - 32:22 [Répondant : Ils disaient que c..] (48:48)	(Super)**

Codes:	[Triage_Considération_population]

No memos

**Répondant** : Ils disaient que c’était la MVE, que nous voulons injecter le virus en se lavant les mains.

**P32: IA_02_HGR_DN.rtf - 32:23 [Répondant : C’était à cause du..] (50:50)	(Super)**

Codes:	[Triage-Isolement-Chlore-thermoflash_Réaction_population]

No memos

**Répondant** : C’était à cause du chlore qui était dans l’eau mais quand on a changé le protocole avec le savon il n’y a plus de problème.

**P32: IA_02_HGR_DN.rtf - 32:24 [Répondant : Pour eux pensaient..] (52:52)	(Super)**

Codes:	[Triage-Isolement-Chlore-thermoflash_Réaction_population]

No memos

**Répondant** : Pour eux pensaient que c’est ce qui contenait le virus de l’épidémie.

**P32: IA_02_HGR_DN.rtf - 32:25 [Répondant : Ils avaient peur d..] (54:54)	(Super)**

Codes:	[Triage-Isolement-Chlore-thermoflash_Réaction_population]

No memos

**Répondant** : Ils avaient peur de passer dans la maisonnette alors que c’est le circuit normal pour passer que tu sois visiteur ou patient tu dois passer au triage. C’est ce qu’eux ne voulaient pas. C’est ainsi que les structures privées ont été fréquentés par rapport aux structure publiques.

**P32: IA_02_HGR_DN.rtf - 32:26 [Répondant : Oui certaines stru..] (56:56)	(Super)**

Codes:	[Triage-Isolement-Chlore-thermoflash_Réaction_population]

No memos

**Répondant** : Oui certaines structures n’avaient pas de triage, d’autres en avaient mais ils ne respectaient pas les normes du triage.

**P32: IA_02_HGR_DN.rtf - 32:27 [Répondant : Oui ce le triage q..] (58:58)	(Super)**

Codes:	[Triage-Isolement-Chlore-thermoflash_Réaction_population]

No memos

**Répondant** : Oui ce le triage qui a posé problème pour la fréquentation.

**P32: IA_02_HGR_DN.rtf - 32:28 [Répondant : Oui donc les struc..] (60:60)	(Super)**

Codes:	[Structure-Service_Utilisation_Alternatives_population]

No memos

**Répondant** : Oui donc les structures qui ne sont pas normés, il des Postes de santé.

**P32: IA_02_HGR_DN.rtf - 32:29 [Répondant : C’est vrai, les ma..] (62:62)	(Super)**

Codes:	[Structure-Service_Utilisation_Alternatives_population]

No memos

**Répondant** : C’est vrai, les malades fréquentaient les tradi-praticiens et nous avons eu un tradi qui notifiait les cas confirmés de la MVE.

**P32: IA_02_HGR_DN.rtf - 32:30 [Répondant : Ils faisaient l’au..] (64:64)	(Super)**

Codes:	[Structure-Service_Utilisation_Alternatives_population]

No memos

**Répondant** : Ils faisaient l’automédication, d’une manière générale notre population utilisaient plus les pharmacies pendant l’épidémie, ils ne venaient pas aux soins et une fois il y a gravité de maladie ils pouvaient aller dans une structure qui n’est pas normée soit une structure traditionnelle.

**P32: IA_02_HGR_DN.rtf - 32:31 [Répondant : Par rapport à la c..] (66:66)	(Super)**

Codes:	[Qualité_soins_Prestataires]

No memos

**Répondant** : Par rapport à la communication entre les prestataires et les malades, même nos centres de santé normés n’ont pas été fréquenté, ils avaient le même problème que nous. Seulement comme il n’avait pas un engagement communautaire, on n’avait pas fait allusion à nos relais communautaires de la place et comme il n’y avait pas les CAC qui était formés récemment mais s’il y avait un engagement communautaire on ne devrait pas avoir les mêmes problèmes par ce qu’il suffisait de faire une sensibilisation sur la MVE il y a un terme qu’on utilisait « ULISHA MEZA » tu as déjà bouffé l’argent pour leurs sacrifier. Maintenant il y a les prestataires qui se réservaient de ne pas communiquer avec la population, ce qui m’inquiète et étonnante même dans les églises il suffisait qu’un prêtre ou un pasteur parle de la MVE les fidèles disent que lui aussi a déjà pris quelques choses pour nous sacrifier.

**P32: IA_02_HGR_DN.rtf - 32:32 [Répondant : Le quelque chose s..] (68:68)	(Super)**

Codes:	[Qualité_soins_Prestataires]

No memos

**Répondant** : Le quelque chose signifier en d’autres termes tu es déjà corrompu par l’équipe de la riposte, on t’a donné quelque chose. C’est ça le problème vécu pendant la période de l’épidémie.

**P32: IA_02_HGR_DN.rtf - 32:33 [utilisait « ULISHA MEZA » tu a..] (66:66)	(Super)**

Codes:	[Considération_prestataire_population]

No memos

utilisait « ULISHA MEZA » tu as déjà bouffé l’argent pour leurs sacrifier. Maintenant il y a les prestataires qui se réservaient de ne pas communiquer avec la population, ce qui m’inquiète et étonnante même dans les églises il suffisait qu’un prêtre ou un pasteur parle de la MVE les fidèles disent que lui aussi a déjà pris quelques choses pour nous sacrifier.

**P32: IA_02_HGR_DN.rtf - 32:34 [Répondant : C’est ce qui avait..] (72:72)	(Super)**

Codes:	[Considération_prestataire_population]

No memos

**Répondant** : C’est ce qui avait fait que les prestataires puissent se taire de ne pas communiquer et c’est ce qui avait causé un problème. Comme la population avait confiance envers nous et que nous, nous ne voulons pas communiquer c’est ainsi que la population puisait les informations au réseaux sociaux alors que n’importe qui pouvait écrire sur les réseaux sociaux.

**P32: IA_02_HGR_DN.rtf - 32:35 [Répondant : Non il n’y a pas a..] (74:74)	(Super)**

Codes:	[Qualité_soins_Prestataires]

No memos

**Répondant** : Non il n’y a pas autres choses.

**P32: IA_02_HGR_DN.rtf - 32:36 [Répondant : Au début on avait ..] (76:76)	(Super)**

Codes:	[Traitement_Invasifs_Prestataire_changement]

No memos

**Répondant** : Au début on avait peur ça c’est vrai même a la réception de malade c’est ainsi que pendant l’épidémie il avait un problème d’accueil, un malade vous arrive dans un état critique si vous ne portez pas d’EPI il faut aller le porter d’abord on fait 15 à 20 minutes et ceux qui l’amènent vont se lamenter contre vous et cela qui était la base d’un conflit entre prestataire et malade. Puisqu’il fallait qu’on se préparer pour transporter le malade, pour lui faire un siège. Donc l’accueil a posé un problème et il fallait se protéger aussi et on ne pouvait pas toucher un malade sans porter des EPI. Pour faire comprendre cela à la population c’est ce qui avait causé problème. C’est comme ça que la population ne voulait pas fréquenter nos structures à cause de cette rigueur. Par ce que dans un poste de santé privé un malade arrive on le reçoit et dans trente minutes on aura tout fini. Mais dans une structure normée on doit faire le triage, on doit chequer et après on peut examiner et ça créer un problème.

**P32: IA_02_HGR_DN.rtf - 32:37 [Répondant : Oui exactement une..] (78:78)	(Super)**

Codes:	[Ressources_humaines_situation_grève]

No memos

**Répondant** : Oui exactement une grève administrative des infirmiers, c’était de janvier jusqu’en avril 2018 suite à la non considération des infirmiers et certains d’eux n’étaient pas alignés sur le listing de la prime de risque. Parfois li y avait omission de certains et lorsqu’on est omis c’est fini.

**P32: IA_02_HGR_DN.rtf - 32:38 [Répondant : C’est avec la décl..] (80:80)	(Super)**

Codes:	[Ressources_humaines_situation_grève]

No memos

**Répondant** : C’est avec la déclaration de l’épidémie qu’on était obligé de lever la grève, comme il y avait la grève de février en avril il y avait la grève administrative et en juillet il y avait une autre grève que je ne sais pas sa modalité.

**P32: IA_02_HGR_DN.rtf - 32:39 [Répondant : Oui, oui on était ..] (82:82)	(Super)**

Codes:	[Ressources_humaines_situation_grève]

No memos

**Répondant** : Oui, oui on était toujours en grève et quand on avait déclaré l’épidémie on était obligé de suspendre la grève.

**P32: IA_02_HGR_DN.rtf - 32:40 [Répondant : Cette gratuité, le..] (84:84)	(Super)**

Codes:	[Gratuité_Structures-Services_concernés]

No memos

**Répondant** : Cette gratuité, le premier contrat était une gratuité globale c’était en octobre 2018 et ç’a pris trois mois et là nous avons constaté un engouement total de malades dans nos structures donc ici chez nous on payait les consultations externes, l’admission en médecine interne, la chirurgie, la gynécologie en tout cas c’était global, après on avait modifié le contrat on avait enlevé les consultations externes, on avait retenu l’admission en médecine interne pour les maladies courantes, la pédiatrie et les malades qui viennent du CTE les sortis non cas.Aujourd4hui c’est presque la même chose.

**P32: IA_02_HGR_DN.rtf - 32:41 [Répondant : Pour la gratuité g..] (86:86)	(Super)**

Codes:	[Gratuité_Rumeurs]

No memos

**Répondant** : Pour la gratuité globale il n’y avait pas de soucis, la population était contente de cette dernière, et maintenant comme c’est une gratuité partielle dirais-je comme ça prend quelques services c’est là que réside le problème, vous pouvez être dans une même chambre l’un a la maladie chronique et l’autre a une maladie courante mais l’un paie et l’autre ne paie pas. Maintenant ça crée de soucis dans la communauté, si on pouvait avoir un autre partenaire qui peut subvenir sur d’autre services ça peut nous soulager.

**P32: IA_02_HGR_DN.rtf - 32:42 [Répondant : Oui, c’est connu.] (88:88)	(Super)**

Codes:	[Gratuité_Information_population]

No memos

**Répondant** : Oui, c’est connu.

**P32: IA_02_HGR_DN.rtf - 32:43 [Répondant : Pendant la riposte..] (92:92)	(Super)**

Codes:	[Médicaments_disponibilité_fosa]

No memos

**Répondant** : Pendant la riposte, pour les intrants du PCI nous avons notre partenaire IRC qui nous dote des intrants pour les médicaments dans notre contrat il y a le 20% qui devrait rester

**P32: IA_02_HGR_DN.rtf - 32:44 [Répondant : Exactement puis qu..] (94:94)	(Super)**

Codes:	[Médicaments_disponibilité_fosa]

No memos

**Répondant** : Exactement puis qu’il y avait un engouement des malades.

**P32: IA_02_HGR_DN.rtf - 32:45 [Répondant : Le système de rapp..] (96:96)	(Super)**

Codes:	[SNIS_fonctionnement_Decrire_pdt MVE]

No memos

**Répondant** : Le système de rapportage des données dépend d’une personne à une autre, ici au niveau de la structure nous n’avons pas connu nous on remettait notre rapport chaque le 6 du mois au BCZS que ça soit le Mapipi et ce dernier c’est chaque lundi au plus tard 12hoo.

**P32: IA_02_HGR_DN.rtf - 32:46 [Répondant : Nous avons de prob..] (98:98)	(Super)**

Codes:	[Réunion_pdt-épidémie-Ebola]

No memos

**Répondant** : Nous avons de problème de la tenue de nos réunions par rapport à nos indicateurs de qualité. Par exemple pour le comité de mot vigilance, si c’est prévu une réunion par mois on pouvait faire deux mois sans tenir réunion raison de la non permanence des acteurs, c’est là que réside le problème des Médecins.

**P32: IA_02_HGR_DN.rtf - 32:47 [épondant : C’est toujours l’en..] (100:100)	(Super)**

Codes:	[Souhait_Changement_gestion_futures_épidémies]

No memos

**épondant** : C’est toujours l’engagement communautaire, le reste peut aller de soi.

**P32: IA_02_HGR_DN.rtf - 32:48 [Répondant : Lorsque je parle d..] (102:102)	(Super)**

Codes:	[Souhait_Changement_gestion_futures_épidémies]

No memos

**Répondant** : Lorsque je parle de l’engagement communautaire veut dire qu’on doit planifier avec la communauté car on ne peut pas mener une action communautaire sans l’avis de la communauté.

**P32: IA_02_HGR_DN.rtf - 32:49 [Répondant : Non, non il fallai..] (104:104)	(Super)**

Codes:	[Considération_prestataire_population]

No memos

**Répondant** : Non, non il fallait réunir la communauté par ce qu’il y a ceux qui sont censé de parler avec la communauté ; deuxième chose dans notre système de santé il faut qu’on considère les infirmiers, si les gens ont fui les structures à cause de ça. On travaille 50ans,20ans,30ans sans même le numéro matricule sans même être aligné sur la liste de prime de risque. Lorsque tu trouves là où on va te donner de l’avantage tu vas fouir pour aller là où on te propose mieux. C’est ainsi que nos structures sont vides, il faut que notre gouvernement pense aux infirmiers.

**P32: IA_02_HGR_DN.rtf - 32:50 [Répondant : Il n’y a plus d’au..] (106:106)	(Super)**

Codes:	[Commentaire-Recommandation_Répondant]

No memos

**Répondant** : Il n’y a plus d’autres seulement, s’il y a une épidémie qu’on puisse impliquer tous les acteurs.

**P33: IA_02_HGR_PSYCHO.rtf - 33:1 [Répondant : On enseigne, on ap..] (6:6)	(Super)**

Codes:	[Répondant_Responsabilité_habituelles]

No memos

**Répondant** : On enseigne, on apprend les gens d’abord les médecins, vers le matin, on prie pour le travail. Et après, l’aumônier passe local par local pour une conversation avec les malades, et ensuite une petite prière pour ces derniers. Et si les médecins constatent des malades qui ont des problèmes spirituels, on nous appelle.

**P33: IA_02_HGR_PSYCHO.rtf - 33:2 [Répondant : il y’a ceux qui on..] (8:8)	(Super)**

Codes:	[Répondant_Responsabilité_habituelles]

No memos

**Répondant** : il y’a ceux qui ont des problèmes familials, l’aumônier discute avec ces malades, il cherche à savoir s’il a un problème entre le malade et ses parents, ils le réunissent et cherche comment résoudre ce problème.

**P33: IA_02_HGR_PSYCHO.rtf - 33:3 [Répondant : Et si le prestatai..] (10:10)	(Super)**

Codes:	[Répondant_Responsabilité_habituelles]

No memos

**Répondant** : Et si le prestataire de service a aussi de problème, soit s’il y’a des mésententes entre le prestataire et le malade, entre deux prestataires, l’aumônier cherche ce prestataire et il voit comment résoudre ce problème.

**P33: IA_02_HGR_PSYCHO.rtf - 33:4 [Répondant : Cette période n’a ..] (12:12)	(Super)**

Codes:	[Répondant_Responsabilité_pdt MVE]

No memos

**Répondant** : Cette période n’a pas été facile, et surtout que la population n’avait pas admis cette épidémie. Et quand les médecins voulaient transférer un malade qui présentait les signes, ça devenait un conflit avec les malades. C’est alors qu’on m’appelait pour parler à la famille du malade ainsi qu’aux autres malades. Et je le montrais que le transfert ce n’est pas pour tuer le malade, mais pour l’aider à s’en sortir. En bref, c’était ça mon travail pendant cette période.

**P33: IA_02_HGR_PSYCHO.rtf - 33:5 [Répondant : Quand on a appris ..] (14:14)	(Super)**

Codes:	[Considération_Ebola_population]

No memos

**Répondant** : Quand on a appris l’existence de l’épidémie a MANGINA, les gens ont eu très peur, et c’est arrivé ici à BUTEMBO, les gens ont commencé à porter les gans, mais comme c’était pendant la période des campagnes électorale, un monsieur est venu de KINSHASA et disait à la population que cette épidémie n’existe pas, et c’est là où la réticence a commencé.

**P33: IA_02_HGR_PSYCHO.rtf - 33:6 [Répondant : Il a de ces centre..] (16:16)	(Super)**

Codes:	[Structure-Service_Fermeture_pdt MVE]

No memos

**Répondant** : Il a de ces centres de santé qui fonctionnaient difficilement, d’autres ont été brulés, on insultait les prestataires de services de santé, il y’a aussi des centres de santé qui ne fonctionnaient que pendant la journée et fermaient la nuit. Ici à l’hôpital, on fonctionnait, mais le service de vaccin n’était pas fréquent, alors on avait fermé ce service, sont rare les gens qui venaient à l’hôpital. Il y’a ceux qui refusaient la gratuite, et d’autres l’acceptaient. Mais pendant cette gratuite, beaucoup des gens sont venus à l’hôpital.

**P33: IA_02_HGR_PSYCHO.rtf - 33:7 [Répondant : Ils croyaient que ..] (18:18)	(Super)**

Codes:	[Gratuité_Rumeurs]

No memos

**Répondant** : Ils croyaient que c’étaient des médicaments pour le tuer, ils disaient que ces médicaments provenaient du pays voisin (le Rwanda), et ils viennent tuer tout le monde avec ce médicament. Mais ceux qui étaient d’accord le prenaient et ils bénéficiaient de ces médicaments.

**P33: IA_02_HGR_PSYCHO.rtf - 33:8 [Répondant : Ceux qui venaient ..] (20:20)	(Super)**

Codes:	[Structures-Services_fonctionnement]

No memos

**Répondant** : Ceux qui venaient au CPN, sont venus malgré que leur effectif a baissé, les femmes mettaient au monde jusqu’à ce que les rimeurs commencent en disant l’accouchement, les femmes sont transférées au CTE pour y passer d’abord le test, mais ceux qui venaient, mettaient au monde, malgré que ces rumeurs ont baissé l’effectif.

**P33: IA_02_HGR_PSYCHO.rtf - 33:9 [Répondant : Quand on a découve..] (22:22)	(Super)**

Codes:	[Prestataire_Pratique de Prévention contre MVE]

No memos

**Répondant** : Quand on a découvert l’épidémie, l’IRS est venu pour former les prestataires de service à propos du PCI qui veut dire protection et contrôle individuel, on a formé presque tous les personnels de la santé, pour toucher un malade il fallait porter la tenue.

**P33: IA_02_HGR_PSYCHO.rtf - 33:10 [Répondant : Au début, on nous ..] (24:24)	(Super)**

Codes:	[Prestataire_Attitude_devant_malade]

No memos

**Répondant** : Au début, on nous avait conseillé de ne pas se précipiter, il fallait se protéger avant de soigne un malade. C’est à la fin que tous ont mal tourné vu que certains étaient bénéficiaires et d’autres non.

**P33: IA_02_HGR_PSYCHO.rtf - 33:11 [Répondant : Cette qualité n’a ..] (26:26)	(Super)**

Codes:	[Qualité_Soins_Patients]

No memos

**Répondant** : Cette qualité n’a pas changé, elle est restée la même.

**P33: IA_02_HGR_PSYCHO.rtf - 33:12 [Répondant : En ce qui concerne..] (28:28)	(Super)**

Codes:	[Qualité_Soins_Patients]

No memos

**Répondant** : En ce qui concerne l’accueil, les malades passaient d’abord par le triage avant d’être orientés dans les services.

**P33: IA_02_HGR_PSYCHO.rtf - 33:13 [Répondant : Nous on avait de l..] (30:30)	(Super)**

Codes:	[Ressources_humaines_Disponibilité et perturbations]

No memos

**Répondant** : Nous on avait de la chance, on avait des médecins stagiaires, on avait pris trois médecins en training, ils ont travaillé, et jusqu’à maintenant ils continuent à travailler.

**P33: IA_02_HGR_PSYCHO.rtf - 33:14 [Répondant : Le problème résida..] (32:32)	(Super)**

Codes:	[Ressources_humaines_Disponibilité et perturbations]

No memos

**Répondant** : Le problème résidait au niveau de l’élaboration de l’horaire, il y’a de ceux-là qui travaillaient à la fois au CTE et ici, alors quand ils étaient au CTE il fallait lui chercher un remplaçant.

**P33: IA_02_HGR_PSYCHO.rtf - 33:15 [Répondant : Non, parce qu’on a..] (34:34)	(Super)**

Codes:	[Ressources_humaines_solution-local_Disponibilité et perturbations]

No memos

**Répondant** : Non, parce qu’on avait des remplaçants pour ces gens qui étaient à la riposte.

**P33: IA_02_HGR_PSYCHO.rtf - 33:16 [Répondant : La population étai..] (36:36)	(Super)**

Codes:	[Structure-Service_Utilisation_population]

No memos

**Répondant** : La population était déjà divisée, il y’a ceux qui venaient prendre les soins dans des structures bien connus, et d’autres refusaient et allaient chercher les soins quelque part. et là où ils allaient c’est là où on enregistrer beaucoup des cas.

**P33: IA_02_HGR_PSYCHO.rtf - 33:17 [Répondant : Ils avaient peur d..] (38:38)	(Super)**

Codes:	[Considération_CTE_population] [Structure-Service_Utilisation_Raisons fuites FOSA publique_population]

No memos

**Répondant** : Ils avaient peur d’aller au CTE, ils croyaient les gens qui y allaient, devraient nécessairement mourir.

**P33: IA_02_HGR_PSYCHO.rtf - 33:18 [Répondant : Beaucoup des gens ..] (40:40)	(Super)**

Codes:	[Structure-Service_Utilisation_Alternatives_population]

No memos

**Répondant** : Beaucoup des gens ont fait recours à ce dernier, et c’est quand ça ne fonctionnait pas là, qu’ils venaient alors à l’hôpital.

**P33: IA_02_HGR_PSYCHO.rtf - 33:19 [Répondant : Beaucoup sont venu..] (42:42)	(Super)**

Codes:	[Structure-Service_Utilisation_Alternatives_population]

No memos

**Répondant** : Beaucoup sont venu à l’hôpital, mais d’autres allaient là.

**P33: IA_02_HGR_PSYCHO.rtf - 33:20 [Répondant : Leur travail était..] (44:44)	(Super)**

Codes:	[Ressources_humaines_conditions_travail]

No memos

**Répondant** : Leur travail était devenu difficile, car on les insultait à cause de ce monsieur qui avait au début que les médecins étaient corrompus.

**P33: IA_02_HGR_PSYCHO.rtf - 33:21 [Répondant : La gratuite a comm..] (46:46)	(Super)**

Codes:	[Gratuité_Début] [Gratuité_Structures-Services_concernés]

No memos

**Répondant** : La gratuite a commencé en octobre, puis novembre et jusqu’au mi-janvier, ça implique la consultation externe, la médecine interne, tous ceux qui n’avait pas d’argent pour se faire soigner, ont profité pendant cette gratuite,

**P33: IA_02_HGR_PSYCHO.rtf - 33:22 [Répondant : La gratuite avait ..] (48:48)	(Super)**

Codes:	[Gratuité_Evolution]

No memos

**Répondant** : La gratuite avait des étapes.

**P33: IA_02_HGR_PSYCHO.rtf - 33:23 [Répondant : pour la première g..] (50:50)	(Super)**

Codes:	[Gratuité_Evolution]

No memos

**Répondant** : pour la première gratuite, beaucoup des gens ont profité, surtout ceux de de l’opération. La deuxième étape c’était uniquement la médecine interne et la pédiatrie qui sont reste.

**P33: IA_02_HGR_PSYCHO.rtf - 33:24 [Répondant : pendant la gratuit..] (52:52)	(Super)**

Codes:	[Gratuité_Conséquences_motivation_prestataire]

No memos

**Répondant** : pendant la gratuite, il n’avait pas de recettes locales, ils envoyé l’argent a la banque dépendamment du travail que vous avez effectué.

**P33: IA_02_HGR_PSYCHO.rtf - 33:25 [Répondant : A partir de septem..] (54:54)	(Super)**

Codes:	[Gratuité_Conséquences_motivation_prestataire]

No memos

**Répondant** : A partir de septembre, on a plus trouvé l’argent de gratuite et cela à cause des troubles au niveau des prestataires.

**P33: IA_02_HGR_PSYCHO.rtf - 33:26 [Répondant : Avant l’épidémie, ..] (56:56)	(Super)**

Codes:	[Médicaments_disponibilité_fosa]

No memos

**Répondant** : Avant l’épidémie, quand il avait des recettes, il avait un pourcentage pour la pharmacie, et c’est à partir de ce pourcentage qu’on achetait les médicaments. Pendant la gratuite, ils avaient l’argent qu’on recevait en deux partie, une partie pour la prime des personnels, l’autre pour le fonctionnement, la pharmacie et autres. On devait acheter les médicaments uniquement chez ASRAMESS, c’est ainsi qu’ASRAMESS n’a pas respecté les réquisitions.

**P33: IA_02_HGR_PSYCHO.rtf - 33:27 [Répondant : C’est parce qu’ils..] (58:58)	(Super)**

Codes:	[Médicaments_disponibilité_fosa]

No memos

**Répondant** : C’est parce qu’ils n’avaient pas des médicaments que nous on voulait, c’est alors qu’ils nous donnaient ce qu’eux voulaient.

**P33: IA_02_HGR_PSYCHO.rtf - 33:28 [Répondant : On a beaucoup cons..] (60:60)	(Super)**

Codes:	[Médicaments_disponibilité_fosa]

No memos

**Répondant** : On a beaucoup consommé, il y’a de fois où l’on prenait des médicaments dans notre stock.

**P33: IA_02_HGR_PSYCHO.rtf - 33:29 [Répondant : Le CTS avait dit q..] (62:62)	(Super)**

Codes:	[Gratuité_Structures-Services_concernés]

No memos

**Répondant** : Le CTS avait dit que qu’on devrait payer pour la transfusion, c’est alors les malades qui nécessitaient la transfusion devraient payer.

**P33: IA_02_HGR_PSYCHO.rtf - 33:30 [Répondant : Le sang était disp..] (64:64)	(Super)**

Codes:	[Médicaments_disponibilité_fosa]

No memos

**Répondant** : Le sang était disponible, malgré les groupes sanguins de gens, le sang était toujours disponible.

**P33: IA_02_HGR_PSYCHO.rtf - 33:31 [Répondant : Ça n’a pas eu vrai..] (66:66)	(Super)**

Codes:	[Gouvernance_soins santé]

No memos

**Répondant** : Ça n’a pas eu vraiment d’impact, car on faisait des différentes réunions, les personnels se réunissaient chaque lundi pour l’évaluation du PCI. C’est au niveau du CODIR, conseil de direction, ou l’on se réunissaient rarement par manque de temps, il avait le staff médical, le staff infirmière qui se réunissaient aussi chaque semaine.

**P33: IA_02_HGR_PSYCHO.rtf - 33:32 [Répondant : A cause du manque ..] (68:68)	(Super)**

Codes:	[Gouvernance_soins santé]

No memos

**Répondant** : A cause du manque du temps, les décisions n’étaient pas respectées.

**P33: IA_02_HGR_PSYCHO.rtf - 33:33 [Répondant : La première périod..] (70:70)	(Super)**

Codes:	[Gratuité_Evolution]

No memos

**Répondant** : La première période s’est terminée le 15 janvier, les gens ont payés en février 2019, en aout 2019, et de janvier 2020 jusqu’au 15 février 2020 et on a encore repris la gratuite jusqu’à maintenant.

**P33: IA_02_HGR_PSYCHO.rtf - 33:34 [Répondant : La première des ch..] (72:72)	(Super)**

Codes:	[Souhait_Changement_gestion_futures_épidémies]

No memos

**Répondant** : La première des choses, il faut recruter les gens connus dans le milieu affecte par l’épidémie, on le forme, car ce sont eux qui maitrisent la mentalité de la population. Mais quand on voie ce sont étrangers qui font la sensibilisation, alors qu’ils ne maitrisent même pas la langue ça créait de réticence. Il ne faut pas trop faire peur à la population. Il faut aussi choisir les sensibilisateurs parmi les habitants du milieu affecte. La population avait remarqué qu’il a trop des étrangers, et ceux qui étaient sélectionnés parmi la population n’étaient même pas des agents de la sante. Autre chose, le vaccin, beaucoup ne l’ont pas admis, et ceux qui voulaient être vaccinés n’arrivaient à être vaccinés vu l’indisponibilité du vaccin.

**P33: IA_02_HGR_PSYCHO.rtf - 33:35 [Répondant : Il faut d’abord fa..] (74:74)	(Super)**

Codes:	[Autres_Problèmes observés]

No memos

**Répondant** : Il faut d’abord faire comprendre aux gens la situation, et c’est en faisant cela que les gens peuvent l’accepter, aussi le problème était quand il avait beaucoup des décès, les gens croyaient qu’on le tuait alors que les malades arrivaient tardivement au centre.

**P33: IA_02_HGR_PSYCHO.rtf - 33:36 [Répondant : Je vous remercie p..] (76:76)	(Super)**

Codes:	[Commentaire-Recommandation_Répondant]

No memos

**Répondant** : Je vous remercie pour ce temps que nous venons de s’entretenir à propos de l’Ebola, je crois que si ça revient un autre jour, ou quelque part d’autre, les choses se passeront bien.

**P34: IA_03_DPS_ANALYSTE_01.rtf - 34:1 [Répondant : Merci pour la ques..] (6:6)	(Super)**

Codes:	[Répondant_Responsabilité_habituelles]

No memos

**Répondant** : Merci pour la question, depuis un certain temps la République a ramené sur le plan santé la reforme au niveau national qu’intermédiaire et qu’au niveau intermédiaire depuis un temps les bureaux ont été restructurés. Et dans les six bureaux il y a le bureau d’information sanitaire, c’est le bureau dans lequel je travaille. Ce bureau a comme objectif principal qui s’occupe des données, la gestion de donné de santé dans l’ensemble des provinces. Voilà globalement ce qu’on fait, il s’occupe également des maladies potentielles, épidémiologiques et épidémique, il s’occupe également de la communication et aussi un volet important la recherche. Voilà globalement et c’est comme ça qu’on dit « le Bureau d’information sanitaire, communication et Recherche ». Et dans ce bureau je suis en train d’occuper par ce que lors de la reforme il y a un collègue qui est parti et depuis qu’il est parti nous avons pris la relève et nous nous occupons de la gestion de données de l’ensemble de la province, comme à la liste chargée de gestion des données dans la province.

**P34: IA_03_DPS_ANALYSTE_01.rtf - 34:2 [Répondant : Quand la riposte a..] (8:8)	(Super)**

Codes:	[Répondant_Responsabilité_pdt MVE]

No memos

**Répondant** : Quand la riposte a commencé, il faut dire que c’est un évènement en termes de santé ; un évènement qui capte l’attention, le système était ébranlé, par ce que tout le monde se jetais comme c’était la première expérience au niveau de la province, on ne savait pas comment commencer alors nous nous sommes jeté là-dessous pour que nous puissions aider. Alors dire que mes activités ont été bouleversées pas tellement par ce qu’après le système a été tel qu’il y a eu de coordination et cette dernière s’occupait de la riposte, néanmoins il y avait des données qui arrivaient au niveau de la province auxquelles nous étions obligés de les réorganiser pour que lors de la réunion parce que chaque jour des réunions se tenaient, nous avion l’information pour que lors de la réunion nous puissions la donner. Quand bien même c’est toujours la coordination et leurs équipes qui s’en occupaient en terme des données.

**P34: IA_03_DPS_ANALYSTE_01.rtf - 34:3 [Répondant : Moi personnellemen..] (10:10)	(Super)**

Codes:	[Répondant_Responsabilité_pdt MVE]

No memos

**Répondant** : Moi personnellement non mais à un moment lorsque l’épidémie prenait de l’ampleur et que ça débordait au niveau de Beni, son excellence monsieur le ministre avait proposer qu’il ait encrage, ça signifie qu’il considérait lui à ce moment-là que l’épidémie d’Ebola n’était plus un problème national par ce qu’au début quand ils sont venus, ils nous disaient que c’est un problème national Et que nous nous ne pouvions pas nous ingérer dans ça par ce que c’est un problème national, donc l’équipe nationale s’occupait de tous. A partir de Décembre 2018 quand il a vu que ça prenne de l’ampleur il a pensé que la division prenne en charge la riposte, c’est à ce moment-là qu’il nous attribua le bureau, nous devions nous occuper de la commission, surveillance et épidémiologie, Novembre, Décembre, Janvier 2019 nous avons impliqué jusqu’en février et après on s’est rendu compte que le même système continuait et nous nous sommes encore désengagé pour laisser le système au niveau national

**P34: IA_03_DPS_ANALYSTE_01.rtf - 34:4 [Répondant : Lorsque nous étion..] (12:12)	(Super)**

Codes:	[Répondant_Responsabilité_pdt MVE_Depuis quand ?]

No memos

**Répondant** : Lorsque nous étions impliqués, nous nous occupions de la centralisation de toutes les données au niveau de la coordination, les équipes de terrain devaient nous ramener les données et nous nous devrions nous occuper de la base des données.

**P34: IA_03_DPS_ANALYSTE_01.rtf - 34:5 [Répondant : Je peux probableme..] (14:14)	(Super)**

Codes:	[SNIS_fonctionnement_Decrire_pdt MVE]

No memos

**Répondant** : Je peux probablement me retrouver par ce qu’il y a eu beaucoup d’évènements qui se sont passés pendant l’épidémie, pour nous nous avons pensé qui n’y a pas eu beaucoup d’implication. La seule chose que je peux dire, notre bureau a connu de secousse ; le chef de bureau a eu un problème, l’analyste qui est chargé de surveillance épidémiologique est parti dans Ebola, l’analyste qui devait se charger des données est parti, il a eu une affectation ailleurs du coup au niveau du bureau il ne restait que le chef du bureau ad intérim et donc pour lui seul travaillait ça devenait compliqué, c’est ainsi que le Chef de division a rapidement comblé en nous mettant là-bas pour qu’en deux nous puissions travailler. Comme nous faisions avant, je ne pas senti que système était secoué par ce que nous avons un data que chaque semaine les zones de santé nous communique les données, et donc nous étions à par semaine épidémiologie nous avions toujours nos informations. C’est vrai que nous avons eu de problème au niveau des zones de santé, par ce qu’au niveau des zones de santé les équipes cadres étaient totalement plongé dans les activités de la riposte et donc du cout et cela que je me retrouve que le système a été embrouillé par que le MCZS et son équipe étaient totalement pris dans la riposte, pour avoir les données et la division cela qu’elle a empathie, et donc les données devenaient un problème pour avoir et tout rapport qu’on demandait ça ne faisait pas bon par ce que tout le temps ils étaient là-bas, nous étions en difficulté qu’est-ce qu’on fait à ce moment-là. On martelait mais ç’a eu un cout par rapport au remonté des informations.

**P34: IA_03_DPS_ANALYSTE_01.rtf - 34:6 [Répondant : Le tout ; quand la..] (16:16)	(Super)**

Codes:	[SNIS_fonctionnement_Decrire_pdt MVE]

No memos

**Répondant** : Le tout ; quand la complétude pose problème déjà reste sera impacté, ça ne permettait pas même de décider. Voyez-vous on avait la complétude de moins de 70%, les données que nous avions ne permettaient pas d’analyser. Et comme il n’avait plus la promptitude ce qui justifie qu’il y avait le retard dans la prise des décisions. Pendant l’épidémie d’Ebola nous avions l’épidémie de rougeole aussi mais on ne s’est mêmes pas aperçue qu’il y avait l’épidémie de rougeole par ce que tout le monde était focalisé dans l’Ebola. C’est plus tard que nous nous sommes rendu compte que l’épidémie de rougeole était là et même l’épidémie du choléra. On a été giflé par des morts qui venaient déjà et tous étaient focalisés vers Ebola alors qu’il y avait la Rougeole et le choléra au même moment et d’ailleurs le choléra et Rougeole continuent jusqu’à présent.

**P34: IA_03_DPS_ANALYSTE_01.rtf - 34:7 [Répondant : Oui il y a certain..] (18:18)	(Super)**

Codes:	[SNIS_fonctionnement_Decrire_pdt MVE]

No memos

**Répondant** : Oui il y a certaines structures, nous avons eu à faire une supervision pour essayer de vois parce que nous là dans le cadre du DHSD il y avait l’incohérence des données, par ce que lors des analyses des données ici il y a des données aberrantes, il y a des données manquantes et donc nous sommes allés sur terrain nous avons noté que ç’a souffert. Tout cela par ce que l’agent qui était censé de le faire est pris de l’autre côté et même s’il pouvait le faire étant surchargé il se disait qu’il va travailler il pourra chercher un temps pour compléter malheureusement il oubliait maintenant les données qu’on devait mettre il y avait discordance et lorsqu’on a vérifié les données de base dans certaines aires de santé par rapport aux données du bureau central on a trouvé une discordance.

**P34: IA_03_DPS_ANALYSTE_01.rtf - 34:8 [Répondant : Pendant l’épidémie..] (20:20)	(Super)**

Codes:	[SNIS_fonctionnement_Decrire_pdt MVE]

No memos

**Répondant** : Pendant l’épidémie nous ne pensons peut-être vers la fin, au début de l’épidémie le système a été ébranlé et tout au long de l’épidémie c’était la même chose. C’est vers la fin que certaines zones ont commencée à se déchargé d’Ebola par ce que le chef de division faisait pression sur l’équipe cadre du bureau central pour qu’on remonte par ce que, la complétude au niveau national le Nord Kivu était devenu la dernière de 26 provinces dans la complétude et la promptitude de rapport seulement si nous notons le DHS2. Donc on a eu ce problème, bref au début et pendant le système était ébranlé c’est maintenant vers la fin qu’on commence à faire, même lorsque nous faisons les analyses des données par ce qu’on le fait trimestriellement, les analyses de troisième trimestre 2019 a monté qu’il y a toujours ce problème d’incohérence. Ce n’est ne pas une négligence mais une surcharge du personnel.

**P34: IA_03_DPS_ANALYSTE_01.rtf - 34:9 [Répondant : Je dirais que tous..] (22:22)	(Super)**

Codes:	[SNIS_fonctionnement_Decrire_pdt MVE]

No memos

**Répondant** : Je dirais que tous entaient affectés, je pense que par rapport aux canevas le premier problème que nous avons, ça va avec la connectivité et beaucoup de zones disent qu’elles n’avaient pas d’internet, et certaines zones disent qu’elles doivent se déplacer vers la ville pour saisir les données et ça c’est le credo de tous les jours, la version du côté d’Ebola ne peut pas être la raison. La raison qui reste c’est la connectivité mais on a senti qu’il y avait baisse.

**P34: IA_03_DPS_ANALYSTE_01.rtf - 34:10 [Répondant : Oui par moment, l’..] (24:24)	(Super)**

Codes:	[Insécurité_Conséquences]

No memos

**Répondant** : Oui par moment, l’insécurité est devenue une histoire endémique et que les équipes sont déjà habituées à ces genres de choses, nous ne disons pas que c’est bon mais ils cherchent les mécanismes par ce qu’ils doivent être performants. L’insécurité en soit ça bouleverse mais pas, on minimise ça même les zones les plus insécurisées remontent les données, surtout qu’ils travaillent avec la connectivité d’internet. Quand ils saisissent nous les trouvons facilement.

**P34: IA_03_DPS_ANALYSTE_01.rtf - 34:11 [Répondant : Je peux vous avoue..] (26:26)	(Super)**

Codes:	[Ressources_humaines_situation_grève]

No memos

**Répondant** : Je peux vous avouer que c’était terrible, donc on a fait trois mois et d’ailleurs l’épidémie d’Ebola nous l’avions récupérée avec retard par ce que les données n’arrivaient pas. Nous on a connu cette grève en Mai, Juin Juillet 2018 que les infirmiers étaient dans la grève là et on ne pouvait pas nous envoyer les données. Pour avoir les données c’était des exercices compliqués, il fallait créer des mouchards dans des équipes pour essayer de remonter mais c’était difficile par ce que s’ils constataient les données arrivaient à travers X personne ils allaient avoir un problème. Avec l’arrivée de MVE ils entaient obligés d’arrêter par eux-mêmes, on leurs avaient demander d’arrêter leurs grèves et continuer. Cette grève était comme suspendue suite à la MVE maintenant cette dernière est finie on ne sait pas s’ils vont encore relancer. C’est une forme compliquée de grève, on aimerait une autre forme de grève mais pas celle qui est administrative car nous étions beaucoup pénalisés.

**P34: IA_03_DPS_ANALYSTE_01.rtf - 34:12 [Répondant : Par rapport à notr..] (28:28)	(Super)**

Codes:	[SNIS_fonctionnement_Decrire_pdt MVE]

No memos

**Répondant** : Par rapport à notre province, ça évolue bien par ce qu’aujourd’hui sur trente-quatre zones que nous avons, toutes ont déjà été formée. Les équipes cadres sont toutes formés, ils savent ceux qu’ils doivent faire par rapport au DHSD et nous nous félicitons de ça. Nous nous félicitons aussi du fait que nonobstant le MVE, les équipes se sont efforcées à ne pas détruire le système du DHS2, donc l’information venait même si s’arrivait avec retard. Alors le problème que nous avons lepiné c’est celui de connectivité de l’internet par ce que chaque fois on nous revient là-dessus et par rapport à la complétude, promptitude et tout ça va un tout petit, seulement que si les choses se normalisaient on pourra facilement récupérer notre leader en rapport avec ça. Par ce qu’il n’y a rien qui peut empêcher que nous remontions. Il y a seulement ses évènements là, s’ils passaient et que les gens reviennent à leur travail à leurs places ça pourrait démarrer. On n’a pas connu un grand cout là-bas

**P34: IA_03_DPS_ANALYSTE_01.rtf - 34:13 [Répondant : En tout cas de l’e..] (30:30)	(Super)**

Codes:	[SNIS_fonctionnement_Solutions aux défis]

No memos

**Répondant** : En tout cas de l’expérience que nous avons eue, nous pourrons conseiller un certain nombre de choses : Quand un système pareil arrive qu’au niveau national on enlève le jargon de dire que ça c’est un problème national, quand ils disent problème national signifie que toutes les décisions se prennent au niveau de Kinshasa, au niveau provincial on ne fait qu’exécuter leurs décisions prises, vous n’êtes pas impliqué c’est déjà une mauvaise approche de considérer que les gens doivent venir de Kinshasa pour le faire. Responsabiliser les équipes, par ce que si on responsabilisait les équipes cadre soit de la zone ou de la DPS connaissant le système ils pouvaient voir comment gérer la situation, par exemple quand il s’agit de la vaccination, je prends le programme de PEV on va dire que telle personne s’occupe de ça et les autres continuent avec les activités de routine, si des choses pareils arrivaient c’est vrai que ça viennent avec panique mais quand même on pouvait dire, le système est là pour ne pas le détruire on prend certaines personnes du système qui travail, s’ils veulent être appuyer par d’autres on pouvait les associer pour qu’une équipe travail aux urgences et l’autres travail dans les activités des routines. Autre chose serait dans la prise en charge qui nous a couté cher, lorsque le MVE est entée des gros sous sont injecté dans le système tous cela qui devait travailler ils suivent le système et je vous ai dit mes collègues ont été obliger de partir comme nous vivons dans une situation de pauvreté donc là où il y a l’argent on y va. Qu’est-ce que je fais au Centre de santé, au Bureau central ou à la DPS si on ne me paie pas alors que là il y a cent dollars par jour, ce genre de chose ne marche pas. Les autorités devraient savoir quand s’arrive on fait un système tel que ça rentre dans les structures qu’on partage par ce que le risque est pour tout le monde. Comme ça les gens ne vont pas déserter le système pour aller chercher là où il y a l’argent. L’implication de la population car ce qu’on a connu c’est l’importation de ressources humaines, donc on amène les ressources humaines d’ailleurs en négligent les locaux ç’a fait la frustration au niveau de la Province, mais si on utilise les personnels locaux et on ne dit pas qu’on ne peut pas prendre les gens qui sont expérimentés.

**P34: IA_03_DPS_ANALYSTE_01.rtf - 34:14 [Répondant : Vraiment pas, lors..] (32:32)	(Super)**

Codes:	[Structures-Services_fonctionnement]

No memos

**Répondant** : Vraiment pas, lorsqu’on regarde les données au système toutes les informations sont renseignées. Certainement qu’il y a eu quelques défaillances par ci par là par ce que nous avons senti qu’il avait certaines zones qui était performantes on avait remarqué qu’elles dégraissaient par rapport aux activités qu’elles fournissaient, donc il y a eu baisse mais on n’a pas trouvé un scandale pour dire que telle gens sont allés pour un service qu’ils n’ont pas eu. Il y avait toujours mais à cause de cet embouteillages les indicateurs ont baissés globalement

**P34: IA_03_DPS_ANALYSTE_01.rtf - 34:15 [Répondant : Oui dans le grand ..] (34:34)	(Super)**

Codes:	[Structure-Service_Utilisation_population]

No memos

**Répondant** : Oui dans le grand nord il y avait et comme vous y serez, vous pourrez vérifier ça. Au fait avec la présence du MVE, comme les gens ne supportaient pas c’est-à-dire utilisaient le service de santé, c’était devenu officieuse, ils étaient d’alerte quand tu vas là-bas soit vous attrapez la maladie ou soit vous mourrez. Il y avait des gens qui ont déserté et nous avons constaté là au grand nord plus les gens ont fréquenté les structures privées où il n’y avait pas la prise en charge de la MVE que nos structures habituelles. Ça se disait même en route que les gens ne voulaient plus aller là où on trait	ait la MVE puisqu’ils avaient peur d’être contaminé, les autres ont eu peur à cause de groupe de pression quand il vous voit là on vous mal traite. Parfois ils se faisaient soigner en cachette. Je me rappelle dernièrement j’étais à Katwa j’avais parlé avec un IT par rapport à ça, il me dira que les gens viennent la nuit, s’il vienne la journée soit il serait mal vu par la population. Il y a même les structures privées qui soignaient la nuit et la journée il n’y a rien, elles ferment pendant la journée.

**P34: IA_03_DPS_ANALYSTE_01.rtf - 34:16 [Répondant : Oui, c’est le grou..] (36:36)	(Super)**

Codes:	[Structure-Service_Utilisation_Alternatives_population]

No memos

**Répondant** : Oui, c’est le groupe de pression, ils avaient peur et quand vous serez là vous allez comprendre, difficile à expliquer donc il y a les groupes de pression, ils campent autours des structures, il y en a même à Katwa où on vient chercher l’IT et ce dernier était obligé d’abandonner la structure pour aller ailleurs. Donc c’est un système de terreurs qu’ils ont organisé eux-mêmes pour faire peur aux gens qu’il ne fallaient pas aller aux structures qui traitent la MVE.

**P34: IA_03_DPS_ANALYSTE_01.rtf - 34:17 [Répondant : Non en fait par ra..] (38:38)	(Super)**

Codes:	[Commentaire-Recommandation_Répondant]

No memos

**Répondant** : Non en fait par rapport à la recherche c’est bon, nous aimerions que ce genre d’évènement quand ç’arrive ne nous surprenne pas, par rapport au choléra nous sommes avec le choléra depuis 1990 et à chaque fois qu’il y a épidémie de choléra on l’attaque comme si c’était la nouvelle épidémie, alors que nous avions plus de vingt ans d’expérience à lutter contre la maladie mais chaque fois qu’il y a une flambée on recommence à mettre les structures en places. Et nous craignons même qu’après avoir combattu plus deux ans Ebola et que cinq ans ; six ans Ebola revenait qu’on puisse la prendre comme si c’était nouvelle. Donc votre recherche nous souhaiterions qu’au bout de celle-ci les décideurs asseyent de normaliser les choses, que les évènements ne nous surprennent pas toujours. Il y a des choses élémentaires qu’on apprises à l’école par exemple quand on est médecin, on consulte il faut laver les mains, porter les gants : c’est des petites choses qu’on peut seulement remonter et mettre dans chaque structure de santé. Des histoires de lavage des mains que ça soit pérenne avec ou sans épidémie que ça reste car nous devons être professionnel après la population suivrait. Donc de tirer quand même l’expérience de celle-ci et que les autorités essayent de prendre des décisions du genre à sécuriser tout le monde. On vous en courage par ce que le résultat permettrait de décider par ce qu’ici chez nous l’information sanitaire et quand on prend les données c’est pour vous permettre de décider.

**P35: IA_03_PROGRAM_MCP 02.rtf - 35:1 [Répondant : je suis le respons..] (2:2)	(Super)**

Codes:	[Fosa-Institution_Présentation]

No memos

**Répondant** : je suis le responsable du PEV, un programme qui appuie techniquement la DPS en matière de la vaccination contre les maladies évitables par le vaccin et nous sommes le conseiller de chef de division dans cette matière et nous bénéficions également de l’appui des partanires l’OMS, de l’Unicef et d’autres partenaires dans les urgences pour appuyer la prévention vaccinale à travers nos activités.

**P35: IA_03_PROGRAM_MCP 02.rtf - 35:2 [Répondant : pendant la période..] (4:4)	(Super)**

Codes:	[Répondant_Responsabilité_pdt MVE]

No memos

**Répondant** : pendant la période d’Ebola, nous étions au front, nous étions parmi les premiers programmes qui a compris et nous étions déjà au front à partir du 27 juillet, on était déjà à Mangina d’abord pour les investigations, on était dans l’équipe d’investigation, parmi les trois personnes qui ont quitté Goma on était dans l’équipe d’investigation au niveau de Mangina et après l’investigation nous sommes restés là-bas pour organiser la vaccination. L’avantage est que j’étais à l’Equateur pour la vaccination contre Ebola donc je comprenais déjà quelque chose et comme le vaccin était là, je commençais à préparer les équipes pour la vaccination. A partir du 1^er^ Aout, on a commencé la vaccination du personnel du première ligne qui était en contact de haut risque à Mangina et nous avons constitué la commission vaccination. Et en ce moment, on avait quatre commissions, on avait la commission vaccination, la commission surveillance, la commission PCI et la commission point d’entrée. C’est juste deux, trois semaines après que d’autres commissions se sont ajoutées notamment la logistique, la prise en charge médicale et la psychosociale donc nous avons joué un rôle maitresse dans la riposte contre la maladie à virus Ebola

**P35: IA_03_PROGRAM_MCP 02.rtf - 35:3 [Répondant : Au début, il y ava..] (6:8)	(Super)**

Codes:	[Considération_Ebola_population]

No memos

**Répondant** : Au début, il y avait du pain sur la planche, la population ne croyait pas à ebola, la population croyait plus à une situation maléfique, il croyait à un problème de sorcellerie parce que ça commençait à Makiki. Makiki, c’est une aire de santé de la province de l’Ituri de la zone de santé de Mandima et Mangina, c’est le grand centre qui est au Nord-Kivu, ils sont venus se faire soigner à Mangina et à partir de là l’épidémie a continue aussi dans un contexte difficile parce qu’en ce moment-là le personnel soignant était en grève donc la surveillance épidémiologique avait de soucis pour collecter les MAPEPI hebdomadaire, ce qui a fait que nous avions connu de retard et nous on est venu juste pour l’investigation et quand l’INRB a confirmé, c’était en ce moment que nous avions tous eu l’information que c’était Ebola. Parmi les quatre commissions qui avaient commission qui avaient commencé, la communication avait commencé et au début cette commission de communication, la population les lapidait, il ne croyait pas, il ne comprenait pas Ebola c’est quoi ?

Mais avec le temps, nous avons impliqué la communauté, les autorités, la société civile, les confessions religieuses, c’est en ce moment-là que l’évêque lui-même est descendu dans la communauté comme Mangina, c’est pratiquement catholique à 80 à 90%, la population a commencé à comprendre c’est comme ça qu’ils nous ont prêté main forte.

Mais un autre aspect autour de Mangina, il y avait des groupes armés notamment les Mai-mai qui ont constitué aussi un problème très sérieux pour aller dans les coins et recoins où il y avait des cas d’Ebola et une autre difficulté qu’on avait dans notre culture, dans la partie nord de notre province, quand quelqu’un est malade, il est mort, on l’enterre dans son village natal. Et aussi les membres de famille quittent loin pour venir assister quelqu’un qui est malade. C’est comme ça qu’après Mangina, le cas est parti à Masereka càd quelqu’un qui était malade, il est parti se faire soigner chez lui à Masereka, il a traversé Beni, il a traversé Kalunguta jusqu’aller à Butembo, Masereka et c’est comme ça que la maladie a pris de l’espace et à continuer à évoluer, heureusement qu’avec la sensibilisation on y est allé progressivement.

**P35: IA_03_PROGRAM_MCP 02.rtf - 35:4 [Répondant : c’est un changemen..] (10:10)	(Super)**

Codes:	[Structures-Services_fonctionnement]

No memos

**Répondant** : c’est un changement négatif d’abord, c’était sur un fond, le personnel était en grève et quand le personnel était en grève, ils adhèrent difficilement et à un moment l’équipe de la riposte avait installé l’Etat-major à Mangina et a commencé à impliquer le personnel de la santé, on ne faisait qu’Ebola et le reste des services a connu des problèmes de mise en œuvre puisqu’on s’occupait plus d’Ebola et tout ce qui est offre de services, on avait des difficulté énorme, je prends par exemple la vaccination en milieu rural, on vaccine 4 fois le mois et on vaccinait pratiquement une fois le mois et ça constituait un aspect négatif.

**P35: IA_03_PROGRAM_MCP 02.rtf - 35:5 [Répondant : la raison était qu..] (12:13)	(Super)**

Codes:	[Structures-Services_fonctionnement]

No memos

**Répondant** : la raison était que tous les relais communautaire, tous les infirmiers étaient embauchés à Ebola et ils ne faisaient plus leur activité régalienne. Ça a été vraiment une conséquence sinon vraiment sur le programme de la vaccination sinon les autres programmes notamment le curatif et autres.

Le deuxième aspect, il y a les tradipraticiens donc quand ils ont vu que tous les prestataires étaient complices dans une maladie qu’il ne croyait pas, la plupart allait chez les tradipraticiens, ce qui fait que l’offre de service a connu beaucoup des difficultés et ça c’est poursuivie une année après, deux ans après malgré la gratuité des soins qui a été opté, ça toujours connu des difficultés par rapport à l’offre de service.

**P35: IA_03_PROGRAM_MCP 02.rtf - 35:6 [Répondant : Non, l’avantage es..] (15:15)	(Super)**

Codes:	[Service_vaccination_Fonctionnement]

No memos

**Répondant** : Non, l’avantage est que c’est moi qui coordonnais la vaccination de routine, la vaccination systématique et je cordonnais la commission donc je réussis à manager avec les médecins chef des zones pour ne pas interrompre avec la vaccination, ça c’est un aspect positif mais comprenez que le médecin chef de zone à lui seul ne pouvait pas, il fallait fédérer tous les IT malheureusement, ils étaient tous déjà embauchés, ça c’est un aspect qui nous a échappé en plus de ça il y avait l’insécurité qui ne faisait que grandir, l’hésitation à la vaccination. Comme on vaccinait contre Ebola, la population devenait de plus en plus hostile à la vaccination de routine mais l’avantage, c’est que beaucoup des gens connaissent déjà la rougeole c’est quoi ? Et les enfants mouraient de rougeole et on avait d’autres gens qui étaient pour la vaccination de routine. Et dans ce contexte, du fait qu’on a diminué la fréquence des vaccinations au niveau des structures, il y avait des flambées de rougeole. On devait aussi riposter et voilà un peu dans cet amalgame, la population nous a soutenu puisqu’il fallait continuer la vaccination de routine mais à un rythme plus au moins ralenti quoi.

**P35: IA_03_PROGRAM_MCP 02.rtf - 35:7 [Répondant : Bon, les ressource..] (17:17)	(Super)**

Codes:	[Ressources_humaines_Disponibilité et perturbations]

No memos

**Répondant** : Bon, les ressources humaines, comme vous savez l’histoire a commencé à Mangina et a suivi un bon nombre de chemin jusqu’ici, presque le gros lot du personnel qui travaillé sont venus de Mangina et de Beni donc on a dégarni complétement et ils ont commencé à faire des mouvements en suivant l’épidémie, c’est un autre aspect, on a vidé pratiquement les ressources humaines mais aussi le deuxième aspect, comme Mangina était l’épicentre contre Beni, le personnel on les a mis en congé surtout les gens traumatisent, ceux qui ont vécu les hostilités, on a importé aussi d’autres personnels dans le cadre du BDOM venus de Beni et Butembo pour venir renforcer Mangina mais ça n’a pas vraiment résolu le problème puisque comme vous le savez au niveau du paquet minimum d’activités au niveau des structures sanitaires et centres de santé, on avait beaucoup des difficultés puisqu’on a affaire à des nouveaux qui ne sont pas formés, on a aussi affaire à la qualité des prestations au niveau du structure.

**P35: IA_03_PROGRAM_MCP 02.rtf - 35:8 [Répondant : Au début non puisq..] (19:19)	(Super)**

Codes:	[Gratuité_Accueil_population]

No memos

**Répondant** : Au début non puisque les gens sont habitués à aller chez les tradipraticiens, ils ont plus confiance à leurs frères qui soignent avec les plantes traditionnelles, c’est vrai là-bas c’est contre nature quand vous allez chez un tradipraticien, vous amenez une poule, un coq, il vous soigne même en crédit et vous allez payer après. Donc la gratuité des soins ça nous apprit du temps pour sensibiliser, on a sensibilisé sérieusement et avec le temps les gens ont compris malheureusement cette gratuité de soins à un certain moment, on l’a coupé aussi. La population était aussi habituée à cette gratuité des soins mais en un moment, on l’a arrêté et ça nous a encore posé des difficultés au moment où la structure économique était paralysée aussi.

**P35: IA_03_PROGRAM_MCP 02.rtf - 35:9 [Répondant : cette gratuité éta..] (21:21)	(Super)**

Codes:	[Médicaments_disponibilité_fosa]

No memos

**Répondant** : cette gratuité était accompagnée par un approvisionnement en médicament parce qu’on avait calculé la moyenne d’approvisionnement dans les structures et les structures étaient approvisionnés par Astramex, ils avaient leur ligne de crédit et Astramex amenait les médicaments régulièrement et les vaccins c’est la même chose, nous n’avons pas connu des ruptures en vaccins sauf qu’on était en rupture des seringues BCG pour les enfants, les nouveau-nés. Et ç’apprit pratiquement trois mois et on a été approvisionné, de manière générale, les structures étaient approvisionnées en intrant durant la période de la crise et nous pour le vaccin, on n’a pas connu les problèmes majeurs

**P35: IA_03_PROGRAM_MCP 02.rtf - 35:10 [Répondant : au niveau de la ba..] (23:23)	(Super)**

Codes:	[SNIS_fonctionnement_Decrire_pdt MVE]

No memos

**Répondant** : au niveau de la base cela ne se faisait plus parce que chaque mois on avait de réunion d’analyse qu’on appelle des réunions inter-infirmières. Mais le fait que le gros des personnels étaient déjà embauchés, on a eu des ralentissements des réunions, les réunions étaient quasi rares, tout le trois mois une réunion au moment où c’est mensuel. Au court de cette réunion 48 heures avant on analyse les données, on prépare pour la réunion inter-infirmière proprement dite donc on a connu un ralentissement des réunions, ça c’est un aspect. Et même l’analyse des données et quand ça remonté au niveau de l’antenne, on fait aussi des analyses mais avec un problème de feedback, on fait des feedbacks aux structures mais ça ne suit les orientations et comme nous sommes une antenne au niveau de la province, on ne pouvait pas toucher les données, on envoyait les données à Kinshasa avec beaucoup d’aberrations. Ça c’est un aspect qualité que nous avons connu durant la période mais maintenant comme on organisait aussi des revues, on essayait de colmater chaque trimestre ce qui a à corriger et partager les données au niveau national. Nous avons aussi connu des problèmes parce que ça c’est par exemple les données des enfants vaccinés, nous avons aussi les données de surveillance épidémiologique ça posait des problèmes également parce que le focus était autour d’Ebola, on a eu l’impression d’oublier d’autres maladies à potentielles épidémiologiques et dans le temps on a eu des épidémies de rougeole, des épidémies de choléra mais Dieu merci, nous avons eu l’appui du niveau central avec tous les partenaires et organisait des ripostes et c’est ce que nous avons fait et pour le moment, la situation semble être maitrisée

**P35: IA_03_PROGRAM_MCP 02.rtf - 35:11 [Répondant : promptitude, c’eta..] (25:25)	(Super)**

Codes:	[SNIS_fonctionnement_Decrire_pdt MVE]

No memos

**Répondant** : promptitude, c’etait un problème, les données ne nous arrivent pas à temps, ça arrivait toujours en retard et ont devait toujours appeler les équipes cadres pour que les données nous arrivent. On avait connu vraiment un problème de promptitude mais au finish, on avait des données et l’analyse souffrait parce que quand on n’est pas prompt don on était obligé d’analyser les données de janvier à la mi-février donc on avait des problèmes par rapport aux actions qu’on devait mener. On a eu également des problèmes de complétude des données au niveau des structures sanitaires et les outils, c’était la même chose, le fait qu’on avait un problème de promptitude, les données quand ils voyaient qu’ils sont vraiment en retard, ils faisaient vite, vite et quand nous avions fait l’auto-évaluation de la qualité des données, on a constaté qu’on a trouvé des items, des registres incomplets donc il y a eu vraiment un sérieux problème de qualité des données.

**P35: IA_03_PROGRAM_MCP 02.rtf - 35:12 [Répondant : d’une part, c’étai..] (27:27)	(Super)**

Codes:	[Gouvernance_soins santé]

No memos

**Répondant** : d’une part, c’était positif mais au-delà de trois à six mois, ce n’était plus intéressant dans cette riposte contre Ebola puisqu’ils sont venus nous appuyer techniquement, on devrait former localement et appuyer localement mais ils ont dépassés le délai et ça nous a presque découragés puisque le gros n’accède pas aux endroits insécurisés et autres mais il y avait des inégalités dans le traitement càd on vient nous appuyer et on fait deux ans, trois ans, non, ça ne marche pas, on devait venir, on nous appuie tout au plus six mois et on nous observe et voir ce que nous allons faire mais cette capacitation qui continue est disproportionnée aussi par rapport au traitement, ça c’est un aspect aussi qui ne nous a pas enchanté don le gros des consultants qui sont venus internationaux, nationaux bien sûr qu’ils nous ont appuyé pendant un certain moment au moins ils devaient transférer les compétence mais c’est devenu éternel ça constitue beaucoup de couac dans le système.

**P35: IA_03_PROGRAM_MCP 02.rtf - 35:13 [Répondant : un aspect aussi im..] (29:29)	(Super)**

Codes:	[Supervision_pdt-épidémie-Ebola]

No memos

**Répondant** : un aspect aussi important qu’on doit signaler, le fait qu’on était dans Ebola, on était aussi dans PEV, on a travaillé dur pour maintenir les performances du programme en commençant par tout ce qui est suivi-évaluation, tout ce qui est mission des supervisions, on restait dans les limites de missions prévues de manière trimestrielle et on organisait également des revues trimestrielles par rapport à toutes ces activités, les revues pratiquement semestrielles et annuelles donc ce qui était bon, aussi pour la coordination, on avait chaque jour dans le cadre d’Ebola, on avait une réunion matinale et au niveau des programmes, chaque week-end, on avait aussi des réunions pour les programmes pour maintenir nos performances et des activités d’une manière générale.

**P35: IA_03_PROGRAM_MCP 02.rtf - 35:14 [Répondant : c’était beaucoup p..] (31:31)	(Super)**

Codes:	[Souhait_Changement_gestion_futures_épidémies]

No memos

**Répondant** : c’était beaucoup plus l’apprentissage càd nous avons plus l’expertise d’une manière générale en ce qui concerne la coordination mais dans le cadre de la coordination, la force qu’il faut, il faut renforcer quand on a une épidémie ailleurs et beaucoup plus responsabiliser les gens de terroir puisque ça c’est la faiblesse que nous avons connu dans cette riposte, on a des équipes cadre des zones et pour l’exemple du Nord-Kivu 90% des médecins chefs de zones sont déjà formés en épidémiologie, en santé publique maintenant on devait les formés, les capacité et les suivre et laisser leurs destins coordonner tous aspects, c’est un aspect très important, le deuxième aspect, c’est l’homme au centre des activités de traitement càd quand on a des activités comme ça, on doit traiter de manière spéciale les gens qui travaillent parce qu’il court les risque, ça aussi c’est l’évidence que nous devons savoir et en troisième lieu, le post épidémie, on doit avoir le plan de résilience de choc par rapport à tout ce qu’on a subi, nous avons travaillé dans un contexte d’insécurité pour la population, c’est injuste, 5, 10 ans nous sommes dans l’insécurité mais quand on a l’épidémie d’Ebola, c’est des milliards qui tombent, c’est injuste, pourquoi n’est pas d’abord terminé l’insécurité qui dure et qui continue à tuer aussi, l’insécurité a tué plus que l’épidémie pourquoi n’est pas arrêté l’insécurité donc de manière holistique, ailleurs s’il y a l’épidémie, il faut être plurisectoriel càd on doit intégrer d’autres domaines pour que nous puissions être bien compris par la communauté mais si on reste santé, on doit impliquer la sécurité, on doit impliquer le transport, on doit impliquer d’autres domaines, comme ça comme la MVE, c’est pratiquement tout est dedans donc ailleurs il faut impliquer tout le monde pour que ça soit vraiment multisectoriel

**P36: IA_04_AS_IT_01.rtf - 36:1 [Répondant : Mon travail consis..] (9:9)	(Super)**

Codes:	[Répondant_Responsabilité_habituelles]

No memos

**Répondant** : Mon travail consiste à être un leader du centre de sante MASULI, qui est un centre étatique intègre dans la zone de sante de KATWA.

**P36: IA_04_AS_IT_01.rtf - 36:2 [Répondant : Je suis prestatair..] (11:11)	(Super)**

Codes:	[Répondant_Responsabilité_habituelles]

No memos

**Répondant** : Je suis prestataire au centre comité, avec une expérience de 12ans.

**P36: IA_04_AS_IT_01.rtf - 36:3 [Répondant : C’était un cas en ..] (13:13)	(Super)**

Codes:	[Début-Progression_Epidémie MVE]

No memos

**Répondant** : C’était un cas en provenance de l’aire de sante de WAYENE, et quand je quittais la communauté pour la maison, il y’avait des mamans qui vendaient des choux et elles m’avaient dit qu’elles savent que quand on trouve un cas on nous donne de l’argent, cette fois voici un cas dans notre quartier, c’est une fille, mais si vous y aller, vous serez tabassé, je me suis rendu au centre de sante de sante, et le lendemain c’était le MSZ qui m’a appelé et me demanda si j’ai reçu l’information, et MSZ m’a demandé d’y aller récolter les données et de rester sur l’endroit, c’est ainsi qu’on a fait l’alerte, on fait comprendre les parents et on a pris le malade. C’était ma première fois de voir un cas. Une fois à la maison, j’ai été tabassé par la population. Le malade restait chez un députe, une fois sa sante s’est altéré, le malade est retourné à la maison. C’est ainsi qu’on avait vacciner plus de 200 personnes ce jour-là.

**P36: IA_04_AS_IT_01.rtf - 36:4 [Répondant : La population comp..] (15:15)	(Super)**

Codes:	[Considération_Ebola_population]

No memos

**Répondant** : La population comprenait bien l’Ebola quand on avait reçu le message de MANGENA jusqu’ici en disant qu’il a une maladie qui se transmet par contamination, et les gens ont compris. Mais quand le cas est arrivé chez nous, la communauté a été intoxiquée par ce fameux députe qui disait que les prestataires venaient et gagnaient de l’argent, et les gens ont perdu confiance aux prestataires, et quand on a découvert un deuxième cas, il est venu encore et il a encore intoxiqué. Et c’est après un temps que les gens ont compris

**P36: IA_04_AS_IT_01.rtf - 36:5 [Répondant : Pour un premier te..] (17:17)	(Super)**

Codes:	[Considération_prestataire_population]

No memos

**Répondant** : Pour un premier temps, on nous considéré comme des sorciers, ils disaient que les prestataires collaboraient avec ceux du CTE pour que les malades qui y arrivent ne puissent pas retourner. Les malades quittaient dans la communauté en étant intoxique, une fois au CTE, ils refusaient la nourriture. Et d’autres interpellait les médicaments du CTE. Alors les malades refusaient les médicaments, d’autres mourraient de ça. Et c’est maintenant, après un temps ils ont compris la situation.

**P36: IA_04_AS_IT_01.rtf - 36:6 [Répondant : Tout le monde a ac..] (19:19)	(Super)**

Codes:	[Triage_Considération_population]

No memos

**Répondant** : Tout le monde a accepté le triage, car personne n’a jamais démoli le bâtiment du triage.

**P36: IA_04_AS_IT_01.rtf - 36:7 [Répondant : C’est là où résida..] (21:21)	(Super)**

Codes:	[Triage-Isolement-Chlore-thermoflash_Réaction_population]

No memos

**Répondant** : C’est là où résidait le problème et les gens ne voulaient pas se laver les mains et surtout que l’eau qui contient du chlore dégage de l’odeur. Et les grands parents disaient ce chlore c’est l’Ebola, mais avec le PSY et même les chefs de 10maisons, on a pu faire comprendre la population que c’est le chlore qui est utilisé pour le lavage des mains.

**P36: IA_04_AS_IT_01.rtf - 36:8 [Répondant : Les mamans de CPN,..] (23:23)	(Super)**

Codes:	[Service_vaccination_Fonctionnement] [Structure-Service_Fermeture_pdt MVE]

No memos

**Répondant** : Les mamans de CPN, CPS nous ont fuis pendant cette période de l’épidémie, on ne parvenait pas de vacciner plus de 10 enfants le BCG, on observait une affaire de 5 malades par semaine alors qu’avant c’était entre 25 et 30 malades.

**P36: IA_04_AS_IT_01.rtf - 36:9 [Répondant : Ils ont fui à caus..] (25:25)	(Super)**

Codes:	[Structure-Service_Utilisation_Raisons fuites FOSA publique_population]

No memos

**Répondant** : Ils ont fui à cause des rumeurs, ils pensaient qu’on devrait les injecter avec le thermomètre a laser. C’est alors les chefs de quartiers qui nous aider à faire comprendre la population. Alors les structures privées avaient profité, les malades qui fouillaient se dirigeaient vers les structures privées. C’est alors quand les privées étaient contaminées, c’est alors que les ONG sont venues et ont créé le triage dans ces hôpitaux. C’est alors que la population a compris que la maladie est partout

**P36: IA_04_AS_IT_01.rtf - 36:10 [Répondant : Il y’a des structu..] (27:27)	(Super)**

Codes:	[Structure-Service_Fermeture_pdt MVE]

No memos

**Répondant** : Il y’a des structures qui ont fermées et surtout les privées, quand on découvrait les cas positifs dans ces structures, il fallait décontaminer ces structures, et quand aller décontaminer, tous les malades fuyaient et ces structures restaient vide et fermaient.

**P36: IA_04_AS_IT_01.rtf - 36:11 [Répondant : La qualité de serv..] (29:29)	(Super)**

Codes:	[Qualité_soins_Prestataires]

No memos

**Répondant** : La qualité de service n’était pas vraiment bien, on faisait la prescription des médicaments mais les malades avaient toujours peur, ils doutaient de ces médicaments. Et ils achetaient leurs médicaments aux pharmacies.

**P36: IA_04_AS_IT_01.rtf - 36:12 [Répondant : Les malades étaien..] (31:31)	(Super)**

Codes:	[Triage-Isolement-Chlore-thermoflash_Réaction_population]

No memos

**Répondant** : Les malades étaient dérangés, par nos tenus, ils ne voulaient pas que nous portions les gans, l’imperméable, ils n’acceptaient pas qu’on puisse les accueillir avec cette tenue, en disant qu’ils ne sont pas contaminés.

**P36: IA_04_AS_IT_01.rtf - 36:13 [Répondant : Ils refusaient tou..] (33:33)	(Super)**

Codes:	[Triage-Isolement-Chlore-thermoflash_Réaction_population]

No memos

**Répondant** : Ils refusaient toujours nos dispositifs, c’était toujours un problème quand on voulait décontaminer leurs cellules. Et ils avaient d’inquiétude à propos du chlore.

**P36: IA_04_AS_IT_01.rtf - 36:14 [Répondant : Avec Ebla, on avai..] (35:35)	(Super)**

Codes:	[Traitement_Invasifs_Prestataire_changement]

No memos

**Répondant** : Avec Ebla, on avait déjà change, parce qu’on utilisait beaucoup des routines, la prescription a changé, on ne pouvait pas prendre un malade qui présentait plus de trois pathologies comme auparavant, on le conduisait au CTE.

**P36: IA_04_AS_IT_01.rtf - 36:15 [Répondant : On fait uniquement..] (37:37)	(Super)**

Codes:	[Instructions_pdt_MVE]

No memos

**Répondant** : On fait uniquement le TDR, palu simple, fièvre céphalée, on a interdit ça parce que ces signes sont confondus aux signes d’Ebola. Les patients venaient et passaient au triage quand leurs fièvres étaient de 36,5 ou 37,2. Alors après trois heures du temps, quand vous les recevez à la consultation, vous allez remarquer que leurs fièvres encore montent, si vous faites le TDR, il peut être négatif, mais il y’a eu contact. C’est pour cela qu’on a interdit le TDR. Avec le liquide biologique, on a eu peur de toucher ce liquide. Il avait le tri des déchets.

**P36: IA_04_AS_IT_01.rtf - 36:16 [Répondant : Il ne fallait pas ..] (39:39)	(Super)**

Codes:	[Prestataire_Attitude_devant_malade]

No memos

**Répondant** : Il ne fallait pas faire le hasard, il fallait être en épis et c’est alors qu’on pouvait aller dans la salle d’accouchement.

**P36: IA_04_AS_IT_01.rtf - 36:17 [Répondant : On avait peur et m..] (41:41)	(Super)**

Codes:	[Prestataire_Attitude_devant_malade]

No memos

**Répondant** : On avait peur et même jusqu’à maintenant on a toujours peur de ce fléau,

**P36: IA_04_AS_IT_01.rtf - 36:18 [Répondant : Ebola a vraiment c..] (43:43)	(Super)**

Codes:	[Prestataire_Attitude_devant_malade]

No memos

**Répondant** : Ebola a vraiment changé la mentalité de tous les prestataires.

**P36: IA_04_AS_IT_01.rtf - 36:19 [Répondant : Quand le malade ar..] (45:45)	(Super)**

Codes:	[Organisation_travail-Circuit des malades_pendant MVE]

No memos

**Répondant** : Quand le malade arrive au triage, la toute première chose c’est la définition, et c’est après qu’on peut l’orienter, soit dans les structures. Et quand il arrive au lit on appelle encore les investigateurs qui vont échanger avec lui, après deux jours, s’il n’a pas de changement on valide encore.

**P36: IA_04_AS_IT_01.rtf - 36:20 [Répondant : Oui parce que nous..] (47:47)	(Super)**

Codes:	[Prestataire_Attitude_devant_malade]

No memos

**Répondant** : Oui parce que nous les IT, nous sommes obligés de préparer les malades quand ils arrivent, on le montre comment on travaille avec crainte vu l’épidémie que nous vivons, on le demande de ne pas être effrayé.

**P36: IA_04_AS_IT_01.rtf - 36:21 [Répondant : On n’a pas eu de p..] (49:49)	(Super)**

Codes:	[Ressources_humaines_Disponibilité et perturbations]

No memos

**Répondant** : On n’a pas eu de problème, on travaillait en collaboration avec eux, ils nous donnaient des idées…

**P36: IA_04_AS_IT_01.rtf - 36:22 [Répondant : Elle n’a pas pertu..] (51:51)	(Super)**

Codes:	[Ressources_humaines_solution-local_Disponibilité et perturbations]

No memos

**Répondant** : Elle n’a pas perturbé la structure, elle travaillait la journée, et ensuite elle venait nous aider le soir. Elle était proche de la coordination, elle nous donnait des informations et avec ça on a été avantagé.

**P36: IA_04_AS_IT_01.rtf - 36:23 [Répondant : Chez nous ça n’a p..] (53:53)	(Super)**

Codes:	[Ressources humaines_Motivation_personnel]

No memos

**Répondant** : Chez nous ça n’a pas causé de problème.

**P36: IA_04_AS_IT_01.rtf - 36:24 [Répondant : La grève a été déc..] (55:55)	(Super)**

Codes:	[Ressources_humaines_situation_grève]

No memos

**Répondant** : La grève a été déclenché a cause du prime de risque, et cela que le cas a quitté MANGENA, car on confondait les signes avec les hémorragies, il n’y avait même pas de rapport hebdomadaire qu’on envoyait.

**P36: IA_04_AS_IT_01.rtf - 36:25 [Répondant : Peut-être deux moi..] (57:57)	(Super)**

Codes:	[Ressources_humaines_situation_grève]

No memos

**Répondant** : Peut-être deux mois.

**P36: IA_04_AS_IT_01.rtf - 36:26 [Répondant : Avec cette gratuit..] (59:59)	(Super)**

Codes:	[Gratuité_Début]

No memos

**Répondant** : Avec cette gratuite, on a été soulage, malgré que la banque ne nous servait pas à temps, parce qu’avant on dépendait des malades.

**P36: IA_04_AS_IT_01.rtf - 36:27 [Répondant : Avec l’aide de REC..] (61:61)	(Super)**

Codes:	[Gratuité_Rumeurs]

No memos

**Répondant** : Avec l’aide de RECO, ils nous ont aider à faire comprendre la population qu’il a l’union européenne qui donnait les médicaments gratuitement. La population disait que c’est impossible qu’on donne les médicaments gratuitement,

**P36: IA_04_AS_IT_01.rtf - 36:28 [Répondant : La gratuite a été ..] (63:63)	(Super)**

Codes:	[Gratuité_Structures-Services_concernés]

No memos

**Répondant** : La gratuite a été seulement aux HGR, aux CS, et aux CHR et d’autres structures.

**P36: IA_04_AS_IT_01.rtf - 36:29 [Répondant : D’autres structure..] (65:65)	(Super)**

Codes:	[Gratuité_Structures-Services_concernés]

No memos

**Répondant** : D’autres structures non. Ils sont au nombre de 18.

**P36: IA_04_AS_IT_01.rtf - 36:30 [Répondant : C’est le BCZ qui n..] (67:67)	(Super)**

Codes:	[Gratuité_Structures-Services_concernés]

No memos

**Répondant** : C’est le BCZ qui nous a proposé ça, mais les privées ont refusé.

**P36: IA_04_AS_IT_01.rtf - 36:31 [Répondant : Dans l’utilisation..] (69:69)	(Super)**

Codes:	[Gratuité_Conséquences_utilisation_services]

No memos

**Répondant** : Dans l’utilisation des services, le taux d’utilisation du curatif a changé, les taux de CPN ont aussi monte, mais chez eux ça baisser.

**P36: IA_04_AS_IT_01.rtf - 36:32 [Répondant : Il a eu augmentati..] (71:71)	(Super)**

Codes:	[Gratuité_Conséquences_motivation_prestataire]

No memos

**Répondant** : Il a eu augmentation, parce que sans cette gratuite, pour avoir même cinq dollars c’était difficile.

**P36: IA_04_AS_IT_01.rtf - 36:33 [Répondant : On a passe par les..] (73:73)	(Super)**

Codes:	[Gratuité_Information_population]

No memos

**Répondant** : On a passe par les voix de RECO.

**P36: IA_04_AS_IT_01.rtf - 36:34 [Répondant : Pour le moment, on..] (75:75)	(Super)**

Codes:	[Gratuité_Evolution]

No memos

**Répondant** : Pour le moment, on demande les cartes d’électeur, ils viennent avec les photocopies de leurs cartes contrairement dans le passé, c’était à nous de le faire. Mais le problème réside au niveau des numéros, ils refusent de donner leurs numéros.

**P36: IA_04_AS_IT_01.rtf - 36:35 [Répondant : C’était due au niv..] (77:77)	(Super)**

Codes:	[Gratuité_Evolution]

No memos

**Répondant** : C’était due au niveau de la DPS.

**P36: IA_04_AS_IT_01.rtf - 36:36 [Répondant : Le taux a baissé, ..] (79:79)	(Super)**

Codes:	[Gratuité_Evolution]

No memos

**Répondant** : Le taux a baissé, mais on le faisait comprendre qu’il fallait payer quelques choses pendant cette période de l’interruption de gratuite.

**P36: IA_04_AS_IT_01.rtf - 36:37 [Répondant : C’est là où résida..] (81:81)	(Super)**

Codes:	[Médicaments_disponibilité_fosa]

No memos

**Répondant** : C’est là où résidait le problème, parce qu’on ne sait pas là ou sont cachés le 10%. Alors on prend uniquement le 20% au niveau de la banque, et jusqu’à maintenant on attend, parce qu’avec ASRAMESS, on ne livrait même pas ce qu’on a commandé.

**P36: IA_04_AS_IT_01.rtf - 36:38 [Répondant : Le travail a été f..] (83:83)	(Super)**

Codes:	[SNIS_fonctionnement_Decrire_pdt MVE]

No memos

**Répondant** : Le travail a été fait, mais on amener ça tardivement, parce qu’on était pris à l’épidémie, il y’a de fois où on voulait travailler, mais l’équipe nous appelait pour observer un autre cas. Avec les réunions, on ne respectait leurs dates, et on amenait le compte rendu chez le TDR avec retard.

**P36: IA_04_AS_IT_01.rtf - 36:39 [Répondant : Quand l’épidémie a..] (85:85)	(Super)**

Codes:	[Souhait_Changement_gestion_futures_épidémies]

No memos

**Répondant** : Quand l’épidémie arrive, il faut faire travailler en premier lieu les personnels de santé qui sont connus dans le milieu, la population n’a pas aimé de voir des nouvelles figures, on faisait même travailler même les femmes libres qu’on ramassait ici à BUTEMBO, peut-être s’ils venaient et recrutaient les enfants bien connus avoir fait la médecine, on n’aura pas de problème.

**P36: IA_04_AS_IT_01.rtf - 36:40 [Répondant : Il fallait aussi i..] (87:87)	(Super)**

Codes:	[Commentaire-Recommandation_Répondant]

No memos

**Répondant** : Il fallait aussi impliquer toutes les autorités politiques.

**P36: IA_04_AS_IT_01.rtf - 36:41 [Répondant : J’aimerai qu’on no..] (89:89)	(Super)**

Codes:	[Commentaire-Recommandation_Répondant]

No memos

**Répondant** : J’aimerai qu’on nous donne à nous qui avons travaillé dans la riposte, des certificats qui montre qu’on a fait quelque chose.

**P37: IA_04_AS_IT_02.rtf - 37:1 [Répondant : Bonjour, Je suis i..] (7:7)	(Super)**

No codes

No memos

**Répondant** : Bonjour, Je suis infirmier au centre de sante de VUHIKA en qualité d’IT dans Cette aire de santé et zone de santé de KATWE.

**P37: IA_04_AS_IT_02.rtf - 37:2 [Répondant : Mon travail consis..] (9:9)	(Super)**

Codes:	[Répondant_Responsabilité_habituelles]

No memos

**Répondant** : Mon travail consiste d’abord à la gestion de centre de santé, assumer la responsabilité de cette structure sanitaire qui est le centre de santé de VUHIKA

**P37: IA_04_AS_IT_02.rtf - 37:3 [Répondant : Autre chose c’est ..] (11:11)	(Super)**

Codes:	[Répondant_Responsabilité_habituelles]

No memos

**Répondant** : Autre chose c’est aussi orienter les activités de soins de santé primaire au sein de cette entité de la zone de santé de KATWA

**P37: IA_04_AS_IT_02.rtf - 37:4 [Répondant : Brièvement l’épidé..] (13:13)	(Super)**

Codes:	[Début-Progression_Epidémie MVE]

No memos

**Répondant** : Brièvement l’épidémie est arrivée dans mon ère de sante le premier cas c’était deux visiteurs en provenance de Beni qui étaient de dames, heureusement Dieu nous a aider à vaincre ce fléau de l’Ebola qui était parmi le deux vainqueurs qui n’ont pas succombé, il a été vite dépisté et vite pris en charge au sein du CTE de Butembo c’était ça le premier cas. Aussi d’autres cas en aient survenu dans notre ère de sante avec un total de 28 cas dans cette aire de sante, on était notifié au sein de l’aire de sante de VUHIKA.

**P37: IA_04_AS_IT_02.rtf - 37:5 [Répondant : Le rôle que j’ai j..] (15:15)	(Super)**

Codes:	[Répondant_Responsabilité_pdt MVE]

No memos

**Répondant** : Le rôle que j’ai joué dans le cadre de la riposte, j’ai joué le rôle dans la surveillance épidémiologique au sein de cette aire de santé de VUHIKA

**P37: IA_04_AS_IT_02.rtf - 37:6 [Répondant : La surveillance ça..] (17:17)	(Super)**

Codes:	[Répondant_Responsabilité_pdt MVE]

No memos

**Répondant** : La surveillance ça veut dire que, voir les différentes façons, comment détecter les cas et je me suis entretenue avec mes différents responsable de FOSA dans l’implication de la mise en œuvre de différentes définitions de cas, comment est-ce qu’on peut définir un cas de la MVE. En tant que I.T j’ai joué aussi un rôle dans la communication pour entrer dans un ménage qui a un cas ce n’était pas facile aux équipes d’accéder à la famille sans l’implication de l’I.T qui est un peu populaire dans cette entité c’est ça les deux rôles que j’ai joués. Dans la sensibilisation aussi, on a essayé à sensibiliser la communauté pour l’acceptation de la vaccination au sein de l’aire de santé& autour du cas qu’on avait enregistré car la vaccination était assez dure

**P37: IA_04_AS_IT_02.rtf - 37:7 [R selon la population elle ne ..] (19:19)	(Super)**

Codes:	[Considération_Ebola_population]

No memos

R selon la population elle ne croyait pas à Ebola , pourquoi elle ne croyait pas a l’Ebola la population disait que c’est un montage pourquoi selon la définition fièvre hémorragique elle s’attendait à une personne qui a Ebola elle pouvait voir hémorragie provient dans tout l’orifice d’une façon la vis-à-vis d’où presque tous les cas qui on étaient notifier positif plusieurs ne présenter pas le signe de hémorragie ils présentaient de vomissement de asthénie , mais la population selon elle avait une mauvaise définition sur la fièvre hémorragique. Elle croyait avoir vu l’hémorragie venir soit par le nez, la bouche ou par les oreilles il y avait eu une petite résistance.

**P37: IA_04_AS_IT_02.rtf - 37:8 [Répondant : Au sein de la comm..] (21:21)	(Super)**

Codes:	[Considération_Ebola_population]

No memos

**Répondant** : Au sein de la communauté

**P37: IA_04_AS_IT_02.rtf - 37:9 [Répondant : par rapport à la r..] (23:23)	(Super)**

Codes:	[Considération_Riposte_Population]

No memos

**Répondant** : par rapport à la riposte je dirais qu’au départ nous ne cesserons pas de le dire, le départ était mal organisé lorsque on avait installé le programme de riposte. Il y avait eu parfois l’implication de gens qu’on ne connaissaient pas dans la communauté soit on te connait comme un monsieur très buveur il vient nous sensibiliser sur cette maladie c’est là où il y avait une petite mésentente entre la communauté et les gens qui sont venus dans les différentes séances de sensibilisation et de communication au sein de la riposte

**P37: IA_04_AS_IT_02.rtf - 37:10 [Répondant : Selon la populatio..] (25:25)	(Super)**

Codes:	[Considération_Riposte_Population]

No memos

**Répondant** : Selon la population elle disait que c’est un montage car on n’aller recruter tout juste au début de gens de la communauté. Là une femme avec de pantalon dans notre culture une ne peut pas portée, vous venez à la sensibilisation avec un pantalon ton message est à l’écart.

**P37: IA_04_AS_IT_02.rtf - 37:11 [Répondant : tout justes au déb..] (27:27)	(Super)**

Codes:	[Structures-Services_fonctionnement]

No memos

**Répondant** : tout justes au début de l’épidémie les activités de santé de routine surtout le curatif a était fréquenter vu en flamber depuis les différents épisodes de la maladie. Deuxièmement est survenue la gratuité des soins, lorsqu’elle s’est installée on a eu submergeance au sein des fosas par les populations qui venaient fréquentées les soins au sein de centre de santé. Du point de vue la CPS, elle a marché chez nous d’une façon normale malgré que l’épidémie était là, les femmes nous amènent les enfants sans inquiétude car ces sont les mêmes personnes qui s’occupaient de parent de CPS et ce sont eux qui ont continué avec la même activité ainsi que la CPN je dirais que chez moi dans mon aire de santé je n’ai pas eu beaucoup des déviations de femmes pour ces activités ici ça, c’est direct d’une façon générale d’ailleurs pour le service de santé dans l’aire de santé.

**P37: IA_04_AS_IT_02.rtf - 37:12 [Répondant : Par la fuite dans ..] (29:29)	(Super)**

Codes:	[Structure-Service_Utilisation_population]

No memos

**Répondant** : Par la fuite dans les structures, pourquoi les malades avaient fuie les structures de santé. Chez moi il y avait certains qui avaient peur de centre de sante lorsqu’ils ont vu le CTE qui était construit dans mon aire de santé, le CTE de MABHOLO il est dans mon aire de santé ils ont cris que avant on embarquer les gens à ITAVE et si vous partez la bas vous risque de mourir a ITAVE Dou ils ont cris que le CTE c’est un mouroir , car ils avait peu qui guérissait beaucoup décédaient mais ceux-là qui sont un peu habitués à nous dans mon fosa continuaient toujours à nous fréquenter malgré la peur d’être emportés dans l’ambulance vers le CTE

**P37: IA_04_AS_IT_02.rtf - 37:13 [Répondant : A ce qui est de la..] (33:33)	(Super)**

Codes:	[Qualité_soins_Prestataires]

No memos

**Répondant** : A ce qui est de la qualité, je dirais que l’accueil que nous les avions réservé avant l’épidémie on a continue avec le même accueil pendant l’épidémie c’est pourquoi les malades sont venu se faire soigner toujours avec le même accueil qu’on avait avant l’épidémie ça va continuer les malades vraiment tout le monde était suspect mais on a continué avec ce même accueil au sein de mon fosa.

**P37: IA_04_AS_IT_02.rtf - 37:14 [Répondant : Le triage on a com..] (35:35)	(Super)**

Codes:	[Triage_Considération_population]

No memos

**Répondant** : Le triage on a commencé par un pré triage, le triage aussi est venue mais malgré le triage ce qui nous a aider aussi un peu le centre de sante à proximité de ce CH, le CH aussi on avait intégré un triage, ils partaient au CH et rencontraient le triage, au centre de santé ils vont rencontrer aussi un triage ; pour chez nous a ce qui est de la peur du triage ça n’a pas posé de problème.

**P37: IA_04_AS_IT_02.rtf - 37:15 [Répondant : Pour le lavage de ..] (37:37)	(Super)**

Codes:	[Triage-Isolement-Chlore-thermoflash_Réaction_population]

No memos

**Répondant** : Pour le lavage de main, je dirais que on a accepté surtout juste au début on avait une insuffisance de récipient pour le lavage de main, ce n’est qu’après qu’on nous avait donné un partenaire, qui nous a appuyé, on a pu éparpiller le lavage de main au sein de la structure, sauf c’est qui avait inquiété les gens c’était le service de la vaccination de ceinture. C’était la vaccination a ceinture pour le contact, si vous n’êtes pas en contact tu étais écarté d’où la population qui entoure le centre de santé, beaucoup son de voyageur qui partent ils voulaient le vaccin mais arrivant au niveau de service de vaccination vous n’êtes pas en contact vous ne serez pas enregistré, c’est là ou pouvait nous amener en incompréhension entre le centre de santé et les équipes de riposte, pourquoi suite au refus de la non vaccination ils veulent le vaccin mais ils sont refusés soit disant ils ne sont pas sur la liste c’est ça ce qui a était chez moi comme un point négatif

**P37: IA_04_AS_IT_02.rtf - 37:16 [Répondant : Pendant la période..] (39:39)	(Super)**

Codes:	[Structures-Services_fonctionnement]

No memos

**Répondant** : Pendant la période de l’Ebola au sein du centre de santé, le service qui n’ont pas bien fonctionnes nous avons d’abord le laboratoire ; ça n’a pas bien fonctionne et les soins ambulatoires, pourquoi par ce qu’on disait qu’il ne faut plus faire saigné le malade car vous ne savez pas qui est porteur du virus et qui ne l’est pas, le laboratoire vraiment na pas bien marche. Le deuxième service qui n’a pas marcher au sein du centre de sante c’était la maternité, vraiment la population avait déjà peur pour la maternité, mais pour les observations il y n’avait pas eu de problème sauf le laboratoire. Il ne faut plus saigner les malades hors si tu ne lui pique pas au dos pour prendre le globule et aujourd’hui ils disent je ne pas fait le test, le médecin n’a pas pris le sang pourquoi par ce que on l’a pas fait saigner ce qui était interdit pour le programme de la riposte.

**P37: IA_04_AS_IT_02.rtf - 37:17 [Répondant : Dans mon aire de s..] (41:41)	(Super)**

Codes:	[Structure-Service_Fermeture_pdt MVE]

No memos

**Répondant** : Dans mon aire de santé il y avait eu deux structures qui avaient fermées la porte craignant le cas de l’épidémie et la première structure c’est une structure qui était à proximité elle avait fermé soit disant je ne veux pas contaminer elle avait fermé jusqu’à maintenant il n’a pas encore ouvert sa structure.

**P37: IA_04_AS_IT_02.rtf - 37:18 [Répondant : Mais oui il est da..] (43:44)	(Super)**

Codes:	[Structure-Service_Fermeture_pdt MVE]

No memos

**Répondant** : Mais oui il est dans le milieu toujours ?

**Répondant** : L’autre structure aussi, elle avait fermé, je dirais pour elle ce n’était pas le problème de l’Ebola, elle avait un problème du personel, son personnel avait eu peur vu le fonctionnement de cette FOSA on risque de se faire contaminer ici ils ont fui en ayant peur de la contamination.

**P37: IA_04_AS_IT_02.rtf - 37:19 [Répondant : Dans d’autre struc..] (46:46)	(Super)**

Codes:	[Ressources_humaines_Disponibilité et perturbations]

No memos

**Répondant** : Dans d’autre structure dans mon aire de santé, j’ai d’autre structure où d’autres agents ont été recruté par exemple au CTE mais ils se sont arranger les structures ont fonctionnées d’une façon normale par exemple au centre de santé j’avais deux agents qui on était recruter au niveau de CTE pour dire le recrutement des agents au sein des activités de la riposte n’ont pas perturbé les activités au sein du FOSA

**P37: IA_04_AS_IT_02.rtf - 37:20 [Répondant : Ça n’a pas affecté..] (48:48)	(Super)**

Codes:	[Ressources_humaines_Disponibilité et perturbations]

No memos

**Répondant** : Ça n’a pas affecté

**P37: IA_04_AS_IT_02.rtf - 37:21 [Répondant : Ça n’a pas posé pr..] (50:50)	(Super)**

Codes:	[Ressources_humaines_Disponibilité et perturbations]

No memos

**Répondant** : Ça n’a pas posé problème, pourquoi au CTE quand même il arrive de temps qui était prévue un peu des horaires qui étaient bien programme

**P37: IA_04_AS_IT_02.rtf - 37:22 [Répondant : Oui nous étions co..] (52:52)	(Super)**

Codes:	[Ressources_humaines_situation_grève]

No memos

**Répondant** : Oui nous étions concernés

**P37: IA_04_AS_IT_02.rtf - 37:23 [Répondant : La grève c’était a..] (54:54)	(Super)**

Codes:	[Ressources_humaines_situation_grève]

No memos

**Répondant** : La grève c’était avant la proclamation de l’épidémie juin juillet 2018, c’est là où on avait eu la grève de professionnel de la sante surtout les activités qui étaient concernées au sein de cette grève c’était le rapportage de tous le rapport MAPEPI et mensuel. La deuxième activité c’était la suspension de la vaccination, ce sont les deux activités qui étaient concernées par la grève des professionnels de santé avant qu’on annonce les deux classements de l’épidémie

**P37: IA_04_AS_IT_02.rtf - 37:24 [épondant : Avant l’Ebola le mo..] (56:56)	(Super)**

Codes:	[Recouvrement_coûts-des-soins_avant gratuité]

No memos

**épondant** : Avant l’Ebola le mode de financement était l’autofinancement par la population

**P37: IA_04_AS_IT_02.rtf - 37:25 [Répondant : Le système de tari..] (58:58)	(Super)**

Codes:	[Recouvrement_coûts-des-soins_avant gratuité]

No memos

**Répondant** : Le système de tarification au sein de la population n’était pas forfaitaire c’était selon le service rendu qu’on a rendu à la population par acte

**P37: IA_04_AS_IT_02.rtf - 37:26 [Répondant : Dans l’aire de san..] (60:60)	(Super)**

Codes:	[Recouvrement_coûts-des-soins_mutuelle]

No memos

**Répondant** : Dans l’aire de santé il n’y avait pas de mutuel

**P37: IA_04_AS_IT_02.rtf - 37:27 [Répondant : OK pour la gratuit..] (64:64)	(Super)**

Codes:	[Gratuité_Structures-Services_concernés]

No memos

**Répondant** : OK pour la gratuite le premier contrat au sein du centre de santé a commencé tout le paquet de centre de santé qui veut dire consultation, CPS, CPN accouchement était dans la gratuité. Le 2ieme contrat a fait tomber la CPS, l’accouchement et la CPN d’où les malades venaient payes la CPN, CPS et l’accouchement, mais pour le curatif on avait maintenu cette même tarification. 3ème contrat s’est épuiser d’ici récemment et à continuer dans la même phase au centre de santé devrait payer les consultations curatives, on voit la consultation, le laboratoire et les médicaments mais on a laissé l’accouchement, la CPN et la CPS que les dames devraient payer

**P37: IA_04_AS_IT_02.rtf - 37:28 [Répondant : Le moyen de commun..] (66:66)	(Super)**

Codes:	[Gratuité_Information_population]

No memos

**Répondant** : Le moyen de communication avons-nous d’abord le CAC qui est installé on le communiqué et à leur tour communique la population. On a aussi les réunions de comité de santé qui son représentant de la population ils les informent et le malade viendra sachant que la gratuité est là, on les forme aussi pendant les séances de CPN, CPS.

**P37: IA_04_AS_IT_02.rtf - 37:29 [Répondant : La gratuité que no..] (68:68)	(Super)**

Codes:	[Gratuité_Interruption]

No memos

**Répondant** : La gratuité que nous avons maintenant est un contrat qui était signé en daté du 16 février au 30 juin 2020

**P37: IA_04_AS_IT_02.rtf - 37:30 [Répondant : Apres le 30 juin o..] (70:70)	(Super)**

Codes:	[Gratuité_Interruption]

No memos

**Répondant** : Apres le 30 juin on ne sait pas ceux qui entreprennent vont gérer comment.

**P37: IA_04_AS_IT_02.rtf - 37:31 [Répondant : Il n’avait pas les..] (72:72)	(Super)**

Codes:	[Gratuité_Rumeurs]

No memos

**Répondant** : Il n’avait pas les rumeurs autour de la gratuité car le premier mois du début on pouvait avoir cent malades par jour, pour la communauté la gratuité était un salut. Ils disaient depuis notre naissance nous n’avons jamais vécu la période de la gratuité des soins. C’est comme aujourd’hui avec la gratuité de l’enseignement, aujourd’hui c’est un salut pour certains. Ils s’étonnaient en disant DAWA YA BURE qui signifie les soins gratuits ? suite aux factures non recouvrées et non payées.

**P37: IA_04_AS_IT_02.rtf - 37:32 [Répondant : La gratuite a étai..] (74:74)	(Super)**

Codes:	[Gratuité_Partenaire d'appui]

No memos

**Répondant** : La gratuite a était appuyée par PDCC

**P37: IA_04_AS_IT_02.rtf - 37:33 [Répondant : La gratuite je dir..] (76:76)	(Super)**

Codes:	[Gratuité_Conséquences]

No memos

**Répondant** : La gratuite je dirais d’abord positives comme j’ai annoncé là, les structures ont été fréquentées c’était positive, négative c’est quand on a commencé le 1^er^ janvier lorsque on a dit qu’il y a plus gratuite la population était déjà habituée à cette gratuite la d’où lorsqu’on a quitté la gratuite au payement des soins il y a eu une insolvabilité qui a était très remarque par la population au sein de la FOSA

**P37: IA_04_AS_IT_02.rtf - 37:34 [Répondant : Là je dirais que l..] (78:78)	(Super)**

Codes:	[Gratuité_Conséquences_motivation_prestataire]

No memos

**Répondant** : Là je dirais que la motivation était meilleure, pourquoi les consultations ont augmenté on devrait aussi payer le tarif dans le contact par rapport aux consultations qu’on a émis je dirais que la motivation a était améliorée.

**P37: IA_04_AS_IT_02.rtf - 37:35 [Répondant : Oui] (80:80)	(Super)**

Codes:	[Gratuité_Accueil_population]

No memos

**Répondant** : Oui

**P37: IA_04_AS_IT_02.rtf - 37:36 [Répondant : Avant pendant cett..] (82:82)	(Super)**

Codes:	[Médicaments_disponibilité_fosa]

No memos

**Répondant** : Avant pendant cette période de gratuite la disponibilité de médicament a était très difficile, pourquoi le contrat qu’on avait signé était que ASSRAMES devrait nous fournir le médicament mais chose qui n’a pas été faite je dirais depuis qu’on a commencé la gratuite on a déjà réussi seulement deux livraisons, quelle quantité de médicament ca on n’en parle pas, de 2 la dernière livraison qu’on a eu chez ASRAMES selon ont le contrat car selon le contrat c’est ASRAMES qui devrait nous fournir le médicament au structures, on imposée leur commande après imposition de commande, mais le commande n’avait pas était respecter, non-respect de commande par le FOSA

**P37: IA_04_AS_IT_02.rtf - 37:37 [Répondant : Non-respect de com..] (84:84)	(Super)**

Codes:	[Médicaments_disponibilité_fosa]

No memos

**Répondant** : Non-respect de commande, vous commandez deux aspirines ou bien une boite de bendazole il te servait la moitié hors ils n’ont pas la statistique de malade de consultation. Par rapport à la commande de médicament il y avait une surfacturation de médicament chez ASRAMES par rapport au marché local, même voir triple par rapport le peu de médicament qui sont arrivés on a surfacturation ça c’est ce qui a était dans l’indicateur du point de vue

**P37: IA_04_AS_IT_02.rtf - 37:38 [Répondant : Ça a affecté l’off..] (86:86)	(Super)**

Codes:	[Médicaments_disponibilité_fosa]

No memos

**Répondant** : Ça a affecté l’offre du service suite à la rupture du stock de médicament

**P37: IA_04_AS_IT_02.rtf - 37:39 [Répondant : Le système d’infor..] (88:88)	(Super)**

Codes:	[SNIS_fonctionnement_Decrire_pdt MVE]

No memos

**Répondant** : Le système d’information sanitaire je dirais que chez moi ça n’a pas eu de perturbation pourquoi toutes les données on était remportée en temps opportun sur tous les canevas de SNIS malgré que l’épidémie q était ça n’a pas perturbé la récolte des informations sanitaire sauf là où ça cause une petite perturbation c’était au niveau de CH privé qui sont dans nos aires de sante on a eu de difficulté a récolté leur donnée pendant la période de l’épidémie

**P37: IA_04_AS_IT_02.rtf - 37:40 [Répondant : C’est qui a laissé..] (90:90)	(Super)**

Codes:	[SNIS_fonctionnement_Decrire_pdt MVE]

No memos

**Répondant** : C’est qui a laissé le centre de santé dans la gratuite les structures type CH comme je viens de le dire sont privées et les malades sont leur capitale d’où pour livre leur formation on croyait que on irait récupérer leur malade pour les apporter par exemple au niveau de CTE pas car ils avaient de tendance a casher les malade par ce que si je ferais mon recette sera chiffre d ou pour rapporte les données aussi c’était vraiment un petit défis pendant la période qu’on a traversée pour le FOSA type CH prive

**P37: IA_04_AS_IT_02.rtf - 37:41 [Répondant : Pour le rapportage..] (92:92)	(Super)**

Codes:	[SNIS_fonctionnement_Decrire_pdt MVE]

No memos

**Répondant** : Pour le rapportage de données je me suis disponibilité étant que responsable de récolté de donne au sein de centre de sante je me suis disponibilitsé pour que les données soient présent, complétées on devrait s’assoir avec le type de centre de santé pour faire le récolte de donne chacun a ce qui et son activité qui lui est attribué au sein de la FOSA et d’analyser réussi les différentes données récoltées dans notre FS car dans car dans notre zone de sante il est demandé chaque recette au moins tous les centre de santé ayant déposé son rapport au sein du BCZS d’ où entre le deux et le cinq on devrait se force chacun ace qui lui concerne de copuler ces données dans ces différents branches pour la CPN on centralise la bas et la CPS de l’autre côté ainsi de suite.

**P37: IA_04_AS_IT_02.rtf - 37:42 [Répondant : Comme je vous lais..] (94:94)	(Super)**

Codes:	[SNIS_fonctionnement_Decrire_pdt MVE]

No memos

**Répondant** : Comme je vous lais dis que chez moi il n’y avait pas eu beaucoup de gens qui étaient dans la riposte, moi je faisais la surveillance étant au niveau du centre de santé, je ne déplaçais pas je faisais par fois de sorties et si on a une petite résistance cela que je sortais

**P37: IA_04_AS_IT_02.rtf - 37:43 [Répondant : La tenue de réunio..] (96:96)	(Super)**

Codes:	[Réunion_pdt-épidémie-Ebola]

No memos

**Répondant** : La tenue de réunion vraiment nous avons remplie toutes les réunions pendant la période de l’épidémie avec le comité de santé et disait vraiment il ne pensait pas rate à aucune réunion malgré que les activités étaient là de sensibilisation on avait pris certains membres en comite de santé dans le suivi de contact, dans le base RDC mais il était forcé aussi à faire leur assistance à la fin de chaque mois selon leur calendrier prévu

**P37: IA_04_AS_IT_02.rtf - 37:44 [Répondant : Nous avons étaient..] (98:98)	(Super)**

Codes:	[Gouvernance_soins santé]

No memos

**Répondant** : Nous avons étaient appuyés, je dirais que leur appui nous a permis de faire l’avancement dans les différentes étapes qui nous sont arrivées dans la riposte, par exemple il y avait des ONG qui étaient venues nous appuies au niveau de lave main qu’on devrait doter au niveau des écoles, au niveau des églises, ça nous a permis aussi une sensibilisation pour le lave main. Mais affaire éducation lave main ne vous amener même pas un matériel vous chanter ils sont venus nous aider à former les jeunes dans le cadre de EDS, ça aussi a permis de réduire

**P37: IA_04_AS_IT_02.rtf - 37:45 [Répondant : Enterrement digne ..] (100:100)	(Super)**

Codes:	[Gouvernance_soins santé]

No memos

**Répondant** : Enterrement digne et sécurisé, a permis aussi une sensibilisation au sein de la population ,soit disant c’est l’enfant de tel qui lui a enterré, c’est ne plus l expatrier qui lui a enterré, car aussi la partie de l’enterrement avait causé beaucoup de problème pour faire le soibe, dans beaucoup d’aires de santé c’est là où il y avait aussi le début de jette de pierre sur les équipes, les experts ils sont arrivé dans l’ère de sante lorsqu’ils faisaient de terrain par exemple là où il y avait beaucoup de décès dans la communauté, c’est là où il y avait de coup de machette. Lorsqu’il voyait de difficulté on les accompagner pour avis de conscientiser les familles ou bien la population qui était enflammée

**P37: IA_04_AS_IT_02.rtf - 37:46 [Répondant : Ça n’a pas était r..] (102:102)	(Super)**

Codes:	[Gouvernance_soins santé]

No memos

**Répondant** : Ça n’a pas était réduite

**P37: IA_04_AS_IT_02.rtf - 37:47 [Répondant : Moi je recommander..] (106:106)	(Super)**

Codes:	[Souhait_Changement_gestion_futures_épidémies]

No memos

**Répondant** : Moi je recommanderais si une autre épidémie arrivée quelque part il faut d’abord : Associer d’abord les acteurs locaux en vue de vaincre toutes les résistances, non pas nous amener les superviseurs d ou par exemple est venue Charles on lui ne connait pas ici, pour dire que chaque culture accepte ces rites, comme il est ici chez nous l’enterrement se fait comme ça, ici pour partir au soin tu dois partir à pieds vous vous amenez une ambulance est ce qu’on a besoin d’une ambulance, on part à pieds car c’est notre culture. Pour dire s’il y a une autre épidémie il faut d’abord que les acteurs proviennent de la base.

**P37: IA_04_AS_IT_02.rtf - 37:48 [Répondant : Il faut d’abord co..] (108:108)	(Super)**

Codes:	[Souhait_Changement_gestion_futures_épidémies]

No memos

**Répondant** : Il faut d’abord consulter la base avant tous les acteurs, les principaux acteurs peuvent être au plafond mais d’abord, il faut savoir quelles sont les rites d’ici, la base d’abord.

**P37: IA_04_AS_IT_02.rtf - 37:49 [Répondant : Moi, je Je dirais ..] (112:112)	(Super)**

Codes:	[Répondant_Responsabilité_pdt MVE]

No memos

**Répondant** : Moi, je Je dirais que nous étions associés car le 1^er^ cas on nous avait réuni dans cette salle ici dès le départ on savait déjà là où on aller avec l’épidémie.

**P37: IA_04_AS_IT_02.rtf - 37:50 [Répondant : Je dirais socialem..] (114:114)	(Super)**

Codes:	[Changements_depuis_Ebola]

No memos

**Répondant** : Je dirais socialement Ebola a réveillé l’esprit de lavage de main, de mesure hygiène. D’une façon générale l’épidémie a amenée d’autres mentalités malgré qu’elles ne sont pas encore bien assimiler qui est enterrement avec sac mortier.

**P37: IA_04_AS_IT_02.rtf - 37:51 [Répondant : Ça se fait pour to..] (116:116)	(Super)**

Codes:	[Changements_depuis_Ebola]

No memos

**Répondant** : Ça se fait pour tous les décès négatifs que positifs on utilisait le sac mortier

**P37: IA_04_AS_IT_02.rtf - 37:52 [Répondant : Dans mon ère de sa..] (118:118)	(Super)**

Codes:	[Changements_depuis_Ebola]

No memos

**Répondant** : Dans mon ère de santé je ne pas encore eu le cas pour voir si on a encore utilisé le sac mortier. Mais si c’est un décès communautaire ça continue toujours, Ebola a éveillé aussi du point de vue enterrement le sac mortier, dans le même cadre l’EDS pour ce qui est de l’enterrement aussi l’équipe de l’EDS a facilité certaines familles à l’octroi des cercueils

**P37: IA_04_AS_IT_02.rtf - 37:53 [Répondant : Les relations entr..] (120:120)	(Super)**

Codes:	[Changements_depuis_Ebola]

No memos

**Répondant** : Les relations entre les hommes, il y avait relation entre les hommes malgré de figure, de visage étrange ou pas ils sont entre de comprendre que c’est la normalité qui peut se faire dans la vie soit dans la communauté, nous croyons s’il y a une autre épidémie qui pouvait survenir dans le futur la génération d’aujourd’hui dont nous sommes pouvait encore dire, on avait vu ce si car histoire ravale le passer

**P37: IA_04_AS_IT_02.rtf - 37:54 [Répondant : Oui c’était verbal..] (122:122)	(Super)**

Codes:	[Instructions_pdt_MVE]

No memos

**Répondant** : Oui c’était verbale.

**P38: IA_04_AS_IT_03.rtf - 38:1 [Répondant : Je suis un titulai..] (9:9)	(Super)**

Codes:	[Répondant_Responsabilité_habituelles]

No memos

**Répondant** : Je suis un titulaire adjoint dans l’aire de sante de Rughenda, mon travail est de coordonner les activités de l’aire de santé

**P38: IA_04_AS_IT_03.rtf - 38:2 [Répondant : Coordonner des dif..] (11:11)	(Super)**

Codes:	[Répondant_Responsabilité_habituelles]

No memos

**Répondant** : Coordonner des différents rapports qui arrivent on le sépare puis on fait un rapport qu’on va envoyer à l’unité centrale

**P38: IA_04_AS_IT_03.rtf - 38:3 [Répondant : Il n’y a pas] (13:13)	(Super)**

Codes:	[Répondant_Responsabilité_habituelles]

No memos

**Répondant** : Il n’y a pas

**P38: IA_04_AS_IT_03.rtf - 38:4 [Répondant : Souvent c’est la c..] (15:15)	(Super)**

Codes:	[Répondant_Responsabilité_habituelles]

No memos

**Répondant** : Souvent c’est la consultation, et de là on passe pour des formations, des réunions etc… alors je fais peu de temps au centre de santé

**P38: IA_04_AS_IT_03.rtf - 38:5 [Répondant : L’Ebola est entre ..] (17:17)	(Super)**

Codes:	[Début-Progression_Epidémie MVE]

No memos

**Répondant** : L’Ebola est entre ici, on a eu des malades et parmi ces malades il y a eu un enfant venu à la poste de bulumbi pour arriver à l’hôpital général de katwa, l’enfant a était récupéré et après on lui a transféré à l’hôpital ou il est mort. Au lieu que ses parents partent à l’hôpital de Katwa ils sont venus ici ou l’on a remarqué que l’enfant était un cas probable par ce que quand la famille de l’enfant était testée tous étaient positif, c’est comme ça que la maladie est entrée ici et c’était le 14 novembre 2018.

**P38: IA_04_AS_IT_03.rtf - 38:6 [Répondant : Ça continue Jusqu’..] (19:19)	(Super)**

Codes:	[Début-Progression_Epidémie MVE]

No memos

**Répondant** : Ça continue Jusqu’on mois de mai, en avril ici il y avait trop de brouis au point qu’il y avait risque qu’un médecin perd sa vie, puis l’IT est partie pendant 3 mois par ce qu’il était incapable d’arriver à la structure.

**P38: IA_04_AS_IT_03.rtf - 38:7 [Répondant : Par ce que le band..] (21:21)	(Super)**

Codes:	[Agression-Menaces_Structure-Prestataire_raisons]

No memos

**Répondant** : Par ce que le bandit lui filet chez lui à la maison pour l’assassine et cela a deux reprise, les bandits étaient main armée c’est pourquoi IT a décidé de partir et il a fait 3 mois d’absence a la structure.

**P38: IA_04_AS_IT_03.rtf - 38:8 [Répondant : Moi j’étais à Buni..] (23:23)	(Super)**

Codes:	[Agression-Menaces_Structure-Prestataire_raisons]

No memos

**Répondant** : Moi j’étais à Bunia en refuge

**P38: IA_04_AS_IT_03.rtf - 38:9 [Répondant : Je suis ici depuis..] (25:25)	(Super)**

Codes:	[Agression-Menaces_Structure-Prestataire_raisons]

No memos

**Répondant** : Je suis ici depuis juillet 2018.

**P38: IA_04_AS_IT_03.rtf - 38:10 [Répondant : non j’étais à la f..] (27:27)	(Super)**

Codes:	[Agression-Menaces_Structure-Prestataire_raisons]

No memos

**Répondant** : non j’étais à la famille je ne travaille plus.

**P38: IA_04_AS_IT_03.rtf - 38:11 [Répondant : J’étais ici, d’ail..] (33:33)	(Super)**

Codes:	[Répondant_Responsabilité_habituelles]

No memos

**Répondant** : J’étais ici, d’ailleurs il y a trois consultations qui sont passé dans le bureau et je suis là, on les a hospitalisés

**P38: IA_04_AS_IT_03.rtf - 38:12 [Répondant : La population disa..] (37:37)	(Super)**

Codes:	[Considération_Ebola_population]

No memos

**Répondant** : La population disait que Ebola c’est quelque chose qu’on a amené ce pourquoi elle ne considérée pas ça comme une maladie

**P38: IA_04_AS_IT_03.rtf - 38:13 [Répondant : Ils disaient qu’ E..] (39:39)	(Super)**

Codes:	[Considération_Ebola_population]

No memos

**Répondant** : Ils disaient qu’ Ebola c’est quelque chose qu’on a amené c’est pourquoi ils n’ont pas accepter que c’est une maladie, que c’est quelque chose qu’on a fabriqué, injecter pour amener ca a la population c’est pourquoi les gens n’ont pas compris, il y a même certains politiciens qui disaient que c’est pas une maladie que c’est quelque chose genre virus qu’on a implanté c’est pourquoi il y a eu une grande résistance qui a fait à ce que plusieurs médecins puisse fuir et il y a eu de structures qui ont fermées de peur d’être menacer par la population

**P38: IA_04_AS_IT_03.rtf - 38:14 [Répondant : Ils ont menaçaient..] (41:41)	(Super)**

Codes:	[Agression-Menaces_Structure-Prestataire_raisons]

No memos

**Répondant** : Ils ont menaçaient soit-disant que nous avons amené ici la maladie pour que nous puissions gagner de l’argent

**P38: IA_04_AS_IT_03.rtf - 38:15 [Répondant : Nous avons déménag..] (43:43)	(Super)**

Codes:	[Agression-Menaces_Structure-Prestataire_raisons]

No memos

**Répondant** : Nous avons déménagé avec nos familles

**P38: IA_04_AS_IT_03.rtf - 38:16 [Répondant : Oui il y a d’autre..] (45:45)	(Super)**

Codes:	[Agression-Menaces_Structure-Prestataire_raisons]

No memos

**Répondant** : Oui il y a d’autres incendiés avec les matériels

**P38: IA_04_AS_IT_03.rtf - 38:17 [Répondant : Ici non il y avait..] (47:47)	(Super)**

Codes:	[Agression-Menaces_Structure-Prestataire_raisons]

No memos

**Répondant** : Ici non il y avait seulement des gens qui me poursuivaient chez moi alors que la distance qui me sépare avec le centre est de 14 kilomètre mais ils venaient jusque chez moi et c’est à cause de ça que j’avais déménagé

**P38: IA_04_AS_IT_03.rtf - 38:18 [Répondant : Oui ils sont ferme..] (49:49)	(Super)**

Codes:	[Structure-Service_Fermeture_pdt MVE]

No memos

**Répondant** : Oui ils sont fermes

**P38: IA_04_AS_IT_03.rtf - 38:19 [Répondant : D’autre ont fermés..] (51:51)	(Super)**

Codes:	[Structure-Service_Fermeture_pdt MVE]

No memos

**Répondant** : D’autre ont fermés surtout pour les médecins qui avaient peur de la contamination et pour ça qu’ils sont fermés et pour le menace

**P38: IA_04_AS_IT_03.rtf - 38:20 [Répondant : Il y a certains se..] (53:53)	(Super)**

Codes:	[Structure-Service_Fermeture_pdt MVE]

No memos

**Répondant** : Il y a certains services qui ont fonctionnes et d’autre n’ont pas fonctionnes

**P38: IA_04_AS_IT_03.rtf - 38:21 [Répondant : Certains services ..] (55:55)	(Super)**

Codes:	[Structures-Services_fonctionnement]

No memos

**Répondant** : Certains services qui n’ont pas fonctionnaient c’est sont les services qui œuvrent dans les activités préventives comme par exemple la vaccination, la CPS, et si tu amènes un enfant là-bas on va lui soumettre au vaccin d’Ebola, eux pensaient que dans quelque mois les gens vont mourir, ils ont évacuaient les enfants sérieusement puis le taux de la vaccination a baissé aussi sérieusement dalleurs il y a de fois où on ferme le site et on se trouve dans l’incapacité d’aller se présenter là-bas de peur que la population ne puisse te tue

**P38: IA_04_AS_IT_03.rtf - 38:22 [Répondant : Aujourd’hui ils co..] (57:57)	(Super)**

Codes:	[Structure-Service_Utilisation_population]

No memos

**Répondant** : Aujourd’hui ils commencent à venir, et nous écouté surtout avec la présence du covid 19 il commence à voir que peut être c’était normal

**P38: IA_04_AS_IT_03.rtf - 38:23 [Répondant : Comme les visites ..] (59:59)	(Super)**

Codes:	[Considération_RECO_depuis MVE]

No memos

**Répondant** : Comme les visites du domicile qu’on n’avait pas fait pour quoi par ce que quand tu arrives dans le quartier le gens disent encore les médecins sont là et lorsque vous arrivez dans une parcelle il commence à dire qu’encore vous venez leur donnée des comprimes qui contiennent le virus Ébola donc pour eux la présence d’un médecin c’est un problème raison pour laquelle on ne faisait pas de visite à domicile.

**P38: IA_04_AS_IT_03.rtf - 38:24 [Répondant : Ils sont tous part..] (61:61)	(Super)**

Codes:	[Service_maternité_fonctionnement]

No memos

**Répondant** : Ils sont tous partis il y avait plus le service d’accouchement.

**P38: IA_04_AS_IT_03.rtf - 38:25 [Répondant : Je ne saurais pas ..] (63:63)	(Super)**

Codes:	[Service_maternité_fonctionnement]

No memos

**Répondant** : Je ne saurais pas répondre à cette question mais pour l’accouchement on a vue personne même a l’hôpital général, se possible que la CPN on ne le faisait plus car les femmes disaient que lorsqu’on est enceinte pour un petit vomissement on t’envoie à la CTE c’est pour quoi elles ont eu peur, mais on ne sait pas si elles ont accouché après mais d’autres commencer à accoucher

**P38: IA_04_AS_IT_03.rtf - 38:26 [Répondant : Que les gens sont ..] (65:65)	(Super)**

Codes:	[Considération_CTE_population]

No memos

**Répondant** : Que les gens sont entre de mourir là-bas eux pensaient que la CTE c’est un mouroir, une fois tu es la tu es un homme mort ce vrai juste pour sortir à la CTE c’était une chance c’est pour quoi après les menaces et conversation entre nous on ne sait pas sils ont amélioré ou comment par ce que au début c’était grave.

**P38: IA_04_AS_IT_03.rtf - 38:27 [Répondant : Seulement au nivea..] (67:67)	(Super)**

Codes:	[Structure-Service_Utilisation_population]

No memos

**Répondant** : Seulement au niveau de service curatif par ce que le service curatif était vraiment bien fréquenter surtout avec la gratuite.

**P38: IA_04_AS_IT_03.rtf - 38:28 [Répondant : Oui nous en avons] (69:69)	(Super)**

Codes:	[Structures-Services_fonctionnement]

No memos

**Répondant** : Oui nous en avons

**P38: IA_04_AS_IT_03.rtf - 38:29 [Répondant : Pendant cette péri..] (71:71)	(Super)**

Codes:	[Structures-Services_fonctionnement]

No memos

**Répondant** : Pendant cette période ces gens vannaient juste pour prendre le médicament et prélever le cracha puis on amène ça, mais pour dire que la personne parte à la CTE ça vraiment c’était un problème, mais ici au niveau local tout se passait bien mais le gens avait été disant qu’on va leur soumettre le vaccin de l’Ebola sans le savoir.

**P38: IA_04_AS_IT_03.rtf - 38:30 [Répondant : Oui] (73:73)	(Super)**

Codes:	[Triage_Considération_population]

No memos

**Répondant** : Oui

**P38: IA_04_AS_IT_03.rtf - 38:31 [Répondant : Dalleurs ce servic..] (75:75)	(Super)**

Codes:	[Triage_Considération_population]

No memos

**Répondant** : Dalleurs ce service à faillir être incendié mais nous avons eu grâce par ce qu’il est tout près d’un camp militaire c’est ce qui a épargné le service si non ce service devrait être incendié.

**P38: IA_04_AS_IT_03.rtf - 38:32 [Répondant : Je ne sais pas mai..] (77:77)	(Super)**

Codes:	[Triage_Considération_population]

No memos

**Répondant** : Je ne sais pas mais le gens n’aimait pas ça, vraiment je ne suis pas encore informé pour savoir pourquoi les gens n’aiment pas ce service hors c’était une maison en planche mais nous avons étaient étonner, pourquoi c’est là où il y a eu trop de menace par ce qu’il y avait triage on avait un sérieux problème.

**P38: IA_04_AS_IT_03.rtf - 38:33 [Répondant : Bon s’ils n’ont pa..] (79:79)	(Super)**

Codes:	[Triage_Considération_population]

No memos

**Répondant** : Bon s’ils n’ont pas compris le triage, par ce que c’était un endroit où on prenait le gens après la sélection pour que celui qui va partir à la CTE ne va plus passer ici alors ils ont commencé à dire que c’est dans cette maison ou l’on capture les gens.

**P38: IA_04_AS_IT_03.rtf - 38:34 [Répondant : Bons la qualité de..] (81:81)	(Super)**

Codes:	[Qualité_soins_Prestataires]

No memos

**Répondant** : Bons la qualité de soin avant Ebola, on n’avait pas le médicament comme au début de l’épidémie, par ce que quand on a commencé on nous a donné un stock suffisant de médicament. Tout juste au début de l’Ebola on avait une quantité suffisante de médicament et les gens venaient, alors si la qualité na pas répondue c’est par ce que les gens étaient nombreux par rapport aux médecins par ce que on arrivait à 50 à 60 consultations par jour et ça c’est déjà un bénéfice pour les agents da la riposte, par ce que les malades ont augmentés par rapport à d’autres jours. Alors si la qualité n’a pas répondu c’est par ce que les malades étaient nombreux c’est pourquoi arriver à 60 on était déjà fatiguer mais avant Ebola on avait pas le médicament ni l’argent et le malade ne venaient pas nombreux.

**P38: IA_04_AS_IT_03.rtf - 38:35 [Répondant : La gratuite est en..] (85:85)	(Super)**

Codes:	[Gratuité_Début]

No memos

**Répondant** : La gratuite est entrée pour que nous trouvions les malades, pour que les gens ne puissent plus mourir par manque de moyen pour paye le soin alors pour que les malades soient là il fallait qui est gratuite pour éviter qu’il n’y eu pas prétexte que je ne pas de l’argent et ce vraie les malades venaient nombreux.

**P38: IA_04_AS_IT_03.rtf - 38:36 [Répondant : Le médicament et l..] (87:87)	(Super)**

Codes:	[Gratuité_Structures-Services_concernés]

No memos

**Répondant** : Le médicament et la consultation

**P38: IA_04_AS_IT_03.rtf - 38:37 [Répondant : Bon on début c’éta..] (89:89)	(Super)**

Codes:	[Gratuité_Structures-Services_concernés]

No memos

**Répondant** : Bon on début c’était comme ça puis on a changé le contrat par ce que la maternité était gratuite et les gens venaient pour cette gratuite. Et puis pendant la période qu’on a changé l’autre contrat les gens ne venaient plus nombreux mais avec le soin curatif au moins par rapport avant la riposte.

**P38: IA_04_AS_IT_03.rtf - 38:38 [Répondant : On faisait la fact..] (91:91)	(Super)**

Codes:	[Recouvrement_coûts-des-soins_avant gratuité]

No memos

**Répondant** : On faisait la facturation par rapport au médicament qu’on donner au patient

**P38: IA_04_AS_IT_03.rtf - 38:39 [Répondant : Oui] (93:93)	(Super)**

Codes:	[Recouvrement_coûts-des-soins_avant gratuité]

No memos

**Répondant** : Oui

**P38: IA_04_AS_IT_03.rtf - 38:40 [Répondant : Presque nous tous...] (95:95)	(Super)**

Codes:	[Répondant_Responsabilité_pdt MVE]

No memos

**Répondant** : Presque nous tous.

**P38: IA_04_AS_IT_03.rtf - 38:41 [Répondant : Au début j’ai comm..] (97:97)	(Super)**

Codes:	[Répondant_Responsabilité_pdt MVE]

No memos

**Répondant** : Au début j’ai commencé par le suivi de contact, 2iemement je suis partis dans la recherche active et cela ou je suis jusqu’aujourd’hui.

**P38: IA_04_AS_IT_03.rtf - 38:42 [Répondant : Probablement par c..] (99:99)	(Super)**

Codes:	[Ressources_humaines_Disponibilité et perturbations]

No memos

**Répondant** : Probablement par ce que on avait beaucoup de travail et beaucoup de contact, et lorsque on faisait de suivi aux malades le matin automatiquement il y avait retard au travail c- à- d on n’arrivait pas à 8heures.

**P38: IA_04_AS_IT_03.rtf - 38:43 [Répondant : Bons il n’y a pas ..] (101:101)	(Super)**

Codes:	[Ressources_humaines_Disponibilité et perturbations]

No memos

**Répondant** : Bons il n’y a pas un grand problème seulement pour les malades qui disaient qu’on le fait trainer au centre rien que ça.

**P38: IA_04_AS_IT_03.rtf - 38:44 [Répondant : Ils deviennent nom..] (103:103)	(Super)**

Codes:	[Ressources_humaines_Disponibilité et perturbations]

No memos

**Répondant** : Ils deviennent nombreux et les médecins arrivent à retard alors celui qui est là le matin reste submerger lui seul.

**P38: IA_04_AS_IT_03.rtf - 38:45 [Répondant : Oui oui] (105:105)	(Super)**

Codes:	[Ressources_humaines_situation_grève]

No memos

**Répondant** : Oui oui

**P38: IA_04_AS_IT_03.rtf - 38:46 [Répondant : Oui] (107:107)	(Super)**

Codes:	[Ressources_humaines_situation_grève]

No memos

**Répondant** : Oui

**P38: IA_04_AS_IT_03.rtf - 38:47 [Répondant : A peu près trois m..] (109:109)	(Super)**

Codes:	[Ressources_humaines_situation_grève]

No memos

**Répondant** : A peu près trois mois on était en grave.

**P38: IA_04_AS_IT_03.rtf - 38:48 [Répondant : Oui on était en gr..] (111:111)	(Super)**

Codes:	[Ressources_humaines_situation_grève]

No memos

**Répondant** : Oui on était en grève puis on a levé ça à cause de l’épidémie

**P38: IA_04_AS_IT_03.rtf - 38:49 [Interviewer : Alors qu’est-ce ..] (112:113)	(Super)**

Codes:	[Ressources_humaines_conditions_travail]

No memos

**Interviewer : Alors qu’est-ce que vous pouvez dire pour les conditions dans lesquelles vous travaillez pendant la période de l’Ebola ?**

1459 R c’était de conditions difficiles, surtout les menaces par ce que il avait trop de travail à notre ère de santé, nous sommes aux environ de 58 décès positifs avec 3 cas probables c’est pour quoi le cas avait augmenter puis on avait refusé de se comprendre, de s’attendre nous avec la population ça n’a pas marcher c’est pour quoi on avait ici la peur par ce que ceux qui étaient ici s’en fermer dans la clôture mais pour dire tu vas venir à ciel ouvert c’était très difficile, tu arrives ici tu portes le habit que tu es venu avec à la maison de façon que le menacer peuvent vous confondre avec la population ils ne saura pas qui est médecin et qui ne l’ait, pas par ce que le menaceur venaient en groupe et on n ignorerai Dou venait ce groupe c’est pour quoi on était ici dans la peur.

**P38: IA_04_AS_IT_03.rtf - 38:50 [Répondant : Nous avons des EPI..] (115:115)	(Super)**

Codes:	[Prestataire_Pratique de Prévention contre MVE]

No memos

**Répondant** : Nous avons des EPI dote par l’PPSP

**P38: IA_04_AS_IT_03.rtf - 38:51 [Répondant : Il y avait déjà de..] (117:117)	(Super)**

Codes:	[Prestataire_Pratique de Prévention contre MVE]

No memos

**Répondant** : Il y avait déjà de formation, on était déjà formé

**P38: IA_04_AS_IT_03.rtf - 38:52 [Répondant : La peur d’Ebola es..] (119:119)	(Super)**

Codes:	[Prestataire_Pratique de Prévention contre MVE]

No memos

**Répondant** : La peur d’Ebola est finie

**P38: IA_04_AS_IT_03.rtf - 38:53 [Répondant : Du point de vue po..] (121:121)	(Super)**

Codes:	[Gratuité_Conséquences]

No memos

**Répondant** : Du point de vue positif avec la gratuite la prime à augmenter, nous avons eu beaucoup de cas et les factures ont augmentées puis les médecins se sont rétablies un peu par rapport avant.

**P38: IA_04_AS_IT_03.rtf - 38:54 [Répondant : Autre je ne sais p..] (123:123)	(Super)**

Codes:	[Gratuité_Conséquences]

No memos

**Répondant** : Autre je ne sais pas si je peux dire que c’est un avantage a la riposte par ce que on n’avait pas ici de toilette, des incinérateurs et des plysiomes mais nous en avons eu à cause de l’Ebola.

**P38: IA_04_AS_IT_03.rtf - 38:55 [Répondant : Ils savaient par c..] (125:125)	(Super)**

Codes:	[Gratuité_Information_population]

No memos

**Répondant** : Ils savaient par ce que on partait dire à l’Eglise qu’il y a gratuite

**P38: IA_04_AS_IT_03.rtf - 38:56 [Répondant : A peu près 4 contr..] (127:127)	(Super)**

Codes:	[Gratuité_Evolution]

No memos

**Répondant** : A peu près 4 contrats

**P38: IA_04_AS_IT_03.rtf - 38:57 [Répondant : Non, le premier mo..] (129:129)	(Super)**

Codes:	[Gratuité_Evolution]

No memos

**Répondant** : Non, le premier moi après changement de contrat on était là mais il y avait diminution de malades mais au fur et à mesure que les malades qui venaient ils disaient aux autres.

**P38: IA_04_AS_IT_03.rtf - 38:58 [Répondant : Avant Ebola on ach..] (131:131)	(Super)**

Codes:	[Médicaments_disponibilité_fosa]

No memos

**Répondant** : Avant Ebola on achetait une petite quantité de médicament, on chercher le médicament en fonction de moyen financier qu’on avait mais les malades ne payaient pas et ils étaient moins nombreux avant la riposte et le médicament n’était pas suffisant pour le nombre de malades d’ailleurs juste au début que la gratuite était bien par ce que le médicament était suffisant et les malades aussi étaient nombreux on n’avait pas de rupture mais pendant cette période il commençait à se fatigue.

**P38: IA_04_AS_IT_03.rtf - 38:59 [Répondant : Les gens qui nous ..] (133:133)	(Super)**

Codes:	[Médicaments_disponibilité_fosa]

No memos

**Répondant** : Les gens qui nous donnaient le médicament, comme aujourd’hui on n’a pas le médicament on n’a qu’un seul antibiotique.

**P38: IA_04_AS_IT_03.rtf - 38:60 [Répondant : Pendant la période..] (135:135)	(Super)**

Codes:	[SNIS_fonctionnement_Decrire_pdt MVE]

No memos

**Répondant** : Pendant la période de l’Ebola c’était difficile de le faire par ce que il y avait la tension ou la journée se passe sans le présence même d’une seule personne ici à cause de menace ce pour quoi pour dire vraiment qu’il y avait une réalité par ce que il y avait de jour qu’on écrivait rien dans le registre et cet jour la a mafifi ne va pas travaille normalement par ce que il y aura de donnée passée sans être enregistre et finir aussi au travaille puis on a vu que qu’il y avait trop de fatigue par ce que on arrivait 1500 malades le mois alors pour combiner tout ça, il nous ait demander d’avoir le temps et beaucoup de force et le malades sont la a même temps, on est entre de compléter, vraiment le faible ne pouvaient pas manqué par ce que le travail devient beaucoup et le rapport nous attend, on était entre de le faire en étant fatigues.

**P38: IA_04_AS_IT_03.rtf - 38:61 [Répondant : Je dirais peut-êtr..] (137:137)	(Super)**

Codes:	[SNIS_fonctionnement_Decrire_pdt MVE]

No memos

**Répondant** : Je dirais peut-être la qualité, mais pour la promptitude on était entre de se forcer quand les malades deviennent nombreux tu commences à faire la consultation en état fatiguer je ne sais pas vraiment la réalité, heureusement ils étaient guéris

**P38: IA_04_AS_IT_03.rtf - 38:62 [Répondant : Les réunions étaie..] (141:141)	(Super)**

Codes:	[Réunion_pdt-épidémie-Ebola]

No memos

**Répondant** : Les réunions étaient régulières avant Ebola mais pendant Ebola il y a de période où on a tout stoppé à cause de menace mais aujourd’hui comme la menace diminue on a récupéré le même horaire normal

**P38: IA_04_AS_IT_03.rtf - 38:63 [Répondant : Bon, moi c’est mon..] (143:143)	(Super)**

Codes:	[Répondant_Responsabilité_pdt MVE_Implication]

No memos

**Répondant** : Bon, moi c’est mon travail bien qu’ils avaient menacé mais aussi je ne voulais pas laisser mais je laisse mon travail à cause de menace mais les autres personnes disaient on va mourir pour quoi et ils ont décidé de laisse à cause de menace

**P38: IA_04_AS_IT_03.rtf - 38:64 [Répondant : Moi j’avais commen..] (145:145)	(Super)**

Codes:	[Répondant_Responsabilité_pdt MVE_Depuis quand ?]

No memos

**Répondant** : Moi j’avais commencé en novembre

**P38: IA_04_AS_IT_03.rtf - 38:65 [Répondant : Ebola est entre ic..] (147:147)	(Super)**

Codes:	[Début-Progression_Epidémie MVE]

No memos

**Répondant** : Ebola est entre ici le 14 novembre 2018

**P38: IA_04_AS_IT_03.rtf - 38:66 [Répondant : C’était rare] (149:149)	(Super)**

Codes:	[Répondant_Responsabilité_pdt MVE_Implication]

No memos

**Répondant** : C’était rare

**P38: IA_04_AS_IT_03.rtf - 38:67 [Répondant : non j’étais ici av..] (151:151)	(Super)**

Codes:	[Gouvernance_soins santé]

No memos

**Répondant** : non j’étais ici avec le gens calme et modérer

**P38: IA_04_AS_IT_03.rtf - 38:68 [Répondant : Non il n’y a pas e..] (153:153)	(Super)**

Codes:	[Gouvernance_soins santé]

No memos

**Répondant** : Non il n’y a pas eu de changement

**P38: IA_04_AS_IT_03.rtf - 38:69 [Répondant : Le changement que ..] (155:155)	(Super)**

Codes:	[Souhait_Changement_gestion_futures_épidémies]

No memos

**Répondant** : Le changement que j’aurais souhaite et que le locaux soient nombreux par rapport aux acteurs étrangers, par ce que permis les étrangers certains ne maitrise pas bien le langues par exemple swahili mais eux savaient lingala et français hors la population d’ici ne maitrise même pas bien swahili alors que les étrangers viennent parler en français et lingala en créant un problème d’incompréhension, alors que la population dit soit disant qu’on les a vendue alors si une autre épidémie arrive il faut que le locaux soient plus nombreux sur terrain. Même si les étrangers peuvent être nombreux dans la coordination et occuper le bureau là ça va mais sur le terrain on doit avoir la présence de locaux par ce que c’est eux qui vont sensibiliser la population qui est en surface

**P38: IA_04_AS_IT_03.rtf - 38:70 [Répondant : Autre chose, si un..] (157:158)	(Super)**

Codes:	[Souhait_Changement_gestion_futures_épidémies]

No memos

**Répondant** : Autre chose, si une autre épidémie arrive ça sera presque la même chose par ce que nos structures sont inadaptées, ils étaient entre de refuse qu’on se met dans la consultation qui occupe une chambrette qui ne peut pas bien accueil deux personne c’est pourquoi il faut qu’ils construisent d’abord les structures comme ça s’il y a un malade qu’il ne puisse pas contaminer l’autre par ce que dans une chambre on garde 3 personnes alors s’il y a un malade il doit contaminer le 2 autres c’est pourquoi il faut il faut qu’ils construisent bien les structures.

**Répondant** : C’est qui a changer ce quoi, les gens ont compris que beaucoup des maladies arrivent par ce que les mains sont salle , même aujourd’hui les gens ont compris qu’ils ne doivent pas toucher n’importe comment un cadavre par ce que tout cadavre est devenu un problème , même la maladie ils sont compris que les malades aussi sont contagieuse, même touche un malade, le parent ont compris que même dans la maison un enfant peut avoir une fièvre mais personne ne va le touche de peur que ça ne puisse pas être contagieuse, ni le médecin aussi c’est la même chose par ce qu’il sait que si un malade arrive ce que il y a un problème.

**P38: IA_04_AS_IT_03.rtf - 38:71 [Répondant : Aujourd’hui cela n..] (160:160)	(Super)**

Codes:	[Prestataire_Pratique de Prévention contre MVE]

No memos

**Répondant** : Aujourd’hui cela n’existe pas, même une plaie on doit porter le gant

**P38: IA_04_AS_IT_03.rtf - 38:72 [Répondant : C’est ce qui pose ..] (162:162)	(Super)**

Codes:	[Prestataire_Pratique de Prévention contre MVE]

No memos

**Répondant** : C’est ce qui pose problème, lorsque tu pars porter le gant les malades disent que on se souci plus deux cela a poser problème même toucher à un malade par ce que on ne pas habituer a porté le gant avant de soigner alors tu commences a porté le gant le malade te dit si tu lui vois comme il commence a parlé beaucoup et a pensés beaucoup que tu lui néglige par ce que tu portes le gant et quand tu termines tu te laves le main ce qui n’était pas dans notre culture on faisait comme ça aujourd’hui, mais au moins aujourd’hui par ce que il a eu un changement

**P38: IA_04_AS_IT_03.rtf - 38:73 [Répondant : Si ça diminue mais..] (164:164)	(Super)**

Codes:	[Prestataire_Pratique de Prévention contre MVE]

No memos

**Répondant** : Si ça diminue mais ça va rester à une grande partie

**P38: IA_04_AS_IT_03.rtf - 38:74 [Répondant : Normalement ici ch..] (166:166)	(Super)**

Codes:	[PCI_Comment_maintenir]

No memos

**Répondant** : Normalement ici chez nous comme aujourd’hui s’il y a plus gratuite on va revenir à la même pauvreté qu’on avait avant pour dire qu’on peut trouver les épis il n’y a pas moyen, ça ne sera pas possible ce pourquoi nous demandons de dotation aux hôpitaux à chaque moment avec ça on peut réussir mais si on nous laisse on va arriver à une certaine période ou on sera fatigué par ce qu’il y a pas de financement

**P38: IA_04_AS_IT_03.rtf - 38:75 [Répondant : Et l’argent qu’il ..] (168:168)	(Super)**

Codes:	[Commentaire-Recommandation_Répondant]

No memos

**Répondant** : Et l’argent qu’il donne aux prestataires, que l’Etat paye les prestataires mais j’ai 30 ans de service sans numéro matricule, sans prime de risque et lorsque les malades viennent comme ça on va mourir pour rien, nos enfants restent de pauvres par ce qu’on ne gagne rien il faut que nous soyons paye que l’Etat se force pour nous paye

**P39: IA_04_AS_PCOSA_03.rtf - 39:1 [Répondant : Je m’occupe de rel..] (10:10)	(Super)**

Codes:	[Répondant_Responsabilité_habituelles]

No memos

**Répondant** : Je m’occupe de relais communautaire

**P39: IA_04_AS_PCOSA_03.rtf - 39:2 [Répondant : Le relais communau..] (12:12)	(Super)**

Codes:	[Répondant_Responsabilité_habituelles]

No memos

**Répondant** : Le relais communautaire veut dire avoir une relation avec la population et voir comment est leur état de santé, voir la propreté et s’il y a un malade je l’amène à l’hôpital.

**P39: IA_04_AS_PCOSA_03.rtf - 39:3 [Répondant : Oui] (14:14)	(Super)**

Codes:	[Répondant_Responsabilité_habituelles]

No memos

**Répondant** : Oui

**P39: IA_04_AS_PCOSA_03.rtf - 39:4 [Répondant : Si Ebola est entré..] (16:16)	(Super)**

Codes:	[Début-Progression_Epidémie MVE]

No memos

**Répondant** : Si Ebola est entré c’est par ce qu’il y a des visiteurs qui sont venus avec.

**P39: IA_04_AS_PCOSA_03.rtf - 39:5 [Répondant : Bon c’était un hom..] (18:18)	(Super)**

Codes:	[Début-Progression_Epidémie MVE]

No memos

**Répondant** : Bon c’était un homme qui est venu faire un mariage coutumier a la famille de sa fiancée qui est ici a KITULU et c’est cet homme qui a fait entrer Ebola ici.

**P39: IA_04_AS_PCOSA_03.rtf - 39:6 [Répondant : Quand il était de ..] (20:20)	(Super)**

Codes:	[Début-Progression_Epidémie MVE]

No memos

**Répondant** : Quand il était de retour à Beni il a fait des examens et c’est après qu’on a constaté que la personne a quittée Beni en étant déjà contaminée par l’Ebola alors que là où ils faisaient leur cérémonie de remise de dote c’est là où on a dit vacciner tout celui qui à participer, on a passé à la zone de santé pour une formation chez le docteur pour suivre tous ces gens.

**P39: IA_04_AS_PCOSA_03.rtf - 39:7 [Répondant : Non il y a eu seul..] (22:22)	(Super)**

Codes:	[Début-Progression_Epidémie MVE]

No memos

**Répondant** : Non il y a eu seulement un seul cas, mais les autres sont venus après alors que le premier cas était déjà.

**P39: IA_04_AS_PCOSA_03.rtf - 39:8 [Répondant : Seulement les visi..] (24:24)	(Super)**

Codes:	[Début-Progression_Epidémie MVE]

No memos

**Répondant** : Seulement les visiteurs qui sont venus dans d’autres endroits, on le trouvait dans des dispensaires et d’autres dans des postes de santé.

**P39: IA_04_AS_PCOSA_03.rtf - 39:9 [Répondant : Dans la riposte j’..] (26:26)	(Super)**

Codes:	[Répondant_Responsabilité_pdt MVE]

No memos

**Répondant** : Dans la riposte j’étais donc là pour suivre le contact.

**P39: IA_04_AS_PCOSA_03.rtf - 39:10 [Répondant : Ils ont dit que c’..] (30:30)	(Super)**

Codes:	[Considération_Ebola_population]

No memos

**Répondant** : Ils ont dit que c’est quelque chose qu’on a créé mais on envoi a la population, même nous le relais communautaire on était attaqué on n’avait pas de relation avec les gens, j’entre ici seulement par ce que c’est mon travail.

**P39: IA_04_AS_PCOSA_03.rtf - 39:11 [Répondant : Oui] (32:32)	(Super)**

Codes:	[Considération_Ebola_population]

No memos

**Répondant** : Oui

**P39: IA_04_AS_PCOSA_03.rtf - 39:12 [Répondant : Ils disaient que c..] (34:34)	(Super)**

Codes:	[Considération_Ebola_population]

No memos

**Répondant** : Ils disaient que c’est le blanc.

**P39: IA_04_AS_PCOSA_03.rtf - 39:13 [Répondant : Ils disaient surto..] (36:36)	(Super)**

Codes:	[Considération_Ebola_population]

No memos

**Répondant** : Ils disaient surtout que nous les Nandes nous sommes **très** peuple qu’on diminue

**P39: IA_04_AS_PCOSA_03.rtf - 39:14 [Répondant : Les gens de la rip..] (38:38)	(Super)**

Codes:	[Considération_Riposte_Population]

No memos

**Répondant** : Les gens de la riposte n’ont pas été les bienvenus à la population par ce qu’ils circulaient avec de voiture et de fois on le jeter de pierre pour les menaces de rentre chez eux hors s’ils rentrent avant de faire leur travail, Ebola va nous attaquer jusqu’à ce que nous allons finir.

**P39: IA_04_AS_PCOSA_03.rtf - 39:15 [Répondant : Oui dalleurs moi d..] (40:40)	(Super)**

Codes:	[Agression-Menaces_Structure-Prestataire_raisons]

No memos

**Répondant** : Oui dalleurs moi dans mon quartier quand j’ai circulé, ils disaient que c’est nous qui profite de cette argent de la riposte.

**P39: IA_04_AS_PCOSA_03.rtf - 39:16 [Répondant : Oui] (42:42)	(Super)**

Codes:	[Agression-Menaces_Structure-Prestataire_raisons]

No memos

**Répondant** : Oui

**P39: IA_04_AS_PCOSA_03.rtf - 39:17 [Répondant : Non ils ne peuvent..] (44:44)	(Super)**

Codes:	[Agression-Menaces_Structure-Prestataire_raisons]

No memos

**Répondant** : Non ils ne peuvent plus m’attaqué.

**P39: IA_04_AS_PCOSA_03.rtf - 39:18 [Répondant : Oui] (46:46)	(Super)**

Codes:	[Structures-Services_fonctionnement]

No memos

**Répondant** : Oui

**P39: IA_04_AS_PCOSA_03.rtf - 39:19 [Répondant : Il y n’avait pas d..] (50:50)	(Super)**

Codes:	[Structures-Services_fonctionnement]

No memos

**Répondant** : Il y n’avait pas de problème, c’est l’Ebola qui était comme un problème qui est entré dans la population qui se demande si c’est quoi, on a dit l’Ebola avait commencé depuis avant comme par exemple à l’équateur et puis ça arrivé ici.

**P39: IA_04_AS_PCOSA_03.rtf - 39:20 [Répondant : Oui mais d’autres ..] (52:52)	(Super)**

Codes:	[Service_vaccination_Fonctionnement]

No memos

**Répondant** : Oui mais d’autres avaient peur, quand Ebola est entré les uns ont dit n’ont dit non il se peut qu’ils vont nous vacciner pour que nous puissions mourir on a dit non, moi j’ai étais vacciné mais je ne suis pas mort.

**P39: IA_04_AS_PCOSA_03.rtf - 39:21 [Répondant : Tous n’ont pas acc..] (54:54)	(Super)**

Codes:	[Service_vaccination_Fonctionnement]

No memos

**Répondant** : Tous n’ont pas acceptés, d’autres avaient peur par ce qu’ici il y avait le vaccin de l’Ebola, peur de centre aussi, on disait non ne pas peur venez

**P39: IA_04_AS_PCOSA_03.rtf - 39:22 [Répondant : Non ça n’a pas pos..] (60:60)	(Super)**

Codes:	[Triage_Considération_population]

No memos

**Répondant** : Non ça n’a pas posé beaucoup problème, nous les avons sensibilisés comment venir ici à l’hôpital, tu commences à entrer ici on t’écrit après avoir écrire la fiche tu pars a un endroit pour le soin.

**P39: IA_04_AS_PCOSA_03.rtf - 39:23 [Répondant : Oui ça n’existait ..] (58:58)	(Super)**

Codes:	[Triage_Considération_population]

No memos

**Répondant** : Oui ça n’existait pas.

**P39: IA_04_AS_PCOSA_03.rtf - 39:24 [Répondant : Non ils avaient pe..] (62:62)	(Super)**

Codes:	[Triage_Considération_population]

No memos

**Répondant** : Non ils avaient peur

**P39: IA_04_AS_PCOSA_03.rtf - 39:25 [Répondant : C’était seulement ..] (64:64)	(Super)**

Codes:	[Triage_Considération_population]

No memos

**Répondant** : C’était seulement à cause de l’Ebola, par ce qu’avec l’Ebola on peut aller à la CTE et d’autres ne quittent pas la bas vivant et on disait que c’est la maladie qui fait à ce qu’il soit mort, on en a attendu qu’ils sont entrés de piquer les gens le seringue directement tu es mort et fin de fin ils ont compris qu’une personne peut y aller et rentre en état guérie.

**P39: IA_04_AS_PCOSA_03.rtf - 39:26 [Répondant : Oui] (66:66)	(Super)**

Codes:	[Triage_Considération_population]

No memos

**Répondant** : Oui

**P39: IA_04_AS_PCOSA_03.rtf - 39:27 [Répondant : Oui] (68:68)	(Super)**

Codes:	[Triage_Considération_population]

No memos

**Répondant** : Oui

**P39: IA_04_AS_PCOSA_03.rtf - 39:28 [Répondant : Ici chez nous ils ..] (70:70)	(Super)**

Codes:	[Triage-Isolement-Chlore-thermoflash_Réaction_population]

No memos

**Répondant** : Ici chez nous ils posaient de questions de savoir pourquoi nous avons placé de lave mains qui a de l’eau qui contient le chlore, on a dit que c’est le chlore qu’ils utilisaient avant qu’on achète à KEIB on met le chlore dans l’eau pour enlever le microbe, alors pour quoi cette eau à une odeur, on a dit que c’est par ce qu’elle contient le chlore.

**P39: IA_04_AS_PCOSA_03.rtf - 39:29 [Répondant : Pour accueil des m..] (72:72)	(Super)**

Codes:	[Qualité_Soins_Patients]

No memos

**Répondant** : Pour accueil des malades à d’autres centres de santé et surtout à MUTSANGA les gens avaient peur par ce qu’il y avait beaucoup de décès alors pour accueillir un malade c’était un grand problème mais ici chez nous on avait dit au gens de ne pas peur parce que c’est une maladie comme tant d’autre, comme on peut mourir d’une autre maladie c’est la même chose que Ebola.

**P39: IA_04_AS_PCOSA_03.rtf - 39:30 [Répondant : Problème d’accueil..] (74:74)	(Super)**

Codes:	[Qualité_Soins_Patients]

No memos

**Répondant** : Problème d’accueillir les malades, le problème est qu’ils avaient peur soit disant que lorsqu’ils se rendent au centre on leur donne un médicament qui n’existe pas presque pour causer leur mort sans savoir que le médicament pour Ebola c’est un médicament comme tant d’autre

**P39: IA_04_AS_PCOSA_03.rtf - 39:31 [Répondant : Ok l’accueil de ma..] (76:76)	(Super)**

Codes:	[Qualité_Soins_Patients]

No memos

**Répondant** : Ok l’accueil de malade.

**P39: IA_04_AS_PCOSA_03.rtf - 39:32 [Répondant : Bon a ce qui me co..] (78:78)	(Super)**

Codes:	[Qualité_Soins_Patients]

No memos

**Répondant** : Bon a ce qui me concerne moi, sur mon centre de santé, les malades disaient que les prestataires ne les ont pas bien accueillis et moi je disais que si les médecins ne vous a pas bien accueilli c’est par ce que peut être ils ont beaucoup de temps ici et le fait de changer les médecins, les nouveaux pourrons peut être bien vous accueillir

**P39: IA_04_AS_PCOSA_03.rtf - 39:33 [Répondant : Lorsque les malade..] (80:80)	(Super)**

Codes:	[Qualité_Soins_Patients]

No memos

**Répondant** : Lorsque les malades arrivent, on le fait beaucoup trainer pour être soigner

**P39: IA_04_AS_PCOSA_03.rtf - 39:34 [Répondant : Ils disaient qu’il..] (82:82)	(Super)**

Codes:	[Qualité_Soins_Patients]

No memos

**Répondant** : Ils disaient qu’il y a permis les médecins qui le répondaient mal

**P39: IA_04_AS_PCOSA_03.rtf - 39:35 [Répondant : Ici aussi c’est la..] (84:84)	(Super)**

Codes:	[Prestataire_Attitude_devant_malade]

No memos

**Répondant** : Ici aussi c’est la même chose, ils disaient que les médecins ne le touchent plus à cause de cette maladie, et lorsqu’ils portaient de gants ils commencent à dire pour quoi de gants alors qu’avant vous nous soignez sans pour autant porter de gants, puis on le disait que c’est cette maladie qui fait à ce que nous puissions portaient le gant.

**P39: IA_04_AS_PCOSA_03.rtf - 39:36 [Répondant : La tenue que porta..] (86:86)	(Super)**

Codes:	[Considération_prestataire_EPI_population]

No memos

**Répondant** : La tenue que portaient les médecins avant de soigner et de masque ça faisait la peur aux malades et les gens se demandaient.

**P39: IA_04_AS_PCOSA_03.rtf - 39:37 [Répondant : Oui] (88:88)	(Super)**

Codes:	[Considération_prestataire_EPI_population]

No memos

**Répondant** : Oui

**P39: IA_04_AS_PCOSA_03.rtf - 39:38 [Répondant : Ils étaient bien s..] (90:90)	(Super)**

Codes:	[Traitement_Invasifs_Prestataire_changement]

No memos

**Répondant** : Ils étaient bien soignés et quand on a dit que le médicament est gratuit ils ont commencé à dire ce gens vont nous terminer.

**P39: IA_04_AS_PCOSA_03.rtf - 39:39 [Répondant : Les uns ont bien r..] (94:94)	(Super)**

Codes:	[Gratuité_Accueil_population]

No memos

**Répondant** : Les uns ont bien réussi et les autres avec difficultés par ce que lors de sensibilisation on disait que si tu tombes malade tu pars à l’hôpital par ce que les médicaments sont gratuits et c’est le gouvernement qui vas payer et puis je dis, moi quand j’étais à l’école on recevait de soin gratuit, on te fait un timbre tu apportes ça au parent et le parent le garde, et ça va être payé au niveau de l’état après ils commencent à nous écouter.

**P39: IA_04_AS_PCOSA_03.rtf - 39:40 [Répondant : Bon les médecins, ..] (96:96)	(Super)**

Codes:	[Ressources_humaines_Disponibilité et perturbations]

No memos

**Répondant** : Bon les médecins, je veux dire la vérité pendant la période de l’Ebola à cause de l’argent ils étaient entrés dans la riposte, ils arrivent ici à retard soit 10h à 11h par ce qu’ils avaient suit le suivi de contact et puis je dis le centre va rester comme ça et eux disent qu’est-ce qu’on gagnés là-bas, et puis je dis bon ça va passer et après vous allez revenir à votre travail.

**P39: IA_04_AS_PCOSA_03.rtf - 39:41 [Répondant : Pendant la période..] (98:98)	(Super)**

Codes:	[Réunion_pdt-épidémie-Ebola]

No memos

**Répondant** : Pendant la période de l’Ebola les gens de mon comité ne sont pas entré dans la riposte tous étaient partis je suis reste avec peu de gens et on faisait de réunions soit disant Ebola c’est une maladie comme les autres on a sensibilisé les gens comment se lavé les mains que la propreté soit la première chose.

**P39: IA_04_AS_PCOSA_03.rtf - 39:42 [Répondant : Oui j’ai a augment..] (102:103)	(Super)**

Codes:	[Commentaire-Recommandation_Répondant]

No memos

**Répondant** : Oui j’ai a augmenté, bon c’est nous qui avons commencé cet hôpital on était de locateur on a fourni tout le moyen possible avec le défunt président, le peu qu’on avait on est parti demander un crédit à la banque on a vendu quelque bien qu’on avait après on a achète une parcelle et construire, quand on a achète cette parcelle il y avait cette maison et l’autre mais lorsque le gouvernement envoi de l’argent les gens de la zone disent que cet argent ne pas à vous laisser les infirmiers gérés cet argent, et cet argent nous on avait rien eu donc j’ai signé pour rien sans pour autant me donner même un franc ce que le travail que je fais et gratuit et je me suis dit non, donc c’est le gouvernement qui saura comment me faire déloger ici pour ce travail que je fais a la population.

**Interviewe**

**P39: IA_04_AS_PCOSA_03.rtf - 39:43 [Répondant : Oui ils recevaient..] (104:104)	(Super)**

Codes:	[Médicaments_disponibilité_fosa]

No memos

**Répondant** : Oui ils recevaient le médicament

**P39: IA_04_AS_PCOSA_03.rtf - 39:44 [Répondant : Oui lorsqu’il n’y ..] (106:106)	(Super)**

Codes:	[Médicaments_disponibilité_fosa]

No memos

**Répondant** : Oui lorsqu’il n’y a pas le médicament, on fait tout le moyen et on dit au malade d’aller acheter le médicament qu’il doit prendre pour la maladie qu’il a

**P39: IA_04_AS_PCOSA_03.rtf - 39:45 [Répondant : Non] (108:108)	(Super)**

Codes:	[Médicaments_disponibilité_fosa]

No memos

**Répondant** : Non

**P39: IA_04_AS_PCOSA_03.rtf - 39:46 [Répondant : Bon, comme corona ..] (110:110)	(Super)**

Codes:	[Souhait_Changement_gestion_futures_épidémies]

No memos

**Répondant** : Bon, comme corona est déjà arrivé au Congo je peux dire aux gens que la propreté est très important par ce que sans la propreté toute ces maladies on peut les attrapées tu vois comme on a fermé des églises, des écoles ce à cause de ça, il faut faire attention et évités de prendre une conversation avec une personne pendant beaucoup de temps alors ils commencent à avoir peur par ce que le virus lui circule, ils ont déjà remarquer que c’est une maladie qui tue mais s’ils trouvent le médicament ils seront guéris.

**P40: IA_04_BCZS_IS_01.docx - 40:1 [Répondant : Moi je suis infirm..] (8:8)	(Super)**

Codes:	[Répondant_Responsabilité_habituelles]

No memos

**Répondant** : Moi je suis infirmière superviseur de la zone de santé et je m’occupe du travail de vaccin(Chikato) et surveillance épidémiologique à la zone de santé de Katwa

**P40: IA_04_BCZS_IS_01.docx - 40:2 [Répondant : En bref je fais de..] (10:10)	(Super)**

Codes:	[Répondant_Responsabilité_habituelles]

No memos

**Répondant** : En bref je fais des supervisions dans des aires de santé donc nous sommes trois superviseurs dans l’équipe cadre la zone est divisée en cinq axes donc y compris le DN et le médecin directeur de l’HGR c’est comme ça que nous divisons la zone de santé et chacun a les aires de santé qu’il supervise, donc moi aussi j’ai des aires de santé que je supervise

**P40: IA_04_BCZS_IS_01.docx - 40:3 [Répondant : J’ai 5 aires de sa..] (12:12)	(Super)**

Codes:	[Répondant_Responsabilité_habituelles]

No memos

**Répondant** : J’ai 5 aires de santé.

**P40: IA_04_BCZS_IS_01.docx - 40:4 [Répondant : Lorsque j’arrive l..] (14:14)	(Super)**

Codes:	[Répondant_Responsabilité_habituelles]

No memos

**Répondant** : Lorsque j’arrive le matin je vérifie si le frigo fonctionne bien et je sers aussi le vaccin à ceux-là qui partent vacciner les enfants après cela si j’ai un rapport à faire je fais ça, comme à la fin du mois je dois centraliser le rapport du PV et chaque lundi je centralise le rapport de mapipi et mardi matin après centralisation j’envoie le rapport à Goma

**P40: IA_04_BCZS_IS_01.docx - 40:5 [Répondant : Oui c’est le trava..] (16:16)	(Super)**

Codes:	[Répondant_Responsabilité_habituelles]

No memos

**Répondant** : Oui c’est le travail que je fais chaque matin par ce qu’après je peux maintenant aller au terrain pour faire la recherche active et je supervise ce qui font la recherche active au terrain à ce qui concerne la maladie à virus Ebola.

**P40: IA_04_BCZS_IS_01.docx - 40:6 [Répondant : Et l’épidémie d’Eb..] (18:18)	(Super)**

Codes:	[Début-Progression_Epidémie MVE]

No memos

**Répondant** : Et l’épidémie d’Ebola est entré dans la zone sans que personne ne soit au courant par ce que le système de surveillance était corrompu puisque nous étions en grève il avait l’argent qu’on avait pas réussi pour prime de risque nous médecins, infirmiers administratif à cause de ça on a fait une grève alors la maladie aussi est venue et elle avait commencée dans la zone de Mabalako les cas passaient inaperçu par ce que il y avait pas de rapportage les gens n’étaient pas informé alors ont étaient surpris qu’ont étaient déjà dans l’épidémie sans le savoir puis nous avons suspendu la grève et nous avons entre dans le reportage normal pour l’épidémie.

**P40: IA_04_BCZS_IS_01.docx - 40:7 [Répondant : A la zone de santé..] (20:20)	(Super)**

Codes:	[Début-Progression_Epidémie MVE]

No memos

**Répondant** : A la zone de santé il y avait un cas et c’était une maman qui était à Mangina puis elle est venue dans la zone de santé de wayene et elle répondait à la définition alors elle s’est cachée dans la communauté lorsqu’elle avait trouvé qu’il y a alerté elle se rendue dans l’aire de santé de masole pour se cacher alors on l’avait fait quitter là-bas pour l’amené au CTE malheureusement la maman était morte et la maladie à commencer dans la zone.

**P40: IA_04_BCZS_IS_01.docx - 40:8 [Répondant : C’est 1er cas à dé..] (22:22)	(Super)**

Codes:	[Début-Progression_Epidémie MVE]

No memos

**Répondant** : C’est 1^er^ cas à dégénérer beaucoup de cas dans l’aire de santé de wayene, ça régénère de cas dans l’aire de santé de wasuli et dans l’aire de santé de Mutsanga par ce que ils sont à proximité et tu sais cette maladie la maman était manipuler par le particulier et le membre de sa famille et ce qui a fait à ce que la maladie devienne très grave dans notre zone de santé par ce que ils disaient que il y a pas la maladie et chose drôle elle était dans la famille d’un député et ce dépité disait qu’il y a pas la maladie ailleurs c’est celui qui était parti ravir la maman là pour l’amener

**P40: IA_04_BCZS_IS_01.docx - 40:9 [Répondant : Dans sa famille wa..] (24:24)	(Super)**

Codes:	[Début-Progression_Epidémie MVE]

No memos

**Répondant** : Dans sa famille wayene pour l’amener dans le quartier binongo derrière l’hôpital puis après le même député avait pris sa tente qui est la maman pour l’amener au CTE à Butembo et cela que le dépité était entre de déclarer qu’il n’y a pas la maladie et quand on avait fait la vaccination autour de ce cas, il était parti se faire vacciner en cachette et il a dit de ne pas lui prendre de photos et encore il l’avait empêcher d’autres gens de prendre le vaccin et c’est là où la situation est devenue grave, peut-être les habitants auraient accepter cette maladie mais lorsque un dépité qui est très influent était parti déclarer que c’est une maladie fabriquée donc, il ne sait pas d’où la maladie est venue et cela à amener la confusion dans la population.

**P40: IA_04_BCZS_IS_01.docx - 40:10 [lorsque un dépité qui est très..] (24:24)	(Super)**

Codes:	[Considération_Ebola_population]

No memos

lorsque un dépité qui est très influent était parti déclarer que c’est une maladie fabriquée donc, il ne sait pas d’où la maladie est venue et cela à amener la confusion dans la population.

**P40: IA_04_BCZS_IS_01.docx - 40:11 [Répondant : Ils disaient que i..] (28:28)	(Super)**

Codes:	[Considération_Ebola_population]

No memos

**Répondant** : Ils disaient que il y a pas la maladie et que c’est les gens-là qui travaillent dans la riposte c’est sont eux qui veulent amené la maladie par ce que quand Ebola était venue les locaux ne faisaient pas parti des agents, ils étaient eux-mêmes qui sont venu et c’est le mêmes gens qui gèrent l’épidémie alors lorsque ils étaient entre de voir si il y a un cas suspect directement beaucoup de véhicule y arrive donc c’était vraiment un panique par ce que la population ne comprenait pas bien par ce que elle était entre de dire cette maladie quand même nous avons de médecin et c’est nos médecins qui aller nous dire que qu’il y a la maladie comment nos médecins ne sont pas au courant que ça soit la vaccination comment c’est pas nos médecins qui nous donnent le vaccin donc c’est une qui est comme la deuxième guerre par ce que on était dans le contexte de massacre de Beni et dans ce même contexte ils ont dit que donc c’est une autre forme de massacre.

**P40: IA_04_BCZS_IS_01.docx - 40:12 [Répondant : D’abord moi dans m..] (30:30)	(Super)**

Codes:	[Changements_depuis_Ebola]

No memos

**Répondant** : D’abord moi dans mon travail ça m’a fait souffrir par ce que j’avais de bonne couverture dans le PV de routine les maman ont fuient soit disant que lorsque ils vont amener leurs enfants à la vaccination on va le retenir pour l’injecter le vaccin d’Ebola pour donner à leurs enfants cette maladie donc, ça c’était un effet qui était néfaste dans mon travail par ce que j’étais entre d’entendre de bonnes couvertures donc quand j’étais en 95,99% mais le fait que Ebola est venu j’ai commencé à atteindre 30% et difficilement j’atteins 50% et par conséquent dans la période d’Ebola il y a eu aussi l’épidémie de rougeole car dernièrement j’étais entre de notifier beaucoup de cas de rougeole et c’était à cause de cela.

**P40: IA_04_BCZS_IS_01.docx - 40:13 [Répondant : Sur le plan social..] (32:32)	(Super)**

Codes:	[Changements_depuis_Ebola]

No memos

**Répondant** : Sur le plan social.

**P40: IA_04_BCZS_IS_01.docx - 40:14 [Répondant : Dans la communauté..] (34:34)	(Super)**

Codes:	[Changements_depuis_Ebola]

No memos

**Répondant** : Dans la communauté vous savez que les gens ne partaient pas au champ, et d’une part la population s’est appauvrie par ce que le gens ne partaient plus au champ et vous savez qu’il y avait deux évènement et nous avons de gens qui partent cultivés leur champ a Oicha mais à cause de l’insécurité ils ne partent plus et après la sécurité Ebola est venue donc c’était vraiment sur le plan social et les gens ne partaient plus au deuil hors ce genre des évènement c’est ce qui nous unissent quand on part compatir avec les autres qui sont endeuillé mais c’était à cause de l’Ebola quand on a interdire au gens ne pas participer au fine ralle.

**P40: IA_04_BCZS_IS_01.docx - 40:15 [Répondant : Dans le cadre de l..] (36:36)	(Super)**

Codes:	[Répondant_Responsabilité_pdt MVE]

No memos

**Répondant** : Dans le cadre de la riposte moi je fais la surveillance donc je m’occupe de supervise la gens qui font de recherche active dans la zone de santé et voir s’ils sont entrés de bien faire leur travail et s’ils ne sont pas entre passer inaperçue les cas qui répondent à la définition.

**P40: IA_04_BCZS_IS_01.docx - 40:16 [Répondant : A part ça ont cent..] (38:38)	(Super)**

Codes:	[Répondant_Responsabilité_pdt MVE]

No memos

**Répondant** : A part ça ont centralisé puis on fait de rapport

**P40: IA_04_BCZS_IS_01.docx - 40:17 [Répondant : Bon moi j’étais im..] (40:40)	(Super)**

Codes:	[Répondant_Responsabilité_pdt MVE]

No memos

**Répondant** : Bon moi j’étais impliqué dans la surveillance mais je n’étais pas entré directement, j’étais entré après dans le 1^er^ temps ils étaient venus pour exécutés tout le travail mais lorsque le travail est devenu difficile et il fallait qu’ils utilisent les locaux alors ils étaient obligés de prendre quelque tête pour les aidés.

**P40: IA_04_BCZS_IS_01.docx - 40:18 [Répondant : Moi je suis entré ..] (42:42)	(Super)**

Codes:	[Répondant_Responsabilité_pdt MVE_Depuis quand ?]

No memos

**Répondant** : Moi je suis entré un mois après voir même un mois et demi mais pour entrer dans un mois et demi je devrais entre au début mais il y avait de problèmes internés de notre maison

**P40: IA_04_BCZS_IS_01.docx - 40:19 [Répondant : Oui mais peut-être..] (44:44)	(Super)**

Codes:	[Répondant_Responsabilité_pdt MVE_Depuis quand ?]

No memos

**Répondant** : Oui mais peut-être je ne peux pas le dire

**P40: IA_04_BCZS_IS_01.docx - 40:20 [Répondant : Oui à moins que vo..] (46:46)	(Super)**

Codes:	[Répondant_Responsabilité_pdt MVE_Depuis quand ?]

No memos

**Répondant** : Oui à moins que vous le permettiez

**P40: IA_04_BCZS_IS_01.docx - 40:21 [Répondant : Donc c’était comme..] (48:48)	(Super)**

Codes:	[Répondant_Responsabilité_pdt MVE_Depuis quand ?]

No memos

**Répondant** : Donc c’était comme ça lorsque on a dit au chef de chercher les agents par ce que le médecin chef de zone était directement impliqué mais ce n’est ne pas celui que nous sommes avec lui, quand ils avaient vu la rémunération que va recevoir les agents ils avaient tendance d’écarter certains gens auxquels ils n’auront pas un intérêt et moi j’étais écarter parmi ce gens-là puisqu’il fallait qu’ils amènent leurs membres de famille et certains agents du bureau qu’ils vont conjuguer avec.

**P40: IA_04_BCZS_IS_01.docx - 40:22 [Répondant : Conjuguer, vous vo..] (50:50)	(Super)**

Codes:	[Répondant_Responsabilité_pdt MVE_Depuis quand ?]

No memos

**Répondant** : Conjuguer, vous voyez j’amène les gens qui sont à mon côté dans un domaine par exemple ou il faut un infirmier ou un psychologue mais ils amènent une catégorie de gens comme femmes libre par ce qu’ils trouvent que toi tu peux t’opposer à eux alors celui qui va sauvegarder leurs intérêts on part avec lui.

**P40: IA_04_BCZS_IS_01.docx - 40:23 [Répondant : Des intérêts je vi..] (52:52)	(Super)**

Codes:	[Répondant_Responsabilité_pdt MVE_Depuis quand ?]

No memos

**Répondant** : Des intérêts je viens de le dire, c’est pourquoi je vous ai demandez si je peux parler

**P40: IA_04_BCZS_IS_01.docx - 40:24 [Répondant : Bon par exemple on..] (54:54)	(Super)**

Codes:	[Répondant_Responsabilité_pdt MVE_Depuis quand ?]

No memos

**Répondant** : Bon par exemple on me dit de vérifier le registre voir de cas qui répondent et tu te dis comme ma sœur sera entre de trouver autant donc c’est elle que je veux mettre là ou bien une maitresse au détriment de celui qui peut bien faire c’est travail au lieu d’un médecin qui est à la structure mais tu préfères amener une personne que peut être vous allez chaque fois partager son salaire ou soit tu auras un % dans son salaire c’est dans ce sens.

**P40: IA_04_BCZS_IS_01.docx - 40:25 [Répondant : Peut-être ce que j..] (56:56)	(Super)**

Codes:	[Structure-Service_Fermeture_pdt MVE]

No memos

**Répondant** : Peut-être ce que j’étais entre de dire que beaucoup d’activités ont souffrir par ce que d’abord la population avait peur pour dire que si une personne part à l’hôpital on va alerter par exemple une femme enceinte peut être elle a de gravidique et si elle part à la CPN il y aura alerte directement elle reste à la maison.

**P40: IA_04_BCZS_IS_01.docx - 40:26 [Répondant : Alerter donc] (58:58)	(Super)**

Codes:	[Structure-Service_Fermeture_pdt MVE]

No memos

**Répondant** : Alerter donc

**P40: IA_04_BCZS_IS_01.docx - 40:27 [Répondant : Donc si une person..] (60:60)	(Super)**

Codes:	[Structure-Service_Fermeture_pdt MVE]

No memos

**Répondant** : Donc si une personne répond à une définition de cas, on appelle le service d’alerte pour qu’il part investiguer le cas et l’amener au CTE

**P40: IA_04_BCZS_IS_01.docx - 40:28 [Répondant : Oui] (62:62)	(Super)**

Codes:	[Structure-Service_Fermeture_pdt MVE]

No memos

**Répondant** : Oui

**P40: IA_04_BCZS_IS_01.docx - 40:29 [Répondant : Donc ils voient co..] (64:64)	(Super)**

Codes:	[Structure-Service_Fermeture_pdt MVE]

No memos

**Répondant** : Donc ils voient comme tout juste au début plusieurs gens ont trouvaient la mort au CTE par ce que les gens n’avaient pas encore maitrisé le traitement et c’était vraiment vers, les gens ont trouvaient CTE comme un mouroir et tu connais les gens qui étaient entre de partir au CTE après donc je voulais dire quoi ceux qui partaient avant d’être affaibli par la maladie ils étaient entre de guérir mais, si tu fais beaucoup de temps en étant malade et tu fais auto médication alors lorsque ont amenés et que tu es déjà affaibli c’était rare de quitter la bas alors juste au début beaucoup de gens ont perdus leur vie mais quand les gens ont compris que c’est une maladie et il faut partir au CTE c’est à ce moment-là que les gens étaient sauvé mais de le début là on a perdu beaucoup de gens.

**P40: IA_04_BCZS_IS_01.docx - 40:30 [Répondant : Dans des hôpitaux ..] (66:66)	(Super)**

Codes:	[Structure-Service_Fermeture_pdt MVE]

No memos

**Répondant** : Dans des hôpitaux il n’y a pas des services qui ont étaient fermés pendant l’Ebola sauf que c’est la population qui avait peur d’utilisé ces services.

**P40: IA_04_BCZS_IS_01.docx - 40:31 [Répondant : Au contraire ont v..] (68:68)	(Super)**

Codes:	[Structures-Services_fonctionnement]

No memos

**Répondant** : Au contraire ont veulent que les gens viennent pour que nous puissions élargir le cas répondent à la définition par ce que quand les gens viennent vous triez les cas suspects pour les amènes au CTE et ceux qui ne sont pas suspect on les envois à l’hôpital normal.

**P40: IA_04_BCZS_IS_01.docx - 40:32 [Répondant : Et ce pour quoi ce..] (70:70)	(Super)**

Codes:	[Service_chirurgie_fonctionnement]

No memos

**Répondant** : Et ce pour quoi ceux qui sont venus nous appuyé dans la riposte ils ont trouvés que il faut par ce que on avait pas la surveillance à base communautaire puis ils se sont dit que dans la surveillance qu’on ajoute à part recherche active que les relais communautaires aussi puisse nous aidé à chercher les gens dans la communauté et c’est ce qui nous a aidés de trouver celle-là qui resté dans la communauté les amenés aux soins mais ils n’ont pas réussi à amènes le femmes enceintes, ils n’ont pas réussi à amènes ceux qui ne suivent pas normalement le calendrier vaccinal par ce que c’était seulement pour les malades .

**P40: IA_04_BCZS_IS_01.docx - 40:33 [Répondant : Il y a certaines s..] (72:72)	(Super)**

Codes:	[Structures-Services_fonctionnement]

No memos

**Répondant** : Il y a certaines structures privées qui sont fermées par ce que dans plusieurs structures les malades commençaient à avoir peur comme la structures qui est bien en norme comme des hôpitaux et structures publique les malades partaient se cacher dans des structures privées de tradi praticien et cela que il y avait beaucoup de cas positif de MVE alors lorsque ils ont remarqué que les infirmiers structures commence à mourir par ce que ils disaient que cette maladie n’existe pas et les malades aller se cacher la bas tant on ils hospitaliser le malades le soir et puis le matin lorsque ils voient les agents de la riposte prêtent à venir ils disent au malades de rentrer à la maison et revenir après le départ de ces agents c’est comme ça qu’il y avait beaucoup de contamination au niveau de ces structures ou l’on cacher les gens mais je ne dis pas que au niveau des hôpitaux il y avait pas de cas d’Ebola par ce que il peut arriver qu’un malade vienne à l’hôpital mais il cache les signes pour que il puisse au triade et une fois à l’hôpital on va remarquer qu’il répond à la définition alors que il est déjà passer à l’hôpital mais beaucoup de cas étaient au privées c’est pour quoi hier lorsque vous avez demandé si dans ces structures il avait des agents de santé qui sont mort mais nous on les avaient pas trouvé par ce que il se cacher dans ces structures et c’est pourquoi on n’était pas au courant si les agents étaient mort.

**P40: IA_04_BCZS_IS_01.docx - 40:34 [Répondant : Les structures nor..] (74:74)	(Super)**

Codes:	[Structures-Services_fonctionnement]

No memos

**Répondant** : Les structures normées je voulais les structures au quelles leurs données on les a codés dans le SNIS par ce que notre zone de santé à des particularités donc tout le monde se réveille chez lui il dit je veux ouvrir le centre il ouvre mais il a des appuis que nous on ne peut pas contrôler au niveau du BCZS.

**P40: IA_04_BCZS_IS_01.docx - 40:35 [Répondant : Bon toutes les act..] (76:76)	(Super)**

Codes:	[Service_vaccination_Fonctionnement]

No memos

**Répondant** : Bon toutes les activités préventives ont baissé, CPS, vaccination, CPN

**P40: IA_04_BCZS_IS_01.docx - 40:36 [Répondant : Même les accouchem..] (78:78)	(Super)**

Codes:	[Service_maternité_fonctionnement]

No memos

**Répondant** : Même les accouchements aussi ont chuté à cause de ça

**P40: IA_04_BCZS_IS_01.docx - 40:37 [Répondant : Les gens étaient l..] (80:80)	(Super)**

Codes:	[Service_maternité_fonctionnement]

No memos

**Répondant** : Les gens étaient là mais ils avaient peur par ce que ces gens disaient si elle arrive au centre par exemple celui qui peut saigner hors saigner aussi c’est parmi la définition de cas et elle devient suspect et directement on l’oriente à la CTE ou soit on fait un prélèvement local e c’est à cause de ça que les femmes avaient peur et plusieurs se rendaient dans des structures privées de tradi-praticien pour accouché là-bas ou bien soit elle accouche a son domicile.

**P40: IA_04_BCZS_IS_01.docx - 40:38 [002746 R : c’est ce qui a étai..] (82:82)	(Super)**

Codes:	[Structure-Service_Utilisation_population]

No memos

002746 R : c’est ce qui a était observé et pour palier à ça ils ont instauré la gratuité de soins et quand ils ont dit au gens que le médicament aussi sera gratuit, l’accouchement devient subventionné dans le PDCC, c’est ça qui nous a aidé à amener les malades aux soins.

**P40: IA_04_BCZS_IS_01.docx - 40:39 [Répondant : Au début c’était p..] (84:84)	(Super)**

Codes:	[Gratuité_Structures-Services_concernés]

No memos

**Répondant** : Au début c’était pour tout le monde et ce qui était pour tout le monde le constant était que tout le monde est malade par ce que il y avait engouement aux hôpitaux et les médecins étaient submergé et ils quittaient aux hôpitaux la nuit entre de soigné alors quand ils avaient essayé le gratuité pour tout le monde pendant un mois et demi ils ont stoppé et cible les maladies qui seront subventionnée mais la première gratuité qui avait fait un mois et demi il parait que ce qui avait subventionné cette gratuité ils avaient trouvé qu’il ne seront pas à mesure par ce que ils avaient pensé que ça sera fois deux mais elle était même fois dix pour les malades et puis le partenaire avait trouvé ça difficile.

**P40: IA_04_BCZS_IS_01.docx - 40:40 [Répondant : Et il y avait de c..] (86:86)	(Super)**

Codes:	[Gratuité_Information_population]

No memos

**Répondant** : Et il y avait de communique à la radio et les médecins disaient aux malades que la gratuité commence et les agents de la base communautaire passaient dans de quartier porte par porte pour informer les malades s’il trouve un malade il le dit de partir à l’hôpital et le malade lui dit qu’il n’a pas l’argent puis on l’informe que le médicament est gratuit directement les malades viennent à l’hôpital.

**P40: IA_04_BCZS_IS_01.docx - 40:41 [Répondant : Si le gens avait p..] (88:88)	(Super)**

Codes:	[Gratuité_Rumeurs]

No memos

**Répondant** : Si le gens avait peur de la gratuité ils n’allaient pas venir nombreux, mais les malades sont venus et malheureusement on était distrait pour prendre de photo donc ça pouvait même être un risque pour la contamination les malades étaient nombreux il y avait même pas la mesure de distançassions sociale comme à Matanda, j’arrivais à Matanda il y avait un grand engouement et ici à l’hôpital.

**P40: IA_04_BCZS_IS_01.docx - 40:42 [Répondant : Non il n’y avait p..] (90:90)	(Super)**

Codes:	[Gratuité_Rumeurs]

No memos

**Répondant** : Non il n’y avait pas de rumeurs sauf pour la nouvelle gratuité qui est d’aujourd’hui

**P40: IA_04_BCZS_IS_01.docx - 40:43 [Répondant : la gratuité d’aujo..] (92:92)	(Super)**

Codes:	[Gratuité_Evolution]

No memos

**Répondant** : la gratuité d’aujourd’hui ils prennent la carte d’identité ou une identité pour vérifier si la personne est malade et avec ça on peut chercher la personne et on la trouve facilement alors les gens commence à dire ces photos qu’ils sont entre de prendre ou des photocopies de nos carte d’électeur puis les gens commencent à dire que ça c’est un autre aspect que ces gens risquent de vendre leurs parcelles.

**P40: IA_04_BCZS_IS_01.docx - 40:44 [Répondant : C’est pourquoi les..] (94:94)	(Super)**

Codes:	[Gratuité_Rumeurs]

No memos

**Répondant** : C’est pourquoi les gens ne sont pas entre de venir aux soins en disant que ça c’est une autre façon et que ça peut être rejoindre la balkanisation et que ces gens viendront prendre leur maison pour les données aux autres sans le savoir

**P40: IA_04_BCZS_IS_01.docx - 40:45 [Répondant : Ce qui est gratuit..] (96:96)	(Super)**

Codes:	[Gratuité_Structures-Services_concernés]

No memos

**Répondant** : Ce qui est gratuit au niveau des hôpitaux c’est la médecine interne mais autres choses non

**P40: IA_04_BCZS_IS_01.docx - 40:46 [Répondant : Le fait de beaucou..] (98:98)	(Super)**

Codes:	[Gratuité_Conséquences]

No memos

**Répondant** : Le fait de beaucoup travailler et la qualité de travail aussi à baisser par ce que tu vas consulter plus de 100 personnes mais tu peux normalement comme il le faut

**P40: IA_04_BCZS_IS_01.docx - 40:47 [Répondant : Si tu travailles e..] (100:100)	(Super)**

Codes:	[Gratuité_Conséquences]

No memos

**Répondant** : Si tu travailles en étant fatiguer tu ne feras pas un bon travail.

**P40: IA_04_BCZS_IS_01.docx - 40:48 [Répondant : La grève a fait pl..] (102:102)	(Super)**

Codes:	[Ressources_humaines_situation_grève]

No memos

**Répondant** : La grève a fait plus de six mois

**P40: IA_04_BCZS_IS_01.docx - 40:49 [Répondant : Sans rapport il n’..] (104:104)	(Super)**

Codes:	[Ressources_humaines_situation_grève]

No memos

**Répondant** : Sans rapport il n’y a pas de travail et sans rapport ils ne sauront pas que vous êtes entre de travailler et toute hiérarchie était bloqué il y a pas analyse et l’épidémie aussi est venue sans que personne ne soit au courant

**P40: IA_04_BCZS_IS_01.docx - 40:50 [Répondant : On était entre de ..] (106:106)	(Super)**

Codes:	[SNIS_fonctionnement_Decrire_pdt MVE]

No memos

**Répondant** : On était entre de dire aux gens de faire le rapport et le garder comme ça quand on va lever la grève directement ils amènent le rapport.

**P40: IA_04_BCZS_IS_01.docx - 40:51 [Répondant : C’est ça après la ..] (108:108)	(Super)**

Codes:	[SNIS_fonctionnement_Decrire_pdt MVE]

No memos

**Répondant** : C’est ça après la grève on avait dit d’amener les rapports certaines personnes ne faisaient pas le rapport et à cette période ils étaient entre de récolter hors lorsque on récolte beaucoup de donnée à la fois il doit y avoir de perte et puis ils font de modification par ce que les autres ne sont plus.

**P40: IA_04_BCZS_IS_01.docx - 40:52 [Répondant : Je dirais oui.] (110:110)	(Super)**

Codes:	[SNIS_fonctionnement_Decrire_pdt MVE]

No memos

**Répondant** : Je dirais oui.

**P40: IA_04_BCZS_IS_01.docx - 40:53 [Répondant : Je dirais oui et n..] (112:112)	(Super)**

Codes:	[SNIS_fonctionnement_Decrire_pdt MVE]

No memos

**Répondant** : Je dirais oui et non par ce que il y a d’autres structures ou les infirmiers étaient attaqué dans des structures que je vous ai parlé hier, à côté de wayene les infirmiers se cacher par ce que la population disait qu’elle va le tué par ce que ils avaient accepté qui y a la maladie alors que il y a pas la maladie la population disait que ces infirmiers ce sont alliés avec ce qui sont venu et que ils ont réussi quelque chose pour faire entre la maladie alors les familles de victime il y a des endroits où ils ont brulé le matériels comme le sable.

**P40: IA_04_BCZS_IS_01.docx - 40:54 [Répondant : A part ça le rappo..] (114:114)	(Super)**

Codes:	[SNIS_fonctionnement_Decrire_pdt MVE]

No memos

**Répondant** : A part ça le rapportage continuer normalement.

**P40: IA_04_BCZS_IS_01.docx - 40:55 [Répondant : Ça poser un problè..] (116:116)	(Super)**

Codes:	[Ressources_humaines_Disponibilité et perturbations]

No memos

**Répondant** : Ça poser un problème dans le sens ou toutes les ressources dont on avait besoins pendant la période de l’épidémie n’était pas disponible par exemple au niveau des infirmiers de fois on cherchait les infirmiers au CTE et les mêmes infirmiers travaillent dans des hôpitaux alors le fait de travailler à deux endroits ça devient difficile alors les gens choisissaient là où ils vont bien se sentir alaise ou ils seront bien rémunérés.

**P40: IA_04_BCZS_IS_01.docx - 40:56 [Répondant : Même moi aussi je ..] (118:118)	(Super)**

Codes:	[Ressources_humaines_Disponibilité et perturbations]

No memos

**Répondant** : Même moi aussi je suis entre de travailler ici mais depuis le mois d’Aout on ne touche rien ici même un 10 franc mais à l’Ebola on nous donne quelque chose c’est pour quoi vous aller remarquer que on passe peu de temps ici qu’a la riposte par ce que là-bas tu vas gagner quelque chose pour nourrir les enfants mais ici tu vas trouver que c’est que tu fais c’est une sacrifice mais on le fait toujours pour protéger le système mais travailler à deux endroits il y arrive qu’un endroit soit laisser que l’autre.

**P40: IA_04_BCZS_IS_01.docx - 40:57 [Répondant : Les conséquences é..] (120:120)	(Super)**

Codes:	[SNIS_fonctionnement_Decrire_pdt MVE]

No memos

**Répondant** : Les conséquences étaient seulement à la promptitude mais ça arrive est-il est déjà à retard.

**P40: IA_04_BCZS_IS_01.docx - 40:58 [Répondant : Je pense pour gens..] (122:122)	(Super)**

Codes:	[Ressources_humaines_Disponibilité et perturbations]

No memos

**Répondant** : Je pense pour gens de CPN ils n’étaient pas détacher surtout ici au niveau de l’hôpital tous sont là.

**P40: IA_04_BCZS_IS_01.docx - 40:59 [épondant : Bon ils s’arrangeai..] (124:124)	(Super)**

Codes:	[Ressources_humaines_Disponibilité et perturbations]

No memos

**épondant** : Bon ils s’arrangeaient dans le service par ce qu’ils sont par exemple à 4 ils s’arrangent que les autres partent au CTE et d’autres restent entre de travaillé donc il n’y avait pas de répond c’est pourquoi je vous ai dit que ça beaucoup joué à la qualité du travail.

**P40: IA_04_BCZS_IS_01.docx - 40:60 [Répondant : Dans la gratuité d..] (126:126)	(Super)**

Codes:	[Médicaments_disponibilité_fosa]

No memos

**Répondant** : Dans la gratuité de soins on avait le médicament et il y a toujours le médicament sauf qu’il y avait un seul problème de nous amener le médicament soit qu’on n’a pas besoin et qui est déjà surfacture c’est ce que nous avons eu comme problème ?

**P40: IA_04_BCZS_IS_01.docx - 40:61 [Répondant : Non c’est pourquoi..] (128:128)	(Super)**

Codes:	[Médicaments_disponibilité_fosa]

No memos

**Répondant** : Non c’est pourquoi je vous ai dit qu’on avait de médicament.

**P40: IA_04_BCZS_IS_01.docx - 40:62 [Répondant : Oui c’est ici chez..] (130:130)	(Super)**

Codes:	[Médicaments_disponibilité_fosa]

No memos

**Répondant** : Oui c’est ici chez nous ou le médicament arrive puis on distribuer ça à de structures.

**P40: IA_04_BCZS_IS_01.docx - 40:63 [Répondant : Au début nous avon..] (132:132)	(Super)**

Codes:	[Réunion_pdt-épidémie-Ebola]

No memos

**Répondant** : Au début nous avons échoué à faire de réunions de l’équipe cadre, de revue mensuelle même de COGE nous avons échoué mais ce n’est qu’après trois mois quand on a commencé l’approche zonale puis on se un peut récessif et voir qu’on doit faire encore de réunion

**P40: IA_04_BCZS_IS_01.docx - 40:64 [Répondant : Vous savez c’est u..] (134:134)	(Super)**

Codes:	[Réunion_pdt-épidémie-Ebola]

No memos

**Répondant** : Vous savez c’est une chose nouvelle et jamais vu une chose pareille donc tout le temps on était plongé dans l’épidémie, en tout cas on avait pas même un petit temps pour arriver ici au bureau par ce que s’il y avait une personne qui est reste ici au bureau dans les trois premiers mois c’était l’AGIS, le secrétaire et le huissier qui étaient ici et le comptable si je peux le dire, ces gens-là ne peuvent pas organiser une réunion cadre ou COGE aucune réunion. Mais ce ne que après trois mois qu’on se dit que on ne peut pas se pencher a une seule chose par ce que on n’avait trouver que l’épidémie va prendre encore beaucoup de temps avant de finir par ce que on avait pensé que peut être l’épidémie n’aller pas dépasser deux à trois mois avant qu’elle ne soit maitriser ou finir mais c’est ne te pas le cas et lorsque ils avaient vu que dans toutes les zones nous tous nous partons à l’auberge le matin à 7h 30 et après avoir finir la réunion on part au terrain et après le terrain on revient au terrain, donc on se réveiller le matin on part la bas jusque le soir alors lorsque c’était une approche zonale donc à Goma on a trouvé que au niveau des centres il y a pas de formation ils se sont dit que ils doivent au moins sacrifier un temps en prenant un temps pour BCZS et un autre à la riposte puis on a commencé à organiser de réunions.

**P40: IA_04_BCZS_IS_01.docx - 40:65 [Répondant : Bon nous nous somm..] (136:136)	(Super)**

Codes:	[Réunion_pdt-épidémie-Ebola]

No memos

**Répondant** : Bon nous nous sommes sacrifié par ce qu’on a trouvé qu’on est entre de faire tomber le système.

**P40: IA_04_BCZS_IS_01.docx - 40:66 [Répondant : Nous étions tous f..] (138:138)	(Super)**

Codes:	[Gouvernance_soins santé]

No memos

**Répondant** : Nous étions tous focalisé dans et nous étions entre de dépendre par ce qu’entre nous il n’y avait personne qui avait la capacité, c’était quelque chose étrange dans le contré et on devrait se mettre ensemble pour s’avoir ce qu’on peut faire.

**P40: IA_04_BCZS_IS_01.docx - 40:67 [Répondant : En tout cas pour l..] (140:140)	(Super)**

Codes:	[Implication_Communauté]

No memos

**Répondant** : En tout cas pour l’implication de la communauté, les agents de la communication ils nous a beaucoup aidé par ce que si ils avaient pas bien faire comprendre la communauté que la maladie existe pour que les gens comprennent que s’il ne s’implique pas cette maladie va nous exterminer et on devrait rester toujours à la première phase qui est le début de l’épidémie, par ce que on n’enterré les gens même à deux heures du matin , si une personne meurt on l’enterre sans souabe pour éviter et avoir peur que le agents de Ebola ne trouve rien sur place.

**P40: IA_04_BCZS_IS_01.docx - 40:68 [Répondant : Alors pour le fair..] (142:142)	(Super)**

Codes:	[Implication_Communauté]

No memos

**Répondant** : Alors pour le faire comprendre que la maladie existe c’est pourquoi nous arrivons à ce niveau.

**P40: IA_04_BCZS_IS_01.docx - 40:69 [Répondant : De ma part le chan..] (144:144)	(Super)**

Codes:	[Souhait_Changement_gestion_futures_épidémies]

No memos

**Répondant** : De ma part le changement que je vu dans cette épidémie, je vu que sans l’implication de la communauté locale rien ne peut marcher. Donc si je parts à un autre endroit je dois savoir que cette communauté de cet endroit doit se mettre ensemble pour chercher de piste de solution à la maladie, mais pas à dire que toi tu vas venir avec ton expertise, que tu sois expert tu ne peux pas réussir si tu ne travailles pas avec la communauté locale et surtout si la communauté ne pas d’accord avec toi, ça j’avais trouvé que c’est très important dans la gestion de l’épidémie.

**P40: IA_04_BCZS_IS_01.docx - 40:70 [Répondant : De mon côté, dans ..] (146:146)	(Super)**

Codes:	[Souhait_Changement_gestion_futures_épidémies]

No memos

**Répondant** : De mon côté, dans chaque service moi je vus dans cette épidémie ce qui m’a donné une leçon, il fallait comme je viens de le dire que si un expert arrive il doit travailler avec nous, prendre un accord ensemble pour savoir comment nous allons travailler pour éviter qui est de mésentente. On doit planifier le travail ensemble pour ne pas privilégier ce qui viennent par ce que si une partie est privilégier l’autre va souffrir ou ça créer un autre problème comme je le bien dit lorsqu’il y avait vaccination les couvertures étaient faible si on avait une autre épidémie il est vrai que on avait le médicament pour la prise en charge suffit de maitriser l’épidémie de la rougeole

**P40: IA_04_BCZS_IS_01.docx - 40:71 [Répondant : Il n’y a pas] (148:148)	(Super)**

Codes:	[Autres_Problèmes observés]

No memos

**Répondant** : Il n’y a pas

**P40: IA_04_BCZS_IS_01.docx - 40:72 [Répondant : Ma conclusion, moi..] (150:150)	(Super)**

Codes:	[Commentaire-Recommandation_Répondant]

No memos

**Répondant** : Ma conclusion, moi d’abord je dis merci aux gens qui sont venus nous appuyé par ce que nous avons ajouté un plus par rapport à ce qu’on faisait avant, nous avons acquérir de nouvelles choses et si une autre épidémie arrive je pense bien que nous avons peur de corona mais nous avons quand même quelque chose d’avance et nous savons que au niveau des structures le gens doivent bien se protéger par ce que avant on faisait des injection prélèvement de sang sans porter le gant on se faisait confiance.

**P40: IA_04_BCZS_IS_01.docx - 40:73 [Répondant : Je pense c’est déj..] (152:152)	(Super)**

Codes:	[PCI_Comment_maintenir]

No memos

**Répondant** : Je pense c’est déjà parce que ni se lavé les mains on n’avait pas cette culture qui tu touches quelque chose après tu te laves les mains je pense si on continue comme ça par ce que chaque chose a ses leçons même si nous avons perdus beaucoup de gens mais cette maladie nous a ouvert les yeux.

**P41: IA_04_BCZS_IS_02.docx.rtf - 41:1 [Répondant : Pour le moment je ..] (8:8)	(Super)**

Codes:	[Répondant_Responsabilité_habituelles]

No memos

**Répondant** : Pour le moment je suis charge du sinise, du VIH.

**P41: IA_04_BCZS_IS_02.docx.rtf - 41:2 [Répondant : Dans les activités..] (10:10)	(Super)**

Codes:	[Répondant_Responsabilité_habituelles]

No memos

**Répondant** : Dans les activités de la zone, quand on est superviseur, on devient polyvalent, on doit faire presque le maximum de ce qu’on a à faire, même si on est chargé du sinise on peut faire autre chose, s’il y’a une activité dans la vaccination ou ailleurs, pour que l’activité ne souffre, si le premier répondant n’est pas là, on est alors sensé réaliser cet activité-là. Dans le cadre du sinise, nous recevons les canevas, le rapport provenance des structures, et nous analysons de la réception, nous saisissons et après nous analysons ça dans le DHSD, et après cette analyse, nous organisons la revue en collaboration avec les autres membres de l’équipe cadre. Par rapport au VIH, nous recevons aussi leurs rapports, nous faisons, nous participons à l’élaboration de la commande pour leur centrale, les ARV, et les autres médicaments. Nous participons de fois à des réunions des personnes vivant avec les VIH et a des consolidations de se donner avec les structures et avec là sous coordination. D’une manière globale, c’est ce que nous faisons, y compris la supervision que nous réalisons dans les structures, dès que l’on analyse un rapport, il y’a des problèmes qui se font voir et à partir de ces problèmes identifies nous planifions des supervisions avec les amis de supervision conjointe. A partir de cette analyse, ça nous ouvre la figure d’aller sur terrain, pour essayer de résoudre ces problèmes constate lors de la saisi de ce rapport

**P41: IA_04_BCZS_IS_02.docx.rtf - 41:3 [Répondant : Par rapport à cett..] (12:12)	(Super)**

Codes:	[Début-Progression_Epidémie MVE]

No memos

**Répondant** : Par rapport à cette épidémie de maladie a virus d’Ebola, il y’avait grève des personnes, on surveillait les maladies et pendant la grève qu’on a informé qu’il y a des cas qu’on vient d’enregistrer dans la zone de sante de MABALAKO, précisément dans l’air de sante de MANGINA. Comme on suivait ça de loin, et après un moment, quelques deux ou trois semaines après, qu’il y’a eu le premier cas qu’on a pu dépister a BUTEMBO. Un cas qui venait de MANGINA et qui s’est diriger vers BUTEMBO, là où il y’avait sa famille, c’est après une semaine qu’on a eu un cas à BUTEMBO, un monsieur qui venait dans une des aires de sante de KATWE qui est rentre et qu’on a appris comme quoi il a présente certains signes et il a été confirmé positif. Par après nous avons eu des cas, qui sont autochtones a BUTEMBO, a KATWE et c’est ainsi qu’on est entre dans le rythme, à partir de ces cas originaires de la zone de sante de KATWE.

**P41: IA_04_BCZS_IS_02.docx.rtf - 41:4 [Répondant : Le cas dont j’ai f..] (14:14)	(Super)**

Codes:	[Début-Progression_Epidémie MVE]

No memos

**Répondant** : Le cas dont j’ai fait allusion c’était le cas de l’aire de sante de MASIKI, et les autres cas qu’on a eu de KATWE c’était des cas de l’aire de santé de MACHANGA.

**P41: IA_04_BCZS_IS_02.docx.rtf - 41:5 [Répondant : d’emblée comme c’é..] (16:16)	(Super)**

Codes:	[Considération_Ebola_population]

No memos

**Répondant** : d’emblée comme c’était une nouvelle épidémie, et dans les habitudes de la zone, on n’a jamais eu des épidémies, personnellement depuis que je suis-là, on n’avait jamais géré une épidémie dans la zone. Quand la population a appris qu’il y’a une épidémie dans la zone, et dans le contexte ou l’épidémie est apparue pendant la période électorale, la population a semblé lier l’épidémie à cette fièvre de la situation de pré-électorale, ils ont lie ça a la politique en disant que, est-ce que les politiciens n’ont pas imposé cette épidémie, n’ont-ils pas amené cette épidémie pour chercher à les étouffer, et c’était ça la rumeur qui circuler en disant que c’était lie à la politique

**P41: IA_04_BCZS_IS_02.docx.rtf - 41:6 [Répondant : Vu que cette popul..] (18:18)	(Super)**

Codes:	[Changements_depuis_Ebola]

No memos

**Répondant** : Vu que cette population n’a pas accepter facilement l’épidémie, cela a été à la base de beaucoup de réticences de la part de la population et cette réticence a été à la base de multiplication des contacts et cela a été à la cause de beaucoup des cas positifs. La population n’a pas permis d’accéder aux familles, aux personnes atteintes de la maladie pour que l’on puisse voir ce qu’on peut faire, même les actions de sante publique autour du cas pour minimiser la contamination des autres, cela a laissé que l’épidémie s’étende sur les autres au moment où c’était peut-être possible à ce qu’on puisse l’arrêter quand il n’avait pas encore assez des cas qui sont atteint.

**P41: IA_04_BCZS_IS_02.docx.rtf - 41:7 [Répondant : sur le plan profes..] (20:20)	(Super)**

Codes:	[Changements_depuis_Ebola]

No memos

**Répondant** : sur le plan professionnel d’abord les gens les précepteurs comme les autres gens, avaient peur d’aller travailler, de participer aux activités de la riposte d’abord suite à la réticence de la population, aux menaces de la population, certaines personnes avaient peur de s’impliquer ouvertement dans les activités de la riposte et cela aussi a été un frein pour qu’on puisse mener correctement les activités. Sur le plan social, il y’a même des familles qui se sont disloqué, il y’a eu des querelles dans des ménages parce qu’il y’avait certaines personnes quand suggérer à ce que même un membre de la famille soit traité ou amené pour le prendre en charge correctement, la population comme elle n’avait pas accepter ça, supposait cette personne doit avoir reçu quelque chose qui aurait permis à ce qu’il puisse autoriser à ce que son frère ou son ami soit pris en charge. Comme au début la, le taux de mortalité était élevé, et quand quelqu’un mourrait et que c’est un membre de la famille ou de la communauté qui aurait annoncé comme quoi il y’a un cas suspect au village ou dans la cellule, cela créer des problèmes dans le village ou dans la société.

**P41: IA_04_BCZS_IS_02.docx.rtf - 41:8 [Répondant : la tâche n’était p..] (22:22)	(Super)**

Codes:	[Répondant_Responsabilité_pdt MVE]

No memos

**Répondant** : la tâche n’était pas si facile comme je l’ai dit au début, la population a été trop réticente mais on n’a pas baissé les bras, nous avons augmenté les contacts, les sensibilisations, jusqu’à ce que la population a compris et surtout quand il y’a eu suffisamment des cas de décès, ils ont compris que c’est une réalité et qu’il faut trouver une solution et ils se sont impliqué d’une manière ou d’une autre. Mais il y’a ces aires de sante qui ont persisté dans cette réticence, et ce sont là les aires qui ont eu beaucoup des cas des décès.

**P41: IA_04_BCZS_IS_02.docx.rtf - 41:9 [Répondant : nous avons intensi..] (24:24)	(Super)**

Codes:	[Répondant_Responsabilité_pdt MVE]

No memos

**Répondant** : nous avons intensifié la sensibilisation dans la communauté, malgré les menaces de ce dernier, et quand nous avons intensifié la sensibilisation, on a intéressé même les autorités politico-administratifs, de fois on les appelait ici et ailleurs et parfois dans leurs milieux. Avec nous, les autorités politiques, religieuses, on est parvenu à faire comprendre à la population que malgré leur soupesons de lier ça à la politique, la maladie est là et il faut qu’on s’implique pour voir l’éradiquer.

**P41: IA_04_BCZS_IS_02.docx.rtf - 41:10 [Répondant : Le changement a ét..] (26:26)	(Super)**

Codes:	[Structures-Services_fonctionnement]

No memos

**Répondant** : Le changement a été là, tout au début quand on a constante que la population a fui comme c’était une réticence, les responsables qui sont impliqué dans la riposte et le gouvernement, ils ont pris l’astuce de prendre en charge les soins, l’épidémie a commencé au mois d’aout mais entre aout et octobre il y’a eu une diminution sensible des cas surtout sur tous le plan des CPN, CPS, consultation et quand on a constaté cette diminution au moment où l’épidémie reprenait une allure un peu galopante, le gouvernement a jugé mieux de prendre en charge tout : consultation, accouchement… et avec cette stratégie et vu le niveau de vulnérabilité de la population, on a senti un engouement des malades dans les structures et cette engouement, les gens s’amenaient, parce qu’on avait appris qu’il a des malades dans la société qui ne se font pas soigner parce que peut-être les conditions socio-économiques n’étaient pas réuni, à partir du mois de décembre ou les soins ont été supporté par le gouvernement, dans tous les services il y’a eu augmentation des cas. Et cela pendant trois mois, et pendant le mois de janvier 2019, on a interrompu avec ce projet, il y’a eu baisse sensible encore de la fréquentation dans les structures. Pour la consultation, nous étions déjà au-delà de cent pour cent on est entré dans le 130%, 140%, mais quand on a interrompu les soins pendant un mois en attendant à ce qu’on signe un nouveau protocole, on est descendu autour de cinquante pour cent, le taux d’utilisation du curatif, et quand on a repris au mois de février, mars on a repris ces soins gratuits mais ce n’était plus global, il y’a eu encore augmentation entre 80 et 90% des nouveaux cas pendant cette prise en charge partielle des soins par le gouvernement. En gros, quand il y’a eu soins gratuits, il y’a eu augmentation, quand il y’a diminution, il y’a eu diminution.

**P41: IA_04_BCZS_IS_02.docx.rtf - 41:11 [Répondant : La baisse sensible..] (28:28)	(Super)**

Codes:	[Structure-Service_Utilisation_population]

No memos

**Répondant** : La baisse sensible était liée à l’épidémie elle-même, la population pense qu’une fois on fréquente la structure, on risque d’être ciblé et être pris comme cas suspect, d’être accompagné au CTE et d’aller y mourir, ce qui était à la base de cette diminution et comme c’était encore au début de l’épidémie.

**P41: IA_04_BCZS_IS_02.docx.rtf - 41:12 [Répondant : C’était ça la caus..] (30:30)	(Super)**

Codes:	[Structure-Service_Utilisation_population]

No memos

**Répondant** : C’était ça la cause majeure.

**P41: IA_04_BCZS_IS_02.docx.rtf - 41:13 [Répondant : C’est au niveau hi..] (32:32)	(Super)**

Codes:	[Gratuité_Interruption]

No memos

**Répondant** : C’est au niveau hiérarchique, au mois de décembre c’était la fin du premier projet ou l’on avait signé le projet pour trois mois, à la fin en attendant qu’on signe le second projet, il y’a eu une période d’un mois et pendant ce mois et avec l’épidémie, cette peur qu’on avait annoncée au début, le niveau socio-économique qui était faible, cela a causé une diminution rapide de 100% à 50%

**P41: IA_04_BCZS_IS_02.docx.rtf - 41:14 [Répondant : Avant l’épidémie, ..] (34:34)	(Super)**

Codes:	[Structure-Service_Fermeture_pdt MVE]

No memos

**Répondant** : Avant l’épidémie, nous avions plus de 300 structures dans la zones, privée, étatique… parmi ces 300 nous avons 39 qui sont intégrées, et ces derniers n’ont pas fermé, elles ont été opérationnelles même pendant l’épidémie, mais les privées, il y’a eu une grande partie n’a pas fonctionné, il y’a un temps où on a fonctionné avec 216, les autres se sont fermées d’elles même, et les autres que la zone de sante avait fermé officiellement, vu qu’il avait augmentation des infections des infirmiers dans ces structures liées à la maladie a virus d’Ebola.

**P41: IA_04_BCZS_IS_02.docx.rtf - 41:15 [Répondant : La raison était qu..] (36:36)	(Super)**

Codes:	[Structure-Service_Fermeture_pdt MVE]

No memos

**Répondant** : La raison était que, une fois ou deux fois on enregistrer des cas positifs, la population s’attaquait à ces structures, et vu que la majorité était des structures privées, ils avaient jugé mieux de fermer, d’autres étaient des locateurs, et ils se voyaient qu’une fois on détruit la maison du bailleur ou du propriétaire, ils risquent d’avoir des problèmes plus tard. Alors, au lieu qu’on brule ou qu’on détruise la structure, ils ont jugé de fermer.

**P41: IA_04_BCZS_IS_02.docx.rtf - 41:16 [Répondant : Parmi les 39, comm..] (38:38)	(Super)**

Codes:	[Structures-Services_fonctionnement]

No memos

**Répondant** : Parmi les 39, comme il y’avait les soins gratuits, on avait pris 30 pour les soins gratuits et les 9 autrement. Parmi les 39, il y’a des structures qui ont été détruites aussi suite à la réticence de la population, c’est comme l’aire de sante de MUCANGA, vu les menaces et destructions de certains matériels ou comme à MUCANGA ils ont brulé tous ce qui est en rapport avec la vaccination, l’aire de santé n’était plus opérationnel, il ont fermé momentanément toutes les activités, il y’a eu même le syndicat, les infirmier qui avaient jugé a un moment donné de suspendre toutes les activités pour chercher à trouver solution de cette résistance de la population et avec les dégâts qu’ils ont commis par rapport à ça.

**P41: IA_04_BCZS_IS_02.docx.rtf - 41:17 [Répondant : C’est lié à l’épid..] (40:40)	(Super)**

Codes:	[Agression-Menaces_Structure-Prestataire_raisons]

No memos

**Répondant** : C’est lié à l’épidémie elle-même car la population pensait qu’on risque de vacciner les enfants le vaccin contre Ebola, elle n’amenait plus les enfants, et ils voyaient le va-et-vient des équipes de la riposte au niveau de cette aire de sante, ils ont vu que les membres de la riposte collaboraient fortement avec l’équipe de riposte, et cela a été la cause de la destruction de cette structure, c’est pourquoi c’était vraiment liée à Ebola.

**P41: IA_04_BCZS_IS_02.docx.rtf - 41:18 [Répondant : Avec la réticence ..] (42:42)	(Super)**

Codes:	[Service_vaccination_Fonctionnement]

No memos

**Répondant** : Avec la réticence les gens pensaient que comme la vaccination était là, on pensait que comme les gens refusaient la vaccination, on pourrait profiter en amenant les enfants on risque de les vacciner, ils avaient la crainte, on disait que si un enfant malade on les pèse avec les matériels, et que les parents s’asseyent cote a cote, tout ça, c’était parmi les facteurs qui laissent à ce qu’il a réticence dans certains aires de sante, et d’autres refusaient d’utilisaient les biens de la structure, tous cela contre la contamination.

**P41: IA_04_BCZS_IS_02.docx.rtf - 41:19 [Répondant : Dans le cas de VIH..] (44:44)	(Super)**

Codes:	[Service_prise-en-charge_fonctionnement]

No memos

**Répondant** : Dans le cas de VIH, il y’a eu diminution des cas dépistés, la moyenne était de 10 à 15 cas positifs lors de la DCIP, mais on a eu une diminution par rapport à ça, les gens avaient peur de venir aux soins, et même les volontaires qui venaient au dépistage ne venaient plus.

**P41: IA_04_BCZS_IS_02.docx.rtf - 41:20 [Répondant : Il y’a eu aussi mo..] (46:46)	(Super)**

Codes:	[Service_prise-en-charge_fonctionnement]

No memos

**Répondant** : Il y’a eu aussi moins des cas dépistés par rapport aux cas attendu.

**P41: IA_04_BCZS_IS_02.docx.rtf - 41:21 [Répondant : Avec l’allure de l..] (48:48)	(Super)**

Codes:	[Service_prise-en-charge_fonctionnement]

No memos

**Répondant** : Avec l’allure de l’épidémie, les personnes soignées étaient obligées de s’impliquer dans la riposte et avec la réticence dont nous sommes entrés de faire allusion et avec les IT responsables des aires de sante, on était obligé de les associer à telle point qu’il puissent accompagner pour chercher à lever cette réticence et amener la population à adhérer aux activités de la riposte. Car a un certain moment, ils ont lâché les activités de routines au sien des structures pour se plonger dans la sensibilisation au sein de leurs aires de sante. Par rapport aux structures, il y’a eu certaines structures qui ont eu beaucoup des cas d’infection et qui témoignent réellement la charge de certains principes, qu’on devrait tenir compte pour éviter à ce que la maladie ne puisse transmettre d’une personne a une autre.

**P41: IA_04_BCZS_IS_02.docx.rtf - 41:22 [Répondant : Il y’a eu augmenta..] (50:50)	(Super)**

Codes:	[Qualité_soins_Prestataires]

No memos

**Répondant** : Il y’a eu augmentation de capacitation des équipes pour la prise en charge, qu’on a constaté que l’équipe de riposte à renforcer les équipes de préventions contre les infections et cela a augmenté la capacitation des prestataires pour la prise en charge des malades. Mais par rapport à l’autre volée, la population a semblée perdre confiance au personnel soignant en disant qu’il est entré de le livrer à l’équipe de riposte car la majorité de gens qu’on alertait mourrait, c’est ainsi que la population a perdu confiance au personnel soignant, c’est ainsi que l’accueil n’était pas facile même si on augmentait les stratégies pour regagner cette confiance, ça n’a pas été facile, ce qui a aussi cause la réticence est que l’équipe qui intervenait dans la sensibilisation, ce n’était pas des personnels soignant autochtones et la population se demandait pour les gens de sensibilisation ne sont pas des personnels soignants autochtones

**P41: IA_04_BCZS_IS_02.docx.rtf - 41:23 [Répondant : On ne peut pas dir..] (52:52)	(Super)**

Codes:	[Structure-Service_Utilisation_population]

No memos

**Répondant** : On ne peut pas dire qu’ils allaient quelque part, ils restaient dans la communauté, ils s’auto-soignaient, ils achetaient des produits au pharmacie, c’est pourquoi on a eu beaucoup des gens qui restent dans les officines de vente médicaments qui étaient dans les quartiers atteint, parce qu’il avait beaucoup des gens qui achetaient des médicaments et quand ils le soignaient ils se contaminaient à la même occasion.

**P41: IA_04_BCZS_IS_02.docx.rtf - 41:24 [Répondant : Le déficit était v..] (54:54)	(Super)**

Codes:	[Ressources_humaines_Disponibilité et perturbations]

No memos

**Répondant** : Le déficit était vraiment la parce qu’au niveau de l’équipe cadre, 80% des membres de ce dernier était plongée dans la riposte et ils étaient obligés d’y aller parce qu’ils devraient aider l’équipe à lever certaines réticences, et certaines activités ont souffert, la supervision ne se faisait plus, la promptitude de rapport était devenue bas, il y’a eu vraiment un impact négatif par rapport au fonctionnement des activités.

**P41: IA_04_BCZS_IS_02.docx.rtf - 41:25 [Répondant : A l’hôpital aussi ..] (56:56)	(Super)**

Codes:	[Ressources_humaines_Disponibilité et perturbations]

No memos

**Répondant** : A l’hôpital aussi parce qu’il a certains médecins qu’on a pris dans l’équipe de riposte qui étaient au CTE, et les autres qui étaient dans d’autres services ou activités de la riposte, c’est pourquoi même jusqu’à maintenant le personnel est encore instable par rapport à ça. Certaines structures fonctionnent difficilement car certains cadres sont partis dans la riposte.

**P41: IA_04_BCZS_IS_02.docx.rtf - 41:26 [Répondant : Cela veut dire que..] (58:58)	(Super)**

Codes:	[Ressources_humaines_Disponibilité et perturbations]

No memos

**Répondant** : Cela veut dire que vous trouverai un responsable qui est plongé dans la riposte, les autres, quand on prend quelqu’un de relève, il ne pourra pas faire rouler le service comme le responsable qui était là depuis longtemps.

**P41: IA_04_BCZS_IS_02.docx.rtf - 41:27 [Répondant : Au niveau de l’hôp..] (60:60)	(Super)**

Codes:	[Ressources_humaines_solution-local_Disponibilité et perturbations]

No memos

**Répondant** : Au niveau de l’hôpital, ils ont essayé de renforcer les personnels avec des nouvelles équipes et de fois on a demandé à ce que si quelqu’un est dans un service qu’il aye un temps de faire quelques heures au sein de l’hôpital, c’est comme même nous ici au niveau du BCZD, nous avons senti cette crise-là, nous nous sommes dit de prendre quelques heures pour les activités du BCZD de 8h00 à 10h00 et après on peut aller dans la riposte que d’y aller directement toute la journée, et c’était la même chose à l’hôpital, il fallait que ces acteurs qui sont dans la riposte, donnent certaines heures pour les activités de l’hôpital, c’est pourquoi s’il y’avait inquiétude dans un service, une personne pourrait aller un certain temps et l’autre pourrait sortir quand le premier serait de retour.

**P41: IA_04_BCZS_IS_02.docx.rtf - 41:28 [Répondant : La gratuite d’une ..] (62:62)	(Super)**

Codes:	[Gratuité_Conséquences]

No memos

**Répondant** : La gratuite d’une manière globale a été positive, nous avons senti qu’il a eu beaucoup des malades et d’autres pour la consultation, et on a senti qu’ils étaient vraiment malades et qu’ils trainaient à la maison pour faute de moyens. Dans le sens négatif, nous avons remarques qu’après l’interruption des soins gratuits il y’a eu baisse sensible de la fréquentation dans les structures et cela a eu un impact négatif parce que quand on était dans les soins gratuits, on supportait les charges et les personnels, mais quand les soins gratuits ne sont plus là toutes les charges reviennent à la structure et quand il y’a moins des personnes qui arrivent ça emboite un peu le fonctionnement et charge du personnel, c’est pourquoi vous avez vu qu’un moment donne les personnels étaient bien rémunérés pendant les soins gratuit parce que le gouvernement avait pris en charge aussi les personnels et quand il y’a eu chute libre de la fréquentation sans rien, et les malades qui n’ont rien et qui étaient habitues aux soins gratuits. C’était le chaud et le froid.

**P41: IA_04_BCZS_IS_02.docx.rtf - 41:29 [Répondant : On a lancé des com..] (64:64)	(Super)**

Codes:	[Gratuité_Information_population]

No memos

**Répondant** : On a lancé des communiques d’abord à travers la voix des ondes, à travers les structures et à travers les agents de la mobilisation qui étaient impliqués dans la riposte, on a essayé d’atteindre tout le monde par ces voies-là.

**P41: IA_04_BCZS_IS_02.docx.rtf - 41:30 [Répondant : Au début, il y’ava..] (66:66)	(Super)**

Codes:	[Gratuité_Rumeurs]

No memos

**Répondant** : Au début, il y’avait des rumeurs que c’était une occasion à ce qu’on puisse attraper tout le monde, mais après un certain temps avec les sensibilisations et les autres malades qui avaient emboité le pas, ils ont compris que ce n’est pas ça le cas, et enfin ils ont adhéré à ça.

**P41: IA_04_BCZS_IS_02.docx.rtf - 41:31 [Répondant : Jusque-là ce n’est..] (68:68)	(Super)**

Codes:	[Gratuité_Partenaire d'appui]

No memos

**Répondant** : Jusque-là ce n’est que le gouvernement, mais à partir de certains partenaires

**P41: IA_04_BCZS_IS_02.docx.rtf - 41:32 [Répondant : Comme je l’ai dit ..] (70:70)	(Super)**

Codes:	[Gratuité_Structures-Services_concernés]

No memos

**Répondant** : Comme je l’ai dit nous avons 300 structures et parmi ces 300, 39 étaient intégrées et parmi ces 39, 30 étaient optées pour la prise en charge des soins gratuits. C’était uniquement 30 sur les 300 structures, c’était insignifiant, c’est pourquoi il y a eu engouement autour de ces structures après adoption ou adhésion de la population.

**P41: IA_04_BCZS_IS_02.docx.rtf - 41:33 [Répondant : Il y avait un crit..] (72:72)	(Super)**

Codes:	[Gratuité_Structures-Services_concernés]

No memos

**Répondant** : Il y avait un criterium de sélection pour les 30 et tous les centres de santé (18) étaient là, et par rapport au centre hospitalier nous avions le HGR et les structures qui avaient notifiées au moins un cas positif parmi les structures intégrées

**P41: IA_04_BCZS_IS_02.docx.rtf - 41:34 [Répondant : Toutes les structu..] (74:74)	(Super)**

Codes:	[Gratuité_Défis]

No memos

**Répondant** : Toutes les structures n’ont pas été prise en compte mais il fallait qu’on prenne en compte toutes les structures pour qu’on puisse se rassurer que toute la population est couverte, et on a eu un engouement et ces structures étaient submergées par les malades. C’est à encourager mais il fallait élargir ça.

**P41: IA_04_BCZS_IS_02.docx.rtf - 41:35 [Répondant : Dans toutes les st..] (76:76)	(Super)**

Codes:	[Recouvrement_coûts-des-soins_avant gratuité]

No memos

**Répondant** : Dans toutes les structures les malades payaient leurs soins, ils venaient se faire soigner et on leur donnait la facture de tout ce qu’on a eu comme acticité autours de lui, et le malade payait cette facture. Il y’avait un moment où le taux de recouvrement de recettes était en dessous de 80% dans la majorité des structures et le cout de soins étaient élevé, parce que quand ce sont des structures privées les soins étaient dans le but lucratif

**P41: IA_04_BCZS_IS_02.docx.rtf - 41:36 [Répondant : Il y’avait des mut..] (78:78)	(Super)**

Codes:	[Recouvrement_coûts-des-soins_mutuelle]

No memos

**Répondant** : Il y’avait des mutuels mais on n’avait pas d’éléments là-dessus, il y’avait la MUSACOM et la MUSOSAM à qui ils payaient les soins mais le nombre des adhérant était vraiment minime

**P41: IA_04_BCZS_IS_02.docx.rtf - 41:37 [Interviewer : Savez-vous les c..] (79:80)	(Super)**

Codes:	[Gratuité_Conséquences] [Recouvrement_coûts-des-soins_mutuelle]

No memos

**Interviewer : Savez-vous les conséquences de la gratuite par rapport au fonctionnement de ces mutuels ? vous en avez à discuter de ça ?**

**Répondant** : On en parle souvent au niveau de comité de gestion, ces mutuelles ont souffert suffisamment à tel point qu’ils n’ont pas des nouveaux adhérents et jusqu’à maintenant ils continuent à souffrir parce qu’il y a des soins gratuits partiellement.

**P41: IA_04_BCZS_IS_02.docx.rtf - 41:38 [Répondant : Avant l’épidémie, ..] (82:82)	(Super)**

Codes:	[Médicaments_disponibilité_fosa]

No memos

**Répondant** : Avant l’épidémie, les structures s’approvisionnaient, nous avons un CDR MUSIENENE ASRAMESS et la grande partie s’approvisionnait sur le marché local et cela était à la base du prix élevé, par rapport au produit sanguin nous avons une chance d’avoir un centre de transfusion sanguine les gens s’approvisionne au CTS mais pendant l’épidémie nous avons connu des grandes difficultés car on ne pouvait plus prélever chez les donneurs bénévoles avec l’épidémie et le sang qu’on utilisait venait en dehors des aires de sante qui étaient en épidémie, cela a été à la base des difficultés, de couvrir toutes les aires de sante, tous les besoins de commandes des structures par rapport au produit sanguin. Et pendant les soins gratuit les structures étaient servies à travers le projet, on le donnait l’argent pour qu’ils payent eux-mêmes les médicaments sur le marché local

**P41: IA_04_BCZS_IS_02.docx.rtf - 41:39 [Répondant : Nous avons une dat..] (84:84)	(Super)**

Codes:	[SNIS_fonctionnement_Decrire_pdt MVE]

No memos

**Répondant** : Nous avons une date qui limite le dépôt des rapports des structures au niveau du BCZD et c’est au plus tard le 7 du mois suivant, mais nous avions constaté que pendant cela n’a plus été respecté, presque toutes les structures étaient plongées dans la riposte, avant l’épidémie nous étions dans la promptitude d’au-delà de 90% mais pendant l’épidémie, on a nagé autour de 50%, vraiment un impact négatif.

**P41: IA_04_BCZS_IS_02.docx.rtf - 41:40 [Répondant : Au fond quand vous..] (86:86)	(Super)**

Codes:	[SNIS_fonctionnement_Decrire_pdt MVE]

No memos

**Répondant** : Au fond quand vous parcourez le canevas vous sentez que la personne ne s’est pas mise pour bien analyser le rapport, et nous avions constaté qu’il n’y avait plus des réunions d’analyse avant l’expédition du rapport, vu cette absence, il avait beaucoup d’erreurs.

**P41: IA_04_BCZS_IS_02.docx.rtf - 41:41 [Répondant : Avant Ebola la mac..] (88:88)	(Super)**

Codes:	[Réunion_pdt-épidémie-Ebola]

No memos

**Répondant** : Avant Ebola la machine semblait bien marcher, au niveau des aires de sante il y avait des réunions, on sentait qu’il avait la cohésion entre la communauté et les structures mais pendant l’épidémie, ces réunions n’ont plus eu lieu et même au niveau de l’équipe cadre, il y a certaines semaines ou il n’y a pas eu des réunions cela pendant les périodes chaudes vu que les membres de l’équipe ou les prestataires étaient impliqués dans la riposte

**P41: IA_04_BCZS_IS_02.docx.rtf - 41:42 [nous avions constaté qu’il n’y..] (86:86)	(Super)**

Codes:	[Réunion_pdt-épidémie-Ebola]

No memos

nous avions constaté qu’il n’y avait plus des réunions d’analyse avant l’expédition du rapport, vu cette absence, il avait beaucoup d’erreurs.

**P41: IA_04_BCZS_IS_02.docx.rtf - 41:43 [Répondant : Comme je l’ai dit,..] (90:90)	(Super)**

Codes:	[Réunion_pdt-épidémie-Ebola] [Souhait_Changement_gestion_futures_épidémies]

No memos

**Répondant** : Comme je l’ai dit, on n’avait jamais vécu de telles situation, c’est pourquoi en un certain moment, on se donnait espoir en disant que tout va passer, on a commencé quelques activités. Comme plus de 80% était plongé dans la riposte, on c’était dit qu’un moment on sera pour les activités de routines, et un moment pour les activités de riposte, car la riposte fait partie des grandes activités de la zone de sante. Si l’épidémie reviennent, on a déjà eu de l’expérience, et on a pris les mesures pour la planification des certaines choses pour les activités de routine ne puissent pas souffrir.

**P41: IA_04_BCZS_IS_02.docx.rtf - 41:44 [Répondant : Si ces équipes on ..] (92:92)	(Super)**

Codes:	[Gouvernance_soins santé]

No memos

**Répondant** : Si ces équipes on ne saurait pas s’en sortir, ils nous ont trop aider, nous avons beaucoup appris de leur part,

**P41: IA_04_BCZS_IS_02.docx.rtf - 41:45 [Répondant : Je ne sais comment..] (94:94)	(Super)**

Codes:	[Gouvernance_soins santé]

No memos

**Répondant** : Je ne sais comment dire, mais ils nous aider à nous montrer quelles décisions prendre, une manière de se comporter devant une population furieuse, face à des telles situations, surtout en cette période de l’épidémie.

**P41: IA_04_BCZS_IS_02.docx.rtf - 41:46 [Répondant : Si de telles épidé..] (96:96)	(Super)**

Codes:	[Souhait_Changement_gestion_futures_épidémies]

No memos

**Répondant** : Si de telles épidémies reviennent, j’aimerais qu’on implique correctement et en première place les équipes locales et qu’on vient en appuis de ces équipes-là. Ce qui a été l’inverse pendant l’épidémie actuelle et cela a été à la base de la réticence de la population.

**P41: IA_04_BCZS_IS_02.docx.rtf - 41:47 [Répondant : L’autre chose c’es..] (98:98)	(Super)**

Codes:	[Commentaire-Recommandation_Répondant]

No memos

**Répondant** : L’autre chose c’est la gestion, les équipes sont en train de rentrer, et ils sont en train de capacité les locaux, nous aimerions que cette gestion poste-riposte se passe quand il y a encore des moyens. Autre chose, nous avons dit qu’on démoli certaines structures, nous aimerions qu’on puisse faire quelque chose pour le réhabiliter, qu’on appui ces structures qui ont été endommager lors de l’épidémie.

**P42: IA_04_BCZS_MCZS.rtf - 42:1 [Répondant : la zone de santé d..] (2:2)	(Super)**

Codes:	[Début-Progression_Epidémie MVE]

No memos

Répondant : la zone de santé de Katwa, il faut savoir qu’elle s trouve dans la ville de Butembo, c’est une deux zones de santé de la ville de Butembo et qui est entrée en épidémie le 5 septembre 2018 et elle est entrée en épidémie au travers d’un malade contact jamais vu qui était à Beni et qui a fui Beni pour venir se cachait à Butembo et arriver à Butembo, elle se cachait dans plusieurs maisons y compris dans la zone de santé de Katwa et puis elle allait se trouver dans un ménage dans la zone de Butembo où elle a tombé gravement malade et elle a été méné dans une structure de la zone de santé de Butembo qu’on appelle centre de santé Utshundo et quelques jours elle est devenue très malade pour être conduit aux cliniques universitaires de graben, là c’est toujours la zone de santé de Butembo et c’est là qu’elle a rendu l’âme alors le prélèvement fait montrer que c’est quelqu’un qui avait déjà la MVE et c’est comme ça que toute la ville est tombée en épidémie ainsi que notre zone de santé.

**P42: IA_04_BCZS_MCZS.rtf - 42:2 [Répondant : Oui, c’est que j’a..] (4:4)	(Super)**

Codes:	[Considération_Ebola_population]

No memos

Répondant : Oui, c’est que j’ai retenu de la MVE, c’est que d’abord c’est une nouvelle maladie qui est apparue dans la ville, une maladie qui n’était pas connue, qui n’était pas maitrisé par le professionnel de la santé de na zone et une maladie qui malheureusement qui a créé beaucoup des rumeurs. C’était vraiment des rumeurs qui allaient dans beaucoup de sens et qui a créé une situation sanitaire et sécuritaire très précaire et cela malheureusement a conduit à beaucoup des résistances, à plusieurs résistances et ces résistances comme vous le savez c’est une maladie, c’est une épidémie et comme l’épidémie se gère d’abord par la population, ça était très difficile de la maitriser, voilà aujourd’hui encore nous sommes dans l’épidémie et la population bien qu’elle a compris parce qu’on a été vraiment appuyé par les partenaires, par l’Etat et nous sommes encore en épidémie et la situation est relativement stabilisée et nous pensons que d’ici là elle sera jugulée. Mais c’est que j’ai plus retenu ce que nous avons un besoin dans la zone de santé, je dirai pour notre zone de santé, ce besoin ressentit de pouvoir renforcer le système sanitaire en matière de surveillance mais aussi en moyen de gestion, le moyen qui soit disponible.

**P42: IA_04_BCZS_MCZS.rtf - 42:3 [Répondant : la plus grande rum..] (6:6)	(Super)**

Codes:	[Considération_Ebola_population]

No memos

Répondant : la plus grande rumeur, c’est qu’il y avait le dénie de la maladie, plutôt la MVE n’existe pas et pour ça par après le vaccin qui était n’était pas un vrai vaccin mais c’était amené pour exterminer la population. La troisième rumeur quand on a compris que la maladie existait, c’était que cette maladie, on l’amenait pour raison d’argent et enfin, une rumeur surtout qui circulait, surtout que cette maladie a fait qu’il n’ait pas vote, dans cette contrée, on a dit qu’on a largué la maladie pour que la population ne puisse pas avoir droit au vote

**P42: IA_04_BCZS_MCZS.rtf - 42:4 [Répondant : Bon, il faut dire ..] (8:8)	(Super)**

Codes:	[Répondant_Responsabilité_pdt MVE]

No memos

Répondant : Bon, il faut dire que je suis le médecin chef de zone ad intérim, j’étais à l’époque médecin chef de staff et puis médecin directeur de l’HGR de Katwa et entant que médecin chef de staff, j’étais dans la commission prise en charge médicale où j’ai eu à œuvre dans le CTE Butembo d’abord, puis j’ai été président de la commission prise en charge au niveau de la sous-coordination de Katwa et puis j’ai pris la charge de médecin chef de zone ad intérim et comme je suis médecin chef de zone, j’ai eu à être membre de la coordination pour gérer la riposte dans la sous coordination de Katwa

**P42: IA_04_BCZS_MCZS.rtf - 42:6 [Répondant : dans la qualité de..] (13:13)	(Super)**

Codes:	[Qualité_soins_Prestataires]

No memos

Répondant : dans la qualité de soins, je peux parler par exemple de nombre de tours de salle qui est prévu ou alors le nombre des consultations prévues par un médecin, on a vu ça augmenté et nous on a dit non, donc nous prestataires de soin ont risque de ne pas nous concentrer sur les cas

**P42: IA_04_BCZS_MCZS.rtf - 42:7 [Mais la difficulté qu’on a con..] (11:11)	(Super)**

Codes:	[Qualité_soins_Prestataires]

No memos

Mais la difficulté qu’on a constaté c’est vrai, quand un afflux très massif des malades alors que les prestataires n’étaient pas encore prêt, on a constaté quand même une baisse de la qualité des prestations et cette baisse de la qualité des prestations a été constaté à cause du nombre des prestataires et cela a créé une chute de la qualité de soins.

**P42: IA_04_BCZS_MCZS.rtf - 42:8 [Répondant : Bon, ce qui est fr..] (10:10)	(Super)**

Codes:	[Structures-Services_fonctionnement]

No memos

Répondant : Bon, ce qui est frai ce que nous avons pu constater en un certain moment que les malades n’arrivent pas à l’hôpital pour la raison évidente que si vous arrivez à l’hôpital, on risque de vous prendre et de vous amener au CTE parce que la maladie n’était pas encore bien comprise et par après avec PDSS, le projet du gouvernement qui a subventionné les soins et au fur à mesure que la population a compris que la maladie est une réalité, on a vu un afflux des malades dans des formations sanitaires.

**P42: IA_04_BCZS_MCZS.rtf - 42:9 [Répondant : ils fuyaient l’hôp..] (15:15)	(Super)**

Codes:	[Structure-Service_Utilisation_Alternatives_population]

No memos

Répondant : ils fuyaient l’hôpital mais ils restent à la maison et les hôpitaux commençaient maintenant à recevoir des malades graves qui s’étaient déjà aggravés et vous aurez à constater qu’il y a eu non seulement des décès liés à la MVE qui ont augmentés mais aussi des décès liées à d’autres pathologies qui ont augmentés du fait des consultations tardives

**P42: IA_04_BCZS_MCZS.rtf - 42:10 [Répondant : la crainte d’être ..] (17:17)	(Super)**

Codes:	[Considération_CTE_population]

No memos

Répondant : la crainte d’être envoyé au CTE était due aux rumeurs càd qu’ils avaient peur qu’on leur colle un diagnostic de MVE pour lequel eux ne croyaient pas et pour cette raison ils pensaient que s’ils arrivent à l’hôpital on va dire qu’ils ont la MVE pour aller au CTE et là effectivement arrivée au CTE parce que tout est connecté parce qu’un malade MVE s’il consulte tardivement, les chances de survie sont réduites et là nous avions constaté tout au début quand nous travaillions au CTE que la plupart des patients MVE diagnostiqués positif dans la suite ils venaient très en retard et c’est pour cela qu’il y avait un taux de décès très élevé

**P42: IA_04_BCZS_MCZS.rtf - 42:11 [Répondant : la vaccination de ..] (19:20)	(Super)**

Codes:	[Service_vaccination_Fonctionnement]

No memos

Répondant : la vaccination de routine a subi des perturbations pour des raisons d’abord sécuritaire parce que je ne me rappelle plus du mois mais les prestataires de soins commis à la vaccination avaient grevé parce qu’il y avait une grève pour des raisons sécuritaire. Toutes les fois que les équipes voulaient faire la vaccination, ils étaient caillasses et menacés raison pour laquelle il y a eu une grève pour ça donc là c’était un élément sécuritaire qui a fait que la vaccination puisse avoir des difficultés dans sa mise en œuvre de ses activités.

La deuxième chose en rapport avec la vaccination, c’est encore en rapport avec les rumeurs, ces rumeurs ont fait que quand les équipes voulaient aller vacciner, on pensait qu’ils donnaient le vaccin à la MVE et là c’était une difficulté de compréhension donc la sensibilisation était amenée dans ce sens pour expliquer aux gens que la vaccination devait continuer maintenant la troisième difficulté, je pense qu’on passer parce que c’est toujours dans le cadre sécuritaire.

**P42: IA_04_BCZS_MCZS.rtf - 42:12 [Répondant : je crois en moins ..] (22:22)	(Super)**

Codes:	[Instructions_pdt_MVE]

No memos

Répondant : je crois en moins que je ne l’abuse, je ne le sais pas comme vous le savez, peut-être que je sois à la zone mais depuis que je suis à la zone, je n’ai pas connaissance d’une lettre pareille pour interdire la vaccination mais en rapport avec la question d’interdire certains actes évasifs qui pourraient faciliter la contamination, ça ce n’était pas une instruction générale, c’était des instructions à partir de chaque corporation pour dire qu’on continue à piquer alors qu’on n’a pas raison alors qu’on peut se fier à un traitement empirique et là en un certain moment on se dit que le TDR pour le paludisme, on pouvait ne pas le faire et au besoin on pouvait faire la goutte épaisse, donc on a réduit certains actes que nous appelons évasifs pour essayer de couper la chaine de transmission parce que nous avions eu des maladies nosocomiale, la MVE nosocomiales dans certaines FOSA

**P42: IA_04_BCZS_MCZS.rtf - 42:13 [Répondant : tout dépend de la ..] (24:24)	(Super)**

Codes:	[Service_chirurgie_fonctionnement] [Service_maternité_fonctionnement]

No memos

Répondant : tout dépend de la période mais il n’y a pas eu une différence sensible par rapport aux accouchements, les accouchements, ce ne sont pas des maladies mais ce sont des éléments que nous n’avons pas vraiment vu une différence remarquable au contraire on avait constaté d’ailleurs que la subvention des soins par PDSS, quand c’était une gratuité totale, on a constaté un afflux de malades au niveau des FOSA et cela a augmenté d’ailleurs à un moment donné le chiffre en terme des accouchements, de la chirurgie, en tout cas ça augmentait et je crois entre septembre et décembre ou janvier.

**P42: IA_04_BCZS_MCZS.rtf - 42:14 [Répondant : avant Ebola, les F..] (26:26)	(Super)**

Codes:	[Recouvrement_coûts-des-soins_avant gratuité]

No memos

Répondant : avant Ebola, les FOSA travaillaient sous format des prestations par les malades et aussi nous avons deux mutuelles de santé dont la pénétration est encore faible et il y a donc un système de mutualisation et un système de paiement cache. C’est ce qui était, avant Ebola

**P42: IA_04_BCZS_MCZS.rtf - 42:16 [Répondant : A ma connaissance,..] (31:31)	(Super)**

Codes:	[Gratuité_Partenaire d'appui]

No memos

Répondant : A ma connaissance, nous n’avons pas d’autres partenaires par rapport à la gratuité des soins.

**P42: IA_04_BCZS_MCZS.rtf - 42:17 [Et maintenant quand la MVE a s..] (29:29)	(Super)**

Codes:	[Gratuité_Evolution]

No memos

Et maintenant quand la MVE a surgi l’Etat congolais à travers PDSS à subventionner les soins dans la globalité et cela je crois c’était jusqu’au mois de décembre où tous les soins étaient gratuits et par la suite les soins ont été subventionné par service ou par exemple au niveau d’une structure avec paquet complémentaire d’activités, un HGR, c’est seulement l’hospitalisation en médecine interne et la pédiatrie qui sont subventionnées donc c’est peu de manière ramasser ce qui se fait par rapport aux soins gratuits

**P42: IA_04_BCZS_MCZS.rtf - 42:18 [Répondant : non, c’était par a..] (28:28)	(Super)**

Codes:	[Recouvrement_coûts-des-soins_avant gratuité]

No memos

Répondant : non, c’était par acte, ce n’était pas un forfait

**P42: IA_04_BCZS_MCZS.rtf - 42:19 [Répondant : pendant la gratuit..] (33:33)	(Super)**

Codes:	[Gratuité_Conséquences_motivation_prestataire]

No memos

Répondant : pendant la gratuité, les agents ont été mieux payés par rapport à la prime qu’ils recevaient avant la gratuité parce que le paiement se faisait sur base du nombre des malades qui venaient dans la structure sanitaire comme il y a eu afflux, il y a eu plus d’argent est entré et je pense que les prestataires ont été mieux payés qu’avant

**P42: IA_04_BCZS_MCZS.rtf - 42:20 [Répondant : les médicaments, l..] (35:35)	(Super)**

Codes:	[Médicaments_disponibilité_fosa]

No memos

Répondant : les médicaments, là encore on va revenir sur les contrats qui ont été signé avec les FOSA, le contrat était qu’on amène tout l’argent, c’était une boite noire et ce sont des formations sanitaires qui font des réquisitions et qui amènent des médicaments et après ça a changé et on a demandé au CDER de fournir les médicaments et un montant je pense entre 20 ou 30% de l’argent qui devait être amené à l’hôpital était retenu pour que cette pharmacie le CDER puisse donner les médicaments bon ça était une difficulté parce que dans les FOSA quand cette mesure a été prise, on a commencé à constater dans certaines FOSA, ils ont commencé à tomber dans les ruptures des médicaments

**P42: IA_04_BCZS_MCZS.rtf - 42:21 [Répondant : je ne pense pas, i..] (37:37)	(Super)**

Codes:	[Médicaments_disponibilité_fosa]

No memos

Répondant : je ne pense pas, il y a eu un problème de communication entre la CDER et les zones de santé pour approvisionner en médicaments

**P42: IA_04_BCZS_MCZS.rtf - 42:22 [Répondant : Oui, je pense que ..] (39:39)	(Super)**

Codes:	[Ressources_humaines_Disponibilité et perturbations]

No memos

Répondant : Oui, je pense que nous sommes en train de le constater jusque maintenant parce qu’on avait constaté des perturbations des activités de routine de la zone de santé et vous savez quand il y a épidémie donc toutes ses ressources, tout le monde nous sommes concentrés pour que combattre cette maladie, la contenir et vue ces multiples activités, il y a plusieurs activités de routine qui à un moment souffrent dès part l’horaire qui est commis aux activités de la riposte et qui nous devons encore le redire que la surveillance fait partir des activités de la zone de santé et quand la maladie est là, elle est bien constaté, vous savez que tout le monde est vraiment orienté vers cette épidémie pour pouvoir la contenir et donc c’est vrai oui, il y a eu quand même une perturbation significative des activités régulières ou traditionnelles de nos formations sanitaires ;

**P42: IA_04_BCZS_MCZS.rtf - 42:23 [Répondant : je peux parler par..] (41:41)	(Super)**

Codes:	[Supervision_pdt-épidémie-Ebola]

No memos

Répondant : je peux parler par exemple des activités de planification des supervisions de plusieurs FOSA où on se trouve avec moins des réalisations selon les planifications

**P42: IA_04_BCZS_MCZS.rtf - 42:24 [Répondant : Oui, pour ce qui e..] (43:43)	(Super)**

Codes:	[Ressources_humaines_Disponibilité et perturbations]

No memos

Répondant : Oui, pour ce qui est des FOSA, je vous disais que la qualité des soins dépend de nombre des personnels et de l’afflux des malades. Cette qualité a été un peu entamé par rapport au nombre des prestataires parce qu’en un moment donné il y avait un afflux massif des malades et par rapport aux heures sue les prestataires sont supposés faires et par moment on devait les allonger et comprenez que quand quelqu’un est fatigué, il ne sera pas bien concentré pour le travail

**P42: IA_04_BCZS_MCZS.rtf - 42:25 [Répondant : non, ce n’est pas ..] (45:45)	(Super)**

Codes:	[Ressources_humaines_situation_grève]

No memos

Répondant : non, ce n’est pas cette même grève parce que là il faut savoir qu’Ebola n’a pas commencé dans la ville de Butembo. Ebola avait commencé à Mangina autour de 80 km de nous ici et la grève des infirmiers, je pense que c’est de celle-là que vous parlez, je crois c’était au mois de mai que cette grève avait été enclenché par les infirmiers pour raison je crois des primes, voilà de primes et ils avaient déjà enclenché cette grève mais la grève dont je parle c’est la grève des prestataires de soins qui avaient commencé le travail et qui étaient menacé sur terrain parce qu’on croyait qu’ils venaient donner le vaccin contre la MVE

**P42: IA_04_BCZS_MCZS.rtf - 42:26 [Répondant : il faut quand même..] (47:47)	(Super)**

Codes:	[SNIS_fonctionnement_Decrire_pdt MVE]

No memos

Répondant : il faut quand même l’accepter qu’il y a eu quand même une perturbation au niveau de la collecte des données. Vous savez à un moment nous nous sommes rendus compte que nous avions des FOSA supplémentaires et les formations spécialisées, CT et CTE qui étaient en train de recevoir des patients, prendre en charge ces patients, les soigner et les guérir alors ces données-là, là c’est déjà au niveau de la collecte des données, les données échappaient à la collecte au niveau de la base comme vous les dites. Ça c’était une difficulté qu’on a constaté récemment corrigé malheureusement ça ne pourra pas avoir un impact significatif sur toutes les données mais ça était quelque chose qui a été constaté que les données CT et CTE au niveau de la collecte des données donc nous ne les avons pas capitalisé par rapport à la collecte donc la transmission vers l’analyse et consort donc ça se faisait au niveau de ces formations sanitaires spécialisées. Alors par rapport aux autres activités au niveau des FOSA, je pense nous n’avons pas connu beaucoup des problèmes, seulement nous devons nous dire qu’on a eu un retard de faire l’encodage et la transmission parce qu’il y a des activités qui se compriment et cela nous a fait chuté par rapport au timing qui était minime par rapport à ce qu’on devait faire.

**P42: IA_04_BCZS_MCZS.rtf - 42:27 [Répondant : je pense que les p..] (49:49)	(Super)**

Codes:	[Gouvernance_soins santé]

No memos

Répondant : je pense que les partenaires ont été un ouf de soulagement pour la zone de santé dès part d’abord leur appui multisectoriel autour de la MVE parce que leur présence a révélé plusieurs défaillances de notre système sanitaire au niveau e la zone de santé. Là il faut se le dire parce que je donne un petit exemple nous avons pu constater par exemple qu’il n’y avait aucune structure sanitaire qui avait un service d’isolement donc ça c’est vraiment une difficulté et nous avions aussi constaté que notre surveillance n’est pas bien assise avec toutes les équipes qui doivent être au niveau de la surveillance en commençant par la cellule d’animation communautaire que les partenaires nous ont aidé d’ailleurs à mettre en place donc cette structure comme CAC malheureusement n’existait pas dans notre zone de santé. Alors autant des problèmes de partenaires et des acteurs du niveau central nous ont fait remarqué nous-mêmes et aussi bien sûr il y a aussi cette capacitation en termes de travail que nous devons rendre, là nous devons compris qu’il y avait encore beaucoup des choses à apprendre en termes des capacitations. Là pour ce qui est de la gouvernance, moi je dirai qu’il y a eu quand même certains éléments à améliorer par rapport à la gouvernance parce que nous avons constaté que notre échelonnement par rapport à la gouvernance est légèrement perturbée parce que la riposte était gérée à partir du niveau central et vous constaterez quand on a commencé à parler de programme de transfert des compétences donc tout ça signifie que le leadership autour de ça, transfert des compétences, je pense que ça ne s’est pas fait aussi vite, on devait d’abord combattre la maladie pour faire ça donc c’était un des points à améliorer qui devait se faire de manière beaucoup plus précoce mais dans la façon de transmettre certaines compétences

**P42: IA_04_BCZS_MCZS.rtf - 42:28 [Répondant : donc ce transfert,..] (51:51)	(Super)**

Codes:	[Gouvernance_soins santé]

No memos

Répondant : donc ce transfert, il y a le niveau central, n’est-ce pas ? Par après il y a le niveau intermédiaire, je parlais de la DPS et puis, il y a maintenant le niveau opérationnel

**P43: IA_05_AS_IT_01.rtf - 43:1 [Répondant : justement comme vo..] (2:3)	(Super)**

Codes:	[Début-Progression_Epidémie MVE]

No memos

Répondant : justement comme vous venez de le dire nous sommes dans l’aire de santé Mangina, cette aire de santé a une population de 27.661. L’épidémie à la MVE a été déclarée le 1^er^ Août 2018 par le ministre de la santé mais avant cette épidémie, il y avait des décès dans la structure, les décès qu’on ne comprenait pas. C’était à partir de mois de juin, juillet, il y avait des décès inexplicable, par jour on pouvait avoir 4 décès et d’autres décès, on pouvait avoir des signes hémorragiques même nous-mêmes on se demandait c’est quelle maladie ? C’est du jamais vu, qu’est-ce que ce ? Donc après décès, il y avait des signes hémorragiques donc il y avait une contamination directe avec les gardes malades. Quand un malade décédait, deux jours après, son garde malade revient avec des signes similaires et pour nous cela n’était pas normal. Finalement on a alerté la zone de santé, nous leurs avons dit que le nombre des décès, ça ne va pas et les signes que les cadavres manifestent ça ne va pas. C’est vraiment une chose du jamais vu et ça nous étonnait et ça nous surprend, la zone de santé est venue pour faire l’enquête et le mode de contamination donc c’était une contagion directe. C’est qui nous avait tiqué, il y avait à Masindende, le nom d’un célèbre quartier, il y avait une dame qui était décédée après son décès, sa sœur qui était garde malade était décédée et dans la même famille endéans de trois semaines, il y avait déjà huit décès donc deux, trois personnes qui étaient venues au deuil sont décédées après donc endéans de trois semaines, il y a déjà huit personnes décédées dans une même famille donc ça nous a tiqué et finalement la zone est venue pour faire la descente et investiguer pour voir eu juste, c’est quoi ? Et vu les signes hémorragiques qui avaient aussi tiqué les membres de l’équipe cadre, on a été obligé d’envoyer les échantillons au niveau de Goma et quand on a envoyé les échantillons au niveau de Goma, il y avait notre infirmier qui était atteint, c’était au mois de juillet et cet infirmier présentait des signes et des fièvres qui ne chutaient pas donc une fièvre persistante, les émissions des selles résistantes et cela nous avait tiqué et tous les infirmiers qui travaillaient aux soins intensifs ont été atteints et le premier infirmier qui était atteint, c’était au mois de juillet avant que l’épidémie ne soit déclarée donc il était parmi les échantillons qu’on avait envoyé. Avant on avait envoyé six échantillons et parmi les six échantillons, il y a quatre qui étaient positifs donc des malades de notre structure entre autre notre infirmier, curieusement lui, il était guéri miraculeusement donc sans avoir le traitement spécifique donc on le soignait avec ce qu’on avait donc d’une façon symptomatique donc on soignait les symptômes mais curieusement il a été guéri mais il avait encore une positivité et on l’a suivi quelques jours au CTE et il était complétement guéri.

Et quand on avait déclaré le 1^er^ Août que c’était l’épidémie à MVE, curieusement le gouvernement a envoyé directement l’équipe de la riposte et l’équipe était directement venue le 2 Août pour s’installer ici pour prendre la situation au sérieux et nous aussi nous avons eu peur donc c’était une épidémie dangereuse et nous nous sommes des religieuses, on était à Butembo pour une retraite alors on apprenait les décès chaque jour et quand on a déclaré que c’est une épidémie alors moi on m’a obligé de venir ici et de laisser les sœurs à Butembo pour venir la situation et je suis arrivée 5 Août et toute l’équipe de la riposte était déjà sur terrain.

**P43: IA_05_AS_IT_01.rtf - 43:2 [Répondant : dans la structure,..] (5:5)	(Super)**

Codes:	[Début-Progression_Epidémie MVE_Agents affectés]

No memos

Répondant : dans la structure, il y a eu dix infirmiers et un médecin, heureusement tous étaient guéri et la plupart de ces infirmiers travaillaient aux soins intensifs

**P43: IA_05_AS_IT_01.rtf - 43:3 [Répondant : au début, la popul..] (7:7)	(Super)**

Codes:	[Considération_Ebola_population]

No memos

Répondant : au début, la population avait peur du nombre des décès donc l’équipe de la riposte était venue quand la population avait peur, le fait que dans une famille, 5 personnes et la population à Mangina là où l’épidémie a commencé avait peur parce qu’on n’a pas encore vu ça endéans une semaine, il y avait 8 décès. Dans une même famille, ici deuil, ici deuil et quand l’équipe de la riposte était venue, la population était curieuse de savoir pourquoi ce nombre des décès ?et cette là a permis que la population puisse accueillir l’équipe de la riposte sans trop des résistances. Alors la population était prête pour accueillir leur message, la population était prête pour dire qu’est-ce que nous pouvons faire pour diminuer le nombre des décès comme ça ou bien pour ne pas se faire contaminer pas d’une manière générale ici à Mangina, il n’y a pas eu une résistance contre la riposte.

**P43: IA_05_AS_IT_01.rtf - 43:4 [Répondant : ici non parce que ..] (9:9)	(Super)**

Codes:	[Agression-Menaces_Structure-Prestataire_raisons]

No memos

Répondant : ici non parce que les gens avaient eu peur tellement qu’après le décès il y a eu le saignement impossible et comme la population avait peut er cette peur a permis qu’on puisse accueillir les gens de la riposte comme des sauveurs, chacun avait peur d’être contaminé et quand l’équipe de la riposte est venue nous les avons accueilli comme des sauveurs et justement quand on a passé le message que voici la maladie qui tue les gens et on a commencé avec différentes formations pour expliquer qu’Ebola, c’est quoi ? Comment ça se transmet ? Et ça, ça nous a permis et grâce à nos relais aussi parce que nous avons des relais actifs et on a continué par la formation des différents relais qui nous ont aidé d’aller sensibiliser dans la communauté. On a utilisé les différentes chaines de radio locales puisqu’ici nous avons trois chaines de radio. On a utilisé toutes ces chaines-là pour la sensibilisation et dans les églises, chaque jour pour la sensibilisation pour savoir comment se prévenir quand vous voyez tel signe, tel signe, il faut venir directement à la structure

**P43: IA_05_AS_IT_01.rtf - 43:5 [Répondant : au début-là, comme..] (11:11)	(Super)**

Codes:	[Début-Progression_Epidémie MVE]

No memos

Répondant : au début-là, comme nous sommes des africains et nous les africains on voit d’abord la sorcellerie. Hum sept personnes dans la famille, il doit y avoir un problème de sorcellerie et tout au début, on disait qu’il avait une famille qui avait mangé le chat mais finalement quand on a fait des analyses et on a compris que ce n’était pas vrai parce que la première personne qui était décédée et qui présentée le signe de saignement et quatre jours après qu’on a eu cette rumeur qu’ils ont mangé le chat de quelqu’un et le propriétaire de ce chat s’est vengé et il a jeté le mauvais sort sur cette famille mais un peu après quand on a essayé de faire le sondage, cette personne qui avait amené la maladie si on peut le dire comme ça et comme notre population vit des travaux champêtres, il travaillait très loin dans la brousse et là il y avait un camp militaire des gens qui étaient venus dans une autre province et c’est une province là où était Ebola avant puisque les militaires aussi ont les mutés, ils quitte une province pour une autre et il s’est fait que ce militaire est mort là au champ et on avait obligé la population de s’occupait des funérailles de ce cadavre alors le monsieur-là était parmi les gens qui ont été choisi pour enterrer le cadavre et on pense que ce monsieur était contaminé par cette personne morte qui était venue d’une autre province. Ça c’est venu après avoir menée des enquêtes de cette première personne qui avait manifestée des signes comme –ça parce qu’on a reconstitué son histoire, il vit où ? Il fréquente qui ? Il vient de quelle village et après on a eu l’information que quelqu’un était décédée au champ et lui fait des travaux champêtres et après on a obligé la population de s’occuper de son enterrement et lui était parmi les gens qui manipulaient la dépouille

**P43: IA_05_AS_IT_01.rtf - 43:6 [Répondant : ça c’est venu aprè..] (13:13)	(Super)**

Codes:	[Considération_Ebola_population]

No memos

Répondant : ça c’est venu après ici à Mangina, cette conception, elle est venue ici après peut-être six mois après

**P43: IA_05_AS_IT_01.rtf - 43:7 [Répondant : cette conception e..] (15:15)	(Super)**

Codes:	[Considération_Ebola_population]

No memos

Répondant : cette conception est venue vu le nombre des véhicules, nous sommes un village mais chaque personne de l’équipe de la riposte avait son véhicule et dans ce véhicule, il y avait une personne tout au plus deux et gens ont commencé à dire que ces gens sont venus pour nous montrer les véhicules, c’est pour une démonstration ou c’était quoi ? En plus de cela il y avait aussi de l’argent qu’ils recevaient comme prime et dans notre pays on a du mal à atteindre 100$ par mois mais ici un relais pour l’encouragerait, il touchait 300$ et les autres, on écoutait 400, 500$. Alors cet esprit d’Ebola business est venu puisque les gens ont senti que les gens de l’équipe de la riposte voulaient qu’Ebola puisse se pérenniser pour gagner aussi sa vie. Et c’est dès là qu’est venu cette conception, ah c’est un business-ça et cette conception est venu parce qu’on demandait aux gens de la riposte de faire le confinement donc pour ne pas éparpiller cette épidémie qui vient d’être déclarée, fermer les frontières, fermer les routes que les gens n’aient plus à Beni, à Butembo, non nous on sentait que les gens de la riposte ne voulaient pas faire ce confinement-là mais c’était une demande de la population au début-là mais on ne fermait pas les frontières , les barrières alors ce passage-là a permis qu’Ebola ait à Beni, Beni vers Butembo et même si vraiment l’épidémie était vive et danse, on pouvait limiter les voyages sans problème. Si on avait pris les mesures comme on l’avait pris pour coronavirus, on ne devait pas avoir le nombre qu’on a eu puisqu’il n’y avait aucun changement parce que les gens se transportent sur les motos et sur les véhicules,, il y avait des contacts, les gens voyageaient en voiture et on se touchait parce qu’il n’y avait aucune mesure pour dire qu’on va se confiner et il y a des barrières, rien

**P43: IA_05_AS_IT_01.rtf - 43:8 [Répondant : oui, ça nous avons..] (17:17)	(Super)**

Codes:	[Structure-Service_Fermeture_pdt MVE]

No memos

Répondant : oui, ça nous avons senti ça d’une façon énorme puisque là ils nous ont plus rappelé la prévention et le contrôle des infections et d’est comme si nous avions relâché ce que nous avions appris au niveau des études, même le port de gants, on a relâché mais grâce à cette équipe de la riposte, on a dit non, non, sin vraiment vous respectez bien les mesures de prévention, vous serez sauvés et nous avons pris cela au sérieux donc ils nous ont vraiment aidé à renforcer et à réveiller encore notre conscience par rapport à comment se prévenir sinon on avait déjà relâché.

**P43: IA_05_AS_IT_01.rtf - 43:9 [Répondant : non, en tout cas t..] (19:19)	(Super)**

Codes:	[Structures-Services_fonctionnement]

No memos

Répondant : non, en tout cas tous les services ont fonctionnés comme il faut et ce qui nous a aidés à faire fonctionnés et développé la structure, c’est le gouvernement en collaboration avec les organisations internationales qui sont venus nous appuyer par les soins gratuits. Ça nous a beaucoup aidés pour récupérer les cas qui se séquestrent dans les maisons

**P43: IA_05_AS_IT_01.rtf - 43:10 [Répondant : Non, non, la vacci..] (21:21)	(Super)**

Codes:	[Service_vaccination_Fonctionnement]

No memos

Répondant : Non, non, la vaccination n’a pas fonctionné puisque nous avons interrompu pendant presque six mois même plus. On avait interrompu dans l’objectif d’arrêter le contact étant donné que la vaccination crée des contacts avec des parents et des enfants, on a interrompu et on se dit voyons d d’abord puisque l’épidémie est encore vive, arrêtons mais comme mesure de barrière.

**P43: IA_05_AS_IT_01.rtf - 43:11 [Répondant : c’était au niveau ..] (23:23)	(Super)**

Codes:	[Instructions_pdt_MVE]

No memos

Répondant : c’était au niveau de la zone

**P43: IA_05_AS_IT_01.rtf - 43:12 [Répondant : c’était verbal] (25:25)	(Super)**

Codes:	[Instructions_pdt_MVE]

No memos

Répondant : c’était verbal

**P43: IA_05_AS_IT_01.rtf - 43:13 [Répondant : par rapport au lab..] (27:27)	(Super)**

Codes:	[Service_laboratoire_fonctionnement]

No memos

Répondant : par rapport au laboratoire, il y avait justement quelques examens qu’on ne faisait plus même au niveau de la structure, on choisissait dons comme on le faisait de routine, tout malade qui venait on prélevait surtout le TA au moins la température avec le thermo flash oui même avec le TA on choisissait vraiment ça c’est un cas qui manifeste une pathologie cardiaque donc on choisissait le cas mais ce n’était pas de routine pour tout malade de vérifie son TA. Ça vraiment ça nous a dérangé de faire le prélèvement de tout malade mais on avait un peu de réserve parce que nous étions dans la période d’une épidémie

**P43: IA_05_AS_IT_01.rtf - 43:14 [Répondant : ça fonctionnait pa..] (29:29)	(Super)**

Codes:	[Service_chirurgie_fonctionnement]

No memos

Répondant : ça fonctionnait parce qu’on ne peut pas fermer la salle d’opération

**P43: IA_05_AS_IT_01.rtf - 43:15 [Répondant : heureusement que n..] (31:31)	(Super)**

Codes:	[Service_chirurgie_fonctionnement]

No memos

Répondant : heureusement que nous avons un appareil électrocardiogramme, le médecin pouvait opérer avec cet appareil mais on n’utilisait plus le stéthoscope

**P43: IA_05_AS_IT_01.rtf - 43:16 [Répondant : ici chez nous il y..] (33:33)	(Super)**

Codes:	[Service_maternité_fonctionnement]

No memos

Répondant : ici chez nous il y avait toujours des accouchements

**P43: IA_05_AS_IT_01.rtf - 43:17 [Répondant : au niveau de notre..] (35:35)	(Super)**

Codes:	[Triage-Isolement-Chlore-thermoflash_Réaction_population]

No memos

Répondant : au niveau de notre structure, dès fois on bricolait avec un triage mais le triage moderne est venu après, ce qui est normé, c’est venu après l’épidémie alors les gens avaient peur parce qu’on avait déjà construit le CTE, les malades ne fréquentaient plus notre structure quand l’épidémie était vive et quand le CTE était vraiment actif parce qu’on referait le cas un cas suspect, on réfère, le fait de référer les cas au CTE, ça jouait négativement sur la fréquentation de la structure alors quand on a mis aussi le triage normé, encore ça aggravait la situation au début alors la population disait la petite maisonnette qu’on est en train de construire, maintenant il parlait de « obasu » le CTE et la population la qualifiait comme la porte qui amène vers le CTE et elle disait que quand n vous entrez dans la porte de la petite maisonnette, on vous amène directement au CTE mais il y avait une mauvaise réputation à cause de nombre des décès donc sur 20 malades qui allaient au CTE, il y avait 15 qui mourraient et cela a marqué négativement la population, ils ne voulaient plus aller au CTE

**P43: IA_05_AS_IT_01.rtf - 43:18 [Répondant : ils se cachaient d..] (37:37)	(Super)**

Codes:	[Structure-Service_Utilisation_Alternatives_population] [Triage-Isolement-Chlore-thermoflash_Réaction_population]

No memos

Répondant : ils se cachaient dans les postes de santé puisque nous avions plusieurs postes de santé privés, ils restaient dans les postes de santé  privés alors c’est à cause de cela que quelques infirmiers des postes de sante se sont faits infectés, il y a 5 postes de santé privés raison pour laquelle ils se sont fait contaminés

**P43: IA_05_AS_IT_01.rtf - 43:19 [Répondant : Non dans notre air..] (39:39)	(Super)**

Codes:	[Début-Progression_Epidémie MVE_Agents affectés]

No memos

Répondant : Non dans notre aire de santé non mais dans l’autre aire de santé de Bingo, oui, mais ici chez nous non

**P43: IA_05_AS_IT_01.rtf - 43:20 [Répondant : parce qu’ils disai..] (41:41)	(Super)**

Codes:	[Triage_Considération_population]

No memos

Répondant : parce qu’ils disaient si tu arrives à la structure, là il collabore avec le CTE donc moindre signe, il vous amène au CTE donc justement les malades avaient fui parce que cela avait une influence négative sur la structure. Mais peu après grâce aux sensibilisations, la population a compris et grâce aussi aux témoignages des vainqueurs qui revenaient du CTE disaient que ce n’est pas un mouroir donc on peut guérir et avec les différentes sensibilisations, a poussé la population d’aller au CTE puisque nombreux se cachaient dans des maisons et ils allaient au CTE à la dernière minute et on avait du mal à les récupérer mais grâce aux différentes sensibilisations ; les relais et nous aussi les IT, on descendait dans la communauté pour faire la sensibilisation et on disait que si vous allez tôt au CTE, vous guérissez. C’est grâce aux sensibilisations et à ceux qui guérissent, cela a fait que la population puisse accepter d’aller au CTE

**P43: IA_05_AS_IT_01.rtf - 43:21 [Répondant : par rapport à l’ac..] (43:43)	(Super)**

Codes:	[Qualité_Soins_Patients]

No memos

Répondant : par rapport à l’accueil, il n’y avait pas des problèmes. Eux-mêmes nous les disent qu’ici nous sommes bien accueilli mais ce qui nous fait peur de venir à la structure et que vous nous referez au CTE. Et les quelques malades qui avaient le courage de venir, nous prenons notre temps pour les sensibiliser que ce n’est pas tous les malades que nous envoyons au CTE ; nous avons des maladies de routine comme le paludisme, le typhoïde, la salmonellose, nous on soigne si c’est la grippe, on soigne mais seulement s’il y a des signes alarment là on peut collaborer avec le CTE pour dire vraiment, on peut aller vous observer au CTE, c’est aussi un hôpital comme ici et ce n’est pas tout le malade qu’on envoie là-bas. Et les quelques malades qui avaient le courage de venir ici et	après le traitement ils retournaient à la maison pour dire aux autres que ce n’est pas tout le monde qu’on envoie au CTE

**P43: IA_05_AS_IT_01.rtf - 43:22 [Répondant : c’est après 48 heu..] (45:45)	(Super)**

Codes:	[Organisation_travail-Circuit des malades_pendant MVE]

No memos

Répondant : c’est après 48 heures, on fait le premier prélèvement et quand le premier prélèvement est négatif et on fait encore le prélèvement un jour après est si le résultat sort toujours négatif, on les ramenait à la structure pour les suivis

**P43: IA_05_AS_IT_01.rtf - 43:23 [Répondant : au niveau du CTE, ..] (47:47)	(Super)**

Codes:	[Organisation_travail-Circuit des malades_pendant MVE]

No memos

Répondant : au niveau du CTE, on le soignait là-bas parce qu’ils avaient aussi d’autres molécule hormis les molécules spécifiques contre Ebola

**P43: IA_05_AS_IT_01.rtf - 43:24 [Répondant : Oui, nous avons co..] (49:49)	(Super)**

Codes:	[Ressources_humaines_Disponibilité et perturbations]

No memos

Répondant : Oui, nous avons connu ça mais ce que nous avons pris comme système parce que ceux-là qui travaillaient à la riposte ils touchaient plus que ceux qui étaient restés à la structure alors nous avions pris comme option, ceux-là qui allaient à la riposte, on prélevait 25% donc ils remettaient 25% à la structure pour encourager ceux qui restaient et le fait que l’équipe de la riposte restait toujours dans le milieu, Mabalako et autres donc on avait organisé la structure donc nous n’avions pas eu de problème parce qu’ils allaient pour la surveillance et ils revenaient pour les gardes nuit donc ceux qui étaient là dans la riposte faisaient des gardes nuit et en plus de cela ils remettaient à la structure 25% de leur revenu alors. En tout cas nous n’avions pas eu des soucis comme tel et nous n’avions pas senti le poids parce qu’ils montaient les gardes nuit et ceux qui n’étaient pas à la riposte venaient pendant la journée et ceux de la riposte faisaient des gardes nuit.

**P43: IA_05_AS_IT_01.rtf - 43:25 [Répondant : je ne sais pas par..] (51:51)	(Super)**

Codes:	[Ressources_humaines_Disponibilité et perturbations]

No memos

Répondant : je ne sais pas parce que ceux qui étaient dans la riposte nous amènent quelque pourcentage et en plus de cela du fait que nous étions soutenu par la gratuité des soins par le gouvernement donc on ne recevait plus un salaire qui étaient comme avant par exemple avant la riposte, on recevait 50$ et grâce au financement de la gratuité des soins, les malades venaient nombreux parce que les soins étaient gratuits ça nous a permis de toucher plus dans la structure presque comme ceux qui étaient dans la riposte et avec le 25% ça renforçait ce qu’on recevait dans la structure donc on n’a pas senti que les gens sont restés dans la structure avec contre cœur ou malgré eux, ça vraiment on n’a pas senti cela parce que nous avons joué le management qui nous a permis peut-être de gérer les conflits de façon interne, en tout cas nous n’avons pas senti ça

**P43: IA_05_AS_IT_01.rtf - 43:26 [Répondant : celui qui touchait..] (53:53)	(Super)**

Codes:	[Gratuité_Conséquences_motivation_prestataire] [Ressources humaines_Motivation_personnel]

No memos

Répondant : celui qui touchait 50 et avec le financement de la gratuité pouvait toucher 100 donc il touchait double

**P43: IA_05_AS_IT_01.rtf - 43:27 [Répondant : la gratuité a comm..] (55:55)	(Super)**

Codes:	[Gratuité_Début]

No memos

Répondant : la gratuité a commencé au mois de décembre

**P43: IA_05_AS_IT_01.rtf - 43:28 [Répondant : recette locale, le..] (57:57)	(Super)**

Codes:	[Recouvrement_coûts-des-soins_avant gratuité]

No memos

Répondant : recette locale, les malades paient

**P43: IA_05_AS_IT_01.rtf - 43:29 [Répondant : ça c’était au nive..] (59:59)	(Super)**

Codes:	[Recouvrement_coûts-des-soins_avant gratuité]

No memos

Répondant : ça c’était au niveau du PCA, seulement PCA

**P43: IA_05_AS_IT_01.rtf - 43:30 [Répondant : ils sont ensemble,..] (61:61)	(Super)**

Codes:	[Recouvrement_coûts-des-soins_avant gratuité]

No memos

Répondant : ils sont ensemble, il n’y pas de différence sauf les activités parce que au niveau du PMA quand il y a des activités de prévention, vaccination, CPN. On intitule ça comme PMA et nous organisons des consultations infirmières et médicales chaque jour, là c’est séparé

**P43: IA_05_AS_IT_01.rtf - 43:31 [Répondant : oui,] (63:63)	(Super)**

Codes:	[Recouvrement_coûts-des-soins_avant gratuité]

No memos

Répondant : oui,

**P43: IA_05_AS_IT_01.rtf - 43:32 [épondant : oui, avant] (65:65)	(Super)**

Codes:	[Recouvrement_coûts-des-soins_avant gratuité]

No memos

épondant : oui, avant

**P43: IA_05_AS_IT_01.rtf - 43:33 [Répondant : c’était gratuit, l..] (67:67)	(Super)**

Codes:	[Recouvrement_coûts-des-soins_avant gratuité]

No memos

Répondant : c’était gratuit, la consultation infirmière était gratuite

**P43: IA_05_AS_IT_01.rtf - 43:34 [Répondant : la consultation mé..] (69:69)	(Super)**

Codes:	[Recouvrement_coûts-des-soins_avant gratuité]

No memos

Répondant : la consultation médicale en soi était 25 maintenant avec les médicaments

**P43: IA_05_AS_IT_01.rtf - 43:35 [Répondant : ça dépendait de la..] (71:71)	(Super)**

Codes:	[Recouvrement_coûts-des-soins_avant gratuité]

No memos

Répondant : ça dépendait de la pathologie de quelqu’un et des médicaments qu’il va recevoir

**P43: IA_05_AS_IT_01.rtf - 43:36 [Répondant : d’abord le PDSS, i..] (73:73)	(Super)**

Codes:	[Recouvrement_coûts-des-soins_avant gratuité]

No memos

Répondant : d’abord le PDSS, il achetait 3,1 et quand le malade vient, il reçoit les soins gratuits et le nombre des malades qui se faisaient consulter maintenant le PDSS vient pour payer leurs factures

**P43: IA_05_AS_IT_01.rtf - 43:37 [Répondant : Oui, avant qu’Ebol..] (75:75)	(Super)**

Codes:	[Recouvrement_coûts-des-soins_avant gratuité]

No memos

Répondant : Oui, avant qu’Ebola n’arrive

**P43: IA_05_AS_IT_01.rtf - 43:38 [Répondant : le système a conti..] (77:77)	(Super)**

Codes:	[Recouvrement_coûts-des-soins_avant gratuité]

No memos

Répondant : le système a continué comme d’habitude et puis on a encore ajouté le PCA, les cas hospitalisés

**P43: IA_05_AS_IT_01.rtf - 43:39 [Répondant : dans notre structu..] (79:79)	(Super)**

Codes:	[Recouvrement_coûts-des-soins_avant gratuité]

No memos

Répondant : dans notre structure parce que nous sommes comme un centre mais dans le niveau des hôpitaux, il payait des actes. Nous sommes un centre e santé et on prend seulement les actes du centre

**P43: IA_05_AS_IT_01.rtf - 43:40 [Répondant : Oui, malheureuseme..] (81:81)	(Super)**

Codes:	[Recouvrement_coûts-des-soins_avant gratuité]

No memos

Répondant : Oui, malheureusement on essayait d’expliquer, les malades ne sentaient pas bien.

**P43: IA_05_AS_IT_01.rtf - 43:41 [Répondant : les malades ambula..] (83:83)	(Super)**

Codes:	[Gratuité_Début]

No memos

Répondant : les malades ambulatoires, les hospitalisés et peu après ils ont changé, ils ont pris seulement le cas de la médecine interne. C’est le 30 juin de l’année passée

**P43: IA_05_AS_IT_01.rtf - 43:42 [Répondant : on a pris seulemen..] (85:85)	(Super)**

Codes:	[Gratuité_Structures-Services_concernés]

No memos

Répondant : on a pris seulement les cas hospitalisés, avant le 30 juin 2019 même les accouchements étaient pris en compte. Pour les accouchements on pouvait payer seulement 20$ et les malades 8$ et PDSS paie le 18$ donc il y avait une somme que les malades payaient

**P43: IA_05_AS_IT_01.rtf - 43:43 [épondant : il y a PDSS et EIP ..] (87:87)	(Super)**

Codes:	[Gratuité_Evolution]

No memos

épondant : il y a PDSS et EIP face mais la gratuité là c’est seulement les cas d’hospitalisation. Cette gratuité dans laquelle nous sommes a commencé à partir du 15 février 2020

**P43: IA_05_AS_IT_01.rtf - 43:44 [Répondant : Nous, on ne peut p..] (91:91)	(Super)**

Codes:	[Gratuité_Evolution]

No memos

Répondant : Nous, on ne peut pas refuser le contrat, on a essayé de persuader la population mais la population n’a pas accepté mais en un moment donné nous avons réclamé, on a dit ceux qui n’amènent pas leur carte d’électeurs, il faut demander n’importe quelle carte même la carte de mutuelle, vous pouvez même prendre le numéro du voisin mais les explications-là sont venues un peu après

**P43: IA_05_AS_IT_01.rtf - 43:45 [Répondant : chez nous, non sau..] (93:93)	(Super)**

Codes:	[Gratuité_Evolution]

No memos

Répondant : chez nous, non sauf seulement la photocopie de la carte mais on a dit si vous refusez de donner la photocopie de n’importe quelle carte, il faut payer et quand il sait qu’il n’a pas d’argent pour payer, il est obligé de photocopier la carte mais au mois de mars où il y a eu beaucoup des massacres, c’est là où ils ont présenté beaucoup des résistances et nous avions reçu peu des photocopies

**P43: IA_05_AS_IT_01.rtf - 43:46 [Répondant : vu que nous traver..] (95:95)	(Super)**

Codes:	[Gratuité_Rumeurs]

No memos

Répondant : vu que nous traversons la situation d’insécurité donc la population est devenue un peu méfiante. Ces gens n’ont pas pu nous égorger et maintenant ils nous ont amené les médicaments

**P43: IA_05_AS_IT_01.rtf - 43:47 [Répondant : quand on a demandé..] (97:97)	(Super)**

Codes:	[Gratuité_Evolution]

No memos

Répondant : quand on a demandé, on nous a dit que le budget ne permettait plus de couvrir tous les actes du fait qu’on ne pensait pas que l’épidémie devait faire deux ans comme ça. Leur budget a bougé raison pour laquelle ils essayé de limiter quelques prestations

**P43: IA_05_AS_IT_01.rtf - 43:48 [Répondant : dans le PDSS, il y..] (99:99)	(Super)**

Codes:	[Médicaments_disponibilité_fosa]

No memos

Répondant : dans le PDSS, il y avait un pourcentage pour les médicaments, il y avait 50% pour le personnel, 30% pour les médicaments et 20% pour le fonctionnement et grâce à cette répartition, ça nous aide de nous procurer les médicaments

**P43: IA_05_AS_IT_01.rtf - 43:49 [Répondant : nous avions connu ..] (101:101)	(Super)**

Codes:	[Médicaments_disponibilité_fosa]

No memos

Répondant : nous avions connu des problèmes quand le gouvernement ou EIP face ont pris le système de retirer à la source donc 20% des médicaments. Mais avant il nous donnait et quand nous donnons à la zone, il nous envoie suffisamment des médicaments, on allait acheter ailleurs qu’à la zone de santé lais dès que PDSS a pris ça d’une façon à la source et o la zone on n’a pas tous les médicaments pour les 20% qu’ils ont eu alors ça jouait sur notre système d’approvision

**P43: IA_05_AS_IT_01.rtf - 43:50 [Répondant : c’était ça l’objec..] (103:103)	(Super)**

Codes:	[Médicaments_disponibilité_fosa]

No memos

Répondant : c’était ça l’objectif mais il ne nous livrait pas les médicaments comme il le faut

**P43: IA_05_AS_IT_01.rtf - 43:51 [Répondant : on a travaillé nor..] (105:105)	(Super)**

Codes:	[SNIS_fonctionnement_Decrire_pdt MVE]

No memos

Répondant : on a travaillé normalement parce que chaque mois, on rapportait les données et on faisait l’analyse. En tout cas dans notre structure, on n’a pas tripoté les données, on n’a pas connu des problèmes de rapportage des données

**P43: IA_05_AS_IT_01.rtf - 43:52 [Répondant : puisque nos regist..] (107:107)	(Super)**

Codes:	[SNIS_fonctionnement_Decrire_pdt MVE]

No memos

Répondant : puisque nos registres sont toujours au complet, on n’a pas eu des soucis sauf au début de la riposte, on nous a tous amené à l’observation pendant 21 jours et cela a joué à nos données mais le peu des malades qui étaient là, on arrivait à les enregistrer toujours

**P43: IA_05_AS_IT_01.rtf - 43:53 [Répondant : nous sommes 75 et ..] (109:109)	(Super)**

Codes:	[SNIS_fonctionnement_Decrire_pdt MVE]

No memos

Répondant : nous sommes 75 et 8 seulement étaient restés. Tous les autres sont partis en quarantaine pendant 21 jours et nous étions remplacés par les gens qui sont venus de Mabalako et les autres de Beni et après 21 jours nous sommes revenus à l’hôpital

**P43: IA_05_AS_IT_01.rtf - 43:54 [Répondant : avec les codesa et..] (111:111)	(Super)**

Codes:	[Réunion_pdt-épidémie-Ebola]

No memos

Répondant : avec les codesa et les relais, oui, oui c’est une fois par mois.

**P43: IA_05_AS_IT_01.rtf - 43:55 [Répondant : nous avons connu d..] (113:113)	(Super)**

Codes:	[Réunion_pdt-épidémie-Ebola]

No memos

Répondant : nous avons connu des perturbations, c’était juste au début de l’année 2019 parce qu’on avait limité les contacts, c’était pendant trois mois et après on a recommencé et pour renforcer la sensibilisation nous on se réunissait avec les présidents des différentes CAC parce qu’avant on réunissait tous les relais alors vue l’épidémie, nous nous sommes dit on limite le nombre, on va se réunir seulement avec les présidents des différentes CAC

**P43: IA_05_AS_IT_01.rtf - 43:56 [Répondant : En tout cas c’étai..] (115:115)	(Super)**

Codes:	[Répondant_Responsabilité_pdt MVE]

No memos

Répondant : En tout cas c’était dans la surveillance mais je n’étais pas à 100% dans la surveillance. Je faisais ce qu’on dit de la surveillance interne au niveau de la structure mais en collaboration avec les relais qui faisaient le contrôle et les rapports de ce qui trouvaient dans la communauté mais notre ITA, c’est lui qui était à 100% dans la riposte comme tel

**P43: IA_05_AS_IT_01.rtf - 43:57 [Répondant : peut-être dans ce ..] (117:117)	(Super)**

Codes:	[Répondant_Responsabilité_pdt MVE]

No memos

Répondant : peut-être dans ce sens-là qu’il y avait plus de travail puisque moi je collaborais plus avec mon ITA, moi je n’ai pas senti le poids parce que mon ITA est un infirmier actif alors maintenant le fait qu’il est actif, on collaborait très bien avec lui et je n’ai pas senti le poids du travail parce que l’ITA est un monsieur qui est vraiment très formidable

**P43: IA_05_AS_IT_01.rtf - 43:58 [Répondant : dans notre structu..] (119:119)	(Super)**

Codes:	[PCI_Situation]

No memos

Répondant : dans notre structure, nous avons une ONG qui est là spécifiquement pour la PCI

**P43: IA_05_AS_IT_01.rtf - 43:59 [Répondant : si RSCI partait d’..] (121:121)	(Super)**

Codes:	[PCI_Comment_maintenir]

No memos

Répondant : si RSCI partait d’abord nous avons reçu les transformations avoir les différentes formations, il y a le comité d’hygiène, commission PCI qui sont déjà intégré dans la structure alors grâce aux différentes réunions que nous tenons régulièrement avec le comité et le comité PCI qui se tient une fois par semaine, ces différentes réunions nous aident à pérenniser les différentes activités

**P43: IA_05_AS_IT_01.rtf - 43:60 [Répondant : en tout cas, nous ..] (123:123)	(Super)**

Codes:	[PCI_Comment_maintenir]

No memos

Répondant : en tout cas, nous espérons vraiment. C’est ça notre souhait

**P43: IA_05_AS_IT_01.rtf - 43:61 [Répondant : c’est qui va nous ..] (125:125)	(Super)**

Codes:	[PCI_Comment_maintenir]

No memos

Répondant : c’est qui va nous aider, c’est de pérenniser ces réunions hebdomadaires parce que les chefs des services sont membres de ce comité

**P43: IA_05_AS_IT_01.rtf - 43:62 [Répondant : si je peux dire so..] (127:127)	(Super)**

Codes:	[Changements_depuis_Ebola]

No memos

Répondant : si je peux dire socialement ce qui est positif quoi, c’est la prise de conscience, la sensibilisation renforcée et les mesures de prévention et de contrôle et dans la communauté, socialement le lavage des mains, c’est devenu un réflexe. Mais d’une façon négative, Ebola est venue comme une séparation, les visites qu’on réalisait sans réfléchir, spontanément comme ça donc on se posait des questions si je vais chez un tel peut-être il a déjà attrapé son Ebola donc ça coupait toutes les relations, nous entant qu’africain

**P43: IA_05_AS_IT_01.rtf - 43:63 [Répondant : d’abord nous allon..] (129:130)	(Super)**

Codes:	[Souhait_Changement_gestion_futures_épidémies]

No memos

Répondant : d’abord nous allons continuer avec les mesures des préventions, des mesures barrières, le lavage des mains à temps et à contre temps, essayer d’éviter les contacts. Ces mesures-ci nous ont aidés pour éviter cette épidémie d’Ebola, il faut continuer à appliquer les mesures barrières

Seulement, nous n’avons pas des matériels comme c’est une maladie grave qui demande des appareils de réanimation nous n’en avons pas ; si ça arrivait ici donc il faut assurer une bonne prise en charge donc il faut des appareils pour assurer la réanimation des cas suspects et même les masques, nous n’en avons pas suffisamment

**P43: IA_05_AS_IT_01.rtf - 43:64 [Répondant : au niveau social d..] (132:133)	(Super)**

Codes:	[Commentaire-Recommandation_Répondant]

No memos

Répondant : au niveau social donc au niveau sanitaire, je n’ai pas à ajouter ou beaucoup des commentaires à faire. Moi seulement je remercie le gouvernement et l’équipe de la riposte pour tout ce qu’ils ont fait seulement au niveau social, ce que la population demande d’aménager la route là où ils passaient avec leurs véhicules puisqu’ils ont abimé cette route avec leurs véhicules mais l’équipe de la riposte est partie sans aménager cette route qu’ils ont abimé avec leurs véhicules

On sait peut-être que cela n’était pas dans leur programme mais ils devaient entretenir cette route pour permettre à la population de bien faire leurs activités comme avant

**P43: IA_05_AS_IT_01.rtf - 43:65 [Répondant : pas tellement, ils..] (135:135)	(Super)**

Codes:	[Commentaire-Recommandation_Répondant]

No memos

Répondant : pas tellement, ils ont construit une petite pièce comme chambre de contrôle des infirmiers, il y a seulement FBIN qui a réhabilité la maternité, mettre les tôles et la peinture mais le bâtiment est resté le même

**P43: IA_05_AS_IT_01.rtf - 43:66 [Répondant : oui,] (137:137)	(Super)**

Codes:	[Commentaire-Recommandation_Répondant]

No memos

Répondant : oui,

**P43: IA_05_AS_IT_01.rtf - 43:67 [Répondant : quand l’épidémie é..] (139:139)	(Super)**

Codes:	[Structure-Service_Utilisation_population]

No memos

Répondant : quand l’épidémie était vive et comme les gens avaient peur d’aller au CTE, le taux d’utilisation avait baissé mais grâce à la sensibilisation, et quand ils ont compris ça le taux d’utilisation avait augmenté

**P44: IA_05_BCZS_AG.rtf - 44:1 [Répondant : au fait par rappor..] (2:2)	(Super)**

Codes:	[Début-Progression_Epidémie MVE]

No memos

Répondant : au fait par rapport à l’épidémie à la PVE dans la zone de santé Mabalako, i y avait un bon moment au mois de juin 2018 où nous avions constaté certains cas des décès dans l’aire de santé  Mangina. Au cours de cette période, alors nous avions diligenté une descente sur terrain pour aller faire le constat parce que les cas des décès qui se suivent, il y avait les mêmes signes, c’est pourquoi on avait effectué une descente sur terrain, après le constat, on avait directement fait le rapport à Goma et Goma a envoyé un médecin pour nous aider à venir faire le prélèvement de l’échantillon et quand on a eu l’échantillon, on a envoyé à Kinshasa et c’est en date du 8 juillet qu’on a déclaré que le cas est sorti positif. C’est en ce moment que le ministère national de la santé a envoyé une équipe venant de Kinshasa

**P44: IA_05_BCZS_AG.rtf - 44:2 [Répondant : en fait, nous somm..] (4:4)	(Super)**

Codes:	[Considération_Ebola_population]

No memos

Répondant : en fait, nous sommes dans la zone de santé Mabalako, Ebola nous avons entendu que c’était à Bikoro dans la province de l’Equateur donc la population était au courant qu’Ebola existe mais il n’y avait personne qui était au courant de ce phénomène, c’est pourquoi quand on avait vu les gens mourir à Mangina la population était vraiment frustrés. D’ailleurs il y avait un déplacement massif à cause de cette maladie et dès lors qu’on a vu les autorités, le gouverneur, le ministre national sont venus avec les partenaires, ils étaient calme et comme les autorités étaient venus nous apporter l’appui, en ce moment-là ils étaient soulagés par la présence des autorités et puis la population a accueilli les autorités et les partenaires, ils ont travaillé dans une franche collaboration et il n’y avait pas vraiment des résistances

**P44: IA_05_BCZS_AG.rtf - 44:3 [Répondant : ici en tout cas on..] (6:6)	(Super)**

Codes:	[Agression-Menaces_Structure-Prestataire_raisons]

No memos

Répondant : ici en tout cas on n’a pas connu des attaques et surtout à Mangina jusqu’aujourd’hui on n’a pas enregistré des résistances

**P44: IA_05_BCZS_AG.rtf - 44:4 [Répondant : bon ça a été toujo..] (8:8)	(Super)**

Codes:	[Triage_Considération_population]

No memos

Répondant : bon ça a été toujours bien accueilli mais là où ça posait des soucis, c’est seulement à Loya du fait qu’en ce moment-là il y avait un cas d’un décès qui était guéris et après il a été encore contaminé et cela a causé des résistances mais aussi une autre résistance, c’est par rapport à la payer de la prime aux prestataires donc le retard qu’on avait connu en ce qui concerna la prime des prestataires, cela a aussi créé des résistances jusqu’à ce qu’il y avait certaines installations qui ont été endommagées par la population.

**P44: IA_05_BCZS_AG.rtf - 44:5 [Répondant : nous étions là com..] (10:10)	(Super)**

Codes:	[Répondant_Responsabilité_pdt MVE]

No memos

Répondant : nous étions là comme un intermédiaire entre les partenaires et les prestataires. Nous comme zone de santé parce que nous sommes toujours avec la population et puis cette population a confiance en nous, nous on faisait que sensibiliser la population de s’approprier de cette activité de la riposte.

**P44: IA_05_BCZS_AG.rtf - 44:6 [Répondant : oui, bien sûr il y..] (12:12)	(Super)**

Codes:	[Structures-Services_fonctionnement]

No memos

Répondant : oui, bien sûr il y avait certaines structures qui n’avaient pas bien fonctionné à cause des flambées de cas parce que le problème est que la population disait que par exemple quand un malade arrive à l’hôpital, quand on constate qu’il y a un signe, la température autour de 39°, on l’amène directement au CTE et comme la population avait crainte du CTE directement on avait constaté une baisse de fréquentation dans les structures à cause de ce circuit. Dès qu’on constate qu’on a 39°, on t’amène au CTE

**P44: IA_05_BCZS_AG.rtf - 44:7 [Répondant : ils craignaient le..] (14:14)	(Super)**

Codes:	[Considération_CTE_population]

No memos

Répondant : ils craignaient le CTE parce que quand on dit que tu as Ebola donc là c’est la vie ou la mort et il y a certains que disaient quand on arrive au CTE, il y a moins de chance que vous puissiez survivre. Mais nous avons efforcé de les faire comprendre qu’au CTE si vous arrivez vite, il y a lieu que vous puissiez recouvrir votre santé

**P44: IA_05_BCZS_AG.rtf - 44:8 [Répondant : il y a certains qu..] (16:16)	(Super)**

Codes:	[Structure-Service_Utilisation_Alternatives_population]

No memos

Répondant : il y a certains qui fuyaient dans la foret, ils allaient mourir dans la foret

**P44: IA_05_BCZS_AG.rtf - 44:9 [Répondant : le laboratoire dan..] (18:18)	(Super)**

Codes:	[Structure-Service_Fermeture_pdt MVE]

No memos

Répondant : le laboratoire dans les centres de santé, on avait interdit, on avait dit qu’on interdit de faire les examens de labo dans les centres de santé mais aussi au CTE, c’était difficile de faire des interventions, il y a eu une dame qui avait Ebola et on l’avait enterré avec sa grossesse parce qu’on en savait pas faire des interventions chirurgicales en ce moment de crise de MVE et cela a posé des polémiques avec la coutume et à un moment les coutumiers demandaient qu’on paie quelque chose

**P44: IA_05_BCZS_AG.rtf - 44:10 [Répondant : en fait on a fait ..] (20:20)	(Super)**

Codes:	[Structure-Service_Fermeture_pdt MVE]

No memos

Répondant : en fait on a fait ça d’une façon managérial avec l’Unicef, on faisait quelque chose pour que la communauté puisse accepter qu’on enterre comme ça

**P44: IA_05_BCZS_AG.rtf - 44:11 [Répondant : par rapport à la v..] (22:22)	(Super)**

Codes:	[Service_vaccination_Fonctionnement]

No memos

Répondant : par rapport à la vaccination de routine, il n’y a pas eu des problèmes parce qu’on avait recruté le personnel du milieu qui faisait des vaccinations de routine dans la zone et ces personnes sont acceptés dans la zone et ça ne posait pas beaucoup des problèmes

**P44: IA_05_BCZS_AG.rtf - 44:12 [Répondant : ici on n’avait pas..] (24:24)	(Super)**

Codes:	[Service_vaccination_Fonctionnement]

No memos

Répondant : ici on n’avait pas vraiment senti ça ici et pendant l’épidémie on avait organisé une campagne de VAR et la zone de santé avait eu 107% donc la zone de santé n’avait pas senti une grande résistance par rapport à ça, il pouvait avoir des cas bénins mais on n’avait pas senti

**P44: IA_05_BCZS_AG.rtf - 44:13 [Répondant : oui, par exemple d..] (26:26)	(Super)**

Codes:	[Service_vaccination_Fonctionnement]

No memos

Répondant : oui, par exemple dans la zone voisine ici à Mandima, je ne pense pas que pendant la campagne, ils ont atteint même 50%

**P44: IA_05_BCZS_AG.rtf - 44:14 [Répondant : la raisons est qu’..] (28:28)	(Super)**

Codes:	[Service_vaccination_Fonctionnement]

No memos

Répondant : la raisons est qu’il n’avait même pas accepté Ebola, vous avez même entendu qu’on avait tué des gens, l’affrontement entre la population et l’équipe d’Ebola donc là c’était terrible, ils n’ont pas accepté la riposte

**P44: IA_05_BCZS_AG.rtf - 44:15 [Répondant : c’est une hypothès..] (30:30)	(Super)**

Codes:	[Ressources humaines_Implication-agents-locaux] [Service_vaccination_Fonctionnement]

No memos

Répondant : c’est une hypothèse, je pense que le problème peut être par rapport au recrutement des agents de la riposte quand on amène des étrangers qui viennent travailler chez eux alors qu’il y a des enfants qui peuvent nous aider par rapport à ça. Je pense que c’est parmi les hypothèses qu’ils évoquaient

**P44: IA_05_BCZS_AG.rtf - 44:16 [Répondant : oui, les ressource..] (32:32)	(Super)**

Codes:	[Ressources humaines_Implication-agents-locaux] [Service_vaccination_Fonctionnement]

No memos

Répondant : oui, les ressources locales mais telles que je vous ai dit que la maladie a commencé ici même ici il y avait beaucoup des gens qui sont venus d’ailleurs avec cette crainte comme ils voulaient que la maladie soit éradiquée, ils ont accepté de travailler même avec les étrangers

**P44: IA_05_BCZS_AG.rtf - 44:17 [Répondant : vous savez quand l..] (34:34)	(Super)**

Codes:	[Ressources_humaines_Disponibilité et perturbations]

No memos

Répondant : vous savez quand l’épidémie a commencé directement le gouvernement nous a aligné par rapport au projet PDSS, la gratuité des soins. Alors avec ce projet de gratuité on avait vraiment constaté un engouement des malades, bien sûr c’était dans le cadre d’aider la population qui était victime d’Ebola qui ne faisait plus les champs parce qu’ils enterraient chaque jour les gens qui mourraient. Pendant cette époque, on avait recruté les gens, les infirmiers sont partis traités au CTE, les autres dans la vaccination et dans différentes commissions, alors dans la structure on avait vraiment constaté un gap significatif et cela avait influencé la qualité de la prise en charge des malades. On a évolué par rapport à ça mais à un moment donné, la DPS nous avait recommandé de signes des protocoles avec certaines personnes en attendant la fin de l’épidémie alors on devait signer des protocoles avec certain personnel pour appuyer les équipes des structures mais pendant cette période on avait constaté une baisse de la qualité des prestations.

**P44: IA_05_BCZS_AG.rtf - 44:18 [Répondant : l’accueil des mala..] (36:36)	(Super)**

Codes:	[Qualité_soins_Prestataires]

No memos

Répondant : l’accueil des malades, par exemple il y a 50 malades qui vous attendent, l’accueil ne sera pas vraiment de bonne qualité et le nursing le prélèvement des signes vitaux ne seront pas des bonnes qualités

**P44: IA_05_BCZS_AG.rtf - 44:19 [Répondant : oui, c’était le ca..] (38:38)	(Super)**

Codes:	[Ressources_humaines_situation_grève]

No memos

Répondant : oui, c’était le cas et d’ailleurs c’est la raison pour laquelle on n’avait pas vite découvert la maladie parce que l’épidémie est venue quand on était en grève et l’IT lui-même n’envoyait pas le rapport et nous on pensait que tout va bien mais quand on a constaté que ls gens sont en train de mourir et la société civile nous a appelé qu’ici ça ne va pas et nous sommes descendu sur terrain et c’est en ce moment que nous avions constaté parce qu’en ce moment il n’y avait plus de rapport

**P44: IA_05_BCZS_AG.rtf - 44:20 [Répondant : je pense que c’éta..] (40:40)	(Super)**

Codes:	[Ressources_humaines_situation_grève]

No memos

Répondant : je pense que c’était au mois d’avril 2018

**P44: IA_05_BCZS_AG.rtf - 44:21 [Répondant : bon en fait là je ..] (42:42)	(Super)**

Codes:	[Ressources humaines_Motivation_personnel]

No memos

Répondant : bon en fait là je ne sais pas comment expliquer, ce que je sais d’abord c’est nous qui gérons les structures et s’il s’agit des recrutements c’est la zone de santé qui faisait ça, on peut prendre autant ici et autant là-bas qui fallait nous aider par rapport à la riposte mais il y a d’autres sans l’autorisation de la zone de santé, ils sont partis d’eux-mêmes parce que quand ils voyaient les offres d’emploi, ils ont postulé, ils ont reçu et sont partis

**P44: IA_05_BCZS_AG.rtf - 44:22 [Répondant : par rapport à la g..] (44:44)	(Super)**

Codes:	[Ressources humaines_Implication-agents-locaux]

No memos

Répondant : par rapport à la gestion de la riposte, nous étions impliqués et moi qui vous parler, j’étais le président de la commission logistique, depuis le début de la riposte, j’étais là-bas et dans la zone de santé donc on avait fait un chronogramme pour que toutes les activités se déroulent normalement et aussi dans la riposte que dans la zone de santé

**P44: IA_05_BCZS_AG.rtf - 44:24 [Répondant : avant l’épidémie l..] (50:50)	(Super)**

Codes:	[Recouvrement_coûts-des-soins_avant gratuité]

No memos

Répondant : avant l’épidémie le mode de paiement, c’était le paiement sur l’acte donc on était toujours dans le projet PDSS mais c’est un projet de la Banque Mondiale via PDSS où on avait mis en place un tarif après négociation avec la population, par exemple si la consultation est de 3,5 et on pouvait négocier avec la population dans ce sens que la population vient payer 1,5 et la banque mondiale vient payer la différence. C’est comme ça qu’on travaillait. Quand la population paie sa contribution, après trois mois la banque mondiale vient payer la différence ?

**P44: IA_05_BCZS_AG.rtf - 44:25 [Répondant : En tout cas on n’a..] (48:48)	(Super)**

Codes:	[Ressources_humaines_solution-local_Disponibilité et perturbations]

No memos

Répondant : En tout cas on n’avait pas trouvé facilement parce que tout le monde était lancé dans la riposte. Je veux donner un exemple, ici à l’hôpital général, il y avait un médecin qui est parti travaillé d’abord avec l’OMS et on avait fait un appel à un médecin à Butembo et jusqu’aujourd’hui il reste dans la riposte, le médecin directeur est dans la riposte donc ça fait presqu’une année et demie depuis qu’il est parti

**P44: IA_05_BCZS_AG.rtf - 44:26 [Répondant : même ici c’était l..] (46:46)	(Super)**

Codes:	[Organisation_travail-Circuit des malades_pendant MVE] [Ressources_humaines_solution-local_Disponibilité et perturbations]

No memos

Répondant : même ici c’était le cas tel que je vous disais, il y avait ceux qui avaient abandonnés carrément comme un infirmier doit rester dans sa structure mais dès lors qu’il est parti, ce qu’il faisait, ça doit souffrir. Maintenant qu’est-ce que nous avions fait ? Nous avions appelés le chef de division et lui nous avait dit de signer les protocoles avec d’autres agents qui peuvent nous aider

**P44: IA_05_BCZS_AG.rtf - 44:27 [Répondant : en octobre 2016] (52:52)	(Super)**

Codes:	[Recouvrement_coûts-des-soins_actuel]

No memos

Répondant : en octobre 2016

**P44: IA_05_BCZS_AG.rtf - 44:28 [Répondant : oui, la zone de sa..] (54:54)	(Super)**

Codes:	[Recouvrement_coûts-des-soins_actuel]

No memos

Répondant : oui, la zone de santé de Kayina, la zone de santé de Bayimbongo dans la partie sud, il y a la zone de santé de Nyiragongo, zone de santé de Rwanguba et la zone de santé de Kirotsha donc c’était six zones de santé. Et la répartition se faisait comme suit le 30% pour l’approvisionnement en médicaments, 50% personnel, 15% fonctionnement et 5% investissement

**P44: IA_05_BCZS_AG.rtf - 44:29 [Répondant : ici il y avait dou..] (56:56)	(Super)**

Codes:	[Recouvrement_coûts-des-soins_actuel]

No memos

Répondant : ici il y avait douze centres de santé, l’hôpital général et le BCZ

**P44: IA_05_BCZS_AG.rtf - 44:30 [Répondant : le projet continua..] (58:58)	(Super)**

Codes:	[Gratuité_Début]

No memos

Répondant : le projet continuait même la période d’Ebola mais on a ajouté d’autres aspects par rapport à la gratuité. C’est la gratuité de soins, c’est ce qui a changé. Le PDSS paie seulement les consultations mais les autres actes n’étaient pas pris en compte

**P44: IA_05_BCZS_AG.rtf - 44:31 [Répondant : je pense que même ..] (60:60)	(Super)**

Codes:	[Gratuité_Structures-Services_concernés]

No memos

Répondant : je pense que même avant l’épidémie, il y avait certains actes que la banque mondiale paie, il y avait la consultation et l’accouchement, la CPN et la CPS et enfant complétement vacciné et le planning familial

**P44: IA_05_BCZS_AG.rtf - 44:32 [Répondant : mais pendant l’épi..] (62:62)	(Super)**

Codes:	[Gratuité_Structures-Services_concernés]

No memos

Répondant : mais pendant l’épidémie pour les centres de santé, il avait payé seulement les consultations, c’était à 3,5 mais par rapport à l’hôpital général, pendant l’épidémie, on payait la médecine interne et la pédiatrie aussi pas des consultations

**P44: IA_05_BCZS_AG.rtf - 44:33 [Répondant : ici non parce qu’o..] (64:64)	(Super)**

Codes:	[Gratuité_Evolution]

No memos

Répondant : ici non parce qu’on était dans les deux projet-là, c’est pourquoi ça n’a pas été la même chose qu’ailleurs

**P44: IA_05_BCZS_AG.rtf - 44:34 [Répondant : oui, en tout cas o..] (66:66)	(Super)**

Codes:	[Gratuité_Structures-Services_concernés]

No memos

Répondant : oui, en tout cas on a fait des plaidoyers et ça n’a pas marché car ils ont dit que nous allons prendre les centres de santé avec l’ancienne configuration, les structures avec l’ancienne configuration. Ailleurs on a pris même le centre hospitalier intégré mais ici ça n’a pas marché

**P44: IA_05_BCZS_AG.rtf - 44:35 [Répondant : non, ils n’ont pas..] (68:68)	(Super)**

Codes:	[Gratuité_Structures-Services_concernés]

No memos

Répondant : non, ils n’ont pas été pris

**P44: IA_05_BCZS_AG.rtf - 44:36 [Répondant : oui, ici vous orga..] (70:70)	(Super)**

Codes:	[Gratuité_Conséquences]

No memos

Répondant : oui, ici vous organisez la gratuité à coté non, ça va jouer directement sur leur fonctionnement

**P44: IA_05_BCZS_AG.rtf - 44:37 [Répondant : en tout cas ça aug..] (72:72)	(Super)**

Codes:	[Gratuité_Conséquences_motivation_prestataire]

No memos

Répondant : en tout cas ça augmentait parce qu’ils étaient avec deux projets pour la banque mondiale et aussi PDSS. En tout cas la motivation a augmenté sensiblement

**P44: IA_05_BCZS_AG.rtf - 44:38 [Répondant : les rumeurs étaien..] (74:74)	(Super)**

Codes:	[Gratuité_Rumeurs]

No memos

Répondant : les rumeurs étaient seulement par rapport à la crainte, quand vous arrivez ou on vous transférez au CTE, c’était ça le problème mais par rapport à la gratuité, il n’y avait pas des problèmes

**P44: IA_05_BCZS_AG.rtf - 44:39 [Répondant : oui, les médicamen..] (76:76)	(Super)**

Codes:	[Médicaments_disponibilité_fosa]

No memos

Répondant : oui, les médicaments sont là mais seulement pour la première livraison ça posait des problèmes parce que la première livraison, on nous avait livré les médicaments sans tenir compte de la base donc il n’avait pas eu le temps de demander nos commandes mais à part la première livraison, on respectait le circuit des médicaments. Et jusqu’aujourd’hui nous avons de stock de cette première livraison qu’on ne sait pas comment consommer

**P44: IA_05_BCZS_AG.rtf - 44:40 [Répondant : il nous approvisio..] (78:78)	(Super)**

Codes:	[Médicaments_disponibilité_fosa]

No memos

Répondant : il nous approvisionnait directement donc le 30% était déposé dans notre compte et puis nous envoyons à la Stramex et la Stramex nous amène les médicaments

**P44: IA_05_BCZS_AG.rtf - 44:41 [Répondant : oui, il y avait de..] (80:80)	(Super)**

Codes:	[SNIS_fonctionnement_Decrire_pdt MVE]

No memos

Répondant : oui, il y avait de problème parce qu’on était frustré au début et quand Ebola a commencé, elle a paralysé le système, on a fait presque plusieurs mois sans tenir les réunions d’analyse parce qu’on était dans la riposte même chose par rapport aux IT, pour le canevas la qualité de la récolté des données n’étaient pas bonne mais à partir de mois de juin 2019, c’est là qu’on a commencé à suivre le circuit normal du SNIS

**P44: IA_05_BCZS_AG.rtf - 44:42 [Répondant : ça souffert parce ..] (82:82)	(Super)**

Codes:	[Réunion_pdt-épidémie-Ebola]

No memos

Répondant : ça souffert parce qu’on était moins de 80% quand on faisait la réévaluation de la tenue des réunions de supervisions, vraiment on était en dessous de 80%

**P44: IA_05_BCZS_AG.rtf - 44:43 [Répondant : d’abord par rappor..] (84:84)	(Super)**

Codes:	[PCI_Comment_maintenir]

No memos

Répondant : d’abord par rapport à ça quand on faisait le recrutement du personnel pendant la PCI, on prenait les points focaux dans les centres de santé, ils étaient formés par rapport à la PCI et puis même au BCZ nous avons un superviseur qui est le point focal de la PCI et avec ça nous pensons que nous avons quand même les ressources pour pérenniser la PCI dans la zone de santé mais ce qui peut poser des problèmes c’est les intrants PCI mais avec le moyen de bord on peut voir comment pérenniser la PCI dans les structures sanitaires

**P44: IA_05_BCZS_AG.rtf - 44:44 [Répondant : oui, c’est ici à M..] (86:86)	(Super)**

Codes:	[Fosa-Institution_Présentation]

No memos

Répondant : oui, c’est ici à Mabalako que l’épidémie a commencé

**P44: IA_05_BCZS_AG.rtf - 44:45 [Répondant : le point positif e..] (88:88)	(Super)**

Codes:	[Souhait_Changement_gestion_futures_épidémies]

No memos

Répondant : le point positif est que nous avions constaté un appui du niveau supérieur mais aussi des partenaires. Mais ce qu’il faut améliorer donc on avait accordé le pouvoir aux partenaires de nous gérer nous les agents de l’Etat donc c’est comme si ce sont eux qui avaient beaucoup de pouvoir sur la gestion de la riposte que nous les agents de l’Etat. Par rapport à nous la logistique, si nous on n’avait pas de prérequis par rapport à la logistique, on ne pouvait pas voir la présence du gouvernement dans la riposte. Tout était fait par les partenaires Qu’il s’agissait de la gestion des matériels roulant, en tout cas tout était entre les mains des partenaires. Pour accéder dans un véhicule de la riposte, il fallait vraiment beaucoup des manœuvres donc à la prochaine, il faut arranger ce problème, il faut valoriser les agents de l’Etat par rapport à la gestion de la riposte

**P45: IA_05_BCZS_IS.rtf - 45:1 [Répondant : Oui, tout juste au..] (4:4)	(Super)**

Codes:	[Début-Progression_Epidémie MVE]

No memos

**Répondant** : Oui, tout juste au début nous n’avions pas su que nous étions en épidémie et nous étions seulement alertés par le décès d’une fille stagiaire de centre de santé de référence de Mangina, il y a les collègues de Mangina qui nous ont appelés qu’il y a une fille qui est décédée mais ce décès présente des signes général pour tous les malades qui décédé présentement à Mangina et c’est en ce moment-là que l’un des superviseurs de la zone de santé étant ami à celui qui l’appelait est parti investiguer à Mangina et finalement à Magina, il y a beaucoup des décès actuellement et c’est décès-là a commencé par l’un des nous, une fille stagiaire de l’ITM et ces décès-là qu’est-ce qui se passe ? et A partir d’un moment, on a commencé à enregistrer les décès sur décès qui ont presque les mêmes symptômes donc quelqu’un qui vient pour un symptôme de paludisme, on le soigne et trois après quand on veut le faire sortir, c’est alors qu’il va présenter des signes hémorragiques. Il y a mêmes des cadavres qui saignaient alors en ce moment quand nous sommes arrivés là-bas, nous sommes arrivés c’était le 27 juillet 2018, on est descendu avec le médecin chef de zone et toute l’équipe de la zone de santé pour s’enquérir de la situation et quand on a essayé de demander, ce jour-là on avait trouvé douze cadavres dans la cité de Mangina et il y avait d’autres cadavres qui étaient venus de Beni et d’autres étaient de là mais ils avaient trouvé la mort dans le village de Mangina. Alors quand on avait poussé les investigations, nous on a conclu directement que c’était une autre maladie qu’eux étaient en train de penser et c’est en ce moment-là qu’on avait fait appel à la DPS et puis ils nous ont appuyé en disant faites un rapport circonstanciel par rapport au cas des décès que vous enregistrez chez vous là-bas et puis nous nous allons venir vous appuyer. Et c’est comme ça qu’ils sont venus nous appuyer et nous avons même prélevé et c’est ce prélèvement qui nous a révélé que c’était Ebola. Mais en ce qui concerne les signes si quelqu’un était formé, il pouvait voir que tous les signes étaient ceux de la maladie Ebola.

**P45: IA_05_BCZS_IS.rtf - 45:2 [Répondant : Bon ; il y avait d..] (6:6)	(Super)**

Codes:	[Début-Progression_Epidémie MVE]

No memos

**Répondant** : Bon ; il y avait déjà un mois depuis qu’on enregistrait les décès, il y avait déjà un mois donc c’était au mois de juillet et les décès avaient probablement au mois de mai. Une épidémie des décès dans la cité de Mangina

**P45: IA_05_BCZS_IS.rtf - 45:3 [Répondant : c’est nous qui avo..] (8:8)	(Super)**

Codes:	[Répondant_Responsabilité_pdt MVE]

No memos

**Répondant** : c’est nous qui avons faits les premières investigations pour essayer d’envoyer le rapport à notre hiérarchie à Goma et après ça nous avions reçu le chargé de la surveillance épidémiologie du niveau de Goma, la surveillance épidémiologique qui était venu nous appuyer et avec lui nous avons fait les prélèvements et quand les résultats étaient sortis positifs, nous avons entré directement dans la riposte contre Ebola avant même que les Kinois n’arrivent, on a quand même fait quelques chose comme isolement donc il faut isoler un tel cas un tel cas en attendant la prise en charge

**P45: IA_05_BCZS_IS.rtf - 45:4 [Répondant : quand les Kinois s..] (10:10)	(Super)**

Codes:	[Répondant_Responsabilité_pdt MVE]

No memos

**Répondant** : quand les Kinois sont venus, ils nous ont encore appuyés et moi je suis resté dans la surveillance où on fait la recherche des cas au niveau des FOSA, des investigations des cas, on était parfois et au début-là on était appelé par la communauté au début-là quand c’était encore à Mangina. On était appelé par la communauté et venait voir un cas de malade ici, on faisait des investigations et après investigations, il y a certains malades qu’on conduisait au CTE et il tombait positif et après ça nous sommes resté à faire la recherche active même jusqu’aujourd’hui

**P45: IA_05_BCZS_IS.rtf - 45:5 [Répondant : juste au début com..] (12:12)	(Super)**

Codes:	[Considération_Ebola_population]

No memos

**Répondant** : juste au début comme Ebola a commencé dans un quartier où il y avait le cas indexé, ces gens-là croyaient à une sorcellerie, ils disaient ceci, je veux raconter ce qu’ils disaient : donc celui que nous nous considérons comme cas index dans la zone que celui-là avait mangé le chat de sa tante, après avoir mangé ce chat-là, c’est en ce moment que la tante avait jeté le mauvais sort. Ça c’était la première information ; la deuxième information, on disait que c’est quelqu’un qui est parti chercher une sirène pour pouvoir s’enrichir alors il a échoué dans les conditions et il a jeté cette sirène au niveau de la rivière Makeke là-bas à Mangina et c’est dès là qu’est sorti le mauvais sort pour tuer les gens

**P45: IA_05_BCZS_IS.rtf - 45:6 [Répondant : Non, non tout le m..] (14:14)	(Super)**

Codes:	[Considération_Ebola_population]

No memos

**Répondant** : Non, non tout le monde qui habitait là-bas, nous nous avons posé la question, ce n’est pas possible moi j’ai mangé la viande du chat, il y a l’autre qui est venu assister à ce deuil et comme celui qui n’a pas mangé de cette viande, comme il peut tomber dans les mêmes symptômes et il est décédé comme celui-là, c’était ça les rumeurs avant Ebola. Jusque maintenant comme nous nous sommes à Mangina, Mangina a finalement compris après le décès de plusieurs personnes à Mangina

**P45: IA_05_BCZS_IS.rtf - 45:7 [Répondant : Bon, le slogan d’E..] (16:16)	(Super)**

Codes:	[Considération_Ebola_population]

No memos

**Répondant** : Bon, le slogan d’Ebola business a commencé quand Ebola a pris de l’ampleur, quand on avait commencé au début il n’y avait pas beaucoup de monde. Nous étions là presque seul et quand il y a eu l’OMS qui est venu et d’autres kinois qui sont venus nous appuyer alors c’est en ce moment-là que les gens de Mangina ont commencé à dire que vous ne voulez pas nous aligner dans cette histoire donc derrière ça parce que vous êtes en train de vois enrichir et c’était cette Ebola-là. C’est dès là que la rumeur est remontée et ils ont dit que vous vous enrichissez vous-mêmes et ils ont même convoqué le médecin chez de zone et à la radio locale, on a dit au médecin chez de zone si vous ne voulez pas venir répondre à l’invitation des jeunes de Mangina, nous allons faire des actions de grandes envergures ; et quand il est arrivé là-bas on lui a dit que voilà Ebola est chez nous mais vous nous amenez des gens à partir de loin pour venir travailler or nous sommes ici et nous pouvons constituer une ressource et voilà c’est comme ça qu’Ebola business a commencé

**P45: IA_05_BCZS_IS.rtf - 45:8 [Répondant : Négatif, c’est nou..] (18:18)	(Super)**

Codes:	[Ressources humaines_Implication-agents-locaux]

No memos

**Répondant** : Négatif, c’est nous qui avons commencé cette épidémie et nous n’étions pas formés et c’est ça la grande problématique, on ne savait pas comment s’y prendre, juste quand les kinois sont venus et nous ont appuyé par des formations alors quand on trouvait des cas dans une aire de santé, d’office l’IT était impliquait dans la recherche des gens qui devaient faire les suivis des contacts. Il était impliqué d’office pour faire les suivis des contacts parce que c’est lui qui a des relais et on va lui demander nous venons de trouver un cas. Qui vont faire le suivi des contacts ? C’est l’IT qui nous donnait ces gens.

**P45: IA_05_BCZS_IS.rtf - 45:9 [Répondant : Oui, il y a eu tro..] (20:20)	(Super)**

Codes:	[Structures-Services_fonctionnement]

No memos

**Répondant** : Oui, il y a eu trop des changements. Je veux prendre le service préventif, il n’y avait plus des séances de vaccination, je veux prendre le service du curatif

**P45: IA_05_BCZS_IS.rtf - 45:10 [Répondant : parce que tout le ..] (22:22)	(Super)**

Codes:	[Service_vaccination_Fonctionnement]

No memos

**Répondant** : parce que tout le monde était suspect donc tout le monde ne voulait pas et dire à vrai il y avait d’abord une rupture des relations entre la communauté et le personnel soignant ; pourquoi cette rupture ? Parce que la population croyait à Ebola business donc tout le monde qui était impliqué parce que nous devons impliquer nos relais avec qui nous travaillons au quotidien, quand nous les impliquons dans la recherche active, nous les impliquons dans les suivis des contacts, nous les impliquons dans la surveillance à base communautaire, automatiquement ils étaient hais par la communauté et tout ce qui concerne nos activités ça boitait parce que c’est le même relais qui va conscientiser les malades d’aller à la structure et quand il vient le malade va lui dire que tu veux m’amener au CTE pour que tu puisses toucher de l’argent. Le service préventif, quand l’IT vient ou le relais vient, on dit que c’est Ebola qui vient, moi je ne peux plus coopérer avec ces gens-là, ce sont des sorciers, ce sont eux qui sont en train de nous vendre et c’était ça le problème

**P45: IA_05_BCZS_IS.rtf - 45:11 [Répondant : Oui, il y a eu la ..] (24:24)	(Super)**

Codes:	[Service_vaccination_Fonctionnement]

No memos

**Répondant** : Oui, il y a eu la vaccination parce qu’on ne pouvait même plus vacciné et on ne pouvait plus faire le sensibilisation parce que la population avait déjà était contre tout personnel de santé, tout relais communautaire. Il n’y avait pas eu de vaccination, il y a eu même une lettre qui est venue de la DPS soi-disant ne vacciner plus les enfants parce que vous ne savez pas le statut de l’enfant que vous allez toucher et leurs parents ne connaissent non plus vos statuts vous qui allez vacciner ces enfants, directement on a suspendu ces activités-là

**P45: IA_05_BCZS_IS.rtf - 45:12 [Répondant : les activités ont ..] (26:26)	(Super)**

Codes:	[Service_vaccination_Fonctionnement]

No memos

**Répondant** : les activités ont été suspendues pendant plus de 4 à 6 mois comme ça

**P45: IA_05_BCZS_IS.rtf - 45:13 [Répondant : je ne sais pas, il..] (28:28)	(Super)**

Codes:	[Instructions_pdt_MVE]

No memos

Répondant : je ne sais pas, il faut voir au niveau du secrétariat. Je sais bien qu’il y a une lettre qui est sortie interdisant la vaccination et c’est venu du PEV Goma.

**P45: IA_05_BCZS_IS.rtf - 45:14 [Répondant : en tout cas, je pe..] (30:30)	(Super)**

Codes:	[Structures-Services_fonctionnement]

No memos

**Répondant** : en tout cas, je peux vous dire c’est presque tous les services parce que je sais que le centre de santé de Mangina a fermé momentanément donc il n’y avait personne qui ne pouvait venir au centre de santé et les autres centres de santé, les services de curatif, comme il y avait des gens de la recherche active et jusqu’à maintenant qui venaient cherché les malades là-bas alors quelqu’un qui avait sa maladie, son paludisme ne pouvait plus venir soi-disant quand je veux venir là-bas ils vont alerter soi-disant que je suis un cas suspect d’Ebola donc même les services curatif, le service promotionnel, tout n’a pas marché pendant cette période

**P45: IA_05_BCZS_IS.rtf - 45:15 [Répondant : les malades dans u..] (32:32)	(Super)**

Codes:	[Structure-Service_Utilisation_Alternatives_population]

No memos

**Répondant** : les malades dans un premier temps, on avait pris les grandes structures de la zone et maintenant on a constaté que les malades ne fréquentaient plus nos structures mais aller aux postes de santé. Nous avons aussi intégrer la surveillance à base communautaire au niveau des postes de santé et la surveillance aussi au niveau des postes de santé alors les malades, ils commencent aller dans les officines pharmaceutiques pour s’achètent les médicaments et les autres allaient vers les tradipraticiens et on a aussi intégrer les tradipraticiens et c’est maintenant l’automédication qui a pris place pendant ce temps-là, c’est automédication

**P45: IA_05_BCZS_IS.rtf - 45:16 [Répondant : je veux d’abord co..] (34:35)	(Super)**

Codes:	[Triage-Isolement-Chlore-thermoflash_Réaction_population]

No memos

**Répondant** : je veux d’abord commencer par la présence des agents de la recherche active, ça faisait fuir les malades parce que quand il y aura une petite température de 38°, on va alerter que je suis un cas suspect et il y aura déjà l’ambulance va venir me chercher sans même mon consentement. Il y a eu le triage avec l’utilisation de thermo flash avant même jusqu’aujourd’hui à Ngazi, il faut d’abord négocier avec un malade pour essayer de prendre sa température parce qu’ils disaient quoi ? Non on est en train de nous tuer en catimini par leur il prend ça comme un revolver donc le triage, ils ont peur du triage parce qu’ils seront tempérés. Le thermo flash parce que c’est une bombe à retardement, ça va les tuer doucement. Vous avez parlé de quoi encore ?

Le lavage des mains, les gens ont eu peur de lavage des mains auparavant soi-disant que nous avons échoué à les tuer par les alertes maintenant nous sommes en train d’amener un système de lavage des mains où on met un produit qui les tue doucement heureusement aujourd’hui Dieu merci tout monde commence à se laver les mains. Alors ça commençait quand on disait qu’on peut se laver les mains avec le savon mais avant c’était seulement le chlore. A un moment donné, on a senti qu’il a une résistance au chlore et on a amené le savon et les gens ont commencé à se laver les mains

**P45: IA_05_BCZS_IS.rtf - 45:17 [Répondant : Oui, le triage ave..] (37:37)	(Super)**

Codes:	[Triage-Isolement-Chlore-thermoflash_Réaction_population]

No memos

**Répondant** : Oui, le triage avec l’isolement ça commençait ici récemment mais avant il n’y avait pas

**P45: IA_05_BCZS_IS.rtf - 45:18 [Répondant : il y a deux grands..] (39:39)	(Super)**

Codes:	[Considération_CTE_population]

No memos

**Répondant** : il y a deux grands problèmes parce que nous quand on a commençait le CTE à Mangina, il y avait la première cause, la séparation du malade avec sa famille, ils n’avaient pas compris que le CTE, c’est les soins intensifs. Ils disaient que vous nous séparez de nos familles et on ne sait pas comment vous êtes en train de traiter nos malades là-bas. Il y a eu ici un taux des mortalités très élevés où la CTE a été comparée à un mouroir, ça aussi ça leur effrayait.

**P45: IA_05_BCZS_IS.rtf - 45:19 [Répondant : là aussi il y avai..] (41:41)	(Super)**

Codes:	[Qualité_soins_Prestataires]

No memos

**Répondant** : là aussi il y avait un problème parce qu’avec Ebola quand on arrive dans la structure, on prend deux, trois personnes qui doivent intégrer la recherche active non seulement dans la structure mais dans toute l’aire de santé et il y avait d’autres infirmiers qui sont partis au niveau du CTE donc il y avait fuite des cerveaux, ça empiétait la qualité des soins parce que quand vous arrivez au niveau de la structure, vous allez trouver un nombre très réduits des infirmiers et des médecins et quand ils sont surchargés, ils ne pouvaient pas donner les soins de qualité et ils étaient tellement surchargés parce que cette période avait coïncidé avec une gratuité où la population a commencé à venir petit à petit et en arrivant au niveau de la structure, il n’y avait presque pas du personnel parce que le personnel était parti dans la riposte.

**P45: IA_05_BCZS_IS.rtf - 45:20 [Répondant : Non, chacun cherch..] (43:43)	(Super)**

Codes:	[Ressources_humaines_Disponibilité et perturbations]

No memos

**Répondant** : Non, chacun cherche là où il voit mieux et à la riposte était mieux par rapport à la structure

**P45: IA_05_BCZS_IS.rtf - 45:21 [Répondant : non, non, c’était ..] (45:45)	(Super)**

Codes:	[Ressources_humaines_Disponibilité et perturbations]

No memos

**Répondant** : non, non, c’était impossible dans quel sens, on arrive au niveau de la structure et quand on arrive au niveau de la structure, on dit non on va prendre un infirmier ou deux infirmiers qui vont intégrer la riposte, ces infirmiers partent sans même demander l’avis de la zone de santé, vous allez vous rendre compte qu’ils travaillent au niveau de CTE et qui lui a fait la lettre de partir ? Et il vous dit franchement que moi je suis dans les affaires privées. En tout cas il ne nous demandait pas d’avis

**P45: IA_05_BCZS_IS.rtf - 45:22 [Répondant : en ce qui concerne..] (47:47)	(Super)**

Codes:	[Prestataire_Attitude_devant_malade]

No memos

**Répondant** : en ce qui concerne la protection contre Ebola, la petite quantité des prestataires qui est restée, tout le monde avait peur parce qu’il ne connaissait pas le statut de celui qui devait venir et on a aussi enregistré beaucoup des gaffes par rapport à ça quelqu’un qui vient en train de saigner, on vous dit non, non, il faut l’isoler parce que c’est un cas suspect, il faudrait que l’équipe de la recherche active vienne pour faire des investigations, après ça que vous pouvez toucher le malade. Il y a ceux qui sont partis par rapport à ça, quelqu’un vient avec les signes d’Ebola, pourquoi ? Parce que tout le monde n’était pas formé et les épis n’étaient pas disponible en quantité avant.

**P45: IA_05_BCZS_IS.rtf - 45:23 [Répondant : Oui, des sérieux p..] (49:49)	(Super)**

Codes:	[Organisation_travail-Circuit des malades_pendant MVE]

No memos

**Répondant** : Oui, des sérieux problèmes parce que vous pouvez venir, vous arrivez à 9 heures par exemple parce que moi je travaillais dans la recherche active. A 9 heures, on a une réunion à Mangina, la réunion matinale et puis le malade est arrivé à 9 heures, la structure a eu le temps de l’isoler, il va attendre jusqu’à ce que l’investigateur vienne de Mangina ; s’il arrive à 11 heures, le malade sera là sans soins ; sans même un soutien psychologique. Le délaisser dans une chambre et quand ces gens vont venir, ils vont faire des investigations et s’ils valident le cas, le cas va attendre l’ambulance et même si l’ambulance venait à 16 heures, il sera là isoler sans recevoir des soins jusqu’allait recevoir les soins à Mangina, c’est ça le problème.

**P45: IA_05_BCZS_IS.rtf - 45:24 [Répondant : il y a ce que je d..] (51:51)	(Super)**

Codes:	[Organisation_travail-Circuit des malades_pendant MVE]

No memos

**Répondant** : il y a ce que je dis, il y a ceux qui sont décédés par rapport au retard de prise en charge

**P45: IA_05_BCZS_IS.rtf - 45:25 [Répondant : moi la recommandat..] (53:53)	(Super)**

Codes:	[Organisation_travail-Circuit des malades_pendant MVE]

No memos

**Répondant** : moi la recommandation, je veux toujours l’adresser à la hiérarchie s’il faut former les gens contre quelque chose qui peut venir, il faut qu’on former tout le monde. Et il faut qu’il fasse un bon plan de contingence pour dire telle épidémie peut venir, il faut qu’on ait quelque chose qui peut nous aider à faire face à ça, c’était ça le problème. C’est la première recommandation, vraiment la formation à tous les niveaux et puis vraiment le plan de contingence, il faut vraiment qu’on respecte ça

**P45: IA_05_BCZS_IS.rtf - 45:26 [Répondant : oui, nous, nous av..] (55:55)	(Super)**

Codes:	[Médicaments_disponibilité_fosa]

No memos

**Répondant** : oui, nous, nous avons reçu les médicaments de la part du gouvernement, le projet PDSS et ces médicaments étaient en qualité et en quantité dans les structures donc toutes les structures qui sont dans notre pyramide sanitaire

**P45: IA_05_BCZS_IS.rtf - 45:27 [Répondant : Oui, c’est la grat..] (57:57)	(Super)**

Codes:	[Gratuité_Début]

No memos

**Répondant** : Oui, c’est la gratuité des soins avec PDSS

**P45: IA_05_BCZS_IS.rtf - 45:28 [Répondant : bon, avant c’était..] (59:59)	(Super)**

Codes:	[Gratuité_Accueil_population]

No memos

**Répondant** : bon, avant c’était compliqué, les disaient comme nous avons refusé leur vaccin et ils veulent nous amener les médicaments gratuitement, c’est une autre façon de nous tuer mais après des séances de sensibilisation, ils ont quand même adhérer parce que nous étions obligé d’entrer dans la sensibilisation pour dire à la communauté que c’est nous qui vous servons ces médicaments et depuis longtemps qu’on vous servait avec ces mêmes médicaments, c’est aujourd’hui qu’on va mettre Ebola dans les médicaments

**P45: IA_05_BCZS_IS.rtf - 45:29 [Répondant : l’épidémie, c’étai..] (61:61)	(Super)**

Codes:	[Gratuité_Début]

No memos

**Répondant** : l’épidémie, c’était déclaré en aout et la gratuité a été décrétée en octobre donc après deux mois

**P45: IA_05_BCZS_IS.rtf - 45:30 [Répondant : non, il n’y avait ..] (63:63)	(Super)**

Codes:	[Gratuité_Début]

No memos

**Répondant** : non, il n’y avait pas de gratuité et même à Mangina, on a dit d’offrir un service gratuit parce que l’épidémie était encore à Mangina.

**P45: IA_05_BCZS_IS.rtf - 45:31 [Répondant : oui, de tuer la po..] (65:65)	(Super)**

Codes:	[Gratuité_Rumeurs]

No memos

**Répondant** : oui, de tuer la population

**P45: IA_05_BCZS_IS.rtf - 45:32 [Répondant : je dis quand on a ..] (67:67)	(Super)**

Codes:	[Gratuité_Conséquences_utilisation_services]

No memos

**Répondant** : je dis quand on a maitrisé Ebola, on a commencé la sensibilisation et le gens ont compris, c’est le taux d’utilisation des services qui a augmenté par rapport à cette gratuité-là

**P45: IA_05_BCZS_IS.rtf - 45:33 [Répondant : le rapportage des ..] (69:69)	(Super)**

Codes:	[SNIS_fonctionnement_Decrire_pdt MVE]

No memos

**Répondant** : le rapportage des données ça connu aussi un petit problème, pourquoi ? le surcharge de l’infirmier titulaire et il y a des moments donnés le rapport venait pas, donc vous arrivez là-bas, celui qui va vous produire le rapport n’est pas disponible, il est dans la recherche active, il est dans une telle ou autre commission et puis le délai, c’est la 7 mais il y a ceux qui amené le rapport même le 20 et après on avait fait un recouvrement terrible de ces rapports-là donc le rapportage n’a pas été bien, ça connu un retard

**P45: IA_05_BCZS_IS.rtf - 45:34 [Répondant : quand on analysait..] (71:71)	(Super)**

Codes:	[SNIS_fonctionnement_Decrire_pdt MVE]

No memos

**Répondant** : quand on analysait les données, premièrement je peux dire qu’il y avait des données fabriquées donc quand il se sent en retard de confectionner le rapport, il pouvait mettre les chiffres au hasard et alors ces chiffres quand on les mettait dans la machine lors de l’analyse des données, ça pouvait nous donner un pourcentage au-delà même de 200% et quand on demandait le jour de l’analyse, il vous dira que ce chiffre-là je ne sais pas qui l’a donné et le vrai chiffre se trouve dans le registre donc il a fait le canevas en hâte pour nous satisfaire en nous le donnant et puis quand il va aller consulter son registre, il va trouver que ce ne sont pas les chiffres qui sont dans son registre

**P45: IA_05_BCZS_IS.rtf - 45:35 [Répondant : non, la solution n..] (73:73)	(Super)**

Codes:	[SNIS_fonctionnement_Solutions aux défis]

No memos

**Répondant** : non, la solution nous avons fait des supervisions, nous avons fait des réunions, les supervisions pour comparer les données du canevas et les données du registre, c’était ça le problème et quand on trouvait qu’il y a des incohérences, on a dit à l’IT que mon cher nous allons te laisser une seule voie, soit tu rentres dans la riposte soit tu restes chez nous parce que ces données ne nous engagent non plus nous à notre niveau

**P45: IA_05_BCZS_IS.rtf - 45:36 [Répondant : Oui, les réunions ..] (75:75)	(Super)**

Codes:	[Réunion_pdt-épidémie-Ebola]

No memos

**Répondant** : Oui, les réunions ont été affectées, les supervisions de même pourquoi ? Parce que nous aussi nous étions là, je ne peux pas accuser les autres, nous aussi nous étions dans la riposte. Parfois les supervisions et même si on était dans la riposte mais on faisait tout le temps dans les aires de santé mais quand vous arrivez, vous pouvez manquer quelqu’un à qui vous pouvez faire un entretien de supervision. Vous arrivez en même temps, il y a le véhicule de l’OMS qui vienne avec les gens de la recherche active, l’IT va vous laisser dans le bureau et va d’abord répondre aux préoccupations de ces gens-là qui sont venus, il vous laisse là et après un moment vous allez vous retrouver seul et vous partez ou bien on va dire à l’IT, bon c’était aussi normal, vous arrivez l’IT on a dit qu’il y a un contact ici qui a fui chez vous ou bien il y a un malade qui présente tel signe, tel signe, allons le voir et l’IT va voir qu’Ebola est une urgence par rapport à ce que vous êtes venu faire, il va vous laisser là-bas

**P45: IA_05_BCZS_IS.rtf - 45:37 [Répondant : Bon, pas tellement..] (77:77)	(Super)**

Codes:	[Gouvernance_soins santé]

No memos

**Répondant** : Bon, pas tellement. Ça je ne peux pas les accuser faussement.

**P45: IA_05_BCZS_IS.rtf - 45:38 [Répondant : ça n’a pas changé ..] (79:79)	(Super)**

Codes:	[Gouvernance_soins santé]

No memos

Répondant : ça n’a pas changé la capacité d’un côté négatif ou d’un côté positif sauf tout monde courrait derrière l’argent. Je venais de vous dire quand un partenaire vient, il dit à l’IT venait on va faire telle, telle chose dans votre centre de santé, il va vous laisser là-bas et en ce moment votre leadership vous n’allez pas l’exercer devant quelqu’un qui vient de voir l’argent devant lui, elle va vous laisser et elle va partir

**P45: IA_05_BCZS_IS.rtf - 45:39 [Répondant : moi selon ; c’est ..] (81:81)	(Super)**

Codes:	[PCI_Comment_maintenir]

No memos

**Répondant** : moi selon ; c’est ce que je suis en train de dire aux IT de ne pas se fier à la politique de la main tendue. Je dis que si le partenaire nous est venu en appui, il est venu nous traçait le chemin, il dit si tu fais ça, ça, ça tu risques de ne pas avoir ceci, ceci ou ceci alors c’est nous avec le frais de fonctionnement de pérenniser les actions et d’acheter ce qui se trouve au niveau du marché, ça je suis en train de dire

**P45: IA_05_BCZS_IS.rtf - 45:40 [Répondant : oui, ça sera suivi..] (83:83)	(Super)**

Codes:	[PCI_Comment_maintenir]

No memos

**Répondant** : oui, ça sera suivi pour les choses que nous pouvons trouver les lavabos, les savons, en tout cas les choses qu’on peut trouver sur les marchés, les IT vont continuer à les faire parce que je leur ai dit qu’après l’épidémie d’Ebola et même si Ebola finissait nous devons faire attention et faire la surveillance comme nous la faisons pour les autres maladies donc Ebola va entrer dans la surveillance donc la population continue à se laver les mains parce que se laver les mains, ce n’est pas seulement contre Ebola mais aussi contre les autres maladies, les maladies des mains sale, je suis en train de prêcher ça aux IT. On a eu peut-être l’occasion la formation des CAC et tout ça on est en train de vulgariser ces messages. Maintenant le comble, c’est les épis qui ne peuvent pas être sur le marché, comme les sacs mortuaires, comme les blouses jetables, je ne sais pas si ça s’est trouvé sur le marché ? Mais quand même le thermo flash ça on peut trouver et les autres aussi

**P45: IA_05_BCZS_IS.rtf - 45:41 [Répondant : dans notre système..] (85:85)	(Super)**

Codes:	[Agression-Menaces_Structure-Prestataire_solution]

No memos

**Répondant** : dans notre système, nous avons eu la chance de suivre la formation des membres de CAC donc quand on a fait la sélection, c’était pendant la flambée de l’épidémie alors la population n’a pas choisi tout le monde qui prestait dans la riposte comme relais communautaire, on a choisi les autres qui n’étaient pas dans le système de la riposte et on croit qu’avec ceux-là qui ont été formé, on va les former et les superviser parce que la population a encore confiance en ces gens qui ont été nouvellement formés. Peut-être, chez nous c’est l’unique avantage que nous avons mais sinon avec les anciens, ils ne peuvent pas dire quelque chose et la population puisse facilement accepter, c’est impossible

**P45: IA_05_BCZS_IS.rtf - 45:42 [Répondant : c’est ce que je di..] (87:87)	(Super)**

Codes:	[Changements_depuis_Ebola]

No memos

**Répondant** : c’est ce que je dis non, rupture de confiance totale avec les soignants et les soignés, c’est ça vraiment la rupture de la confiance

**P45: IA_05_BCZS_IS.rtf - 45:43 [Répondant : je le dis d’une au..] (89:89)	(Super)**

Codes:	[Souhait_Changement_gestion_futures_épidémies]

No memos

**Répondant** : je le dis d’une autre façon, la première chose c’est de former les gens sur toutes les épidémies possibles qui peuvent venir. C’est ce que j’ai toujours dit même aux gens de Goma, pourquoi ? Quand il y a certaines choses, il privilégie certaines zones de santé, c’est ce que les zones de santé de Mutwanga, Beni, Kamango, elles étaient formées en Ebola avant et on nous a laissé ici chez nous. La deuxième chose, le plan de la contingence de la province, il faudrait qu’on élabore et rendre disponible les intrants parce que je vois que quand le docteur Guy est venu, le responsable de la surveillance, il est venu qu’avec trois sacs mortuaires sans autres choses, trois sacs mortuaires, on te dit qu’il y a douze décès par jour et toi tu viens avec trois sacs, ça va servir comment ? Nous avons reçu MSF le tout premier mois là-bas vers le 5 aout, MSF qui a amené un carton de 25 pièces de SRO, tu vois, il faut que notre province élabore un plan de contingence de toutes les épidémies et qu’il rende disponibles les intrants de toutes ces épidémies-là comme ça quand il y a une épidémie, on peut prendre le stock dans la province et versé dans la zone affectée et sinon, on insiste sur la formation des agents. Qu’on nous forme à toutes les épidémies possibles

**P46: IA_06_BCZS_AG.rtf - 46:1 [Répondant : La gratuité des so..] (9:9)	(Super)**

Codes:	[Gratuité_Début]

No memos

DATA SOURCE UNACCESSIBLE!: C:\Users\Dr Gabriel Kyomba\AppData\Roaming\Scientific Software\ATLASti\Repository\Managed Files\Partie 2\IA_06_BCZS_AG_815F3F168484FE3CF145CF64BD0B7EE_rtf.atldoc

**P46: IA_06_BCZS_AG.rtf - 46:2 [Répondant : La zone est entrée..] (11:11)	(Super)**

Codes:	[Début-Progression_Epidémie MVE]

No memos

DATA SOURCE UNACCESSIBLE!: C:\Users\Dr Gabriel Kyomba\AppData\Roaming\Scientific Software\ATLASti\Repository\Managed Files\Partie 2\IA_06_BCZS_AG_815F3F168484FE3CF145CF64BD0B7EE_rtf.atldoc

**P46: IA_06_BCZS_AG.rtf - 46:3 [Répondant : Il y avait le tick..] (13:13)	(Super)**

Codes:	[Recouvrement_coûts-des-soins_avant gratuité]

No memos

DATA SOURCE UNACCESSIBLE!: C:\Users\Dr Gabriel Kyomba\AppData\Roaming\Scientific Software\ATLASti\Repository\Managed Files\Partie 2\IA_06_BCZS_AG_815F3F168484FE3CF145CF64BD0B7EE_rtf.atldoc

**P46: IA_06_BCZS_AG.rtf - 46:4 [Répondant : Le projet là en 20..] (15:15)	(Super)**

Codes:	[Recouvrement_coûts-des-soins_avant gratuité]

No memos

DATA SOURCE UNACCESSIBLE!: C:\Users\Dr Gabriel Kyomba\AppData\Roaming\Scientific Software\ATLASti\Repository\Managed Files\Partie 2\IA_06_BCZS_AG_815F3F168484FE3CF145CF64BD0B7EE_rtf.atldoc

**P46: IA_06_BCZS_AG.rtf - 46:5 [Répondant : Le même projet a c..] (17:17)	(Super)**

Codes:	[Recouvrement_coûts-des-soins_avant gratuité]

No memos

DATA SOURCE UNACCESSIBLE!: C:\Users\Dr Gabriel Kyomba\AppData\Roaming\Scientific Software\ATLASti\Repository\Managed Files\Partie 2\IA_06_BCZS_AG_815F3F168484FE3CF145CF64BD0B7EE_rtf.atldoc

**P46: IA_06_BCZS_AG.rtf - 46:6 [Répondant : Tous les services...] (19:19)	(Super)**

Codes:	[Gratuité_Structures-Services_concernés]

No memos

DATA SOURCE UNACCESSIBLE!: C:\Users\Dr Gabriel Kyomba\AppData\Roaming\Scientific Software\ATLASti\Repository\Managed Files\Partie 2\IA_06_BCZS_AG_815F3F168484FE3CF145CF64BD0B7EE_rtf.atldoc

**P46: IA_06_BCZS_AG.rtf - 46:7 [Répondant : Le paquet compléme..] (21:21)	(Super)**

Codes:	[Gratuité_Structures-Services_concernés]

No memos

DATA SOURCE UNACCESSIBLE!: C:\Users\Dr Gabriel Kyomba\AppData\Roaming\Scientific Software\ATLASti\Repository\Managed Files\Partie 2\IA_06_BCZS_AG_815F3F168484FE3CF145CF64BD0B7EE_rtf.atldoc

**P46: IA_06_BCZS_AG.rtf - 46:8 [Répondant : Il y avait ce que ..] (23:23)	(Super)**

Codes:	[Gratuité_Défis]

No memos

DATA SOURCE UNACCESSIBLE!: C:\Users\Dr Gabriel Kyomba\AppData\Roaming\Scientific Software\ATLASti\Repository\Managed Files\Partie 2\IA_06_BCZS_AG_815F3F168484FE3CF145CF64BD0B7EE_rtf.atldoc

**P47: IA_06_BCZS_MCZS.rtf - 47:1 [Répondant : dans la zone de sa..] (2:2)	(Super)**

Codes:	[Début-Progression_Epidémie MVE]

No memos

**Répondant** : dans la zone de santé, nous avions enregistré d’abord 75 cas et le premier cas était un décès survenu dans une structure privée et il a été soigné comme tout autre malade sans protection et c’est après le décès que le prestataire avait constaté que le corps saigné de partout, c’est ainsi que les autres avaient alerté l’équipe de riposte qui sont venues voir et après on a conclu que c’était positif

**P47: IA_06_BCZS_MCZS.rtf - 47:2 [Répondant : effectivement, c’é..] (4:4)	(Super)**

Codes:	[Début-Progression_Epidémie MVE]

No memos

**Répondant** : effectivement, c’était un taximan qui allait à Beni, à Butembo qui faisait déjà de trafics alors que l’épidémie avait déjà commencé au niveau de Beni et de Butembo mais nous au niveau de la zone de santé, on n’était pas encore contaminé

**P47: IA_06_BCZS_MCZS.rtf - 47:3 [Répondant : nous avons 20 aire..] (6:6)	(Super)**

Codes:	[Début-Progression_Epidémie MVE]

No memos

**Répondant** : nous avons 20 aires de santé et nous avons sept aires de santé qui ont été affectées

**P47: IA_06_BCZS_MCZS.rtf - 47:4 [Répondant : pour la population..] (8:8)	(Super)**

Codes:	[Considération_Ebola_population]

No memos

**Répondant** : pour la population, il y avait beaucoup d’hypothèse, la première chose, c’était très compliqué parce que nous étions dans une période de guerre, ils ont pensé que c’était une guerre biologique d’abord et deuxièmement, ils ont pensé que c’est un business, c’était une maladie business parce que tout le monde qui devait travailler là-bas devait avoir de l’argent

**P47: IA_06_BCZS_MCZS.rtf - 47:5 [Répondant : on pensait que c’é..] (10:10)	(Super)**

Codes:	[Considération_Ebola_population]

No memos

**Répondant** : on pensait que c’était pour éliminer la population

**P47: IA_06_BCZS_MCZS.rtf - 47:6 [Répondant : oui pour les struc..] (12:12)	(Super)**

Codes:	[Structures-Services_fonctionnement]

No memos

**Répondant** : oui pour les structures qui ont enregistré les cas en tout cas les malades ont fuie les structures et aussi nous avons constaté que le taux de mortalité a augmenté même le taux de mortalité maternelle parce que les gens ne voulaient plus fréquenter les structures ; et ils venaient tardivement et venaient à mourir à la structure. Un moment donné dans les aires de santé où nous avions connu beaucoup des cas, nous avions trois aires de santé où on a connu plus de cas, nous avons à un moment donné suspendu quelques activités telles que la vaccination, la CPS et les restes

**P47: IA_06_BCZS_MCZS.rtf - 47:7 [Répondant : la raison était qu..] (14:14)	(Super)**

Codes:	[Structures-Services_fonctionnement]

No memos

**Répondant** : la raison était que la population n’était plus d’accord avec les services parce que quand on vaccinait contre Ebola or pour eux la vaccination contre Ebola était Ebola elle-même

**P47: IA_06_BCZS_MCZS.rtf - 47:8 [Répondant : le laboratoire, on..] (16:16)	(Super)**

Codes:	[Service_laboratoire_fonctionnement]

No memos

**Répondant** : le laboratoire, on avait suspendu dans toute la zone de santé le TDR et on a soigné tout cas de paludisme comme simple

**P47: IA_06_BCZS_MCZS.rtf - 47:9 [Répondant : Bon, ils ont d’abo..] (18:18)	(Super)**

Codes:	[Structure-Service_Utilisation_Alternatives_population]

No memos

**Répondant** : Bon, ils ont d’abord resté à la maison, ils automédiquent mais aussi au début ils allaient chez les tradipraticiens parce qu’au début les tradipraticiens n’étaient pas prise en compte dans la riposte et leurs services avaient un afflux des malades, on était obligé de les intégrer aussi dans les activités.

**P47: IA_06_BCZS_MCZS.rtf - 47:10 [Répondant : tout d’abord comme..] (22:22)	(Super)**

Codes:	[Organisation_travail-Circuit des malades_pendant MVE]

No memos

**Répondant** : tout d’abord comme on avait que deux centres de transit et de traitement, c’était seulement à Butembo et toutes les centres périphériques devaient envoyer tous les malades suspects à Butembo et après isolement du malade, on devait investiguer et après l’investigation, on devait maintenant alerté le services si l’investigation était maintenant validée étant validé on devait les envoyer au CT en attendant que le cas soit confirmé ou infirmé pour que le cas entre au CTE ou dans la structure qui a envoyé le cas. Alors le malade fuyait à un moment donné, il fuyait quand ils étaient dans l’isolement quand on ne faisait pas attention et il ne trouvait plus le malade parce qu’il est déjà reparti chez lui parce que pour eux, ils pensaient que toute personne qu’on envoyait au CT ou au CTE, c’était pour lui injecter la maladie et pour aller mourir donc pour eux, la personne qui entrait au CTE devait revenir mort

**P47: IA_06_BCZS_MCZS.rtf - 47:11 [Répondant : pour certains mala..] (24:24)	(Super)**

Codes:	[Considération_prestataire_EPI_population]

No memos

**Répondant** : pour certains malades, comme c’était une nouvelle pratique, les malades ont trouvé que les prestataires, on n’arrivait plus à les reconnaitre surtout quand on a mis les épis qui sont complémentaire au sein des zones rouges, ou des zones jaunes et ils avaient peur ;; l’outil par rapport à ça mais pour les prestataires et pour les structures de soins les équipements de protection individuelle ont été vraiment une protection réelle pour la prévention parce que nous avons la preuve à HGR Beni nous avons reçu deux cas positifs, d’abord un cas qui est décédé et comme on avait l’habitude d’utiliser les épis ça n’a pas posé problème, un deuxième cas qui était pendant une intervention chirurgicale alors que constaté déjà positif et qui a même été opéré et au premier jour post-opératoire il est entré en coma et c’est ça qui nous avait alerté et nous avions prélevé l’échantillon et au laboratoire, l’échantillon s’est révélé positif et maintenant nous avions envoyé au CTE mais sinon parmi nos prestataires, il n’y a eu personne qui a été contaminé par Ebola suite à l’utilisation des épis.

**P47: IA_06_BCZS_MCZS.rtf - 47:12 [Répondant : par rapport à l’ac..] (26:26)	(Super)**

Codes:	[Qualité_soins_Prestataires]

No memos

**Répondant** : par rapport à l’accueil, les malades étaient très résistants parce qu’ils avaient très peur et ils pensent qu’on se faisait de l’argent derrière eux comme des cas suspects, ils pensent que tout cas couterait 1000$ et que nous prestataires on faisait aussi de business parce que pour eux un décès couterait 1000$

**P47: IA_06_BCZS_MCZS.rtf - 47:13 [Répondant : oui, effectivement..] (28:28)	(Super)**

Codes:	[Ressources_humaines_Disponibilité et perturbations]

No memos

**Répondant** : oui, effectivement parce que le travail était devenu énorme, on n’avait pas, dans toutes les structures n’avaient pas par exemple les équipes de triage et c’était devenu une obligation pour toutes les structures donc il fallait recourir aux stagiaires ou alors on était obligé de recruter les journaliers pour nous aider à bien travailler

**P47: IA_06_BCZS_MCZS.rtf - 47:14 [Répondant : Effectivement, nou..] (30:30)	(Super)**

Codes:	[Ressources_humaines_Disponibilité et perturbations]

No memos

**Répondant** : Effectivement, nous avons eu deux médecins qui ont fuis le service parce qu’il semblait qu’il n’y avait pas de prise en charge par rapport à la prise en charge de la riposte Ebola. Ils sont partis et il y a un qui est là même jusqu’aujourd’hui parce qu’il veut d’abord avoir l’argent d’Ebola et après nous avons eu même des infirmiers qui ont fui les services qui sont allés travailler parce que dans les activités de la riposte, on est quand même bien payé. Oui, les gens ont soit d’argent, les gens ont soit d’être bien payés et les structures ne les prennent pas très bien en charge comme on les souhaite.

**P47: IA_06_BCZS_MCZS.rtf - 47:15 [Répondant : la grève avait eu ..] (32:32)	(Super)**

Codes:	[Ressources_humaines_situation_grève]

No memos

**Répondant** : la grève avait eu lieu, c’était au deuxième trimestre 2018 donc la grève venait de prendre fin

**P47: IA_06_BCZS_MCZS.rtf - 47:16 [Répondant : nous étions dans l..] (34:34)	(Super)**

Codes:	[Recouvrement_coûts-des-soins_avant gratuité]

No memos

**Répondant** : nous étions dans le tarif préétabli appuyer par l’Union Européenne et avec le tarif réduit, les services étaient quand même fréquenter surtout la gynéco-obstétrique qui était vraiment fréquenter et le service des ambulatoire donc l’hospitalisation était un peu….. et à partir et maintenant nous sommes dans la gratuité et le contrat court à partir du 15 février 2019 jusqu’au 30 juin 2020

**P47: IA_06_BCZS_MCZS.rtf - 47:17 [Répondant : nous étions dans u..] (36:36)	(Super)**

Codes:	[Recouvrement_coûts-des-soins_avant gratuité]

No memos

**Répondant** : nous étions dans un tarif forfaitaire

**P47: IA_06_BCZS_MCZS.rtf - 47:18 [Répondant : Oui, les malades d..] (38:38)	(Super)**

Codes:	[Recouvrement_coûts-des-soins_avant gratuité]

No memos

**Répondant** : Oui, les malades d’un même service paient la même chose donc forfaitaire par service

**P47: IA_06_BCZS_MCZS.rtf - 47:19 [Répondant : Non, non, Union eu..] (40:40)	(Super)**

Codes:	[Recouvrement_coûts-des-soins_avant gratuité]

No memos

**Répondant** : Non, non, Union européenne via Prodec

**P47: IA_06_BCZS_MCZS.rtf - 47:20 [Répondant : oui, c’est la grat..] (42:42)	(Super)**

Codes:	[Gratuité_Début]

No memos

**Répondant** : oui, c’est la gratuité des soins

**P47: IA_06_BCZS_MCZS.rtf - 47:21 [Répondant : le 16 février 2019..] (44:44)	(Super)**

Codes:	[Gratuité_Début]

No memos

**Répondant** : le 16 février 2019 jusqu’au 30 juin 2020

**P47: IA_06_BCZS_MCZS.rtf - 47:22 [Répondant : avec l’union europ..] (46:46)	(Super)**

Codes:	[Gratuité_Structures-Services_concernés]

No memos

**Répondant** : avec l’union européenne, nous sommes dans une gratuité totale

**P47: IA_06_BCZS_MCZS.rtf - 47:23 [Répondant : des rumeurs tout a..] (48:48)	(Super)**

Codes:	[Gratuité_Accueil_population]

No memos

**Répondant** : des rumeurs tout au début parce que la population était reçue à l’hôpital et on faisait aussi le triage mais quand ils ont trouvé qu’on a soigné les gens de la même manière comme avant ils ont compris

**P47: IA_06_BCZS_MCZS.rtf - 47:24 [Répondant : non, la gratuité, ..] (50:50)	(Super)**

Codes:	[Gratuité_Structures-Services_concernés]

No memos

**Répondant** : non, la gratuité, c’est seulement dans les structures étatiques

**P47: IA_06_BCZS_MCZS.rtf - 47:25 [Répondant : oui, même pour les..] (52:52)	(Super)**

Codes:	[Gratuité_Structures-Services_concernés]

No memos

**Répondant** : oui, même pour les structures étatiques si dans ce centre de santé, il y a un paquet complémentaire, le paquet complémentaire n’est pas pris en compte dans la gratuité. C’est ça la seule différence quoi

**P47: IA_06_BCZS_MCZS.rtf - 47:26 [Répondant : le paquet compléme..] (54:54)	(Super)**

Codes:	[Gratuité_Structures-Services_concernés]

No memos

**Répondant** : le paquet complémentaire dans les centres de santé que nous appelons ici centre de santé de référence, le paquet complémentaire n’est pas concerné par la gratuité des soins et on continue à appliquer le tarif forfaitaire pour le paquet complémentaire

**P47: IA_06_BCZS_MCZS.rtf - 47:27 [Répondant : étant donné que le..] (56:56)	(Super)**

Codes:	[Gratuité_Conséquences_motivation_prestataire]

No memos

**Répondant** : étant donné que le taux d’utilisation a augmenté, les conséquences positives sont très claires sur l’augmentation de la rémunération des prestataires parce que le travail est devenu aussi énorme

**P47: IA_06_BCZS_MCZS.rtf - 47:28 [Répondant : le défi comme je v..] (58:58)	(Super)**

Codes:	[Gratuité_Défis]

No memos

**Répondant** : le défi comme je vous ai dit que c’était dans un contexte compliqué, la population n’a pas accueilli cette gratuité avec joie, pour eux c’était une façon de les appeler en grand nombre à l’hôpital pour les éliminer au même moment et au début de la gratuité, ce n’est qu’après quand la population a compris qu’on a commencé à avoir un afflux des malades.

**P47: IA_06_BCZS_MCZS.rtf - 47:29 [Répondant : pour les structure..] (60:60)	(Super)**

Codes:	[Médicaments_disponibilité_fosa]

No memos

**Répondant** : pour les structures appuyées par l’union européenne, il n’y a eu des problèmes par rapport aux médicaments parce que lui payé les structures et c’est aux prestataires d’aller payés les médicaments à la centrale

**P47: IA_06_BCZS_MCZS.rtf - 47:30 [Répondant : c’est le prix de c..] (62:62)	(Super)**

Codes:	[Médicaments_disponibilité_fosa]

No memos

**Répondant** : c’est le prix de certains médicaments

**P47: IA_06_BCZS_MCZS.rtf - 47:31 [Répondant : en comparant la si..] (64:64)	(Super)**

Codes:	[SNIS_fonctionnement_Decrire_pdt MVE]

No memos

**Répondant** : en comparant la situation de la complétude, la situation ‘était plus la même

**P47: IA_06_BCZS_MCZS.rtf - 47:32 [Répondant : parce que les pres..] (66:66)	(Super)**

Codes:	[SNIS_fonctionnement_Decrire_pdt MVE]

No memos

**Répondant** : parce que les prestataires étaient tiraillés par les activités de la riposte donc ils avaient un travail double avec les activités de la riposte qui est venue s’ajouter

**P47: IA_06_BCZS_MCZS.rtf - 47:33 [Répondant : la qualité des don..] (68:68)	(Super)**

Codes:	[SNIS_fonctionnement_Decrire_pdt MVE]

No memos

**Répondant** : la qualité des données n’était pas très mauvaise, c’était presque la même chose sauf que la complétude n’était pas totale

**P47: IA_06_BCZS_MCZS.rtf - 47:34 [Répondant : Affirmatif, ça vra..] (70:70)	(Super)**

Codes:	[Gouvernance_soins santé]

No memos

**Répondant** : Affirmatif, ça vraiment était affecté, le travail était venu très double et certaines activités de routine souffraient dans le bureau central. La supervision de la riposte était différente de la supervision de routine. Pour la supervision de routine, je dois descendre et former les prestataires mais là on était pressé par le temps et avant 14 heures on doit déposer le rapport à la coordination et aussi par rapport aux réunions de chaque jour, on devait se réunir chaque jour pour la surveillance de la riposte

**P47: IA_06_BCZS_MCZS.rtf - 47:35 [Répondant : c’est une question..] (72:72)	(Super)**

Codes:	[Ressources humaines_Implication-agents-locaux]

No memos

**Répondant** : c’est une question très difficile étant donné que nos chefs sont en prison à cause de cette situation donc je m’abstiens d’en parler

**P47: IA_06_BCZS_MCZS.rtf - 47:36 [Répondant : il faut qu’on s’ap..] (74:74)	(Super)**

Codes:	[Souhait_Changement_gestion_futures_épidémies]

No memos

**Répondant** : il faut qu’on s’appuie seulement sur les structures sanitaires

**P47: IA_06_BCZS_MCZS.rtf - 47:37 [Répondant : pour les acquis de..] (76:76)	(Super)**

Codes:	[PCI_Comment_maintenir]

No memos

**Répondant** : pour les acquis de la PCI, il y aura des activités qui seront pérennisés automatiquement par exemple le port des épis de base mais par rapport à l’usage des épis complémentaire et aux autres aspects de la PCI, ça ne sera pas possible. Ça va revenir à la case du départ étant donné comme je venais de vous dire il y a des structures qui ont recrutés certains agents et qui vont les mettre de côté et puis qui va continuer à s’occuper du triage par exemple. Nous avons utilisé énormément les gants, les masques et consorts. Déjà aujourd’hui les structures sont déjà en rupture en gants, en masques et autres intrants approvisionnés par l’OMS et d’autres partenaires. Maintenant que nous n’avons plus d’appui, c’est ça le problème, on ne saura pas

**P47: IA_06_BCZS_MCZS.rtf - 47:38 [Répondant : Oui, j’avais juste..] (78:78)	(Super)**

Codes:	[Commentaire-Recommandation_Répondant]

No memos

**Répondant** : Oui, j’avais juste une précision par rapport aux épis, au début de l’épidémie, c’est vrai que je vous ai donné le premier cas mais c’est un cas positif ça signifie que chez nous comme l’épidémie avait commencé à Mangina et il y avait des cas probable qui avait alerté au niveau central et nous avions eu un cas probable qui venait de Mangina, c’était un enfant qui venait d’un orphelinat tenu par les religieux qui a fait qu’un enfant venu de Mangina et que sa mère venait de mourir. Sa grand-mère maternelle et sa grand-mère paternel étaient à l’hôpital et après elles seraient mortes alors le nourrisson avait fait juste deux jours ici et il faisait des fièvres et il est décédé dans ce tableau des fièvres, il faisait seulement la fièvre pas une autre signe pathologique même para clinique. C’était notre cas probable que nous avions reçu dans la zone et qui n’avait pas généré d’autres cas

**P47: IA_06_BCZS_MCZS.rtf - 47:39 [Répondant : il couvrait toutes..] (80:80)	(Super)**

Codes:	[Gratuité_Structures-Services_concernés]

No memos

**Répondant** : il couvrait toutes les aires de santé de la zone
