# Supplementary material for: Barriers and facilitators to healthcare facility utilization by non-Ebola patients during the 2018–2020 Ebola outbreak in the Democratic Republic of Congo
Source: Glob Health Res Policy. 2024 Nov 19;9:47. doi: 10.1186/s41256-024-00387-6 (PMC11575170; doi:10.1186/s41256-024-00387-6)
Supplement: Supplementary file 9 — Additional file 9. Ethical committee letter French. [file 41256_2024_387_MOESM9_ESM.pdf]

REPUBLIQUE DEMOCRATIQUE DU CONGO
Ministère de l'Enseignement Supérieur et Universitaire
Université de Kinshasa
ECOLE DE SANTE PUBLIQUE
**COMITE D'ETHIQUE**

***No d'Approbation: ESP/CE/11/2020***

Kinshasa, le 05 février 2020

**KYOMBA KALOMBE Gabriel**
Investigateur Principal
Ecole de Santé Publique
Université de Kinshasa

**Concerne** : L'approbation du protocole de l'étude intitulé : « **Effet de la riposte contre Ebola sur la performance des services de santé de routine dans les hôpitaux de l'Est de la République Démocratique du Congo** ».

Monsieur l'Investigateur Principal,

Le Bureau du comité d'éthique de l'Ecole de Santé Publique de l'Université de Kinshasa examiné attentivement votre protocole de recherche dont le sujet est repris en marge. Il en résulte que l'étude que vous comptez entreprendre est pertinente et peut contribuer à l'amélioration de l'état de santé des communautés par le renforcement des systèmes de santé.

Ainsi, vu que le principe éthique relatif au respect de la personne est pris en compte, le Bureau du Comité approuve le protocole et autorise la mise en œuvre de l'étude pour la période allant du 10 février 2020 au 09 février 2021.

Cependant, il est porté à votre connaissance que toute modification apportée au présent protocole devra au préalable obtenir l'approbation du comité d'éthique. Bien plus, tout incident devra lui être notifié sans délai.

Veuillez agréer, Monsieur l'Investigateur Principal, l'expression de notre considération distinguée.

ESP UNIKIN
Scientia Splendet et Conscientia
COMITE D'ETHIQUE

**Professeur Dr Patrick Kayembe Kalambayi**

Président du comité d'éthique

*Université de Kinshasa, Ecole de Santé Publique, B.P 11850 Kin I, E-mail: espsec_unikin@yahoo.fr ; www.espkinshasa.net, Contact : +243 817493194, 851463831*
