# Supplementary material for: Barriers and facilitators to healthcare facility utilization by non-Ebola patients during the 2018–2020 Ebola outbreak in the Democratic Republic of Congo
Source: Glob Health Res Policy. 2024 Nov 19;9:47. doi: 10.1186/s41256-024-00387-6 (PMC11575170; doi:10.1186/s41256-024-00387-6)
Supplement: Supplementary file 10 — Additional file 10. Ethical committee letter English. [file 41256_2024_387_MOESM10_ESM.pdf]

DEMOCRATIC REPUBLIC OF CONGO

Ministry of Higher and University Education

Université de Kinshasa

SCHOOL OF PUBLIC HEALTH

ETHICS COMMITTEE Approval No: ESP/CE/11/2020

Kinshasa, February 05, 2020

KYOMBA KALOMBE Gabriel

Principal Investigator

School of Public Health

University of Kinshasa

Concerned: Approval of the protocol of the study entitled; "**Effect of the response to Ebola outbreak on the performance of routine health services in hospitals in eastern Democratic Republic of Congo**".

Mr Principal Investigator,

The office of the Ethics Committee of the Kinshasa School of Public Health (Université de Kinshasa) has carefully examined your research project, as outlined above. Our conclusion is that the study you intend to undertake is relevant and can contribute to improving the communities health status by strengthening health systems.

Therefore, considering that the ethical principle relating to respect for the person is fully taken into account, the Committee office approves the project and approves the implementation of the study for the period from February 10, 2020 to February 9, 2021.

Please note that any changes to the present project must first be approved by the Ethics Committee. Moreover, any incident must be notified to him without delay.

Please accept, Mr. Principal Investigator, the expression of our highest consideration.

Professor Dr. Patrick Kayembe Kalambayi

Chairman of the Ethics Committee
